# The *Polycomb* Orthologues in Teleost Fishes and Their Expression in the Zebrafish Model

**DOI:** 10.3390/genes11040362

**Published:** 2020-03-27

**Authors:** Ludivine Raby, Pamela Völkel, Xuefen Le Bourhis, Pierre-Olivier Angrand

**Affiliations:** University Lille, CNRS, Inserm, CHU Lille, Centre Oscar Lambret, UMR 9020 - UMR 1277 - Canther - Cancer Heterogeneity, Plasticity and Resistance to Therapies, F-59000 Lille, France; ludivine.raby.etu@univ-lille.fr (L.R.); pamela.voelkel@univ-lille.fr (P.V.); xuefen.le-bourhis@univ-lille.fr (X.L.B.)

**Keywords:** polycomb group proteins, CBX, Teleost fishes, zebrafish, phylogenetic analysis

## Abstract

The Polycomb Repressive Complex 1 (PRC1) is a chromatin-associated protein complex involved in transcriptional repression of hundreds of genes controlling development and differentiation processes, but also involved in cancer and stem cell biology. Within the canonical PRC1, members of Pc/CBX protein family are responsible for the targeting of the complex to specific gene loci. In mammals, the Pc/CBX protein family is composed of five members generating, through mutual exclusion, different PRC1 complexes with potentially distinct cellular functions. Here, we performed a global analysis of the *cbx* gene family in 68 teleost species and traced the distribution of the *cbx* genes through teleost evolution in six fish super-orders. We showed that after the teleost-specific whole genome duplication, *cbx4*, *cbx7* and *cbx8* are retained as pairs of ohnologues. In contrast, *cbx2* and *cbx6* are present as pairs of ohnologues in the genome of several teleost clades but as singletons in others. Furthermore, since zebrafish is a widely used vertebrate model for studying development, we report on the expression of the *cbx* family members during zebrafish development and in adult tissues. We showed that all *cbx* genes are ubiquitously expressed with some variations during early development.

## 1. Introduction

Teleosts, with over 29,000 species, represent the largest and most diverse infraclass (*Teleostei*) of vertebrates accounting for about half of the living vertebrates and more than 96% of all fish species [1,2,3]. Gene and gene family early sequencing revealed the existence of a whole-genome duplication event that occurred in the teleost lineage about 320 Myr ago, before its diversification [4,5,6], and known as the teleost genome duplication (TGD). Ohnologous genes produced by whole genome duplication are presumably redundant and one copy is subsequently lost randomly [7]. Less frequently, both ohnologues are maintained due to accumulation of mutations leading to neofunctionalization, subfunctionalization or subneofunctionalization of one or two of the ohnologues [8]. Following the TGD in the teleost ancestor, about 15% to 20% of the ohnologues were retained as pairs [9]. Interestingly, an asymmetric acceleration of evolutionary rate is observed for one of the ohnologues allowing a possible neofunctionalization and/or subfunctionalization. Furthermore, the teleost evolution is associated with major interchromosomal rearrangements having occurred after the TGD [10,11] and to the fact that teleost genes evolved faster compared to their tetrapode orthologues [12]. These genomic characteristics, sub/neofunctionalization of the retained ohnologues, large interchromosomal rearrangements and rapid evolutionary rates, are thus believed to be, in part, responsible for the remarkable diversity of the teleost fishes in terms of morphology, physiology, behaviors and adaptations.

Here, we report on the study of the Polycomb orthologues in teleosts. Initially discovered in Drosophila, Polycomb repression is a conserved epigenetic mechanism involved in the repression of hundreds of genes implicated in various biological processes such as the regulation of tissue homeostasis, maintenance and lineage specification of stem cells, cell differentiation and development [13,14,15,16]. Polycomb repression is ensured by at least two chromatin-modifying protein complexes, named Polycomb Repressive Complex 1 and 2 (PRC1 and PRC2). According to the canonical pathway, the PRC2 complex is recruited at defined chromatin locations and initiates gene silencing by catalyzing trimethylation of histone H3 at lysine 27 (H3K27me3). The PRC1 protein complex is then recruited to the target regions by binding to H3K27me3 epigenetic marks, catalyzes the monoubiquitinylation of histone H2A at lysine 119 (H2AK119ub1) and induces local chromatin compaction to maintain gene silencing [17,18,19,20,21]. In *Drosophila*, the PRC2 core complex is composed of Suppressor of zeste 12 [Su(z)12], Extra sex combs (Esc) and Enhancer of zeste [E(z)], which is the catalytic subunit of the complex [22,23]. The canonical PRC1 core complex comprises the Polycomb protein itself (Pc), Polyhomeotic (Ph), Posterior sex combs (Psc) and Sex combs extra (Sce). Sce deposits the H2AK119ub1 epigenetic mark, whereas Pc binds to H3K27me3 through its chromodomain [24,25]. In mammals, PRC1 organization appears to be more complex than in Drosophila because there are several orthologues for each of its components [26,27]. Actually, mammalian genomes code for five orthologues for Pc (CBX2, 4, 6, 7 and 8), six Psc orthologues (BMI1, PCGF1, 2, 3, 5, and 6), three Ph orthologues (PHC1, 2 and 3) and two Sce orthologues (RING1 and RNF2). Due to combinatorial permutations, distinct PRC1 complexes, different in their subunit composition, are present in mammalian cells [28,29,30,31,32,33]. Functionally, PRC1 diversity depends largely on the Pc orthologue present in the complex since it has been shown that the different CBX proteins could bind in part, to different targets [33,34,35,36]. Indeed, differentiation of embryonic or hematopoietic stem cells relies on a dynamic switch in the expression of the *CBX* genes and on the identity of the Polycomb paralogues present in the PRC1 [33,35,36].

In a previous study, we showed that some PRC1 subunit members are not similarly retained in all teleost species [37]. In zebrafish (*Danio rerio*), the Psc orthologues *pcgf1* and *pcgf6* exist as singletons, *bmi1a/b* and *pcgf5a/b* as pairs, whereas *pcgf2* and *pcgf3* are absent. In contrast, in medaka (*Oryzias latipes*, strain HdrR), *bmi1*, *pcgf2*, *pcgf3*, *pcgf5* and *pcgf6* are all present as singletons but *pcgf1* is absent. Similarly, the medaka genome contains two Sce orthologues (*ring1* and *rnf2*), whereas only *rnf2* is present in zebrafish [37]. However, it is not known whether the Pc orthologues are all maintained nor whether the *CBX* genes are present as singletons or pairs in teleosts. Thanks to recent advances in teleost genome sequencing, we conducted the analysis of the Pc/CBX gene family in 68 teleost species that belong to 21 different orders. Furthermore, we report here on the expression of the Pc/CBX family members during zebrafish development and in adult tissues since this teleost is a widely used vertebrate model for studying development and gene functions.

## 2. Materials and Methods

### 2.1. In Silico Identification and Analyses of Pc/CBX Sequences in Databases

TBLASTN searches in the NCBI (National Center for Biotechnology, Bethesda, Maryland, USA) and Ensembl databases were used to retrieve the teleost cbx genes. The human (CBX2_HUMAN, Q14781; CBX4_HUMAN, O00257; CBX6_HUMAN, O95503; CBX7_HUMAN, O95931 and CBX8_HUMAN, Q9HC52) and zebrafish (Cbx2, F1Q982_DANRE; Cbx4, B7ZUU4_DANRE; Cbx6a, XP_021325056.1; Cbx6b, X1WBY5_DANRE; Cbx7a, F1RBK7_DANRE; Cbx7b, E7F8F2_DANRE; Cbx8a, Q6TH37_DANRE and Cbx8b, A8KC45_DANRE) reference sequences were used as queries. Teleost Cbx3 sequences were identified through TBLASTN searches using zebrafish Cbx3a, F1QNF2_DANRE and Cbx3b, E9QDB7_DANRE reference protein sequences as queries. Sequence alignments were performed using the Clustal Omega [38] and ClustalW [39] algorithms. Phylogeny was determined by aligning the teleost Cbx sequences with the MUSCLE algorithm [40] included in MEGA X (Pennsylvania State University, USA) [41]. Phylogenetic trees were produced by the Maximum Likelihood (ML) method and JTT matrix-based model [42], the Neighbor-Joining (NJ) method [43] and the Maximum Parsimony (MP) using the Subtree-Pruning-Regrafting (SPR) algorithm [44] in MEGA X [41].

SMART and Pfam protein domains were identified using the Simple Modular Architecture Research Tool [45]. Consensus patterns were drawn in logo format using WebLogo [46].

Syntenic group identification at the *cbx* gene loci and vicinal genes in teleost genomes were assessed by manual chromosome walking and reciprocal BLAST searches using the NCBI Gene database (National Center for Biotechnology, Bethesda, MD, USA).

### 2.2. Zebrafish Maintenance, Embryo Preparation and Animal Ethics Statements

Zebrafish from the TU strain were kept at 27.5 °C in a 14/10 h light/dark cycle. The evening before spawning, males and females were separated into individual breeding tanks (Tecniplast). Spontaneous spawning occurred the following morning when the plastic divider is removed. Embryos were collected and staged according to Kimmel et al. [47]. The chorions were removed from embryos by action of 1% pronase (Sigma, St. Louis, MO, USA) for 1 min. Zebrafish embryos were fixed overnight in 4% paraformaldehyde in PBS (phosphate-buffered saline, Euromedex, Souffelweyersheim, France), dehydrated gradually to 100% methanol and kept at −20 °C.

Zebrafish were maintained in compliance with the French and European Union guidelines for the handling of laboratory animals (Directive 2010/63/EU of the European Parliament and of the Council of 22 September 2010 on the protection of animals used for scientific purposes). The experimental procedures carried out on zebrafish were reviewed and approved by the local Ethics Committee, CEEA 75 Nord Pas-de-Calais and the French Ministry of Higher Education and Research (APAFiS approval number 13527-2018011722529804_v3).

### 2.3. Whole-Mount In Situ Hybridization

Antisense-RNA probes were synthesized using the DIG RNA Labeling Kit (SP6/T7) (Roche, 11175025910) according to the manufacturer’s instruction. The following IMAGE cDNA clones purchased at imaGenes GmbH (Berlin, Germany), were used as templates: *cbx2*, cDNA clone MGC:103563 IMAGE:7241894; *cbx7a*, cDNA clone MGC:110152 IMAGE:7289412; *cbx8a*, cDNA clone MGC:153854 IMAGE:8001741; *cbx8b*, cDNA clone MGC:111978 IMAGE:7399500. *cbx4*, *cbx6a*, *cbx6b* and *cbx7b* antisense probes were generated using RT-PCR from total mRNA extracted from zebrafish larvae at 5 dpf using the RNeasy Mini Kit (Qiagen, Hilden, Germany). After Reverse Transcription, cDNAs were amplified by PCR using the probe-specific primers, coupled to the T7 sequence for forward primers and the SP6 sequence for reverse primers.

The primers used for probe generation were:

ISH_cbx4a1_508F: 5′-TAATACGACTCACTATAGGGGTCGAGCAGCATATGAGCGG-3′
ISH_cbx4a1_1921R: 5′-GATTTAGGTGACACTATAGAATTTGCCAGGGGCATGAAT-3′
ISH_cbx6a2_1210F: 5′-TAATACGACTCACTATAGGGGTGGGTCGAGCTTCGGTCTGCC-3′
ISH_cbx6a2_1460R: 5′-GATTTAGGTGACACTATAGGCAGTTGTGGTGGGAGAGTTTGGGT-3′
ISH_cbx6b_1037F: 5′-TAATACGACTCACTATAGGGCCCCTTCGACTAAGGCCCCTGA-3′
ISH_cbx6b_1633R: 5′-GATTTAGGTGACACTATAGGCTTGACCACATCCAAGCCCAACC-3′
ISH_cbx7b_304F: 5′-TAATACGACTCACTATAGGGGCTCATCTGCCACTCTCGCTGGA-3′
ISH_cbx7b_692R: 5′-GATTTAGGTGACACTATAGAAGCCTCTCGCAACCAAAGCCTCT-3′

In situ hybridization was performed as described by Thisse and Thisse [48]. Briefly, the fixed embryos were rehydrated and permeabilized with 10 μg/mL proteinase K for 30 s (1-cell stage embryos or 10 min (24 and 48 hpf embryos) at room temperature. Ten to 15 embryos from each time point were hybridized with digoxigenin-labeled antisense RNA probes at 70 °C. After extensive washing, the probes were detected with anti-digoxigenin-AP Fab fragment (Roche Diagnostics, 1093274, diluted at 1:10,000), followed by staining with BCIP/NBT (5-bromo-4-chloro-3-indolyl-phosphate/nitro blue tetrazolium) alkaline phosphate substrate. The embryos were imaged using a Leica MZ10F stereomicroscope equipped with a Leica DFC295 digital camera.

### 2.4. RNA Extraction and RT-PCR

Total RNAs were purified from 1 hpf, 3 hpf, 6 hpf, 1 dpf, 2 dpf, 3 dpf, 4 dpf and 5 dpf wild-type embryos or larvae, as well as from adult tissues using Trizol, as previously described [49]. cDNA was synthesized using Superscript III (18080-044, Invitrogen, Carlsbad, CA, USA) following the manufacturer’s instructions. A quantity of 1 μg of total RNAs were used to perform the reverse transcription experiments. Primers used were:

cbx2_984F: 5′-TCAAAGGTCAGCATCTCTCCCGA-3′
cbx2_1111R: 5′-TCAAATCAGTCCCGTTTCCCTGC-3′
cbx4a1_656F: 5′-GGAACCTCCCTCCAGCCCTAC-3′
cbx4a1_785R: 5′-AGTGTCCGCTCTGCTTTGTTAGC-3′
cbx6a2_1295F: 5′-CCGATTGGCACCCTGAAATGGC-3′
cbx6a2_1461R: 5′-TGCAGTTGTGGTGGGAGAGTTTGG-3′
cbx6b_1275F: 5′-ACCAGAGGTGCCCGTGTCAGA-3′
cbx6b_1437R: 5′-AGAAGAAGCAGAAGGCGGATGGC-3′
cbx7_1691F: 5′-CCTCACCTGCTTGTCCTGTAACCA-3′
cbx7_1933R: 5′-AGGCTCGGCTTTGATTTCTGCC-3′
cbx7b_304F: 5′-GCTCATCTGCCACTCTCGCTGG-3′
cbx7b_428R: 5′-TCCCTTCTGTCCCACTCTTCCTCA-3′
cbx8a_831F: 5′-GGTTGGCTCTGGTGGTGCCATAA-3′
cbx8a_977R: 5′-TTCAAACAAGGCAGCCAGGACTG-3′
cbx8b_787F: 5′-AGCAACGTCATTCCAAGCAGGAGT -3′
cbx8b_1045R: 5′-AGGGTCTCCAAGCATCTTCCACC -3′
Dr_cDNA_ube2a_F: 5′-TGACTGTTGACCCACCTTACAG-3′
Dr_cDNA_ube2a_R: 5′-CAAATAAAAGCAAGTAACCCCG-3′
Dr_beta-actin_cDNA_5: 5′-CGTGACATCAAGGAGAAGCT-3′
Dr_beta-actin_cDNA_3: 5′-ATCCACATCTGCTGGAAGGT-3′

PCR reactions were performed as follow: 95 °C 4 min, (95 °C 45 s, 65 °C 45 s, 72 °C 1 min) 35 cycles, 72 °C 10 min.

RT-PCR experiments were performed from at least two independent RNA extraction samples.

## 3. Results

### 3.1. In Silico Identification of Teleost Pc Orthologues

Polycomb (Pc) and its CBX mammalian orthologues interact with H3K27me3 epigenetic marks via the chromodomain (SMART, SM00298; Pfam, PF00385) [34], whereas the Pc box at the C-terminus (Pfam, PF17218) is involved in transcriptional silencing and binding to other PRC1 components such as RNF2 [50]. Adjacent to the chromodomain, all CBX protein have a DNA binding motif, the AT-hook (SMART, SM000384; Pfam, PF02178) (in CBX2) or an AT-hook-like motif (in the other four CBX proteins, CBX4, CBX6, CBX7 and CBX8) [51]. Less conserved sequences in the middle of the CBX proteins could play a role in specifically directing each CBX family member to distinct regions of the chromatin [51,52].

**Figure 1:**
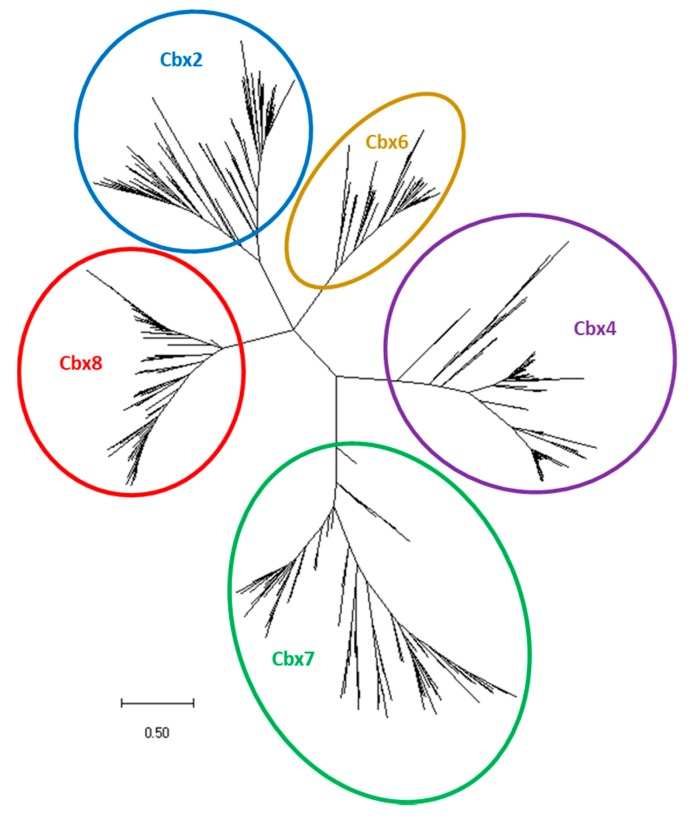
Unrooted phylogenetic tree of the evolutionary relationships between teleost Pc orthologues. The tree generated using the Maximum Likelihood methods and JTT matrix-based model, with the highest log likelihood (-1307026.38) is shown. The tree is drawn to scale, with branch lengths measured in the number of substitutions per site. This analysis involves 558 amino acid sequences (116 for Cbx2, 129 for Cbx4, 72 for Cbx6, 117 for Cbx7 and 124 for Cbx8) with a total of 1529 positions in the final dataset. Evolutionary analyses were conducted in MEGA X.

We applied TBLASTN searches in the NCBI and Ensembl databases using the human and zebrafish Pc orthologues as queries, to identify the corresponding genes in 67 teleost species (Table A1). A total of 689 genes from 68 teleosts including zebrafish, were identified. Based on sequence alignments and phylogenetic analyses (Figure 1, Supplementary File S1, Supplementary File S2), these Pc orthologues were associated to one of the paralogous class CBX2, CBX4, CBX6, CBX7 or CBX8 (Supplementary Table S1). In contrast to Psc/PCGF and Sce/RING1 orthologues that are not all present in fishes including zebrafish and medaka [37], the five *cbx* paralogues, *cbx2*, *cbx4*, *cbx6*, *cbx7* and *cbx8*, are maintained in all sequenced teleost genomes. In many cases, but not systematically, the *cbx* genes are even retained as pairs of ohnologues (Supplementary Table S1).

**Figure 2:**
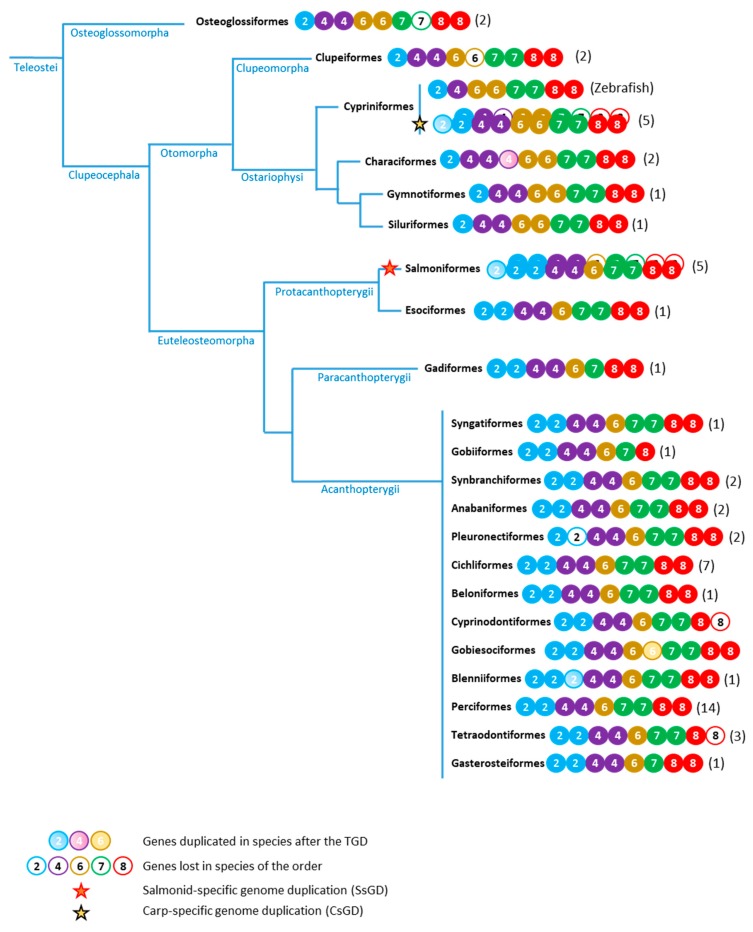
Simplified cladogram of the teleost fishes showing the content of the Pc/cbx genes in different orders. *Cbx2*, *cbx4*, *cbx6*, *cbx7* and *cbx8* genes are shown as solid circle blue, violet, yellow, green and red, respectively. Empty circles indicate that a given *cbx* gene is absent in at least one specie in the order. Circles filled with clear colors show duplicated genes in at least one specie in the order. Stars indicate a recent whole genome duplication in the salmoniformes (red star—salmonid-specific 4th round of genome duplication, SsGD) and in the carp lineage of cypriniformes (black star—carp-specific genome duplication, CsGD). The number of species studied in each order is in brackets.

To investigate the evolution of the *cbx* gene content in teleosts, a cladogram of the teleost fishes [53,54,55,56] showing the *cbx* gene content in different fish orders, has been drawn (Figure 2). From this representation, it appears that *cbx7* and *cbx8* are present as pairs of ohnologues in teleost. In contrast, *cbx2* and *cbx6* are retained as singleton in the genome of particular clades of teleost fishes. Following the TGD, one of the two *cbx2* ohnologues has been lost in Osteoglossomorpha and Otomorpha, whereas one *cbx6* ohnologue has been lost in Euteleosteomorpha. Finally, the loss of a *cbx4* ohnologue occurred later in teleost evolution since it just concerns fishes from the order of the Cypriniformes and zebrafish is so far the only teleost containing *cbx4* as a singleton in its genome.

### 3.2. The cbx6 Paralogue in Teleost Fishes

**Figure 3:**
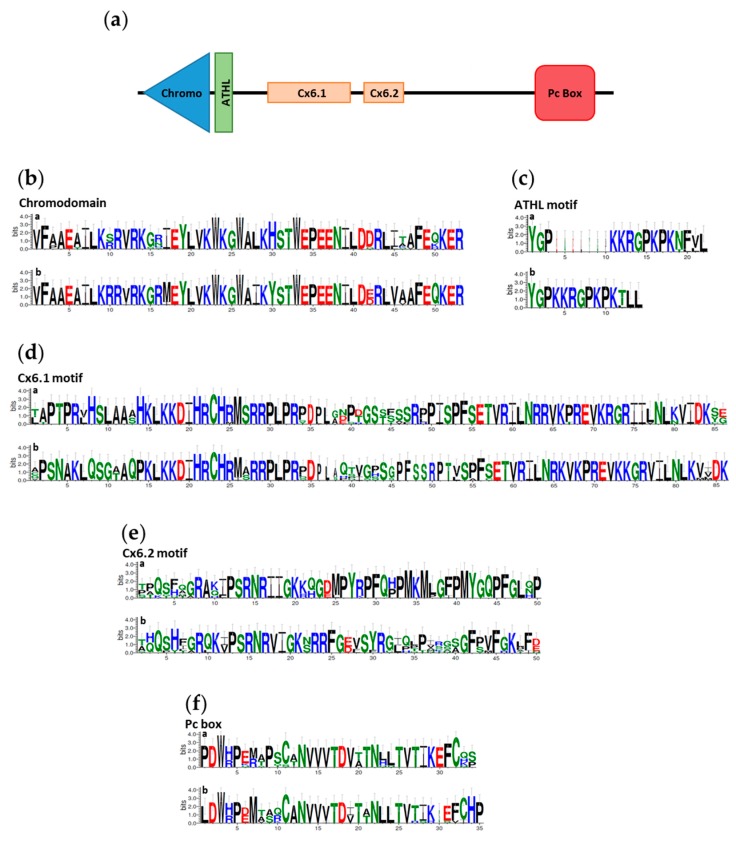
The Cbx6 conserved protein motifs. (**a**) Schematic representation of Cbx6 and its conserved regions. The sequences of the chromodomain (**b**), the ATHL (**c**), Cx6.1 (**d**), Cx6.2 (**e**) motifs and the Pc box (**f**) is shown for the Cbx6a (a: up) and Cbx6b (b: down) proteins. The height of amino acids is proportional to the frequency of occurrence (degree conservation) in each position and drawn in a logo format. Basic amino acids are in blue, hydrophobic in black, polar in green and acidic in red.

Our phylogenetic analysis shows that in Clupeomorpha and Ostariophysi fishes, *cbx6* is found as a pair of ohnologues hereafter called *cbx6a* or *cbx6b* (Supplementary Figure S1). While Cbx6a and Cbx6b belong to distinct branches of the phylogenetic Cbx6 tree, protein sequence alignments showed that Cbx6a and Cbx6b share high levels of similarity within conserved motifs such as the chromodomain, the ATHL motif, the Cx6.1 motif and the Pc box (Figure 3, Supplementary Figure S2). In contrast, the Cx6.2 motif, as well as sequences outside of the conserved motifs, present lower levels of similarity between Cbx6a and Cbx6b proteins.

Previous synteny analyses at genomic levels in zebrafish and tetraodon showed that the chromosomal correspondence of duplicated gene pairs arising from the TGD has been extensively preserved in doubly conserved synteny blocks, while local gene order has been largely scrambled [57,58]. Examination of the genes surrounding the *cbx6* ohnologues reveals that *cbx6a* is flanked at its 5′ by *pla2g6* coding for the phospholipase A2 group VI in Ostariophysi fishes from the orders Cypriniformes (zebrafish, goldfish), Chariciformes (Mexican cavefish), Siluriformes (channel catfish) and Gymnotiformes (electric eel), as well as in Clupeomorpha fishes (Atlantic herring) (Supplementary Figure S3). In contrast, the *baiap2l2* gene encoding the BAR/IMD domain containing adaptor protein 2 like 2 is located at the 5′ position of *cbx6b*, thus defining distinct doubly conserved synteny blocks for *cbx6a* and *cbx6b*. 

**Figure 4:**
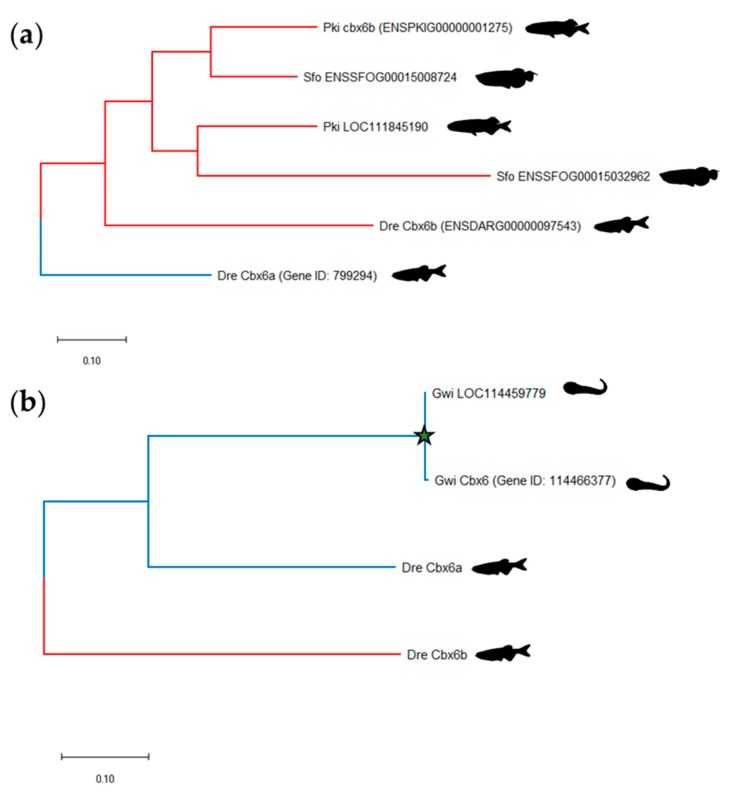
Phylogenetic trees of the evolutionary relationships between Cbx6 proteins from teleost fishes. (**a**) Phylogenetic tree of the evolutionary relationships between zebrafish (Dre, *Danio rerio*) Cbx6a and Cbx6b proteins with their orthologues in elephant fish (Pki, *Paramormyrops kingsleyae*) and Asian bonytongue (Sfo, *Scleropages formosus*). (**b**) Phylogenetic tree of the evolutionary relationships between zebrafish Cbx6 proteins and their orthologues in blunt-snouted clingfish (Gwi, *Gouania willdenowi*). The evolutionary history was inferred using the Neighbor-Joining method conducted in MEGA X. The star illustrates the recent duplication of the cbx6 gene in *Gouania willdenowi*.

Among the Osteoglossomorpha, the elephant fish (*Paramormyrops kingsleyae*) and the Asian bonytongue (*Scleropages formosus*) are so far the only two species that have fully sequenced genomes [59,60]. Like Ostariphysi and Clupeomorpha, the *cbx6* gene exists as pairs of ohnologues in the elephant fish and in the Asian bonytongue genomes (Figure 2, Supplementary Table S1). Phylogenetic analyses revealed that the Osteoglossomorpha Cbx6 ohnologues are more closely related to each other and to the Ostariphysi and Clupeomorpha Cbx6b rather than to Cbx6a (Figure 4A, Supplementary Figure S1). However, syntenic studies identify that the Osteoglossomorpha *cbx6* ohnologues are located within the same distinct doubly conserved synteny blocks as those harboring *cbx6a* and *cbx6b* in Ostariphysi and Clupeomorpha (Supplementary Figure S3). Thus, consistent with the teleost cladogram, the two Osteoglossomorpha *cbx6* ohnologues originate from the TGD but are less divergent to each other than *cbx6a* to *cbx6b*.

The study of the *cbx6* paralogue content in teleost also revealed that *cbx6* is present as a singleton in Euteleosteomorpha fishes including Protacanthopterygii, Paracanthopterygii and Acanthopterygii (Figure 2, Supplementary Table S1), indicating that one *cbx6* ohnologue arising from the TGD has been lost in this fish clade. The only Acanthopterygii that has its genome sequenced and possesses a pair of cbx6 genes is the blunt-snouted clingfish (*Gouania willdenowi*, order Gobiesociformes). However, both the phylogenetic analyses (Figure 4B) and the syntenic studies indicating that one *cbx6* is located in a highly rearranged region of Chromosome 1 (Supplementary Figure S3), suggesting that the second copy of *cbx6* arose from a recent duplication event.

### 3.3. The cbx2 Paralogue in Teleost Fishes

**Figure 5:**
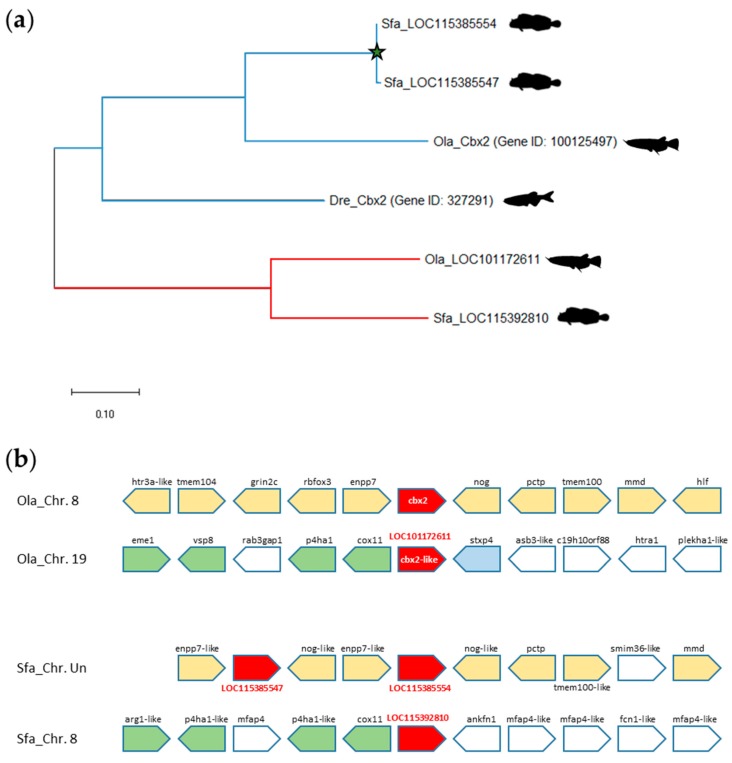
The *cbx2* paralogues in the jewelled blenny (*Salarias fasciatus*). (**a**) Phylogenetic tree of the evolutionary relationships between zebrafish (Dre, *Danio rerio*) Cbx2 protein and its orthologues in medaka (Ola, *Oryzia latipes*) and jewelled blenny (Sfa, *Salarias fasciatus*). The evolutionary history was built using the Neighbor-Joining method conducted in MEGA X. (**b**) Blocks of synteny at the *cbx2* locus in medaka and jewelled blenny. The *cbx2* genes are shown in red, while orthologous gene loci are highlighted with different colors. Chromosomal locations of *cbx2* and the neighboring gene loci were drawn based on information from the NCBI Gene server. Chromosome (Chr.) numbers are indicated. Un, unplaced scaffold.

A search for the *cbx2* paralogues in the sequenced teleost genomes revealed that *cbx2* is present as a singleton in Osteoglossomorpha and Otomorpha fishes, but exists as a pair of ohnologues in Euteleosteomorpha (Figure 2, Supplementary Table S1). This suggests that after the TGD leading to the duplication of *cbx2*, one of the ohnologues was lost at least twice independently in the Osteoglossomorpha and in the Otomorpha fish lineages. Within the Acanthopterygii, the jewelled blenny (*Salarias fasciatus*, order Blenniformes) possesses three copies of *cbx2* in its genome (Supplementary Table S1). However, phylogenetic analyses revealed that two of these copies (LOC115385554 and LOC115385547) are highly similar and might arise from a recent gene duplication event (Figure 5A). 

The synteny studies showed that the *cbx2* ohnologues are associated to distinct doubly conserved synteny blocks in Acanthopterygii including in the medaka fish model (Figure 5B, Supplementary Figure S4). In jeweled blenny, one of these blocks was being subjected to rearrangements generating a second *cbx2* gene within the synteny block. Thus, it is likely that the additional *cbx2* copy in the jeweled blenny genome results from a recent intrachromosomal rearrangement. Similar rearrangements are also found in species of the Cypriniformes and Salmoniformes orders. 

Cypriniformes is the largest group of freshwater fishes comprising about 4300 described species including zebrafish, goldfish, carps, barbels, minnows, loaches and suckers [61]. Among them, several species were subject to an additional whole-genome duplication event called the carp-specific genome duplication (CsGD). Indeed, while the zebrafish diploid genome is composed of 50 chromosomes, the diploid status of common carp (*Cyprinus carpio*), goldfish (*Carassius auratus*) and Chinese barbels (*Sinocyclocheilus anshuiensis, S. grahami* and *S. rhinocerous*) is supported by a double-size karyotype consisting of approximately 100–104 chromosomes [62,63,64]. In zebrafish, *cbx2* is present as a singleton and consistent with the CsGD event, there are two *cbx2* gene copies in common carp and Chinese barbels (Supplementary Table S1). However, in goldfish, an additional *cbx2* copy is identified and genomic analyses showed that two goldfish *cbx2* genes (LOC113067828 and LOC113067392) are located in the same linkage group (LG28B, position NC_039293.1) (Supplementary Figure S4) as the result of an intrachromosomal rearrangement. Similarly, within the Protacathopterygii, the northern pike (*Esox lucius*, order Esociformes) has a karyotype composed of about 50 chromosomes [65]. An additional whole-genome duplication event, called the salmonid-specific genome duplication (SsGD), occurred in the common ancestor of Salmoniformes after their divergence from Esociformes [66,67]. Then, while the Northern pike genome harbors a pair of *cbx2* ohnologues, most of the Salmonidae including rainbow trout (*Oncorhynchus mykiss*), Coho salmon (*Oncorhynchus kisutch*), Arctic char (*Salvelinus alpinus*) and huchen (*Hucho hucho*) host four *cbx2* genes in their genome because of the SsGD (Supplementary Table S1). However, the Atlantic salmon (*Salmo salar*) possesses a fifth *cbx2* copy in its genome. Remarkably, two of these Atlantic salmon *cbx2* genes (LOC106606958 and LOC106606946) are located on the same chromosome (ssa06, position NC_027305.1) (Supplementary Figure S4), suggesting that the additional *cbx2* copy appeared due to a recent chromosomal rearrangement.

### 3.4. The cbx4, cbx7 and cbx8 Paralogues in Teleost Fishes

Our TBLASTN searches in the sequenced teleost genomes showed that the three Pc orthologues *cbx4*, *cbx7* and *cbx8* are present as pairs of ohnologues in all teleost clades (Supplementary Table S1, Figure 2). One possible exception could concern the *cbx7* paralogue in Paracanthopterygii. Within the Paracanthopterygii super-order, Atlantic cod (*Gadus morhua*, order Gadiformes) is the only specie for which whole-genome sequencing data are available (Ensembl, assembly gadMor1) and in its genome, *cbx7* is found as a singleton. However, a cod-specific loss of one *cbx7* ohnologue due to a particular chromosomal rearrangement in this specie cannot be ruled out. Alternatively, the failure in the identification of the second *cbx7* homologue could be the result of a technical artefact such as sequencing or gene prediction information missing. Then, a definitive conclusion about the presence of *cbx7* as a singleton or a pair of ohnologues in Paracanthopterygii could be made when genomic information will be available for other species of the clade.

**Figure 6:**
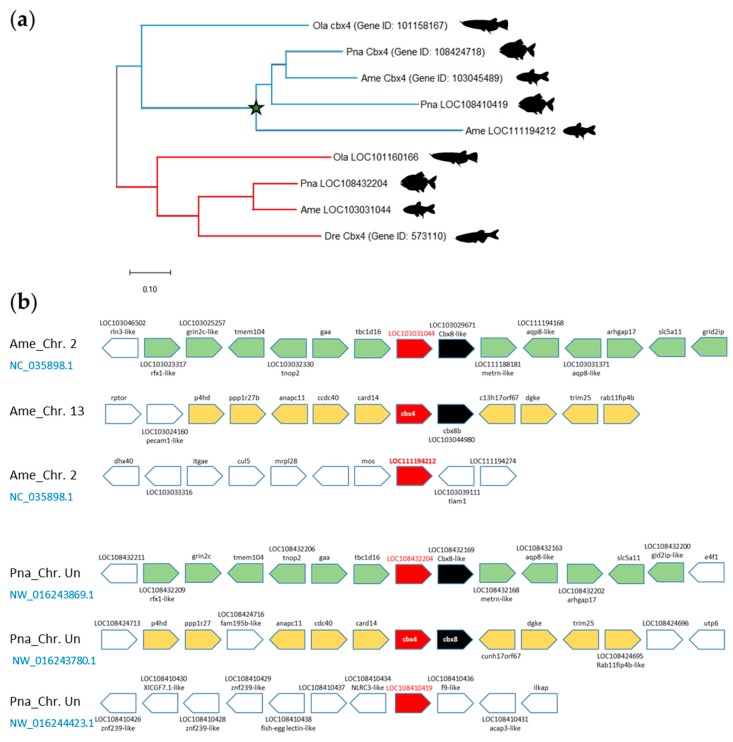
The *cbx4* paralogues in Characiformes. (**a**) Phylogenetic tree of the evolutionary relationships between zebrafish (Dre, *Danio rerio*) Cbx4 protein and its orthologues in medaka (Ola, *Oryzia latipes*), Mexican cavefish (Ame, *Astyanax mexicanus*) and red-bellied piranha (Pna, *Pygocentrus nattereri*). The evolutionary history was built using the Neighbor-Joining method conducted in MEGA X. The star indicated a possible *cbx4* gene duplication having occurred in Characiformes. (**b**) Blocks of synteny at the *cbx4-cbx8* locus in Mexican cavefish and red-bellied piranha. The *cbx4* genes are shown in red, *cbx8* in black, while orthologous gene loci are highlighted with different colors. Chromosomal locations of *cbx4*, *cbx8* and the neighboring gene loci were drawn based on information from the NCBI Gene server. Chromosome (Chr.) numbers are indicated. Un, unplaced scaffold.

The *cbx4* paralogue is even present as three copies in the Mexican cavefish (*Astyanax mexicanus*) and red-bellied piranha (*Pygocentrus nattereri*) genomes, two species of the teleost order Characiformes. Genomic analysis reveals a remarkable conservation of *cbx4* chromosomal organization (Supplementary Figure S5). First, *cbx4* is located immediately 5′ to *cbx8*. This feature is not specific to teleost, since it is also the case in mammals including in human [37]. Second, the *cbx4-cbx8* ohnologous loci are associated to two recognizable—with distinct signatures—but conserved synteny blocks. The first *cbx4-cbx8* locus is flanked by the genes *card14* (caspase recruitment domain family member 14) and *dgke* (diacylglycerol kinase epsilon), whereas the other is flanked by the genes *tbc1d16* (TBC1 domain family member 1) and *arhgap17* (Rho GTPase activating protein 17). The phylogenic analysis of the Mexican cavefish and red-bellied piranha *cbx4* paralogues suggest that two copies of *cbx4* arise from the duplication of the same *cbx4* orthologue in the two Characiformes species (Figure 6A). In the Mexican cavefish, cbx4 (Gene ID: 10304589) and LOC111194212 derive from the same ancestor gene, while in red-bellied piranha, *cbx4* (Gene ID: 108424718) and LOC108410419 also originate from the same ancestor.

Remarkably, in Mexican cavefish, *cbx4* is associated to the *card14-dgke* synteny block, whereas LOC111194212 local chromosomal organization totally lack the characteristic of the conserved *cbx4-cbx8* synteny blocks (Figure 6B). Similarly, red-bellied piranha, *cbx4* is associated to the *card14-dgke* synteny block whereas LOC108410419 is in a gene region without homology with the conserved *cbx4-cbx8* synteny blocks. The similar properties of the three *cbx4* copies in Mexican cavefish and red-bellied piranha suggest that the additional *cbx4* copy in these species originate from a single *cbx4* duplication event having occurred in the Characiformes before their speciation.

In contrast to Characiformes having three *cbx4* copies, zebrafish contains a single *cbx4* copy in its haploid genome. The loss of one *cbx4* ohnologue in zebrafish is specific and restricted since it does not affect the neighboring *cbx8* gene nor the other genes of the syntenic block (Supplementary Figure S5). Furthermore, this *cbx4* ohnologue loss occurred in the zebrafish lineage after it diverged from the carp lineage. Indeed, the goldfish and the three Chinese barbels from the genus *Sinocyclocheilus* all contain four *cbx4* gene copies consequential to a pair of ohnologues subjected to the CsGD (Supplementary Table S1, Supplementary Figure S5).

### 3.5. The cbx3 Genes in Teleost Fishes

**Figure 7:**
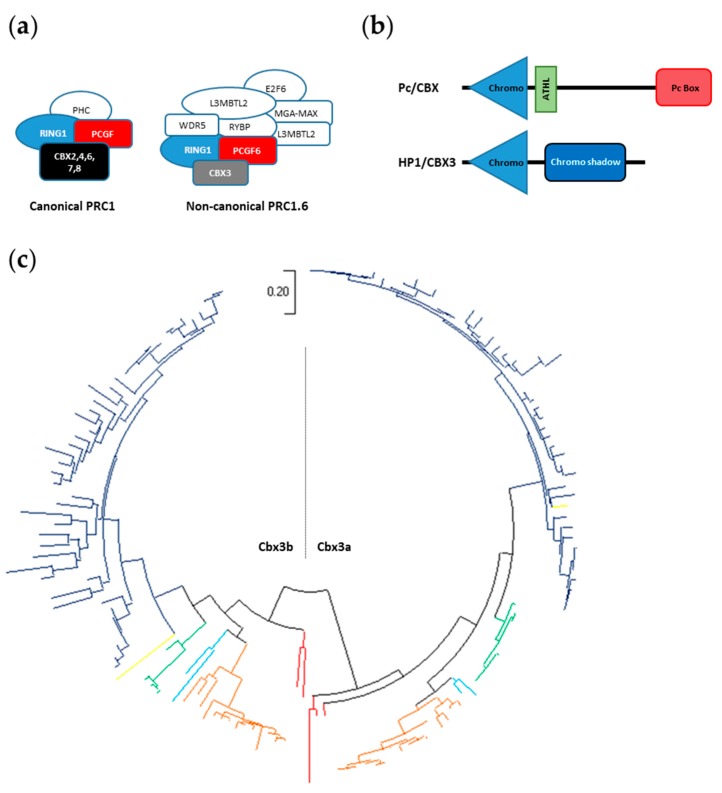
Cbx3 in teleost fish. (**a**) Schematic representation of the canonical PRC1 complex (left) and of the non-canonical PRC1.6 complex (right). (**b**) Schematic representation of the Pc/CBX (CBX2, CBX4, CBX6, CBX7 and CBX8) proteins with the conserved chromodomain, ATHL motif and Pc box (up) and of the HP1/CBX3 protein with the conserved chromodomain and chromo shadow domain (down) (**c**) Phylogenetic tree of the evolutionary relationships between the teleost Cbx3 proteins. The tree generated using the Maximum Likelihood methods and JTT matrix-based model, with the highest log likelihood (-12504.92) is shown. The tree is drawn to scale, with branch lengths measured in the number of substitutions per site. This analysis involves 150 amino acid sequences with a total of 316 positions in the final dataset. Evolutionary analyses were conducted in MEGA X. Both Cbx3a and Cbx3b are present in Osteoglomorpha (red branches), Clupeomorpha (light-blue branches), Ostariophysi (brown branches), Protacanthopterygii (green branches), Paracanthopterygii (yellow branches) and Acanthopterygii (dark-blue branches).

The canonical PRC1 (cPRC1) complex is the functional homologues of *Drosophila* PRC1 composed of Pc, Psc, Ph and Sce. However, in mammal, a heterogeneous group of non-canonical PRC1 (ncPRC1) complexes have also been described [68,69,70]. These ncPRC1 are characterized by the absence of the Pc orthologues CBX2, CBX4, CBX6, CBX7 and CBX7, but the presence of YY1-binding protein (RYBP), or its homolog YAF2 associated to RING1/RNF2, one of the PCGF proteins and various other subunits. Among the different ncPRC1 complexes, PRC1.6 (also named E2F6.com, [71,72]) (Figure 7A) is composed of the transcriptional repressor E2F6 in association with RNF2-PCGF6-RYPB/YAF2. In addition, the complex contains WDR5, the oncoprotein L3MBTL2, the transcription factors MAX and MGA and the chromodomain-containing protein CBX3. In contrast to CBX2, CBX4, CBX6, CBX7 and CBX8 which are orthologous to the *Drosophila* Pc protein, CBX3 is an orthologue of the *Drosophila* protein HP1 / Su(var)205. In addition, Pc orthologues contain a chromodomain, an AT hook/ATHL motif and a Pc box, whereas CBX3 is composed of a chromodomain associated to a chromo shadow domain (Pfam ID: PF00385) (Figure 7B). Finally, while the chromodomain of Pc and its orthlogues recognize H3K27me3, the CBX3 chromodomain preferentially binds to the H3K9me3 epigenetic marks.

Although PRC1.6 function is not known and there is no evidence showing that PRC1.6 exists in teleost fishes, we conducted a TBLASTN search to identify CBX3 orthologues in fishes. From the NCBI and Ensembl databases, we identified 151 *cbx3* genes in the 68 teleost species covered by this study (Supplementary Table S2, Supplementary File S3). Phylogenetic analyses show that *cbx3* is present as a pair of ohnologues *cbx3a* and *cbx3b* in all the teleost clades studied, Osteoglomorpha, Clupeomorpha, Ostariophysi, Protacanthopterygii, Paracanthopterygii and Acanthopterygii (Figure 7C, Supplementary File S3, Supplementary File S4). Then, like *cbx4*, *cbx7* and *cbx8*, *cbx3* has been retained as two gene copies in the teleost genome after the TGD.

### 3.6. Pc Gene Expression in the Zebrafish Model

**Figure 8:**
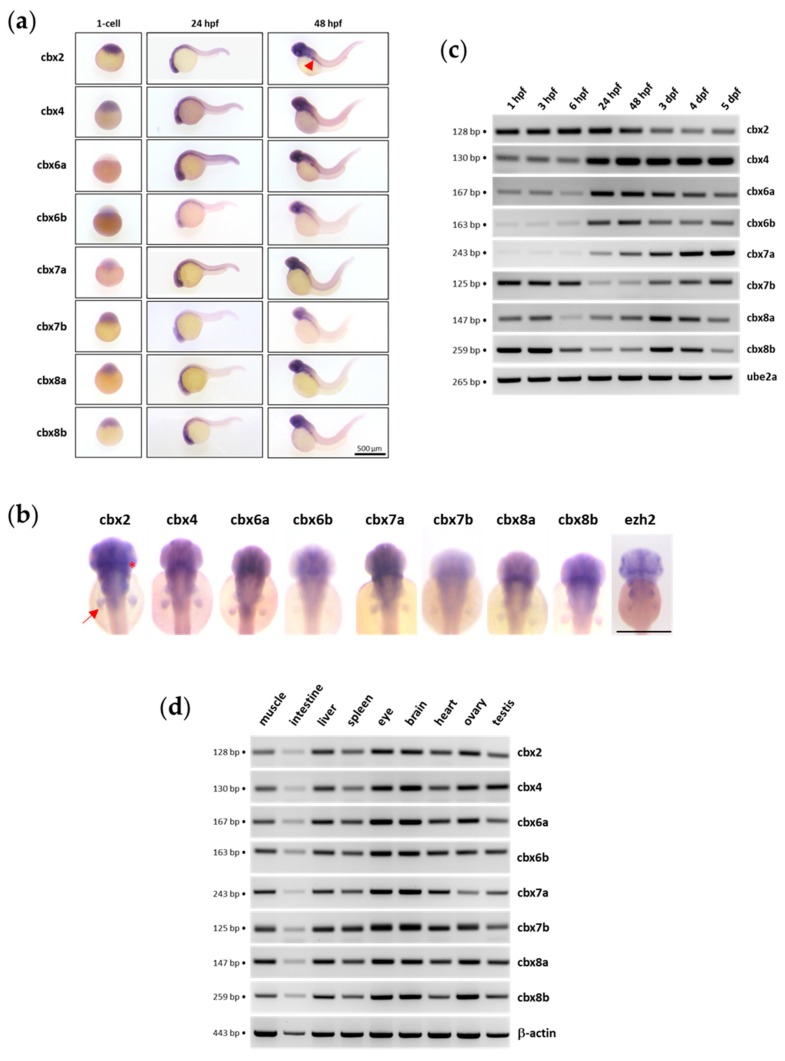
Expression of the *cbx* family members in zebrafish. (**a**) In situ hybridization at the 1-cell stage, 24 hpf and 48 hpf showing maternally provided transcripts (1-cell stage) and zygotic mRNA distribution at 24 and 48 hpf. The red arrowhead shows the gut. (**b**). Dorsal view of the anterior region of embryos at 48 hpf after in situ hybridization. The arrow shows the pectoral fin buds and the asterisk the midbrain–hindbrain boundary. Scale bar is 500 µM. (**c**) RT-PCR experiment showing the detection of *cbx2* (n = 3), *cbx4* (n = 3), *cbx6a* (n = 2), *cbx6b* (n = 2), *cbx7a* (n =3), *cbx7b* (n = 4), *cbx8a* (n = 3) and *cbx8b* (n = 3) transcripts at 1 hpf, 3 hpf, 6 hpf, 24 hpf, 48 hpf, 3 dpf, 4 dpf and 5 dpf. Ube2a is used as a control. The size of the amplicons is indicated. (**d**) RT-PCR experiment showing *cbx* transcripts in adult zebrafish tissues (n = 2). Beta-actin is used as a control. The size of the amplicons is indicated. All RT-PCR agarose gels are shown in Supplementary File S6.

The zebrafish genome encodes eight Pc orthologues. These *cbx* paralogues are *cbx2*, *cbx4*, *cbx6a*, *cbx6b*, *cbx7a*, *cbx7b*, *cbx8a* and *cbx8b*. Since zebrafish serves as a powerful vertebrate model for studying gene expression during early development and modeling human diseases, we have investigated gene expression profiles for the eight *cbx* paralogues in this organism. In zebrafish, zygotic transcription starts at about cell cycle 10–13 (around 3.5 hours post fertilization (hpf)). Before this midblastula transition (MBT) stage, all developmental events rely on maternally deposited gene products [73,74]. The study of *cbx* expression patterns before the MBT at the 1-cell stage, as well as after the MBT at 24 and 48 hours post-fertilization (hfp), using whole-mount in situ hybridization revealed that all the *cbx* family members are globally ubiquitously expressed (Figure 8A, Supplementary File S5). In situ hybridization showed that the *cbx* transcripts are maternally loaded into the embryos since a signal could be detected before MBT at the 1-cell stage, even if the labelling remains quite low for *cbx6a*, *cbx6b* and *cbx7a*. At 24 hpf, *cbx* mRNAs are ubiquitously present in the embryo. The expression becomes more restricted in the developing brain, the gut and in the pectoral fin buds at 48 hpf. However, some differences in the *cbx* expression could be observed. At 48 hpf, the expression pattern of *cbx2* is similar to the *ezh2* expression profile with a marked signal at the midbrain–hindbrain boundary and in the pectoral fin buds, whereas *cbx6b* or *cbx7b* show a more diffuse labelling in the brain and a weaker signal in the pectoral fin buds (Figure 8B).

Analyses of mRNA abundance measured by RT-PCR showed that *cbx* mRNA levels vary during zebrafish development from 1 hpf to 5 days post-fertilization (dpf) (Figure 8C). In particular, *cbx4*, *cbx6a*, *cbx6b* and *cbx7a* expression is reduced at early stages and increases after 6 hpf, whereas for *cbx7b*, *cbx8a* and *cbx8b*, a decrease in mRNA levels is found between 6 and 48 hpf. A delay between the degradation maternal mRNAs occurring at MBT and the start of zygotic expression of these genes might account for the reduction in mRNA abundancy between 6 and 48 hpf. It is worth noting that these variations in *cbx* mRNA abundance parallel those reported using large-scale RNA-Seq experiments during zebrafish development [75]. Finally, in adult zebrafish, our RT-PCR experiments showed that all *cbx* family members are expressed ubiquitously (Figure 8D).

## 4. Discussion

The Pc/CBX family member proteins are components of the canonical PRC1 protein complex that maintain transcriptional repression of hundreds of genes involved in development, differentiation, signaling or cancer. Since these proteins directly bind to the epigenetic mark H3K27me3, they are key elements targeting the PRC1 complex to its chromatin sites. While *Drosophila melanogaster* contains a single Pc protein, mammalian genomes code for five CBX paralogues, CBX2, CBX4, CBX6, CBX7 and CBX8. This diversity might be crucial for the control of gene expression programs since it is believed that changes in the CBX protein content within the PRC1 complexes could relocalize the PRC1 to different target genes during the differentiation processes. 

Here, we performed a global analysis of the *cbx* gene family in teleost fishes and traced the distribution of the *cbx* genes through teleost evolution. Teleost fish experienced at least three rounds of whole-genome duplication; the first two before the divergence of lamprey from the jawed vertebrates, and a third teleost-specific TGD at the base of the teleosts [58,76,77]. The TGD was followed by a rediploidization process associated to a massive loss of duplicated genes. However, a number of genes are still maintained as pairs of ohnologues in teleost genomes. Consequently, it is expected that the number of *cbx* family members will be higher in teleost fishes than in mammals. Database searches identified 689 *cbx* genes in 68 teleost species that belong to 21 different orders and 6 different super-orders (Osteoglossomorpha, Clupeomorpha, Ostariophysi, Protacanthopterygii, Paracanthopterygii and Acanthopterygii). The five *cbx* paralogues, *cbx2*, *cbx4*, *cbx6*, *cbx7* and *cbx8* were found in all fish teleost species. This is in total contrast with the situation observed for other components of the PRC1 protein complex [37]. Indeed, mammalian genomes contain six Psc/PCGF paralogues, *PCGF1*, *PCGF2*, *PCGF3*, *BMI1*, *PCGF5* and *PCGF6*, whereas both *pcgf2* and *pcgf3* are absent in zebrafish and *pcgf1* is absent in medaka. Similarly, there are two Sce/RING1 paralogues in mammals, *RING1* and *RNF2*, while *rnf2* is the only paralogue present in zebrafish. The maintenance of the five *cbx* paralogues in teleost could suggest the absence of functional redundancy between the Cbx family members, whereas Pcgf members could be redundant. The hypothesis of a Pcgf redundancy in teleost fishes is in agreement with the absence of certain *pcgf* gene members in the genome of several fish species including zebrafish and the medaka [37] and with the fact that *pcgf1* zebrafish mutants are viable and fertile [78]. However, the situation might be different in mammals where PCGF functions are definitively not redundant [79,80].

Surprisingly, although about 15% to 20% of the ohnologues were retained as pairs after the TGD in teleost [9], the maintenance of two ohnologues is globally a general characteristic of all *cbx* family members. *Cbx8* is present as a pair of ohnologues (*cbx8a* and *cbx8b*) in all the teleost clades examined, as it is the case for *cbx4* and probably also for *cbx7*. *Cbx7* is identified as a pair of ohnologues in all clades except in Paracanthopterygii. However, the Atlantic cod is the only specie from this super-order having its genome sequence available. It is thus difficult to conclude whether the loss of one of the two *cbx7* ohnologues is specific to the Atlantic cod or whether it reflects a feature common to all Paracanthopterygii. In contrast, *cbx2* and *cbx6* are present as pairs of ohnologues in the genome of several teleost clades but as singletons in others. Following the TGD, one *cbx6* ohnologue has been lost in the common ancestor of Euteleosteomorpha. Concerning *cbx2*, one of the two ohnologues has been lost in Osteoglossomorpha and Otomorpha, but retained as two copies in Euteleosteomorpha. This suggests that the loss of *cbx2* ohnologues occurred at least twice, in Osteoglossomorpha and in Otomorpha, during teleost evolution. The reason why *cbx4*, *cbx7*, *cbx8*, but also *cbx3* are retained as pairs of ohnologues in the teleost genomes, whereas *cbx2* and *cbx6* remain as singletons in different teleost clades is not clear. One possibility could be linked to a possible neofunctionalization and/or subfunctionalization of one of the *cbx3*, *cbx4*, *cbx7*, *cbx8* ohnologues, but it could also be due to constraints applied by the presence/absence of other genes in the blocks of synteny. 

Our analysis of the teleost *cbx* gene family also shed light on other rearrangements having occurred later in particular teleost lineages. For instance, a third copy of *cbx2*, probably arising from an intrachromosomal rearrangement, is identified in jewelled blenny (*Salarias fasciatus*, order Blenniformes). Notably, an additional *cbx4* copy also originate from a single *cbx4* duplication event having occurred in the Characiformes before their speciation since these three *cbx4* copies are found in the genomes of the two Characiformes, the Mexican cavefish (*Astyanax mexicanus*) and the red-bellied piranha (*Pygocentrus nattereri*), for which genomic sequences are available. 

In this landscape, the genomic *cbx* gene content in zebrafish, with *cbx2* and *cbx4* present as singletons but *cbx6*, *cbx7* and *cbx8* retained as pairs of ohnologues, appears as an original combination, unique among the teleost fishes having their genome sequenced.

Zebrafish has proven being a unique vertebrate model for studying Polycomb group (PcG) genes during early development [81,82,83,84]. In this context, the description of PcG gene expression during development is of particular interest. The expression of the Psc/PCGF family as well as several other PcG genes has already been reported [78,85], but very little is known about Pc/CBX gene expression during zebrafish development. Whole-mount in situ hybridization and RT-PCR experiments showed that zebrafish *cbx* genes are maternally expressed in the embryo at diverse levels. In particular, *cbx4*, *cbx6a* and *cbx7a* mRNAs appear less abundant at the 1-cell stage than at later developmental stages, while it is not the case for *cbx2*, *cbx7b*, *cbx8a* or *cbx8b*. At 24 hpf, *cbx* mRNAs are present in all the embryos but the expression becomes enriched in anterior regions such as the brain, the pectoral fin buds and the gut at 48 hpf. If all the *cbx* genes are expressed in the brain, differences in their expression patterns could be observed using in situ hybridization. Finally, all the *cbx* family members are expressed in adult zebrafish tissues.

In conclusion, our observations contribute to the understanding of Polycomb orthologues evolution in fish and the characterization of *cbx* expression during zebrafish development will be useful to future studies aiming at understanding the functional role of each *cbx* family member.

## Supplementary Materials

The following are available online at https://www.mdpi.com/2073-4425/11/4/362/s1, Table S1: The teleost Pc orthologues, Table S2: The teleost *cbx3* genes, File S1: Rooted version including gene names of the phylogenetic tree of Figure 1, File S2: List of the protein sequences encoded by the teleost Pc orthologues identified in this study, File S3: List of Cbx3 protein sequences encoded by the teleost genomes and identified in this study, File S4: Version of the Cbx3 phylogenetic tree of Figure 7 including the gene symbols, File S5: *in situ* hybridization on groups of zebrafish embryos at the 1-cell stage, 24 hpf and 48 hpf for *cbx2*, *cbx4*, *cbx6a*, *cbx6b*, *cbx7a*, *cbx7b*, *cbx8a* and *cbx8b*, File S6: Original RT-PCR gels, Figure S1: Phylogenetic tree of the evolutionary relationships between teleost Cbx6 paralogues, Figure S2: Sequence alignment for Cbx6 proteins from Ostariophysi and Clupeomorpha fishes, Figure S3: Blocks of synteny at the *cbx6* locus in teleost fishes, Figure S4: Blocks of synteny at the *cbx2* locus in teleost fishes, Figure S5: Blocks of synteny at the *cbx4-cbx8* locus in teleost fishes, Figure S6: Average quantification of RT-PCR amplicons.

## Appendix A

**Table A1:** List of the teleost genomes.

		Family	NCBI Genome	Ensembl ID	Assembly
**Aca**	Eastern happy (*Astatotilapia calliptera*)	Cichlidae	ID: 15018	ENSACAG	fAstCal1.2
**Aci**	Midas cichlid (*Amphilophus citrinellus*)	Cichlidae		ENSACIG	Midas_v5
**Ame**	Mexican cavefish (*Astyanax mexicanus*)	Characidae	ID: 13073	ENSAMEG	Astyanax_mexicanus-2.0
**Aoc**	Clown anemonefish (*Amphiprion ocellaris*)	Pomacentridae	ID: 8181	ENSAOCG	AmpOce1.0
**Ape**	Orange clownfish (*Amphiprion percula*)	Pomacentridae		ENSAPEG	Nemo_v1
**Apo**	Spiny chromis (*Acanthochromis polyacanthus*)	Pomacentridae	ID: 54316	ENSAPOG	ASM210954v1
**Ate**	Climbing perch (*Anabas testudineus*)	Anabantidae	ID: 33761	ENSATEG	fAnaTes1.1
**Bsp**	Siamese fighting fish (*Betta splendens*)	Osphronemidae	ID: 36335		fBetSpl5.2
**Cau**	Goldfish (*Carassius auratus*)	Cyprinidae	ID: 10773		ASM336829v1
**Cca**	Common carp (*Cyprinus carpio*)	Cyprinidae	ID: 10839		common carp genome
**Cgo**	Channel bull blenny (*Cottoperca gobio* )	Bovichtidae	ID: 76087		CotGob3.1
**Cha**	Atlantic herring (*Clupea harengus*)	Clupeidae	ID: 15477		Ch_v2.0.2
**Cse**	Tongue sole (*Cynoglossus semilaevis*)	Cynoglossidae	ID: 11788	ENSSCEG	Cse_v1.0
**Cva**	Sheepshead minnow (*Cyprinodon variegatus*)	Cyprinodontidae	ID: 13078	ENSCVAG	C_variegatus-1.0
**Dcl**	Denticle herring (*Denticeps clupeoides*)	Denticipitidae	ID: 7889		fDenClu1.1
**Dre**	Zebrafish (*Danio rerio*)	Cyprinidae	ID: 50	ENSDARG	GRCz11
**Eel**	Electric eel (*Electrophorus electricus*)	Gymnotidae	ID: 72955	ENSEELG	Ee_SOAP_WITH_SSPACE
**Elu**	Northern pike (*Esox lucius*)	Esocidae	ID: 22932	ENSELUG	Eluc_v4 / Eluc_V3
**Ena**	Live sharksucker (*Echeneis naucrates*)	Echeneidae	ID: 22910		fEcheNa1.1
**Fhe**	Mummichog (*Fundulus heteroclitus*)	Fundulidae	ID: 743	ENSFHEG	Fundulus_heteroclitus-3.0.2
**Gac**	Stickleback (*Gasterosteus aculeatus*)	Gasterosteidae		ENSGACG	BROAD S1
**Gaf**	Western mosquitofish (*Gambusia affinis*)	Poeciliidae		ENSGAFG	ASM309773v1
**Gmo**	Cod (*Gadus morhua*)	Gadidae		ENSGMOG	gadMor1
**Gwi**	Blunt-snouted clingfish (*Gouania willdenowi*)	Gobiesocidae	ID: 76090	ENSGWIG	fGouWil2.1
**Hbu**	Burton’s mouthbrooder (*Haplochromis burtoni*)	Cichlidae	ID: 3328	ENSHBUG	AstBur1.0
**Hco**	Tiger tail seahorse (*Hippocampus comes*)	Syngnathidae	ID: 16754	ENSHCOG	H_comes_QL1_v1
**Hhu**	Huchen (*Hucho hucho*)	Salmonidae		ENSHHUG	ASM331708v1
**Ipu**	Channel catfish (*Ictalurus punctatus*)	Ictaluridae	ID: 198	ENSIPUG	IpCoco_1.2
**Kma**	Mangrove rivulus (*Kryptolebias marmoratus*)	Rivulinae	ID: 23212	ENSKMAG	ASM164957v1
**Lbe**	Ballan wrasse (*Labrus bergylta*)	Labridae	ID: 14767	ENSLBEG	BallGen_V1
**Lca**	Barramundi perch (*Lates calcarifer*)	Centropomidae	ID: 14180	ENSLCAG	ASM164080v1 ASB_HGAPassembly_v1
**Lcr**	Large yellow croaker (*Larimichthys crocea*)	Sciaenidae	ID: 12197		L_crocea_2.0
**Mal**	Swamp eel (*Monopterus albus*)	Synbranchidae	ID: 24053	ENSMALG	M_albus_1.0
**Mar**	Zig-zag eel (*Mastacembelus armatus*)	Mastacembelidae	ID: 31700	ENSMAMG	fMasArm1.1
**Mmo**	Ocean sunfish (*Mola mola*)	Molidae		ENSMMOG	ASM169857v1
**Mze**	Zebra mbuna (*Maylandia zebra*)	Cichlidae	ID: 2640	ENSMZEG	M_zebra_UMD2a
**Nbr**	Lyretail cichlid (*Neolamprologus brichardi*)	Cichlidae	ID: 3329	ENSNBRG	NeoBri1.0
**Nco**	Black rockcod (*Notothenia coriiceps*)	Nototheniidae	ID: 10753		NC01
**Nfu**	Turquoise killifish (*Nothobranchius furzeri*)	Nothobranchiidae	ID: 2642		Nfu_20140520
**Oki**	Coho salmon (*Oncorhynchus kisutch*)	Salmonidae	ID: 13127		Okis_V1
**Ola**	Medaka (*Oryzia latipes*)	Adrianichthyidae	ID: 542	ENSORLG	ASM223467v1
**Omy**	Rainbow trout (*Oncorhynchus mykiss*)	Salmonidae	ID: 196		Omyk_1.0
**Oni**	Tilapia (*Oreochromis niloticus*)	Cichlidae	ID: 197	ENSONIG	O_niloticus_UMD_NMBU Orenil1.0
**Pfl**	Yellow perch (*Perca flavescens*)	Percidae	ID: 15707		PFLA_1.0
**Pfo**	Amazon molly (*Poecilia formosa*)	Poeciliidae	ID: 13072	ENSPFOG	Poecilia_formosa-5.1.2
**Pki**	Elephantfish (*Paramormyrops kingsleyae*)	Mormyridae	ID: 66590	ENSPKIG	PKINGS_0.1
**Pla**	Sailfin molly (*Poecilia latipinna*)	Poeciliidae	ID: 17477	ENSPLAG	P_latipinna-1.0
**Pma**	Mudskipper (*Periophthalmus magnuspinnatus*)	Oxudercidae		ENSPMG	PM.fa
**Pme**	Shortfin molly (*Poecilia mexicana*)	Poeciliidae	ID: 14658	ENSPMEG	P_mexicana-1.0
**Pna**	Red-bellied piranha (*Pygocentrus nattereri*)	Serrasalmidae	ID: 10452	ENSPNAG	Pygocentrus_nattereri-1.0.2
**Pny**	Makobe Island cichlid (*Pundamilia nyererei*)	Cichlidae	ID: 3330	ENSPNYG	PunNye1.0
**Pra**	Indian glassy fish (*Parambassis ranga*)	Ambassidae	ID: 76088		fParRan2.1
**Pre**	Guppy (*Poecilia reticulata*)	Poeciliidae	ID: 23338	ENSPREG	Guppy_female_1.0_MT
**Sal**	Arctic char (*Salvelinus alpinus*)	Salmonidae	ID: 12179		ASM291031v2
**San**	安水金**线鲃** (*Sinocyclocheilus anshuiensis*)	Cyprinidae	ID: 38362		SAMN03320099.WGS_v1.1
**Sdu**	Greater amberjack (*Seriola dumerili*)	Carangidae	ID: 12614	ENSSDUG	Sdu_1.0
**Sfa**	Jewelled blenny (*Salarias fasciatus*)	Blenniidae	ID: 7248	ENSSFAG	fSalaFa1.1
**Sfo**	Asian bonytongue (*Scleropages formosus*)	Osteoglossidae	ID: 7635	ENSSFOG	fSclFor1.1 / ASM162426v1
**Sgr**	Golden-line barbel (*Sinocyclocheilus grahami*)	Cyprinidae	ID: 9142		SAMN03320097.WGS_v1.1
**Sla**	Yellowtail amberjack (*Seriola lalandi dorsalis*)	Carangidae	ID: 12613	ENSSLDG	Sedor1
**Sma**	Turbot (*Scophthalmus maximus*)	Scophthalmidae		ENSSMAG	ASM318616v1
**Spa**	Bicolor damselfish (*Stegastes partitus*)	Pomacentridae	ID: 13077	ENSSPAG	Stegastes_partitus-1.0.2
**Srh**	犀角金**线鲃** *(Sinocyclocheilus rhinocerous)*	Cyprinidae	ID: 38394		SAMN03320098_v1.1
**Ssa**	Atlantic salmon (*Salmo salar*)	Salmonidae	ID: 369		ICSASG_v2
**Tni**	Tetraodon (*Tetraodon nigroviridis*)	Tetraodontidae		ENSTNIG	TETRAODON 8.0
**Tru**	Japanese puffer (*Takifugu rubripes*)	Tetraodontidae	ID: 63	ENSTRUG	fTakRub1.2 / FUGU5
**Xco**	Monterrey platyfish (*Xiphophorus couchianus*)	Poeciliidae	ID: 16825	ENSXCOG	X_couchianus-1.0 Xiphophorus_couchianus-4.0.1
**Xma**	Platyfish (*Xiphophorus maculatus*)	Poeciliidae	ID: 10764	ENSXMAG	X_maculatus-5.0-male
