# Supplementary material for: The Polycomb Orthologues in Teleost Fishes and Their Expression in the Zebrafish Model
Source: Genes (Basel). 2020 Mar 27;11(4):362. doi: 10.3390/genes11040362 (PMC7230241; doi:10.3390/genes11040362)
Supplement: Supplementary file 1 [file genes-11-00362-s001.zip › Supp Material/Supp Figure S2.pdf]

```
                         10        20        30        40        50        60
                          |         |         |         |         |         |
Sgr_LOC107585029   MELSAAGDRVFAAEAILKRRIRKGRMEYLVKWKGWAIKYSTWEPEENILDERLVAAFEQK
San_LOC107662367   MELSAAGDRVFAAEAILKRRIRKGRMEYLVKWKGWAIKYSTWEPEENILDERLVAAFEQK
Srh_LOC107740604   MELSAAGDRVFAAEAILKRRIRKGRMEYLVKWKGWAIKYSTWEPEENILDERLVAAFEQK
Cau_LOC113046746   MELSAAGDRVFAAEAILKRRIRKGRMEYLVKWKGWAIKYSTWEPEENILDERLVAAFEQK
Cca_LOC109095357   MELSAAGDRVFAAEAILKRRVRKGRMEYLVKWKGWAIKYSTWEPEENILDERLVAAFEQK
Cau_LOC113047918   MELSAAGDRVFAAEAILKRRVRKGRMEYLVKWKGWAIKYSTWEPEENILDERLVAAFEQK
Sgr_LOC107602318   MELSAAGDRVFAAEAIMKRRVRKGRMEYLVKWKGWAIKYSTWEPEENILDERLVAAFEQK
San_LOC107690738   MELSAAGDRVFAAEAIMKRRVRKGRMEYLVKWKGWAIKYSTWEPEENILDERLVAAFEQK
Srh_LOC107710048   MELSAAGDRVFAAEAIMKRRIRKGRMEYLVKWKGWAIKYSTWEPEENILDERLVAAFEQK
Dre_Cbx6b          MELSAAGDRVFAAEAILKRRVRKGRMEYLVKWKGWAIKYSTWEPEENILDERLVAAFEQK
Ame_cbx6b          MELSAAGDRVFAAEAILKRRVRKGRMEYLVKWKGWAIKYSTWEPEENILDDRLVVAFEQK
Pna_LOC108413245   MELSAAGDRVFAAEAILKRRVRKGRMEYLVKWKGWAIKYSTWEPEENILDERLVAAFEQK
Ipu_LOC108260787   MELSASGDRVFAAEAILKRRVRKGRMEYLVKWKGWAIKYSTWEPEENILDDRLVAAFEQK
Eel_LOC113569862   MELSVAGDRVFAAEAILKRRVRKGRIEYLVKWKGWAIKYSTWEPEENILDDRLVAAFEQK
Srh_LOC107720374   MEVSAVGEQVFPAEAILKSRVRKGRIEYLVKWKGWALKHSTWEPEENILDDRLITAFEQK
San_LOC107667890   MEVSTVGEQVFPAEAILKSRVRKGQIEYLVKWKGWALKHSTWEPEENILDDRLIMAFEQK
Cca_LOC109066300   MEVSAVGERVFPAEAILKSRVRKGRIEYLVKWKGWALKHSTWEPEENILDDRLITAFEKK
Cau_LOC113065021   MEVSAVGERVFPAEAILKSRVRKGRIEYLVKWKGWALKHSTWEPEENILDDRLLAAFEQK
Sgr_LOC107602676   MELSAVGERVFAAEAILKSRVRKGRIEYLLKWKGWALKHSTWEPEGNILDDRLITAFEQK
San_LOC107704552   MELSAVGERVFAAEAILKSRVRKGRIEYLLKWKGWALKHSTWEPEGNILDDRLITAFEQK
Srh_LOC107717667   MELSAVGERVFAAEAILKSRVRKGRIEYLLKWKGWALKHSTWEPEGNILDDRLITAFEKK
Cca_LOC109066296   MELSAEGERVFAAEAILKSRVRKGRIEYLVKWKGWALKHSTWEPEENILDDRLITAFEQK
Cau_LOC113039859   MELSAEGERVFAAEYILKSRVRKGRIEYLVKWKGWALKHSTWEPEENILDDRLITAFEQK
Dre_Cbx6a          MELSAAGDRVFAAEAILKSRVRKGHIEYLVKWKGWALKHSTWEPEENILDDRLIKAFEQK
Ame_LOC103038090   MELSGMGERVFAAEAILKRRVRKSNIEYLVKWKGWALKHSTWEPEENILDDRLISAFEKK
Pna_Cbx6           MELSAMGDRVFAAEAILKRRVRKGSIEYLVKWKGWALKHSTWEPEENILDGRLITAFEQK
Eel_LOC113585732   MELSAIGDRVFAAEAILKRRVRKGRIEYLVKWKGWALKHSTWEPEENILDDRLITAFERK
Ipu_LOC108259424   MELTAIGDRVFAAEAILKRRVRKGNIEYLVKWKGWALKHSTWEPEENILDDRLIAVFEQK
Dcl_LOC114786320   MELSAVGDRVFAAEAILKRRVRKGRIEYLVKWKGWALKYSTWEPEENILDDRLITGFERK
Cha_LOC105903838   MELSAVGDRVFAAEAILKRRVRKGQIEYLVKWKGWALKNSTWEPEENILDDRLVTAFEQK
                   **::  *::**.** *:* *:**. :***:******:* ****** **** **:  **:*

Prim.cons.         MELSAAGDRVFAAEAILKRRVRKGRIEYLVKWKGWALKYSTWEPEENILDDRLVAAFEQK


                         70        80        90       100       110       120
                          |         |         |         |         |         |
Sgr_LOC107585029   EREQEMYGP-------KKRGPKPKTLLLKSRAQVAETSSRVP----------EFKHTRPQ
San_LOC107662367   EREQEMYGP-------KKRGPKPKTLLLKSRAQVAETSSRVP----------EFKHTRPQ
Srh_LOC107740604   EREQEMYGP-------KKRGPKPKTLLLKSRAQVAETSSRVP----------EFKHTRPQ
Cau_LOC113046746   EREQELYGP-------KKRGPKPKTLLLKSRAQVADTSSRVP----------EFKHTRPQ
Cca_LOC109095357   EREQEMYGP-------KKRGPKPKTLLLKSRAQVAETSSRVP----------EFKHTRPQ
Cau_LOC113047918   EREQEMYGP-------KKRGPKPKLLLLKSRAQVAETSSRVP----------EFKHTHPK
Sgr_LOC107602318   EREQEMYGP-------KKRGPKPKTLLLKSRAQVTETSSRVP----------QFKHTRPQ
San_LOC107690738   EREQEMYGP-------KKRGPKPKKLLLKSRAQVAETSSRVP----------EFKHTRPQ
Srh_LOC107710048   EREQEMYGP-------KKRGPKPKTLLLKSRAQVAETSSRVP----------ELKHTRPQ
Dre_Cbx6b          EREQEMYGP-------KKRGPKPKTLLLKSRAQAE--SPRVP----------EFKHSRPQ
Ame_cbx6b          EREQELYGP-------KKRGPKPKTLLLKSRAQTSDTPHRVP----------EFKHTRPQ
Pna_LOC108413245   EREQELYGP-------KKRGPKPKTLLLKSRAQASDGTPRVP----------EFKHTRPQ
Ipu_LOC108260787   EREQELYGP-------KKRGPKPKTLLLKSRAQAAESSPRVL----------EFKPSRPQ
Eel_LOC113569862   EREQELYGP-------KKRGPKPKTLLLKSRAHAAEGSPRVP----------EFKHSRPQ
Srh_LOC107720374   EREQELYGP-------KKRGPKPKNFVLKVRAQAGD-RPRSS----------NTRRTPPR
San_LOC107667890   XRQQELYGP-------KKRGPKPKNFVLKARAQAGD-RPRSS----------NTRRTPPR
Cca_LOC109066300   EREQELYGP-------KKRGPKPKNFVLKARAQTGD-RPRSS----------DTQRTPPQ
Cau_LOC113065021   ERQQELYGP-------KKRGPKPKNFVLKARAHAGD-RPRSSDTQRTPPQISNTQRTPPR
Sgr_LOC107602676   ERQQELYGP-------KKRGPKPKNFVLKARAQAGD-RPRSS----------NTQRTPPR
San_LOC107704552   ERQQELYGP-------KKRGPKPKNFVLKARAQAGD-RPRSS----------NTQRTPPR
Srh_LOC107717667   EREQELYGP-------KKRGPKPKNFVLKARAQAGD-KPRSS----------NTQRTPPR
Cca_LOC109066296   EREQELYGP-------KKRGPKPKNFVLKARAQAGD-RPRSS----------NTQRTPPR
Cau_LOC113039859   EREQELYGP-------KKRGPKPKNFVLKARAQAGD-RPRSS----------NTQRTPPR
Dre_Cbx6a          EREQELYGPEQELYGPKKRGPKPKNFVLKARAQSGD-RPRSS----------YTRRTPSC
Ame_LOC103038090   EREQELYGP-------KKRGPKPKNLLLKARTQP---SPRVS----------KTRNTPPR
Pna_Cbx6           EREQELYGP-------KKRGPKPKNFLLKARAQTGETSSRGS----------SARHTPPH
Eel_LOC113585732   EREQELYGP-------KKRGPKPKTFVLKARAQAGEPSSRGS----------TTRQAPPR
Ipu_LOC108259424   ERDQELYGP-------KKRGPKPKNFVLKARAQAGETLSRGS----------STRRAPPC
Dcl_LOC114786320   EREQELYGP-------KKRGPKPKNFILKARAQTGDTSNRFS----------VARQTHNR
Cha_LOC105903838   ERQQELYGP-------KKRGPKPKNFVLKARAHAGETSSRAT----------DPRLISRR
                    *:**:***       ******** ::** *::      *              :     

Prim.cons.         EREQELYGPEQELYGPKKRGPKPKN2LLKARAQAG2TSPRVSDTQRTPPQISE2KHTPPQ
```

```
                              130       140       150       160       170       180
                                |         |         |         |         |         |
Sgr_LOC107585029    QHSKLPPPS---------AAP-----------SYTPTAPSNAKLQSGTAQPKLKKDIHRC
San_LOC107662367    QHSKLPPPS---------AAP-----------SYTPTAPSNAKLQSGTAQPKLKKDIHRC
Srh_LOC107740604    QHSKLPPPS---------AAP-----------SYTPTAPSNAKLQSGTAQPKLKKDIHRC
Cau_LOC113046746    QHSKLPPPS---------ATP-----------SYTPTVPSNAKLQSGTAQPKLKKDIHRC
Cca_LOC109095357    QHSKLPPPS---------AAP-----------SYTPTAPSNAKLQSGTAQPKLKKDIHRC
Cau_LOC113047918    QHSKFPPPS---------AAP-----------SYTPTAPSNAKLQSGTAQPKLKKDIHRC
Sgr_LOC107602318    QHSKLPPPS---------AAP-----------SYTPTTPSNAKLQSGAAQPKLKKDIHRC
San_LOC107690738    QHSKLPPPS---------AAP-----------SYTPAAPSNAKLQSGAAQPKLKKDIHRC
Srh_LOC107710048    QHSKLPPPS---------AAP-----------SYTPTAPSNAKLQSGAAQPKLKKDIHRC
Dre_Cbx6b           PHSKPPPPPP-------PAAP-----------SYTPTAPSNAKLQSGTAQPKLKKDIHRC
Ame_cbx6b           PSSKPAPPPL-------PAP------------TYTPTCPSNAKLQSGAAQPKLKKDIHRC
Pna_LOC108413245    PSSKPPPPPL-------PTP------------SYPPSCPSNAKLQSGAAQPKLKKDIHRC
Ipu_LOC108260787    PSSKPPPPPP-------PAS------------SYHPSGPSNAKLQSGAAQPKLKKDIHRC
Eel_LOC113569862    PSSKPPPQPP-------PLSPP----------SYPPSGPSNAKLQSGAAQPKLKKDIHRC
Srh_LOC107720374    TTAKPPAS-------SSSAS-----------SSSSFSTAPTPRVHSLAAAHKLKKDIHRC
San_LOC107667890    TTAKPPAS-------SSSAS-----------SSSSFSTAPTPRVHSLAAAHKLKKDIHRC
Cca_LOC109066300    IPAKLPAS-------SSSASSA--PSQ--PSSSSSLSTTPTPRVHSLAAAHKLKKDIHRC
Cau_LOC113065021    ITAKLPSS-------SSSASSA--PPQ--PSSSSSFSTAPTPRVHSLAAAHKLKKDIHRC
Sgr_LOC107602676    TTAKPPAS-------SSSASAA--PPP--PSSSSSYNTAPTPRVHSLAAAHKLKKDIHRC
San_LOC107704552    TTAKPPAS-------SSSASAA--PPP--PSSSSSYNTAPTPRVHSLAAAHKLKKDIHRC
Srh_LOC107717667    TTAKPPAS-------SSSASAA--PPQ--PSSSSSYNTAPTPRVHSLAAAHKLKKDIHRC
Cca_LOC109066296    TTAKPPAS-------SSAASAA--PPQ--PSSSSSYNTAPTPRVHSLAAAHKLKKDIHRC
Cau_LOC113039859    TTAKPPAS-------STSASAA--PPQ--PSLSSSYNTAPTPRVHSLAAAHKLKKDIHRC
Dre_Cbx6a           TTAKPPTA-------SSSASSA--TPQ--PSSSSSHSTAPTPRVHSLAAAHKLKKDIHRC
Ame_LOC103038090    SSSSSASS-------SSSAVVPPVNPVRSSSSSSSSSLAPTPRLHSLAASHKLKKDIHRC
Pna_Cbx6            SSS-------------SAAVTP--NPP---SSSSSASLAPTPRLHSLAASHKLKKDIHRC
Eel_LOC113585732    SSS--------------SSVAP-ANPP---SSSSSSSLAPTPRLHSLAASHKLKKDIHRC
Ipu_LOC108259424    TSS-------------SSPVPP--NPP------SSSSLAPTPRLHSLAASHKLKKDIHRC
Dcl_LOC114786320    SAATRPSS--------SSASSVLAPHT--STSSASSSLAPTAKLNSLAATHKLKKDIRRC
Cha_LOC105903838    SSSRPPSSRPPTSRPPSSTSTALVSAP--ASSSSSSSPAPSPKLNSLAATHKLKKDIRRC
                      :                                   ....:::* :*  ******:**
Prim.cons.          2HSKPPPPSPPTSRPSSSASPAL2PP2RSPSSSSSPSTAPT2KL2SLAAQHKLKKDIHRC

                              190       200       210       220       230       240
                                |         |         |         |         |         |
Sgr_LOC107585029    HRMARRPLPRPD---QTVGPSSPFSSRPTVSPFSETVRILNRKVKPREVKKGRVILNLK-
San_LOC107662367    HRMARRPLPRPD---QTVGPSSPFSSRPTVSPFSETVRILNRKVKPREVKKGRVILNLK-
Srh_LOC107740604    HRMARRPLPRPD---QTVGPSSPFSSRPTVSPFSETVRILNRKVKPREVKKGRVILNLK-
Cau_LOC113046746    HRMARRPLPRPD---QTIGPSSPFSSRPTVSPFSETVRILNRKVKPREVKKGRVILNLK-
Cca_LOC109095357    HRMARRPLPRPD---QTVGPSGPFSSRPTVSPFSETVRILNRKVKPREVKKGRVILNLK-
Cau_LOC113047918    HRMARRPLPRPD---QTVSPSGPFSSRPTVSPFSETVRILNRKVKPREVKKGRVILNLK-
Sgr_LOC107602318    HRMARRPLPRPD---QTVSPSGPFSSRPTVSPFSETVRILNRKVKPREVKKGRVILNLK-
San_LOC107690738    HRMARRPLPRPD---QTVSPSGPFSSRPTVSPFSETVRILNRKVKPREVKKGRVILNLK-
Srh_LOC107710048    HRMARRPLPRPD---QTVSPSGPFSSRPTVSPFSETVRILNRKVKPREVKKGRVILNLK-
Dre_Cbx6b           HRMARRPLPRQD---HTVGPSGPFSSRPTVSAFSETVRILNRKVKPREVKKGRVILNLK-
Ame_cbx6b           HRMSRRPLPRSDPLSQSVGHS--------LSPFSETVRILNRKVKPREVKKGRVILNLK-
Pna_LOC108413245    HRMSRRPLPRPDPLAQSVGHS--------LSPFSETVRILNRKVKPREVKKGRVILNLK-
Ipu_LOC108260787    HRMARRPLPRPDPLAPPIGSSGPFSSRPTVSPFCETVRILNRKVKPREVKKGRVILNLK-
Eel_LOC113569862    HRISRRPLPRLDPLAQQVGSSGPFSSRPTVSPFSETVRILNRKVKPREVKKGRVILNLK-
Srh_LOC107720374    HRMSRRPLPRPDPLGDPTGS--TSSSRPPISPFSETVRILNRKVKPREVKRGRIILNLN-
San_LOC107667890    HRMSRRPLPRPDPLGDPTGS--TSSSRPPISPFSETVRILNRRVKPREVKRGRIILNLN-
Cca_LOC109066300    HRMSRRPLPRPDPLGEPTGS--TSSSRPPISPFSETVRILNRRVKPREVKRGRIILNLN-
Cau_LOC113065021    HRMSRRPLPRPDPLGEPTGS--TTSSRPPISPFSETVRILNRRVKTREVKRGRIILNLN-
Sgr_LOC107602676    HRMSRRPLPRPDPLGDPMGT--TSLSRPPISPFSETVRILNRRVKPREVKRGRIVLNLK-
San_LOC107704552    HRMSRRPLPRPDPLGDPTGT--TSSSRPPISPFSETVRILNRRVKPREVKRGRIILNLK-
Srh_LOC107717667    HRMSRRPLPRPDPLGDPTGT--TSSSRPPISPFSETVRILNRRVKPREVKRGRIILNLK-
Cca_LOC109066296    HRMSRRPLPRPDPLGDPTGS--TSSSRPPISPFSETVRILNRRVKPREVKRGRIIVNLK-
Cau_LOC113039859    HRMSRRPLPRPDPLGNPRGS--TSSSRPPISPFSETVRILNRRVKPREVKRGRIILNLK-
Dre_Cbx6a           HMMSRRPLPRSDPLANSTG----SSSRHPISPFSETVRILNRRVKPREVKRGRIILNLK-
Ame_LOC103038090    HRMSRRPLPRY----DPDGS--SFSFRPPISPFSETVRILNRRVKPREVKRGRIILNLK-
Pna_Cbx6            HRMSRRPLPRP----DPDGS--SFSSRLPISPFSETVRILNRRVKPREVKRGRIILNLK-
Eel_LOC113585732    HRMSRRPLPRPDLLANPDGS--SFASRPPISPFSETVRILNRRVKPREVKRGRIILNLK-
Ipu_LOC108259424    HRMSRRPLPRPDPLANPDGS--SFVSRLPISPFSETVRILNRRVKPREVKRGRIILNLK-
Dcl_LOC114786320    HRMSRRPLPRPDPVGPSNNT--SFSSRPPVSPFSETVRILNRRVKPREVKRGRIILNLK-
Cha_LOC105903838    HRMSRRPLPRPDPLAPSSGSG-SFPSRPPVSPFSETVRILNRRVKPREVKRGRIILNLKG
                    * ::******        .          :*.*.********:**.****:**:::**:
Prim.cons.          HRMSRRPLPRPDPLGQPVGSSGPFSSRPP2SPFSETVRILNR2VKPREVKRGRIILNLKG
```

```
                            250       260       270       280       290       300
                              |         |         |         |         |         |
Sgr_LOC107585029   -VVDKAGNGNGVANSRRTHVPSTQQSHFGRQKIPSRNRVIGKNRRFGEVSYRGIQPTIRS
San_LOC107662367   -VVDKAGNG-GVANSRRTHMPSTQQSHFGRQKIPSRNRVIGKNRRFGEVSYRGIQPTIRS
Srh_LOC107740604   -VVDKAGNG-GVANSRRTHVPSTQQSHFGRQKIPSRNRVIGKNRRFGEVSYRGIQPTIRS
Cau_LOC113046746   -VVDKAGNG-AVANSRRTHVPSTQQSHFGRLKIPSRNRVIGKNRRFGEVSYRGIQPTIKS
Cca_LOC109095357   -VVDKAGNG-TVANSRRTHVPSAQQSHFGRQKIPSRNRVIGKNRRFGELSYRGIQTPIRS
Cau_LOC113047918   -VVDKAGHG-GVPNSRRTHVPSAQQSHFGRQKIPSRNRVIGKNRRFGEFSYRGIPTPNRN
Sgr_LOC107602318   -VVDKAGNG-GVANSRRTHVPSAQQSHFGRQKIPSQNRVIGKNRRFGELSYRGIQTPIRS
San_LOC107690738   -VVDKAGNG-GVANSRRTHVPSAQQSHFGRQKIPSRNRVIGKNRRFGELSYRGIQTPIRS
Srh_LOC107710048   -VVDKAGNG-GVANSRRTHVPSAQQSHFGRQKIPSRNRVIGKNRRFGELSYRGIQTPIRS
Dre_Cbx6b          -VLDKAENT-AVANNKRTHVPSTQQSHFGRQKIPSRNRVIGKNRRFGEVSYRGIQPPIRG
Ame_cbx6b          -MIDKAGSG-GSANNKRTQS-TTHQSHTARQKVPSRNRVIGKSRRFGEVSYRGLQLPVSG
Pna_LOC108413245   -VIDKAGNS-GVANNRRTPS-TAHQSYIGRQKVPSRNRVIGKSRRFGDVSYRGLPLPVSG
Ipu_LOC108260787   -VMDKTG----PANNKRTQN-SSHQSHLGRQKVPSRNRVIGKSRRFGDVSFRCLQQPMGG
Eel_LOC113569862   -VIDKPGGG-GVANNRRTPS-ATHQPHIGRQKVPSRNRVIGKSRRFGDVSYRGLHLPATA
Srh_LOC107720374   -VIDKSEN--SGVTSRRSPQ-----SFAGRTKIPSRNRIIGK--KQGDMPYRPFQHPMKM
San_LOC107667890   -VIDKSEN--SGVTSRRSPQ-----SFAGRAKIPSRNRIIGK--KQGDMPYRPFQHPMKM
Cca_LOC109066300   -VIDKSEN--SGVTSRRSPQ-----SFAGRAKIPSRNRIIGK--KQGDMPYRPFQHPMKM
Cau_LOC113065021   -VIDKSEN--SGVTSRRSPQ-----SFGARAKIPSRNRIIGK--KQGDMPYRPFQHPMKM
Sgr_LOC107602676   -VIDKSQ-------------------------IPSRNRIIGK--KHGDMPYRPFQHPMKM
San_LOC107704552   -VIDKSQ-------------------------IPSRNRIIGK--KHGDMPYRPFQHPMKM
Srh_LOC107717667   -VIDKSQ-------------------------IPSRNRIIGK--KHGDMPYRPFQHPMKM
Cca_LOC109066296   -VIDKSEN--SGVASRKTPQ-----SFMGRAKIPSRNRIIGK--KHGDMPYRPFQHPMKM
Cau_LOC113039859   -VIDKSEN--SGVASRRTPQ-----SFTGRAKIPSRNRIIGK--KHGDMPYRPFQHPMKM
Dre_Cbx6a          -VIDKSEN--GGMTSRRTPQ-----SFAGRAKIPSRNRIIGK--KHGDMPYRPFQHPMKM
Ame_LOC103038090   -VIDKCG---GRAANRGPTQ-----SHQGRAQIPSRNRIIGK--KQGDMPYRPFQPPMKM
Pna_Cbx6           -VIDKYG---GGATNRRPAQ-----TQQGRAQLPSRNRIIGK--KQGDMPYRPFQPPMKM
Eel_LOC113585732   -VIDKYGSGGGGATHRRPAQ-----SHQGRAQIPSRNRIIGK--RQGELPYRPFQPPMKM
Ipu_LOC108259424   -VIDKYG--------AGTTK-----GHQGRAHIPSRNRIIGK--RQGEMPYKPFQPPMKM
Dcl_LOC114786320   -VIDKPGSGAG-TSSRKVPT-----SHLGRATIPSRNRIIGR--KCGNMPYRPFQPPVKM
Cha_LOC105903838   SCMDKAGGGG--------PA-----AR--RAAIPSRNRIIGK--RFGDVPYRPFQPPMKM
                     :**                           :**:**:**:  : *:..:: :  .
Prim.cons.         SVIDKAGNGGGVANSRRTPQPSTQQSHFGRQKIPSRNRIIGKNRRFGDMPYRPFQPPMKM


                            310       320       330       340       350       360
                              |         |         |         |         |         |
Sgr_LOC107585029   SGFPVFGKLFDSHSLNNAENQ---TQ---SGESCNNTTNNLSSSQ--SSKV-DVSKVQTL
San_LOC107662367   SGFPVFGKLYDSHSLSNAENQ---TQ---SGESRNNTTNNLSSSQ--NSKV-DVSKGQTL
Srh_LOC107740604   SGFPVFGNLFDSHSLSNAENQ---TQ---SGESRNNTTNNLSSSQ--SSKM-DVSKGQTL
Cau_LOC113046746   SGFPVFGKLFDSHSLSNAENQ---TQ---SGESRNNTTNNLASSQ--SSKA-DVSKGQTL
Cca_LOC109095357   SGFPVFGKLFDSHSLNNAENQ---TQ---SGESRNNMTNTLSSSQ--CSKL-DASKGQTL
Cau_LOC113047918   SGFPVFGKLFDSHSLNNAENQ---TQ---SGESRDNTAKNLSSS-----------KNQTL
Sgr_LOC107602318   SGFPVFGKLFESHSLNNAENQ---TQ---SGESRNNTTNNLSSSQ--SSKV-DASKGQTL
San_LOC107690738   SGFSVFGKLFDSHSLNNAENQ---TQ---SGESRNNTTNDLSSSQ--SSKV-DASKGQTL
Srh_LOC107710048   SGFPVFGKLFDSHSLNNAENQ---TQ---SGESRNNTTNNLSSSQ--SSKV-DASKGQTL
Dre_Cbx6b          SGFPVFGKLFDSHSLKNAE----------SGESRNSMTNNLSSSQ--SSKLLDVSKGQNR
Ame_cbx6b          SGFSMFGKTFNSHPMDNSGNQPAVEHKESGQKSSSKGLSSQSSSQ--SSKVATSDVPSAL
Pna_LOC108413245   AGFSVFGKAFEAHSMDPSGNQPKLEH--SGHKPG-KSLSLQSLSQ--SSKLMTSDVPKAL
Ipu_LOC108260787   AGFSVFRKPIEAYPMDPSEEKPKAEQ--SGHKPGTNPS--PSSSQ--SSKVKNSAVPEAL
Eel_LOC113569862   AGFPVFGKPFECHPMEVSEEKPKVEQ--CVPKVACNSSSLPSSSQ--SPKVMTPDIPKAL
Srh_LOC107720374   LGFPMYGQPFGLHPCGSMSSM--------ANEESNSGANRRGGNRGKSCDSHSSANAQKF
San_LOC107667890   LGFPMYGQPFGLHPCGSMSSV--------ANEESNTGANQRGGN---CCDSHSSANAQKF
Cca_LOC109066300   LGFPMYGQPFGLHPCGSVSSM--------ANEESNTGANRRGGN---CCDSHSSANVQKF
Cau_LOC113065021   LGFPMYGQPFGLHPCGSVSSM--------ANEESKTGANQRGGN---CCDSHSSASTQKF
Sgr_LOC107602676   LGFPMYGQPFGLNPCGSMSSM--------ANEGSNTGVNRRGGG---CCDSHSSANAQKF
San_LOC107704552   LGFPMYGQPFGLNPCGSMSSM--------ANEGSNTGVNRRGGG---CCDSHSSANARKF
Srh_LOC107717667   LGLPMYGQPFGLNPCGSMSSM--------ANEGSNTGVNRRGGG---CCDSHSSANAQKF
Cca_LOC109066296   LGFPMYGQPFGLNPCGSMSSM--------ANDGSNTVVNQRGGG---CCDSHSSANAQKF
Cau_LOC113039859   LGFPMYGQPFGLNPCGSMSSM--------ANEGSNTGVNQRGGG---CCDSHSSASTQKF
Dre_Cbx6a          LGIPMYGQPFGLNPSGGASSM--------ANEGSNTRASHGGGS---RCGSQSSA--QRF
Ame_LOC103038090   LGFPMYGQPFGLQPFPPVSAQ--------PNRRAGSTSGTRGRGG--GCGPGRPRKATKF
Pna_Cbx6           LGFPMYGQPFGLQPCLPVSVQ--------TSKRAGSTPVAKGRG------PGRPSKP-KF
Eel_LOC113585732   PGFPMYGQPFGLQPCPMVSVK--------TSRSAGPTTGSKGAGR--NCGPGHSSAE---
Ipu_LOC108259424   PGFPMYGQPFGLQPCPPASAS--------AGTRAGSAIGSKTRAH--S--SADSSRV---
Dcl_LOC114786320   PGFPMYGKPFGLQQMNPVPFQ--------THIRFGCNTNARGAHSTSASATTGAPQALQ-
Cha_LOC105903838   TGFPMYGKPYGMQRIGSDSFQ--------ANAGMASNTEAS-IGGTSAPASASTPHAVP-
                    *:.:: :
Prim.cons.         LGFPMYGKPFGLHPCGNASNQPKVTQKESANESSNNTTNNRGSSQTSSSKSHSSSKAQKL
```

```
                             370       380       390       400       410       420
                               |         |         |         |         |         |
Sgr_LOC107585029     DELHP-SNSSSEVSDGEPPSPPQT--QSQHSSLPPKAS-----ATKAQDPALHK------
San_LOC107662367     DELPP-SNSSSEVSDGEPPSPPQT--QSQHSSLPPKAS-----ATKAQDPALHK------
Srh_LOC107740604     DELPP-SNSSSEVSDGEPPSPPQT--QSQHSSLPPKAS-----ATKAQDPALHK------
Cau_LOC113046746     DELPP-SNSSSEVSDGEPPSPPQT--QSQHSSLPPKAS-----ATKAQDPALHK------
Cca_LOC109095357     DELPP-SNSSSEVSDGEPHSPPQT--QSQHSSLQPKSS-----ATKAPDPTLHK------
Cau_LOC113047918     DELPP-SNSSSEVSDGEPNSPPQT--QSQRSSLQPKSS-----TIKAPDPTLHK------
Sgr_LOC107602318     DELPQ-SNSSSEVSDGEPPSPPQT--QSQHSSLQPKSS-----ATKAPDPTLHK------
San_LOC107690738     DELPP-SNSSSEVSDGEPPSPPQT--QSQHSSLQPKSS-----ATKAPDPTLHK------
Srh_LOC107710048     DELPP-SNSSSEVSDGEPPSPPQT--QSQHSSLQPKSS-----ATKAPDPTLHK------
Dre_Cbx6b            NEEPPPSNSSSEVSDGEPPSPRQT--QSQHSSLQPKASSTKSPSTKAPDPTLHK------
Ame_cbx6b            NEPPP-SASSSEVSDGESHPTSVAPTKSHQPLSAHDHK-----SSENNCFKLPSLSLQHN
Pna_LOC108413245     SEAPP-SASSSEVSDGESHLTSSA--QSHQPSLAHQ-------SSKVNPAILPP--L---
Ipu_LOC108260787     VEPPR-SASSSEVSDSEPHPPLPP--QFHPN-QLPQ-------SGEANSQISHA------
Eel_LOC113569862     SEPPC-SPSSPEVSEGELLFPPPT--HPQPPPSAAQ-------PCQANLAILPP------
Srh_LOC107720374     QYQPPPSPSSFSESNNSSPS-LQKQPTQPEAPT---------SPTKLDSAASSRS-----
San_LOC107667890     QYQPPPSPSSFSGSNNSSPS-LQKQPTQPEAPT---------SPTKLDSAASSRS-----
Cca_LOC109066300     QYQTPPSPSSSSGSNNSSPS-LQKQPTQPEAPT---------SPTKLDSAGSSRS-----
Cau_LOC113065021     QYQHPPSPSSSSGSNNSSLS-LQKTQTPLEAPM---------SPTKLDSSASSRS-----
Sgr_LOC107602676     QYQPPPSPSSSSGSNNSSPS-PQKQVTQPEAP------------TKLDSAASSRS-----
San_LOC107704552     QYQPPPSPSSSSGSNNSSPS-PQEQVTQPEAP------------AKLDSAASSRS-----
Srh_LOC107717667     QYQPPPSPSSSSGSNNSSPS-PQKQVTQPEAP------------TKLDSAASSRS-----
Cca_LOC109066296     QYQPPPSPTSSSGSINITPS-LQKQATQPEAP------------TKLDSAASSRS-----
Cau_LOC113039859     QYQPPPSPSSSSGSNNSPPS-PQKQATQPEAP------------TKLDSAASTCS-----
Dre_Cbx6a            QYQPTPSPSNSSGSNGSSPS-TQKQPTQKEGPT---------SSAKLTPTESSRS-----
Ame_LOC103038090     PYQSPPSPSSSSGSESDASSPPKIQPGPPQASPSETAPSS--PPV-LSPETPSRGSEP--
Pna_Cbx6             PFQSPPSPSSSSGSESDAPSPPQIQPGPPQAPP-ETAPSS--PPV-LSPETPSRCSGV--
Eel_LOC113585732     QFQSPPSPSSSSGSESDAPSLPQIQATPPKAPP-DSAPSS--PPV-LSPETPSRATDD--
Ipu_LOC108259424     RIQSPPSPSSSSGTESDAPSSRQIHPRRKKAQLAAAATSS--PPLALSTETSSQA-----
Dcl_LOC114786320     HYQPPPSPSSSGSSESQPPSPPSIQVSAPSAVA---GAPT--STAKLRSQTQPQ-PGD--
Cha_LOC105903838     QYMSPPSPSSSSGSDSRPPTPRQSKLAAPHPPP---RTRA--SPPKLRAPAAPQGSGT--
                           * :.   : .
Prim.cons.           QEQPPPSPSSSS2SDGEPPSPPQTQPQSP2APLQPKASSSKSSPTKLDSPALSRSSGDHN


                             430       440       450       460       470       480
                               |         |         |         |         |         |
Sgr_LOC107585029     -----LSAQPVASKN-----SSGPSAVPSSPMFSSSSSA---SSSSEDNEHILD--LSVP
San_LOC107662367     -----LSAQPVASKN-----SSGPSALPSSPMFSSSSSA---SSSSEDNEHILD--LSVP
Srh_LOC107740604     -----LSAQPVASKN-----SSGPSALPSSPMFSSSSSA---SSSSEDNEHILD--LSVP
Cau_LOC113046746     -----LSAQPAASKN-----SSGPSALPSSPMFSSSSSA---SSSSEDNEHILD--LSVP
Cca_LOC109095357     -----LSGQTVPSKN-----SLAPSALPSSPMFSSSSSA---SSSSEDNERILD--LSVP
Cau_LOC113047918     -----HSAQTVPSEN-----SLVPTSLPSSPMFSSSSSA---SSSSEDNERILD--LSVP
Sgr_LOC107602318     -----LSAQPVPSKN-----SLGLSALPSSPMFSSSSSA---SSSSEDSERILD--LSVP
San_LOC107690738     -----LSAQPVPSKN-----SLGLSALPSSPMFSSSSSA---SSSSEDSERILD--LSVP
Srh_LOC107710048     -----LSAQPVPSKN-----SLGLSALPSSPMFSSSSSA---SSSSEDSERILD--LSVP
Dre_Cbx6b            -----LGTQPVASKNK----DSASSALPSSPMFSSSSSA---STSSEDNEHILD--LSVP
Ame_cbx6b            LKDQKQSNQLKDSQPKKSNPSAAQSLLPSSPMFSSSSSS---SSSSEDNEHILD--LSVP
Pna_LOC108413245     ---K-PSNQLTDLKP---NSSAAKSVLPSSPMFSSSSSS---SSSSEDNEHILD--LSVP
Ipu_LOC108260787     ---AKSSPQLTDSKA---KLT--QSAMPSSPMFSSSSSSSSLSSSSEENEHILD--LSVP
Eel_LOC113569862     ---AKPNTQLTDCKP---NSTPAQSTLPSSPMFSSSSS----SSSSEDSEHILD--LSVP
Srh_LOC107720374     ---RDASQPPPKSSS--------APFLLSSPSYSSSL-----SSSLEDEDQGSPN-LATS
San_LOC107667890     ---RDASQPPPKSSS--------APFLPSSPSYSSSL-----SSSLEDEDQGSPN-LATS
Cca_LOC109066300     ---QDASQPHPKSSS--------APFLPSSPSYLSSP-----SFSLDDEDQSSPN-LTTS
Cau_LOC113065021     ---QDSSQPHPKSSS--------APFLPSSHSYSSSP-----SFSLEDEDQGSQN-LTTS
Sgr_LOC107602676     ---RDASQPHPKSSS--------VPFLPSSPSYSSS--------SLEGENQGSPN-LVTS
San_LOC107704552     ---QDASQPHPKSSS--------APFLPSSPSYSSS--------SLEGENQGSPN-LVTS
Srh_LOC107717667     ---RDASQPHPKSSS--------APFLPSSPSYSSS--------SLEGENQGSPN-LVTS
Cca_LOC109066296     ---RDASQPHPKSSS--------APFLPSSPSYSSSP-----SSSLEDEDQGSPN-LTTS
Cau_LOC113039859     ---QDASQPHPKSSS--------APFLPSSPSYSTSP-----SSSIEDEDQGSPN-LAIS
Dre_Cbx6a            ---HDTSQPHPKSSS--------TPFLPSSPSYSSSP-----SSSQEDEDLGSLK-SATS
Ame_LOC103038090     -PQNHSSSAKPGSASN------PTSFLPSSPSLSSSA-S--SSSSPEDEEEKAQ------
Pna_Cbx6             -PQSRSSGAKPGSASS------PTSFLPSSPSLSS---S--SSSSPEDEDERAQK-LPAP
Eel_LOC113585732     -PQNCPSSAQPGSVSD------PVPFLPSSPSLSSSS-P--SSSSPEDDDERARN-VPPA
Ipu_LOC108259424     --HNRSTCTKSGSS---------APRLPSSPPHSSSSSS--LSSSPEDKKGRAQN-LPFV
Dcl_LOC114786320     -PQFQPGSTQASTAP--------VPFLPASPSLSSSQ----SSSSFEDDEDEGRGGLSTQ
Cha_LOC105903838     -SQHQPTAAQSAAVPE------SAAFLPCSPSLSSS------SSSLEDEEGEDAANLSAS
                                             . : .*    :         * : ..
Prim.cons.           LPQ2DLSQQPPKSKSKKSNSS2GASFLPSSPSFSSSSSASSSSSSSEDEE2ILDN2LSVP
```

```
                               490       500       510       520       530       540
                                 |         |         |         |         |         |
Sgr_LOC107585029      -HEMDRRLRRRHPFSG----------------RHPLKVPEVPVSEE----PSEEEEDFDW
San_LOC107662367      -HEMDRRLRRRHPFSG----------------RHPLKVPEVPVSEE----PSEEEEDLDW
Srh_LOC107740604      -HEMDRRLRRRHPFSG----------------RHPLKVPEVPVSEE----PSEEEEDLDW
Cau_LOC113046746      -HEMDRRLRRRHPFSG----------------RHPLKVPEVPVSEE----PSEEEEDLDW
Cca_LOC109095357      -HEMDRRLRHRHPFSG----------------RHPLKVPEVPVSEE----PSEEEEDLDW
Cau_LOC113047918      -HEMDKRLRRRHPFSG----------------RHPLKVPEVPVSEE----PSEEEEDLDW
Sgr_LOC107602318      -HEMDRRLRRRHPFSG----------------RHPLKVPEVPVSEE----PSEEEEDLDW
San_LOC107690738      -HEMDRRLRRRHPFSG----------------RHPLKVPEVPVSEE----PSEEEEDLDW
Srh_LOC107710048      -HEMDRRLRRRHPFSG----------------RHPLKVPEVPVSEE----PSEEEEDVDW
Dre_Cbx6b             -HEMDRRLRRRHPFSG----------------RHPLKVPEVPVSEE----PSEEEEDLDW
Ame_cbx6b             -HGSDRRGKRHYHFCR----------------HRQPKLPEIPISEE----ASEEEE-LDW
Pna_LOC108413245      -HGSDRRAKRHHHFCR----------------HQQPKLPEIPMSEE----ASEEEEELDW
Ipu_LOC108260787      -HGTDRRARQRQHFRG----------------RRQPKVPEIPISEE----ASEEDQDLDW
Eel_LOC113569862      -HSTERRCRRRNSFGP----------------RRPAEFPENPLPHK----VSEEDEDLDW
Srh_LOC107720374      -RGRKRKLRHRTQVGQ---AS----------VCQVSDRTTTPHPEEN---REPKEGDPDW
San_LOC107667890      -RGRKRKLRHRTQVGR---AS----------VCQVSDRTTTPHPEEN---REPKEGDPDW
Cca_LOC109066300      -RGRKRKLRHRTQVGR---AS----------LCQVSDRTTTPLPVET---REPKEGDPDW
Cau_LOC113065021      -RGRKRKLQHRTQVGR---AS----------VCQVSDCTTTPLPEET---REPKEGNPDW
Sgr_LOC107602676      -QGGKRKLRHRTQVGR---AS----------VCQVSDRNTAPLPEEN---RVPKEGDPDW
San_LOC107704552      -RGGKRKLRHRTQVGR---AS----------VCQVSDRNTAPLPEEN---RVPKEGDPDW
Srh_LOC107717667      -RGGKRKLRHRTQVGR---AS----------VCQVSDRNTAPLPEEN---RVPKEGDPDW
Cca_LOC109066296      -RGGKRKLRHRTQVGR---AS----------VCQVSDRTTAPLPEEN---RVPKEGDPDW
Cau_LOC113039859      -RGGKRKLRHRTQVGR---AS----------VCQVSDCTTTPLPEEN---RVPKEGDPDW
Dre_Cbx6a             -RGRKQELRHQTQVGR---AS----------VCQVSNQTTASFPEKN---RVPKEGDPDW
Ame_LOC103038090      --GGKRKPCRRIRAS--------------------EAATSHPAAEKRAERRALKKADPDW
Pna_Cbx6              PKGGRRKPCRRFRAS--------------------EGANSRPTAEK----KALKRGNPDW
Eel_LOC113585732      PKDGKWKPRRRTRASV---S-----------KSARQGDNSRPLAEK----SAPERGDPDW
Ipu_LOC108259424      ARGRKRKARRRFQTS---------------------NPSSGDGTEK----TAPKPGDPDW
Dcl_LOC114786320      SKGKRRGRHCHHHQSSDCHATLVIANASQPLTQAIDRNAYQPSTEQA---RAPVEGDPDW
Cha_LOC105903838      QKVKRSLRRRRSQAS----QP-TSASDNNTVSSPAEDATSAALPGET---RAPVEGDPDW
                                :                                . :             **
Prim.cons.            PHGM2RRLRRRHQFS2DCHASL22A222222VRQP2KVPEVP2SEENAERRS2EEGDPDW


                               550       560       570       580       590       600
                                 |         |         |         |         |         |
Sgr_LOC107585029      HPDMTSQCANVVVTDITANLLTVTIK-EFCHPPS---------ATPPPCYPKNISAQHDT
San_LOC107662367      RPDMTSQCANVVVTDITANLLTVTIK-EFCHPPS---------ATPPPCYPKNISAQHDT
Srh_LOC107740604      HPDMTSQCANVVVTDITANLLTVTIK-EFCHPPS---------ATPPPCYPKNISAQHDT
Cau_LOC113046746      HPDMTSRCANVVVTDITANLLTVTIK-EFCHPPS---------ATPPPCYPKNISPQHDT
Cca_LOC109095357      HPDMTSQCANVVVTDITANLLTVRSRSSVIHPPLPLL------PATPKIYQLKMTQNSQS
Cau_LOC113047918      RPDMTSKCANVVVTDITANLLTVTIK-EFCHPPS---------VNPPPCYPKNILAQHDT
Sgr_LOC107602318      HPDMTSQCANVVVTDITANLLTVTIK-EFCHPPS---------ATPPPCYPKNISAQHDT
San_LOC107690738      HPDMTSQCANVVVTDITANLLTVTIK-EFCHPPS---------ATPPPCYPKNISAQHDT
Srh_LOC107710048      HPDMTSQCANVVVTDITANLLTVTIK-EFCHPPS---------ATPPPCYPKNISAQHDT
Dre_Cbx6b             RPDMTSRCANVVVTDITANLLTVTIK-EFCHPPS---------ASSPPCYSKNVSTQNDT
Ame_cbx6b             HPEMAAQCANVVVTDVTTNLLTVTIK-EFCHPHGLPP------SSSSPC-----------
Pna_LOC108413245      HPEMVAQCANVVVTDVTTNLLTVTIK-EFCHPHGLPP------SASSPCYANHFSASNKT
Ipu_LOC108260787      HPEMAAQCANVVITDVTTNLLTVTIK-EFCHPPGLPA------VASSPCCANNLPSSKKT
Eel_LOC113569862      RPEMAARCANVVVTDVTTNLLTVTIK-EFCHPPGLPP------PVSSACFSNHLSASNKT
Srh_LOC107720374      NPEMAPSCTNVVVTDVTTNLLTVTIK-EFCHSGA-GS------EPSSPCQADNPPCPTTA
San_LOC107667890      NPEMAPSCTNVVVTDVTTNLLTVTIK-EFCHSGA-GS------EPSSPCLADNPPCPTTA
Cca_LOC109066300      HPQMTPSCTNVVVTDVTTNLLTVTIK-EFCHSGA-GS------ETSSPCQADNPPCPTTA
Cau_LOC113065021      HPEMTPSCTNVVVTDVTTNLLTVTIK-EFCQSGA-GS------KPSSPCQADNPP-----
Sgr_LOC107602676      HPEMAPSSANVVVTDVTTNLLTVTIK-EFCHSGA-GS------EPSSPCQADKPPCPTTV
San_LOC107704552      HPEMAPSCANVVVTDVTTNLLTVTIK-EFCHSGA-GS------EPSSPCQADKPPRPTTA
Srh_LOC107717667      HPEMAPSCANVVVTDVTTNLLTVTIK-EFCHSGA-GS------EPSSPCQADKPPCPTTA
Cca_LOC109066296      HPEMAPSCTNVVVTDVTTNLLTVTIK-EFCHSGA-GS------EPSSPCQADNPPCPTTA
Cau_LOC113039859      HPEMAPSCANVVVTDVTTNLLTVTIK-EFCQS---GA------EPSSPCQADKPPCPTTA
Dre_Cbx6a             HPEMAPSSANVVVTDVTTNLLTVTIK-EFCNSGA-GS------DPSSPCQADNPNSPTTT
Ame_LOC103038090      RPERSPGCINVVVTDVATNHQTVTIK-EFCKPGA-GSKGSTGSSPPSPSQAKNPGSSAPA
Pna_Cbx6              RPERAPSCANVVVTDVATNQLTVTIK-EFCKPGA-SS---TDSTPPSPSQAKSPSFSTLS
Eel_LOC113585732      RPERAPSCANVVVTDVATNHLTVTIK-EFCQPGA-GSAGSVASAPASPSHSENLTSFTPS
Ipu_LOC108259424      HPKRTPTCANVVVTDVATNHLTVTIK-EFCQSRA-AS------GPPSPSHSENAKSSTT-
Dcl_LOC114786320      RPDMAPCCANVVVTDVTTNLLTVTIK-EFCQPTETGS------MPPSPSIN-STPPSVST
Cha_LOC105903838      RPEMAPRCANVVVTDVTTNLLTVTIK-EFCQPAEAGS------GPPSPSATSSSPPPPSQ
                      .*.  . .  ***:**:::*  **  :  ..  :.             ..
Prim.cons.            HPEMAPSCANVVVTDVTTNLLTVTIKSEFCHP2ALGS2GST3SAPSSPC2AKNPPAPTTT
```

```
                            610       620       630       640
                             |         |         |         |
Sgr_LOC107585029    KQPKQHPNKT---------------------------------
San_LOC107662367    KQPKQHPNKT---------------------------------
Srh_LOC107740604    KQPKQHPNKT---------------------------------
Cau_LOC113046746    KQPKDHPNKA---------------------------------
Cca_LOC109095357    NTPTKHECRHHRYALLQPSEGILEGLLVVCLQFQLPGAHVSLC
Cau_LOC113047918    KQPKQHPNKT---------------------------------
Sgr_LOC107602318    KQPKHHPNKT---------------------------------
San_LOC107690738    KQPKHHPNKT---------------------------------
Srh_LOC107710048    KQPKHHPKKT---------------------------------
Dre_Cbx6b           QQPKQNPNKT---------------------------------
Ame_cbx6b           -------------------------------------------
Pna_LOC108413245    KPQP---------------------------------------
Ipu_LOC108260787    NP-----------------------------------------
Eel_LOC113569862    NTRP---------------------------------------
Srh_LOC107720374    AS-----------------------------------------
San_LOC107667890    AS-----------------------------------------
Cca_LOC109066300    AS-----------------------------------------
Cau_LOC113065021    -------------------------------------------
Sgr_LOC107602676    AS-----------------------------------------
San_LOC107704552    AS-----------------------------------------
Srh_LOC107717667    AS-----------------------------------------
Cca_LOC109066296    AS-----------------------------------------
Cau_LOC113039859    AS-----------------------------------------
Dre_Cbx6a           AYK----------------------------------------
Ame_LOC103038090    AT-----------------------------------------
Pna_Cbx6            GT-----------------------------------------
Eel_LOC113585732    SA-----------------------------------------
Ipu_LOC108259424    -------------------------------------------
Dcl_LOC114786320    TSVATESKP----------------------------------
Cha_LOC105903838    AELPSLPLPATSVAQP---------------------------

Prim.cons.          A2PKQHPNKT222A22QPSEGILEGLLVVCLQFQLPGAHVSLC
```

Supplementary Figure S2. ***Sequence alignment for Cbx6 proteins*** from Ostariophysi including zebrafish (Dre, *Danio rerio*), goldfish (Cau, *Carassius auratus*), common carp (Cca, *Cyprinus carpio*), three Chinese barbels (San, *Sinocyclocheilus anshuiensis*; Sgr, *Sinocyclocheilus grahami* and Srh, *Sinocyclocheilus rhinocerous*), cave fish (Ame, *Astyanax mexicanus*), red-bellied piranha (Pna, *Pygocentrus nattereri*), channel catfish (Ipu, *Ictalurus punctatus*), electric eel (Eel, *Electrophorus electricus*) and Clupeomorpha including denticle and atlantic herrings (Dcl, *Denticeps clupeoides*; Cha, *Clupea harengus*). The alignment was performed with the CLUSTALW multiple alignment tool. Sequences of the chromodomain are highlighted in bleu, of the ATHL motif in grey, of the Cx6.1 motif in light green, of the Cx6.2 motif in dark green and of the Pc-box in violet.
