# Supplementary material for: The Polycomb Orthologues in Teleost Fishes and Their Expression in the Zebrafish Model
Source: Genes (Basel). 2020 Mar 27;11(4):362. doi: 10.3390/genes11040362 (PMC7230241; doi:10.3390/genes11040362)
Supplement: Supplementary file 1 [file genes-11-00362-s001.zip › Supp Material/Supp Figure S4.pdf]

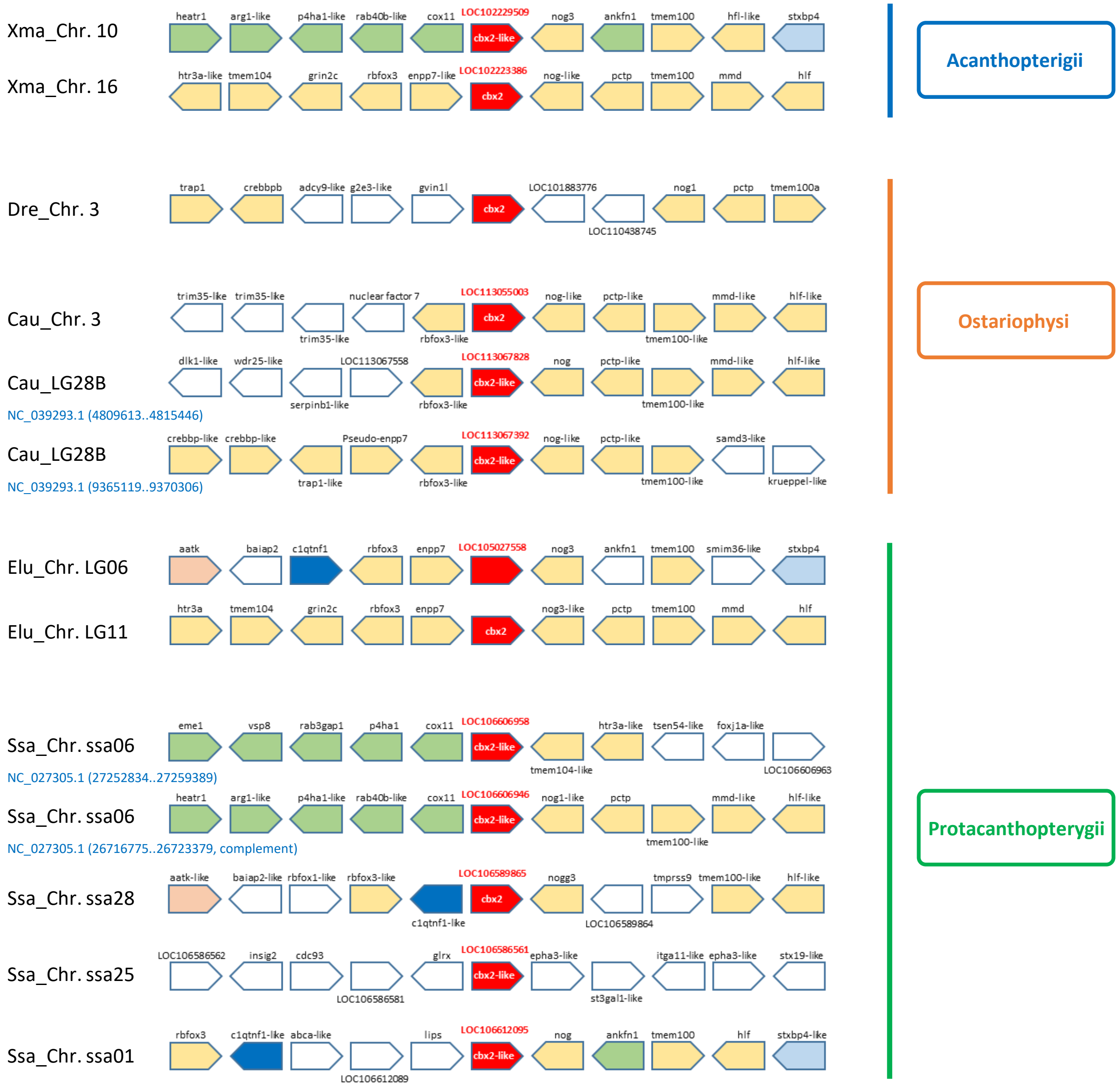

Supplementary Figure S4. ***Blocks of synteny at the cbx2 locus in teleost fishes.*** The cbx2 genes are shown in red while orthologous gene loci are highlighted with different colors. Chromosomal locations of cbx2 and the neighboring gene loci were drawn based on information from the NCBI Gene server. Chromosome (Chr.) or linkage group (LG) numbers are indicated.
