# Supplementary material for: The Polycomb Orthologues in Teleost Fishes and Their Expression in the Zebrafish Model
Source: Genes (Basel). 2020 Mar 27;11(4):362. doi: 10.3390/genes11040362 (PMC7230241; doi:10.3390/genes11040362)
Supplement: Supplementary file 1 [file genes-11-00362-s001.zip › Supp Material/Supp Figure S5.pdf]

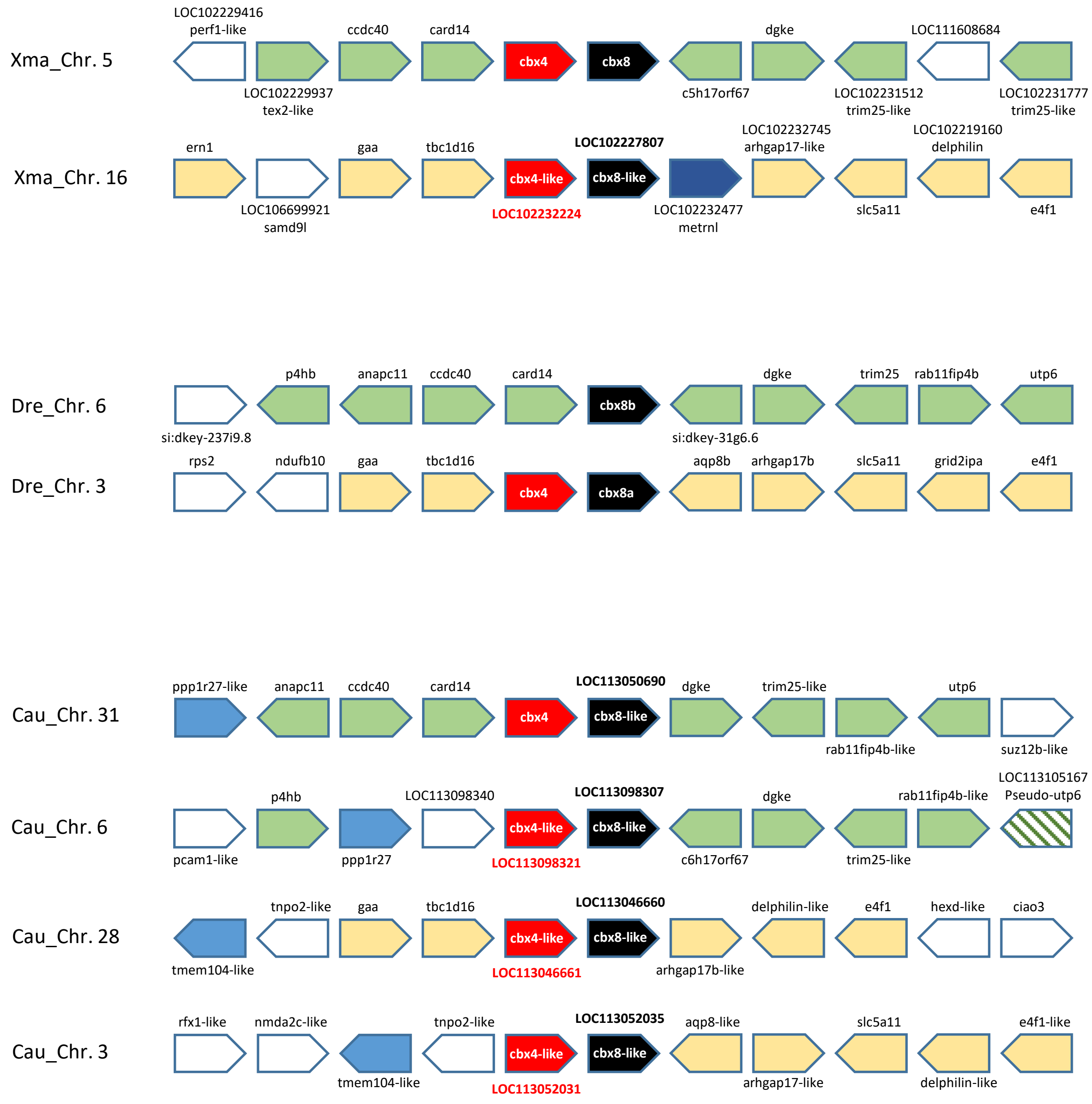

Supplementary Figure S5. ***Blocks of synteny at the cbx4-cbx8 locus in teleost fishes.*** The cbx4 genes are shown in red, cbx8 in black while orthologous gene loci are highlighted with different colors. Chromosomal locations of cbx4, cbx8 and the neighboring gene loci were drawn based on information from the NCBI Gene server. Chromosome (Chr.) or linkage group (LG) numbers are indicated. Xma, platyfish (Xiphophorus maculatus, Cyprynodontiformes); Dre, zebrafish (Danio rerio, Cypriniformes); Cau, goldfish (Carassius auratus, Cypriniformes).
