# Supplementary material for: The Polycomb Orthologues in Teleost Fishes and Their Expression in the Zebrafish Model
Source: Genes (Basel). 2020 Mar 27;11(4):362. doi: 10.3390/genes11040362 (PMC7230241; doi:10.3390/genes11040362)
Supplement: Supplementary file 1 [file genes-11-00362-s001.zip › Supp Material/Supp File S2.pdf]

>Dre_Cbx2 (Gene ID: 327291)
MEELSAVGEQVFDAECILNKRTRKGKLEYLVKWRGWSSKHNSWEPQENLLDPRLLVAFNKREQEKELLISKKGKRPRG
RPRKIMETIPVVSKSSSSSSSSSSSGSSSSSSSSSSSTDDDDEDDHNMTPKPIPRPREHLPVPQKKAQIVVAKPGPPK
KRGRKALPPELKAIRQVKGTRKILKPISRDSDLRGIKKPLMPASFTYTGLNRTSGREPMAMHNRGSFTHKSSLSSLGR
SIGSVSSPPTLNRSPQTKSASDFKLSVSDMNSGLDPKTPTCKSPGVAALNLHSSNGQTCPQLSPTVPKDQTLLQRSAS
LPKSPSSSFSSLKTPSSLQALNLQSVNKTVQGNGTDLKTSPHSGRKSSGFNTSSAPNTPSKFQTSQQALKSPQKLKAD
DLAERLGKKSQARTEKILPTEGRDSQPAQDRPSSKDPSKQSKTLSELSTGEEGSSSDTDHDSSFPRDSHDLSISVQAG
QDWRPTRSLIEHVFVTDVTANLVTVTVKESPTSVGFFSIRNY

>Dre_Cbx4 (Gene ID: 573110)
MELPAVGEHVFAVEGIEKKRLRKGRMEYLVKWRGWSPKYNTWEPEENILDPRLLVAFQNRERQEQMVGYRKRGPKPKH
PLIQLPAFARRSSILGGLQDTSLDEENQPKVDSLQMHRSRPQHYQLNSKKHHQYQPSCKEISVEQHMSGKKKHFYQLN
SKKHHHYQPDPKMYDTPLTGPKEVKGQDPSNKGWNLPPALQQKWIRNKDTGCLSKVKDLSIELKGLPDNANKAERTLK
TSAKEFALPNGISSKMKIIKNKNKNGRIVIVMSKYMDKGVHSSKVKNRDVSQVGTVHNEEPPVNGTDNRSDGETPDQG
EGRENGSIEKPSEYVDEKSLRKDMPGQKATEICDRPNSSPAKVDAKSNHLKRHLSEPSEDVRNCKQLLSFRSISAPNS
SPQREPINLHYKSSPVDNYDFSDSIPEEPIDLSAKTLKRFTALDKVSGSSAQMDKTDSQCKAFVGNVIITDITTNCLT
VTFKEYVQE

>Dre_Cbx6a (Gene ID: 799294)
MELSAAGDRVFAAEAILKSRVRKGHIEYLVKWKGWALKHSTWEPEENILDDRLIKAFEQKEREQELYGPEQELYGPKK
RGPKPKNFVLKARAQSGDRPRSSYTRRTPSCTTAKPPTASSSASSATPQPSSSSSHSTAPTPRVHSLAAAHKLKKDIH
RCHMMSRRPLPRSDPLANSTGSSSRHPISPFSETVRILNRRVKPREVKRGRIILNLKVIDKSENGGMTSRRTPQSFAG
RAKIPSRNRIIGKKHGDMPYRPFQHPMKMLGIPMYGQPFGLNPSGGASSMANEGSNTRASHGGGSRCGSQSSAQRFQY
QPTPSPSNSSGSNGSSPSTQKQPTQKEGPTSSAKLTPTESSRSHDTSQPHPKSSSTPFLPSSPSYSSSPSSSQEDEDL
GSLKSATSRGRKQELRHQTQVGRASVCQVSNQTTASFPEKNRVPKEGDPDWHPEMAPSSANVVVTDVTTNLLTVTIKE
FCNSGAGSDPSSPCQADNPNSPTTTAYK

>Dre_Cbx6b (ENSDARG00000097543)
MELSAAGDRVFAAEAILKRRVRKGRMEYLVKWKGWAIKYSTWEPEENILDERLVAAFEQKEREQEMYGPKKRGPKPKT
LLLKSRAQAESPRVPEFKHSRPQPHSKPPPPPPPAAPSYTPTAPSNAKLQSGTAQPKLKKDIHRCHRMARRPLPRQDH
TVGPSGPFSSRPTVSAFSETVRILNRKVKPREVKKGRVILNLKVLDKAENTAVANNKRTHVPSTQQSHFGRQKIPSRN
RVIGKNRRFGEVSYRGIQPPIRGSGFPVFGKLFDSHSLKNAESGESRNSMTNNLSSSQSSKLLDVSKGQNRNEEPPPS
NSSSEVSDGEPPSPRQTQSQHSSLQPKASSTKSPSTKAPDPTLHKLGTQPVASKNKDSASSALPSSPMFSSSSSASTS
SEDNEHILDLSVPHEMDRRLRRRHPFSGRHPLKVPEVPVSEEPSEEEEDLDWRPDMTSRCANVVVTDITANLLTVTIK
EFCHPPSASSPPCYSKNVSTQNDTQQPKQNPNKT

>Dre_Cbx7a (Gene ID: 550551)
MELSSIGEQVFAVESITKKRIRKGNVEYLLKWQGWSPKYSTWEPEDNILDPRLVLAFEEKAEKDRALAYKKKGLRPRQ
VILRNIYPMDLRSAHKVPDKPSPRIRLSLTRSMSTEVDQNRRRYRDSVVYRRLKNRYKNRQCRSRLFEGIKPSKQPMR
HPLPAKDCTENAWNEDEQKRKVKKMRKDEENTTQVHQDIPSGQEMSEGYNSSAEQESEITIKEDENCSSIFDQEEKPS
EDTGTAIGAPESSTITDTEKNEPVTNTAGDEDCVWVSHDTTNTDQSLHKCTKSGAEEGVFDRVQNRPSVIEVRLSATC
GQEEVRESAEVGSSEAKDKEGIDAEVTAEFQVPIDQLQTPVTTAVHPGKVIVTQVTINSLTVTFKEAMTAEGFFSECG
LEV

>Dre_Cbx7b (Gene ID: 101882803)
MELSAIGEQVFAVESITKKRVRKGHVEYLLKWKGWPPKYSTWEPEEHILDPRLVLAYEEKEQKERSVVWRKRGRKPKR
LHEQRSIYTMDLRSTHRHTDQSSAHLPLSLDPRFQSTEACIFQQLNHHKKKKRAETSEEEWDRREEEDDDEDDEGVME
EGEEEETRLGGTKTGSSTLNKHVRREDWSPTTESEAMSLPEDWSPVMGPEEVMVTDITINSLTVTFREALVARGFFSS
WEMEI

>Dre_Cbx8a (Gene ID: 100150672)
MELSAVGERVFAAESIIKRRIRRGRMEYLVKWKGWSQKYSTWEPEENILDERLFAAFEEREREREMYGPKKRGPKPET
FLMKAKAKAKSKTYEFGREMSRDIRVSFPVAEPVVTPRAREGLRTVVPTIFPPSTINRGESVRVRPPDSERDTLTHGT
SIQCPLDFTNSPKKRGPKPKMHPAGGSSSEGIKRKADESLSYRPSKTERSGETSNCDIMHFTQKYKAETNHDGKQMGS
RSSDVKFSHGGSFLKAGLGILGHRQKVGSGGAINQQAKMKHPPKNNLFRSTDQTREQLSLSFVDDTDQSWLPCLKNME
KIVVTDVTSNSLTVTIKESSTDKGFFKENR

>Dre_Cbx8b (Gene ID: 799361)
MELSAVGERVFAAESIIKRRIRRGHMEYLVKWKGWSPKYSTWEPEENILDPRLFVAFEEREREREIFGPKKRGPKLKT
FLLKAQAKEKAKSYEFRNDSSRGIHVTYSSPEPVVAPRAREGLRAVVPTIFPPSTVNRGESVRLQSPEPREHHSPHSP
RPTDEFSLTPKKRGRKPKLRFTDGYTSNLHPEHVKRAADETMASIPSKMAKLGLGEDDERCPDISGRLKISHKDLDAT
CSHKQSNVIPSRSTLHISPQWSLHSSRMEAGLLGHRTNPQSNHFHQKHLKHYSKKRTLEHGDSTSRQPSLIAKIPVSH
IFREPGVEDAWRPSVINVEKVIVTDVTTNFLTVTIKESNTDEGFFKDKT

>Cca_LOC109087174

MRTVMEELSAVGGQVFDAECILNKRLKKGKLEYLVKWRGWSSKHNSWEPQENLLDPRLLAAFNKSLKLSVSDMSSSGV
DPTTPTCKSPGVAALNLHSSNGQTCPQLSPNVPKASDQTLLQRSGSLQKSPSSSFSSLKTPSSLQALNLQSITKTAQG
NGTSTNDGSYLKDTSNPSRKSSGFNTRHEQSPALNVPSKFPTSQQVLKSPQLNKSKVDDLSERLGKKNQGRVDKILIQ
TAEGRDHPAQDRSSSKDPGKPSKTLSELSTGEEGSSSDSDHESSFPSDNRDLAISVQAGQDWKPTRNLIEHVFVTDVT
ANLVTVTVKESPTSVGFFSIRNY

>Cca_LOC109046180
MRRVMEELSAVGEQVFDAECILNKRLKKGKLEYLVKWRGWSSKHNSWEPQENLLDPRLLAAFNKRKSSNTPTCKSPGV
AALNIHSSNGQTCPQLSPNVLKASDQTLLQRSGSLQKSPSSSFSSLKTPTSLQALNLQSINKTALGNGTSANDGSYLK
GAYNPDKKSSGFNPRREHSPAQNAPSKFPTNQQVLKSPQRDKSKAAYLSERLGKKNQGRVDNIVVPTSEGRDQPVLDR
SSYKDAGKPSKILSELSTGEEGSSSDSDHDSTFRSDNHDLAISVQAGQDWKPTRSLIEHVFVTDVTANLVTVTVKESP
TSVGFFSIRNY

>Cca_Cbx4 (Gene ID: 109102575)
MELPAAGEHVFAVESIEKKRIRKGRCEYLVKWRGWSPKYNTWEPEENILDPRLLVAFQNRERQEQLIGYRKRGPKPKH
LLLQVPSFARRSSVLSDLQEVSHGEDYQPKATSDLVQSQQYQLNGKKHHPYQPNPKDKQGEAQISGKKKHYYQLNSKK
HHHYQPNPKMYNSLYQRVKETKVPELTDKEWNLSPALQQKWIQDKDSGCLSKVRNITMGLKKLPKLNGEADLNMNTNS
DAKEDKAPPNGISSKLKIVKNKNKNGRIVIVMSKYMENGTQAAKIKEGTADSEEKGQAQDCKSCGTDESTEKIKHTKK
QSLVKGIKKDSKAGTACFMLSNGLSSCGEDNPKKACSILFEPNNLKKPGNTGDYVSNEQHLQLTTKTSLTTVPFEKSD
QRGTQSTQYGPITFPQKRCYSEPDSDSREAKRFLSSQSISAPNTDLSYSCNDTTHFNDHQHSSGHDFEILDSNQDEPI
DLSCIRSREERKIATHSQIGNSIPTGKATVSTPEQVEKEMKKSVQSFTPFLGNIIITDVTANCLTVTFKEYVTV

>Cca_LOC109092326
MGYRKRGPKPKHLLLQVPSFARRSSVLTDLQEVSQSEDNQSKTTSDLVPSQQYQLNSKKHHPYQPNPKDKQGEARING
KMKHYFQLNSKKHHHYQPNPKMYDSLYQRVKETKVPELTDKEWNLSPALQQKLIQSKDSECISKVRNITMELKKLPKL
NGGADLNMNTNSDAKEDKAPPNGVRSKLKIVKNKNKNGRIVIVMSKYMENGTQAAKIKKGTADSEGKRQAQDCKSCGT
DESTEKIKHTKKQSVVKVIKKDSKAESACFRLSNGLSSCVGDNPKKTCSIVFEPNNSKKPGSTGDYVSNEQPLQLTNK
SVRQTSLTTVPIETSDQKGTQSTQYGPLSLPRKRCYSEPESDSGEVKRHLISQSTNTPNTDSCNGTTHFNDHQHSCGH
DFENLDSNQDEPIDLSCVRLREERKNPTRSQIGNSIPTGKAAVSAPEQVEKEMKKSVQSFTPFLGNIIITDVTANCLT
VTFKEYVTV

>Cca_LOC109066300
MEVSAVGERVFPAEAILKSRVRKGRIEYLVKWKGWALKHSTWEPEENILDDRLITAFEKKEREQELYGPKKRGPKPKN
FVLKARAQTGDRPRSSDTQRTPPQIPAKLPASSSSASSAPSQPSSSSSLSTTPTPRVHSLAAAHKLKKDIHRCHRMSR
RPLPRPDPLGEPTGSTSSSRPPISPFSETVRILNRRVKPREVKRGRIILNLNVIDKSENSGVTSRRSPQSFAGRAKIP
SRNRIIGKKQGDMPYRPFQHPMKMLGFPMYGQPFGLHPCGSVSSMANEESNTGANRRGGNCCDSHSSANVQKFQYQTP
PSPSSSSGSNNSSPSLQKQPTQPEAPTSPTKLDSAGSSRSQDASQPHPKSSSAPFLPSSPSYLSSPSFSLDDEDQSSP
NLTTSRGRKRKLRHRTQVGRASLCQVSDRTTTPLPVETREPKEGDPDWHPQMTPSCTNVVVTDVTTNLLTVTIKEFCH
SGAGSETSSPCQADNPPCPTTAAS

>Cca_LOC109066296
MELSAEGERVFAAEAILKSRVRKGRIEYLVKWKGWALKHSTWEPEENILDDRLITAFEQKEREQELYGPKKRGPKPKN
FVLKARAQAGDRPRSSNTQRTPPRTTAKPPASSSAASAAPPQPSSSSSYNTAPTPRVHSLAAAHKLKKDIHRCHRMSR
RPLPRPDPLGDPTGSTSSSRPPISPFSETVRILNRRVKPREVKRGRIIVNLKVIDKSENSGVASRKTPQSFMGRAKIP
SRNRIIGKKHGDMPYRPFQHPMKMLGFPMYGQPFGLNPCGSMSSMANDGSNTVVNQRGGGCCDSHSSANAQKFQYQPP
PSPTSSSGSINITPSLQKQATQPEAPTKLDSAASSRSRDASQPHPKSSSAPFLPSSPSYSSSPSSSLEDEDQGSPNLT
TSRGGKRKLRHRTQVGRASVCQVSDRTTAPLPEENRVPKEGDPDWHPEMAPSCTNVVVTDVTTNLLTVTIKEFCHSGA
GSEPSSPCQADNPPCPTTAAS

>Cca_LOC109095357
MELSAAGDRVFAAEAILKRRVRKGRMEYLVKWKGWAIKYSTWEPEENILDERLVAAFEQKEREQEMYGPKKRGPKPKT
LLLKSRAQVAETSSRVPEFKHTRPQQHSKLPPPSAAPSYTPTAPSNAKLQSGTAQPKLKKDIHRCHRMARRPLPRPDQ
TVGPSGPFSSRPTVSPFSETVRILNRKVKPREVKKGRVILNLKVVDKAGNGTVANSRRTHVPSAQQSHFGRQKIPSRN
RVIGKNRRFGELSYRGIQTPIRSSGFPVFGKLFDSHSLNNAENQTQSGESRNNMTNTLSSSQCSKLDASKGQTLDELP
PSNSSSEVSDGEPHSPPQTQSQHSSLQPKSSATKAPDPTLHKLSGQTVPSKNSLAPSALPSSPMFSSSSSASSSSEDN
ERILDLSVPHEMDRRLRHRHPFSGRHPLKVPEVPVSEEPSEEEEDLDWHPDMTSQCANVVVTDITANLLTVRSRSSVI
HPPLPLLPATPKIYQLKMTQNSQSNTPTKHECRHHRYALLQPSEGILEGLLVVCLQFQLPGAHVSLC

>Cca_LOC109100093
MELSAIGEEVFAVESIIKKRVRKGHVEYLLKWKGWPPKYSTWEPEEHILDPRLVLAYEEKEQRERSVRCRKRGPKPKR
LFMQQNIYAMDLRSAHKDAEKSSAHLPLSLDPRFQSTGACIYQRLTHHKKKKKTSRETSEEEWDRREEEDDDDDDEGV
MEEEEEEEEEEEDTRQGGIKTGSNTLNKHVRRRCWSPTTESGAMTISPLPEDWSPVMGPEEVIVTDITINSLTVTFRE
ALVARGFFRSWEMEI

>Cca_LOC109076671
MNMELSSIGEQVFAVESITKKRIRKGNVEYLLKWQGWPPKYSTWEPEDNILDPRLVLAYEEKEEKERALAYKRKGLRP
RRVILRNLYPMDLRSKHKVSDKSTPRIRLSLTRSMGTEIDQNGRRCQRIEKRKNRQYRSKLMNDIKRFQQPRRHPLLL

KDSEKEWDGDEEDEQRKKVKKMRTNGEKTTEVHQDIPSGQEMSEGYNSSAEHEAVITIEETENCSSIFDHAEKPSEYT
GPVLGATVNSTITNTQENTPVTNTAGDEDSVRVSHDMTNTDQSLHNSTKSGAKQGVFDKVQNRPSVIEVHSSAKCRQD
EVSERARLDSSEAKATTTKEGMDAVVTAECTLLQVPVDQSQTPSTTTVHPGKVIVTQVTINSLTVTFKEAMSAEGFFS
GYGL

>Cca_LOC109061103
MNMELSSIGEQVFAVESITKKRIRKGNVEYLLKWQGWAPKYSTWEPEDNILDPRLVLAYEEKEEKDRALAYKRKGLRP
RRVILRSIYPMDLRSKHKVPDKPIPRIRLSLTRSMGTEIDQNGRHYRPIDKYKNRQCRSKLMDAIKLSQHSLPAKDSE
KEWDGDDEDDEQKNKVKKMRENEEKTTEVHQDIQSSQEITEGYSSSAEHEAVIIIEETENCSSIYDHAETPSEDTSQV
LGATENSTITNTQENEPVTNTTGGEDSIRISHDTTNTDQSLHNSTESGAKQGVFDKVQNRPSVIEVHSSTKCRQEEVS
EGGEADHSEAKEKEGMDADITAECTLLQVPTDQSQTPSTTTVDPEKVTVTQVTINSLTVTFKEAMSAEGFFSGDGLEI

>Cca_LOC109102576
MELSAVGERVFAAESIIKRRIRRGRMEYLVKWKGWSPKYSTWEPEENILDSRLFVAFEEREREREIFGPKKRGPKLKT
FLLKAQAKEKSKSYEFRNDLSRGITYSSPEPVVTPRAREGLRAVVPTIFPPSTVNRGESVRIPPPESREHRSPHSPRP
STDAFTLTPKKRGRKPKLCFTDGFTSSLHPEHSKRAADETMASIPSKMAKLGLGEEEERCSEISGRIKISHKHMDATY
SHKQNRVVPSRSTQHISSEWSLHSSRVSADLQGHRNNPQTSHFHHKHLKYHSKRRTFEHADSTNRHPSLIAKIPVSRI
FGEPDEGDAWRPSFSNVEKVVVTDVTSNFLTVTIKESSTDKGFFKDKR

>Cca_LOC109092325
MELSAVGERVFAAESIIKRRIRRGRMEYLVKWKGWSPKYSTWEPEENILDSRLFVAFEEREREREIFGPKKRGPKLKT
FLLKAQAKEKVKSFEFRNESSRDVSYPSPEPVVTPRAREGLRAVVPTIFPPSTVNRGESVRIPPPEPREHRSPHSPRP
STDAFTFSPKKRGRKPKLRFTDGYTSSLHPKHSKRAADETMASIPSKMAKLGLGEEEERCSEISGRIKISHKHMDATY
SHKQNRVVPSRSTQHISPEWSLHSSRVSADLQGHRTNPQTSHFHHKHLKHHSKRRTFEHGDSTSRQPSLIAKIPVSRI
FGEPEEGDAWRPSLCNVEKVIVTDVTSNFLTVTIKESSTDKGFFKDKR

>Cau_LOC113055003
MRRAMEELSAVGEQVFDAECILNKRIKKGKVEYLVKWRGWSSKHNSWEPQENLLDPRLLAAFNKREQERELLISKKGK
RPRGRPRKILEIIPVVSKSSSSSSSSSSSGSSSSSSSSSSSSDDDDEEDDNDRNPKLSPHPREHHPVPQKKAQILVAK
PGPPKKKRGRKALPPELKAQRQAKGPRKFLKPMSRHSELRGSIKKPLMPASFTYTGLNRISGREPLALQNRGSFTQKS
SLSSLGRSVGSTSSPTVLSRPPQSKTASDFKLSVSDMSSGDIDPSTPTCKSPGVAVLNIHSSNGQTCPKLSPNVLKAS
DQTLLQRSGSLQKSPSSSFSSLKTPTSLQALNLQSINKTAQGNGSSANDDSYLKGAPNPGKKSSGFNPRCEHSPAPNT
PSKFPTNQQVLKSPQRDKSKADYLSEKLGKKNQGRVDNILVPTSDGRDQPVPDRSSYKDAGKPSKILSELSTGEEGSS
SDSDHDSSFPSNNRDLAISVQAGQDWKPTRSLIEHVFVTDVTANLVTVTVKESPTSVGFFSIRNY

>Cau_LOC113067828
MEELSAVGGQVFDAECILNKRLRKGKVEYLVKWRGWSSKHNSWEPQENLLDPRLLAAFNKREQERELLIRNKGKRPRG
RPRKILETIPVVSKSSSSSSSSSSSGSSSSSSSSSSSSDDDDNEDDNDDRNPKPSPRPREHHPVPQKKAQIVVAKPEPP
KKKRGRKALPPELKVQRQTKGPRKMLKPFSRDYEVQGSIKKPLMPASFTYTGLNRNSGREMMTLQNRGSFTQKNSLSS
LGRSVGSASSPPTLSRLPQTKTASDFKLSVSDMSSRGVDPKTPTCKSPGVAALNLHSSNGQTCPQLSPNVPKASDQTL
LQRSGSLQKSTPSSFSSLKTPSSLQALNLQSINKTAQGNGNSTNDGSHLKGTSNHSRKSSGLNTKHEQSPAPNVPSKF
PTSQQVLKSPQRDKSKPEDLSERLGKNNQGRVDNILPQTTEGRDQPAQDRSSSKDAGKPSKTLSELSTGEEGSSSDSD
HDSSFPSNNRDLAISVQAGQDWRPTRNLIEHVFVTDVTANLVTVTVKESPTSVGFFSIRNY

>Cau_LOC113067392
MRTVMEELSAVGGQVFDAECILNKRLRKGKVEYLVKWRGWSSKHNSWEPQENLLDPRLLAAFNKREQERELLIRNKGK
RPRGRPRKILETIPVVSKSSSSSSSSSSSGSSSSSSSSSSSSDDDDNEDDNNDRNPKPSPRSREHHPVPQKKAQIVVAK
PEPPKKKRGRKALPPELKMQRQTKGPRKMLKPFSRDYEVQGSIKKPLMPASFTYTGLNRNSGREMMTLQNRGSFTQKN
SLSSLGRSVGSASSPPTLSRLPQTKTASDFKLSVSDMSSRGVDPKTPTCKSPGVAALNLHSSNGQTCPQLSPNVPKAS
DLTLLQRSGSLQKSPPSSFSSLKTPSSLQALNLQSINKTAQGNGNSTNDGSYLKGTSNPSRKSSGLNTKHEQSPAPNV
PSKFPTSQQVLKSPQRDKSKADDLSERLGEKNQGRVDNILPQTTEGRDQPAQDRSSSKDAGKPSKTLSELSTGEEGSS
SDSDHDSSFPSNNRDLAISVQAGQDWRPTRNLIEHVFVTDVTANLVTVTVKESPTSVGFFSIRNY

>Cau_Cbx4 (Gene ID: 113050689)
MELPAAGEHVFAVESIEKKRIRKGRYEYLVKWRGWSPKERQEQLIGYRKRGPKPKHLLLQVPSFARRSGVLSDLQEVS
QGDDYQPKATSDLVQSQQYQLNSKKHHPYQPNSKDKQGEARINGKKKHYYQLNSKKHHHYQPNPKMYDSLYQRVKETK
VPELTDKEWNLSPALKQKWVKDKDSGCLSKVRNITMGLKKLPKLNGEADLIMNTNSDAKEDKAPPNGISSKLKIVKNK
NKNGRIVIVMSKYMEKGTHAAKIKEGTADSEEKRQAQNCKPCGTDGSTQKIKHTKKQSLVKGIKKDSKAETACFRLSN
GLSSFGGDNPKKACSIVFEPNNLKKPGNTRDDLSHEQPLQLTTKTSLTTVPFEKSDQRGIQSTQHGPITLPQKHCYTE
PESDSREAKRFLSSQSISPPNTDLSYSCNDTTHFNDHQHSSRHDFEILDSNQDEPIDLSCVRSREERKIPTHSQIGNS
IPTVKEAVSTPEQVEKEMKKSVQSFTPFLGNIIITDVTANCLTVTFKEYVTV

>Cau_LOC113098321
MELPAAGEHVFAVESIEKKRIRKGRCEYLVKWRGWSPKYNTWEPEENILDPRLLVAFQNRERQEQLMGYRKRGPKPKH
LLLQVPSFARRSSVLTNLQEVSQGEDNQSKTTSDLVQSQQYQLNSKKHHPYQPNPKDKQGEARINGKMKHYFQLNSKK
HHHYQPNPKMYDSLYQRVKETKVHELTDKEWNFSPALQQKVIQSKDSECLSKVRNITMELKKLPKLNGGADLNMNTNS
DAKEDKAPPNGISSKLKIVKNKNKNGRIVIVMSKYMENGTQAAKIKKGTADSKEKRQAQDDSTEKIKHTKKQSLMKVI
KKDSKAESACFRLSNGLSYCGGDNPKKTCSIVFEANNSKKLGSTGDFISNKQPLQLTNKSVRQTSLTTVPIETSDQKG

TQSTQYGPLTLPQKRCYSEPDSDSGEAKRLLISQSANAPNADFSHSCNSTTHFNGHQQSGGHDFEILDSNQDEPIDLS
CVRLREEKKNPTHSQIGNTIATGKAAVSAPEQVEKEMKKSVRSFTPFLGNIIITDVTANCLTVTFKEYVTV

>Cau_LOC113052031
MDLPAVGEHVFAVEGIEKKRLRKGRIEYLVKWRGWSAKYNTWEPEENILDPRLLVAFQNRERQEQMVGYRKRGPKPKN
PLVQLPAFARRSSILGGLQDTSLDEENQPKVEPLQLHCSRPQHYQLNSKKHHQYQPSCREISIEQNVSGKKKHFYQLN
SKKHHHYQPDPKMYDTPLTGPNEVRVQDPSSKVWNLPPALQQKWIRNKDTSCLSKVKDLSIELKSLPDNADKAERALK
TNTKEFALPNGIRSKMKIIKNKNKNGRIVIVMSKYMDKGVHSSKVKNKDVSQVETQHNEESTLNNKDNLSDGETSEGR
ENGTAENTNTFSSSSECIHENSIKMAELSKATSTETEQTVMYICNQPNSSPMEAEKAPPSHMDTKLNQRKRNLSEPIE
DVRNCKQFISSRIIHTNSVQDYAPNTMLSSPQREPIDLHYRGCLASRAYSYDLSDTSPEEPIDLSCGPTKTLKQFPTA
EKVSGSSVQVENTNNHFKPFVGNVIITDITTNCVTVTFKEYVHG

>Cau_LOC113046661
MDLPAVGEHVFAVEGIEKKRLRKGRIEYLVKWRGWSAKYNTWEPEENILDPRLLVAFQNRERQEQMVGYRKRGPKSKH
PLVQLPAFARRSSILGGLEDTSLDEENQPKVEPLQIQHSRPQHYQLNSKKHHQYQPSCREISLEQHVSGKKKHFYQLN
SKKHHHYQPDPKMYDTPLTRPKEVKVQDPSNKGWNLPPAFHQKWIRNKDSGCLSKVKDLSIELKSLPDNGNKAERALK
TSAKEFALPNGISSKMKIIKNKNKNGRIVIVMSKYMDKGVHSSKVKNKDASKMDAEHNEKSTLNKTDNLSDAETSAGR
ENGSVENAGTFSSSSECIHKTSSKKAELPKDTPSETEQMVIDLCDQPNAENAAPAHPDTKLNHRKRNLSEPKEEVRNC
KQFFSSRSISAPNTVLSSPQREPMNLHYSRSLASRAYSYDFSDPIPEEPIDLSCGPTKTLKQFPTAEKVSGSSAQVEK
TNSHGKPFVGNVIITDITTNCLTVTFKEYIQG

>Cau_LOC113065021
MEVSAVGERVFPAEAILKSRVRKGRIEYLVKWKGWALKHSTWEPEENILDDRLLAAFEQKERQQELYGPKKRGPKPKN
FVLKARAHAGDRPRSSDTQRTPPQISNTQRTPPRITAKLPSSSSSASSAPPQPSSSSSFSTAPTPRVHSLAAAHKLKK
DIHRCHRMSRRPLPRPDPLGEPTGSTTSSRPPISPFSETVRILNRRVKTREVKRGRIILNLNVIDKSENSGVTSRRSP
QSFGARAKIPSRNRIIGKKQGDMPYRPFQHPMKMLGFPMYGQPFGLHPCGSVSSMANEESKTGANQRGGNCCDSHSSA
STQKFQYQHPPSPSSSSGSNNSSLSLQKTQTPLEAPMSPTKLDSSASSRSQDSSQPHPKSSSAPFLPSSHSYSSSPSF
SLEDEDQGSQNLTTSRGRKRKLQHRTQVGRASVCQVSDCTTTPLPEETREPKEGNPDWHPEMTPSCTNVVVTDVTTNL
LTVTIKEFCQSGAGSKPSSPCQADNPP

>Cau_LOC113039859
MELSAEGERVFAAEYILKSRVRKGRIEYLVKWKGWALKHSTWEPEENILDDRLITAFEQKEREQELYGPKKRGPKPKN
FVLKARAQAGDRPRSSNTQRTPPRTTAKPPASSTSASAAPPQPSLSSSYNTAPTPRVHSLAAAHKLKKDIHRCHRMSR
RPLPRPDPLGNPRGSTSSSRPPISPFSETVRILNRRVKPREVKRGRIILNLKVIDKSENSGVASRRTPQSFTGRAKIP
SRNRIIGKKHGDMPYRPFQHPMKMLGFPMYGQPFGLNPCGSMSSMANEGSNTGVNQRGGGCCDSHSSASTQKFQYQPP
PSPSSSSGSNNSPPSPQKQATQPEAPTKLDSAASTCSQDASQPHPKSSSAPFLPSSPSYSTSPSSSIEDEDQGSPNLA
ISRGGKRKLRHRTQVGRASVCQVSDCTTTPLPEENRVPKEGDPDWHPEMAPSCANVVVTDVTTNLLTVTIKEFCQSGA
EPSSPCQADKPPCPTTAAS

>Cau_LOC113046746
MELSAAGDRVFAAEAILKRRIRKGRMEYLVKWKGWAIKYSTWEPEENILDERLVAAFEQKEREQELYGPKKRGPKPKT
LLLKSRAQVADTSSRVPEFKHTRPQQHSKLPPPSATPSYTPTVPSNAKLQSGTAQPKLKKDIHRCHRMARRPLPRPDQ
TIGPSSPFSSRPTVSPFSETVRILNRKVKPREVKKGRVILNLKVVDKAGNGAVANSRRTHVPSTQQSHFGRLKIPSRN
RVIGKNRRFGEVSYRGIQPTIKSSGFPVFGKLFDSHSLSNAENQTQSGESRNNTTNNLASSQSSKADVSKGQTLDELP
PSNSSSEVSDGEPPSPPQTQSQHSSLPPKASATKAQDPALHKLSAQPAASKNSSGPSALPSSPMFSSSSSASSSSEDN
EHILDLSVPHEMDRRLRRRHPFSGRHPLKVPEVPVSEEPSEEEEDLDWHPDMTSRCANVVVTDITANLLTVTIKEFCH
PPSATPPPCYPKNISPQHDTKQPKDHPNKA

>Cau_LOC113047918
MELSAAGDRVFAAEAILKRRVRKGRMEYLVKWKGWAIKYSTWEPEENILDERLVAAFEQKEREQEMYGPKKRGPKPKL
LLLKSRAQVAETSSRVPEFKHTHPKQHSKFPPPSAAPSYTPTAPSNAKLQSGTAQPKLKKDIHRCHRMARRPLPRPDQ
TVSPSGPFSSRPTVSPFSETVRILNRKVKPREVKKGRVILNLKVVDKAGHGGVPNSRRTHVPSAQQSHFGRQKIPSRN
RVIGKNRRFGEFSYRGIPTPNRNSGFPVFGKLFDSHSLNNAENQTQSGESRDNTAKNLSSSKNQTLDELPPSNSSSEV
SDGEPNSPPQTQSQRSSLQPKSSTIKAPDPTLHKHSAQTVPSENSLVPTSLPSSPMFSSSSSASSSSEDNERILDLSV
PHEMDKRLRRRHPFSGRHPLKVPEVPVSEEPSEEEEDLDWRPDMTSKCANVVVTDITANLLTVTIKEFCHPPSVNPPP
CYPKNILAQHDTKQPKQHPNKT

>Cau_LOC113047419
MNMELSSIGEQVFAVESITKKRIRKGNVEYLLKWQGWAPKYSTWEPEDNILDPRLVLAYEEKEEKERALAYKRKGLRP
RRVILRSIYPMDLRSKHKVPDKPIPRIRLSLTRSMGTEIDQNGRHYRPIEKYKNRQCRSKLLSDIKLSQHSPPAKDSE
KEWDGDDEDEQKKKVIMMIENEEKTTEVHQDIPSSQETMDGYSSSAEHEAVIIIKETENCSSIYEHAEKPSEDSSQVL
GATETSPISNTQENEPVTNTTGGEDSIRVSHDTTNTDHSLHNSTESGAKQGVLDKVQNRPSVIEVHSSTKCRQEEVRE
RDEVDRWEAKEKEGMDGDITAEHTLLQVPTDQSQTPSTTTVHPGKVTVTQVTINSLTVTFKEAMSAEGFFGDDELEI

>Cau_LOC113046823
MNMELSSIGEQVFAVESITKKRIRKGNVEYLLKWQGWPPKEEKERALAYKRKGLRPRQVILRNLYPMDLRSKHKVSDK
STPRIRLSLTRSMGTEIDQNGRRCQRLEKRKNRRYRSKLMNDIKRFQQPRRHPLLLKDSEKEWDGDEEDEQKRKVKKM
RANEENTPEVHQDIPSGQEMSEGYNSSAEHEAVITIEETENCSSIFDHAEKPSKDTGPVLGATVYSTITNTQEKEPVT

NSAGGEDSVRVSHDTTNTDQSLHNSTKSGAKQGVFDQVKEQNRPSVIEVHSSVKCRQDEVSERGKLDSTGAKEMEGMD
AVVTADCTLLQVPVDQSQTPSTTTVHPGKVIVTQVTINSLTVTFKEAMSAEGFFNGYGL

>Cau_LOC113111712
MELSAIGEQVFAVESIIKKRFRKGHVEYLLKWKGWPPKYSTWEPEEHILDPRLVLAFEEKEQKERSVRWRKRGPKPKR
LFMQRNIYAMDLRSAHKDTEKSSAHLPLSLDPRFQSTGACLYRQLTHHKKKKKASRETSEEEWDRREEEEDDDDDDEG
VMEEEEEEEEEEDTRHGGTKTGSNTLNKRVRRGRWSPATGSEAMTISPLPEDWSPVMGPEEVIVTDITINSLTVTFRE
ALVARGFFRSWEMEI

>Cau_LOC113056054
MMELSAIGEQVFAVESIIKKRVKKGHVEYLLKWKGWPPKEQREMSVRCRKRGPKPKRLFMQSNIYAMDLPSAQKDAEE
LSLDPRFQSTGACIYQRLTLHEKKKKKSRETSEEEWDRREEEDDDDDDEGVMEEEEEEEEDKRQGGTKIGSNTLNNHI
RRGCWSPTTESEAMTRSPLPEDWSPVMGPEEVIVTDITINSLTVTFREALVATGFFRSWEMEIDHYRG

>Cau_LOC113052035
MELSAVGERVFAAESIIKRRIRRGRMEYLVKWKGWSQKYSTWEPEENILDERLFAAFEEREREREMYGPKKRGPKPET
FLMKAKAKAKGKNFEFRQEMSRDLHVSFPVAEPVVTPRAREGLRTVVPTIFPPSTINRGESVRVRPPDPERDPLMHGM
LVQRPLDFASSPKKRGPKPKLRPGGSSTEGVKRKADEPLSYRPSKSERLGETSNCDVTQLTKKFQAESSHVQKQMGSR
SSDVKFTHGGTILKAGLGVLGPQRKDSPSGAIKHSKMKHPPKSNLFRSTDQTREQLSLSFIDETDQSWLPCLKNMEKI
VVTDVTSNSLTVTIKESSTDKGFFKENR

>Cau_LOC113046660
MELSAVGERVFAAESIIKRRIRRGRMEYLVKWKGWSQKYSTWEPEENILDERLFAAFEEREREREELYGPKKRGPKPET
FLMKAKAKGKNYEFRREMSRDLHVSFPVAEPIVTPRAREGLRTVVPTIFPPSTINRGESVRVRPPDFERDPLTLDFAS
SPKKRGPKPKLRPGGSSTEGVKRKADEPLSYRPSKSERSGETSNCDVIHVTQKFPAESSLVQKPSDVKFTHGGTILKP
GPGVVGHRRKDSSSGAINQSKMKHPPQNSLFRSTAQTREQLSLSFVDETDQSWLPCLKNMEKIVVTDVTSNSLTVTIK
ESSTDKGFFKENR

>Cau_LOC113098307
MELSAVGERVFAAESIIKRRIRRGRMEYLVKWKGWSPKYSTWEPEENILDSRLFVAFEEREREREIFGPKKRGPKLKT
FLLKAQAKEKEKSYEFRNESSSGIAYPSPEPVIAPRAREGLRAVVPTIFPPSTVNRGESIRIPPPEPREHRFPHSPRP
STDAFTLSPKKRGRKPKLRFTDGYTSSLHPEHSKRAADETMASIPSKMAKLGLGEEEERCSEISGRIKISHKHMDATF
SHKQNRVALSRSTQHISPEWSLHSNRVSADLQGHRTNPQTHHFHHKHLKHHSKRRKFEHGDSTSKQPSLIAKIPVSRI
FGEPEEGDAWRPSFCNVEKVIVTDVTTNFLTVTIKESSTDKGFFKDKR

>Cau_LOC113050690
MELSAVGERVFAAESIIKRRIRRGRMEYLVKWKGWSPKYSTWEPEENILDSRLFVAFEDGEREREIFGPKKRGPKLKT
FLLKAQAKEKSKSYEFRNDLSRGITYPSPEPVVTPRAREGLRAVVPTIFPPSTVNRGESVRISPPESREHRSPHSPRP
STDEFTLISKKRGRRPKLRFTDGYTSSLHPEYSKRAADEKTMASIPSKMAKLGLGEEEERCSEISGRIKISHKHIDGT
YSHKQNRVVPSRSTQHISSEWSLHSSRVSGDLQGHRNNPQTSHFHHKHLKYHSKRRTFEHADSTSKHPSLIAKIPVSR
IFGESDEGDAWRPSFSNVEKVIVTDVTSNFLTVTIKESNTDKGFFKDKR

>Sgr_LOC107571836
MEELSAVGEQVFDAECILNKRLRKGKLEYLVKWRGWSSKHNSWEPQENLLDPRLLAAFNKREQERELLISKKGKRPRG
RPRKILETIPVVSKSSSSSSSSSSSGSSSSSSTSSSSSDDDDDEDDNDRNPKLSPRPRELHPVPQKKAQIVVAKPEPP
KKKRGRKALPPELKAQRQAKGPRKILKPISRDSELRGSIKKPLMPASFTYTGLNRNSGRELMALQNRGYFTQKSSLSS
LGRSVGSASSPTALSRLPQSKTASDFKLSVSDMSSGGVDPNMPTCKSPGVAALNIHSSNGQTCPQLSPNVLKVSDQTL
LQRSGSLQKSPSSSFSSLKTPSSLQALNLQSINKTAQGNGTSVNDGSYLKGTSNPGRKSSVFNARHKHSPAPNAPSKF
PTNQQVLKSPQRDKSKADDLSERLGKKNQGRADKILVPTSEGRDQPVLDRSLSKDAGKPSKTLSELSTGEEGSSSDSD
HDSSFPSDNRDLAISVQAGQDWRPTRSLIEHVFVTDVTANLVTVTVKESPTSVGFFSIRNY

>Sgr_LOC107601561*
MGKNKELSKDVRDKIVDLHEAGIGYKAISKKLGGHTLDEKCCAVLILHGATKAAHGWASHPVCDLLSLPRTRSQCLCF
RHNSWEPHENLLDPRLLAAFNKREQERELLIRNKGKRPRGRPRKILETIPVVSKSSSSSSSSSSSGSSSSSSSSSFSSD
DDNDEDDNDRNPKPSPRPREHHPVPQKKAQIMVAKPEPPKKKRGRKALPPELKAQRQAKGPRKMLKPISRDSELRRSI
KKPLMPASFTYTGLNRNSGREPMALQNRGSFTQKNSLSSLGRSVGLASLPTKLSRLPQTKTASDFKLSVSDMSSGGVD
PKTPTCKSPGVAALNLHSSNGQTCPQLSPNVPKASDQTLLQRSGSLQKSPSSSFSSLKTPSSLQALNLQSVNKMTQGN
GTSTNDGSYLKGTANPSRKSSGFNASHEQSPALNVPSKFPTSQQVLKSPQQDKSKADDLSERLGKNNQGRVDKILTQK
TEGRDQPAQDRSSSKDAGKPSKTLSELSTGEEGSSSDSDHDSSFPSDNRDLAISVQAGQDWRPTRNLIEHVFVTDVTA
NLVTVTVKESPTSVGFFSIRNY

>Sgr_Cbx4 (Gene ID: 107591927)
MELPAAGEHVFAVESIEKKRIRKGRCEYLVKWRGWSPKYNTWEPEENILDPRLLVAFQNRERQEQLIGYRKRGPKPKH
LLLQVPSFARRSSVLSDLQEVSQGEDYQPKATSDLVQSQQYQLNSKKHHPYQPNPKDKQGEARINGKKKHYYQLNSKK
HHHYQPNPKMYDSLYQRVKETKVPELTDKEWNLSQALQQKWIQDKDSGCLSKMRNITMGLKKLPKLNGEADLNMNTNS
DAKEDKAPPNGKSSKLKIVKNKNKNGRIVIVMSKYMENGTKAAKIKEGTADSEEKRQALDCKSCGTDESTEKIKHTKK
QSLVKVIKKDSKAESACFSLSNGLSSCGGDNPKKACSIVFEPNNLKKPGNTGDYVSNEQPLQLTTKTSLTTVPFEKSD

QRGTQSTQYGPITFPQKRCYSEPDSDSREAKRFLSSQSISAPNTDLSHSCNGTTHFDDHQHSGGHDFEILDSNQDEPI
DLSCVRSREERKIPTHSQIGNSVPTGKAAVSTPEQVEKEIKKSVQSFTPFLGNIIITDVTANCLTVTFKEYVTV

>Sgr_LOC107553295
MELPAAGEHVFAVESIEKKRIRKGRCEYLVKWRGWSPKYNTWEPEENILDPRLLVAFQNRERQEQLMGYRKRGPKPKH
LLLQVPSFARRSSVLIDLQEVSQGEDNQSKTTSDLVQSQQYQLNSKKHHSYQLNPKDKQGEARINGEKKHYYQLNSKK
HHHYQPNPKMYDSLYQRVKETKGPELSDKEWNLSPALQQKWVQSKDSECLSKVRNITMGLKKLPKLNGGADLNMNTNS
DAKEDKAPPNGISSKLKIVKNKNKNGRIVIVMSKYMENGTQAAKIKKATADSEEKMQAQDCKSCGKDESTEKIKHTKK
QSLVKVIKKDSKAESACFRLSNGLSSCGGDNPKKTCSIVFEPNNSKKPGSTGDDVSNEQPLQLTNKTSLTTVPIEKSD
QKGTQSTQYGPLTFPRKRCYSEPDSDSGEAKRLLISQRTNAPNTDLSHSCNGTTQFNDHQHSGGHDFKMLDSNQDEPI
DLSCVRLREERKNPTHSQIGNSIPTGKAAVSAPEQVEKEMKKSVRSFTPFLGNIIITDVTANCLTVTFKEYVTV

>Sgr_LOC107562304
MDLPAVGEHVFAVEGIEKKRLRKGRIEYLVKWRGWSAKYNTWEPEENILDPRLLVAFQNRERQEQMVGYRKRGPKPKH
PLVQLPAFARRSSILGGLEDTSLDEENQPKVDPLQLHCPRPQHYQLNSKKHHQYQPSCREISVEQHVSGKKKHFYQLN
SKKHHHYQPDPKMYDTPLTGPKEVKVQDPSSKVWNLPPALQQKWIQNKDCLSKVKDLSIELKGLPDNANKSERALKTK
AKEFALPNGISSKMKIIKNKNKNGRIVIVMSKYMDKRVHSSKVKNKDVSEVETQHNEEPTLNKTDNLSDGETSEGLEN
GDTENTNTFSSSECVHENAIKKVELPKDMPTETEQTVMYICDQANSSPMEAEKAPPSHMDTKLNHRKRNLSEPNEDMR
NCKQFFSSRSISALNTVLSSPQREPIDLHYRGSLTNRAYSYDFSDTIPEEPIDLSCGPTKTLQLFPTAEKVSGSSAQV
EKTNNHFKPFVGNVIITDITTNCLTVTFKEYVQG

>Sgr_LOC107559247
MDLPAVGEHVFAVEGIEKKRLRKGRIEYLVKWRGWSAKYNTWEPEENILDPRLLVAFQNRERQEQMMGYHKRGPKSKH
PLVQLPAFARRSSILGGLEDTSLDEENQPKAEPLQIHRSWPQHYQLNSKKHHQYQPSCKEISVEQHVSGKKKHFYQLN
SKKHHHYQPDRKMYDTPLTGPKEVKVQDPSNKGWNLPPALQQKWIRNKDTGCLSKVKDLSIELKSLPDNANKAERALK
TSAKEFALPNGIGSKMKIIKNKNKNGRIVIVMSKYMDKGVHPSKVKSKDVSQVEAQHNEEATLNNTDNPSDGETSAGR
ENGSVENTSTFSSSSECIHKNSIKKAELPKDTPTETEQIVIDLCDQPNSEKAAPAHTDTGLNHRKRNLSEPNEDVRTC
KQFFSSRSISAPNTVLSSPQREPINLHYRGSLADRAYSYDFSDTIPEEPIDLSCGPTKTLKQFPTAEKVSGSSAPVEK
TNSHVKPFVGNVIITDITTNCLTVTFKEYVQG

>Sgr_LOC107602318
MELSAAGDRVFAAEAIMKRRVRKGRMEYLVKWKGWAIKYSTWEPEENILDERLVAAFEQKEREQEMYGPKKRGPKPKT
LLLKSRAQVTETSSRVPQFKHTRPQQHSKLPPPSAAPSYTPTTPSNAKLQSGAAQPKLKKDIHRCHRMARRPLPRPDQ
TVSPSGPFSSRPTVSPFSETVRILNRKVKPREVKKGRVILNLKVVDKAGNGGVANSRRTHVPSAQQSHFGRQKIPSQN
RVIGKNRRFGELSYRGIQTPIRSSGFPVFGKLFESHSLNNAENQTQSGESRNNTTNNLSSSQSSKVDASKGQTLDELP
QSNSSSEVSDGEPPSPPQTQSQHSSLQPKSSATKAPDPTLHKLSAQPVPSKNSLGLSALPSSPMFSSSSSASSSSEDS
ERILDLSVPHEMDRRLRRRHPFSGRHPLKVPEVPVSEEPSEEEEDLDWHPDMTSQCANVVVTDITANLLTVTIKEFCH
PPSATPPPCYPKNISAQHDTKQPKHHPNKT

>Sgr_LOC107585029
MELSAAGDRVFAAEAILKRRIRKGRMEYLVKWKGWAIKYSTWEPEENILDERLVAAFEQKEREQEMYGPKKRGPKPKT
LLLKSRAQVAETSSRVPEFKHTRPQQHSKLPPPSAAPSYTPTAPSNAKLQSGTAQPKLKKDIHRCHRMARRPLPRPDQ
TVGPSSPFSSRPTVSPFSETVRILNRKVKPREVKKGRVILNLKVVDKAGNGNGVANSRRTHVPSTQQSHFGRQKIPSR
NRVIGKNRRFGEVSYRGIQPTIRSSGFPVFGKLFDSHSLNNAENQTQSGESCNNTTNNLSSSQSSKVDVSKVQTLDEL
HPSNSSSEVSDGEPPSPPQTQSQHSSLPPKASATKAQDPALHKLSAQPVASKNSSGPSAVPSSPMFSSSSSASSSSED
NEHILDLSVPHEMDRRLRRRHPFSGRHPLKVPEVPVSEEPSEEEEDFDWHPDMTSQCANVVVTDITANLLTVTIKEFC
HPPSATPPPCYPKNISAQHDTKQPKQHPNKT

>Sgr_LOC107602676
MELSAVGERVFAAEAILKSRVRKGRIEYLLKWKGWALKHSTWEPEGNILDDRLITAFEQKERQQELYGPKKRGPKPKN
FVLKARAQAGDRPRSSNTQRTPPRTTAKPPASSSSASAAPPPPSSSSSYNTAPTPRVHSLAAAHKLKKDIHRCHRMSR
RPLPRPDPLGDPMGTTSLSRPPISPFSETVRILNRRVKPREVKRGRIVLNLKVIDKSQIPSRNRIIGKKHGDMPYRPF
QHPMKMLGFPMYGQPFGLNPCGSMSSMANEGSNTGVNRRGGGCCDSHSSANAQKFQYQPPPSPSSSSGSNNSSPSPQK
QVTQPEAPTKLDSAASSRSRDASQPHPKSSSVPFLPSSPSYSSSSLEGENQGSPNLVTSQGGKRKLRHRTQVGRASVC
QVSDRNTAPLPEENRVPKEGDPDWHPEMAPSSANVVVTDVTTNLLTVTIKEFCHSGAGSEPSSPCQADKPPCPTTVAS

>Sgr_LOC107576933*
MSYFPWLKWCRPTIHREREQELYGPKKRGPKPKNFVLKARAQAGDRPRSSNTRRTPPHTTAKPPASSSSASSSSSFST
APTPRVHSLAAAHKLKKDIHRCHRMSRRPLPRPDPLGDSTGSTSSSRPPISPFSETVRILNRRVKPREVKRGRIILNL
NVIDKSENSGVTSRRSPQSFAGRAKIPSRNRIIGKKQGDMPYRPFQHPMKMLGFPMYGQPFGLHPCGSMSSMANEESN
TGANRRGGNCCDSHSSANAQKFQYQPPPSPSSFSGSNNSSPSLQKQPTQPEAPTSPTKLDSAASSRSRDASQPPPKSS
SVPFLPSSLEDEDQGSPNLATSRGRKRKLRHRTQVGQASVCQVSDRTTTPHPEENREPKEGDPDWNPEMAPSCTNVVV
TDVTTNLLTVTIKEFCHSGAGSEPSSPCQADNPPCPTTAAS

>Sgr_LOC107584093
MELSAIGEQVFAVESILKKRVRKGHVEYLLKWKGWPPKYSTWEPEEHILDPRLVLTYEEKEQRERSVRWRKRGPKPKR
LFVQPHIALLKRNIYAMDLRSAHKDTEKSSVHLPLSLDPRFQSTGACIYRQLTHHKKKKKASRETSEEEWDRREDDDD

DDDIFMEEEEEEEEEEDTRQGGTKTGSNTLNKRVRRGRWSPTTGSEAMTISPLPEDWSPVMGPEEVIVTDITINSLTV
TFREALVARGFFRSWEMEI

>Sgr_LOC107554058
MELSAIGEQVFAVESIIKKRVRKGHVEYLLKWKGWPPKYSTWEPEEHILDPRLVLAYEEKEERERSVRWRKRGPKPKR
LFMQRNIYAMDLRSAHKATENSSAHLPLSLDPRFQSTGACIYQRLTHHKKKKKTSRETSEEEWDRREEEDHEDDDDEG
VMEEEEEDTRQGGTKTGSNTLNKHVRRGRWIPTTESAAMTISPLPEDWSPVMGPEEVIVTDITINSLTVTFREALVAR
GFFRSWEMEI

>Sgr_LOC107602332
MNMELSSIGEQVFAVESITKKRIRKGNVEYLLKWQGWAPKYSTWEPEDNILDPRLVLAYEEKEEKERALAYKRKGLRP
RRVILRSIYPMDLRSKHKVPDKPTPRIRLSLTRSMGTEIDQNGRRYRPIEKYKNRHCRSKLMNDIKPSQYSLPAKDSE
KEWDEDDEDEQENKVKKMRENEEKTTEVHQDIPSSQETTEGYSSSAEDEAVITIKETENCSSIYDHAEKPSEDTSQVL
GATENSTITNTQENEPVTNTTGGEDSVRVSHDTTNTGQSLHSTESGAKQVVFDQVQNRPSVIEVHSSTKCRQEEVRES
GEVDHSEAKEKEGMDAEITAERTLLQVPIDQSQTPSTTTVHPGKVTVTQVTINSLTVTFKEAMSAEGFFSGDGLEV

>Sgr_LOC107578989
MELSAVGERVFAAESIIKRRIRRGRMEYLVKWKGWSQKYSTWEPEENILDERLFAAFEEREREREMYGPKKRGPKPET
FLMKVKAKAKGKNCEFRREMSRDLHVSFPVAEPVVTPRAREGLRTVVPTIFPPSTINRGESVRVHPPVSERDPLMHGM
LVQRPLDFASSPKKRGPKPKLRPGGSSTEGVKRKADEPLSYRPSKTERSGETSNCDVVRLTQKFQAESSHVKKQMGSR
SSDVKFTHGGNILKAGLGVLGHRRKDSPSGAIKQSKMKHPPKSNLFRSTDQTREQLSLSFVDETDQSWLPCLKNMEKI
VVTDVTSNSLTVTIKESSTDKGFFKENR

>Sgr_LOC107559248
MELSAVGERVFAAESIIKRRIRRGRMEYLVKWKGWSQKYSTWEPEENILDERLFAAFEEREREREMYGPKKRGPKPET
FLMKAKAKAKGKNYEFRREMSRDLHVSFPVAEPVVTPRAREGLRTVVPTIFPPSTINRGESVRVRPPDLERDPLAHGI
QRPLDFASSPKKRGPKPKLRPGGSSTEGVRRKADEPLSYRPSKTERSGETSNCDGIHLTQKFQAESSHVQKQMGSRSS
DVKFTHGGTILKAGLGVLGHRRKDSSSGAIKQSKMKHPPRNHLFRSTDQAREQLSLSFVEETDQSWLPCLKNMEKIVV
TDVTSNSLTVTIKESSTDKGFFKENR

>Sgr_LOC107601979
MELSAVGERVFAAESIIKRRIRRGRMEYLVKWKGWSPKYSTWEPEENILDSRLFVAFEEREREREIFGPKKRGPKLKT
FLLKAQTKEKAKSYEFRNESFRGITYPSPEPVVTPRAREGLRAVVPSIFPPSTVNRGESVRIPPPEPREHRSPHSPRP
STDAFTLAPKKRGRKPKLRFTDGYTSSLNPEHLKRAADETMASIPSKMAKLGLGEEEERCSEISGRIKISHKHMDATY
SHKQNRVVPSRSTQHISPEWSLHSSRVSADLQGCRNNPQTSHFHHKHLKYHSKRRIFEHADSTSRHPSLIAKIPVSRI
FGEPEEGDAWRPSFSNVEKVIVTDVTSNFLTVTIKESSTDKGFFKDKR

>Sgr_LOC107591932
MELSAVGERVFAAESIIKRRIRRGRMEYLVKWKGWSPKYSTWEPEENILDSRLFVAFEEREREREIFGPKKRGPKLKT
FLLKAQTKEKAKSYEFRNESFRGITYPSPEPVVTPRAREGLRAVVPSIFPPSTVNRGESVRIPPPEPREHRSPHSPRP
STDAFTLAPKKRGRKPKLRFTDGYTSSLNPEHLKRAADETMASIPSKMAKLGLGEEEERCSEISGRIKISHKHMDATY
SHKQNRVVPSRSTQHISPEWSLHSSRVSADLQGCRNNPQTSHFHHKHLKYHSKRRIFEHADSTSRHPSLIAKIPVSRI
FGEPEEGDAWRPSFSNVEKVIVTDVTSNFLTVTIKESSTDKGFFKDKR

>Sgr_LOC107553294
MELSAVGERVFAAESIIKRRIRRGRMGYLVKWKGWSPKYSTWEPEENILDSRLFVAFEEREREREIFGPKKRGPKLKT
FLLKAQAKEKAKSYGFRNESPRGIEYPSPEPLVTPRAREGLRSVVPTIFPPSTVNRGESVRIPPPEPREHRSPHSPRP
STDAFTLTPKKRGRKPKLCFTDGYTSSLHPEHSKRAADETMASIPSKMAKLGLGEEEERCSEISGRIKISHKHMDATY
SHKQNRVVPSRSTQHISPEWSLHSSRVSADLQGHRTNPQTSHFHHKHLKHHSKRRTFEHGDSTSRQPSLIAKIPVSRI
FGEPEEGDAWRPTFCNVEKVIVTDVTSNFLTVTIKESSTDKGFFKDKR

>Sgr_LOC107572510*
MLLDSRRRGGRLQYLVDWEGYGPEERSWIPASNILDPSLIEEFHRARPDRPAPRPRGRPRRAPGVAPRGGGPVTPSQQ
REPSPEY

>Srh_LOC107718939
MRRVMEELSAVGEQVFDAECILNKRLRKGKLEYLVKWRGWSSKHNSWEPQENLLDPRLLAAFNKREQERELLISKKGK
RPRGRPRKILETIPVVSKSSSSSSSSSSSGSSSSSSSSSSSSDDDDDEDDNDRNPKPSPRPREHHPVPQKKAQIVVAK
PEPPKKKRGRKALPPELKAQHQAKGPRKILKPISRDSELRRSIKKPLMPASFTYNGLNRNSGRELMALQNRGYFTQKS
SLSSLGRSIGSASSPTALSRPPQSKTASDFKLSVSDMSSGGVDPNAPTCKSPGVAALNIHSSNGQTCPQLSPNVLKVS
DQTLLQRSGSLQKSPSSSFSSLKTPSSLQALNLQSINKTAQGNGTSVNDGSYLKGTSNPGRKSSVFNARHEHSPAPNT
PSKFPTNQQVLKSPQRDKSKADDLSERLGKKTQGREDKILVPTSEGRDQPVPDRSSSKDAGKPSKTLSELSTGEEGSS
SDSDHDSSFPSDNRDLAISVQAGQDWRPTRSLIEHVFVTDVTANLVTVTVKESPTSVGFFSIRNY

>Srh_LOC107710978
MRTVMEELSAVGEQVFDAECILNKRLRKGKLEYLVKWRGWSSKHNSWEPQENLLDPRLLAAFNKREQERELLIHNKGK
RPRGRPRKILETIPVVSKSSSSSSSSSSSGSSSSSSSSFSSDDDNDEDDNDRNPKPSPRPREHHPVPQKKAQIVVAKP
EPPKKKRGRKALPPELKAQRQAKGPRKMLKPISRDSELQGSIKKPLMPASFTYTGLNRNSGREPMALQNRGSFTQKNS

LSSLGRSVGLASLPATLSRLPQTKTASDFKLSVSDMSSGGVDPKTPTCKSPGVAALNLHSSNGQTCPQLSPNAPKASD
QTLLQRSGSLQKSPSSSFSSLKTPSSLQALNLQSVNKMTQGNGTSTNDGSYLKGTANPSRKSSGFNARHEQSPEPNVP
SKFPTSQQVLKSPQRDKSKADDLSERLGKNNQGRVDKILTQTTEGRDHPTQDRSSSKDAGKPSKTLSELSTGEEGSSS
DSDHDSSFPSDNRDLAISVQAGQDWRPTRNLIEHVFVTDVTANLVTVTVKESPTSVGFFSIRNY

>Srh_Cbx4 (Gene ID: 107710281)
MELPTAGEHVFAVESIEKKRIRKGRCEYLVKWRGWSPKYNTWEPEENILDPRLLVAFQNRERQEQLIGYRKRGPKPKH
LLLQVPSFARRSSVLSDLQEVSQVEDYQPKATSDLVQSQQYQLNSKKHHPYQPNPKDKQGEARINGKKKHYYQLNSKK
HHHYQPNPKMYDSLYQRVKETKVPELTDKEWNLSPALQQKWIQDKDSGCLSKVRNINMGLKKLPKLNGEADLNMNTNS
DAKEDKAPPNGKSSKLKIVKNKNKNGRIVIVMSKYMENGTQAAKIKEGTPDSEEKRQALDCKSCGTDESTEKIKHTKK
QSLVKVIKKDSKAESACFRLSNGLSSCGGDNPKKACSIVFEPNNLKKPGNTGDYVSNEQPLQLTTKTSLTTVPFEKSD
QRGTQSTQYGPITFPQKRCYSEPDSDSREAKRFLSSQSISAPNTDLSHSCNGTTHFDDHQHNGGHDFEILDSNQDEPI
DLSCVRSREERKIPTHSQIGNSIPTVKAAVSTPEQVEKEMKKSVRSFTPFLGNIIITDVTANCLTVTFKEYVTV

>Srh_LOC107758118
MDLPAVGEHVFAVEGIEKKRLRKGRIEYLVKWRGWSAKYNTWEPEENILDPRLLVAFQNRERQEQMMGYRKRGPKSKH
PLVQLPAFARRSSILGGLEDTSLDEENQPKAEPLQIHRSWPQHYQLNSKKHHQYQPSCKEISVEQHVSGKKKHFYQLN
SKKHHHYQPDPKMYDTPLTGPKEVKVQDPSNKGWNLPPALQQKWIRNKDTGCLSKVKDLSIELKSLPDNANKAERALK
TSAKEFALPNGIGSKMKIIKNKNKNGRIVIVMSKYMDKGVHSSKVKNKDVSQVEAQHNEESTLNNTDNPSDGETSAGR
ENGSAENTSTFSSSSECIHKNSIKKAELPKDTPTETEQIVIDLCDQPNSEKAAPAHTDTGLNHRKRNLSEPNEDVRTC
KQFFSSRSISAPNTVLSSPQREPINLHYRGSLADRAYSYDFSDTIPEEPIDLSCGPTKTLKQFPTAEKVSGSSAPVEK
TNSHVKPFVGNVIITDITTNCLTVTFKEYVQG

>Srh_LOC107736694*
VMSKYMDKGVHSSKVKNKDVSEVETQHNEEPTLNKTDNLSDGETSEGLENGAVENTNTFSSSECVHENAIKKVELPKD
TPTETEQTVMYICDQPNSSPMEAEKAPPSHMDTKLNHRKRNLSEPNEDVRNCKQFFSSRSISAPNTVLSSPQREPIDL
HYRGSLTNRAYSYDFSDTIPEEPIDLSCGPTKTLQQFPTAEKVSGSLAQAEKTNNHFKPFVGNVIITDITTNCLTVTF
KEYVQG

>Srh_LOC107728779*
MDLPAVGEHVFAVEGIEKKRLRKGRIEYLVKWRGWSAKYNTWEPEENILDPRLLVAFQNRERQEQMVGYRKRGPKPKH
PLVQLPAFARRSSILGGLEDTSLDEENQPKVDPLQLHCPRPQHYQLNSKKHHQYQPSCREISVEQHVSVKKKHFYQLN
SKKHHHYQPDPKMYDTPLTGPKEVKVQDPSSKVWNLPPALQQKWIQNKDTACLSKVKDLSIEHKGLPDNANKSERALK
TRAKEFAFPNGISSKMKIIKNKNKNGRIVIVMSKYMDKGVHSSKVKNKDVSEVETQHNEEPTLNKTDNLSDGETSEGL
ENGAVENTNTKGTRGKHS

>Srh_LOC107710010
MELPTAGEHVFAVESIEKKRIRKGRCEYLVKWRGWSPKYNTWEPEENILDPRLLVAFQNRERQEQLMGYRKRGPKPKH
LLLQVPSFARRSSVLTDLQEVSQGEDNQSKTTSDLVQSQQYQLNSKKHHSYQLNPKDKQGEARINGGKKHYYQLNSKK
HHHYQPNPKMYDSLYQRVKETKVPELADKEWNLSPALQQKWVQSKDSECLSKVRNITMELKKLPKLNGGTDLNMNTNS
DAKEDKAPPNGISSKLKIVKNKNKNGRIVIVMSKYMENGTQAAKIKKGTADSEEKRQAQDCKSCGTDESTEKIKHTKK
QSLVKVIKKDSKAESACFRLSNGLSSCGGDNPKKTCSIVFEPNNSKKPGSTGDYVSNEQPLQLTNKTSLTTVPIEKSD
QKGTQSTQYGPLTLPRKRCYSEPDCDSGEAKRLLISQSINAPNTDLSHSCNGTTHFNDHQHSGGHDFEILDSNQDEPI
DLSCVRLREERKNPTHSQIGNSIPTGKAAVSAPEQVEKEMKKSVRSFTPFLGNIIITDVTANCLTVTFKEYVTV

>Srh_LOC107720374
MEVSAVGEQVFPAEAILKSRVRKGRIEYLVKWKGWALKHSTWEPEENILDDRLITAFEQKEREQELYGPKKRGPKPKN
FVLKVRAQAGDRPRSSNTRRTPPRTTAKPPASSSSASSSSSFSTAPTPRVHSLAAAHKLKKDIHRCHRMSRRPLPRPD
PLGDPTGSTSSSRPPISPFSETVRILNRKVKPREVKRGRIILNLNVIDKSENSGVTSRRSPQSFAGRTKIPSRNRIIG
KKQGDMPYRPFQHPMKMLGFPMYGQPFGLHPCGSMSSMANEESNSGANRRGGNRGKSCDSHSSANAQKFQYQPPPSPS
SFSESNNSSPSLQKQPTQPEAPTSPTKLDSAASSRSRDASQPPPKSSSAPFLLSSPSYSSSLSSSLEDEDQGSPNLAT
SRGRKRKLRHRTQVGQASVCQVSDRTTTPHPEENREPKEGDPDWNPEMAPSCTNVVVTDVTTNLLTVTIKEFCHSGAG
SEPSSPCQADNPPCPTTAAS

>Srh_LOC107717667
MELSAVGERVFAAEAILKSRVRKGRIEYLLKWKGWALKHSTWEPEGNILDDRLITAFEKKEREQELYGPKKRGPKPKN
FVLKARAQAGDKPRSSNTQRTPPRTTAKPPASSSSASAAPPQPSSSSSYNTAPTPRVHSLAAAHKLKKDIHRCHRMSR
RPLPRPDPLGDPTGTTSSSRPPISPFSETVRILNRRVKPREVKRGRIILNLKVIDKSQIPSRNRIIGKKHGDMPYRPF
QHPMKMLGLPMYGQPFGLNPCGSMSSMANEGSNTGVNRRGGGCCDSHSSANAQKFQYQPPPSPSSSSGSNNSSPSPQK
QVTQPEAPTKLDSAASSRSRDASQPHPKSSSAPFLPSSPSYSSSSLEGENQGSPNLVTSRGGKRKLRHRTQVGRASVC
QVSDRNTAPLPEENRVPKEGDPDWHPEMAPSCANVVVTDVTTNLLTVTIKEFCHSGAGSEPSSPCQADKPPCPTTAAS

>Srh_LOC107710048
MELSAAGDRVFAAEAIMKRRIRKGRMEYLVKWKGWAIKYSTWEPEENILDERLVAAFEQKEREQEMYGPKKRGPKPKT
LLLKSRAQVAETSSRVPELKHTRPQQHSKLPPPSAAPSYTPTAPSNAKLQSGAAQPKLKKDIHRCHRMARRPLPRPDQ
TVSPSGPFSSRPTVSPFSETVRILNRKVKPREVKKGRVILNLKVVDKAGNGGVANSRRTHVPSAQQSHFGRQKIPSRN
RVIGKNRRFGELSYRGIQTPIRSSGFPVFGKLFDSHSLNNAENQTQSGESRNNTTNNLSSSQSSKVDASKGQTLDELP
PSNSSSEVSDGEPPSPPQTQSQHSSLQPKSSATKAPDPTLHKLSAQPVPSKNSLGLSALPSSPMFSSSSSASSSSEDS

ERILDLSVPHEMDRRLRRRHPFSGRHPLKVPEVPVSEEPSEEEEDVDWHPDMTSQCANVVVTDITANLLTVTIKEFCH
PPSATPPPCYPKNISAQHDTKQPKHHPKKT

>Srh_LOC107740604
MELSAAGDRVFAAEAILKRRIRKGRMEYLVKWKGWAIKYSTWEPEENILDERLVAAFEQKEREQEMYGPKKRGPKPKT
LLLKSRAQVAETSSRVPEFKHTRPQQHSKLPPPSAAPSYTPTAPSNAKLQSGTAQPKLKKDIHRCHRMARRPLPRPDQ
TVGPSSPFSSRPTVSPFSETVRILNRKVKPREVKKGRVILNLKVVDKAGNGGVANSRRTHVPSTQQSHFGRQKIPSRN
RVIGKNRRFGEVSYRGIQPTIRSSGFPVFGNLFDSHSLSNAENQTQSGESRNNTTNNLSSSQSSKMDVSKGQTLDELP
PSNSSSEVSDGEPPSPPQTQSQHSSLPPKASATKAQDPALHKLSAQPVASKNSSGPSALPSSPMFSSSSSASSSSEDN
EHILDLSVPHEMDRRLRRRHPFSGRHPLKVPEVPVSEEPSEEEEDLDWHPDMTSQCANVVVTDITANLLTVTIKEFCH
PPSATPPPCYPKNISAQHDTKQPKQHPNKT

>Srh_LOC107734182
MELSAIGEQVFAVESIIKKRVRKGHVEYLLKWKGWPPKYSTWEPEEHILDPRLVLTYEEKEQRERSVRWRKRGPKPKR
LFMQRNIYAMDLRSAHKDTEKSSTHLPLSLDPRFQSTGACIYRQLTHHKKKKKASRETSEEEWDRREDDDDDDDEGVM
EEEEEEEEEDTRQGGTKTGSNTLDKRVRRGRWSPTTGSEAMTISPLPEDWSPVMGPEEVIVTDITINSLTVTFREALV
ARGFFRSWEMEI

>Srh_LOC107709653
MELSAIGEQVFAVESIIKKRVRKGHVEYLLKWKGWPPKYSTWEPEEHILDPRLVLTYEEKEERERSVRWRKRGPKPKR
LFMQRSIYAMDLRSAHKDTEKSSAHLPLSLDPRFQSTGACIYQRLTHHKKKKKTSRETSEEEWDRREEEDDDEGVMEE
EEEEEEDTRRGETKTGSNTLNKHVRRGHWSPTTESEAMTISPLPEDWSPVMGPEEVIVTDITINSLTVTFREALVARG
FFRSWEMEI

>Srh_LOC107748788
MNMELSSIGEQVFAVESITKKRIRKGNVEYLLKWQGWPPKYSTWEPEDNILDPRLVLAYEEKEEKERALAYKRKGLRP
RQVILRNLYPMDLRSKHKVLGKSTHRIRLPLTRSMGTEIDQNGRRCQRIEKRKNRPCRSKLMNDIKPFQQPRRHPLLP
KDSEKEWDGDEEDEQKKKAKKMRTNEENTTEVHQDIPSGQEMSEGYNSSAEHEAVITIKETENCSSIFDHAEKPREDT
GPVLGATVNSTITNTQENEPVTNTAGDEDSVRVSHDTTNTDQSLHNSTKSGAEQGVFDKVPDRPSVIEVHSSAKCRQD
EVSERGELDRSETKEKEGMDAVVTAECTTTLQVPIDQLQTPTTTAVHPGKVIVTQVTINTLTVTFKEAMSAEGFFSGY
GL

>Srh_LOC107723608
MELSSIGEQVFAVESITKKRIRKGNVEYLLKWQGWAPKYSTWEPEDNILDPRLVLAYEEKEEKDRALAYKRKGLRPRQ
VILRSIYPMDLRSKHKVPDKPTPRIRLSLTRSMGTEIDQNGRRYRPIEKYKNRHCRSKLVNDIKPSQYSLPAKDSEKE
WDEDDEDEQENKVKKMRENEEKTTEVHQDIPSSQETTEGYSSSAEDEAVIIIKESENCSSIYDHAEKPSEDTSQVLGA
TENSTITNTQENEPVTNTTGGEDSVRVSHDTTNTDQSLHSTESGAKQVVFDQVQNRPSVIEVHSSTKCRPEEVRESGE
VDHSEAKEKEGMDAEITAERTLLQVPIDQSQTPSTTTVHPGKVTVTQVTINSLTVTFKEAMSAEGFFSGDGLEV

>Srh_LOC107758117
MELSAVGERVFAAESIIKRRIRRGRMEYLVKWKGWSQKYSTWEPEENILDERLFAAFEEREREREMYGPKKRGPKPET
FLMKAKAKAKGKNYEFRREMSRDLHVSFPVAEPVVTPRAREGLRTVVPTIFPPSTINRGESVRVRPPDLERDPLTHGT
PVQRPLDFASSPKKRGPKPKLRPGGSSAEGVKRKADEPLSYRPSKTERSGETSSCDVIHLTQKIQAESSHVQKQMGSR
SSDVKFTHGGTILKSGLGVLGHRRKDSSSGAINQSKMKHPPKNHLFRSTDQAREQLSLSFVDETDQSWLPCLKNMEKI
IVTDVTSNSLTVTIKESSTDKGFFKENR

>srh_LOC107728583
MELSAVGERVFAAESIIKRRIRRGRMEYLVKWKGWSQKYSTWEPEENILDERLFAAFEEREREREMYGPKKRGPKPET
FLMKAKAKAKGKNCEFRREISRDLHVSFPVAEPVVTPRAREGLRTVVPTIFPPSTINRGESVRVRPPVSERDPLMHGM
LVQRPLDFASSPKKRGPKPKLRPGGSSTEGVKRKADEPLSYRPSKTERSGETSNCDVIHLTQKFQAESSHVKKQMGSR
SSDVKFTHGGNILKAGLGVLGHRRKDSPSGAIKQSKMKHPPKSNLFRSTDQTREQLSLSFVDETDQSWLPCLKNMEKI
VVTDVTSNSLTVTIKESSTDKGFFKENR

>Srh_LOC107710282
MELSAVGERVFAAESIIKRRIRRGRMEYLVKWKGWSPKYSTWEPEENILDSRLFVAFEEREREREIFGPKKRGPKLKT
FLLKAQTKEKAKSYEFRNESFRGITYPSPEPVVTPRAREGLRAVVPSIFPPSTVNRGESVRIPPPEPREHRSPHSPRP
STDAFTLAPKKRGRKPKLRFTDGYTSSLHPEHLKRAADETMASIPSKMAKLGLGEEEERCSEISGRIKISHKHMDATY
SHKQNRVVPSRSTQHISPEWSLHSSRVSADLQGRRNNPQTSHFHHKHLKYHSKRRTFEHADSTSRHPSLIAKIPVSRI
FGEPEEGDAWRPSFSNVEKVIVTDVTSNFLTVTIKESSTDKGFFKDKR

>Srh_LOC107710028
MELSAVGERVFAAESIIKRRIRRGRMEYLVKWKGWSPKYSTWEPEENILDSRLFVAFEEREREREIFGPKKRGPKLKT
FLLKAQAKEKAKSYGFRNESSRGIAYPSPELLVTPRAREGLRSVVPTIFPPSTVNRGESVRIPPPEPREHRSPHSPRP
STDAFTLTPKKRGRKPKLHFTDGYTSSLRPEHSKRAADETMASIPSKMAKLGLGEEEERCSEISGRIKISHKHMDATY
SHKQNRVVPSRSTQHISPEWSLHSSRVSADLQGHRTNPQTSHFHLKHLKHHSKRRTFEHGDSTSRQPSLIAKIPVSRI
FGEPEEGDAWRPTFCNVEKVIVTDVTSNFLTVTIKESSTDKGFFKDKR

>Srh_LOC107740650* = LOC107740604 (?)
MELSAAGDRVFAAEAILKRRIRKGRMEYLVKWKGWAIKYSTWEPEENILDERLVAAFEQKEREQEMYGPKKRGPKPKT
LLLKVS

>Srh_LOC107738665*
MLLDSRRRGGQLQYLVDWEGYGPEERSWVNAPDILDPSLIEEFHRARPDRPAPRPRGRPRRAPGVAPRGGGSVMPGQQ
REPSPEY

>San_LOC107696361
MRRVMEELSAVGEQVFDAECILNKRLRKGKLEYLVKWRGWSSKHNSWEPQENLLDPRLLAAFNKREQERELLISKKGK
RPRGRPRKILETIPVVSKSSSSSSSSSSSGSSSSSSSSSSSSDDDDDEDDNDRNPKPSPRPREHHPVPQKKAQIVVAK
PEPPKKKRGRKALPPELKAQRQAKGPRKILKPISRDSELQGSIKKPLMPASFTYTGLNRNSGRELMALQNRGYFTQKS
SLSSLGRSVGSASSPTALSRLPQSKTASDFKLSVSDMSSGGVDPNMPTCKSPGVAALNIHSSNGQTCPQLSPNVLKVS
DQTLLQRSGSLQKSPSSSFSSLKTPSSLQALNLQSINKTAQGNGTSVNDGSYLKGTSNPGRKSSVFNARHEHSPAPNA
PSKFPTNQQVLKSPQRDKSKADDLSERLGKKNQGRADKILVPTSEGRDQPVPDRSSSKDAGKPSKTLSELSTGEEGSS
SDSDHDSSFPSDNRDLAISVQAGQDWRPTRSLIEHVFVTDVTANLVTVTVKESPTSVGFFSIRNY

>San_LOC107695690
MRTVMEELSAVGEQVFDAECILNKRLRKGKLEYMVKWRGWSSKHNSWEPQENLLDPRLLAAFNKREQERELLIRNKGK
RPRGRPRKILETIPVVSKSSSSSSSSLSSGSSSSSSSFSSDDDNDEDDNDRNPKPSPRPREHHPVPQKKAQIVVAKP
EPPKKKRGRKALPPELKAQRQAKGPRKMLKPISRDSELRGSIKKPLMPASFTYTGLNRNSGREPMALQNRGSFTQKNS
LSSLGRSVGLASLPTKLSRLPQTKTASDFKLSVSDMSSGGVDPKTPTCKSPGVAALNLHSSNGQTCPQLSPNVPKASD
QTLLQRSESLQKSPSSSFSSLKTPSSLQALNLQSINKMTQGNGTSTNDGSYLKGTANPSRKSSGFNARHEQSPAPNVP
SKFPTSQQVLKSPQRDKSKADDLSERLGKNNQGRVDKILTQTTEGRDQPAQDRSSSKDAGKPSKTLSELSTGEEGSSS
DSDHDSSFPSDNRDLAISVQAGQDWRPTRNLIEHVFVTDVTANLVTVTVKESPTSVGFFSIRNY

>San_Cbx4 (Gene ID: 107701204)
MELPAAGEHVFAVESIEKKRIRKGRCEYLVKWRGWSPKYNTWEPEENILDPRLLVAFQNRERQEQLIGYRKRGPKPKH
LLLQVPSFARRSSVLSDLQEVSQGEDYQPKATSDLVQSQQYQLNSKKHHPYQPNPKDKQGEARINGKKKHYYQLNSKK
HHHYQPNPKMYDSLYQRVKETKVPELTDKEWNLSPALQQKWIQDKDSGCLSKVRNITMGLKKLPKLNGEADLNMNTNS
DAKEDKAPPNGKSSKLKIVKNKNKNGRIVIVMSKYMENGTQAAKIKEGTADSEEKRQALDCKSCGTDESTEKIKHTKK
QSLVKVIKKDSKAESACFSLSNGLSSCGGDNPKKACSIVFEPNNLKKPGNTGDYVSNEQPLQLTTKTSLTTVPFEKSD
QRGTQSTQYGPITFPQKRCYSEPDSDSREAKRFLSSQSISAPNTDLSHSCNGTTHFDDHQHSDGHNFEILDSNQDEPI
DLSCVRSREERKIPTHSQIGNSIPTGKAAVSTPEQVEKEMKKSVRSFTPFLGNIIITDVTANCLTVTFKEYVTV

>San_LOC107669529
MELPAAGEHVFAVESIEKKRIRKGRCEYLVKWRGWSPKYNTWEPEENILDPRLLVAFQNRERQAQLMGYRKRGPKPKH
LLLQVPSFARRSSVLTDLQEVSQGEDNQSKTTSDLVQSQQYQLNSKKHHSYQLNPKDKQGRARINGEKKHYYQLNSKK
HHHYQPNPKMYDSLYQRVKETKVPELADKEWNFSPALQQKWVQSKDSECLSKVRNITMELKKLPKVNGGADLNMNTNS
DAKEDKAPPNGISRKLKIVKNKNKNGRIVIVMSKYMENGTQAAKIKKGTADSEEKRQAQDCKSCGTDESTEKIKHTKK
QSLLKVIKKDSKAESACFRLSNGLSSCGGNNPKKTCSIVFEPNNSKKPGSTGDYVSNEQPLQLTNKTSLTTVPIEKSD
QKGTQSTQYGPLTLPRKRCYSEPDSDSGEAKRLLISQSTNAPNTDLSHSCNGTTHFNDHQHSGGHDFEILDSNQDEPI
DLSCVRLREEKKNPTHSQIGNSIPTGKAAVSAPEQVEKEMKKSVQSFTPFLGNIIITDVTANCLTVTFKEYVTV
>San_LOC107690020
MDLPAVGEHVFAVEGIEKKRLRKGRIEYLVKWRGWSAKYNTWEPEENILDPRLLVAFQNRERQEQMMGYRKRGPKSKH
PLVQLPAFARRSSILGGLEDTSLDEENQPKAEPLQIHRSWPQHYQLNSKKHHQYQPSCKEISVEQHVSGKKKHFYQLN
SKKHHHYQPDPKMYDTPLTGPKEVKVQDPSNKGWNLPPALQQKWIRNKDTGCLSKVKDLSIELKSLPDNANKAERALK
TSAKEFALPNGIGSKMKIIKNKNKNGRIVIVMSKYMDKGVHSSKVKNKDVSQVEAQHNEESTLNNTDNPSDGETSAGR
ENGSVENTSTFSSSSECIHKNSIKKAELPKDTPTETEQIVIDLCDQPNSEKAAPAHTDTGLNHRKRNLSEPNEDVRTC
KQFLKQFSAPNTVLSSPQREPINLHYRGSLANRAYSYDFSDTIPEEPIDLSCGPTKTLKQFPTAEKVSGSSAPVEKTN
SHVKPFVGNVIITDITTNCLTVTFKEYVQG

>San_LOC107670662
MDLPAVGEHVFAVEGIEKKRLRKGRIEYLVKWRGWSAKYNTWEPEENILDPRLLVAFQNRERQEQMVGYRKRGPKPKH
PLVQLPAFARRSSILGGLEDTSLDEENQPKVDPLQLHCPRPQHYQLNSKKHHQYQPSCREISVEQHVRGKKKHFYQLN
SKKHHHYQPDPKMYDTPLTGPKEVKVQDPSSKVWNLPPALQQKWIQNKDTACLSKVKDLSIELKGLPDNANKSERALK
TRAKEFALPNGISSKMKIIKNKNKNGRIVIVMSKYMDKGVHSSKVKNKDVSEVETQHNEEPTLNKTDNLSDGETSEGL
ENGAVENTNTFSSSECVHENSIKKVELPKDTPTETEQTVMYICDQPNSSPMETEKAPPSHMDTKLNHCKRNLSEPNED
VRNCKQFFSSRSISAPNTVLSSLQREPIDLHYRGSLTNRAYSYDFSDTIPEEPIDLSCGPTKTLQQFPTVEKVSGSSA
QVEKTSNHFKPFVGNVIITDITTNCLTVTFKEYVQG

>San_LOC107667890
MEVSTVGEQVFPAEAILKSRVRKGQIEYLVKWKGWALKHSTWEPEENILDDRLIMAFEQKXRQQELYGPKKRGPKPKN
FVLKARAQAGDRPRSSNTRRTPPRTTAKPPASSSSASSSSSFSTAPTPRVHSLAAAHKLKKDIHRCHRMSRRPLPRPD
PLGDPTGSTSSSRPPISPFSETVRILNRRVKPREVKRGRIILNLNVIDKSENSGVTSRRSPQSFAGRAKIPSRNRIIG
KKQGDMPYRPFQHPMKMLGFPMYGQPFGLHPCGSMSSVANEESNTGANQRGGNCCDSHSSANAQKFQYQPPPSPSSFS
GSNNSSPSLQKQPTQPEAPTSPTKLDSAASSRSRDASQPPPKSSSAPFLPSSPSYSSSLSSSLEDEDQGSPNLATSRG

RKRKLRHRTQVGRASVCQVSDRTTTPHPEENREPKEGDPDWNPEMAPSCTNVVVTDVTTNLLTVTIKEFCHSGAGSEP
SSPCLADNPPCPTTAAS

>San_LOC107704552
MELSAVGERVFAAEAILKSRVRKGRIEYLLKWKGWALKHSTWEPEGNILDDRLITAFEQKERQQELYGPKKRGPKPKN
FVLKARAQAGDRPRSSNTQRTPPRTTAKPPASSSSASAAPPPPSSSSSYNTAPTPRVHSLAAAHKLKKDIHRCHRMSR
RPLPRPDPLGDPTGTTSSSRPPISPFSETVRILNRRVKPREVKRGRIILNLKVIDKSQIPSRNRIIGKKHGDMPYRPF
QHPMKMLGFPMYGQPFGLNPCGSMSSMANEGSNTGVNRRGGGCCDSHSSANARKFQYQPPPSPSSSSGSNNSSPSPQE
QVTQPEAPAKLDSAASSRSQDASQPHPKSSSAPFLPSSPSYSSSSLEGENQGSPNLVTSRGGKRKLRHRTQVGRASVC
QVSDRNTAPLPEENRVPKEGDPDWHPEMAPSCANVVVTDVTTNLLTVTIKEFCHSGAGSEPSSPCQADKPPRPTTAAS

>San_LOC107690738
MELSAAGDRVFAAEAIMKRRVRKGRMEYLVKWKGWAIKYSTWEPEENILDERLVAAFEQKEREQEMYGPKKRGPKPKK
LLLKSRAQVAETSSRVPEFKHTRPQQHSKLPPPSAAPSYTPAAPSNAKLQSGAAQPKLKKDIHRCHRMARRPLPRPDQ
TVSPSGPFSSRPTVSPFSETVRILNRKVKPREVKKGRVILNLKVVDKAGNGGVANSRRTHVPSAQQSHFGRQKIPSRN
RVIGKNRRFGELSYRGIQTPIRSSGFSVFGKLFDSHSLNNAENQTQSGESRNNTTNDLSSSQSSKVDASKGQTLDELP
PSNSSSEVSDGEPPSPPQTQSQHSSLQPKSSATKAPDPTLHKLSAQPVPSKNSLGLSALPSSPMFSSSSSASSSSEDS
ERILDLSVPHEMDRRLRRRHPFSGRHPLKVPEVPVSEEPSEEEEDLDWHPDMTSQCANVVVTDITANLLTVTIKEFCH
PPSATPPPCYPKNISAQHDTKQPKHHPNKT

>San_LOC107662367
MELSAAGDRVFAAEAILKRRIRKGRMEYLVKWKGWAIKYSTWEPEENILDERLVAAFEQKEREQEMYGPKKRGPKPKT
LLLKSRAQVAETSSRVPEFKHTRPQQHSKLPPPSAAPSYTPTAPSNAKLQSGTAQPKLKKDIHRCHRMARRPLPRPDQ
TVGPSSPFSSRPTVSPFSETVRILNRKVKPREVKKGRVILNLKVVDKAGNGGVANSRRTHMPSTQQSHFGRQKIPSRN
RVIGKNRRFGEVSYRGIQPTIRSSGFPVFGKLYDSHSLSNAENQTQSGESRNNTTNNLSSSQNSKVDVSKGQTLDELP
PSNSSSEVSDGEPPSPPQTQSQHSSLPPKASATKAQDPALHKLSAQPVASKNSSGPSALPSSPMFSSSSSASSSSEDN
EHILDLSVPHEMDRRLRRRHPFSGRHPLKVPEVPVSEEPSEEEEDLDWRPDMTSQCANVVVTDITANLLTVTIKEFCH
PPSATPPPCYPKNISAQHDTKQPKQHPNKT

>San_LOC107695130
MNMELSSIGEQVFAVESITKKRIRKGNVEYLLKWQGWPPKYSTWEPEDNILDPRLVLAYEEKEEKERALAYKRKGLRP
RQVILRNLYPMDLRSKHKVLGKPTQRIRLSLTRSMGTEIDQNGRRCQRIEKRKNRQCRSKLMNDIKPFQQPRRHPLLP
KDSEKEWDGDEEDEQKKKVKKMRTNEENTTEVHQDIPSGQEMSEGYNSSAEHEAVITIKETENCSSFFDHAEKPREDT
GPVLGATVNSTITNTQENEPVTNTAGDEDSVRVSHDTTNTDQSLHNSTKSGAEQGVFDKVPDRPLVIEVNSSAKCRQD
KVSERGELDRSEAKEKEGMDAVVTAECTTTLQVPIDQLQTPTTTAVHPGKVIVTQVTINTLTVTFKEAMSAEGFFSGY
GL

>San_LOC107672137
MNMELSSIGEQVFAVESITKKRIRKGNVEYLLKWQGWAPKYSTWEPEDNILDPRLVLAYEEKEEKDRALAYKRKGLRP
RRVILRSIYPMDLRSKHKVPDKPTPRIRLSLTRSMGTEIDQNGRRYRPIEKYKNRHCRSKLMNDIKPSQYSLPAKDSE
KEWDEDDEDEQENKVKKMRENEEKTTEVHQDIPSSQETTEGYSSSAEDEAVITIKETENCSSIYDHAEKPSEDTSQVL
GATENSTITNTQENEPVTNTTGGEDSVRVSHDTTNTDQSLHGTESGAKQVVFDQVQNRPSVIEVHSSTKHRQEEVSES
GEVDHSEAKEKEGMDAEITAERTLLQVPIDQSETPSTTTVHPGKVTVIQVTINSLTVTFKEAMSAEGFFSGDGLEV

>San_LOC107692056
MELSAIGEQVFAVESIIKKRVRKGHVEYLLKWKGWPPKYSTWEPEEHILDPRLVLTYEEKEERERSVRWRKRGPKPKR
LFMQRNIYAMDLRSAHKDTEKSSAHLPLSLDPRFQSTGACIYQRLTHHKKKKKTSRKTSEEEWDRREEEDDEDDDDEG
VMEEEEEEEEEEDTRQGGTKTGSNTLNKHVRRGRWSPTTESEAMTISPLPEDWSPVMGPEEVIVTDITINSLTVTFRE
ALVARGFFRSWEMEI

>San_LOC107660863
MELSAIGEQVFAVESIIKKRVRKGHVEYLLKWKGWPPKYSTWEPEEHILDPRLVLTYEEKEQRERSVRWRKRGPKPKR
LFMQRNIYAMDLRSAHKDTEKSSVHLPLSLDPRFQSTGACIYQRLTHHKKKKKASRETSEEEWDRREEEDDDDDDDDD
EGVMEEEEEEEKEEEDTRQGGTKTGSNTLNKRVRSGRWSPTTGSEAMTISPLPEDWSPVMGPEEVIVTDITINSLTVT
FREALVARGFFRSWEMEI

>San_Cbx8 (Gene ID: 107701205)
MELSAVGERVFAAESIIKRRIRRGRMEYLVKWKGWSPKYSTWEPEENILDSRLFVAFEERERERSEIFGPKKRGPKLKT
FLLKAQTKEKAKSYEFRNESFRGITYPSPEPVVTPRAREGLRAVVPSIFPPSTVNRGESVRIPPPEPREHRSPHSPRS
STDAFTLAPKKRGRKPKLRFTDGYTSSLHPEHLKRAADETMASIPSKMAKLGLGEEEERCSEISGRIKISHKHMDATY
SHKQNRVVPSRSTQHISPEWSLHSSRVSADLQGRRNNPQTSHFHHKHLKYHSKRRTFEHADSTSRHPSLIAKIPVSRI
FGEPEEGDAWRPSFSNVEKVIVTDVTSNFLTVTIKESSTDKGFFKDKR

>San_LOC107669528
MELSAVGERVFAAESIIKRRIRRGRMEYLVKWKGWSPKYSTWEPEENILDSRLFVAFEEREREREIFGPKKRGPKLKT
FLLKAQAKEKAKSYGFRNESSRGIAYPSPEPLVTPRAREGLRSVVPTIFPPSTVNRGESVRIPPPEPREHRSPHSPRP
STDAFTLTPKKRGRKPKLRFTDGYTSSLHPEHSKRAADETMASIPSKMAKLGLGEEEERCSEISGRIKISHKHMDATY

SHKQNRVVPEWSLHSSSVSADLQGHRTNPQTSHFHHKHLKHYSKRRTFERGDSTSRQPSLIAKIPVSRIFGEPEEGDA
WRPTFCNVEKVIVTDVTSNFLTVTIKESSTDKGFFKDNR

>San_LOC107670683
MELSAVGERVFAAESIIKRRIRRGRMEYLVKWKGWSQKYSTWEPEENILDERLFAAFEEREREREMYGPKKRGPKPET
FLMKAKAKAKGKNCEFRREMSQDLHVSFPVAEPVVTPRAREGLRAVVPTIFPPSTINRGESVRVRPPVSERDPLMHGM
LVQRPLDFASSPKKRGPKPKLRPGGSSTEGVKRKADEPLSYRPSKTERSGETSNCDVIHLTQKFQAESSHVKKQMGSR
SSDVKFTHGGNILKAGLGVLEHRRKDSPSGAIKHSKMKHPPKSNLFRSTDQTREQLSLSFVDETDQSWLPCLKNMEKI
VVTDVTSNSLTVTIKESSTDKGFFKENR

>San_LOC107689994
MELSAVGERVFAAESIIKRRIRRGRMEYLVKWKGWSQKYSTWEPEENILDERLFAAFEEREREREMYGPKKRGPKPET
FLMKAKAKAKGKNYEFRREMSRDLHVSFPVAEPVVTPRAREGLRTVVPTIFPPSTINRGESVRVRPPDLERDALTHGI
QRPLDFASSPKKRGPKPKLRPGGSSAEGVKRKADEPLSYRPSKTERSGETSNCDGIHLTQKFQAESSHVQKQMGSRSS
DVKFTHGGTILKAGLGVLGHRRKDSSSGAISQSKMKHPSKNHLFRSTGQAREQLSLSFVDETDQSWLPCLKNMEKIIV
TDVTSNSLTVTIKESSTDKGFFKENR

>San_LOC107661146*
MECCSCAPPTRHPQGQGTGRPSQEVRSRLHPRTMGLALHKRPSPSAPVQEVESQPAGCPRGERDQEEAVNESPPPIIV
DGEEAYQVQEILDSRRRGRLLQYLIDWEGYGPEERSWVNSQDILDPSLTTEFHRTHPHKPAPRSRGRPRRRSPPRFRS
RSEVVF

>San_LOC107669533*
MRLPCKKLSPRYVGPFTIIKQINPVTYQLQLPSQYKIHSSFHVSLLKPYHPPVSVSTEPGPTEEPPLPLIQEDGAIYK
VNEILDSRRRGGILEYLVDWEGYGPEERSWVPRVDILDPTLLEAFHSTHPDRPGPRRRGRPPRHRGHRPLGEGRGGWG
TVTDQPGSSTTQSQRTLSPEY

>Cva_LOC107089316
MEELSAVGEQVFDAECILNKRLRKGKLEFLVKWRGWSSKHNSWEPQENILDPRLLAAFNKKEQEKELLMLKRRKRPRG
RPRKIIENIPEPQKSSSSSSASSSTSSDSSSSCTSSSSSSSDDEDEGDDVKQASPTVRPRELHPVPQKKAQIVMAKQE
PPRKRSRKPLPSDAKDFHQNKGQRKMVKTTKETELPGAIKKPVHPASFTFMGFHRSSAKDPVGGQYRSPLSPSAVKNP
ISTAGSGRPSVQSSSLPLNKSNQNRNTSEGKVSISSMNSGASLDLKAAACKSKGVAALNLNTSKHPVQGSPQRTLSPP
GGQKKPQPTGSTAQRGPTTKTAPSQPPKNASSNQGSSLQPLNLQNKQLQVSDAPGSGVTAASGPRNPVVPARKTTATQ
NPETGAVKSPVTSGRSPARKPQPAGDKFRGVNEIQNAKVQNRLDKSYTEIQNPQERTATKGSKKAKMTDMSTGEEESS
SDSDRDSSYAGQGHPVGGHNQDWKPTRSLIEHVFVTDVTANLVTVTVKESPTSVGFFSIRNY

>Cva_LOC107082907
MNTDSLPEALMISFYSIETHNSWEPEENILDPRLLAAFHKREQERELLFQKKGKRPRGRPRKILPPPTATKDSRSSSS
SSSSGLSSEPSSSSEDEDHIKKSKPSPRVHPVPQKRPQILLAKPDPPRKKKRGRKPLHPDLKALRQGKSLPPPPPPPP
RHHQPIRSPRDDLRPGVKKPLQPASFTYPGLSRSSREEGSSSSFSQSSASKPGSLSCIWTGRSLSSSSSSSSHNKGG
ASQKPSELRRSFSETGSSRAEAFKASPVKSAGGLAPSGLHSGFGGHPIAAQRPQLAHRRQDGQTGPVQHKQNSGFSKQ
NPSTKERPSQALNLRALNLQSVGKLPAAGGLQGGAAAGGQRSGPALESGRSRSQPSAAGRQEERKHGLSLTELSTGDS
DDTSSSESEHDAPSLPDDSRPGVGGNATESDTETDWRPARSLMEHVFVTDVTANFITVTVKESPTSVGFFSSRNH

>Cva_Cbx4 (Gene ID: 107085514)
MELPAAGEHVFAVESIEKKRSRKGRVEYLVKWRGWSPRYNTWEPEENILDPRLLDAFQDRERQEQLMGYRKRGPKPKH
LLVQVPSFAKRSNFLANLQEASLEDDSCQKSSPIQMIRPQSQQFQLNNKKLHQYQPLSREHEAEQQPNGKKFFYELNS
KKHHQYQPDLKSQEPVFNKPRDIQAPELANKGYNLPAVLQQKWVRDKDSGCLTKVKDITMELKKLPADLNGQKEPERI
KPKEDTSPHPNGVSSSKLKIVKNKNKNGRIVIVMSKYMENGIQSAKTKHGDSDGAERPLQGADSSMERHLAKMKLVKK
LGLMNGFPKEPKDNPTPPSPGINGDCCKEKELSTQTDRTVAEQDKPTAVGGEGQFPQHQPLQLTTKPDLLHVPPERGA
LDFKGTQNGLHGLKRHLTDADAEEHGCSKRLISNKSISVLNTIPPPSQSPAFDQNGLSPALLQDYGYADQEEPMDLSI
VKSRPVSSVFCETEPETQTEEHVHSHDTQTQTDTKTESQTETHSPSVEERVDSVSGSPHQAKKEEAFPSFQPSLGNIV
ITDITANCLTVTFKEYITA

>Cva_LOC107092841
MELPAAGEHVFAVEGIEKKRIRKGKIEYLVKWRGWSPKYNTWEPEENILDPRLLVAFQHRERQEQMMGYRKRGPKPKH
LLLQVPSFARRSTIPAGFQETANEADGTLKSDSTQVQRSQPQQYQLNSKKHHQYQPSSQEVPTDALSNSKKKFIYQLN
SKKHHHYEPDPHMYDAQVSRLKEVVKVQEPASNLANPGWTLPLALQQKWIHNKDTGCLSKVKELAVEVRKPVKEAQSE
NALKPNPKEASLPTAVSSKMKIIKNKNKNGRIVIVMSKYMDGNKVHGAKDKHRESSRDEKTHGIKTSENNPAPMTKID
EHPENGIPKEICKGSSLSVAEHPLKCSPKDRQFSKPSPSTAEEYNTEVARGQADLPEGLSLQLTASSPQTSWTADTAI
PTPTAIDQIRIPSFPCNRKRKLSDPIEDRNVGQIYLASRSLSVPAATMAPPQDKPMDLHCSTARHSGMCTTYQEVGSQ
EEPMDLSCPKNKRPVEVEAQPPAKPEPAIEDTSSAVNNTQKSLDKSPEEPAKQISPFMGNIIITDITTNSLTVTFKEY
VSF

>Cva_Cbx6 (Gene ID: 107090076)
MELSAAGDRIFAAEAILKRRVRKGRIEYLVKWKGWAMKHSTWEPEENILDDRLILGFEQKEREREMHGPKKRGPKPKN
LAMKDRGQKGESSSRAFSSRQTAPRSSSSTSNRAAPSSSASSSAAPHTLPLSSSSPAPSPKLNSLAATHKLKKDIHRC

HRMSRRPLPRSHPVGPAFSSSSGFPSRMHVSPFSETVRILNRRVKPREVKRGRIILNLKVIDKPGRGGAAAGSRNGQS
GRQNIPSRNRIIGRKGDAPYRPFQPPLKMLGFPMYGKPFGLQCGRPMSFHSQTGSCSSTGARDTNSSSSQCQAPPPSS
APSNSSSNAAKRPQPAAEASKDSESSTAVPETQKPSASSCSEANDKVPASSSPTSSLEDQDEDDMDCSQASEEGRSPG
HHKAKPQSPSAVSSATPDQPTAPADPQRVPTEGDPDWHPEMAPSCKDVVVTDVTTNLVTVTIKEFPTPASGPASPASD
ENASTPPPAASSENAAIPKP

>Cva_LOC107092875
MELSAIGEQVFAVESILKKRLRKGNVEYLLKWKGWPPKYSTWEPEEHILDRRLVQAFEDKEEKERVVGHRRKGSKSKR
VLLQNTVYTMDLRSAHKIPAKPPPRLRLSLTRSLLPDDDDEDEDEDETYVPCRTGSRIQAALRKSTRSRCLNLDHQSS
SPEEWEEEEEENSAEDSEHREEEEEAAEMREAVFSGHRNADKWSTDGAAESEQLWRPVMAPGEVTVTDVTLNSLTVTF
RESRVAKGFFQDWGLEVWDQVGGEGSERATWDP

>Cva_LOC107093081
MELSSIGEQVFAVESITKKRIRKGHVEYLLKWQGWPPENSTWEPEDNILDPLLVMAYEANQEKLRSLSFRKKGLRPRK
LVLRNIFGMDLRSAHKDDEKPPPRLRLSLTRSMSTDVEQACRRQARRRSRQRVTKVYSKHSKSFHGQKMKLGLMEKDW
GGTSEEEKNGCDSNTEERCGDSLYGQSECSSTPFLEEEDMDMEEEEKNVSPDLWPNGVIFATIPNHTNALEQSKDNTL
ASEAKPEGLVPPSGRSHSAEAKGVEACSEPPKEEKKNATSVIVRFQGSGKTAGEAVSVPHESELKKNEARSDNQSVIV
TASERPKGPTEAPRPGKVIVTNVTVNSLTVTFKEATGAEGFFNGF

>Cva_LOC107092814
MELSAVGESVFAAESIIKRRIRRGRWEYLVKWKGWSQKYSTWEPEENILDERLFAAFEERERERELFGPKKRGPKPET
FLLKAKAKAKEKTYDFRRETPRSIQVSYPIPEPVITPRAREGLRAVVPTIFPPSAINRGESVPIRSPEPERRPRPAMT
EALFDQESGRFPRKRGRKPKFHPHYDPHDPSISAEPVSKRSRSLDEQSITSRRLHYHGETSEHSLLQLTKRFQEETTI
TPKPSSEQRYAGMSYTCALSPGMRKSDQEGHRTYSLNRMHFPKHSELKRSAEELPSQPDPSAPSAPTWTPCFTNWDTV
TVTDVTMNLLTVTIRESSTAKGFFKDKR

>Cva_LOC107085518
MELSAVGERVFAAESIIKRRIRKGSIEYLVKWKGWSPKYSTWEPEENILDSRLFAAFEQREREREL YGPKKRGPKPKT
FLLKAQAKVKTKSYEFRSEAVRGMHITYPTPEPVFTPRAREGLRAVVPTIFPPSTINRGESVRVIPHEHQPSPQQSVS
DRLVQTPKKRGPKPKPRFIGDGRSPVVLETSKRIAEEVGGQGPYKLSKFHGDEMRLQKLSHRHSEDHPRSSKHHYQHH
YHHHLAQKLSSGGSYKRFLSHHSTHPHRTKDRCSYLAAAQVKHHQKTNQSRSGAAEFPQMEKPYFLDRQSPTKLDDDD
QEETTWRPSLSDVEKVLVTDVTANFLTVTIKESSTSKGFFKDKR

>Xma_LOC102229509
MEGVTVGQVFDAECILSKRQRKGKFEYLVKWRGWSSKHNSWEPEENILDPRLLAAFHKREQERELLFQKKGKRPRGRP
RKILPPPADTKDSHSSSSSFGLSSEPSSSSEEDDDDHVKKAKQSPRIHPVPQKRPQILLAKPDPPHKKKRGRKPLHPD
LKALRQGKSLPSPPRHHQPIRSPRDEPRPGVKKPLQPASFTYPGLSRSSREEGASSFSQSSTSKPGSLNCVWAGRSLS
ASSSHNKGSPSQKPSELRRAVSETGSVRADAFKGSAQKPAPLGLHSSFGGHPIAAQRPPLTHRRHDNVGGQSGLVQHK
QQNSSFSKQSPSPTPRERTSQALNLRALNLQSVGKLPGSGGLQGGSRSTGVSKGTTAGGQRSGPGAAEPGRSRTQPAG
GSRQEEHKHGSSSQNRNLNELSTGDSDDTSSSESEHDPSSFPGDTRPSVSSNTAESDTETDWRPARSLMEHVFVTDVT
ANFITVTVKESPTSVGFFNSRNH

>Xma_LOC102223386
MEELSAVGEQVFDAECILNKRLRKGKLEFLVKWRGWSSKHNSWEPQENILDPRLLAAFNKKEQEKELLMLKRRKRPRG
RPRKIIENIPEPQKSSTSSSASSSSSSDSSSSCSSSSSSSEDEDEGDDGHAKQASPTVRPRELHPVPQKKAQIVMAKQ
EPLRKRNRKPLPPEAKEFQQNKGPRKVLKTARETDLPGAIKKPVHPASFTFMGFHRSSAKDAAPGPYRSPLTQGGTVK
PATSSPGSGRSSVQTVSLALNKSNQIRNATEGKLSISGGNSGGSLDLKAAACKSKGVAALNVNTTKHPVQGTTQHTLS
SPSGPKKPHSTGSAMQRVAGTKAGVSQSKSSSSNQGNQPLNLQNKQSQSGDAPGSGTTAALGLRNPVIPARKPTVAQN
LETNANKSPVSSGRPPARKPQAGGDKLREMKEFQKVQGRLDKSCSEIQNPPARTATKGSKKAKMTDMSTGEEESSSDS
DHDSSYAGQGHPVAGQNQDWKPTRSLIEHVFVTDVTANLVTVTVKESPTSVGFFSIRNY

>Xma_Cbx4 (Gene ID: 102230727)
MELPAAGEHVFAVESIEKKRSRKGRVEYLVKWRGWSPRYNTWEPEENILDPRLLDAFQDRERQEQLMGYRKRGPKPKH
LLVQMPSFARRSNILANLQEASLEGNSCQKSSSIQMICPQAPQFQLNSKKLHHYQPLIRECDPEQQSNGKKFFYELNS
KKHHHYQPDLKQHEPVFAKPRDVKAPELSNKGYNLPAVLQQKWVRDKDSGCLTKVKDITMELKKIPADLKGQKEPERV
KPKEDASTHSNGVSSSKLKIVKNKNKNGRIVIVMSKYMENSMQTAKIKSSDSEGAERPLQGAESSMEGRLAEVKLVKK
LSLINGFSKQPKDNPTVPTSGIKADCCKEKELSPQTERTVMEQDKGAEGQLPQDKPLQLTNKPDELPLPSEREVPDKR
GIQNGHHGLKRHLSDTDAEEQGLGKRFLSYRSISAPNTDSSPSQSFIVDRNEQQSPALLHNYGYADQEEPMDLSIVKS
RPTSLVCVGTEPETRAEEPAHPPDDSRTQADTQTESQTETHNLSEEESADIVSDSNHQARKDEAFPSFQPCLGNIVIT
DITANCLTVTFKEYVTA

>Xma_LOC102232224
MELPAAGEHVFAVEGIEKKRIRKGKIEYLVKWRGWSPKYNTWEPEENILDPRLLVAFQHRERQEQMMGYRKRGPKPKH
LLLQVPSFARRSNIPAGFEETVQDTEGTFKLDPVQRPQPQHYQLNSKKHHQYQPNSQEIPADSLNNSKKKFIYQLNSK
KHHHYEPDPHMYDAQVSRLKEVVKVQEPASKLANPGWNLPLALQQKWIRNKDTGCLSKVKELAVEVRKPVKDAQSENA
LKPNPKEATLPSAVSSKMKIIKNKNNNGRIVIVMSKYMDGNKVHGAKDKHRESSRDEKTQSSKTQENNPPPGAKMTEH

PENGIPKEICKGSSVSVAEHPLKCSPKDRHFSKPSPSTAEEYNTEVARGQADLPDDLPLQLTASSPPTSWTADTAIPT
PTAVDQIRIPSFPCDRKRKLSDPVEDRNLGKILLASRSLSVPTAAVTPPQDKPMDLHCSVARRSSMCTMYDANGSQEE
PMDLSCPKNRRQVEAEVQPPVEAEPVVEDTPPPKENTQKSCEKSPEEPANKISPFMGNIIITDITTNSLTVTFKEYVS
F

>Xma_Cbx6 (Gene ID: 102234048)
MELSAAGDRIFAAEAILKRRVRKGRLEYLVKWKGWAMKHSTWEPEENILDDRLLLGFEQKEREREMHGPKKRGPKPKN
VVAKGRGQKAEPTSRASSSRQNTPRSSSTASSRASSSSSAAPNALPFSSSSSSSAPAPSPKLNSLAATHKLKKDIHRC
HRMSRRPLPRADPTRPAFSSSSGFQSRMHVSPFSETVRILNRRVKPREVKRGRIILNLKVIDKPGRGGGGAAGSRNVQ
TGRQNIPSRNRIIGRKGEAPYRPFQPPLKMLGFPMYGKPFGLQCGGPMSFHSQTGSSSTTGARHSNSSSSQRQALPPS
SSSSSTSTNVTAKHPQPATEASKESKSSKSPETQNASCSEANKILVPPSPSPSLEDQDEDAMDCSEASEEARSPCQHK
APSTVSSVAPEQSSLPPESKRVPAEGDPDWHPEMAPSCKDVVVTDVTTNLVTVTIKEFPSPASGPASPSASVENGSTP
PPAATSEDSSVPKP

>Xma_LOC102225821
MELSAIGEQVFAVESILKKRLRKGNVEYLLKWKGWPPKYSTWEPEEHILDRRLVQAFEDKEEKDRVIGHRRKGSKSKR
LLLQNTVYTMDLRSAHKIPAKPPPRLRLSLTRSLVPDDEEDEDGACVPSRTHRKSKQSRRLSLNLQGPSQEAWEEEEE
EEEEENSAEDSEHREEEEAEMGEGVFNGHRGPDNWSTAVGSGGSDKLWRPMVAPGEVTVTDVTLNALTVTFRESRAAR
GFFRDWGLEVWEQVGDGAPQSCWRH

>Xma_LOC102232562
MELSSIGEQVFAVESITKKRIRKGTVEYLLKWQGWPTEYSTWEPEDNILDPLLVLAYEENQEKLRSLSFRKKGLRPRK
LVLRNIFGMDLRSAHKDDEKAPPRLRLSLTRSMSTDVEQACRRRARRRNRQRVTKAFPKHSSNKPIHEQKMKVEPVEK
DWGGTSEEDKPGCESTTEERREDSLYGQSECSSPPFLEEQDMDTEVEEKAASPDMWTDEADGRIFETSQRDTNSQNES
ESEAKPEVVVSPSARSDWVKEVGVEAFSESPTMEKKNATSVIVRVQECGKTTGDAVSVCSDSEPQKDEVRSDNQSVIV
TASDRPKVPYEAPRPGKVIVTNVTVNCLTVTFKEASCSDGFFNGC

>Xma_Cbx8 (Gene ID: 102230986)
MELSAVGERVFAAESIIKRRIRKGRIEYLVKWKGWSPKYSTWEPEENILDSRLFAAFEQREREREЛYGPKKRGPKPKT
FLLKAQAKGKTKSYEFRSEAVRGMHITYPTPEPVFTPRAREGLRAVVPTIFPPSAVNRGESMRVRPSELSLHGHQRSS
LQHSDSDGTVQAPKKRGPKPKPRFVDADFSPVVSEPRKRRAEEEEEEEEEEEERHGPHKLSKFQGAEEMRLQKLAHRHS
ENHKHHQHHHYYHHHHHHTQKVSQLHSHRSLEPHRTTDGSSYLVAAHLKHHHKASRGPPTEFPPMEKPYFLDRQSPTR
FDDSQDQTAWRPCLSNVEKVLVTDVTANFLTVTIKESSTSKGFFKDKR

>Xma_LOC102227807
MELSAVGESVFAAESIIKRRIRRGRWEYLVKWKGWSQKYSTWEPEENILDERLFAAFEERERERELFGPKKRGPKPET
FLLKAKAKAKEKTYEFRREAPRGIQVSYPIPEHVITPRAREGLRAVVPTIFPPSAVNRGESVPIRPPEPERRPRPVPL
EALLDQESRGSPKKRGRKPKLHPHYDPHDASGSSEPAPKRSRSLEEQSIMSRRLHHHGETSDHSLLQLTKRFQEETTI
TPKSNSEQKHAGLSYTCAFSPGERKRDPEGHRTYSLSRLDFPQHGKLKRSAEERGHQAAVLSSQADPTAPSAPTWTPC
YTNWDTVTVTDVTMNLLTVTIRESSTAKGFFRD

>Pfo_LOC103146900
MEELSAVGEQVFDAECILNKRLRKGKLEFLVKWRGWSSKHNSWEPQENILDPRLLAAFNKKEQEKELLMLKRRKRPRG
RPRKIIENIPEPQKSSTSSSASSSSSSDSSSSCSSSSSSSEDEDEGDDSRAKQASPTVRPRELHPVPQKKAQIVMAKQ
EPPRKRNRKPLPPEAKEFQQNKGPRKVLKTAKETDLPGAIKKPVHPASFTFMGFHRSSAKDAAAGPYRSPLTQGGAVK
PAISSPGSGRSSVQTASLALSKSNQSRNATEGKLSIGNSGGSLDLKTAACKSKGVAALNVNTTKHPVQGTTQHTLSPP
SGPKKPQSTGSAMQRVAGTKAGTSQSSKNTSSSQGHQPLNLQSKQSQSGDAPGSGTTAASGLRSPVIPARKPTVAQNL
EANANKSPVSSGRPPARKPQAGGDKLREMKEFQKVQGRLDKSCSEIQNPPARTATKGSKKAKMTDMSTGEEESSSDSD
HDSSYAGQGHPVAGQNQDWKPTRSLIEHVFVTDVTANLVTVTVKESPTSVGFFSIRNY

>Pfo_LOC103147077
MEGVTVGQVFDAECILSKRQRKGKFEYLVKWRGWSSKHNSWEPEENILDPRLLAAFHKREQERELLFQKKGKRPRGRP
RKILPPPTDTKDSHSSSSSFGLSSEPSSSSEEDDDDDHVKKAKQSPRIHPVPQKRPQILLAKPDPPRKKKRGRKPLHP
DLKALRQGKSLPSPPRHHQPIRSPRDEPRPGVKKPLQPASFTYPGLSRSSREEGASSFSQGSTSKPGSLNCVWAGRSL
SASSSHNKGSPSQKPSELRRAVSETGSVRADAFKGSAQKPAPLGLHSSFGGHPIAAQRPPLTHRRQDSVGGQSTGLVQ
HKQQNSSFSKQLPSPTPRERTSQALNLRALNLQSVGKLPGSGGLQGGSRSTGVGKGTTAGGQRSGPGVTEAGRSRTQP
AGGSRQEEHKHGPSSQNRNLNELSTGDSDDTSSSESEHDPSSFPDDTRPSVSSNAAESDTETDWRPARSLMEHVFVTD
VTANFITVTVKESPTSVGFFNSRNH

>Pfo_Cbx4 (Gene ID: 103133063)
MELPAAGEHVFAVESIEKKRSRKGRVEYLVKWRGWSPRYNTWEPEENILDPRLLDAFQDRERQEQLMGYRKRGPKPKH
LLVQVPSFARRSNILADLQEASLEGNSCQKSSSIQMMCPQAQQFQLNSKKLHQYQPLIRECDAEQQSNGKKFFYELNS
KKHHHYQPDLKQHEAVFAKPRDVKAPELSNKGYNLPAVLQQKWVRDKDSGCLTKVKDITMELKKIPPADLKGQKEPER
VKPKEDASTHSNGVSSSKLKIVKNKNKNGRIVIVMSKYMENGMQTAKIKSSDSEGAERQGAESSVERHLAKMKLVKKL
GLINGFSKQPKDNPTVPSSGIKADCCKEKELSPQTERTAMERDKGAEGQLPQDKPLQLTNKPDELPLPSEREVPDKTG
IQNGHHGLKRHLSDTDAAEQGLGKRFLSCRSISAPNTDSSPSQSLTVDRNEQQSPALLHDYGYADQEEPMDLSIVKSR

PTSSVCVETEPETRAEEPAHPPDDSRTQTDTQTESQTETHNPSEEEESADIVPDSNHQARKDEAFPSFQACLGNIVIT
DITANCLTVTFKEYVTA

>Pfo_LOC103141632
MELPAAGEHVFAVEGIEKKRIRKGKIEYLVKWRGWSPKYNTWEPEENILDPRLLVAFQHRERQEQMMGYRKRGPKPKH
LLLQVPSFARRSNIPAGLEETVQDAEGTFKLDPVQRPQPQHYQLNSKKHHQYQPNSQEIPADALNNSKKKFIYQLNSK
KHHHYEPDPHMYDAQVSRLKEVVKVQETANKLANPGWNLPLALQQKWIRNKDTGCLSKVKELAVEVRKPVKDAQRENA
LKPNPKEATLPSSVSSKMKIIKNKNKNGRIVIVMSKYMDGNKVHGAKDKHRESSREEKTQSSKTQENNPPPGAKMTEH
PENGIPKEICKGSSVSVAEHPLKCSPKDRHFSKPSPSTAEEYNTEVARGQADLPDDLPLQLTASSPPTSWTADTAIPT
PTAVNQIRIPSFPCDRKRKLSDPVEDRNVGKILLASRSLSMPTAAVTPPQDKPMDLHCSVAHHSSTCATYDANGSQEE
PMDLSCPKNRRRVEAEVQPPVEAEPAVEDTPPVKDNTQTSCEKSPEEPAKKISPFMGNIIITDITTNSLTVTFKEYVS
F

>Pfo_Cbx6 (Gene ID: 103154832)
MELSAAGDRIFAAEAILKRRVRKGRLEYLVKWKGWAMKHSTWEPEENILDDRLILGFEQKEREREMHGPKKRGPKPKN
FVAKGRSQKAEPTSRASSSRQSTPRSSSTTSSRASSSSSSSSSSAAPNALPFSSSSSSSVPAPSPKLNSLAATHKLKK
DIHRCHRMSRRPLPRTDPTRPAFSSSSGFQSRMHVSPFSETVRILNRRVKPREVKRGRIILNLKVIDKPGRGGGAAGS
RNVQTGRQNIPSRNRIIGRKGEAPYRPFQPPLKMLGFPMYGKPFGLQCGGPMSFHSQMGSSSTTGPRDSNSSSSQRQA
PPPSSSSSSTSSNVTAKRPQSAAEASKESKSSKSPETQNASCSEANKTPVPPSPSPSLEDQDEDAMDCSEASEGARSP
CQHKAPSTVSSVAPEQSSPPLEPKRVPTEGDPDWHPEMAPSCKDVVVTDVTTNLVTVTIKEFPSPASGPASPSASIEN
ASTPPPAAASEDSSVPKP

>Pfo_LOC103143382
MELSAIGEQVFAVESILKKRLRKGNVEYLLKWKGWPPKYSTWEPEEHILDRRLVQAFEDKEERDRVIGHRRKGSKSKR
LLLQNTVYTMDLRSAHKIPAKPPPRLRLSLTRSLVPDDEEDEDEDEDEEDACVPSRRQATHRKSKQSRRLSLNLQRPS
QEAWEEEEEDEDEEEEEEEEEEEEEEKNSAEDSEHLSQPKTTNHSQKEKEEAKMGEGVFNGHRGLDNWSTAVGSGGADT
SDKLWRPLVCPGEVTVTDVTLNALTVTFRESRAARGFFQDWGLEVWEQVGDGAPRSCWRH

>Pfo_LOC103153429
MELSSIGEQVFAVESITKKRIRKGNVEYLLKWQGWPPEYSTWEPEDNILDPLLVLAYEENQEKLRSLSFRKKGLRPRK
LVLRNIFGMDLRSAHKDDEKPPPRLRLSLTRSMSTDVEQACRRRARRRNRQRVTKAFPKHSSNKPIHEQKMKIEPVEK
DWGGTSEEDKPGCESTTEERCEDSLYGQSECSSPAFLEEQDMDMEVEEKAASPDMWTDEADGGIFETSQGDTNSHDES
EDNTLVSEAKPEVMVSPSGKSDWVEEEGVEAFSESPTMEKKNATSVIVRVQGCGKTMGDAVSVCSDSEPKKDEVRTDN
QSVIVTAPDRPKVPYEAPRPGRVIVTNVTVNCLTVTFKEASCSEGFFNGC

>Pfo_LOC103133078
MELSAVGERVFAAESIIKRRIRKGRIEYLVKWKGWSPKYSTWEPEENILDARLFAAFEQREREРELYGPKKRGPKPKT
FLLKAQAKGKTKSYEFRSEALRGMHVTYPTPEPVFTPRAREGLRAVVPTIFPPSAVNRGESMRVRPSELSLHEHQRSS
LQQTDSDGHVQTPKKRGPKPKPRFVDDNFSPVVSEPRKRGTEEQEEEEEEEEEEEEEEEEEETRGPHKLSKFQGDEEM
RLQKLAHRHSENHKRHHHHHYYHHHHHHTQKVSRGSSHKQLHSHRSMEPHRTKDGSGYLVAHFKQPHKASRGRPTELP
PMEKPYFLDRRSPTRLDDSQDQTAWRPCLSNVEKVLVTDVTANFLTVTIKESSTSKGFFKDKR

>Pfo_LOC103141633
MELSAVGESVFAAESIIKRRIRRGRWEYLVKWKGWSQKYSTWEPEENILDERLFAAFEERERERELFGPKKRGPKPET
FLLKAKAKAKEKTYEFRREAPRGIQVSYPIPEPVVTPRAREGLRAVVPTIFPPSAVNRGESVPIRPPEPERRPRPAPL
EALLDQESTRSPKKRGRKPKLHPHYDPHDASGSSEPAPKRSRSLEEQSVMSRRLHHHGETSDHGLLQLTKRFQEETTI
TPKSSGEQKHAGLSYACAFSPGVRKRDPEGHRTYSLSRLHFPQHGKLQRNAEERRHQAAVLSSQADPTAPSAPTWTPC
FTNWDTVTVTDVTMNLLTVTIRESSTAKGFFRDKR

>Gaf_Cbx2 (ENSGAFG00000004995)
MEELSAVGEQVFDAECILNKRLRKGKLEFLVKWRGWSSKHNSWEPQENILDPRLLAAFNKKEQEKELLMLKRRKRPRG
RPRKIIENIPEPQKSSTSSSASSSSSSDSSSSCSSSSSSSSEDEDEGDDSQAKQVSPTIRPRELHPVPQKKAQIVMAKQ
EPPRKRNRKPLSPEAKEFQQNKGPRKVLKTAKETDLPGAIKKPVHPASFTFMGFHRSSAKDAAPGPYRSPLTQGGAVK
PATSSPGSGRSSVQTASLALNKSNQSRNATEGKLSISGGNSGGSLDLKTAACKSKGVAALNVNTTKHPVQGTTQHTLS
PPSGPKKPHSTGSAMQRVAGTKAGASQSKNASSSQGHQPLNLQNKQSQSGDAPGSGTTAALGLRSPVIPARKPTVGQN
LEANANKSSVSSGRPPARKPQAGGDKLREMKEFQKVQGRLDKSCSEIQNPPARTATKGSKKAKMTDMSTGEEESSSDS
DHDSSYAGQSHPVAGQNQDWKPTRSLIEHVFVTDVTANLVTVTVKESPTSVGFFSIRNY

>Gaf_ENSGAFG00000015448
MEGVTVGQVFDAECILSKRQRKGKFEYLVKWRGWSSKHNSWEPEENILDPRLLAAFHKREQERELLFQKKGKRPRGRP
RKILPPPTDTKDSHSSSSSFGLSSEPSSSSEEDDDDHVKKAKQSPRIHPVPQKRPQILLAKADPPRKKRGRKPLHPDL
KALRQGKSLPSPPRHHQPIRSPRDEPRPGVKKPLQPASFTYPGLSRSSREEGPSSFSQSSTSKPGSLNCVWSGRSLSA
SSSHNKGSPSQKPSELRRAVSETGSVRADAFKGSAQKPTPLGLHSSFGGHPIVAQRPPLTHRRHDSVGGHAGLVQHKQ
QNSSFSKQSPSPTPRERSSQALNLRALNLQSVGKLPGSGSLQGGSRSTGVSKGTTAGGQRSGPGAAEPGRSRTQPAGG
SRQEEHKHGSSSQNRNLNELSTGDSDDTSSSESEHDPSSFPGDSRPSVSGNTAESDTETDWRPARSLMEHVFVTDVTA
NFITVTVKESPTSVGFFNSRNH

>Gaf_Cbx4 (ENSGAFG00000020219)
MELPTAGEHVFAVEGIEKKRIRKGKIEYLVKWRGWSPKYNTWEPEENILDPRLLVAFQHRERQEQMMGYRKRGPKPKH
LLLQVPSFARRSNIPAGFEETLQDAEGTFKLDPVQHPQPQHYQLNSKKHHQYQPNSQEIPADALNNSKKKFIYQLNSK
KHHHYEPDPHMYDAQVSRLKEVVKVQEPASKLANPGWNLPLALQQKWIRNKDTGCLSKVKELAVEVRKPVKDAQSENA
LKPNPKDATLPSAMSSKMKIIKNKNKNGRIVIVMSKYMDGNKVHGAKDKHRESSREEKTQSSKTQEDNPLPGAKMTEH
PENGIPKEICKGSSVSVAEHPLKCSPKDRHFSKPSPSTAEEYNTEVARGQADLPDDLPLQLTASSPPTSWTADTAIPT
PTAVDQIRIPSFPDRKRKLSDPVEDRNVGKILLASRSLSVPTAAVTPPQDKPMDLHCSVARHSSMCTTYDANGSQEEP
MDLSCPKNRRRVEAEVQPPVEAEPAVEDTSPAKKNTQKSCEKSPEEPAKKISPFMGNIIITDITTNSLTVTFKEYVSF

>Gaf_ENSGAFG00000007827
MELPAAGEHVFAVESIEKKRSRKGRVEYLVKWRGWSPRYNTWEPEENILDPRLLDAFQDRERQEQLMGYRKRGPKPKH
LLVQVPSFARRSNILANLQEASLEGNSCQKSSSIQMICPQAQQFQLNSKKLHQYQPLIRECDAEQQSNGKKFFYELNS
KKHHHYQPDLKQHEPVFAKPRDVKAPELSNKGYNLPAVLQQKWVRDKDSGCLTKVKDITMELKKIPADLKGQKEPERV
KPKEDASTHSNGVSSSKLKIVKNKNKNGRIVIVMSKYMENGMQTAKIKSSDSEGTERPLQGAESSVERHLAKMKLVKK
LGLINGFSKQPKDNPTVPSSGIKADCCKEKELSPQTERTVMEQDKGTEGQLPQDKPLQLTNKPDELPLPSEREVTDKR
EIQNSHHGLKRHLSDTDAEEQGLGKRFLSCRSISAPITDSSPSQSLVVDRNEQQSPALLPDNGYADQEEPMDLSIVKS
RPTSSVCVETEPGTRAEEPAHPPDDSRTQTDTQIESQTETHNPSEEESTDIVSDSNHQARKDETFPSFQPCLGNIVIT
DITANCLTVTFKEYITA

>Gaf_ENSGAFG00000007822
MELSAVGERVFAAESIIKRRIRKGRIEYLVKWKGWSPKYSTWEPEENILDSRLFAAFEQREREREELYGPKKRGPKPKT
FLLKAQAKGKTKSYEFRSEAVRGMHITYPTPEPVFTPRAREGLRAVVPTIFFPPSAVNRGESMRVRPSELSLHGYQRSS
LQLTDSDGTVQAPKKRGPKPKPRFVDDDFSPVVSEPHKRRADEEEEEEEEEEEEQEEEEEEDEEERHGPHKLSKFQGD
QERRLQKLVHRHSESHKYHQHHHYYHPHPHHHHTQKVSRGSSHEQLHSHRSMEPHRTTDGSSYLVAAHLRHHHKVSRG
QPTEFPPMEKPYFLDKQSPTRFDDSQDQTAWRPRLSNVEKVLVTDVTANFLTVTIKESSTSKGFFKDKR

>Gaf_Cbx7a (ENSGAFG00000010693)
MELLSIGEQVFAVESITKKRIRKGTVEYLLKWQGWPPEYSTWEPEDNILDPLLVLAYEENQEKLRSLSFRKKGLRPRK
LVLRNIFGMDLRSAHKDDEKPPPRLRLSLTRSMSTDVEQACRRQARRRKRQRVTKAFPKRSSNKPIHEQKMKVEPVEK
VWGGTSEEDKAGCESTTEERCEDSLYGQSECSSPPFLEEQDMDMEVEEKAASPDMWTDEVDGGIFETSQRHTNSHDKL
EDNTLVSEAKPEVVVFPSSRSDWVEEEAFSESPTMEKKNATSVIVRVQGCGKTTGDAVSVCSDSEPKKDEARSDNQSV
IVTASDRPKAPYEAPRPGKVIVTNVTVNCLTVTFTEASCSEGFFNGC

>Gaf_Cbx7b (ENSGAFG00000008146)
MELSAIGEQVFAVESILKKRLRKGNVEYLLKWKGWPPKYSTWEPEEHILDRRLVQAFEDKEEKERVIGHRRKGSKSKR
LLLQDTVYTMDLRSAHKIPAKPPPRLRLSLTRSLVPDDEEDEDEDEDDAWVPSRTHRKSKQGRRLSLNVQSPSQDAWE
EEEEEEEEEENSAEDSEHREEEEAEMGEGVFNGHRGPDNWSTAVGSGGAAASDKLWRPMVAPGEVTVTDVTLNALTVT
FRESRAARGFFRDWGLEVWEQTETVALETAWLDRLL

>Gaf_Cbx8a (ENSGAFG00000020216)
MELSAVGESVFAAESIIKRRIRRGRWEYLVKWKGWSQKYSTWEPEENILDERLFAAFEERERERELFGPKKRGPKPET
FLLKARAKAKEKTYEVRREAPRGIQVSYPIPEPIVTPRAREGLRAVVPTIFFPPSAVNRGESVPIRPPEPERRPRPVPL
EALLDQESRRSPKKRGRKPKLHPHYDQHDASGSSEPAPKRSRSLEEQSIMSRRLHHHGETSDHSLLQLTKRFQEETTI
TPKSNSEQKHAGLSYACAFSPGVHKRDPEGHRTYSLSRLHFPQHGKLKRSAEEQRHQAAVLSSQADPTAPAAPTWTPC
YTNWDTVTVTDVTMNLLTVTIRESSTAKGFFRDKR

>Pre_Cbx2 (Gene ID: 103469046)
MEELSAVGEQVFDAECILNKRLRKGKLEFLVKWRGWSSKHNSWEPQENILDPRLLAAFNKKEQEKELLMLKRRKRPRG
RPRKIIENIPEPQKSSTSSSASSSSSSDSSSSCSSSSSSSSEDEDEGDDSRAKQASPTVRPRELHPVPQKKAQIVMAKQ
EPPRKRNRKALPPEAKEFQQNKGPRKVLKTAKETDLPGAIKKPVHPASFTFMGFHRSSAKDAAAGPYRSPLTHGGAVK
PAISSPGSGRSPVQTATLALSKSNQSRNATEGKLSIGNSGGSLDLKTAACKSKGVAALNVNTTKHPVQGTTQHTLSPP
SGPKKPQSTGSAMQRVAGTKAGASQSSKNTSSSQGHQPLNLQNKQSQSGDAPGSGTTAASGLRSPVIPARKPTVAQNL
EANSNKSPVSSGRPPARKPQAGGDKLREMKEFQKVQGRLDKSCSEIQNPPARTATKGSKKAKMTDMSTGEEESSSDSD
HDSSYAGQGHPVAGQNQDWKPTRSLIEHVFVTDVTANLVTVTVKESPTSVGFFSIRNY

>Pre_LOC103481889
MEGVTVGQVFDAECILSKRQRKGKFEYLVKWRGWSSKHNSWEPEENILDPRLLAAFHKREQERELLFQKKGKRPRGRP
RKILPPPTDTKDSHSSSSSFGLSSEPSSSSEEEDDDDHVKKAKQSPRIHPVPQKRPQILLAKPDPPHKKKRGRKPLHP
DLKALRQGKSLPSPPRHHQPIRSPRDEPRPGVKKPLQPASFTYPGLSRSSREEGASSFSQSSTSKPGSLNCVWAGRSL
SASSSHNKGSPSQKPSELRRAVSETGSVRADAFKGSAQKPAPLGLHSSFGGHPIATQRPPLTHRRQDSVGGQSGLVQH
KQQNSSFSKQLPSPTPRERTSQALNLRALNLQSVGKLPGSSGLQGGSRSTGVGKGTTAGGQRSGPGAAEAGRSRTQPA
GGNRQEEHKHGPNSQNRNLNELSTGDSDDTSSSESEHDPSSFPDDTRPSVSSNAAESDTETDWRPARSLMEHVFVTDV
TANFITVTVKESPTSVGFFNSRNH

>Pre_Cbx4 (Gene ID: 103463659)
MELPAAGEHVFAVESIEKKRSRKGRVEYLVKWRGWSPRYNTWEPEENILDPRLLDAFQDRERQEQLMGYRKRGPKPKH
LLVQVPSFARRSNILADLQEASLEGNSCQKSSSIQMICPQAQQFQLNSKKLHQYQPLIRECDAEQQSNGKKFFYELNS
KKHHHYQPDLKQHEAVFAKPRDVKAPELSNKGYNLPAVLQQKWVRDKDSGCLTKVKDITMELKKIPADLKEQKEPERV

KPKEDASTHSNGVSSSKLKIVKNKNKNGRIVIVMSKYMENGMQTAKIKSSDSEGAERPLQGAESSVERHLAKMKLVKK
LGLINGFSKQPKDNPTVPGSGIKADCCKEKELSPQTERTAMERDKGAEGQLPQDKPLQLTNKPDELPLPSEREVPDKT
GIQNGHHGLKRHLSDTDAAEQGLGKRFLSCRSISAPNTDSSPSQSLAVDRNEQQSPALLHDYGYADQEEPMDLSIVKS
RSTSSVCVETEPETRAEEPAHPPDDSRTETDTQTESQTETHDPSEEESADIVPDSNHQARKDEAFPSFQACLGNIVIT
DITANCLTVTFKEYVTA

>Pre_LOC103469229
MELPAAGEHVFAVEGIEKKRIRKGKIEYLVKWRGWSPKYNTWEPEENILDPRLLVAFQHRERQEQMMGYRKRGPKPKH
LLLQVPSFARRSNIPAGLEEPVQDAEGTFKSDPVQRPQPQHYQLNSKKHHQYQPNSQEIPADALNNSKKKFIYQLNSK
KHHHYEPDPHMYDAQVSRLKEVVKVQETASKLANPXWNLPLALQQKWIRNKDTGCLSKVKELAVEVRKPVKDAQSENA
LKPNPKEATLPSAVSSKMKIIKNKNKNGRIVIVMSKYMDGNKVHGVKDKHRETSRDEKTEXSKTQENNPPXGAKMTKH
PENGIPKEICKGSSVSVAEHPLKCSPKDRHFSKPSPSTAEEYNTEVARGQADLPDDLPLQLTASSPPTSWTADTAIPT
PTAVDQIRIPSFPCDRKRKLSDPVEDRNVGKILLASRSLSVPTAAVTPPQDKPMDLHCSVARHNSTSATYBANGSQEE
PMDLSCPKNRRRVEAEVQPPVXAEVQPPVEAEPAVENTPPAKENEQKSCEKSPEEPAKNISPFMGNIIITDITTNSLT
VTFKEYVSF

>Pre_Cbx6 (Gene ID: 108166384)
MELSATGDRIFAAEAILKRRVRKGRLEYLVKWKGWAMKHSTWEPEENILDDRLILGFEQKEREREIHGPKKRGPKPKN
FAAKGRGQKAEPTSRASSSRQSMPRSSSTTSSRASSSSSSSSSSAAPNALPFSSSSSAPAPSPKLNSLAATHKLKKDI
HRCHRMSRRPLPRTDPRGPAFSSSSGFQSRMHVSPFSETVRILNRRVKPREVKRGRIILNLKVIDKPGRGGGTAGSRN
VQTGRQNIPSRNRIIGRKGEAPYRPFQPPLKMLGFPMYGKPFGLQCGGPMSFHSQTGSSSTTGPRDINSSSSQRQARP
PSSSSSSTSSKVTAKRPQPAAEASKESKSSKSPETQNASCSEAXKTPVPPSPSPSLEDQDEDAXDCSXAASEGARXPC
QHKGPSTVSSVAPEQSSSPLEPKRVPAEGDPDWHPEMAPSCKDVVVTDVTTNLVTVTIKEFPSPASGPASPSASIENA
STPPPAAASEDSSVLKP

>Pre_LOC103482006
MELSAIGEQVFAVESILKKRLRKGNVEYLLKWKGWPPKYSTWEPEEHILDRRLVQAFEDKEERDRVIGHRRKGAKSKR
LLLQNTVYTMDLRSAHKIPAKPPPRLRLSLTRSLVPDDEEDEDEEDACVPSRTQAAHRKSKQSRRLSLQRPSQEAWEE
EDEEEEEEEDEKEEEEEEEKKNSAEDSEHREEEEAEMGEGVFIGHRGPDTWSTAVGSCGAAASDKLWQPMVCPGEVTVT
DVTLNALTVTFRESRAARGFFRDWGLEVWEQVEKVRHGGFLYLWLG

>Pre_LOC103468464
MELSSIGEQVFAVESITKKRIRKGNVEYLLKWQGWPPEYSTWEPEDNILDPLLVLAYEENQEKLRSLSFRKKGLRPRK
LVLRNIFGMDLRSAHKDDEKPPPRLRLSLTRSMSTDVEQACRRRARRRNRQRVTKAFQKHSSNKPIHEQKMNVEPVEK
DWGGTSEEDKPGCESTTEERCEDSLYGQSECSSPPFLEEQDMDTEVEEKVASPDMWTDEADGGIFETSQRHTNSHDES
XDNXLVSEAKPEVVVSPSGRSDWVEEEGVEAFSESPTTEEKNATSVIVRVQGCGKXTGDAVSVCSDSEPKKDEARSDN
QSVIVTASDRPKVPYEAPRPGRVIVTNVTVNCLTVTFKEASCSEGFFNGC

>Pre_Cbx8 (Gene ID: 103463651)
MELSAVGERVFAAESIIKRRIRKGRIEYLVKWKGWSPKYSTWEPEENILDSRLFAAFEQREREREELYGPKKRGPKPKT
FLLKAQAKGKNNSYEFRSEAVRGMHVTYPTPEPVFTPRAREGLRAVVPTIFPPSAVNRGESMRVRPSELSLHDHQRSS
LQQTDSDGHVQTPKKRGPKPKPRFVDDDFSPVISEPRKRGTEEEEEEEEEEEEEEEEDEEEEEEEEEEERRGPHKLSKFQ
GDEETRLQKLDHRHSENHKHHHHHHYYHHHHHTQKVSRGSSHKQLHSHRSMEPHRTKDGSGYLVAATHFKQQHKASRG
RPAGLPPMEKPYFLDRRSPTRFDDSQDQTAWRPSLSNVEKVLVTDVTANFLTVTIKESSTSEGFFKDKR

>Pre_LOC103469230
MELSAVGESVFAAESIIKRRIRRGRWEYLVKWKGWSQKYSTWEPEENILDERLFAAFEERERERELFGPKKRGPKPET
FLLKAKAKAKEKTYEFRREAPRGIQVSYPIPEPVVTPRAREGLRAVVPTIFPPSAVNRGESVQIRPPEPERRPRPAPL
EALLDQESTRSPKKRGRKPKLHPHYDPHDASGSSEPAPKRSRSLEEQSVMSRRLHHHGETSDHSLLQLTRRFQEETTI
TPKSSSEQKHAGLSYACAFSPGVRKRDPEGHRTYSLSRLHFPRHGKLKRSAEERRHQASQADQTAPSAPTWTPSFTNW
DTVTVTDVTMNLLTVTIRESSTAKGFFTDKR

>Xco_LOC114160441
MEELSAVGEQVFDAECILNKRLRKGKLEFLVKWRGWSSKHNSWEPQENILDPRLLAAFNKKEQEKELLMLKRRKRPRG
RPRKIIENIPEPQKSSTSSSASSSSSSDSSSSCSSSSSSSEDEDEGDDGHAKQASPTVRPRELHPVPQKKAQIVMAKQ
EPLRKRNRKPLPPEAKEFQQNKGPRKVLKTARETDLPGAIKKPVHPASFTFMGFHRSSAKDAAPGPYRSPLTQGGAVK
PATSSPGSGRSSVQTASLALNKSNQIRNATEGKLSISGGNSGGSLDLKTAACKSKGVAALNVNTTKHPVQGTTQHTLS
SPSGPKKPHSTGSAMQRVAGTKAGASPSKSSSSNQGHQPLNLQNKQSQSGDAPGTTAASGLRNPVIPARKPTVAQNLE
TNANKSPVSSGRPPARKPQAGGDKLREMKEFQKVQGRLDKSCSEIQNPPARTATKGSKKAKMTDMSTGEEESSSDSDH
DSSYAGQGHPVAGQNQDWKPTRSLIEHVFVTDVTANLVTVTVKESPTSVGFFSIRNY

>Xco_LOC114152221
MEGVTVGQVFDAECILSKRQRKGKFEYLVKWRGWSSKHNSWEPEENILDPRLLAAFHKREQERELLFQKKGKRPRGRP
RKILPPPADTKDSHSSSSSFGLSSEPSSSSEEDDDDHVKKAKQSPRIHPVPQKRPQILLAKPDPPHKKKRGRKPLHPD
LKALRQGKSLPSPPRHHQPIRSPRDEPRPGVKKPLQPASFTYPGLSRSSREEGASSFSQSSTSKPGSLNCVWAGRSLS
ASSSHNKGSPSQKPSELRRAVSETGSVRADAFKGSAQKPAPLGLHSSFGGHPIAAQRPPLTHRRHDNVGGQSGLVQHK
QQNSSFSKQSPSPTPRERTSQALNLRALNLQSVGKLPGSGGLQGGSRSTGVSKGTTAGGQRSGPGAAEPGRSRTQPAG

GSRQEEHKHGSSSQNRNLNELSTGDSDDTSSSESEHDPSSFPGDTRPSVSSNTAESDTETDWRPARSLMEHVFVTDVT
ANFITVTVKESPTSVGFFNSRNH

>Xco_Cbx4 (Gene ID: 114145263)
MELPAAGEHVFAVESIEKKRSRKGRVEYLVKWRGWSPRYNTWEPEENILDPRLLDAFQDRERQEQLMGYRKRGPKPKH
LLVQVPSFARRSNILANLQEASLEGNSCQKSSSIQMICPQAPQFQLNSKKLHHYQPLIRECDPEQQSNGKKFFYELNS
KKHHHYQPDLKQHEPVFAKPRDVKAPELSNKGYNLPAVLQQKWVRDKDSGCLTKVKDITMELKKIPADLKGQKEPERV
KPKEDASTHSNGVCSSKLKIVKNKNKNGRIVIVMSKYMENGMQTAKIKSSDSEGAERPLQGAESSMEGRLAEMELVKK
LSLINGFSKQPKDNPTVPTSGIKADCCKEKELSPQTERTVMEQDKGAEGQLPQDKPLQLTNKPDELPLPSEREVPDKK
GIQNGHHGLKRHLSDTDAEEQGLGKRFLSYRSISAPNTDSSPSQSLIVDRNEQQSPALLHDYGYADQEEPMDLSIVKS
RPTSLVCVGTEPETRAEEPAHPLDDSRTQADTQTESQTETHNLSEEESADIVSDSNHQARKNEAFPSFQPCLGNIVIT
DITANCLTVTFKEYVTA

>Xco_LOC114160311
MELPAAGEHVFAVEGIEKKRIRKGKIEYLVKWRGWSPKYNTWEPEENILDPRLLVAFQHRERQEQMMGYRKRGPKPKH
LLLQVPSFARRSNIPAGFEETVQDTEGTFKLDPVQRPQLQHYQLNSKKHHQYQPNSQEIPADSLNNSKKKFIYQLNSK
KHHHYEPDPHMYDAQVSRLKEVVKVQEPASKLANPGWNLPLALQQKWIRNKDTGCLSKVKELAVEVRKPVKDAQSENA
LKPNPKEATLPSAVSSKMKIIKNKNNNGRIVIVMSKYMDGNKVHGAKDKHRESSRDEKTQSSKTQENNPPPGAKMTEH
PENGIPKEICKGSSVSVAEHPLKCSPKDRHFSKPSPSTAEEYNTEVARGQADLPDDLPLQLTASSPPTSWTADTAIPT
PTAVDQIRIPSFPCDRKRKLSDPVEDRNLGKILLASRSLSVPTAAVTPPQDKPMDLHCSVARRSSMCTMYDANGSQEE
PMDLSCPKNRRQVEAEVQPPVEAEPVVEDTPPPKENKQESCEKSPEEPANKISPFMGNIIITDITTNRLTVTFKEYVS
F

>Xco_Cbx6 (Gene ID: 114145658)
MELSAAGDRIFAAEAILKRRVRKGRLEYLVKWKGWAMKHSTWEPEENILDDRLLLGFEQKEREREMHGPKKRGPKPKN
VVAKGRGQKAEPTSRTSSSRQNTPRSSSTASSRASSSSSAVPNALPFSSSSSAPAPSPKLNSLAATHKLKKDIHRCHR
MSRRPLPRTDPTRSAFSSSSGFQSRMHVSPFSETVRILNRRVKPREVKRGRIILNLKVIDKPGRGGGGAAGSRNVQTG
RQNIPSRNRIIGRKGEAPYRPFQPPLKMLGFPMYGKPFGLQCGGPMSFHSQTGSSSTTGARHSNSSSSQRRALPPSSS
SSSTSTNVTAKHPQPATEASKESKSSKSPETQNASCSEANKILVPPSPSPSLEDQDEDAMDCSEASEEARSPCQHKAP
FTVSSVAPEQSSPHPESKRVPAEGDPDWHPEMAPSCKDVVVTDVTTNLVTVTIKEFPSPASGPASPSASVENGSTPPP
AATSEDSSAPKP

>Xco_LOC114160060
MELSSIGEQVFAVESITKKRIRKGTVEYLLKWQGWPTEYSTWEPEDNILDPLLVLAYEENQEKLRSLSFRKKGLRPRK
LVLRNIFGMDLRSAHKDDEKPPPRLRLSLTRSMSTDVEQACRRRRRARRRNRQRVTKAFPKHSSNKPIHEQKMKVAPV
EKDWGGTSEEDKPGCESTTEERREDSLYGQSECSSPPFLEEQDMDTEVEEKAASPDMWTDEADGRIFETSQRDTNSQN
ESEDNTLVSEAKPEVVVSPSGRSDWVKEVGVEAFSESPTMEKKNATSVIVRVQECGKTTDDAVSVCSDSEPQKDEVRS
DNQSVIVTASDRPKVPYEAPRPGKVIVTNVTVNCLTVTFKEASCSDGFFNGC

>Xco_LOC114151814
MELSAIGEQVFAVESILKKRLRKGNVEYLLKWKGWPPKYSTWEPEEHILDRRLVQAFEDKEEKDRVIGHRRKGSKSKR
LLLQNTVYTMDLRSAHKIPAKPPPRLHLSLTRSLVPDDEEDEDGACVPSRTHRKSKQSRRLSLNLQGPSQEAWEEEEE
EENSAEDSEHREEEEAEMGEGVFNGHRGPDNWSTAVGSGGSDKLWRPMVAPGEVTVTDVTLNALTVTFRESRAARGFF
RDWGLEVWEQVGDGAPQSCWRH

>Xco_Cbx8 (Gene ID: 114145391)
MELSAVGERVFAAESIIKRRIRKGRIEYLVKWKGWSPKYSTWEPEENILDSRLFAAFEQREREQELYGPKKRGPKPKT
FLLKAQAKGKTKSYEFRSEAVRGMHITYPTPEPVFTPRAREGLRAVIPTIFPPSAVNRGESMRVRPSELSLHGHQRSS
LQHTDSDGTVQAPKKRGPKPKPRFVDGDFSPVVSEPHKRRAEEEEEEEEEEEEERHGPHKLSKFQGDEEMRLQKLAHRH
SENHKHHQHHHYYHHHHHHTQKVSQLHSHRSLEPHRTTDGSSYLVAAHLKHHHKASRGPPTEFPPMEKPYFLDRQSPT
RFDDSQDQTAWRPCLSNVEKVLVTDVTANFLTVTIKESSTSKGFFKDKR

>Xco_LOC114160318
MELSAVGESVFAAESIIKRRIRRGRWEYLVKWKGWSQKYSTWEPEENILDERLFAAFEERERERELFGPKKRGPKPET
FLLKAKAKAKEKTYEFRREAPRGIQVSYPIPEHVVTPRAREGLRAVVPTIFPPSAVNRGESVPIRPPEPERRPRPVPL
EALLDQESRGSPKKRGRKPKLHPHYDPHDASGSSEPAPKRSRSLEEQSIMSRRLHHHGETSDHSLLQLTKRFQEETTI
TPKSNSEQKHAGLSYTCAFSPGERKRDPEGHRTYSLSRLDFPQHGKLKRSAEERGHQAAVLSSQADPTAPSAPTWTPC
YTNWDTVTVTDVTMNLLTVTIRESSTAKGFFRD

>Pla_LOC106937812
MEGVTVGQVFDAECILSKRQRKGKFEYLVKWRGWSSKQNSWEPEENILDPRLLAAFHKREQERELLFQKKGKRPRGRP
RKILPPPTDTKDSHSSSSSFGLSSEPSSSSEEDDDDDHVKKAKQSPRIHPVPQKRPQILLAKPDPPRKKKRGRKPLHP
DLKALRQGKSLPSPPRHHQPIRSPRDEPRPGVKKPLQPASFTYPGLSRSSREEGASSFSQGSTSKPGSLNCVWAGRSL
SASSSHNKGSPSQKPSELRRTVSETGSVRADAFKGSAQKPAPLGLHSSFGGHPIAAQRPPLTHRRQDSVGGQSGLVQH
KQQNSSFSKQLPSPTPRERTSQALNLRALNLQSVGKLPGSGGLQGGSRSTGVGKGTTAGGQRSGPGVTEAGRSRTQPA
GGSRQEEHKHGPSSQNRNLNELSTGDSDDTSSSESEHDPSSFPDDTRPSVSSNAAESDTETDWRPARSLMEHVFVTDV
TANFITVTVKESPTSVGFFNSRNH

>Pla_LOC106943510
MEELSAVGEQVFDAECILNKRLRKGKLEFLVKWRGWSSKHNSWEPQENILDPRLLAAFNKKEQEKELLMLKRRKRPRG
RPRKIIENIPEPQKSSTSSSASSSSSSDSSSSCSSSSSSSEDEDEGDDSRAKQASPTVRPRELHPVPQKKAQIVMAKQ
EPQRKRNRKPLPPEAKEFQQNKGPRKVLKTTKETDLPGAIKKPVHPASFTFMGFHRSSAKDAAAGPYRSPLTQGGAVK
PAISSPGSGRSSVQTASLALSKSNQSRNATEGKLSIGNSGGSLDLKTAACKSKGVAALNVNTTKHPVQGTTQHTLSPP
SGPKKPQSTGSAMQRVAGTKAGASQSSKNTSSSQGHQPLNLQSKQSQSGDAPGSGTTAASGLRSPVIPARKPTVAQNL
EANANKSPVSSGRPPARKPQAGGDKLREMKEFQKVQGRLDKSCSEIQNPPARTATKGSKKAKMTDMSTGEEESSSDSD
HDSSYAGQGHPVAGQNQDWKPTRSLIEHVFVTDVTANLVTVTVKESPTSVGFFSIRNY

>Pla_Cbx4 (Gene ID: 106958633)
MELPAAGEHVFAVESIEKKRSRKGRVEYLVKWRGWSPRYNTWEPEENILDPRLLDAFQDRERQEQLMGYRKRGPKPKH
LLVQVPSFARRSNILADLQEASLEGNSCQKSSSIQMMCPQAQQFQLNSKKLHQYQPLIRECDAEQQSNGKKFFYELNS
KKHHHYQPDLKQHEAVFAKPRDVKAPELSNKGYNLPAVLQQKWVRDKDSGCLTKVKDITMELKKIPPADLKGQKEPER
VKPKEDASTHSNGVSSSKLKIVKNKNKNGRIVIVMSKYMENGMQTAKIKSSDSEGAERQGAENSVERHLAKMKLVKKL
GLINGFSKQPKDNPTVPSSGIKADCCREKELSPQTERTAMERNKGAEGQLPQDKPLQLTNKPDELPLPSEREVPDKTG
IQNGHHGLKRHLSDTDAAEQGLGKRFLSCRSISAPNTDSSPSQSLTVDRNEQQSPALLHDYGYADQEEPMDLSIVKSR
PTSSVCVETEPETRAEEPAHPPDDSRTQTDTQTESQTETHNPSEEEESADIVPDSNHQARKDEAFPSFQACLGNIVIT
DITANCLTVTFKEYVTA

>Pla_LOC106957183
MELPAAGEHVFAVEGIEKKRIRKGKIEYLVKWRGWSPKYNTWEPEENILDPRLLVAFQHRERQEQMMGYRKRGPKPKH
LLLQVPSFARRSNIPAGLEETVQDAEGTFKLDPVQRPQPQHYQLNSKKHHQYQPNSQEIPADALNNSKKKFIYQLNSK
KHHHYEPDPYMYDAQVSRLKEVVKVQETANKLANPGWNLPLALQQKWIRNKDTGCLSKVKELAVEVRKPVKDAQRENA
LKPNPKEATLPSSVSSKMKIIKNKNKNGRIVIVMSKYMDGNKVHGAKDKHRESSREEKTQSSKTQENNPPPGAKMTEH
PENGIPKEICKGSSVSVAEHPLKCSPKDRHFSKPSPSTAEEYNTEVARGQADLPDDLPLQLTASSPPTSWTADTAIPT
PTAVNQIRIPSFPCDRKRKLSDPVEDRNVGKILLASRSLSVPTAAVTPPQDKPMDLHCSVARHSSTCATYDANGSQEE
PMDLSCPKNRRRVEAEVQPPVEAEPAVEDTPPVKDNTQKSCEKSPEEPAKKISPFMGNIIITDITTNSLTVTFKEYVS
F

>Pla_Cbx6 (Gene ID: 106936647)
MELSAAGDRIFAAEAILKRRVRKGRLEYLVKWKGWAMKHSTWEPEENILDDRLILGFEQKEREREMHGPKKRGPKPKN
FVAKGRGQKAEPTSRASSSRQSTARSSSTTSSRASSSSSSSSSSAAPNALPFSSSSSSSVPAPSPKLNSLAATHKLKK
DIHRCHRMSRRPLPRTDPTRPAFSSSSGFQSRMHVSPFSETVRILNRRVKPREVKRGRIILNLKVIDKPGRGGGAAGS
RNVQTGRQNIPSRNRIIGRKGEAPYRPFQPPLKMLGFPMYGKPFGLQCGGPMSFHSQMGSSSTTGPRDSNSSSSQRQA
PPPSSSSSSTSSNVTAKRPQSAAEASKESKSSKSPETQNASCSEANKTPVPPSPSPSLEDQDEDAMDCSEASEGARSP
CQHKAPSTVSSVAPEQSSPPLEPKRVPTEGDPDWHPEMAPSCKDVVVTDVTTNLVTVTIKEFPSPASGPASPSASIEN
ASTPPPAAASEDSSVPKP

>Pla_LOC106961268
MELSAIGEQVFAVESILKKRLRKGNVEYLLKWKGWPPKYSTWEPEEHILDRRLVQAFEDKEERDRVIGHRRKGSKSKR
LLLQNTVYTMDLRSAHKIPAKPPPRLRLSLTRSLVPDDEEDEDEDEDEEDACVPSRRQATHRKSKQSRRLSLNLQRPS
QEAWEEEEEDEEEEEEEEEEEEEEEEKNSAEDSEHLSQPKTTNHSQKEKEEAKMGEGVFNGHRGLDNWSTAVGSGGVDT
SDKLWRPLVCPGEVTVTDVTLNALTVTFRESRAARGFFQDWGLEVWEQVGDGAPRSCWRH

>Pla_LOC106961608
MELSSIGEQVFAVESITKKRIRKGNVEYLLKWQGWPPEYSTWEPEDNILDPLLVLAYEENQEKLRSLSFRKKGLRPRK
LVLRNIFGMDLRSAHKDDEKPPPRLRLSLTRSMSTDVEQACRRRARRRNRQRVTKAFPKHSSNKPIHEQKMKIEPVEK
DWGGTSEEDKPGCESTTEERCEDSLYGQSECSSPPFLEEQDMDTEVEEKAASPDMWTDEADGGIFETSQGHTNSHDES
EDNTLVSEAKPEVMVSPSGKSDWVEEEGVEAFSESPTMEKKNATSVIVRVQGCGKTMGDAVSVCSDSEPKKDEVRTDN
QSVIVTASDRPKVPYEAPRPGRVIVTNVTVNCLTVTFKEASCSEGFFNGC

>Pla_LOC106957191
MELSAVGESVFAAESIIKRRIRRGRWEYLVKWKGWSQKYSTWEPEENILDERLFAAFEERERERELFGPKKRGPKPET
FLLKAKAKAKEKTYEFRREAPRGIQVSYPIPEPVVTPRAREGLRAVVPTIFPPSAVNRGESVPIRPPEPERRPRPAPL
KALLDQESTRSPKKRGRKPKLHPHYDPHDASGSSEPAPKRSRSLEEQSVMSRRLHHHGETSDHGLLQLTKRFQEETTI
TPKSSGEQKHAGLSYACAFSPGVRKRDPEGHRTYSLSRLHFPQHGKLQRNAEERRHQAAVLSSEADPTAPSAPTWTPC
FTNWDTVTVTDVTMNLLTVTIRESSTAKGFFRDKR

>Pla_LOC106958634
MELSAVGERVFAAESIIKRRIRKGRIEYLVKWKGWSPKYSTWEPEENILDARLFAAFEQREREREELYGPKKRGPKPKT
FLLKAQAKGKTKSYEFRSEALRGMHVTYPTPEPVFTPRAREGLRAVVPTIFPPSAVNRGESMRVRPSELSLHEHQRSS
LQQTDSDGHVQTPKKRGPKPKPRFVDDNFSPVASKARKRGTEEEEEEEEEEEEEEEDEEEEEETRGPHKLSKFQGDEE
MRLQKLAHRHSENHKHHHHHHYYHHHHHHTQKVSRGSSHKQLHSHRSMEPHRTKDGSGYLVAHFKQPHKASRGRPTEL
PPMEKPYFLDRRSPTRLDDSQDQTAWRPCLSNVEKVLVTDVTANFLTVTIKESSTSKGFFKDKR

>Pme_LOC106924748

MEGVTVGQVFDAECILSKRQRKGKFEYLVKWRGWSSKHNSWEPEENILDPRLLAAFHKREQERELLFQKKGKRPRGRP
RKILPPPTDTKDSHSSSSSFGLSSEPSSSSEEDDDDHVKKAKQSPRIHPVPQKRPQILLAKPDPPRKKKRGRKPLHP
DLKALRQGKSLPSPPRHHQPIRSPRDEPRPGVKKPLQPASFTYPGLSRSSREEGASSFSQGSTSKPGSLNCVWAGRSL
SASSSHNKGSPSQKPSELRRAVSETGSVRADAFKGSAQKPAPLGLHSSFGGHPIAAQRPPLTHRRQDSVGGQSTGLVQ
HKQQNSSFSKQLPSPTPRERTSQALNLRALNLQSVGKLPGSGGLQGGSRSTGVGKGTTAGGQRSGPGATEAGRSRTQP
AGGSRQEEHKHGPSSQNRNLNELSTGDSDDTSSSESEHDPSSFPDDTRPSVSSNAAESDTETDWRPARSLMEHVFVTD
VTANFITVTVKESPTSVGFFNSRNH

>Pme_LOC106914323
MEELSAVGEQVFDAECILNKRLRKGKLEFLVKWRGWSSKHNSWEPQENILDPRLLAAFNKKEQEKELLMLKRRKRPRG
RPRKIIENIPEPQKSSTSSSASSSSSSDSSSSCSSSSSSSEDEDEGDDSRAKQASPTVRPRELHPVPQKKAQIVMAKQ
EPPRKRNRKPLPPEAKEFQQNKGPRKVLKTAKETDLPGAIKKPVHPASFTFMGFHRSSAKDAAAGPYRSPLTQGGAVK
PAISSPGSGRSSVQTASLALSKSNQSRNATEGKLSIGNSGGSLDLKTAACKSKGVAALNVNTTKHPVQGTTQHTLSPP
SGPKKPQSTGSAMQRVAGTKAGTSQSSKNTSSSQGHQPLNLQSKQSQSGDAPGSGTTAASGLRSPVIPARKPTVAQNL
EANANKSPVSSGRPPARKPQAGGDKLREMKEFQKVQGRLDKSCSEIQNPPARTATKGSKKAKMTDMSTGEEESSSDSD
HDSSYAGQGHPVAGQNQDWKPTRSLIEHVFVTDVTANLVTVTVKESPTSVGFFSIRNY

>Pme_Cbx4 (Gene ID: 106928181)
MELPAAGEHVFAVESIEKKRSRKGRVEYLVKWRGWSPRYNTWEPEENILDPRLLDAFQDRERQEQLMGYRKRGPKPKH
LLVQVPSFARRSNILADLQEASLEGNSCQKSSSIQMMCPQAQQFQLNSKKLHQYQPLIRECDAEQQSNGKKFFYELNS
KKHHHYQPNLKQHEAVFAKPRDVKAPELSNKGYNLPAVLQQKWVRDKDSGCLTKVKDITMELKKIPPADLKGQKEPER
VKPKEDASTHSNGVSSSKLKIVKNKNKNGRIVIVMSKYMENGMQTAKIKSSDSEGAERQGAESSVERHLAKMKLVKKL
GLINGFSKQPKDNPTVPSSGIKADCCKEKELSPQTERTAMERDKGTEGQLPQDKPLQLTNKPDELPLPSEREVPDKTG
IQNGHHGLKRHLSDTDAAEQGLGKRFLSCRSISAPNTDSSPSQSLTVDRNEQQSPALLHDYGYADQEEPMDLSIVKSR
PTSSVCVETEPETRAEEPAHPPDDSRTQTDTQTESQTETHNPSEEEESADIVPDSNHQGRKDEAFPSFQACLGNIVIT
DITANCLTVTFKEYVTA

>Pme_LOC106904416
MELPAAGEHVFAVEGIEKKRIRKGKIEYLVKWRGWSPKYNTWEPEENILDPRLLVAFQHRERQEQMMGYRKRGPKPKH
LLLQVPSFARRSNIPAGLEETVQDAEGTFKSDPVQRPQPQHYQLNSKKHHQYQPNSQEIPADALNNSKKKFIYQLNSK
KHHHYEPDPHMYDAQVSRLKEVVKVQETANKLANPGWNLPLALQQKWIRNKDTGCLSKVKELAVEVRKPVKDAQRENA
LKPNPKEATLPSAVSSKMKIIKNKNKNGRIVIVMSKYMDGNKVHGAKDKHRESSREEKTQSSKTQENNLPPGAKMTEH
PENGIPKEICKGSSVSVAEHPLKCSPKDRHFSKPSPSTAEEYNTEVARGQADLPDDLPLQLTASSPPTSWTADTAIPT
PTAVDQIRIPSFPCDRKRKLSDPVEDRNVGKILLASRSLSMPTAAVTPPQDKPMDLHCSVARHSSTCATYDANGSQEE
PMDLSCPKNRRRVEAEVQPPVEAEPAVEDTPPVKDNTQTSCEKSPEEPAKKISPFMGNIIITDITTNSLTVTFKEYVS
F

>Pme_Cbx6 (Gene ID: 106904597)
MELSAAGDRIFAAEAILKRRVRKGRLEYLVKWKGWAMKHSTWEPEENILDDRLILGFEQKEREREMHGPKKRGPKPKN
FVAKGRSQKAEPTSRASSSRQSTPRSSSTTSSRASSSSSSSSSSAAPNALPFSSSSSSSAPAPSPKLNSLAATHKLKK
DIHRCHRMSRRPLPRTDPTRPAFSSSSGFQSRMHVSPFSETVRILNRRVKPREVKRGRIILNLKVIDKPGRGGGAAGS
RNVQTGRQNIPSRNRIIGRKGEAPYRPFQPPLKMLGFPMYGKPFGLQCGGPMSFHSQTGSSSTTGPRDSNSSSSQRQA
PPPFSSSSSTSSNVTAKRPQPAAEASKESKSSKSPETQNASCSEANKTPVPPSPSPSLEDQDEDAMDCSEASEGARSP
CQHKAPSTVSSVAPEQSSPPLEPKRVPTEGDPDWHPEMAPSCKDVVVTDVTTNLVTVTIKEFPSPASGPASPSASIEN
ASTPPPAAASEDSSVPKP

>Pme_LOC106910083
MELSAIGEQVFAVESILKKRLRKGNVEYLLKWKGWPPKYSTWEPEEHILDRRLVQAFEDKEERDRVIGHRRKGAKTKR
LLLQNTVYTMDLRSAHKIPAKPPPRLRLSLTRSLVPDDEEDEDEDEEDACVPSRTQAAHRKSKQSRRLSLNLQRPSQE
AWEEEEEDEEEEEEEEEEEEEKNSAEDSEHLSQPKTTNHSQKEKEEAKMGEGVFNGHRGLDNWSTAVGSGGADTSDKL
WRPLVCPGEVTVTDVTLNALTVTFRESRAARGFFQDWGLEVWEQVGDGAPRSCWRH

>Pme_LOC106933457
MELSSIGEQVFAVESITKKRIRKGNVEYLLKWQGWPPEYSTWEPEDNILDPLLVLAYEENQEKLRSLSFRKKGLRPRK
LVLRNIFGMDLRSAHKDDEKPPPRLRLSLTRSMSTDVEQACRRRARRRNRQRVTKAFPKHSSNKPIHEQKMKIEPVEK
DWGGTSEEDKPGCESTTEERCEDSLYGQSECSSPAFLEEQDMDMEMEEKAASPDMWTDEADGGIFETSQGDTNSHDES
EDNTLVSEAKPEVMVSPSGKSDWVEEEGVEAFSESPTMEKKNATSVIVRVQGCGKTMGDAVSVCSDSEPKKDEARTDN
QSVIVTAPDRPKVPYEAPRPGRVIVTNVTVNCLTVIFKEASCSEGFFNGC

>Pme_LOC106904421
MELSAVGESVFAAESIIKRRIRRGRWEYLVKWKGWSQKYSTWEPEENILDERLFAAFEERERERELFGPKKRGPKPET
FLLKAKAKAKEKTYEFRREAPRGIQVSYPIPEPVVTPRAREGLRAVVPTIFPPSAVNRGESVPIRPPEPERRPRPAPL
EALLDQESTRSPKKRGRKPKLHPHYDPHDASGSSEPAPKRSRSLEEQSVMSRRLHHHGETSDHGLLQLTKRFQEETTI
TPKSSGEQKHAGLSYACAFSPGVRKRDPEGHRTYSLSRLHFPQHGKLQRNAEEQRHQAAVLSSQADPTAPSAPTWTPC
FTNWDTVTVTDVTMNLLTVTIRESSTAKGFFRDKR

>Pme_LOC106928182

MELSAVGERVFAAESIIKRRIRKGRIEYLVKWKGWSPKYSTWEPEENILDSRLFAAFEQRERERELYGPKKRGPKPKT
FLLKAQAKGKTKSYEFRSEAVRGMHVTYPTPEPVFTPRAREGLRAVVPTIFPPSAVNRGESMRVRPSELSLHEHQRSS
LQQTDSDGHVQTPKKRGPKPKPRFVDDNFSPVVSEPRKRGTEEEEEEEEEEEEEEEEEEEEEETRGPHKLSKFQGDEEM
RLQKLAHRHSENHKRHHHHHYYHHHHHTQKVSRGSSHKQLHSHRSMEPHRTKDGSGYLVAHFKQPHKASRGRPTELPP
MEKPYFLDRRSPTRLDDSQDQTAWRPCLSNVEKVLVTDVTANFLTVTIKESSTSKGFFKDKR

>Nfu_Cbx2 (Gene ID: 107397219)
MEELSAVGEQVFDAECILNKRTRKGKLEFLVKWRGWSSKHNSWEPQENILDPRLLVAFNKKEQEKELLMLKRGKRPRG
RPRKILENIPDPSKSSSSSPSSSSSTDSSSSCSSSSSSSDDDDDDDNDSHVKQSSPTVRTRELHPVPQKKAQIVMKQD
SSKKRSRKLLSPEVKEFQLNKSPHKVLKTIKDLDLPGAIKKPVHPSSFTFMGFHRGTLGAQYRSPLNQGGAIKTPMSS
AGPGRLTGQTSSLPLNKSNQNRNVTEGKLSVPSMNTGASLDLKTAASKSKGVAAFNLNTSKHPIQGTPQHTLSPPNGQ
KKPQAPVSTMQRIPSSKAGASLPSKNNPSVQQPSPQPLNLQNKHSQVSDSPGNGSTPGSGVRNPPNPTRKSTVAQNQE
ANLPKSPVTSGRPPVRKPQPGADRAREVNEIQTAKVQGRLDKSCTEIHNQQERIATKGSKKAKMTEMSTGEEESSSDS
DPDSSYPGHCHSVVSQNQDWKPTRSLIEHVFVTDVTANLVTVTVKESPTSVGFFSIRNY

>Nfu_LOC107387235
MEGVTVGQVFDAECILSKRQRKGKFEYLVKWSGWSSKHNSWEPEENILDPRLLAAFHKREQERELLFQKKGKRPRGRP
RKIVPPPTSTKDSRSSSSSSGLSSSPSSSSEEEEDDDDDDDDHVKKVRPSSRLHPVPQKRPQILLAKPDPPYKKKRGR
KPLHPDLKALRQTKSPSLPPPLSPPPPRHHQPIRLPRDDPRLGVKKPLQPASFTYTGLSRSSRDEGSSTSQSSSSSFS
PSATSKPGSLNCVWINRSLSTSSHNKVSLSHKSSELRRSISETGSSRSSLKAGGAASPGLSSFGGGPAALQHPQMGQR
RQEGVGCHGGHVQHKQQNSSFTKPSSSSTSRERTNQALNLRALNLQSVSKTSNSSSLQGAASSRTSSIVGKGSAAGAQ
RSSLTAGGAAETGRLGRQEERKHGLSSQNRSLNELSTGDSDDTSSSESERDPSLFPSDSRPSLGDNATESDTETDWRP
ARSLLEHVFVTDVTANFITVTVKESPTSVGFFTSRNH

>Nfu_LOC107376532
MELPAAGEHVFAVEGIEKKRIRKGKIEYLVKWRGWSPKYNTWEPEENILDPRLLVSFQHRERQEQLMGYRKRGPKPKH
LLLQVPSFARRSSIPAGFEDTSQDIEATIKPDPIQVQRSQPQQYQLNSKKHHPYQPGNAEVPADQLANSKKKFIYQLN
SKKHHHYEPDPHMYDTQASRLKEVVKVQEPASKPTNPGWNLPLALQQKWIRDKDTGCLSKVKELAVEVRKPAVKEAQS
ENALKPNPKEATLPTAVSSKMKIIKNKNKNGRIVIVMSKYMDSNKVHGAKDKHRDSSKEDKAQSTKPSDHLESSIPKE
ICNSRPISVAEHPLKCSPKDRHFSKPSPSTAEEYNTEVARGQADLPEDMPLQLTASSPPTSWTADTNIPMPTAVDQIR
IPSFPGDRKRKLSDPVEDRSKVFLASRSLSVPCSAVAPPQDKPMDLHCSGPRDSRSPTCEAEGSQDEPMDLSCHKNRK
QVEAETQPQVDPQPTVKDTPPVHEDTQEPTDTSKEQENKKLSPFMGNIIITDITTNSLTITFKEYVPF

>Nfu_LOC107395725
MELPAAGEHVFAVESIEKKRSRKGRVEYLVKWRGWSPRYNTWEPEENILDPRLLDAFEDRERQEQLMGYRKRGPKPKH
VLVQVPSFARRSTILADLQESSLEDDNCQKSSPIQMICPQVQQYQLNSKKHHQYQPLCRESEQKNNGKKFYYQLNSKK
HHHYQPDLKVHEPVFTKPQEVKAPELVNKGYNLPPALQQKWVRDKDSGCLTKVKDITMELKKLPADLNGHKEPEKVEA
TEDASPQSNGVRSSKLKIVKNKNKNGRIVIVMSKYMENGMQAAAATNGDAAEKSSQRADSSIENHLAKMKLVKKLGLV
NGFVKHSKDKPAVLGSGLNGDGLKEKEPSPLVGRTETEQEKPEVRGQGKFLEDQPLQLTSSGASDERENRAGSGGLKR
PVGDLGGEDRDAKRFLSCRSISVPNAVPSPPLSVSTSQNGLQTPAGLQDCGYVDQEEPMDLSMVKSRSSVCSATATEM
QTEAVTQTEPQTETHNASEDSSNHRTTKDETFPSFQPCLGNIVITDITTNCLTVTFKEYVAA

>Nfu_Cbx6 (Gene ID: 107375179)
MELSAAGDRIFAAEAILKRRVRKGRLEYLVNWKGWAMKHSTWEPEENILDDRLILGFEQKEREREMSGPKKRGPKPKN
LAMKTQTPKGETSSRASSSRQNTPRLSLSTSSRAAFSSAPAPNLPSSSSSSSNMGPTPKLNSLAATHKLKKDIHRCHR
MSRRPLPRSDPMASSYSTSGGFPSRMHVSPFSETVRILNRRVKPREVKRGRIILNLKVIKPSRGSTGAKNITTGRQNI
PSRNRIIGRKGEAPYRPFQPPLKMLGFPMYGKPFGLQCGGPMSFYSHMGSCTSTGARNSHSTSSQYQAPPSPSSSSGS
EGKSCTNTTAKLSPPPAEASPFSKSNKALAETQKNSPARVSSSDVNPGVLASSFLPSSPSSSLEDEEQSSMDHSAPSD
TGKKNPRQHKAKHQSPSVLSPVTPAEQTPTSVEPQRVPAEGDPDWHPEMAPSCKDVVVTDVTTNLVTVTIKEFPTPAS
GPSSPSAGLENASSPAPVTVPEEPSSAKP

>Nfu_LOC107388224
MELSAIGEQVFAVESIVKKRVRKGNVEYLLKWKGWPPKYSTWEPEEHILDRRLVQAFEEKEEKDRVVGHRKKGAKAKK
LLLQSTVYTMDLRSAHKIPSKPSPRLRLSLTRSLDHDDNDDETWVACRLRSRTVHHKSKKTRSRNRCFDSTPSSSSQE
DWDEEEEEDEQEEKQTSESILNGQDVTDSWNTAAQTDTNAASEKPWRPIAGPGEVTVTDVTLNSLTVTFRESRVAKGF
FRNWGLEVWQQ

>Nfu_LOC107376332
MELSSIGDQVFAVESITKKRVRKGNVEYLLKWQGWPPEYSTWEPEDNILDPLLVLAYEENQEKIRSLAYRKKGLRPRK
LMLRNIFAMDLRSASKDSEKPPPRLRLSLTRSMSTDVEQLCRRPAGRRYRSRMTKLGSKRSSCKPIHLQKKKLDSPLK
HWGEASEEDRPECESAAEEKCEDSCYGHSECSSPPFVERQDLDVDVEKVDDQLTPINTEMWTNTADGGTTGTCQISAH
EQPKDNALASEAKLGDAVVSLGDRLDFDGGETGVEAGSECETGVEAGSECPSVDSIPRERSNTTSVIVRVQDYASTGV
KVEEGSANNQSVTMATSGPAEEERPEKVIVTNVTVNSLTVTFVEADGAEGFFKGC

>Nfu_LOC107395726

MELSAVGERVFAAESIIKRRIRKGRSEYLVKWKGWSHKYSTWEPEENILDSRLFVAFEQRERQRELFGPKKRGPKPKT
FLLKAESQGNTRSYELRNEAIRGMDVTYSCPEPVVTPRAREGLRTVVPTIFPPSAVNQGESICLRASDLSVLEHQRSF
HGDGSDEIHLPRKRGPKPKPLLEDCELHERQAEEPVRRCALQLGEEMGLVRVAHRHPENLSHKHHHHPLSRPVSSRTS
YYPFYSDNSRHTHTNLLDTHRTKDGSRCSAPAHFKHHSKVGPSRPAALPLMEKPYFLDRPSPTRIDADSVEMTWRPSL
SNVEKVLVTDVTTNFLTVTIKESSTSKGFFRDKR

>Nfu_LOC107376533
MELSAVGESVFAAESIIKRRIRRGRWEYLVKWKGWSQKYSTWEPEENILDERLFAAFEERERERELFGPKKRGPKSES
FLLKAKAKVKEKTYEFRRETPRSIQVSYPIPEHVVTPRAREGLRAVVPTIFPPSAVNRGESVPIRAPEPERRPRPPIP
EALDPESMRLPKKRGRKPNLYYDEQDSSPEPCPKRSRFPEESKMSRRMSYHGETSEQSLIQLTRRFQEETTITPKSSS
ERRATSAGLACANAGVRKSDRERIYDLSRIRFSQCDNLKHEERGAEPSPSSATPWTPCFSSLDSVTVTDVTMNFLTVT
IRESSTEKGFFKEKR

>Fhe_Cbx2 (Gene ID: 105932784)
MEELSAVGEQVFDAECILNKRLRKGKLEFLVKWRGWSSKHNSWEPQENILDPRLLAAFNKKEQEKELLMLKRRKRPRG
RPRKIIENIPEPEKSSSSSSASSSSSSDSSSSCSSSSSSSSDDEDEGDDGYVKQASPTVRPRELHPVPQKKAQIVMAKQ
EPPRKRSRKPLPPEGKDFQNKGPRKVPKTAKEVDLPGAIKKPVHPASFTFMGFHRSSGKDPVAGQYRSPLAQGGPAKG
PVGSAGSGRPSVQTSSLPASKSNQSRNAAEGKLSISSVNSGGSLDLKAAASKSKGVAALNLNTSKHPVQGTTQHTLSP
PGGQKKPHSPGSALLRVPGAKGGASQPAKSASSNQGPSLQPLNLQNKQISDAPGGGATAPPGLRNPAVPARKTAVTQN
LETDANKGPATSGRPPARKPQPGGDKLREMNEIQTAKVQARLDKIQSPRDRTATKGSKKAKMTDMSTGEEESSSDSDQ
DSPYAGRAHPAAGQNQDWKPTRSLIEHVFVTDVTANLVTVTVKESPTSVGFFSLRNY

>Fhe_LOC105937668
MEGVTVGQVFDAECILSKRQRKGKFEYLVKWRGWSSKHNSWEPEENILDPRLLAAFHKREQERELLFQKKGKRPRGRP
RKILPPPAATKDSGSSSSSSSSGLSSEPSSSSEEDEEHVKKAKPSPRVHPVPQKRPQILLAKPDPPHKKKRGRKPLHP
DLKALRQAKSLPPPPPPRHHQPIRSSRDDLRPGVKKPLQPASFTYPGLSRSSREEGSSSFSQSSGSKPASLGCIWTDR
SLSASSSSSHSKGGPSQKPSELRRSFSETGSGSRADAFKASLKPGGASAPPGLHGSFGGHAIAAQRPPLAHRRQEGGQ
TGLLQHKQQNSSFSKQSPTPRERASQAANLRALNLQRVSKLPPGAVLQGGPRSGKGAAAGGQRSGPPSGGAGEPGRSR
AQPAAGSRPEERKHGPSSQGRSLNELSTGESDSSSSESERDASPFPDDSRPSGGGRAAESDTETDWRPARSLMEHVFV
TDVTANFITVTVKESPTSVGFFSSRNH

>Fhe_Cbx4 (Gene ID: 105940177)
MELPAAGEHVFAVESIEKKRSRKGRVEYLVKWRGWSPRYNTWEPEENILDPRLLDAFQDRERQEQLMGYRKRGPKPKH
LLVQVPSFARRSNILANLQGASLEDDGCQKSGPIQMIRPQAQQFQLNSRKLHQYQPLSREREAEQQSNGKKFFYELNS
KKHHHYQPDLKLHEPVYPKPRDIKAPELANKGYNLPAVLQQKWVRDKDSGCLTKVKDITMELKKLPADLNGQKGTERA
KPKEDASPQSNGVSGGKLKIVKNKNKNGRIVIVMSKYMENGMQTAKIKNADSDDAERPLQGAESSVERHLAKMKLVKQ
LGLMNGFAKQPKDDPAVPGSGIKGDCCKQKELSLQAERTAKEQDRPTEVGGEGRLPQDQPLQLTNKLDLPSERGAPEK
TGAQNRLHGLKRHLSDADAEEHGCSKRFLSCRSISAPDTVDSPSRTAVVDQNGRQSPALQQDYGYQDQDEPMDLSIVK
SRPAPSACAEPETGTRAEEHAHSHDDTHTHTDTHTESQTETHEPSEEERADSASGSHQQARRDEAFPSFQPCLGNIVI
TDITANCLTVTFKEYISA

>Fhe_LOC105933040
MELPAAGEHVFAVEGIEKKRIRKGKIEYLVKWRGWSPKYNTWEPEENILDPRLLVAFQHRERQEQMLGYRKRGPKPKH
LLLQVPSFARRSSIPGSFEETAQDAEGTLKADPTQTPRSQPQQYQLNSKKHHQYQPSSQEVPADALNNSKKKFIYQLN
SKKHHHYEPDPHMYDAQVSRLKEVVKVQEPPSKLANPGWTLPVALQQKWIRNKDTGCLSKVKELAVEVRKPVKEAPSE
NALKPNPKEATLPSAVSSKMKIIKNKNKNGRIVIVMSKYMDGNKVHGAKDKHRDSPREEKTQSAEPSDSNPTARTKMT
ELPENGIPKEICKGSSLSVAEHPLKCSPKDRHFSKPSPSTAEEYNTEVARGQADLPEDLPLQLTASSPPTSWTADTTI
PTPTAVDQIRIPSFPCDRKRKLSDPVEDRNVGKTYLASRSLSVPGASAAPPQDKPMDLHCGATRPSGTRATYGEDGSQ
EEPMDLSFPRNRRQLEAEAPPQAEAEPAVKDTPPAKENAQTPSEKAPEDPAKKVSPFMGNIIITDITTNSLTVTFKEY
VAF

>Fhe_cbx6a (ENSFHEG00000010219)
MELSAAGDRIFAAEAILKRRVRKGRLEYLVKWKGWAMKHSTWEPEENILDDRLILGFEQKEREREMHGPKKRGPKPKN
FVVKACGSKAEPSSRASSSRQNTSRSSSSTSSRAAPSSSSAAPHTLPFSSSSSAPSPKLNSLAATHKLKKDIHRCHRM
SRRPLPRSHPTGPAFSTPGGFPSRMHVSPFSETVRILNRRVKPREVKRGRIIILNLKVINKPGRGGAAAGSRNGQSGRQ
NIPSRNRIIGRKGEAPYRPFQPPLKMLGFPMYGKPFGLQCGGPMSFHSQTGSCSSAGARASSSSQRQAPPPPSSSSSN
SSNAAAKPPQSDAEASKESKSSKAPPGTQKPPSSASCSEANNKSPAPPSPSSSLEDQDEDAVDSSQASEGGRNSPCQH
KAKHQSASAVPPVDPDQPSAPVEPQRVPAEGDPDWHPEMGPSCKDVVVTDVTTNLVTVTIKEFPSPASGPASPSASVE
SASSPPPAATSEEPSVPKP

>Fhe_LOC105930863
MELSAIGEQVFAVESILKKRLRKGNVEYLLKWKGWPPKYSTWEPEEHILDRRLVQAFEDKEERDRVIGHRRKGAKSKR
LLLQDTVYTMDLRSAHKIPAKPPPRLRLSLTRSLVADEEDDETYVPCAAGSRIQLTHRKSKHRGPQSRCLNGTPPSPS
REDWEEEEEEENSPEESEQRDEEEEQEEAQMEGAEMREGVFNGHRDTDNWSTAAGANAATASEGLWRPIIGPGEVTVTD
VTLNSLTVTFRESRVAKGFFRDWGLEV

>Fhe_LOC105917993

MELSSIGEQVFAVESITKKRIRKGHVEYLLKWQGWPPENSTWEPEDNILDPLLVMAYEENQEKLRSLSFRKKGLRPRK
LVLRNIFGMDLRSAHKDNEKPPPRLRLSLTRSMSTDVEQVCRRPARRRIRQRVTKAFPKHSSNKPIRDQRMEMEPMEK
DWGGTSEEDKHGCESTTEERCGDSLYGQSECSSPQFLEEMDVELEVEEKAVCPDMWPDGGIFGTSPRHTNALEQSEEN
TLAAEAEPGDAVSPGDGSGWGEAKAVEACPECPQEEKKNATSVIVRVQGCGKTTGEAVSVTSDSELKKDEVRSDNQSV
IVTASGRPAAPGEAPHRGKVIVTNVTVNCLTVTFKEAREAEGFFHGC

>Fhe_Cbx8 (Gene ID: 105940179)
MELSAVGERVFAAESIIKRRIRKGRIEYLVKWKGWSPKYSTWEPEENILDSRLFAAFEQREREРELYGPKKRGPKPKT
FLLKAQAKVKTKFYEFRREAVRGMHITFPTPEPVFTPRAREGLRAVVPTIFPPSSVNRGESVRPSEPSPHEHRHPPLQ
QAASDGHAHTPKKRGPKPKPRFVDDGFGPAASEPHRRRAEEGESHGAYRLAQFQEDEETRLQKSAHRHSENHAHVHKH
HHHHQHHYSHRHHHHTQKASSGASYKHFHAQRCADPHRTKDGSAHLAAAHFHKHRPRTGQNPSRAAGPPPTEKPYFLD
RRSPARLEDGQDDATWRPCLSNAEKVLVTDVTANFLTVTIKESSTSQGFFKDSR

>Fhe_LOC105933049
MELSAVGESVFAAESIIKRRIRRGRWEYLVKWKGWSQKYSTWEPEENILDERLFAAFEERERERELFGPKKRGPKPET
FLLKAKAKSKEKTYEFRRETPRGIQVSYPVPEPVITPRAREGLRAVVPTIFPPSAVNRGESVPIRHPEPERRPRPAAS
EAFLDQESVQFPRKRGRKPKLLPHYGPQDASGSAEPASKRSRSLEEQSVVSRRLHHHGETSDHTLLQLTKRFQEETTI
TPKSGSEQRHAGLSYTCAFSPSARKSEGHRTYSLSRMHFPQHGQPRRPEDERRRQAAAESSPPDPFAPSAPTWTPCCT
NWDTVTVTDVTMNLLTVTIRESSTAKGFFKDKR

>Kma_Cbx2 (Gene ID: 108237824)
MEELSAVGEQVFDAECILNKRLRKGKLEFLVKWRGWSSKHNSWEPQENILDPRLLAAFNKKEQEKELLMLKRGKRPRG
RPRKILENVPEPSKSSSSSSASSSSSCDSSSSCSSSSSSSDDDDDESHVKQPSPAVRTRELHPVPQKKAQIVMKQEPQ
KKRSKKPPEAKEFLQSKSPRKILKTTKDPDLPGAIKKPVHPSSFTFMGFHRSSARDVVAAQYRSPVQGGAVKMSSVGP
SRSPVQTPSLPLNRTSQIRSGTEGKLSISSMNSGASLDLKTAACKSKGVAALNLNTSKHPIQGSTQHTLSPPSGHKKP
PAPVSTVQRTPSTKAVASSQGSGPQPLNLQNKKSLGPGPTPASDLRGPSNPARKTTVPQSQESNPPKSPVIPGRLLAR
KAPPVGDKDGNEIQSNKVQGRLDKSCPDIQNQQERIPTKGSKKAKITEMSTGEEESSSDSDPDDSCAGQGHSVSQNQD
WKPTRSLIEHVFVTDVTANLVTVTVKESPTSVGFFSIRNY

>Kma_LOC108249256
MDGVTVGQVFDAECILSKRQRKGKFEYLVKWSGWSSKHNSWEPEENILDPRLLAAFHKREQERELLFQKKGKRPRGRP
RKILPPPAAMKDARSSSSSSGLSSSPSSSSEEEEEEEEEEEEDDDEEHMKKAKPSPRLHPVPQKRPQILLAKPDPPRKK
KRGRKPLHPDLKALRPPLPRHHQPIRSLREDPRPGVKKPLQPASFTYTGLSRTSRDEGTSSPQSSFSQSSASKPGALS
CIWTGRSLSASSSSHKTGPSHRNSELRRSVSETGSGFKGSLKAGGASASLHSSFGGGPTAPQRPPLGPRRQEGAGGPQ
NSTFSSPTPRDRASQALSLRALNLQSVSSSLGGNPDPRGAGIGQRSALTAGGAAEPGRGREAGSRQEERKHGSSSQNR
SLNELSTGDSDETSSSESEHDATMFPDNSRPSLGTSAADSDTETDWRPARSLLEHVFVTDVTANFITVTVKESPTSVG
FFSSRNH

>Kma_Cbx4 (Gene ID: 108245485)
MELPAAGEHVFAVESIEKKRSRKGRVEYLVKWRGWSPRYNTWEPEENILDPRLLDAFQDRERQEQLMGYRKRGPKPKH
LLVQVPSFARRSNVLADLQETSLEDDDCQKSSPIQMLRPQAQQYQLNSKKHHQYQPLCREREAEQQANGKKFYYQLNS
KKHHHYQPDLKVHEPMFAKPQEVKAPELANKGYNLPPVLQQKWVRDKDSGCLTKVKDITMELKKLPADLNEHKEPEKV
KTKEDASAQANGVSGSKLKIVKNKNKNGRIVIVMSKYMENGMQATKSKNGDPETAERSPQGTDSSVEKHLTKMKLIKK
LGLVNGFAKLPTDKPAVLGSELNGESLPKKEQLLRIEQTVTEQDKHDEVRGGGPLPEDQPLQLTSKPNLPSSDKGGSQ
AGFQGLKRPFSDTDGEEHGANKRFLSCSSSNMVSSPSQCVAIDQNGHQSRAGLQDCGYTEQEEPIDLSIVKPRPLICS
ATPAEVLTLPHIETQTQTETHKPPQESSESDSNHKTRKDETFPSFQPFLRNIVITDITANCLTVTFKEYVTA

>Kma_LOC108239245
MELPAAGEHVFAVEGIEKKRIRKGKIEYLVKWRGWSPKYNTWEPEENILDPRLLVAFQHRERQEQMMGYRKRGPKPKH
LLLQVPSFARRSSIPAGFEETSQDAEAMLKPDPVQVQRSQPQQYQLNSKKHHQYQPNSQEVPADQLANSRKKFIYQLN
SKKHHHYEPDPHMYDAQASRLKEVVKVQEPASKPANPGWNLPLALQQKWIRDKDTGCLSKVKELAVEVRKPAIKEAQS
ENALKPNPKDAMLPSAISSKMKIIKNKNKNGRIVIVMSKYMDSNKVHGAKDKHRESSKEEKPQTVKPPENNPTLRTKM
LEHLESGISKAICNGRSISVAEHPIKCSPKDRHFSKPSPSTAEEYNTEVARGQADLPEDLPLQLTASSPPTSWTADTS
IPTPTAVDQIRIPSFPGDRKRKLSDPVEDRNFGKVYLMSRSLSVPCSAVAPPQDKPMDLHCSGPRLSSTSTYDVEESQ
EEPMDLSYPKNRKQVETEAPPQSEPEPAVKDTPPGQEDAQKTTEKSKEAPAKKASPFMGNIIITDITTNSLTVTFKEY
VPF

>Kma_Cbx6 (Gene ID: 108231568)
MELSAAGDRIFAAEAILKRRVRKGRLEYLVKWKGWAMKHSTWEPEENILDDRLILGFEQKEREREMHGPKKRGPKPKH
LAAKTRGQKGETSSRASSSRQNTSRSSSSTSSRAAPSSSASLPNLPSSSSSSSTVAPSPKLNSLAATHKLKKDIHRCH
RMSRRPLPRSNPMASTFSSPGGFPSRMHVSPFSETVRILNRRVKPREVKRGRIILNLKVIDKPSKGGAAAGARNMAAG
RQNIPSRNRIIGRKGEAPYRPFQPPLKMLGFPMYGKPFGLQCGGPMSFHSHPGSRTSSGARNSHSTSSHYQAPPSPSS
SSGSEGKSQPAAKASPPSKDSKSGKALTETPKSPSAPASSSDANPGALACSSLPSSPSSSLEDEEQGSTDPSLPSEAG
RKNPRQRRAKNQPPSTLPPGTPGEQTPAPAEPQRVPAEGDPDWHPEMAPSCKDVVVTDVTTNLVTVTIKEFPSPASGP
SSPSAGPEGASSPPPPVTPSEDPSPVKP

>Kma_LOC108236253

MELSSIGDQVFAVESITKKRVRKGNVEYLLKWQGWPPEYSTWEPEDNILDPLLVMAYEENQEKIRSLAYRKKGLRPRK
LVLRNIFAMDLRSAHKDAEKPPPRLRLSLTRSMSTDVEQAIRRPSRRRNRLRLTKRSFNKPVLQQQQQKKVDSMQKD
WGGSGEEEKPECESAAEERCEDSCYGQSEWSSPPFLEQQDLDVEVEVEVEEKVDQQAETCADRLDRGAPEQPKDTTLV
SEPWPGDAVVSPGDGSEKDVEAGWEDGPRERNNATLVIVRVQGSNVCATAEPQREQVDNHCVTTTTAGLPIVPAEAER
PRKVIVTNVTVNSLTVTFKEAKGPEGFFKSC

>Kma_LOC108250439
MELSAIGEQVFAVESIVKKRVRKGNVEYLLKWKGWPPKYSTWEPEEHILDRRLVQAFKEKEERDRVIGHRRKGSKAKK
LLLQNTVYTMDLRSAHKIPAQPPPRLHLSLMRSLVPGDEDGLYAACRLRPPPQQPHRKSKHRRSPHRCLNSSSPSSSQ
ENLEEEEESLAEDGEESEEERQMDQKEAIKRGERDLNGQGGTDHWDSAVVPDASEKAWTPIISPGQVTVTDITLNSLT
VTFRESRVARGFFRDWGLEVWQQ

>Kma_LOC108228443
MELSAVGERVFAAESIIKRRIRKGRIEYLVKWKGWSPKYSTWEPEENILDSRLFVAFEQREREREL YGPKKRGPKPKT
FLLKAQAKVKTRSYEFRNEAVRGITYPSPEPVVTPRAREGLRAVVPTIFPPSAVNRGESICLRPSELSVHDHREAGSD
GPRKRGPKPKPRLVDGSCGPSVSEPHKRRAEEQLSHGAHKLPKLPRGEEGNHKHHHVYYHHHHHHHHCSQGRETSGGT
SYRQLCSDRSLPPYKSDSHGPQGGSGYLAPAHFKHHSKVSPGRPAAPPLMEKPYFLDRPSPTRLHDDLEEVTWRPSLS
DVEQVLVTDVTANFLTVTIKESSTSKGFFRDKR

>Kma_LOC108239259
MELSAVGESVFAAESIIKRRIRRGRWEYLVKWKGWSQKYSTWEPEENILDERLFAAFEERERERELFGPKKRGPKPET
FLLKAKAKAKERTYEFRREAPRGIQVAYPIPEPVTTPRAREGLRAVVPTIFPPSAVNRGESVPVRPPEPERRPRPPPP
DALMDQGSTRYPKKRGRKPKFHYEPQEAAGGSAEPASKMSRRLLHHGETSEQSLLQLTRRFQEKTTITPKPTGERRHA
GGLSYAFNRSARRGVQEGHRTYGLVSFHAPGRLRHPAEERAEPSQSESWTPCLSSWDSVTVTDVTMNFLTVTIRESST
DKGFFRGKR

>Mze_LOC101465599
MEGVTVGQVFDAECILSKRPRKGKFEYLVKWRGWSSKHNSWEPEENILDPRLLAAFHKREQERELLFQKKGKRPRGRP
RKIQPAPTVTKDDRSSSSSSSGLSSTASSSSSEDEEHTKKVKPAPRVHPVPQKRPQILLAKPDTPRKKKRGRKPLHPD
LRALRQAKSRPPPPPSPPGPRHHQLPRDEPRASVKKPLQPASFTYTGLSRSAREEAGSASQTSSASFSQASKPGGLGC
IWTNRSLSTSSLSSSSTSQNKATPTAQNKNSLSELKRSVPEIGRGDGFKVTPVKQGGASGSLGLHSSFGGGAVVQRPL
LAQRRQDGSGGQSGVAPHKHLSTSLSKPPSSASPSPRDRASQALSLRALNLQSVGKLPPTSGLQGSNTSGVAVARANP
RSSGSGMVIKGGAKDARTGGGAPEQGMTKDKLTGGGAARGNSGVRQDGRKHVLGSQNRNLNELSTGDSDETSTSESET
EDPLFPSNSRPSRGNNATDSDTETDWRPARSLLEHVFVTDVTANFITVTVKESPTSVGFFSSRNH

>Mze_LOC101477503
MELPAAGEHVFAVESIEKKRSRKGRVEYLVKWRGWSPRYNTWEPEENILDPRLLDAFQDRERQEQMMGYRKRGPKPKH
LLVQVPSFARRSSILADLQEASLDEDSCQNASPIQMLRPQPQHYQLNSKKHHQYQPLCREREAEQQANGKKFYYQLNS
KKHHHYQPDIKVHEPIFAKPKIDVQAPELANKEYNLPPVLQQKWVRDKDSGCLTKVKDITMELKKLPANFNGHKEQEK
PKPKEDALPQPNGVSSSKLKIVKNKNKNGRIVIVMSKYMENGMQAAKVKNGECDAAEKPPQGTDNGAEKHLEKMKLVK
KLGLINGFAKSPVDKATSLSSGFNGNCPKEKEPSLKAELTVTEQDKHVEVRGQGQLPEDQPLQLTTKPNLLSRPLNRG
VPSSQDKREGQGGFQGLKRHLSDTDGEEREGSKRFLSSRSISAPNTASSPVQSINIDQNGHHSHPGLQDCGYVDQVEP
IDLSIVKSRPKAPVPTAAQPETHTQPEALTHTEEERQTEADTQTETQLETQQPAEVENAESLSVSNHEQKKEDTFPSF
KPFLGNIVITDITTNCLTVTFKEYIAA

>Mze_LOC101467610
MELPAAGEHVFAVEGIEKKRIRKGKIEYLVKWRGWSPKYNTWEPEENILDPRLLVAFQHRERQEQLMGYRKRGPKPKH
LFLQVPSFARRSSIPAGFEETSQDAEGNLKSDPIQAQQRSQPQQYQLNSKKHHQYQPSSQEVPGDQLASNKKKFIYQL
NSKKHHHYEPDPNMYDTQASRLKEVVKVQEAASKPANPGWNLPLALQQKWIRDKDTGCLSKVKELAVEVRKPAVKEAE
SEHALKPNPKDATLPSAISSKMKIIKNKNKNGRIVIVMSKYMDSNKVHGAKGKHGESSREDKPQNMKTSENNPAHATK
LEEHLENGIPKEICNGSSLSVAEHPIKCSPKDRHFSKPSPSTAEEYNTEVACGQADLPDDLPLQLTASSPMTSWAVDT
NIPTPTAVDQIRIPSFPADRKRKLSDPVEDRSVSKIYLSSRSFSVPSTVVTPPQDKPMDLHCNGPRQSGVCTYEDLGS
QEEPMDLSCPKTKKQAPAETPPEPEPAVKDAPPVTEDTQISTEASKEAPVKKTSPFMGNIIITDITTNSLTVTFKEYV
SF

>Mze_ENSMZEG00005006699
MELSAGDRIFAAEAILKRRVRKGRLEYLVKWKGWAMKHSTWEPEENILDDRLILGFEQKERERELHGPKKRGPKPKNL
VMKARGQKGESGSRASNTRQTTLRPTSSTSNRAASSSSSASAHLLPSSSSSAVAPSPKLNSLAATHKLKKDIHRCHRM
SRRPLPRTDPMATTFSNPGGFPSRMHVSPFSETVRILNRRVKPREVKRGRIILNLKVIDKAGRGGTAAGGRNISAGRQ
NIPSRNRIIGKKREAPYRPFQPPLKMLGFPMYGKPFGLQCGGPVPFHSHPSSCSSTGARESRTSPSQFQAPPSPSSSS
GSEGKSPTNATAAQSQPSTGASPSSKDPKSSKPHADTQKSQSTKSSTPAASSDANQAASSSSPFLPSSPSSSLEDEED
EGAPDRTVPSEEGRKNPRQCRAKHQLPTALPPATPGDQTSAPTEPPRVPAEGDPDWHPEMAPSCKDVVVTDVTTNLVT
VTIKEFPSPASCPASPSASPENASSPPPTAASDDPSPAKP

>Mze_Cbx7 (Gene ID: 101468311)

MELSAIGEQVFAVESIVKKRVRKGNVEYLLKWKGWPPKYSTWEPEEHILDRRLVQAYEEKEQRERALGPRRKGAKSKR
ILLQNTVYTMDLRSAHKIPEKPAPRLRLSLTRSLLPEDDHQPYRQESQHRLRTKEFRCFNSNPPSPRQENWEEHGEED
EEEEDEEEGENSMEDDEDREEEQEETQKNTAKESDGILNGHERTDGWSSTGPDEGTTSENIWRPVICPGEITVTDITL
KSLTVTFRESRVAKGFFRDWGLKV

>Mze_LOC101477848
MELSSIGDQVFAVESITNKRVRKGAVEYLLKWQGWPQKYSTWEPEDNILDPLLVLAYEENREKIRTLTYRRKGLRPRR
PILRNVFAMDLRSAHKMTEKPPPRIRLSLTRSMSTDVDHVEHGSMYQRQVRRNGWQRVAKGGRDGKRMHLQKKKMEPM
EEDWGGTSEEEKSESTMGERCEGSLYGLSECSSPALLEQHDLELQVGEKLGGDLMPVSSHTWIDIMGECDSTPKPMLV
RSQSKDSTSASEARLGDAVSVSDRSDSDRGEEGWESGPERSNATSVIVSIRGSRGTAGDAATVFSLAEVRKEEARGDD
SIVAPETASPTADQPKTVILTKVTINSVTVTFKEARVAEGFFKGY

>Mze_Cbx8 (Gene ID: 101477208)
MELSAVGERVFAAESIIKRRIRKGRIEYLVKWKGWSPKYSTWEPEENILDSRLFAAFEQREREREL YGPKKRGPKPKT
FLLKAQAKVKSKSYDFRSEAVRGMRVTYPTPEPIATPRAREGLRAVVPTIFPPSTVNRGESVRVRPPELSPREHQQSL
LHTSSEDLIHIPKKRGPKPKPRFNEVFSEHHKRRGEEQESHGPHKLAKLQGGEDMRLVKLAHRHPENHGHSHKHHYHH
HHHHHHHTQRSGESSYKQLYSDRGLHPHRTDMDTHRIKDVSAYLVPAHFKHHSKMSLSRPAELPQMEKPYFLDRPSPT
RLDDDLDEVTWRPSLGNVEKVLVTDVTTNFLTVTIKESSTSKGFFKDKR

>Mze_LOC101467917
MELSAVGESVFAAESIIKRRIRRGRWEYLVKWKGWSQKYSTWEPEENILDARLFSAFEEREREREMFGPKKRGPKPET
FLLKAKAKAKEKTYEFRREAHRGIQVSYPIPEPIITPRAREGLRTVVPTIFPPSAVNRGESVHIRPPELERRPRTTPA
AQQFPKKRGRKPKMHMHYDKDDGSSSAEPDAKRSKFTNEPTSHHSRRPHHHGETSDHCLMQLTKRFQEETTITPKSHS
EQRHLGGAGFSYPCALASDLRKSEQGRHRIHCLSMKRPAANENSLPREQASPSHTDSIHESSEHPASSWTPCFAHLDT
VTVTDVTLNFLTVTVRESSTDKGFFKDKR

>Oni_Cbx2 (Gene ID: 100704060)
MEELSAVGEQVFDAECILNKRMRKGKLEFLVKWRGWSSKHNSWEPQENILDPRLLAAFNKKEQEKELLLRKRGKRPRG
RPRKILENMPEAPKSSSSSSGSSSSSSDSSSSCSSSSSSSDDDDDDNGQVKQVSPSVRTRELNPVPQKKAQIVMAKQE
PPKKRSRKPLPSEVKEFQQNKGPRKTAKDTDIPGAIKKPVHPASFTFMGFHRGSARDTVGGPNRSSLAQVGAVKNSLS
SVGSGRPLVQPSSLPLNKSSQSRNVTESKLPVSGMSSGTSLDLKAAPVKSKGVAALNLNTSKHPIQGTTQHTLSSPNG
QKKPPAPVMTPQRTPGAKTAASLPSNNVSSNQGLQPLNLQNKLVQSNDAPGNGTAPVSGPRNAANPARKPVTQNPECN
PPKSPATPGRLQARKTQPGTDKVREVNDIQASRVQGRLERSGVHKSPTDVQSQQDRSASKDGKKAKMNEMSTGEEESS
SDSDQDSSYAGQVAVQSQDWKPTRSLIEHVFVTDVTANLVTVTVKESPTSVGFFSIRNC

>Oni_LOC100695490
MEGVTVGQVFDAECILSKRPRKGKFEYLVKWRGWSSKHNSWEPEENILDPRLLAAFHKREQERELLFQKKGKRPRGRP
RKIQPAPTVTKDDRSSSSSSSGLSSTASSSSSEDEEHTKKVKPAPRVHPVPQKRPQILLAKPDTPRKKKRGRKPLHPD
LRALRQAKSRPPPPPPPPAPRHHQLPRDEPRPSVKKPLQPASFTYTGLSRSSRDEASSASQTTSASFSQASKPSALGC
IWTNRSLSTSSLSSSSASQNKATPTAQNKNSVSELKRSVLEMGRGDGFKVTPLKQGGASGSLGLHSSFGGGAAVQRPL
LAQRRQDGSGGQSGVAPHKQLSTSLSKPSSSASPSSRDRASQALSLRALNLQSVGKLPPTSSLQGSNTSGMAVARANP
RSSGSGMVVKGGAKDARAGGGATEQGRMKDKLTGGGAARGNGGGRQDGRKHALGSQNRNLNELSTGDSDETSTSESET
EDALFPSNSRPSLGNNATDSDTETDWRPARSLLEHVFVTDVTANFITVTVKESPTSVGFFSSRNH

>Oni_Cbx4 (Gene ID: 100712470)
MELPAAGEHVFAVESIEKKRSRKGRVEYLVKWRGWSPRYNTWEPEENILDPRLLDAFQDRERQEQMMGYRKRGPKPKH
LLVQVPSFARRSSILADLQEASLDEDSCQNASPIQMLRPQPQHYQLNSKKHHQYQPLCREREAEQQANGKKFYYQLNS
KKHHHYQPDIKVHEPIFAKPKQDVQAPELANKEYNLPPVLQQKWVRDKDSGCLTKVKDITMELKKLPANFNGHKEPEK
PKPKEDALPQPNGVSSSKLKIVKNKNKNGRIVIVMSKYMENGMQPAKVKNGECDAAEKPLQGSDNGAEKHLEKMKLVK
KLGLINGFAKSPVDKASSLSSGFNGNCPKEKEPSLKAELTVTEQDKHVEVRGQGQLPEDQPLQLTTKPNLLSRPLNRG
VPSSQDKREGQGGFQGLKRHLSDTDGEEREGSKRFLSSRSISAPNTASSPVQSITIDQNGHHGHPGLQDCGYVDQVEP
IDLSIVKSRPKAAVPTAAQPETHTQPEALTRTEEERQTEPDTQTETQLETQQPAEEENAESLSVSNHEQKTEDTFPSF
KPFLGNIVITDITTNCLTVTFKEYIAA

>Oni_LOC100692384 / ENSONIG00000017179
MELPAAGEHVFAVEGIEKKRIRKGKIEYLVKWRGWSPKYNTWEPEENILDPRLLVAFQHRERQEQLMGYRKRGPKPKH
LLLQVPSFARRSSIPAGFEETSQDAEGNLKSDPIQAQQRSQPQQYQLNSKKHHQYQPSSQEVPGDQLASNKKKFIYQL
NSKKHHHYEPDPNMYDTQASRLKEVVKVQEAASKPANPGWNLPLALQQKWIRDKDTGCLNKVKELAVEVRKPAVKEAE
SEHALKPNPKDATLPSAISSKMKIIKNKNKNGRIVIVMSKYMDSNKVHGAKGKHGESSREDKPQNMKTSENNPAHATK
LEEHLENGIPKEICNGSSLSVAEHPIKCSPKDRHFSKPSPSTAEEYNTEVARGQADLPDDLPLQLTASSPLTSWAVDT
NIPTPTAVDQIRIPSFPADRKRKLSDPVEDRSVSKIYLSSRSFSVPSTVVTPPQDKPMDLHCNGPRQSSVCTYEDLGS
QEEPMDLSCPKTNKQAQAETPPEPEPAVKDAPPVTEDTQISTEASKEAPVKKISPFMGNIIITDITTNSLTVTFKEYV
ST

>Oni_Cbx6 (Gene ID: 102077022)
MELSAAGDRIFAAEAILKRRVRKGRLEYLVKWKGWAMKHSTWEPEENILDDRLILGFEQKERERELHGPKKRGPKPKN
LVMKARGQKGESSSRASNTRQTTSRPTSSTSNRATSSSSSSASAHLLPSSSSSAVAPSPKLNSLAATHKLKKDIHRCH

RMSRRPLPRTDPMATTFSNPGGFPSRMHVSPFSETVRILNRRVKPREVKRGRIILNLKVIDKAGRGGTAAGSRNISAG
RQNIPSRNRIIGKKREAPYRPFQPPLKMLGFPMYGKPFGLQCGGPVPFHSHPGSCSSTGARESRTSPSQYQAPPSPSS
SSGSEGKSPTNATAAQSQPSTGASPSSKDPKSSKPHADTQKSQSTKSSTPAPSSDANQAASSSPFLPSSPSSSLEDEE
DEGAPDRTVPSEEGRKNPRQRRAKHQLPIALPPTAPGDQTSAPAEPQRVPAEGDPDWHPEMAPSCKDVVVTDVTTNLV
TVTIKEFPSPASCPASPSASPENASSPPPVAASDDPSPAKP

>Oni_Cbx7 (Gene ID: 102076083)
MELSAIGEQVFAVESIVKKRVRKGNVEYLLKWKGWPPKYSTWEPEEHILDRRLVQAYEEKEQRERALGPRRKGSKSKR
ILLQNTVYTMDLRSAHKIPEKPEPRLRLSLTRSLLPEDDHQSYRQESQAQHRLRRKEFRCFNSNPPSPRQENWVEHGE
EDEEEEDEEEEENSMEDDEDREEEQEETQKNTAKESNGILNGHERTDGWSATGPDEGTTSENIWRPVICPGEVTVTDI
TLNSLTVTFRESRVAKGFFRDWGLKV

>Oni_LOC100700457
MELSSIGDQVFAVESITNKRVRKGTVEYLLKWQGWPQKYSTWEPEDNILDPLLVLAYEENREKIRTLTYRRKGLRPRR
PILRNVFAMDLRSAHKMTEKPPPRIRLSLTRSMSTDVDHVERGRMYQRQVRRNGRQRVAKGGRDSKRMHLQKKVEPME
EDWGVTSEEEKSDSTMGERCEGSLYGLSECSSPALLEQHDLELEVEEKVDGDLMPVSSHTWIDVMAECDSTPKPMLVR
SQSNDSASASEATPGDAVSVSDRSDSDRGEEGWELVPERSNATSVIVSIRESRGTAGDAATVCSLAEVRKEEARGNDQ
IVAPETASPTADQPETVILTKVTINSVTVTFKEARVAEGFFRGY

>Oni_Cbx8 (Gene ID: 100712202)
MELSAVGERVFAAESIIKRRIRKGRIEYLVKWKGWSPKYSTWEPEENILDSRLFAAFEQREREREL YGPKKRGPKPKT
FLLKAQAKVKSKSYDFRSEAVRGMRVTYPTPEPVATPRAREGLRAVVPTIFPPSTVNRGESVRVRPPELSPREHHQSL
LHQTNSEELIHIPKKRGPKPKPRFNDGFSEHHKRRAEEQESHGPHKLAKLQGGEDMRLVKLAHRHPENHGHSHKHHYH
HHHHHHHHTQRSGESSYKQLYSDRGLHPHRTDMDTHRMKDGSGYLVPAHFKHHSKMSLSRPAELTQMEKPYFLDRPSP
TRLDDDLDEVTWRPSLGNVEKVLVTDVTANFLTVTIKESSTSKGFFKDKR

>Oni_LOC100692384 / ENSONIG00000017182
MELSAVGESVFAAESIIKRRIRRGRWEYLVKWKGWSQKYSTWEPEENILDARLFSAFEERERER EMFGPKKRGPKPET
FLLKAKAKAKEKTYEFRREAPRGIQVSYPIPEPIITPRAREGLRTVVPTIFPPSAVNRGESVHIRPPEPERRPRTTPA
AALTVQESQQFPKKRGRKPKMHMHYDKDDGSSSAEPDAKRSKFIKEPTSHHSRRPHHHGETSDHCLMQLTKRFQEETT
ITPKSHSEQRHLGGAGFSYPCALASDLRKSEQGRHRTHCLNMKRPAANEYSLPREQAAVTPSHTDSIHDSSEHPASSW
TPCFAHLDTVTVTDVTLNFLTVTVRESSTDKGFFKDKR

>Hbu_LOC102307411
MEELSAVGEQVFDAECILNKRMRKGKLEFLVKWRGWSSKHNSWEPQENILDPRLLAAFNKKEQEKELLLRKRGKRPRG
RPRKILENMPEAPKSSSSSSGSSSSSSDSSSSCSSSSSSSDDDDDDDGGQVKQVSPSVRTRELNPVPQKKAQIVMAKQ
EPPKKRSRKPLPSEVKEFQQNKGPRKTAKDTDIPGAIKKPVHPASFTFMGFHRGSARDAVGGPNRSSLAQVGAVKNSL
SSVGSGRPSVQPSSLPLNKSSQSRNVTESKLPVSGMSSGTSLDLKAAPSKSKGVAALNLNTSKHPIQGTTQHTLSSPN
GQKKPAPVTTAQRIPGAKTAASLPSNVSSNQGLQPLNLQNKLVQSSDAPGNGTAPVSGPRNAANPARKPVTQNQECKP
PKSPATPGRLQARKTQPGTDKVREVNDIQASRVQGRLERSGVHKSPTEAQSQQDRSASKDGKKAKMNEMSTGEEESSS
DSDQDSSYAGQVAVQSQDWKPTRSLIEHVFVTDVTANLVTVTVKESPTSVGFFSIRNC

>Hbu_LOC102302910
MEGVTVGQVFDAECILSKRPRKGKFEYLVKWRGWSSKHNSWEPEENILDPRLLAAFHKREQERELLFQKKGKRPRGRP
RKIQPAPTVTKDDRSSSSSSSGLSSTASSSSSEDEEHTKKVKPAPRVHPVPQKRPQILLAKPDTPQKKKRGRKPLHPD
LRALRQAKSRPPPPPSPPRHQQLPRDEPRASVKKPLQPASFTYTGLSRSAREEAGSASQTSSASFSQASKPGGLGCIW
TNRSLSTSSLSSSSTSQNKATPTAQNKNSLSELKRSVPEIGRGDGFKVTPLKQGGASGSLGLHSSFGGGAVVHRPLLA
QRRQDGSGGQSGVAPHKHLSTSLSKPPSSASPSPRDRASQALSLRALNLQSVGKLPPTSGLQGSNTSGVAVARANPRS
SGSGMVVKGGAKDARTGGRAPEQGVTKDKLTGGGAARGNSGVRQDGRKHALGSQNRNLNELSTGDSDETSTSESETED
ALFPSNSRPSRGNNATDSDTETDWRPARSLLEHVFVTDVTANFITVTVKESPTSVGFFSSRNH

>Hbu_Cbx4 (Gene ID: 102295423)
MELPAAGEHVFAVESIEKKRSRKGRVEYLVKWRGWSPRYNTWEPEENILDPRLLDAFQDRERQEQMMGYRKRGPKPKH
LLVQVPSFARRSSILADLQEASLDEDSCQNASPIQMLRPQPQHYQLNSKKHHQYQPLCREREAEQQANGKKFYYQLNS
KKHHHYQPDIKVHEPIFAKPKIDVQAPELANKEYNLPPVLQQKWVRDKDSGCLTKVKDITMELKKLPANFNGHKEQEK
PKPKEDALPQPNGVSSSKLKIVKNKNKNGRIVIVMSKYMENGMQAAKVKNGECDAAEKPPQGTDNGAEKHLEKMKLVK
KLGLINGFAKSPVDKATTLSSGFNGNCPKEKEPSLKAELTVTEQDKHVEVRGQGQLPEDQPLQLTTKPNLLSRPLNRG
VPSSQDKREGQGGFQGLKRHLSDTDGEEREGSKRFLSSRSISAPNTASSPVQIINIDQNGHHSHPGLQDCGYVDQVEP
IDLSIVKSRPKAPVPTAAQPETHTQPEALTHTEEERQTEADTQTETQLETQQPAEVENAESLSVSNHEQKKEDTFPSF
KPFLGNIVITDITTNCLTVTFKEYIAA

>Hbu_LOC102309824
MELPAAGEHVFAVEGIEKKRIRKGKIEYLVKWRGWSPKYNTWEPEENILDPRLLVAFQHRERQEQLMGYRKRGPKPKH
LFLQVPSFARRSSIPAGFEETSQDVEGNLKSDPIQAQQRSQPQQYQLNSKKHHQYQPSSQEVPGDQLASNKKKFIYQL
NSKKHHHYEPDPNMYDTQASRLKEVVKVQEAASKPANPGWNLPLALQQKWIRDKDTGCLSKVKELAVEVRKPAVKEAE
SEHALKPNPKDATLPSAISSKMKIIKNKNKNGRIVIVMSKYMDSNKVHGAKGKHGESSREDKPQNMKTSENNPAHATK
LEEHLENGIPKEICNGSSLSVAEHPIKCSPKDRHFSKPSPSTAEEYNTEVARGQADLPDDLPLQLTASSPMTSWAVHT

NIPTPTAVDQIRIPSFPADRKRKLSDPVEDRSVSKIYLSSRSFSVPSTVVTPPQDKPMDLHCNGPRQSGVCTYEDLGS
QEEPMDLSCPKTKKQAQAETPPEPEPAVKDAPPVTEDTQISTEASKEAPVKKTSPFMGNIIITDITTNSLTVTFKEYV
SF

>Hbu_Cbx6 (Gene ID: 102311013)
MELSAAGDRIFAAEAILKRRVRKGRLEYLVKWKGWAMKHSTWEPEENILDDRLILGFEQKERERELHGPKKRGPKPKN
LVIKARGQKGESGSRASNTRQTTLRPTSSTSNRAASSSSSASAHLLPSSSSSAVAPSPKLNSLAATHKLKKDIHRCHR
MSRRPLPRTDPMATTFSNPGGFPSRMHVSPFSETVRILNRRVKPREVKRGRIILNLKVIDKAGRGGTAAGGRNISAGR
QNIPSRNRIIGKKREAPYRPFQPPLKMLGFPMYGKPFGLQCGGPVPFHSHPGSCSSTGARESRTSPSQFQAPPSPSSS
SGSEGKSPTNATAAQSQPSTGASPSSKDPKSSKPHADTQKSQSTKSSTPAASSDANQAASSSSPFLPSSPSSSLEDEE
DEGAPDRTVPSEEGRKNPRQCRAKHQLPTTLPLAAPGDQTSAPTEPPRVPAEGDPDWHPEMAPSCKDVVVTDVTTNLV
TVTIKEFPSPASCPASPSASPENASSPPPTAASDDPSPAKP

>Hbu_Cbx7 (Gene ID: 102304092)
MELSAIGEQVFAVESIVKKRVRKGNVEYLLKWKGWPPKYSTWEPEEHILDRRLVQAYEEKEQRERALGPRRKGAKSKR
ILLQNTVYTMDLRSAHKIPEKPAPRLRLSLTRSLLPEDDHQPYRQESQHRLRRKEFRCFHSNPPSPRQENWEEHGEED
EEDEEEEENSMEDDEDREEEQEETQKNTAKESDGILNGHERTDGWSSTGPDEGTTSENIWRPVICPGEITVTDITLKS
LTVTFRESRVAKGFFRDWGLKV

>Hbu_LOC102302921
MELSSIGDQVFAVESITNKRVRKGAVEYLLKWQGWPQKYSTWEPEDNILDPLLVLAYEENREKIRTLTYRRKGLRPRR
PILRNVFAMDLRSAHKMTEKPPPRIRLSLTRSMSTDVDHVEHGSMYQRQVRRNGWQRVAKGGRDGKRMHLQKKKMEPM
EEDWGGTSEEEKSESTMGERCEGSLYGLSECSSPALLEEHDLELQVGEKLGGDLMPVSSHTWIDIMGECDSTPKPMLV
RSQSKDSTSASEARLGDAVSVSDRSDSDRGEEGWESGPERSNATSVIVSIRGSRGTAGDAATVCSLAEVRKEEARGDD
SIVAPETASPTADQPKTVILTKVTINSVTVTFKEARVAEGFFKGY

>Hbu_Cbx8 (Gene ID: 102295731)
MELSAVGERVFAAESIIKRRIRKGRIEYLVKWKGWSPKYSTWEPEENILDSRLFAAFEQREREREL YGPKKRGPKPKT
FLLKAQAKVKSKSYDFRSEAVRGMRVTYPTPEPIATPRAREGLRAVVPTIFPPSTVNRGESVRVRPPELSPREHQQSL
LHTSSEDLIHIPKKRGPKPKPRFNEVFSEHHKRRGEEQESHGPHKLAKLQGGEDMRLVKLAHRHPENHGHSHKHHYHH
HHHHHHHTQRSGESSYKQLYSDRGLHPHRTDMDTHRIKDVSAYLVPAHFKHHSKMSLSRPAELPQMEKPYFLDRPSPT
RLDDDLDEVTWRPSLGNVEKVLVTDVTTNFLTVTIKESSTSKGFFKDKR

>Hbu_LOC102309508
MELSAVGESVFAAESIIKRRIRRGRWEYLVKWKGWSQKYSTWEPEENILDARLFSAFEERERERE MFGPKKRGPKPET
FLLKAKAKAKEKTYEFRREAPRGIQVSYPIPEPIITPRAREGLRTVVPTIFPPSAVNRGESVHIRPPESERRPRTTPA
AAQQFPKKRGRKPKMHMHYDKDDGSSSAEPDSKRSKFTKEPTSHHSRRPHHHGETSDHCLMQLTKRFQEETTITPKSH
SEQRHLGGAGFSYPCALASDLRKSEQGRHRTHCLSMKRPAANENSLSREQASPSHTDSTHESSEHPASSWTPCFAHLD
TVTVTDVTLNFLTVTVRESSTDKGFFKDKR

>Nbr_LOC102797064
MEGVTVGQVFDAECILSKRPRKGKFEYLVKWRGWSSKHNSWEPEENILDPRLLAAFHKREQERELLFQKKGKRPRGRP
RKIQPAPTVTKDDRSSSSSSSGLSSTASSSSSEDEEHTKKVKPAPRVHPVPQKRPQILLAKPDTPRKKKRGRKPLHPD
LRALRQAKSRPPPPPSPPGPRHHQLPRDEPRASVKKPLQPASFTYTGLSRSSREEAGSASQTSSASFSQASKPGGLGC
IWTNRSLATSSLSSGSTSQNKATPTAQNKNSLSELKRNVPEIGRGDGFKVTPLKQGGASGSLGLHSSFGGGAVVQRPL
LAQRRQDGSGGQSGVAPHKHLSTSLSKPPSSASPSPRDRASQALSLRALNLQSVGKLPPTSGLQGSNASGVAVARANP
RSSGSGMVVKGGAKDARAGGGAPEQGKDKLTGGGAARGSSGVRQDGRKHALGSQNRNLNELSTGDSDETSTSESETED
ALFPSNSRPSRGNNATDSDTETDWRPARSLLEHVFVTDVTANFITVTVKESPTSVGFFSSRNH

>Nbr_LOC102782953
MEELSAVGEQVFDAECILNKRMRKGKLEFLVKWRGWSSKHNSWEPQENILDPRLLAAFNKKEQEKELLLRKRGKRPRG
RPRKILENMPEAPKSSSSSSGSSSSSSDSSSSCSSSSSSSDDDDEDDQVKQVSPSVRTRELNPVPQKKAQIVMAKQEP
PKKRSRKPLPSEVKEFQQNKGPRKTAKDTDIPGAIKKPVHPASFTFMGFHRGSARDTVGGPNRSSLAQVGAVKNSLSS
VGSGRPSVQPSSLPLNKSSQSRNVTESKLPVSGMSSGTSLDLKAAPSKSKGVAALNLNTSKHPIQGTTQHTLSSPNGQ
KKPPAPVTTAQRIPGAKTAASLPSNVSSNQGLQPLNLQNKLVQSNDAPGNGTAPASGPRNAANPARKPVTQNQECKPP
KSPATPGRPQARKTQPGTDKVREVNDIQASRVQGRLERSGVHKSPTEAQSQQDRSASKDGKKAKMNEMSTGEEESSSD
SDQDSSYAGQVAVQSQDWKPTRSLIEHVFVTDVTANLVTVTVKESPTSVGFFSIRNC

>Nbr_LOC102794988
MELPAAGEHVFAVEGIEKKRIRKGKIEYLVKWRGWSPKYNTWEPEENILDPRLLVAFQHRERQEQLMGYRKRGPKPKH
LFLQVPSFARRSSIPAGFEETSQDAEGNLKSDPIQAQQRSQPQQYQLNSKKHHQYQPSSQEVPGDQLANNKKKFIYQL
NSKKHHHYEPDPNMYDTQASRLKEVVKVQEAASKPANPGWNLPLALQQKWIRDKDTGCLSKVKELAVEVRKPAVKEAE
SEHALKPNPKDATLPSAISSKMKIIKNKNKNGRIVIVMSKYMDSNKVHGAKGKHGESSREDKPQNMKTSENNPAHATK
LEEHLENGIPKEICNGSSLSVAEHPIKCSPKDRHFSKPSPSTAEEYNTEVARGQADLPDDLPLQLTASSPMTSWAVDT
NIPTPTAVDQIRIPSFPPDRKRKLSDPVEDRSVSKIYLSSRSFSVPSTVVTPPQDKPMDLHCNGPRQSSVCTYEDLGS
QEEPMDLSCPKTKKQAQAETPPEPEPAVKDAPPVTEDTQISTEASKEAPVKKTSPFMGNIIITDITTNSLTVTFKEYV
SF

>Nbr_LOC102784839
MELPAAGEHVFAVESIEKKRSRKGRVEYLVKWRGWSPRYNTWEPEENILDPRLLDAFQDRERQEQMMGYRKRGPKPKH
LLVQVPSFARRSSILADLQEASLDEDSCQNASPIQMLRPQPQHYQLNSKKHHQYQPLCREREAEQQANGKKFYYQLNS
KKHHHYQPDIKVHEPMFAKPKIDVQAPELANKEYNLPPVLQQKWVRDKDSGCLTKVKDITMELKKLPANFNGHKEQEK
PKPKEDALPQPNGVSSSKLKIVKNKNKNGRIVIVMSKYMENGMQAAKVKNGECDGAEKPPQGTDSGAEKHLEKMKLVK
KLGLINGFAKSPVDKATSLSSGFNGNCPKEKEPSLKAELTATEQDKHVEVRGQGQLPEDQPLQLTTKPNLLSRPLNRG
VPSSQDKREGQGGFQGLKRHLSDTDGEEREGSKRFLSSRSISAPNTASSPVQSINIDQNGHHGHPGLQDCGYVDQVEP
IDLSIVKSRPKAAVPTTAQPETHTQPEALTHTEEERQTEADTQTETQLETQQPAEEENAESVSNHEQKKEDTFPSFKP
FLGNIVITDITTNCLTVTFKEYIAA

>Nbr_LOC102784197
MELSAAGDRIFAAEAILKRRVRKGRLEYLVKWKGWAMKHSTWEPEENILDDRLILGFEQKERERELHGPKKRGPKPKN
LVMKARGQKGESSSRASNTRQTTLRPTSSTSNRAASSSSSASAHPLPSSSSSAVAPSPKLNSLAATHKLKKDIHRCHR
MSRRPLPRTDPMATAFSNPGGFPSRMHVSPFSETVRILNRRVKPREVKRGRIILNLKVIDKAGRGGTAAGGRNISAGR
QNIPSRNRIIGKKREAPYRPFQPPLKMLGFPMYGKPFGLQCGGPVPFHSHPGSCSSTGARESRTSPSQFQAPPSPSSS
SGSEGKSPTNATAAQSQPSTGASPSSKDPKSSKPHADTQKSQSTKSSTPAASSDANQAASSSSPFLPSSPSSSLEDEE
DEGAPDRTVPSEEGRKNPRQCRVKHQLPIAPSPAAPGDQTSAPAEPPRVPAEGDPDWHPEMAPSCKDVVVTDVTTNLV
TVTIKEFPSPASCPASPSASPENASSPPPTAASDDPSPAKP

>Nbr_LOC102778087
MELSAIGEQVFAVESIVKKRVRKGNVEYLLKWKGWPPKYSTWEPEEHILDRRLVQAYEEKEQRERALGPRKKGSKSKR
ILLQNTVYTMDLRSAHKIPEKPAPRLRLSLTRSLLPEDDHQPYRQESQHRLRRKEFRCFNSNPPSPRHENWEEHGEED
EEEEDEEEEENSMEEDEDREEEQEETQKNTAKESNGILNGHERTDGWSSTGPDEGATSENIWRPVICPGEITVTDITL
NSLTVTFRESRVAKGFFRDWGLKV

>Nbr_LOC102783421
MELSSIGDQVFAVESITNKRVRKGAVEYLLKWQGWPQKYSTWEPEDNILDPLLVLAYEENREKIRTLTYRRKGLRPRR
PVLRNVFAMDLRSAHKMTEKPPPRIRLSLTRSMSTDVDHVERGSMYQRQVRRNGRQRVAKGGRDGKRMHLQKKKIEPM
EEDWGGTSEEEKSESTMGERCEGSLYGLSECSSPALLEQHDLELEVGEKVGGDLMPVSSHTWIDIMGECDSTPKPMLV
RSQSKDSASASEARLGDAVSVSDRSDSDRGEEGWESGPERSNATSVIVSIRGSRGTAGDAATVCSLAEVRKEEARGDD
SIVAPETASPTADQPETVILTKVTINSVTVTFKEARVAEGFFKGY

>Nbr_LOC102790274
MELSAVGESVFAAESIIKRRIRRGRWEYLVKWKGWSQKYSTWEPEENILDARLFSAFEESHIRPPESERRPRTTPAAA
LTVQESQQFPKKRGRKPKMHMHYDKDDGSSSAEPDAKRSKFTKEPTSHHSRHPHHHGETSDHCLMQLTKRFQEETTIT
PKSHSEQRHLGGAGFSYPCALASDLRKSERGRHRIHCLSMKRPAANENSLPREQASPSHTDSTHESSEHPASSWTPCF
AHLDTVTVTDVTLNFLTVTVRESSTDKGFFKDKR

>Nbr_LOC102784560
MELSAVGERVFAAESIIKRRIRKGRIEYLVKWKGWSPKYSTWEPEENILDSRLFAAFEQREREREELYGPKKRGPKPKT
FLLKAQAKVKSKSYDFRSEAVRGMRVTYPTPEPIATPRAREGLRAVVPTIFPPSTVNRGESVRVRPPELSPREHQQSL
LHASSEDLVHIPKKRGPKPKPRFNDVFSEHHKRRGEEQESHGPHKLAKLQGGEDMRLVKLAHRHPENHGHSHKHHYHH
HHHHHHHTQRSGESSYKQLYSERGLHPHRTDTDTHRIKDVSAYLVPAHFKHHSKMSLSRPAELPQMEKPYFLDRPSPT
RLDDDLDEVTWRPSLGNVEKVLVTDVTANFLTVTIKESSTSKGFFKDKR

>Pny_Cbx2 (Gene ID: 102192419)
MEELSAVGEQVFDAECILNKRMRKGKLEFLVKWRGWSSKHNSWEPQENILDPRLLAAFNKKEQEKELLLRKRGKRPRG
RPRKILENMPEAPKSSSSSSGSSSSSSDSSSSCSSSSSSSDDDDDDGGQVKQVSPSVRTRELNPVPQKKAQIVMAKQ
EPPKKRSRKPLPSEVKEFQQNKGPRKTAKDTDIPGAIKKPVHPASFTFMGFHRGSARDAVGGPNRSSLAQVGAVKNSL
SSVGSGRPSVQPSSLPLNKSSQSRNVTESKLPVSGMSSGTSLDLKAAPSKSKGVAALNLNTSKHPIQGTTQHTLSSPN
GQKKPAPVTTAQRIPGAKTAASLPSNVSSNQGLQPLNLQNKLVQSSDAPGNGTAPVSGPRNAANPARKPVTQNQECKP
PKSPATPGRLQARKTQPGTDKVREVNDIQASRVQGRLERSGVHKSPTEAQSQQDRSASKDGKKAKMNEMSTGEEESSS
DSDQDSSYAGQVAVQSQDWKPTRSLIEHVFVTDVTANLVTVTVKESPTSVGFFSIRNC

>Pny_LOC102215081
MELPAAGEHVFAVESIEKKRSRKGRVEYLVKWRGWSPRYNTWEPEENILDPRLLDAFQDRERQEQMMGYRKRGPKPKH
LLVQVPSFARRSSILADLQEASLDEDSCQNASPIQMLRPQPQHYQLNSKKHHQYQPLCREREAEQQANGKKFYYQLNS
KKHHHYQPDIKVHEPIFAKPKIDVQAPELANKEYNLPPVLQQKWVRDKDSGCLTKVKDITMELKKLPANFNGHKEQEK
PKPKEDALPQPNGVSSSKLKIVKNKNKNGRIVIVMSKYMENGMQAAKVKNGECDAAEKPPQGTDNGAEKHLEKMKLVK
KLGLINGFAKSPLDKATSLSSGFNGNCPKEKEPSLKAELTVTEQDKHVEVRGQGQLPEDQPLQLTTKPNLLSRPLNRG
VPSSQDKREGQGGFQGLKRHLSDTDGEEREGSKRFLSSRSISAPNTASSPVQSINIDQNGHHSHPGLQDCGYVDQVEP
IDLSIVKSRPKAPVPTAAQPETHTQPEALTHTEEERQTEADTQTETQLETQQPAEVENAESLSVSNHEQKKEDTFPSF
KPFLGNIVITDITTNCLTVTFKEYIAA

>Pny_LOC102204981
MELPAAGEHVFAVEGIEKKRIRKGKIEYLVKWRGWSPKYNTWEPEENILDPRLLVAFQHRERQEQLMGYRKRGPKPKH
LFLQVPSFARRSSIPAGFEETSQDAEGNLKSDPIQAQQRSQPQQYQLNSKKHHQYQPSSQEVPGDQLASNKKKFIYQL
NSKKHHHYEPDPNMYDTQASRLKEVVKVQEAASKPANPGWNLPLALQQKWIRDKDTGCLSKVKELAVEVRKPAVKEAE

SEHALKPNPKDATLPSAISSKMKIIKNKNKNGRIVIVMSKYMDSNKVHGAKGKHGESSREDKPQNMKTSENNPAHATK
LEEHLENGIPKEICNGSSLSVAEHPIKCSPKDRHFSKPSPSTAEEYNTEVARGQADLPDDLPLQLTASSPMTSWAVDT
NIPTPTAVDQIRIPSFPADRKRKLSDPVEDRSVSKIYLSSRSFSVPSTVVTPPQDKPMDLHCNGPRQSGVCTYEDLGS
QEEPMDLSCPKTKKQAPAETPPEPEPAVKDAPPVTEDTQISTEASKEAPVKKTSPFMGNIIITDITTNSLTVTFKEYV
SF

>Pny_Cbx6 (Gene ID: 102193150)
MELSAAGDRIFAAEAILKRRVRKGRLEYLVKWKGWAMKHSTWEPEENILDDRLILGFEQKERERELHGPKKRGPKPKN
LVMKARGQKGESGSRASNTRQTTLRPTSSTSNRAASSSSSASAHLLPSSSSSAVAPSPKLNSLAATHKLKKDIHRCHR
MSRRPLPRTDPMATTFSNPGGFPSRMHVSPFSETVRILNRRVKPREVKRGRIILNLKVIDKAGRGGTAAGGRNISAGR
QNIPSRNRIIGKKREAPYRPFQPPLKMLGFPMYGKPFGLQCGGPVPFHSHPGSCSSTGARESRTSPSQFQAPPSPSSS
SGSEGKSPTNATAAQSQPSTGASPSSKDPKSSKPHADTQKSQSTKSSTPAASSDANQAASSSSPFLPSSPSSSLEDEE
DEGAPDRTVPSEEGRKNPRQCRAKHQLPTTLPLAAPGDQTSAPTEPPRVPAEGDPDWHPEMAPSCKDVVVTDVTTNLV
TVTIKEFPSPASCPASPSASPENASSPPPTAASDDPSPAKP

>Pny_Cbx7 (Gene ID: 102210279)
MELSAIGEQVFAVESIVKKRVRKGNVEYLLKWKGWPPKYSTWEPEEHILDRRLVQAYEEKEQRERALGPRRKGAKSKR
ILLQNTVYTMDLRSAHKIPEKPAPRLRLSLTRSLLPEDDHQPYRQESQHRLRTKEFKCFNSNPPSPRQENWEEHGEED
EEEDEEEEENSMEDDEDREEEQEETQKNTAKESNGILNGHERTDGWSSTGPDEGTTSENIWRPVICPGEITVTDITL
KSLTVTFRESRVAKGFFRDWGLKV

>Pny_LOC102212884
MELSSIGDQVFAVESITNKRVRKGAVEYLLKWQGWPQKYSTWEPEDNILDPLLVLAYEENREKIRTLTYRRKGLRPRR
PILRNVFAMDLRSAHKMTEKPPPRIRLSLTRSMSTDVDHVEHGSMYQRQVRRNGWQRVAKGGRDGKRMHLQKKKMEPM
EEDWGGTSEEEKSESTMGERCEGSLYGLSECSSPALLEEHDLVLQVGEKLGGDLMPVSSHTWIDIMGECDSTPKPMLV
RSQSKDSTSASEARLGDAVSVSDRSDSDRGEEGWESGPERSNATSVIVSIRGSRGTAGDAATVCSLAEVRKEEARGDD
SIVAPETASPTADQPKTVILTKVTINSVTVTFKEARVAEGFFKGY

>Pny_Cbx8 (Gene ID: 102214806)
MELSAVGERVFAAESIIKRRIRKGRIEYLVKWKGWSPKYSTWEPEENILDSRLFAAFEQRERERELYGPKKRGPKPKT
FLLKAQAKVKSKSYDFRSEAVRGMRVTYPTPEPIATPRAREGLRAVVPTIFPPSTVNRGESVRVRPPELSPREHQQSL
LHTSSEDLIHIPKKRGPKPKPRFNEVFSEHHKRRGEEQESHGPHKLAKLQGGEDMRLVKLAHRHPENHGHSHKHHYHH
HHHHHHHTQRSGESSYKQLYSDRGLHPHRTDMDTHRIKDVSAYLVPAHFKHHSKMSLSRPAELPQMEKPYFLDRPSPT
RLDDDLDEVTWRPSLGNVEKVLVTDVTTNFLTVTIKESSTSKGFFKDKR

>Pny_LOC102195395*
MELSAVGESVFAAESIIKRRIRRGRWEYLVKWKGWSQKYSTWEPEENILDARLFSAFEERERerEMFGPKKRGPKPET
FLLKAKAKAKEKTYEFRREAPRGIQVSYPIPEPIITPRAREGLRTVVPTIFPPSAINRGESVHIRPPESERRPRKTPA
AAQQFPKKRGRKPKMHMHYDKDDGSSSAEPDSKRSKFTNEPTSHHSRRPHHHGETSDHCLM

>Aca_Cbx2 (Gene ID:113020225)
MEELSAVGEQVFDAECILNKRMRKGKLEFLVKWRGWSSKHNSWEPQENILDPRLLAAFNKKEQEKELLLRKRGKRPRG
RPRKILENMPEAPKSSSSSSGSSSSSSDSSSSCSSSSSSSDDDDDDDGSQVKQVSPSVRTRELNPVPQKKAQIVMAKQ
EPPKKRSRKPLPSEVKEFQQNKGPRKTAKDTDIPGAIKKPVHPASFTFMGFHRGSARDTVGGPNRSSLAQVGAVKNSL
SSVGSGRPSIQPSSLPLNKSSQSRNVTESKLPVSGMSSGTSLDLKAAPSKSKGVAALNLNTSKHPIQGTTQHTLSSPN
GQKKPAPVTTAQRIPGAKTVASLPSNVSSNQGLQPLNLQNKLVQSSDAPGNGTAPVSGPRNAANPARKPVTQNQECKP
PKSPATPGRLQARKTQPGTDKVREVNDIQASRVQGRLERSGVHKSPTEAQSQQDRSASKDGKKAKMNEMSTGEEESSS
DSDQDSSYAGQVAVQSQDWKPTRSLIEHVFVTDVTANLVTVTVKESPTSVGFFSIRNC

>Aca_LOC113027934
MEGVTVGQVFDAECILSKRPRKGKFEYLVKWRGWSSKHNSWEPEENILDPRLLAAFHKREQERELLFQKKGKRPRGRP
RKIQPAPTVTKDDRSSSSSSSGLSSTASSSSSEDEEHTKKVKPAPRVHPVPQKRPQILLAKPDTPRKKKRGRKPLHPD
LRALRQAKSRPPPPPSPPGPRHHQLPRDEPRASVKKPLQPASFTYTGLSRSAREEAGSASQTSSASFSQASKPGGLGC
IWTNRSLSTSSLSSSSTSQNKATPTAQNKNSLSELKRSVPEIGRGDGFKVTPVKQGGASGSLGLHSSFGGGAVVQRPL
LAQRRQDGSGGQSGVAPHKHLSTSLSKPPSSASPSPRDRASQALSLRALNLQSVGKLPPTSGLQGSNTSGVAVARANP
RSSGSGMVIKGGAKDARTGGGAPEQGMTKDKLTGGGAARGNSGVRQDGRKHVLGSQNRNLNELSTGDSDETSTSESET
EDPLFPSNSRPSRGNNATDSDTETDWRPARSLLEHVFVTDVTANFITVTVKESPTSVGFFSSRNH

>Aca_LOC113023624
MELPAAGEHVFAVESIEKKRSRKGRVEYLVKWRGWSPRYNTWEPEENILDPRLLDAFQDRERQEQMMGYRKRGPKPKH
LLVQVPSFARRSSILADLQEASLDEDSCQNASPIQMLRPQPQHYQLNSKKHHQYQPLCREREAEQQANGKKFYYQLNS
KKHHHYQPDIKVHEPIFAKPKIDVQAPELANKEYNLPPVLQQKWVRDKDSGCLTKVKDITMELKKLPANFNGHKEQEK
PKPKEDALPQPNGVSSSKLKIVKNKNKNGRIVIVMSKYMENGMQAAKVKNGECDAAEKPPQGTDNGAEKHLEKMKLVK
KLGLINGFAKSPVDKATSLSSGFNGNCPKEKEPSLKAELTVTEQDKHVEVRGQGQLPEDQPLQLTTKPNLLSRPLNRG
VPSSQDKREGQGGFQGLKRHLSDTDGEEREGSKRFLSSRSISAPNTASSPVQSINIDQNGHHSHPGLQDCGYVDQVEP
IDLSIVKSRPKAPVPTAAQPETHTQPEALTHTEEERQTEADTQTETQLETQQPAEVENAESLSVSNHEQKKEDTFPSF
KPFLGNIVITDITTNCLTVTFKEYIAA

>Aca_LOC113020834
MELPAAGEHVFAVEGIEKKRIRKGKIEYLVKWRGWSPKYNTWEPEENILDPRLLVAFQHRERQEQLMGYRKRGPKPKH
LFLQVPSFARRSSIPAGFEETSQDAEGNLKSDPIQAQQRSQPQQYQLNSKKHHQYQPSSQEVPGDQLASNKKKFIYQL
NSKKHHHYEPDPNMYDTQASRLKEVVKVQEAASKPANPGWNLPLALQQKWIRDKDTGCLSKVKELAVEVRKPAVKEAE
SEHALKPNPKDATLPSAISSKMKIIKNKNKNGRIVIVMSKYMDSNKVHGAKGKHGESSREDKPQNMKTSENNPAHATK
LEEHLENGIPKEICNGSSLSVAEHPIKCSPKDRHFSKPSPSTAEEYNTEVARGQADLPDDLPLQLTASSPMTSWAVDT
NIPTPTAVDQIRIPSFPADRKRKLSDPVEDRSVSKIYLSSRSFSVPSTVVTPPQDKPMDLHCNGPRQSGVCTYEDLGS
QEEPMDLSCPKTKKQAPAETPPEPEPAVKDAPPVTEDTQISTEASKEAPVKKTSPFMGNIIITDITTNSLTVTFKEYV
SF

>Aca_Cbx6 (Gene ID:113024612)
MELSAAGDRIFAAEAILKRRVRKGRLEYLVKWKGWAMKHSTWEPEENILDDRLILGFEQKERERELHGPKKRGPKPKN
LVMKARGQKGESGSRASNTRQTTLRPTSSTSNRAASSSSSASAHLLPSSSSSAVAPSPKLNSLAATHKLKKDIHRCHR
MSRRPLPRTDPMATTFSNPGGFPSRMHVSPFSETVRILNRRVKPREVKRGRIILNLKVIDKAGRGGTAAGGRNISAGR
QNIPSRNRIIGKKREAPYRPFQPPLKMLGFPMYGKPFGLQCGGPVPFHSHPSSCSSTGARESRTSPSQFQAPPSPSSS
SGSEGKSPTNAAAAQSQPSTGASPSSKDPKSSKPHADTQKSQSTKSSTPAASSDANQAASSSSPFLPSSPSSSLEDEE
DEGAPDRTVPSEEGRKNPRQCRAKHQLPTALPPATPGDQTSAPTEPPRVPAEGDPDWHPEMAPSCKDVVVTDVTTNLV
TVTIKEFPSPASCPASPSASPENASSPPPTAASDDPSPAK

>Aca_LOC113027596
MELSAIGEQVFAVESIVKKRVRKGNVEYLLKWKGWPPKYSTWEPEEHILDRRLVQAYEEKEQRERALGPRRKGAKSKR
ILLQNTVYTMDLRSAHKIPEKPAPRLRLSLTRSLLPEDDHQPYRQESQHRLRTKEFRCFNSNPPSPRQENWEEHGEED
EEEEDEEEGENSMEDDEDREEEQEETQKNTAKESDGILNGHERTDGWSSTGPDEGTTSENIWRPVICPGEITVTDITL
KSHTVTFRESRVAKGFFRDWGLKV

>Aca_LOC113021152
MELSSIGDQVFAVESITNKRVRKGAVEYLLKWQGWPQKYSTWEPEDNILDPLLVLAYEENREKIRTLTYRRKGLRPRR
PILRNVFAMDLRSAHKMTEKPPPRIRLSLTRSMSTDVDHVEHGSMYQRQVRRNGWQRVAKGGRDGKRMHLQKKKMEPM
EEDWGGTSEEEKSESTMGERCEGSLYGLSECSSPALLEQHDLELQVGEKLGGDLMPVSSHTWIDIMGECDSTPKPMLV
RSQSKDSTSASEARLGDAVSVSDRSDSDRGEEGWESGPERSNATSVIVSIRGSRGTAGDAATVCSLAEVRKEEARGDD
RIVAPETASPTADQPKTVILTKVTINSVTVTFKEARVAEGFFKGY

>Aca_Cbx8 (Gene ID:113023623)
MELSAVGERVFAAESIIKRRIRKGRIEYLVKWKGWSPKYSTWEPEENILDSRLFAAFEQREREREELYGPKKRGPKPKT
FLLKAQAKVKSKSYDFRSEAVRGMRVTYPTPEPIATPRAREGLRAVVPTIFPPSTVNRGESVRVRPPELSPREHQQSL
LHTSSEDLIHIPKKRGPKPKPRFNEVFSEHHKRRGEEQESHGPHKLAKLQGGEDMRLVKLAHRHPENHGHSHKHHYHH
HHHHHHHTQRSGESSYKQLYSDRGLHPHRTDMDTHRIKDVSAYLVPAHFKHHSKMSLSRPAKLPQMEKPYFLDRPSPT
RLDDDLDEVTWRPSLGNVEKVLVTDVTTNFLTVTIKESSTSKGFFKDKR

>Aca_LOC113020844
MELSAVGESVFAAESIIKRRIRRGRWEYLVKWKGWSQKYSTWEPEENILDARLFSAFEEREREREMFGPKKRGPKPET
FLLKAKAKAKEKTYEFRREAPRGIQVSYPIPEPIITPRAREGLRTVVPTIFPPSAVNRGESVHIRPPELERRPRTTPA
AAQQFPKKRGRKPKMHMHYDKDDGSSSAEPDAKRSKLTNEPTSHHSRRPHHHGETSDHCLMQLTKRFQEETTITPKSH
SEQRHLGGAGFSYPCALASDLRKSEQGRHMTHCLSMKRPAANENSLPREQASPSHTDSIHESSEHPASSWTPCFAHLD
TVTVTDVTLNFLTVTVRESSTDKGFFKDKR

>Aci_Cbx2 (ENSACIG00000010758)
MEELSAVGEQVFDAECILNKRMRKGKLEFLVKWRGWSSKHNSWEPQENILDPRLLAAFNKKEQEKELLLRKRGKRPRG
RPRKILVTSKSSSSSSGSSSSSSDSSSSCSSSSSSSDDDDDDDSQVKQVSPGLRTRDLNPVPQKKAQIVMAKQEPPKK
RSRKPLPSEVKEFQQNKGPHKTAKDSDIPGAIKKPVHPASFTFMGFHRGSARDTVGGPNRSSLTQVGAVKNSMSPVGS
GRPSVQSASLPLNKASQSRNVTEGKLSVSGMSSGTSLDLKAVPRTTQHTLSSPNGQKKAQAPVTTVQRTPNTKTVASL
PSNVSSNQGLQPLNLQNKLVQSNDVPGNGTAPVSGPRNAANPARKPTVTQNQEFNPPKSPATPGRVQARKTQPGADKV
REVNEIQTSRVQSRLEKSGVHKSPTEVQSQQDRSASKDGKKAKMNEMSTGEEESSSDSDQDSPYAGRVAVQSQDWKPT
RSLIEHVFVTDVTANLVTVTVKESPTSVGFFSIRNC

>Aci_ENSACIG00000023237
GAGSCSMEGVTVGQVFDAECILSKRPRKVSVGRGPVVLHNSWEPEENILDPRLLAAFHKREQERELLFQKKGKRPRGR
PRKIQVKTLPVPTVTKDNRSSSSSSSGLLSSASSSSSEDEEHTKKVKTGPRVHPIPQKRPQIMLAKPDPPRKKKRGRK
PLHPDLRALKQAKSHPPPPPPPAPRHHQISRDEPRPSVKKPLQPASFTYTGLSRASRDEASKPGSLSCIWTNRSLSGS
SPSSGSSSHNRATPSSQSKNSLSELKRSIPEAVRGDGFKATPLKQGGTSGSLGLHSSFXGGSAVRRSPLGQRRQDGAG
GQSTLVTHKQLNSSLSKPSSSASPSPRDRASQALSLRALNLQSVSKPPSSIGLQGSNTSGAAVARASLRSSGSSMVIK
GGSGSTKNTRAGATEQGRVKEKLAGGGAARGNGGGRQDERKHGLGSQNRNLNELSTGDSEETSSSESEPEDTLFPSSS
RPSLGNNATDSDTETDWRPTRSLLEHVFVTDVTANFITVTVKESPTSVGFFSSRNH

>Aci_ENSACIG00000019976*
MELPAAGEHVFAVESIEKKRSRKGRVEYLVKWRGWSPRYNTWEPEENILDPRLLDAFQDR

>Aci_ENSACIG00000021421*
MDLPAAGEHVFAVEGIEKKRIRKGKIEYLVKWRGWSPKYNTWEPEENILDPRLLVAFQLRERQEQMMGYRKRGPKPKH
PFFQVPSFARRSSIPTGLEETSQDAEANLKSDPIQVQQRSQPQQYQLNSKKHHQYQPSSQEIPGDQMWGNS

>Aci_cbx6a (ENSACIG00000003935*)
MELSAVGDRVFAAEAILKRRVRKGRLEYLVKWKGWAMKHSTWEPEENILDDRLILGFEQKERERELHGPKKRGPKPKN
FVTKVLRQTTSRPTSSTSSRAASSSSSSASPHHIPSSSSSTVAPSPKLNSLAATHKLKKDIHRCHRMSRRPLPRTDPM
ATTFSTPGGFPSRMHVSPFSETVRILNRRVKPREVKRGRIILNLKVIDKAGRGGTAAGSRNISAGRQNIPSRNRIIGK
KGEAPYRPFQPPLKMLGFPMYGKPFGLQCGGAVPFHSXPGSCSSTGARDSRTTSSQYQSPPSPSSSSGSEGKSPTNAT
SAQSQPATRASPSSKDPKSSKPHAETQKSQSTKPSTPAPSLDANQAASSSPFLPASPSSSVEDEEDEGAPDRTTAYHL
KHHTPTVKRGGGGVVLQPQDPGHLAVIESSMNSFVFQSILESSVRLTPEAWSKLSDATGQ

>Aci_ENSACIG00000010051*
MELSSIGDQVFAVESITNKRVRKGTVEYLLKWQGWPPKYSTWEPEDNILDPLLVLAYEEK

>Aci_ENSACIG00000021398*
MELSAIGEQVFAVESVVKKRVRKGHVEYLLKWKGWPPKYSTWEPEEHILDRRLVQAYEEK

>Aci_Cbx8a (ENSACIG00000021387)
MELSAVGESVFAAESIIKRRIRRGRWEYLVKWKGWSQKYSTWEPEENILDARLFSAFEERERER EREMFGPKKRGPKPET
FLLKAKAKAKEKTYEFRREAPRGIQVSYPIPEPVITPRAREGLRTVVPTIFPPSAVNRGESVHIRPPESERRPRPTPP
APPTVQESQRFPKKRGRKPKLHTHFDKDDGSSSAEPDAKRSKLMREPMSHHSRPPHHHGETSDHCLMQLTKRFQEETR
ITPKSNSEQRHLGGAGLSYSCALASRSSDQGRHRTHCLSMIPRPSKLKQHAAHDQVKEYSLPREQAAVTSSQIDSIHE
QPASSWTPCFTNLDTVTVTDVTLNFLTVTVRENSTDKGFFKDKR

>Aci_ENSACIG00000019984*
MELSAVGERVFAAESIIKRRIRKGRIEYLVKWKGWSPKYSTWEPEENILDSRLFAAFEQRPSLGNVEKVLVTDVTTNF
LTVTIKESSTSKGFFKDKR

>Tru_LOC101075336
MEELSAVGEQVFDAECILNKRLRKGKLEFLVKWRGWSAKHNSWEPQENILDPRLLAAFNKKEHEKELLMRKKGKRPRG
RPRKILENLPEPEKSSSSSSGSSSSSSDSSSSCSSSSSSEDDDEDEDGGQMNPSIRTRDPXPVPQKKAQIVMAKQEPP
LKRSRKPLPPDVKDFPQNKGPRRVLKTYQESSLPGTIKKPVHPASFTFMGFHRGSRDPAGVQTRSPLTPASSAGAQSV
GRSGPSRSTDEGKTSGSGPGSGAALALKADAAKGKEAQNSNSARISSPGTVQQTPGSPSGPKRPPGPALLQRVPTSRA
APSLPSRTSSGSGLQPLNLQSKLVPARDATGNGTGPVRNAAAKKSLVAPTREYSPPGRLQSRKNLQDKVPENTEIQNP
KVQTRLDKSSIQKPAEVQQDRSAPKDGKKKNELSAGEDESSSESDQDQNASVAVQNQDWKPMRSLIEHVFVTDVTANL
VTVTVKESPTSVGFFSLRNY

>Tru_LOC101071244*
MYPQQTATEGKVRVSGQVERLVVQAQQLGAGGKHPGPAAASGVPQEERELLFQKKGKRPRGRPRKIPLPEVSPDPSSS
SSSSGLSEPSSDDEEQVKKVKAGPRVQPGPQKRPQILLARPDLPRRKKRGRKPLHPDLKAKNEPPPQPSRHHHLIRPQ
RAELRPGIKKPLQPASFTYTGLARTSRDESSTSSSFSHTSAKPGSVSGTWSNRSLVSCGSASFNRTSPSPQHKMSLSE
LKRSVLDTGIFKAVPLKNPSAASSLGLHGGFVGGQAVQRPPLSLRKQEGVGGASCLVQHKQQNAALSKPSSLTAHRDR
INQAFGLRTLNLHAVGRTPSSGSHQGNISGAGAATSRSSLRSNAGVVKGVAERLKDTRTSAGQGRGLPAGGGSDQRAA
KETLAGGARLEDRNLSRGVNELSAGDSDESSSSESEAAAAAYLSNSRRSLGDDTNESDTETDWRPARSLLEHVFVTDV
TANFLTVTVKESPTSVGFFSSGGH

>Tru_LOC101076082
MEIPAAGEHVFAVENIEKKRSRKGRFEYLVKWRGWSSKYNTWEPEENILDPRLLDAFQERERQEQLMGYRKRGPKPKH
LLIQVPSFARRSSVLADIREVSLEEGRCQPAKPAETLRPQNQQYQLNSQKHHQYPPVCREQDGDRQSSGKKFYYQLNG
KKHHQYQPDLKVQGPLCAEALDVKALELPRQGCNLPPVLQQKWVRDKDSGCLTKVKDIAMELKKLPADLNGHKEPKNM
GCAEEEPRQSDAVSSSKLKIVKNKNKNGRIVIVMSKYMENGIHPAKTRAGDSEAAEPATPGTDGGAESHYEKMRLVRE
LGLMNGFAKHKPTVPSSGFNGDGLEERKQPPKSDPTVAERGKDVEVTGQQQLPADQPLQLTTKSNLLSVTPDRGAPPH
GDRRGGQGGLQALKRRLPDAASQEQGGVKRLVSSSGDSASSQSPEQERGFVDQEEPIDLRVLKPRPAEPPAGNAAEAP
PAPERPTDRLESQLQTEAPAAAEGAPPPLSESRHEQRKEEEFPSFQPLLGNIVITDITTNCLTVTFKEYVAA

>Tru_LOC101075073
MELPAAGEHVFAVEGIEKKRIRKGKVEYLVKWRGWSPKYNTWEPEENILDPRLLVAFQHRERQEQLVGYRKRGPKPKH
LLLQVPSFARRSSIPAGLEETCQEADDNAKADPMEVQRPQPQQYQLNSKKHHQYQPSNQEVPTDQQMNGKKKLIYQLN
SKKHHHYEPDLNMYEAQGSRLKEVVKVQESGSKPANPGWNLPLALQQKWVRDKETGCLSKVKESAVEARKPSVKDAES
EHALKPNSKDASLPSSISSKMKIIKNKNKNGRIVIVMSKYMDNNKVHGAKAKHGETSSDMKAQSTKPPENNPAHSTKI
VEYLENGIPKELCSARTLPVAEHPMKCPPKDRHFSKPSPSTAEEYNTEVARGQADLPDDLPLQLTASSPVTSWAPDTN
IPTPTSLDHIRIPSFPKDRKRKLSDPVDHRSVSKAYLTSRSLSLPSTAVTPPQDTPMDLHCSGRRHSSACETVDSGGQ
EEPMDLSFPKTKKPAEPEVPLAPPPAVETAPPDSGDAQKSVEKAAVKKISPFMGNIIITDITTNSLTVTFKEYVSF

>Tru_Cbx6 (Gene ID: 101072963)
MMELSAAGDRIFAAEAILKRRVRKGQLEYLVKWKGWAMKHSTWEPEENILDDRLILGFEKKERERELHGPKKRGPKPK
NIAAKGRGQKGETSSRNSNARQTASRSAPSTSNRPTPSSSASPQHLPSPSSSAFAPSAKLNSHAATHKLKKDIHRCHR
MSRRPLPRSDPMAGSFSTPGGLPSRLHVSPFSETVRILNRRVKPREVKRGRIILNLKVIDKAGRGGSSVGSRNTAAGR
QNIPSRNRIIGKKGEVPYRPFQPPLKMLGFPMYGKPFGIQYGGPLAFHSSPATCSTTATRDTDSASTQYQIPPSPSSS
CSSEGKCPTSTSAAQSQPSECPKSSTPHAETQKGQNAEPPAPAPPADPASASLPSSPSSSLDGEKECVLDCSPPPQEC
KKNPHQFRAKHQPTGNPGDHTSPPAESKRVPAEGDPDWHPEMAPSCKDVVVTDVTTNLVTVTIKEFPSHASPSAGPED
PSTPPPSASVDSPSPPKP

>Tru_LOC101070872
MELSAIGEQVFAVESIVKKRVRKGNVEYLLKWKGWPPKYSTWEPEEHILDQRLVQAYEEKEQRDRALGHRRKGSKTKR
LLQNTVYTMDLRSAHKTPEKPPPRLHLSLTRSVVSEDEEQLYDDCRQESEPEPEPAHRKVQFKCLNSPPPSPAREDWG
GEDEDKDNLDSEDDGEDDRESFKSGQEKTDDWCSTIRSDEVLAAEVSDTEIWKPVIGPGEVTVTDITLNSLTVTFRES
AVAKDFFREWSLKV

>Tru_LOC101079131
MELSSIGDQ*VFAVESITKKRVRKVQYLGTGGQYFGPTACLGLRAEEKIRALAYRRKGLRPRK*LVLRNLFAMDLRSSHK
EKPRPRLRLSLTRAMSTDIDQGERAGMFRRPLWRKNRQRASKRGPEGPSHRTICPLRKKKSLEEDWVATSEEDKQESN
KNEDVLYDGQSKCSPPAFRQQEDMDEETVDDTESTGTWTDGSCTGTPGQTQTLACEQSKEDAFAAEVGPGDTGTACER
SCWVLQGLQGGTEAAQHPAVCQSKAVSVIVCVSGCGEMPGDSVCVAAKEAGGDAAKERRLTSQVAAKYPGKVITTNVT
INSLTVTFKEATTAEGFFNGC

>Tru_Cbx8 (Gene ID: 101076311)
MELSAVGERVFAAESIIKRRIRKGRIEYLVKWKGWSPKYSTWEPEENILDSRLFVAFEQREREREYGPKKRGPKPKT
FLLKAQAKEKVKSYDFRSDSVRGMHISYPSPEPVAAPRAREGLRAVVPTIFPPSTVNRGESVRVHPSDACTRDLRLSS
LQPGADGLHFPKKRGPKPKLRFKDGGCGPAASERGRAEAPVSQHAHQHAHQHAHQHAHQHAHQHAHQHAHQHAQLQEG
GKLRLLRVSHGHHGHHHHRHHHRHHSANYPAPSQRPYRRFYSDRGLLAHRRDTDARRTQDGGSYTATPPPRQRPATKP
SRPAHLPLLDKPYVLDKPSPSTLDTDLDQVTWRPCLSSVEKVLVTDVTSNFLTVTIKESSTCQGFFKAKR

>Tru_LOC101075336
MEELSAVGEQVFDAECILNKRLRKGKLEFLVKWRGWSAKHNSWEPQENILDPRLLAAFNKKEHEKELLMRKKGKRPRG
RPRKILENLPEPEKSSSSSSGSSSSSSDSSSSCSSSSSSEDDDEDEDGGQMNPSIRTRDPXPVPQKKAQIVMAKQEPP
LKRSRKPLPPDVKDFPQNKGPRRVLKTYQESSLPGTIKKPVHPASFTFMGFHRGSRDPAGVQTRSPLTPASSAGAQSV
GRSGPSRSTDEGKTSGSGPGSGAALALKADAAKGKEAQNSNSARISSPGTVQQTPGSPSGPKRPPGPALLQRVPTSRA
APSLPSRTSSGSGLQPLNLQSKLVPARDATGNGTGPVRNAAAKKSLVAPTREYSPPGRLQSRKNLQDKVPENTEIQNP
KVQTRLDKSSIQKPAEVQQDRSAPKDGKKKNELSAGEDESSSESDQDQNASVAVQNQDWKPMRSLIEHVFVTDVTANL
VTVTVKESPTSVGFFSLRNY

>Tni_Cbx2 (ENSTNIG00000011276)
MEELSAVGEQVFDAECILNKRLRKGKLEFLVKWRGWSAKHNSWEPQENILDPRLLAAFNKKEQEKELLMRKKGKRPRG
RPRKVLVSTAGSAGPTGVRLSCPVFQENLPGPGKSSSSSSGYEGQVNPSIRTRDHPVPQKKAQIVMAKPEPPPKRSRK
PPPPDASKGPRRVLKTFPEPGPPGTIKKPVHPASFTFMGFHRGSGGPIRSPLSSRSSGGVGRSGPGRNPDEGRTLESR
LAPKADTKEAQNPTRTRSPSQGSAQHGPGSPETPGTGALQRTPGAKAAPVLPSKAASAPASAPQPLNLQSKPVQAKDA
SGHTASAPAKRSVAPPNREYAPAAPPGRLPSRKSLQDKVPETAEVQKAKVQTRLDKSGVQKAAELHQDRSAAKDGKKK
NEMSAGEDESSSESDQDQSASAVQNQGWKPARSLIEHVFVTDVTANLVTVTVKESPTSVGFFSLRPY

>Tni_ENSTNIG00000003572
VMEGVTVGQVFDAECILSKRPRKGKFEYLVKWRGWSSKHNSWEPEENILDPRLLAAFHKCREQERELLFQKKGKRPRG
RPRKHPLPEVAADRSSSSSSSGLSEPSSDDEEQVKKVKPGPRVQPGPQKRPQILLARPDPPRRKKRGRKPLHADLRTK
TESPPLPSRHHHLIRPQRVELRPGMKKPLQPASFTYTGLTRTSREESSTSSSSFSHTSTKPGSMSGTWSNRTLNSSCG
SASFSRTSPSPQNKMSLSELKRSVLDSSSFKTLPLKSPGAASSLGLPGGFGGGQGVQRSPLSLRKAEGGGGASCLVQH
KQNSGLSKPSSSPAHRDRLNQALSLRTLNLQASRSSLRSIAGVVKADRIKDTRAPAGQGRTHPAGGVAKETLAGGARL
EDRNPSRGGNELSAGDSDESSSSESEAAAALYMGNSRRSLGDDTNESDAETDWRPARSLLEHVFVTDVTANFLTVTVK
ESPTSVGFFSSGTH

>Tni_Cbx4 (ENSTNIG00000017994)
MELPAAGEHVFAVEGIEKKRIRKGKVEYLVKWRGWSPKYNTWEPEENILDPRLLVAFQHRERQEQLMGYRKRGPKPKH
LLLQVPSFARRSSIPAGLEEPSQDADNNAKADPVEVQRPQPQQYQLNSKKHHQYQPSNQEVPADQQANGKKKFIYQLN
SKKHHHYEPDLNMYEAQGSRLKEESGSKPANPGWNLPLALQQKWVRDKETGCLSKVKELAVEARKPAVKDAESERALK
PNPKEASLPSSVSSKMKIIKNKNKNGRIVIVMSKYMDNNKVHGTKAKHGETSSDHKPQTTKPPENNPAHPTKIVEYPE
NGIPKEMCSGRTLPVAEHPMKCPPKDRHFSKPSPSTAEEYNTEVARGQADLPDDLPLQLTASSPVTSWAPDTNIPTQT
ALEHIRIPSFPKDRKRKLSDPAEHRSVSKTYLSARSLSAPSTAATPPQDTPMDLHCRGRRHSSTHEAADSGGQEEPMD
LSFPKTKKPAEPEVPPVAEPPVKAAQPDSADTQKTVKKAPAKKVSPFMGNIVITDITTNSLTVTFKEYISF

>Tni_ENSTNIG00000004608

MEIPAAGERVFAVESIEKRRTAPQGRVEYLVKWRGWSSRYNTWEPEENILDPGCWTPSGRVREHQEQLKRGPKAKHLL
IRVPSFARRSSVLAELREVSLEEGRCQAAKPVDMQYQLTSSKHQQYSPVRRERDGRGRSTGKPGYYQLNSKQQRPYQP
GLEVQGPLRAEAQDAEAPQPPANGYRLPPVLQQKWVRDKDSGCLTKVKDITTELQKLPADLSGPKGGRKVGCAEGGPP
RAEGLRSRKLKIVKNKNKNGRIVIVMSKFMEGGTHPANRRTGKLRSSPAAPRTTGRSPGLGGDGPEEKRRAPERDPAV
TEPDKDVKVKAQEQLPADPPSQPTGKANLTPHREAPPTRSQEEAKRDFRPQSDASPRPPAQQPPLQDPGDVDQEEPMD
LRVVRAVGAVGAVGAAWPEAQAAQQPDRLRPLLGNIVITDITTNCLTVTFKEYVAA

>Tni_Cbx6a (ENSTNIG00000018842)
MELSAAGDRIFAAEAILKRRVRKGQLEYLVKWKGWAMKHSTWEPEENILDDRLILGFEKKERERELHGPKKRGPKPKN
VAAKPFHAVGMAARVAVIHLPSSSSSSFAPSPKLNSLAATHKLKKDIHRCHRMSRRPLPRSDPMAGSFPNPGGLPSRL
HVSPFSETVRILNRRVKPREVKRGRIILNLKVIDKPGRGGSSVGSRNVAAGRQNIPSRNRIIGKKGEAPYRPFQPPLK
MLGFPMYGKPFGLHSSSSGGKSPPSASADPSQPSESPKSSPPDRETQPSPSSAPPDPAPASLPSSPSSSVDGEKDCAL
DSSSSSQECRKNSHQFRAKRPPAVGPGEQSAPPAESKRVPAEGDPDWHPEMAPSCTDVVVTDVTTNLVTVTIKEF

>Tni_Cbx7a (ENSTNIG00000006270)
MELSSIGDQVFAVESITKKRVRKGNVEYLLKWQGWPPKYSTWEPEDNILDPQLVLAYEENQERIRALAYRRKGLRPRK
LVPANIFAMDLRSAHKVKPSPRLRLSLTRAMSTDVEQGRTGRGHPKGPRRGPATAPSVPWPRRGPCRRTGLPPTKKKR
RPPREAPALCSSLQRSPLFPGACACPAAARRRHGRGPACGEGDKEMLGAAEESGPPSRGPAKYPGKVVTTKVTINALT
VTFREAAMAEGFFRGR

>Tni_Cbx7b (ENSTNIG00000012945)
MELSAIGEQVFAVEAIVKKRVRKGNVEYLLKWKGWPPKYSTWEPEEHILDQRLVQAYEEKEQRDRAVGHRRRGSRAKR
LLQVAGLSSQPPSPAQEDWEAGDQERPDSEDDDRPSFKSERAPTRTSEVTVTDVTLNALTVTFRESTRAKDFFREWEL
RV

>Tni_Cbx8a (ENSTNIG00000017995)
MELSAVGESVFAAESIMKRRIRRGRWEYLVKWKGWSQKYNTWEPEENILDERLFAAFEERERERELFGPKKRGPKPET
FLLKVRKAKAKERAYDFRGDAFRGIQVSYPVPEPVLTPRAREGLRAVVPTLFPPSAINRGESVYYVRSLEPESRYKATV
EHLPRKRGRKPKQSFDDEEDDGGASAEPYDKRSRAAEEAQSRRLHPRREAPDHGLIQLTRRFQEETTITPKPYSDQRR
VGGAGSTYSCSVGPDPRLRQPPHRREPQPGAFGDHDPAWTPRFSNHLDTVTVTDVTVNLLTVTVRESSSEKGFFRQKR

>Tni_ENSTNIG00000004609
MELSAVGERVFAAESIIKRRIRKGRLEYLVKWKGWSPKYSTWEPEENILDSRLFVAFEQRERERELYGPKKRGPKPKT
FLLKAQAKEKVKSYDFRSESVRGMHISYPTPEPVATPRAREGLRAVVPTIFPPSTVNRGESVRVQPPELCSRDLRVPP
LPAGPDALHFPKKRGPKPQCASRTAPRPGGVPVGQGGGGGAGEPQTRPVRQGEELRLLRVSNHGLGQTAPGGSYRRLY
DRGLHTHRRDTQTPRTQDGCSHAAPLKPASSTNPRRPTQLPLLDKPYLLDKPSPSPLDLDLDEVTWRPCLSSVEKVLV
TDVTSNFLTVTIKESSTSQGFFRAKR

>Mmo_Cbx2 (ENSMMOG00000020204)
MEELSAVGEQVFDAECILNKRLRKEKLEFLVKWRGWSLLIFHGVFNFVCREQEKELLIRKKGKRPRGRPRKILVITKS
SSSSSGSSSSCSDSSSSCSSSSSSSSDDDDDDEGQANQSVRTRDLHPVPQKKAQIVVAKQEPLKKRSRKPPSPDMNNGP
RKILKTLKDPDVRGAIKKPVHPASFTFMGFHRGPVRDPVGGQNRSPLTQGGAVKNLIGSVGSPSPNKSSQNRNVTEAK
PSVSSMSSGMSLDLKAAASKSKGVTAPNSNTSKSPIQGNTQHTLSSPNGPKKLQAPVSTLQRMPNTKAVASFPSKNIS
STQGSGLQPLNLQSKLAQNNDAAGNGPSPAAEIQSPRVQGRLEKSSMQKSSMELQQERSACKDGKKTTDMSTGEDESS
SDSDQDYTGQNRSVTVQNQDWKPTRNLIEHVFVTDVTANLVTVTVKESPTSVGFFSIRNY

>Mmo_ENSMMOG00000009201
MEGVTVGQVFDAECILSKRPRKGKFEYLVKWRGWSSKHNSWEPEENILDPRLLAAFHKREQELLVQKKGKRPRGRPRK
ILVTPPHLSGPLPPPTPTTSDPSLLFQPPEPAATEDGRSSSSSSSVLSSSPSCSSSEDEEHMKKAKAGPRVHPAPQKR
PHVLLAKAEPPRKKKRGRKPLHPDAKSRPPAPPPPPRHHQLFRAPREDPRGGVKKPLQPASFTYTGLSRGSRDEASAS
QTSGSSFSQGSASKPGSLGCLWSARSTSPXXXXXXXXXXXXAAQRSPLSPRRQDGVGGATGSVQHRQQSSSLSSVVPR
DRANQALSLRALNLQSVSSHQGNARSSLRSGLPAGSGAEQRGGREVLAGSTRHEERKHGPGNQGRVVNELSAGDSDET
SSSDLLEHVFVTDVTANFITVTVKESPTSVGFFNSRNH

>Mmo_ENSMMOG00000006960*
MELPAAGEHVFAVESIEKKRSRKGRVEYLVKWRGWSPKYNTWEPEENILDPRLLDAFQDRERQEQLMGYRKRGPKPKH
LLVQVPSFARRSSILADLHEASLDEDSCEKTPPIQMLRPQGQQYQLNSKKHHQYQPLCRERILCNQRGTAAAPLNNWR
TL

>Mmo_ENSMMOG00000007637*
MELPAAGEHVFAVEGIEKKRIRKVTGRVEYLVKWRGWSPKYNTWEPEENILDPRLLVAFQHR

>Mmo_Cbx6a (ENSMMOG00000011204)
MELSAAGERIFAAEAILKRRVRKGQLEYLVKWKGWAMKHSTWEPEENILDDRLILGFEQKERERELHGPKKRGPKPKN
FVMKTRTQKGETSSRVSNAPSSSSSSSSPFAPTPKLSSLAATHKLKKDIHRCHRMSRRPLPRFDPMANTFSNPGSFPS
RLHVSPFSETVRILNRRVKPREVKRGRIILNLKVIDKAGRGGTAAGSKNVMPGRQNIPSRNRIIGKKGDVPYRPFQPP
LKMLGFPMYGKPFGLQCGGPLPFHSHPGTCSTTGSRDTHSSSSQYQTPPSPSSSSSSEGKSPTNTSADQSQPTTGALL

SNKTPNSSTAHTKTQKSQSAKPPVPSSDADLTASTFLPSSPSSSLEGDREERVLDSSAPPEECRKNPRGRRAKRQLPV
SPGDQSSAPAEPKRVPAEGDPDWHPEMAPSCKDVVVTDVTTNLFTVTIKEFPSPASPSSSREGTSSPPPSTSSDDLSP
PKP

>Mmo_Cbx7b (ENSMMOG00000003559)
MELSAIGEQVFAVESIVKKRVRKGNVEYLLKWKGWPPKYSTWEPEEHILDQRLVQAYEEKEQRDRALGHRRKGSKAKR
LLQERTDEWSSTIGTDAVPASGRPDDDVWRPAVAPGEVTVTDVTLNSLTVTFRESRMAKGFFRDCGLEV

>Mmo_ENSMMOG00000002654*
MELSSIGDQVFAVESITKKRVRKGNVEYLLKWQGWPPKYSTWEPEDNILDPRLVLAYEEK

>Mmo_Cbx8a (ENSMMOG00000007636)
MELSAVGESVFAAESIIKRRIRRGRWEYLVKWKGWSQKYSTWEPEENILDARLFAAFEERERERELFGPKKRGPKPET
FLLKAKAKEKTYDFRGDPRRGIQVSYPIPEPVVTPRAREGLRTVVPTIFPPSAVNRGESVHVGPVEAERRPRPTPSAA
PTVHQSAQSPKKRGRKPKPPLPYDNDEGGCSAEPYAKRSRLQHHHHHHPHHGETSDHSLIQLTKRFQEETTITPRCCS
EHGLAGGAGLPYSDGRKGEKHRTGYGMSVPQPRKQERPEEHPWHQERECAPGPADVTTRSWTPCFTSADTVTVTDITM
NLLTVTVKENSTDKGFFRQNT

>Lcr_Cbx2 (Gene ID: 104923389)
MEELSAVGEQVFDAECILNKRLRKGKLEFLVKWRGWSSKHNSWEPQENILDPRLLAAFNKKEQEKELLIRKKGKRPRG
RPRKILENVPEAPKSSSSSSGSSSSSSDSSSSCSSSSSSSEDDDDDGGHVKQASPGVRTRDLHPVPQKKAQIVVAKQE
PPKKRSRKPLPPDVKEFQQNKGPRKILKTSKDIDLPGAIKKPVHPASFTFMGFHRGSPRDPVGGQNRSSLTQGGAVKN
SMSSVGSGRSVQPASPSLNKSSQSRNATEGKLSISSMNSGTSLDLKAAASKSKGVAALNLNTSKHPIQGTTQHTLSSP
NGQKKPQAPVSTLQRIPNTKPVASLPSKNASANQGSGLQPLNLQNKLVQSKDAPGNGTTPVSGLRNATNPARKTTVAQ
NQEYNPPKSPATPARLQARKNQPGADKVKEMTEIQTPRVQGRLEKSGVQKSSAEVQSQQERSAPKDGKKAKMNDMSTG
EDESSSDSDQDYAGENRTVAVQNQDWKPTRSLIEHVFVTDVTANLVTVTVKESPTSVGFFSIRNY

>Lcr_LOC104928297
MRDTYVTQLRGHTAGHSRISNNLCNAQRPPPGNANIKGKFEYLVKWRGWSSKHNSWEPEENILDPRLLAAFHKREQER
ELLFQKKGKRPRGRPRKILPPAPAAVKDSRSSSSSSGLSSSSSSSSEEEEDEEEEEEEVKKVKPGPRVAPVPQKRPQI
VMSKPDPPRKKKRGRKPLHPDLRALRQAKSRPSPPPPPPPPPPPPPPRHHQVLRLPRDEPRPGVKKPLQPASFTYTGLS
RTSRDEAASASQTSASSFSQTAASKSGSLGCIWSSRSMSPSTGASSYKSSPSPQSKNSLSELKRSVSDGGAGFKVSSL
KQGGGSGSLGLHGSFGGGQTVQRSPLSQRRQEGGGPVQHKQQNSSLSKPSSSPTPRDRASQALSLRVLNLQSVNTSSA
GNNTSGAGAAASRSSLRSGGMVVKGGPGSRTSAGGQRSGPAAGGGTDQARLREDRADAKKLTGSGSGRGNGGGRHEER
KHGLSSQNRSLNELSTGDSDETSSSESEHDAVLYPNNSRPSLGNDATESDTETDWRPARSLLEHVFVTDVTANFITVT
VKESPTSVGFFNSRNH

>Lcr_Cbx4 (Gene ID: 104921555)
MELPAAGEHVFAVESIEKKRSRKGRVEYLVKWRGWSPKYNTWEPEENILDPRLLDAFQDRERQEQLMGYRKRGPKPKH
LLVQVPSFARRSSILADLHEASLDDESCQKTSPITMLRPQGQQYQLNSKKHHQYQPLCRERENEQQANGKKFYYQLNS
KKHHHYQPDLKVHEPIFAKPREVKAPDLANKGYNLPPVLQQKWVRDKDSGCLTKVKDITMELKKLPADLNGHKEPEKV
KPKEDALAQSNGVSNSKLKIVKNKNKNGRIVIVMSKYMENGMQAAKIKNGNSEGAEKAPQGTDNSTENHLEKMRLVKK
LGLMNGFAKNLKDKPTVPSSGFNTDCPKEKEQSPKTEPTVTEQDKHVEVRGQGQLPADQPLQLTTKPNLLSLPLDRGV
PAPLDKRGNQGGFQGLKRHFSDTDYVEHGSSKRFLSSRSISAPNTVSSPTQSISIHQNGHQSHIGLQDCGYTDQEEPI
DLSIVKSRPKPAQPETHTQAETVTQAQTHTQDATDTQAQTQTDARTETQPQTETQKTTEEEKVDSSTVSNHEKRKEDT
FSSFQPFLGNIVITDITTNCLTVTFKEYITA

>Lcr_LOC104918816
MELPAAGEHVFAVEGIEKKRIRKGKIEYLVKWRGWSPKYNTWEPEENILDPRLLVAFQHRERQEQLMGYRKRGPKPKH
LLLQVPSFARRSSIPAGFEESSQDAEGTLKSDPVQFQRPQPQQYQLNSKKHHQYQPSNQEVPSDQLTNGKKKLIYQLN
SKKHHHYEPDLNMYDAQASRLKEVVKVQEPASKPANPGWNLPLALQQKWVRDKDTGCLSKVKELAVEVRKPAVKEAES
EHALKPNPKDATLPSAISSKMKIIKNKNKNGRIVIVMSKYMDSNKVHGAKGKHGESSSEEKPQNTKPSENNPAHTTKM
LEYPENGIPKEICNGSSLPAAEHPIKCSPKDRHFSKPSPSTAEEYNTEVARGQADLPDDLPLQLTASSPMTSWAVDTN
IPTPTAVDQIRIPSFPNDRKRKLSDPVEDRSVSKTYLTSRSFSVPSTVVTPPQDKPMDLHCSGPRHTSTCTYEVMDSG
SQEEPMDLSCRKTQKQVEPEVQPEPEPVVQNTPPVVEETEKSTEKSKEAPVKKISPFMGNIIITDITTNSLTVTFKEY
VSF

>Lcr_Cbx6 (Gene ID: 104925448)
MELSAVGDRVFAAEAILKRRVRKGQLEYLVKWKGWAMKHSTWEPEENILDDRLILGFEQKELHGPKKRGPKPKNFVMK
ARAQKGGTSSRATNARHATSRSSSSTSSRATPSSSASPHPLASSSSSSSSTAAPSPKLNSLAATHKLKKDIHRCHRMS
RRPLPRTDPMAAAFSNPGGFPSRVHVSPFSETVRILNRRVKPREVKRGRIILNLKVIDKPGRGGAAASSRNVTAGRQN
IPSRNRIIGKKGEAPYRPFQPPLKMLGFPMYGKPFGLQCGGPVAFHSHPGSCSSTGARDTHSTSSQYRSPPSPSSSSG
SEGKSPTNASAAQSQPSTGASPSSEASKSSKPHTETQKGQSTKAAPAPSSDPDSMASASPFLPSSPSSSLEDEGEEGA
PDRSVPPEGGRRNPRQRRAKHQLHAAPSSVTPGDQSTAPAEPKRVPAEGDPDWYPEMAPSCKDVVVTDVTTNLVTVTI
KEFPSPASCPSSPTASPEDASSPPPSTTSDDPSVPKP

>Lcr_LOC104932065

MELSSIGDQVFAVESITKKRVRKGNVEYLLKWQGWPPKCSTWEPEDNILDPRLVLAYEENQEKIRALAYRRKGLRPRR
LVLRNIFAMDLRSAHKEKPPPRLRLSLTRAMSTDVDQGERGSMYRRPAWRKSKQRASKRGPLGPSHKTIRQLRKKDEP
MEDDWCGTSEEEKQESESAAEERREDSLYGPSECSSPPLLERQDLEIEVEEKVDAELTVVGTETWTDSGTVETRQNQT
FVCDQSKAGPGDAVTASDRSDWYRSGEEEEEEEEEEQEEKAVESVSECPTLESSVCQSINNTTSVIVSVQGSSKTAGD
TAVATEVCSAVCSAAEVWKEEVRGDNQSVTTATQAAEHPEKVIVTNVTINSLTVTFKEATVAEGFFKGY

>Lcr_LOC104918881
MELSAIGEQVFAVESIVKKRVRKGNVEYLLKWKGWPPKYSTWEPEEHILDQRLVQAYEEKEQRDRALGHRRKGSKTKK
LLMQNTIYTMDLRSAHKSPEKPPPRLRLSLTRSLVSEDEDQPYSACRQDEHLQVAHRKSKPRRSQFKCLDSNPPSPTQ
EDWDGQGEEEEEDDEEEEEDNVEYEEDIEEEKESSPKEITERSVNGQKRTDEWKSAIGPDEVIASDKPDDDVWRPIIG
PGEVTVTDVTLNSLTVTFRESRVAKGFFRDWGLEV

>Lcr_Cbx8 (Gene ID: 104921554)
MELSAVGERVFAAESIIKRRIRKGRIEYLVKWKGWSPKYSTWEPEENILDSRLFAAFEQREREREELYGPKKRGPKPKT
FLLKAQAKVKAKSYEFRNEAVRGMHITYPTPEPVVTPRAREGLRAVVPTIFPPSTVNRGESVRVRPSEICAREHQQPP
LQQTSPDGLIHIPKKRGPKPKPRFKDSSCGPAVSEPHKRRAEEQANHIPHKLAKLQGGEEMRMVKMSHRHPENHGHSH
KHHHHHHHHHHHHSHNRGISGGGSYKPFYSERSLHPHRTDMDTHRTKDSSNYLAPPHFKHQSKMGQSLSRLADLPQM
EKPYFLDRPSPTRLDVNLDEVTWRPSLGNVEKILVTDVTTNFLTVTIKESSTSKGFFKDKR

>Lcr_LOC113747116
MELSAVGESVFAAESIIKRRIRRGRWEYLVKWKGWSQKYSTWEPEENILDARLFTAFEERERERELFGPKKRGPKPET
FLLKAKAKEKTYEFRGEAPRSIQVSYPIPEPIITPRAREGLRAVVPTIFPPSAVNRGESVHVRPPEPERRPRPTPPAA
LTVQESARLPKKRGRKPKLHLHYEKDDGGSSAELDDKRSRLLEEPSSHGLSKMPRRLHHHGETSDHTLIQLTKRFQEE
TTITPKSCGEQRHAGLSYSRTFKSDQGGHRTSYVSKMPIPQPRKLKHSAESRRHQMKECRPGPAVIPTQAEAILDPSA
RPTSSWTPRFTNVDSVTVTDVTMNFLTVTVRESSTDKGFFRESR

>Pfl_Cbx2 (Gee ID: 114569523)
MEELSAVGEQVFDAECILNKRLRKGKLEFLVKWRGWSSKHNSWEPQENILDPRLLAAFNKKEQEKELLMRKKGKRPRG
RPRKILENVPEAPKSSSSSSGSSSASSDSSSSCSSSSSSSDDDDDDDDDDNGHAKKASPGVRTRDLHPVPQKKAQIVV
AKQEPPKKRSRKPQSTEAKEFPQNKGPRKILKSSKDTDLPGAIKKPVHPASFTFMGFPRGLPRDTLGGQNRSPLTQGG
AVKNSVSTVGSCRSVQPASPSLNKSSQSRNVAEGKLSISSMSSGTSLDLKAATGKSKGVAALNLNASKHLVQGSTQHT
LSSPNGQKKPQAPVSTMQRIPITKAVASLPSKNASSNQGSGPQPLNLQNKLVQSNDAPGNGTTPTSGLRNATNPARKT
TVSQNQEYNSPKSPATPGRLQARKSQPGADKAREATEIQTPRVQGRLDKSSVQKSSTEVQSQQERSASKDKKAKMNDM
STGEEESSSDSDQDPSYVGQDRSVAVQNQDWKPTRSKLEHVFVTDVTANLVTVTVKESPTSVGFFSIRNY

>Pfl_LOC114548436
MEGVTVGQVFDAECILSKRPRKGKFEYLVKWRGWSSKHNSWEPEENILDPRLLAAFHKREQERELLFQKKGKRPRGRP
RKILPPLPASTKDSRSSSSSSSGLSSSASSSSSEDEDHTKKAKPGPRVLPVPQKRPQIVLAKPDPPCKKKRGRKPLHP
DLRALRQAKSGPPPLPTLPPSPPPLPPPPPPPLPPRHHQPHHQVLRPSRDDPRPGVKKPLQPASFTYTGLSRASREEA
SSASRTSASSFSPAPSKPGSLGCVWTSRSMSPSSSSYSKTSSPQRNSLSEGPRYKASGSGSLGSHGSFVGGQAAVQRS
PLSQRRQEGPAAGPHRHQNLAKPGPPRDRANQNQALSLQSVVGKAPGASCLAGNSASAASRSSLRSGSVVVKGGASKS
AKGTRTPGSGGHQRPGLAPGGGGGGGSDLGRSRDDRGEEAPAGSVSGGRLEDRKRLALAAQNRSLNELSTGDSDETSS
SQSERDEASPFPNNNNNNNGGGGGGGGGPRLGNESDTETDWRPARSLLEHVSVTDVTANFITVTVKESPTSVGFFNAR
NH

>Pfl_Cbx4 (Gene ID: 114546081)
MELPAAGEHVFAVESIEKKRSRKGRSEYLVKWRGWSPKYNTWEPEENILDPRLLDAFQDRERQEQLMGYRKRGPKPKH
LLVQVPSFARRSSILADLHEASLDEDSCQKPSPIQMLRPQGQQYQLNSKKHHPYQPLCRERDAEQQAHGKKLYYQLNS
KKHHHYQPDLNGHEPLFAKPREVKAPELANKGYNLPPVLQQKWVRDKDSGCLTKVKDITMELKKLPADLNGHKEPETA
RPKEDALAQSNGVSNGKLKIVKNKNKNGRIVIVMSKYMENGMKAAKVKNGNSETGEKPAQGTDTGAENHLEKMKLIKK
LGLMNGFSKNPKDKPRVSSSGLNGHCPKEKEQSPKSEPTVTEQDKHVGVGGQGQLSADQPLQLTTEPNLLALPLDGGV
PSPLDKRGSQGGFQGLKRHLCDTDSEEHGSSKRFGTSRSTGAANTVSSPTQSVSMDQNGHQSPIGLQDCGYVDQEEPI
DLSIVKSRPKATQPETHPQAQTVTQAKTHTRDETDTQAQTQTDAQTESQPRTETQKTTEEENVTSLSVSDPEKRKEET
FPSFQPFLGNIVITDITTNCLTVTFKEYIKV

>Pfl_LOC114569420a
MELPAAGEHVFAVEGIEKKRIRKGKIEYLVKWRGWSPKYNTWEPEENILDPRLLVAFQHRESQEQLMGYRKRGPKPKH
LLLQVPSFARRSCIPAGFEESSQDTEVNVKSDPVPVQRPQPQQYQLNSKKHHQYQPSSQEVPADQPTNGKKKFIYQLN
SKKHHHYEPDPNMYDAQASRLKEVVKVQELASKPANPGWNLPLALQQKWVRDKDTGCLSKVKELEVEVRKPAVKDAKS
EQALKPNPKQATLPSAVSSKMKIIKNKNKNGRIVIVMSKYMDSNKVHGAKGKHGESSSEVKHQNTKPLENNPAHTTKM
MEHPENGIPKELCNGSSLPAAEHPLKCSPKDRHFSKPSPSTAEEYNTEVARGQADLPDDLPLQLTASSPLTSWAVDTN
IATPTSVDQIRIPSFPNDRKRKLSDHVEDRSVSKTYLTSRSFSVPSTVVMPTQDKPMDLHCSGPRHSSTCTYEVVDSG
SQEEPMDLSCPKTKNQVELEIQPEPELVVTNTPPVTEDTQKSTEKSKEAPVEKTSPFMGNIIITDITTNSLTVTFKEY
GMDEDAINKAFEETSVGIYVIKEHASSDEPGDIGIVLEGIKMLQDLYNVALPVATLFGLMGGSVDRSDFDCPTVPDIP

>Pfl_Cbx6 (Gene ID: 114569708)

MKHSTWEPEENILDDRLILGFERKEREREMHGPKKRGPKPKNFVMKAHAQKGGTSSRASNARQTTSRSSSSTSSRATP
SSSASPHLASSSSSSSSVAPSPKLNSLAATHKLKKDIHRCHRMSRRPLPRSDPMASSFSNPGGFPSRLHVSPFSETVR
ILNRRVKPREIKRGRIILNLKVINKPGRGGTAAGGRNLTEGRQNIPSRNRIIGKKGDTPYRPFQPPLKMLGFPMYGKP
FGLQCGGPLSFHSHPGSSTGARNTQPTSSKHQSPSSPSSSASAAQSQPATGGSPSSEGAKSSKPHAESQKGQSASRAA
TAPSSDANPLASASSFLPSSPSSSLEDVEDEDDAPELSAAPEGGRKSTRQRGAKRQPPAASGTPGDQIAAPTEPQRVP
AEGDPDWHPEMAPSCKDVVVTDVTTNLVTVTIKEFPSPASPSASPESASSPPPSTTSDDVSPPKP

>Pfl_Cbx7 (Gene ID: 114548596)
MELSAIGEQVFAVESIVKKRVRKGNVEYLLKWKGWPPKYSTWEPEEHILDQRLVQAYEEKEQREKTLGHRRKGPKGKR
LILQDTVYTMDLRSAHKTPEQPGPRLRLSLTRSLLPGDEEGQSYGACRHEPQLAHPGSRPSRSQFMCLDSNPPSPTQE
DWEGRGEDEEEEEDNVESEEDGEEEEEEKEEALKESSEKSGGTLNGQERTDGWSSALGPDGLTASEKPDDAWRPIVRP
GDVTVTDVTLNSLTVTFRESRVAKGFFRDWGPGGLICDV

>Pfl_LOC114570306
MELSSIGDQVFAVESITKKRVRKGNVEYLLKWQGWPPKYSTWEPEENILDPRLVPAYEENQEKIRALAYRRKGLRPRR
LVLRNIFAMDLRSANKALEKPPPRLRLSLTRSMSTDVDQGEQSSLYRGLARRKSKQRVSKRGPAGPSNKTTFPLEKLE
EPIEEDWGVTSEEEKHESESITEERSKDRLYGQSECGSPPLLERQDLAMDVEEKEDADLTAVGTETWIDRPGGGTSKT
RQNQTFACDQSKDSVSVPEAIPEDVVSVSDRSDWDLGEESVESEGLEIRLESSVCQSSSTTSVIVSVQGSGKTASECS
PTEARKEEGSDDSPSVTTTAPGAQTAPVAAEQPGNVISTDVTINSLTVTIKEAVVAEGFFKGY

>Pfl_Cbx8 (Gene ID: 114569533)
MELSAVGERVFAAESIIKRRIRKGRIEYLVKWKGWSPKYSTWEPEENILDSRLFVAFEQREREREL YGPKKRGPKPKT
FLLKAQVKAKSYEFRGDAVQGMHITYPTPEPVVTPRAREGLRAVVPTIFPPSTVNRGESVRVRPPELSTREYRQPSLQ
HIGPDGLLHVPKKRGPKPKPRFKDSSCSPVVSEPHKRRAEEQVSHSHHKLAKLQGGEETRLIKVSHRHPESHGHKPHH
HHHHHHHHHLTHNRGSSYKQLYSERSLHPHRTDPDTHRTKDSSTYLAPVHFKHQSKMSQGSSRPAELPQMEKPYFLD
RPSPTRLDVDLDEVAWRPSIGDVEKVLVTDVTTNFLTVTIKESSTSKGFFKDKR

>Pfl_LOC114569420b
MELSAVGESVFAAESIIKRRIRRGRWEYLVKWKGWSQKYSTWEPEENILDARLFAAFEERERERELFGPKKRGPKPET
FLLKAKAKEKAYEFRREAPRGIQVSYPIPEPVITPRAREGLRAVVPTIFPPSAVNRGESVHIRPPEPERRPRPTPTAS
LTVREAVRFPKKRGRKPKLQLHYEKDHEGSSAEPDTKRSRSLEEPVSHGLSNMSRRLFHHGETSDHSLIQLTRRFQEK
TTITPKHSREQRHAGLSYSCAFSPDVLKSDQGGDRTQCLSRMSIPRPSSSAEHRRHQVKECCLPPEQPAVISTQSESI
HDQSTRPASSWTPSYTNLDTVTVTDVTMNFLTVTVRESRTEKGFFREKR

>Sdu_LOC111237087
MEGVTVGQVFDAECILSKRPRKGKFEYLVKWRGWSSKHNSWEPEENILDPRLLAAFHKREQERELLFQKKGKRPRGRP
RKILPAPTATKDSRSSSSSSSGLSSSASSSSSEDEDHTKKAKRVHPVPQKRPQILLAKPDPPRKKKRGRKPLHPDLRA
LRQAKSRPPAPSPPPPPPPPPPPPPRHHQALRLLRDEPRPGVKKPLQPASFTYTGLSRSSRDEAASASQTSASSFSQ
TAASKPGSLSCIWTNRSLSSSSSSSSHNKASPSAQNKNSASELKRSMSESGSRGDGFKVSPLKQSGSSGSLGLHSSFG
GGQAVAQRSQLGQRRQEGVGGQSGLVQHKQQNSGLSKPSSLSSPTPRDRANQALSLRALNLQSVSKLPAGSSTSSAMV
AASRSSLRSGGGIVVKGGVGSIKETRMSAGGQRSGLAAGGTAEPGRGKEMMAGGSSSRGSGGGGRQEDRKHGLSSQNR
SLNELSTGDSDETSSSESEHDDTALYPSKSRPSLGNDATESDTETDWRPTRSLLEHVFVTDVTANFITVTVKESPTSV
GFFNSRNH

>Sdu_LOC111217430
MEELSAVGEQVFDAECILNKRLRKGKLEFLVKWRGWSSKHNSWEPQENILDPRLLAAFNKKEQERELLLRKRGKRPRG
RPRKILENVPEAPKSSSSSSGSSSSSSDSSSSCSSSSSSSDDDDEDSSVKQATPSVRTRDLHPVPQKKAQIVVAKQEP
PKKRSRKPLPPEVKEFQQNKSPRKILKTSKDTDLPGAIKKPVHPASFTFMGFHRGSARDTVGSQNRSSLAQGGAVKNS
MSSVGSGRSVQPASLSLNKSSQSRNVTEGKLSISSMSSGTSLDLKAAAGKSKGVAALNLNTSKHPIQGTTQHTLSSPN
GQKKPQGPVSTLQRIPNTKAVASVPSKNASSNQGSGLQPLNLQSKPMQSNDAPGNGTTPVSGVRNATNPARKTTVTQN
QEYNPKSPATPGRLQARKNQPGADKVKEANEIQTSRVQGRLEKNSVHKSSTEVQSQQERSAYKDGKKAKMNDMSTGEE
ESSSDSDQDSSYTGQDRSVAVQNQDWKPTRSLIEHVFVTDVTANLVTVTVKESPTSVGFFSIRNY

>Sdu_Cbx4 (Gene ID: 111218007)
MELPAAGEHVFAVESIEKKRSRKGRVEYLVKWRGWSPKYNTWEPEENILDPRLLDAFQDRERQEQLMGYRKRGPKPKH
LLVQVPSFARRSSILADLHEASLEEDGCQKSSAIQMLRPQGQQYQLNSKKHHQYQPLCREREAEQQTNGKKFYYQLNS
KKHHHYQPDLKVHEPIFVKPRDIRAPELPNKGYNVPPMLQQKWVRDKDSGCLTKVKDITMELKKLPADLNGHKEPEKV
KPKEDALPQSNGVSSSKLKIVKNKNKNGRIVIVMSKYMENGMQAAKIKNGDSETPEKPPKGTDNSAENDLEKMKLVKK
LGLMNGFAKNPKEKPTIPSSGFNVVCPKEKEQSPKMEPTVTEQDKHVEVRGQGQLPADQPLQLTTKPNLLSLPLDRGV
SSPLDKRGAQGGFQGLKRHLSDTDCDEHGSNKRFLSSRSISVPNTVSSPSQSISIDQNGHQSHTGLQLCGYADQEEPI
DLSIVKTRPKAAASTATQPERHTQAETLTQTQDETHTQAETHTQMDTQTEAQPQTETQKSAEKVKVDSLSVSNNEKRK
EETFPSFQPFLGNIVITDITTNCLTVTFKEYVTV

>Sdu_LOC111227040
MELPAAGEHVFAVEGIEKKRIRKGKIEYLVKWRGWSPKYNTWEPEENILDPRLLVAFQHRERQEQLMGYRKRGPKPKH
LLLQVPSFARRSSIPAGFEETCQDAEGGLKSDPVQVQRSQPQQYQLNSKKHHQYQPSSQEVPADQLTNGKKKFIYQLN
SKKHHHYEPDPNMFDTQASRLKEVVKVQESACKPANPGWNLPLALQQKWVRDKDTGCLSKVKELAVEVRKPAVKEAES

EHALKPNPKDATLPSAISSKMKIIKNKNKNGRIVIVMSKYMDSNKVHGAKGKHGESSSEDKPQNTKPSENNPAHRTKM
AEHPENGIPKEICNGSSLPAAEHPIKCSPKDRHFSKPSPSTAEEYNTEVARGQADLPDDLPLQLTASSPLMSWAVDTN
IPTPTAVDQIRIPSFPNDRKRKLSEPVEDRSVSKTYLTSRSFSVPSTVVTPPQDKPMDLQCSGRRASSACTYEVMDPG
SQEEPMDLSCPKTKRQVEPEIQPEPAPEPEPAVRDTPPITEDTQKSTEKSKEAPVKKISPFMGNIIITDITTNSLTVT
FKEYVSF

>Sdu_Cbx6 (Gene ID: 111216565)
MELSAAGDRIFAAEAILKRRVRKGRIEYLVKWKGWAMKHSTWEPEENILDDRLILGFEQKERERELHGPKKRGPKPKN
FVIKARSQKREPSRQATSTRQTTSRSSLSTSSRAAPSSSASPHHLPSSSSSSSTVAPSPKLNSLAATHKLKKDIHRCH
RMSRRPLPRSDPMAPTFSNPGGFPSRMHVSPFSETVRILNRRVKPREVKRGRIILNLKVIDKPGRGGTAAGSRNITTG
RQNIPSRNRIIGKKGEAPYRPFQPPLKMLGFPMYGKPFGLQCGGPVPFHPHTGSCSSTGARDSNTTPSQYQSPPSPAS
SSGSEGKSPTASAAQSKPATGASPSSEGPKPSKPHAETQKGQSAKNATPAPSSDANPVVSASPFLPSSPSSSLEDEEE
EGAVDRSVPSEGGRKNLRQRRAKRQPPTTLPSVAPGDQSSAPTEPQRVPAEGDPDWHPEMAPSCKDVVVTDVTTNLGT
VTIKEFPSPASCPASPSSSPENASSPPPASTSDDPSPAKP

>Sdu_Cbx7 (Gene ID: 111219914)
MELSAIGEQVFAVESIVKKRVRKGNVEYLLKWKGWPPKYSTWEPEEHILDQRLVQAYEEKEQRDRALGHRRKGSKSKR
VLLQNTVYTMDLRSAHKTPEKPSPRLRLSLTRSLDEDEDEDEDEPYSACRQGPRSQVAHHKGKPSRSQFRCLDSNPAS
PTQEDWEGLEDEEEEEEEEEEEEGEEEEEEEEEDNVEDEEDREEEETEKTDEWSSAIVPDGVTASDKSDDVWRPVISA
GEVTVTDITLNSLTVTFRESRVAKGFFRDWGLEV

>Sdu_LOC111230359
MELSSIGDQVFAVESITKKRVRKGNVEYLLKWQGWPPKYSTWEPEDNILDPRLVLAYEENQEKIRALAYRRKGLRPRR
LVLRSIFAMDLRSAHKDAEKTPPRLRLSLTRSMSTDVDQGERGSMYHRSVRRKSRQRVAKRRQDGPSNKPIRPLRKKE
EPMEEDWSGISEEEKQESESTTEDRREDSLYGQSECSSPPLLERQDLEMEAEEKVDADLTAWTDGPGGGTSETTQSQT
LACNQSKDSASVPVTGPGDAVTVGDRSDWDTGEEGVESGSECPRLERINTTSVIVSVQASGETEGADAAAAAAAAAA
AAAADACSTAEVRNEEVGGDNQSVTTTTPGSQSDQTTAEHPGKVIVTDVTINSLTVTFKEAMVAEGFFKGY

>Sdu_Cbx8 (Gene ID: 111217943)
MELSAVGERVFAAESIIKRRIRKGRIEYLVKWKGWSPKYSTWEPEENILDSRLFAAFEQRERERELYGPKKRGPKPKT
FLLKAQAKVKAKSYEFRSEAVRGMHVTYPTPEPVVTPRAREGLRAVVPTIFPPSTVNRGESVRVRPPELSTREHQQSS
LQQNSPDGLLHVPKKRGPKPKPRFKDSSYSPAAPEPHKRRAEEQLSHGPHKLAKLQGVEEMRLFKVAHRHPENHGHSH
KHHHHHHHHHHHHHHHQQNRELSGGSSYKQLYSDRSLHPHRIDIDTHRTKEGSSYLAPAHFKHQPKMSQSVSRPAELPL
MEKPYFLDRPSPTRLDDDLDEVSWRPSLGSVEKVLVTDVTTNFLTVTIKESSTSKGFFKDKR

>Sdu_LOC111227041
MELSAVGESVFAAESIIKRRIRRGRWEYLVKWKGWSQKYSTWEPEENILDARLFAAFEERERERELFGPKKRGPKPET
FLLKAKAKAKEKTYEFRREAPRAIQVSYPIPEPVITPRAREGLRAVVPTIFPPSAVNRGESVHVRPPEPERRPRPTPP
AALTLQESTGFPKKRGRKPKLQLHYDQDDGCSSAEPDTKRSRFLEEPVSHGLSKMTRRLHHHGETSDHSLLQLTKRFQ
EETTITPKSSCEQRHLVGAGLSYTCAFSPDKARKSDHMGHRTHFLSRVSVPQAGKLKHSAEHRSHQLKQYCLPREQPA
VISTEAESIHDETARSASSWTPSFTNLDTVTVTDVTMNFMTVTIRESSTDKGFFSDKR

Sla_LOC111655533*
MSIYFRHNSWEPQENILDPRLLAAFNKKEQERELLLRKRGKRPRGRPRKILENVPEAPKSSSSSSGSSSSSSDSSSSC
SSSSSSSDDDDEDSSVKQATPSVRTRDLHPVPQKKAQIVVAKQEPPKKRSRKPLPPEVKEFQQNKSPRKILKTSKDTD
LPGAIKKPVHPASFTFMGFHRGSARDTVGSQNRSSLAQGGAVKNSMSSVGSGRSVQPASLSLNKSSQSRNVTEGKLSI
SSMSSGTSLDLKAAAGKSKGVAALNLNTSKHPIQGTTQHTLSSPNGQKKPQGPVSTLQRIPNTKAVASVPSKNASSNQ
GSGLQPLNLQSKPMQSNDAPGNGTTPVSGMRNATNPARKTTVTQNQEYNPKSPATPGRLQARKNQPGAEKVKEANEIQ
TSRVQGRLEKNSVHKSSTEVQSQQERSAYKDGKKAKMNDMSTGEEESSSDSDQDSSYTGQDPSVAVQNQDWKPTRSLI
EHVFVTDVTANLVTVTVKESPTSVGFFSIRNY

>Sla_LOC111664318
MEGVTVGQVFDAECILSKRPRKGKFEYLVKWRGWSSKHNSWEPEENILDPRLLAAFHKREQERELLFQKKGKRPRGRP
RKILPAPTATKDSRSSSSSSSGLSSSASSSSSEDEDHTKKAKRVHPVPQKRPQILLAKPDPPRKKKRGRKPLHPDLRA
LRQAKSRPPAPSPPPPPPPPPPPPPPPRRHHQALRLLRDEPRPGVKKPLQPASFTYTGLSRSSRDEAASASQTSASSFS
QTAASKPGSLSCIWTNRSLSSSSSSSSHNKASPSAQNKNSASELKRSMSESGSRGDGFKVSPLKQSGSSGSLGLHSSF
GGGQAVAQRSQLSQRRQEGVGGQIGLVQHKQQNSGLNKPSSLSSPTPRDRANQALSLRALNLQSVSKLPAGSSTSSAM
VAASRSSLRSGGGIVVKGGVGSIKETRMSAGGQRSGLAAGGTAEPGRGKEMMAGGSSSRGSGRQEDRKHGLNSQNRSL
NELSTGDSDETSSSESEHDDTALYPSKSRPSLGNDATESDTETDWRPTRSLLEHVFVTDVTANFITVTVKESPTSVGF
FNSRNH

>Sla_LOC111667573
MELPAAGEHVFAVEGIEKKRIRKGKIEYLVKWRGWSPKYNTWEPEENILDPRLLVAFQHRERQEQLMGYRKRGPKPKH
LLLQVPSFARRSSIPAGFEETCQDAEGGLKSDPVQVQRSQPQQYQLNSKKHHQYQPSSQEVPADQLTNGKKKFIYQLN
SKKHHHYEPDPNMFDTQATRLKEVVKVQESACKPANPGWNLPLALQQKWVRDKDTGCLSKVKELAVEVRKPAVKEAES
EHALKPNPKDATLPSAISSKMKIIKNKNKNGRIVIVMSKYMDSNKVHGAKGKHGESSSEDKPQNTKPSENNPAHRTKM
AEHPENGIPKEICNGSSLPAAEHPIKCSPKDRHFSKPSPSTAEEYNTEVARGQADLPDDLPLQLTASSPLMSWAVDTN

IPTPTAVDQIRIPSFPNDRKRKLSDPVEDRSVSKTYLTSRSFSVPSTVVTPPQDKPMDLQCSGRRASSACTYEVMDPG
SQEEPMDLSCPKTKRQVEPEIQPEPEPAPEPEPAVRDTPPITEDTQKSTEKSKEAPVKKISPFMGNIIITDITTNSLT
VTFKEYVSF

>Sla_LOC111655477
MELPAAGEHVFAVESIEKKRSRKGRVEYLVKWRGWSPKYNTWEPEENILDPRLLDAFQDRERQEQLMGYRKRGPKPKH
LLVQVPSFARRSSILADLHEASLEEDGCQKSSAIQMLRPQGQQYQLNSKKHHQYQPLCREREAEQQTNGKKFYYQLNS
KKHHHYQPDLKVHEPIFVKPRDIRAPELPNKGYNVPPMLQQKWVRDKDSGCLTKVKDITMELKKLPADLNGHKEPDKV
KPKEDALPQSNGVSSSKLKIVKNKNKNGRIVIVMSKYMENGMQAAKIKNGDSETPEKSPKGTDNSAENDLEKMKLVKK
LGLMNGFAKNPKEKPTIPSSGFNVVCPKEKEQSPKMEPTVTEQDKHVEVRGQGQLPADQPLQLTTKPNLLSLPLDRGV
SSPLDKRGAQGGFQGLKRHLSDTDCDEHGSNKRFLSSRSISVPNTVSSPSQSISIDQNGHQSHTGLQLCGYADQEEPI
DLSIVKTRPKAAASTATQPERHTQAETLTQTQDETHTQAETHTQMDTQTEAQPQTETQKSAEKVKVDSLSVSNNEKRK
EETFPSFQPFLGNIVITDITTNCLTVTFKEYVTV

>Sla_LOC111673425
MELSAAGDRIFAAEAILKRRVRKGRIEYLVKWKGWAMKHSTWEPEENILDDRLILGFEQKERERELHGPKKRGPKPKN
FVIKARSQKREPSRQATSTRQTASRSSLSTSSRAAPSSSASPHHLPSSSSSSSTVAPSPKLNSLAATHKLKKDIHRCH
RMSRRPLPRSDPMAPTFSNPGGFPSRMHVSPFSETVRILNRRVKPREVKRGRIILNLKVIDKPGRGGTAAGSRNITTG
RQNIPSRNRIIGKKGEAPYRPFQPPLKMLGFPMYGKPFGLQCGGPVPFHPHTGSCSSTGARDSNTTPSQYQSPPSPAS
SSGSEGKSPTAPAAKSKPATGASPSSEGPKPSKPHAETQKGQSAKNPTPAQSSDANPVVSASPFLPSSPSSSLEDEEE
EGAVDRSVPSEGGRKNLRQRRAKRQPPTTLPCVAPGDQSSAPTEPQRVPAEGDPDWHPEMAPSCKDVVVTDVTTNLGT
VTIKEFPSPASCPASPSSSPEN

>Sla_LOC111646103
MELSAIGEQVFAVESIVKKRVRKGNVEYLLKWKGWPPKYSTWEPEEHILDQRLVQAYEEKEQRDRALGHRRKGSKAKR
LLLQNTVYTMDLRSAHKTPEKPSPRLHLSLTRSLVPEDEDEDEDEDEDEDEDEDEDEDEDEDEDEDESYTACRQGPRP
QVAHHKVKPSRSQFRCLDSNPASPTQEDWEGLEDEEEEEGEEEEEEEEEDNVEDEEDREEEETEVAQKDTTEKSGGIH
NGQEKTDEWRSAIVPDGVTASEKSDDVWRPVISAGEVTVTDITLNSLTVTFRESRVAKGFFRDWGLEV

>Sla_LOC111647569
MELSSIGDQVFAVESITKKRVRKGNVEYLLKWQGWPPKYSTWEPEDNILDPRLVLAYEENQEKIRALAYRRKGLRPRR
LVLRSIFAMDLRSAHKDAEKTPPRLRLSLTRSMSTDVDQGERGSMYHRSVRRKSRQRVAKRRQDGPSNKPIRPLRKKE
EPMEEDWSGISEEEKQESESTTEDRREDSLYGQSECSSPPLLERQDLEMEAEEKVDADLTAWTDGPGGGTSETTQSQT
LACNQSKDSASVPVTGPGDAVTVGDRSDWDTGEEGVESGSECPRLERINTTSVIVSVQGSGETEGADTAAAAAAAAAA
AAAAAAAAAAAADACSTAEVRNEEVGGDNQSVTTTTPGSQSDQTTAEHPGKVIVTDVTINSLTVTFKEAMVAEGFFKG
Y

>Sla_Cbx8 (Gene ID: 111655455)
MELSAVGERVFAAESIIKRRIRKGRIEYLVKWKGWSPKYSTWEPEENILDSRLFAAFEQREREREL YGPKKRGPKPKT
FLLKAQAKVKAKSYEFRSEAVRGMHVTYPTPEPVVTPRAREGLRAVVPTIFPPSTVNRGESVRVRPPELSTREHQQPS
LQHNSPDGLLHVPKKRGPKPKPRFKDSSYSPAAPEPHKRRAEEQLSHGPHKLAKLQGVEEMRLFKVAHRHPENHGHSH
KHHHHHHHHHHHHHHQQNRELSGGSSYKQLYSDRSLHPHRIDIDTHRTKEGSSYLAPAHFKHQPKMSQSVNRPAELPL
MEKPYFLDRPSPTRLDDDLDEVSWRPSLGSVEKVLVTDVTTNFLTVTIKESSTSKGFFKDKR

>Sla_LOC111667559
MELSAVGESVFAAESIIKRRIRRGRWEYLVKWKGWSQKYSTWEPEENILDARLFAAFEERERERELFGPKKRGPKPET
FLLKAKAKAKEKTYEFRREAPRAIQVSYPIPEPVITPRAREGLRAVVPTIFPPSAVNRGESVHVRPPEPERRPRPTPP
AALTLQEPTEFPKKRGRKPKLQLHYDQDDGCSSAEPDTKRSRFLEEPVSHGLSKMTRRLHHHGETSDHSLLQLTKRFQ
EETTITPKSSGEQRHLVGAGLSYTCAFSPDKARKSDHMGHRTHFLSRVSVPQPGKLKHSAEHRSHQLKQYCLPREQPA
VISTEAESIHDETARSASSWTPSFTNLDTVTVTDVTMNFLTVTIRESSTDKGFFTDKR

>Lbe_LOC114919638
MEGVTVGQVFDAECILSKRPRKGKFEYLVKWRGWSSKHNSWEPEENILDPRLLAAFHKREQERELLFQKKGKRPRGRP
RKIQPPAPAATKDSRSSSSSSSGLTSSASSSSSEEDDEEDHTKKVKAAPRVHPVPQKRPQILLAKPDPPRKRGRKPLP
PDLRALRQAKSRPSPPPPPPPPPPPPRHHQVLRPSREETRPGVKKPLQPASFTYTGLSRSSREEAAAAASQTSSSSFS
QTAASKSGSLSGMWTSRSMSPSSSYKTSSSSHSKNSLSELKRSHSDTSGGRDGFKASSLKQGGGGAMMHGGFGQTAPR
SSLSQRRQEGGGGQTASLQHKQQNSSQSKMASSLTPRDRASQALNLRALNLQSVGKTAAGSNAAVASRTSLRSGGVMV
KAGGLKDTRAASVQRSSVPAGGAAEQGRAREERGRETSAEGKKLAGSSGGRHEERKHNVQTRSLNELSTGDSDETSSS
ESEHGADLFPNNSRPSFGDDATESDTETDWRPAHSLLEQVFVTDVTANFITVTVKESPDQCRIL

>Lbe_LOC109999250
MELPAAGEHVFAVESIEKKRSRKGRVEYLVKWRGWSPKYNTWEPEENILDPRLLDAFQDRERQEQLMGYRKRGPKPKH
LLVQVPSFARRSSILADLHGATLDDDSCKKTSPIQMLRPQSQQYQLNSKKHHQYQPLCREREAEQQQQATGKKFYYQL
NSKKHHHYQPDLKVHEPMFAKPKEVKAPELANKGYNLPPVLQQKWVRDKDSGCLTKVKDITMELKKLPADLNGEPERV
KPKEDAVTQSNGVSSSKLKIVKNKNKNGRIVIVMSKYMENGMKATKIKNGDSGSAEKHLQRTDSSAEEHLEKMRLVKK
LGLMNGFAKKLKDKSTVLTSGFSRDHPREKEQSPKTEPTVTERDKHVEVRGQGQLPKDQPLQLTTKSNLLPLPLDRGV
PHSADIQGSQGGFQGLKRHFSDSDSEEHGSSKRFFSTRSISTPHPVSSPAQSVSVAQNGHQSPIGLPVCEYTDQEEPM

DLSIVKSRHCPTRAETHKQTETVTHTETSTQEETDTQAMTHVAQTETEHQTETENATGEEKVNSLSVTHHEKKTDDSF
PSFKPFLGNIVITDITTNCLTVTFKEYIAV

>Lbe_LOC109985836
MELPAAGEHVFAVEGIEKKRIRKGKIEYLVKWRGWSPKYNTWEPEENILDPRLLVAFQHRERQEQLMGYRKRGPKPKH
LLLQVPSFARRSSIPAGFEESSQDPEGVLKSDPVQVQRPQPQHYQLNSKKHHQYQPSSQEVPADQLHNGKKKFIYQLN
SKKHHHYEPDLNMYDAQASRLKEVVKVQEPASKPANPGWNLPLALQQKWVRDKDTGCLSKVKELEVRKPAVKDAESEH
ALKPNPKDATLPNAVSSKMKIIKNKNKNGRIVIVMSKYMDSNKVHGAKGKHGESSSVEKPQNTKPSENNPAHTTKMVA
YPENIIPKEICNGSSPPAAEHPIKCSPKDRHFSKPSPSTAEEYNTEVARGQADLPEDLPLQLTASPPPTSWVVDTNIP
TPTDNDQIRIPSFPNDRKRKISDTTDDRSVCKSYLTSRSFSVPSTVVAPPQDSPMDLHCSGPRHSGTRTYEALDSGSQ
EEPMDLSCPKTKKQAEPERQPEPEPVVENTTPVSEETHEPTEKSKEAPVKKPSPFMGNIIITDITTNSLTVTFKEYVS
F

>Lbe_Cbx6 (Gene ID: 109991257)
MELSAVGDRVFAAEAILKRRVRKARLEYLVKWKGWAMKHSTWEPEENILDDRLILGFEQKERDRELHGPKKRGPKPKT
LAMKARGRKGGNSSRISNSRQTTSCSSVSTSSRATPSPSTSAAPHRLGSSSSSSSTVAPSPKLSSLAATHKLKKDIHR
CHRMSRRPLPRSDPMASSFSNPGGFPSRPHISPFSETVRILNRRVKPREVKRGRIILNLKVIDKPGRGGTATSGRNNT
VGRQNIPSRNRIIGKKGEAPYRPFQPPMKMLGFPMYGKPFGLQCGGLQPFPSHPGSCSSTGARDSHSSSSKYQSPPSP
SNSSCSEGKSTTNAAAAKSQGSAGAXPSTGGPKSSTPQTETQKGTKPPAPPQSSDEDPTASGSPFLPSSPSSSLEDEE
EEGAPDHSEGGRRKPSQQRAKHQLPASSSSDTPGDKSSTPTEPKRVPAEGDPDWHPEMAPSCKDVVVTDVTTNLVTVT
IKEFPSPASCPASPSTSPEEASSPPPSNTSDNPSPPKP

>Lbe_Cbx7 (Gene ID: 109992249)
MELSAIGEQVFAVESVLKKRVRKGNVEYLLKWKGWPPKYSTWEPEEHILDQRLVQAYEEKEQRDRALGHRRKGSKTKR
LLLQSTVYTMDLRSAHKTQEAPPPRLRLSLTRSLVPEDEDPSFGVYRKVPQPQLEHLKSKHGRPQLKCLDSDPAKQED
WAEEGEEEEEEEEEEEVDNVEDEDDMEVEVEEEEGEKEEATGKSKSILDGRKRTDSWSSAIEPDELTASEKPDRDDIW
RPIIGAGEVTVTDVTLNSLTVTFRESRVAKGFFRDLGLEV

>Lbe_LOC110004121
MELSSIGDQVFAVESITKKRVRKGNVEYLLKWQGWSPKYSTWEPEDNILDPRLVLAYEENQEKIRALAYRRKGLRPRR
LVLRNIFAMDLRSTHKEAEKPPPRLRLSLTRSMSTDVDQGERGGVYHRYTWGKTKHRASRRTIRPPEKKKEDEDEWSS
EEEERDCVTDDRREGSLYAPSVCSSPPMLERQDVELEVMEEEAEHLGGGAKEAWTDREEMSKMQTFECDQPEVSVCVP
EEGPEDAVTGDDDTFWGGVEEEVEAECPTLDRNDNTTSITTSMTAEEREEDVTRATPGIEAPPTAAEHAGKVIVTKVT
IDSLTVTFKEATVAEGFFKKY

>Lbe_Cbx8 (Gene ID: 109999244)
MELSAVGERVFAAESIIKRRIRKGRIEYLVKWKGWSPKYSTWEPEENILDSRLFVAFEQREREREELYGPKKRGPKPKT
FLLKAQAKVKAKSYEFRSDAVRGMHITYPTPELIVTPRAREGLRAVVPTIFPPSTVNRGESVRHRPPELSIREYQQPF
LQQTSSDGLIHAPKKRGPKPKPRFKDVSCRPLAPEPHRGRAESQLGQYKVPKYQGVEEMRVVRVAHRHPESHKHHHHP
HHHHHTHNIGASGGGSHKQFYADRGLHPHRVDMNTHGTKDSSSYLAPAHFKHRSKMSQNPSRPLELPQMEKPYFLDRP
SPTRLDAQVDEVTWKPSLGNVEKVLVTDVTTNFLTVTIKESSTSKGFFKDKR

>Lbe_LOC109985837
MELSAVGESVFAAESIIKRRIRRGRWEYLVKWKGWSQKYSTWEPEENILDARLFAAFDERERERELFGPKKRGPKPET
FLLKAKAKAKEKAYEFRGEASRGIQVAYPIPEPVITPRAREGLRAVVPTIFPPSAVNRGESVYVRLPEPETRPKPAPA
ASLLVQEPGRAPKKRGRKPKPHLEYDEDDDEEDGSGEPDVKLSRPLEEPGSRGFSKLSRRLHHPHHHHHHHQGETSDH
SLMQLTKRFQEKTTITPKSSSEQRHAGLSYSCAYRPDVRTSQQRVHRTSCFVPPPKKPNHSSSEQQRHHQVRELCPPR
EQPDVSSSHDPSTRPASSWTPSFSSLDSVTVTDVTMNFLTVTVRESSTDKGFFRGKR

>Cgo_LOC115005214
MEGVTVGQVFDAECILSKRPRKGKFEYLVKWRGWSSKHNSWEPEENILDPRLLAAFHKREQEKELLFQTKGKRPRGRP
RKILPAPAATKDSRSSSSSSSVLSSSASSSSSEDEDHTKKARSGPRVQPVPQKRPQIVLAKPELPCKKKRGRKPLHPD
VRALRQAKSRPPPPPPPPSPPPPPPLPPPPPPPRHHQVLRPPREDPRPGVKKPLQPASFTYTGLSRSSRDEAAAAAASQ
PSASSFSASKASSLSCMWTSRSMSPSSSYSRTSPAPQSKNSLSELKRSISDTGGGRGDGYKASALKHGGGSLGLHSSF
GGGQAAVQRSPLSQRRQDGSLQHKQQSLNKAAPAPTPRDRANQALNLRALNLQSVSKPVSVSGLQGNIAAASRTSLRS
GAGGIVVKGGGIKETRTSAGGQRSGGGSEQARLREEMLAETKKMTGSSSARGSGGERKHGLASQNRSLNELSTGDSDE
SSSSSESERDAAPYPNNSRPSLGDDVATESDTETDWRPARSLLEHVFVTDVTANFITVTVKESPTSVGFFNSLNH

>Cgo_LOC115012765
MEELSAVGEQVFDAECILNKRLKKGKLEFLVKWRGWSSKHNSWEPQENILDPRLLAAFNKKEQEKELLLLKKGKRPRG
RPRKILENVPEATKSSSSSSGSSTSSSSDSSSCSSSSSSSEDDDDSSHVKQANPSVRTRDLHPVPQKKAQIVVAKQEP
PKKRSRKPPSPEVKELQQKGPRKTPKTPKDADVPGAIKKPVHPASFTFMGFHRGSPRDLSGQNRSSVTPGAVVKHSIS
SAGSGRSVQPASLSLNKSSQSRNVTEGKLSISSMTSGTSLDLKAAASKSKGVAALNLNTSKHLIQGTTQHTLSSPNGQ
RKAQAPVSTLQRIPNTKAVAALPSKNASSNQGSGPQPLNLRNKPVQSKDAPGNCTAPASGLRSPTNPARKTSVSQNQE
YNPPKSPATPGRLQARKSQAGAEKVKEATDIQTPRVQSRLGKSSVQKSSTEVQIQQERSASKDGKKAEMSTGEEESGS
DSDQDSSYVGQDGSVAVQNQDWKPTRSLIEHVFVTDVTANLVTVTVKESPTSVGFFSMRNY

```
>Cgo_Cbx4 (Gene ID: 115013642)
MELPAAGEHVFAVESIEKKRSRKGRFEYLVKWRGWSPKYNTWEPEENILDPRLLDAFQDRERQEQLMGYRKRGPKPKH
LLVQVPSFARRSSILADLHGASLDEDSCQKPSPIQMLRPQGQQYQLNSKKHHPYQPLCREREAEQQTNGKKFYYQLNS
KKHHHYQPDLKGHEPMFAKPREVKAPELVNKGYNLPPVLQQKCVRDKDSGCLTKVKDIAMELKKLPADLNGHKEPEKV
KPKEDALPQSNGVSNGKLKIVKNKNKNGRIVIVMSKYMENGMQAAKIKNGDSETAGKPPQGTDSSAENHLEKMKLVRK
LGLMNGFAKKTKDKPTVTSSGFNGLCPKEKEQSLKTEPTVTEQDKHVEVRGQGQLSADQPLQLTTKPNLLSLPLDRGV
PSPLDKRGSHGGLQALKRHLSDSEEHGSSKRFLSSRSISAPNAATPPTQSISIDQNGHHSHIVLQDCGYADQEEPIDL
SVVKSRPKATQPETHTQAETVTQAETHTQGETDTQTQTDVQTETQPQTETHKSTEEEKDNSSSVCKHETRKEETFPSF
QPFLGNIVITDITTNCLTVTFKEYITV

>Cgo_LOC115012777
MELPAAGEHVFAVEGIEKKRIRKGKIEYLVKWRGWSPKYNTWEPEENILDPRLLVAFQHRERQEQLMGYRKRGPKPKH
LLLQVPSFARRSCIPAGFEESSQDAEVSIKSDPIPVQRPQPQQYQLNSKKHHQYQPSSQEVPADQPTNGKKKFIYQLN
SKKHHHYEPDPNMYDAQASRLKEVVKVQEPASKPANPGWNLPLALQQKWIRDKDTGCLSKVKELAVEMRKPAVKDAES
EHALKPNPKDATLPSAISSKMKIIKNKNKNGRIVIVMSKYMDGNKVHGAKGKHGESSSEVKPQNTKPSENNPAHTTKI
VEHPENGIPKELCNGRSLPAAEHPIKCSPKDRHFSKPSPSTAEEYNTEVARGQADLPDDLPLQLTASSPMTSWAVDTN
IPTPTSVEQIRIPSFPNDRKRKLSDPAEDRSVSKTYLASRSFSVPSTVVTPPQDKPMDLHCNGPRHSSTSTFEVVDSG
SQEEPMDLSCPKTKKQVEPEIQPEPEPAVTNTPPVMEDTQQSTEKSKEAPVKNTISPFMGNIIITDITTNSLTVTFKE
YVSF

>Cgo_cbx6 (Gene ID: 115005610*)
MSQSDCSSLTQDAAASVLALSAPLRVAKLLSSARYHGDRGEGRSEGVGMRESKQAPVAPSPKLNSLAATHKLKKDIHR
CHRMSRRPLPRSDPMASSFSNPGGFPSRLHVSPFSETVRILNRRVKPREVKRGRIILNLKVIDKAGRGGAAAGTRNIT
AGRQNIPSRNRIIGKKGEAPYRPFQPPLKMLGFPMYGKPFGLQCGGPLSFHSHPGSSTGAHSTSSQYHSTPSSSTGSE
GKSCTKASAWSQPATGASASSVAHKSSKAPAETQKSQSAKPAASAPSSDANPEASPVLPSSPSSSSSAILEDEEEDGA
LESCATPEGGKKRRAKRQPPTAAADQSSAPAEPQRALAEGDPNWHPEMAPSCKDVVVTDVTTNLVTVTIKEFPSPASC
PASPSATPDNASPPPPSTTSDDPSPAKP

>Cgo_LOC115012745
MEQSSIGDQVFAVESITKKRVRKGNVEYLLKWQGWSPKYSTWEPEDNILDPRLVLAYEEHQEKIRALAYRRKGLRPRR
LVLRNIFAMDLRSANKASEKPPPRLRLSLTRSMSTDVDQGERGSLYRRLARRKSKQRVSKRGSEGPSNNSILPLRKIE
EPIEEDWGATSEESNTEERHEDSLYECSSPPLLERQDLEMEVEEKLDIDLAAVSTERWTDGADGWTSDTRQSQMFACD
QSKDSASVSEALPEDAVSVGDRSDWDKSEEGVECPRLQSRNCQSNNTTSTIASLQGSSGTAGDYFTTEEATNEEASGN
NQSKTGTSPGSQADPAAEEHPMKVISTDVTINSLTVTFKEATVAEGFFKGY

>Cgo_LOC115024515
MELSAIGEQVFAVEAIVKKRVKKGNVEYLLKWKGWPPKYSTWEPEEHILDQRLVKAYEEKEQEEKTLGHKRKGSKAKT
LVLQDTVYTMDLRSAHKTPEQPPPRLRLSLTRSLLPEDEDQPYGACRRDPQLEHREVQPRRSKFTCLDSNPPSPTQED
WEGWGEEEDVGSEEDTEEEALKETAERSGGTLDGQRRTVERRSIAPHEITAAGKPDDVWRPIVVPGEVTVTDVTLNSL
TVTFRESRVAKGFFRDLALKV

>Cgo_Cbx8 (Gene ID: 115013830)
MELSAVGERVFAAESIIKRRIRKGRIEYLVKWKGWSPKYSTWEPEENILDFRLFAAFEEREREELYGPKKRGPKPKT
FLLKAQAKIKAKSYEFRGDAVRGMHITYPTPEPVVTPRAREGLRAVVPTIFPPSAVNRGESVRVRQPELSAREYQQPS
LQQTGPDGLIHIPKKRGPKPKPRFMDGNCSTTVFEPLKRRAEQQGSHGPHTLAKLQGGEEMRLINHRHPEIHGHKRHH
HHHHHHTHDRGSSGGSSFNQLYLERSLHPHRTDMDSHRTKDSSSYLAPVHFRHQSRMSQSLSSELPQMEKPYFLDRP
SPTRLDVDLDEVTWRPSVGNVEKVLVTDVTTNFLTVTIKESSTSKGFFKDKR

>Cgo_LOC115012434
MELSAVGESVFAAESIIKRRIRRGRWEYLVKWKGWSQKYSTWEPEENILDARLFAAFEESEREELFGPKKRGPKPET
FLLKAKAKEKTYEFRREAPRGFQVSYPTPEPVIIPRAREGLRAVVPTIFPPSAVNRGESVYIRPPEPERRPRSTPPAA
LTDQEAVRFPKKRGRKPKLRLHYDEKADGGISAEPDTKRSRSLEEPVSHGLSNMSRRLLHHGVAPDHSLMQLTRRFQE
KSTITPKHSSSSSSSSSEQRHAGLCCSAFNPDVGQLGHRTKSLVSVPRPSSSEEHRRHQVKECCEPDVISTQSESIHEQ
AARPAESWSPSFTNLDTVTVTDVTMNLLTVTVRESSSDKGFFREKRRVRASDV

>Ena_cbx2 (Gene ID: 115047492)
MEELSAVGEQVFDAECILNKRLRKGKLEFLVKWRGWSSKHNSWEPQENILDPRLLAAFNKKEQERELLLRKRGKRPRG
RPRKVLENVPEAPKSSSSSSGSSSSSSDSSSSCSSSSSSSEDDDDDEDSNVKQVNPSVRTRDLHPVPQKKAQVVVAKQ
EPSKKRIRKPLPPDVKEFQQNKSPRKILKISKDADLPGAIKKPVHPASFTFMGFHRGSGRDTAGGQSRSPLPQGGAVK
TSMSSVGSGRPVQPASLPLNKSSQGRNVAEGKLSISSMSSGTSLDLKAAGKSKGVAALNLNTSKHPIQGTTQHPVSPP
NGQKKPQGSVLTLQRIPNTKAVASVPSKTASSNQGSGLQPLNLQSKPAQSDDAAAPGSGVRNATNLARKTNVAQQNQE
YNPPKSPATPGRLQARKNQPGAEKVKEANEIQTSRIQGRLEKNSMHKSSTEVQSQQERSTYKDGKKAKMNDMSTGEEE
SSSDSDQDSSYTGQERSVAVQNQDWKPTRSLIEHVFVTDVTANLVTVTVKESPTSVGFFSIRNY

>Ena_LOC115059891
MEGVTVGQVFDAECILSKRPRKGKFEYLVKWRGWSSKHNSWEPEENILDPRLLAAFHKREQERELLFQKKGKRPRGRP
RKILPPVPTTTKDSRSSSSSSSGLLSSASSSSDDEDHSKKVKRVHPIPQKRPQIMLAKPNRPRKKKRGRKPLHPDLRA
```

LRQVKSRPPTPPTPPLPVRHHQAHRPPRDEPRSGVKKPLQPASFTYTGLSRTSRDEAGSASQTSSFSQAVATKSGSLS
CIWTSRSLSSSMPSSGSSHNKVSPAAQSKNSVSDLKRSMSESGSRDAFRVSPLKQSGGSGSLGLHGGVGGSQAAAQRS
QLGQRRQESVGGQSGSVQHKQQNFGLSKPSLSSPSPRDRVNQALSLRALNLQSVSKPSAGSGLHGGNTGSAVATASRS
SSRSGSSIVIKGGVTSIKTGGQRPGLAAGGAAEQGRGKELLTGASSGRGNGGGRQEDRKHVLISQNRSLNEFSTGDSD
ETSSSESEHGALLYPSNSRPSLGNDATESDTETDWRPTRNLMEHVFVTDVTANFITVTVKESPTSVGFFNSRNH

>Ena_LOC115047550
MELPAAGEHVFAVEGIEKKRIRKGKIEYLVKWRGWSPKYNTWEPEENILDPRLLVAFQNRERQEQLMGYRKRGPKPKH
LFFQVSSFARRSSIPAGFEETCQDTENSPKSDPIQVQRSQPQQYQLNSKKHHQYQPNGQEVLPDQLTNSKKKFIYQLN
SKKHHHYEPDPNMFDTQASRLKEVVKVQESSCKPPNPGWNSPLALQQKWVRDKDTGCLNKVKELAVEVRKPAVKEAES
EHALKPNPKDAMLPSSSSSKMKIIKNKNKNGRIVIVMSKYMDSNKVHGAKGKHGESSSEDKPQNTKPLENNPAHRTKM
VEHPENGIPKEICNGSSLPAAEHPIKCSPKDRNFSKPSPSTAEEYNTEVARGQADLPDDLPLQLTASSPPMSWAVDTN
IPTPTAVDQIRIPSFPNDRKRKLSDPMEDRNVSKTYITSRSFSVPSTVITPPQDKPMDLQCSGRRLNSTCTYEAMVAG
SQDEPMDLSCPKTKRQVEPEIQPELVPEPEPAVSDSPPVTEDTQESTEKSKEEPVEKISPFMGNIIITDITTNSLTVT
FKEYVSF

>Ena_LOC115044326
MELPAAGEHVFAVESIEKKRSRKGRVEYLVKWRGWSPKYNTWEPEENILDPRLLDAFQDRERQEQLMGYRKRGPKPKH
LLVQVPSFARRSSILADLHEASLDEDNCHKSSPIQMLRPQGQQYQLNSKKHHQYQPLCREREAEQQTNGKKFYYQLNS
KKHHHYQPDLKVHEPIFAKPRDIRVPEVPNKGHNVPPMLQQKWVRDKDSGSLTKVKDITMELKKLPADLNGHKEPEKV
KPTEDALPQSNGVSSSKLKIVKNKNKNGRIVIVMSKYMENGMQAAKIKNGDEKPPKGADNSTEDELEKMKLVKKLGLM
NGFTKIPKGKPTVPSSGFNIDCPKDREQSPKVEPTVTEQDKHVEVRGQGQLPADQPLKLTTKSNLLSLPLDRGVPSAL
DKRGAQGGFQGLKRHLSDTDCDEHRSKRFLSPGSISVPNTVSSPSQSISIDQNGHQTPSGLQVCGYAEQEEPIDLSIV
KTRPKAAAPTANQPEAHPQAESLTQKHSESSTKAETHTQAETQLDTQKEAQPQTESLKSAEGVKVDSVSVSNNEKIKE
DAFPSFQPFLGNIVITDITTNCLTVTFKEYVTV

>Ena_cbx6 (Gene ID: 115044595)
MELSAAGDRIFAAEAILKRRVRKGRIEYLVKWKGWAMKHSTWEPEENILDDRLILGFEQKEREREMNGPKKRGPKPKN
FVMKARGQKREPSSQASSTRQTTLRSTSTRAAPSSSASPHHLPSSSSSSSAVAPSPKLNSLAATHKLKKDIHRCHRMS
RRPLPRSDPMAPAFSNPGGFPSRMHVSPFSETVRILNRRVKPREVKRGRIILNLKVIDKPGRGGTAAGSRNITAGRQN
IPSRNRIIGKKGEAPYRPFQPPLKMLGFPMYGKPFGLQCGGPVRLHSQTGACSSMGTRDSKSNPPQYQSPPSPASSSG
SEGKSPTASAGQSKPATGASPSSEGPKSSKPHTETQKGQIAKLSPPAASSDANAVVSATSFLPSSPSSSLEDEEDEGA
LDRSVPSDGVRKNPRQRRVKRQPPTTLLFAAPGDQSSAPTEPKRVPAEGDPDWHPQMAPSCKDVVVTDVTTNLGTVTI
KEFPSPASCPASPSASPENGSSPPPTSTTSDPSQAKP

>Ena_cbx7 (Gene ID: 115060227)
MELSAIGEQVFAVESIVKKRVRKGNVEYLLKWKGWPPKYSTWEPEEHILDQRLVQAYEEKEQRDRAVGHRRKGSKSKR
LLLQNTVYTMDLRSAHKTPEKPPPRLRLSLTRSVVPEDEDEDEDEDEDEDEDEDEPYGDCRQGPRAQEADHKSRRSQF
RCLDSSPLTQDDWEGLGEEEEEEDEKEEEEEEDNVEDEDREEEETGLAQMETTKRNILNGRERTDGWSSAIEPDEVTA
SEKTDDVWRPVVGTGEVTVTDITLNSLTVTFRESRVAKGFFRTWGLEV

>Ena_LOC115047474
MELSSIGDQVFAVESITKKRVRKGNVEYLLKWQGWPPKYSTWEPEDNILDPRLVLAYEENQEKIRALAYRRKGLRPRR
LVLRNIFAMDLRSAHKVAEKPRLRLSLTRSMSTDVDQGEQGSMYHRSVRRKSKQRVAKRGPVGPPNKPIRPLRKKDVP
MEEDWNSVSEEEKQESESTTKERHEGSLYGQSECSSPPLLERQDLEMEAEEKVDADLTAVGSDTWTDGADGEKSETGQ
NQTSVYDQSKDSALVPVAGPGDAVTTVTAADRSGWDRDEEGLESGSECPILERINATSVIVTIQGSRETAAAACSTAE
TRNEEVRGDNQTMTMTTPGSQSTHTAAEHPGKVIVTDVTINSLTVTFKEAMVAEGFFKGY

>Ena_LOC115045432
MELSAVGERVFAAESIIKRRIRKGRIEYLVKWKGWSPKYSTWEPEENILDSRLFAAFEQREREREL YGPKKRGPKPKT
FLLKAQAKVKAKSYEFRNEAVRGMHITYPTPEPVVTPRAKKGLRAVVPTIFPPSTINRGESVRIRPPDLNPREHQQSS
PQPSNPDSFIHVPKKRGPKPKPRFKDSSYSMALEPHKRRTEDQLSHSPHKLAKLQGVEEMRLFKVAHRHPDIHGHSHK
HHHHHHRHHHHHHHHYQQNREHSSGSSYKQLYSDHSLHPHRIDSDPHRTKEGSSYLAPAHFKHQPKMTQSLSRPTELP
LMEKPYFLDRPSPTRLDVDLDEVSWRPSLGSVEKVLVTDVTSNFLTVTIKESSTSKGFFKDKR

>Ena_LOC115047560
MELSAVGESVFAAESIIKRRIRRGRWEYLVKWKGWSQKYSTWEPEENILDARLFTAFEERERERELFGPKKRGPKPET
FLMKAKAKAKEKTYEFRREAPRGIQVSYPIPEPVITPRAREGLRTVVPTIFPPSAVNRGESVHVRPPEPERRPRPSPP
AALALQESVGFPKKRGRKPKLHLLYDQEDACSSAEPDLKRSRFLEEPVSHGPTKMTRRPHHHGETSDHSLLQLTKRFQ
EETTITPKSSSEQRHFGGAGLSYTCAFSPDARKNEQMGHRTHFLSRMSGPQSGRLKPSAEHRSHQVRQCCSPREQPAA
EPFHDTSPRSASSWTPSFSNLDSVTVTDVTMNFLTVTIRESSTDKGFFRDKR

>Pra_Cbx2 (Gene ID: 11444058)
MEELSAVGEQVFDAECILNKRMRKGKLEFLVKWRGWSSKHNSWEPQDNILDPRLLAAFNKKEQEKELLLRKRGKRPRG
RPRKILENLPEAAKSRSSSSASSSSSSDSSSSCSSSSSSSDDDDDDDSQDQQANPSVRTRELHPVPQKKAQIVVAKQE
PPKKRSRKPLPLEVKEFQQNKGPRKILKTTKDIDLPGAIKKPVHPANFTFMGFHRGSARDAAGGQNRSSLTPGGAVKN
SVGAVGSGRSSVQPPSFPLNKSSQSRNVTEGKLSISNMNSGTSLDLKAAAGKSKGVAALNLNTSKHPIQGTTQHTLSS
PNGQKKLQTPVSTVQRIPNTKTVSLLSKNAPSNQSSGLQPLNLQNKPVQSNDPPGNGTTPASGQRNPATPARKTTVPQ

NQECNTPATPVKLQGRKTQTGADKVREVNKIQTSRVQGRLEKSSMHKSPTEVQSQQERSASKDGKKAKMTDMSTGEEE
SSSDSDQDSYAGQDRSVAIQNQDWKPTRSLIEHVFVTDVTANLVTVTVKESPTSVGFFSIRNY

>Pra_LOC114452074
MEGVTVGQVFDAECILSKRPRKGKFEYLVKWRGWSSKHNSWEPEENILDPRLLAAFHKREQERELLFQKKGKRPRGRP
RKILPPAPAATKDIRSSSSSSSGLSSSASSSSSEDEDHTKKAKPSPRVHPVPQKRPQIMLAKPDPPRKKKRGRKPLPP
DLKALRQAKSRSPPPLPPPRQHQALRPPRDELRPGVKKPLQPASFTYTGLSRTSRDEAASSSQTSSSSFAQSASSKPG
TLSSIWTSRSLSTSSPSSGSSHNKANPSAQSKNSVSELKRSISETGSSRGDFKVSPLKQGGASGTLGLHSSFGSGQLA
VQRSTLSQRRQESIGGQGGLVQHKQQNSSLSKPSSSSSPTPRDRVNQALSLRALNLQSVSRPPTSSSLQGNNTSSAAA
RLSLRSGSNMVIKGSTKETRISAGGQRSSLAAGSAVEQGRGKEKQEDRKHTLSSQNRNLNELSTGDSDETSSSESEPD
LSLYPSNSRPSLGINATDSDTETDWRPARSLLEHVFVTDVTANFITVTVKESPTSVGFFNSRNH

>Pra_Cbx4 (Gene ID: 114436730)
MELPAAGEHVFAVESIEKKRSRKGRVEYLVKWRGWSPRYNTWEPEENILDPRLLDAFQDRERQEQLMGYRKRGPKPKH
LLVQVPSFARRSSILADLQEESLDDDVGCQKAQQYQLNSRKHHQYQPLCREAEPQANGKTFYYQLNSKKHHHYQPDLK
VHEPIFSKPREVKAPELANKGYNLPPVLQQKWVRDKDSGCLTKVKDITMELKKLPADLNGHKAPEKVKPKEDTSPQSN
GVTSSKLKIVKNKNKNGRIVIVMSKYMENGMQTAKIKNGSSERAEASPQGAESAAENHLEKMKLVKKLGLMNGFSKSL
KDKATVPTSGFNRDCSKEKEQSPRAEPTVTEQDKHVGVRGQGQLPADQPLQLTTKPNLLPLPLDSSQDKGGSGLKRHL
SDTDAVEHGGGKRFLSCRSISAPDAASSPATNVQQSRSAELQDCVYADQEEPIDLSMVKSRPTASLTTHTQPETVTHT
QDETQTHNPEPAEEDAAEPVCNQETRKEETSPSFQPFLGNIVITDITTNCLTVTFKEYVTA

>Pra_LOC114439615
MELPAAGEHVFAVEEIEKKRIRKGKTEYLVKWRGWSSKYNTWEPEENILDPRLLVAFQHRERQEQLMGYRKRGPKPKH
LLLQVPSFARRSSIPAGFEEISPDAEGSHKSDPLQVQRSQPQQYQLNSKKHHQYQPSSQGVPADQIANGKKKFIYQLN
SKKHHHYEPDPNMYDAQASRLKEVVKVQEPASKPANPGWNLPLALQQKWVRDKDTGCLSKVKELAVEVRKPAVKEAES
EHALKPNPKDATLPSSISSKMKIIKNKNKNGRIVIVMSKYMDSNKVHGAKGKHGESSREEKSQKPSENNPAHRTKMAE
HPENGIPKEICNGSSLSAAEHPIKCSPKDRQFSKPSPSTAEEYNTEVARGQADLPDDLPLQLTASSPVASWAVDTNTP
TPTAVDQIRIPSFPGDRKRKLSDPVEDRSVSKVYLTSRSFSVPSTVVTPPQDKPMDLHCSSSRRSSTCTYEDMGSQDE
PMDLSCPKNKTQIQTETPPEPEPVQDTPPVTEDTQKSDTSKEAVKKVSPFMGNIIITDITTNSLTVTFKEYVSF

>Pra_Cbx6 (Gene ID: 114439322)
MEPSAAGDRIFAAEAILKRRVRKGRLEYLVKWKGWAMKHCTWEPEENILDDRLILGFEKKEREREMHGPKKRGPKPKN
FVTKTRGPKGESSSRASNSRQSASARSSSSTSTRAAPSSSPSPNLPSPSSSSSAVAPSPKLNSLAATHKLKKDIHRCH
RMSRRPLPRMDPMGPAFSNPSGFPSRMHVSPFSETVRILNRRVKPREVKRGRIILNLKVIDKPGRGGTAAGGSRSITS
GRQNIPSRNRIIGKKGEAPYRPFQPPLKMLGFPMYGKPFGLQCGGAVSFHSHPGSCSSTGARDRRTTTPKYQSPPSPS
SSSGSEGKSPTNATAVTGATPSNKNPKSSKPHTETQQGQSTKPSAPALSSDANLAASASPFLPSSPSSSLDDEEEEGA
PVPAEGERKSLRQRRAKCQLPSDSTEDQTSTPAEPQRVPAEGDPDWHPEMAPSCKDVVVTDVTTNLVTVTIKEFPSPA
SCPASPSACPENTSSPPPSTATDDPAPEKP

>Pra_LOC114437794
MELSAIGEQVFAVESILKKRVRKGNVEYLLKWKGWPPKYSTWEPEEHILDQRLVQAYEEKEQRDRAVGYRRKGSKAKR
LILENTVYTMDLRSAHKAPEKPPPRLRLSLMRSVAPEDEDQPYDACRPHHQISKQRRSRFRCLNSNPPSSTDEDWEGG
GEEEEEEEEEEDGEEEEEVTAKKTEGRLNGQESTDGWSATVGPDEVAASEKPSDVWRPVIVPGEVTVTDVTLNSLTVT
FRESRVAKGFFRDWGLAV

>Pra_LOC114439674
MELSSIGDQVFAVESITKKRVRKGNVEYLLKWQGWPPEYSTWEPEENILDPRLVLDYEENQEKTRALAYRRKGLRPRR
LVLRNIFAMDLRSAHKVTEKPPPRLRLSLTRSMSTDVDQVYRRPLRRKIRQKVTKRVPEGVPNKPIHLQKKKEEEEEA
LVEDWGGISEEDKPVSDGIIEERCEGSLYGRSECSSPPLLERQDLDMEVEEKVETAVSTEMWTDGEDGEKTCETTQTF
DQSKDSNPAPEPAPGDVVAVGDRSDIDRRDDEAGSEVPRAERSNATSVIVSVLAASQERKEEDEDNQSVTTTTTTTTT
TLGSQTAEHPGKVFVTDVTVNSLTVTFKEAVMAEGFFNGY

>Pra_Cbx8 (Gene ID: 114436737)
MELSAVGERVFAAESIIKRRIRKGRIEYLVKWKGWSPKYSTWEPEENILDSRLFAAFEQREREREELYGPKKRGPKPKT
FLLKAQAKVKAKSYEFRSESVRGMHITYPTPEPVVTPRAREGLRAVVPTIFPPSTVNRGESVRVRPPELSTREHQQSP
LQHTGPDGLIHIPKKRGPKPKPRFKDGSFGPAASEPHKRRPEEHLSHSSHKLAKLDHRHHLHHHHHHHHHHAHSRGV
SAGSSYKQLYSDRSLHRTDMDTHKTEHASAYLAPPHFKHHHSKTSPGLSRPAELPQMEKPYFLDRPSPTRLDDDLDEV
TWRPSLGNAEKVLVTDVTSNFLTVTIKESSTSKGFFKDKR

>Pra_LOC114439616
MCGIKQPEEDEGSQKIIKETEINEVHGESWSSRLLGRWEYLVKWKGWSQKYSTWEPEENILDARLFAAFEEREREREK
FGPKKRGPKPETFLLKAKAKEKTYEFRREASRGIQVSYPVPEHVITPRAREGLRAVVPTIFPPSTVNRGESVHIRPPE
PERRPRPTPPSALNAPESLRFPKKRGRKPKLLLRYDKEDGSSSAEPPDTKRSRLHEEPVSHCLSKMSRRLHHYHHHHH
HHHHHHGETSEHSLLQLTKRFQEETTITPRSSSEQRPTGGAGLSYTCAFSSNRSDPGGHRTKCLKVSSIPQLKHSAG
QQGREVKESAAMSSQSSSWTPCLTMDSVTVTDVTINFLTVTIKESSTDKGFFRDKR

>Lca_LOC108874733

MEGVTVGQVFDAECILSKRPRKGKFEYLVKWRGWSSKHNSWEPEENILDPRLLAAFHKREQERELLFQKKGKRPRGRP
RKILPAPAATKDSRSSSSSSSGLSSSASSSSSEDEDHTKKAKPGPRVHPVPQKRPQIMLAKPDPPRKKKRGRKPLHPD
LRALRQARSRPSPPPPPPPPPPRHHQALRPTRDEPRPGVKKPLQPASFTYTGLSRASRDEPVSISQTSTSSFSQSASK
PGSLSCIWTNRSLSTSSPSSSSHNKTSPSPQSKNSASELRRSMSESSNRGDGLKVSPLKQSGGSGSLGLHGSFGGGQG
AAQRSQLGQRRQEGVGSQIGMVQHKQQNSGLSKPLSSQTPRDRANQALSLRALNLQSVSRPPSSSSLQGNNTSSAGAT
PSRSSLRSGGGGIVKGGAGSIKETRMSAGGQRSGLAAGGATDQGRGKEMGGSSGRGTGRQEERKHGLNSQNRSLNELS
TGDSEETSSSESEHDASLFPNNSRPSLGDDATESDTETDWRPTRSLLEQVFVTDVTANFITVTVKESPTSVGFFNSRN
H

>Lca_LOC108874388
MEELSAVGEQVFDAECILNKRLRKGKLEFLVKWRGWSSKHNSWEPQENILDPRLLAAFNKKEQEKELLMRKRGKRPRG
RPRKILENVPEAPKSSSSSSGSSSSSSDSSSSCSSSSSSSEDDDEDGSTKQASPSVRTRDTHPVPQKKAQIVVAKQEP
PKKRSRKPLPPEVKEFQQNKGSRKILKTPQDTDLPGAIKKPVHPASFTFMGFHRGSVRDTVGSQNRSSLTQGGAVKNS
MSSVGSGRSVQPASLPLNKSVQSRNVTEGKLSISSMSSGASLDLKAAAGKSKGVAALNLNTSKHPIQGTTQHTLSSPN
GQKKPQGPVSTLQRIPNTKAMASVPLKNPSSSQGSGLQPLNLQSKPVQNNDAPGNGTTPAAGLRNATNPARKTTVTQN
QEYNPPKSPATPGRPPARKNQPGADKVKEATEIQASRVQGRLDKSSVHKSTTEVQSQQERPAYKDGKKADMSTGEEES
SSDSDQDSSYTGQDRSMGVQNQDWKPTRSLIEHVFVTDVTANLVTVTVKESPTSVGFFSIRNY

>Lca_Cbx4 (Gene ID: 108884566)
MELPAAGEHVFAVESIEKKRSRKGRVEYLVKWRGWSPKYNTWEPEENILDPRLLDAFQDRERQEQLMGYRKRGPKPKH
LLVQVPSFARRSSILADLHEASLDEDSCQKSSPIQMLRPQGQQYQLNSKKHHQYQPLCREREAEQQTNGKKFYYQLNS
KKHHHYQPDLKAHEPIFAKPRDVRSPELPNKGYNVPPVLQQKWVRDKDSGCLTKVKDITMELKKLPADLNGHKEPEKV
KPKEDALPQSNGVSSSKLKIVKNKNKNGRIVIVMSKYMENGMQAAKIKNGDSESAEKPPQGTDSSMENSVEKMKLVKK
LGLMNGFAKNPKDKVIVPSSGFNIGRPKEKEQSPQTEPTVMEQDKHVGVRGQGQLPADQPLQLTTKPNLLSLPLDRGV
PSSPDKRGNQGGFQGLKRHLSDTDCDEHESSKRFLSSRSISVPNTASSLSQSIGTDQNGHGSHAGPQVCGYADQEEPI
DLSIVKSRPKAAASTATQPEIYTQAETVTHTQGETQTQMGAQTETQPQTETQKTAEKVKVDSLFVPNNEKRKEETFPS
FQPFLGNIVITDITTNCLTVTFKEYITV

>Lca_LOC108879647
MELPAAGEHVFAVEGIEKKRIRKGKIEYLVKWRGWSPKYNTWEPEENILDPRLLVAFQHRERQEQLMGYRKRGPKPKH
LLLQVPSFARRSSIPAGFEETCQDVESGLKSDPVQVQRSQPQQYQLNSKKHHQYQPSNQEVPADQLTNGKKKFIYQLN
SKKHHHYEPDPNMYDAQASRLKEVVKVQEPACKLANPGWNLPLALQQKWVRDKDTGCLSKVKELAVEVRKPAVKEAES
EHALKPNPKDATLPSAISSKMKIIKNKNKNGRIVIVMSKYMDSNKVHGAKGKHGESSGEEKLQNTKQSESNPVHRTKM
VEHPENGIPKEICNGSSLPAAEHPIKCSPKDRHFSKPSPSTAEEYNTEVARGQADLPDDLPLQLTASPPPMSWAVDTN
IPTPTAVDQIRIPSFPNDRKRKLSDPVEDRSVSKTYLTSRSFSVPSTVITPPQDKPMDLHCSGRRLSSTCTYEVLDSG
SQEEPMDLSCPKTKRQVEPEIQPEPAPEPEPAVRDTPPVTEDTQKSTEKCKEAPVKKISPFMGNIIITDITTNSLTVT
FKEYVSF

>Lca_Cbx6 (Gene ID: 108887174)
MELSAVGDRVFAAEAILKRRVRKGRIEYLVKWKGWAMKHSTWEPEENILDHRLILGFEQKERERELHGPKKRGPKPKN
FVMKARAQKSRTSSQASSTRRSTSRSSVSTSSRAAPSSSASPHHLPSSSSSSSTVAPSPKLNSLAATHKLKKDIHRCH
RMSRRPLPRADPMAPTFSSPGGFPSRMHVSPFSETVRILNRRVKPREVKRGRIILNLKVIDKPGRGGTAAGSRNVTTG
RQNIPSRNRIIGKKGEAPYRPFQPPQKMLGFPMYGKPFGLQFGGPVPFHSHPGSCSSTGARDSRTNCSQYQSPPSPSS
SSGSEGKSPTASAAQVQPTTGTSPSSEGPKPSKLHTETQKGQSAKHSAPAPSSDANPVVPASSFLPSSPSSSLDDEEE
ESALDRSAPSEGGRKNPRQRRSKRQLPTSVPSVAQGDQSSAPIEPQRVPAEGDPDWHPQMAPSCKDVVVTDVTTNLVT
VTIKEFPSPASCPASPSGSPENASSPAATSDDLSPAKP

>Lca_LOC108877652
MELSSIGDQVFAVESITKKRVRKGNVEYLLKWQGWPPKYSTWEPEDNILDPRLVLAYEENQEKIRALAYRRKGLRPRR
LVLRNIFAMDLRSAHKVAEKPPPRLRLSLTRSMSTDVDQGERGGMYRRPLRRRSKQRMAKRGPDGPSNKPIRPLRKKE
EPMEEDWCDTSEEEKQESESTTEERREDSLYGQSECSSPPLLERQDLEMEVEEKLDADLTAVSSDTWTDGPVRVTSET
GQNQTFVCIQSKDSASVPVAGQGDAVTVGNRSDWDRGEEGSVEPGSECSRLERNNTTSVIVSIQGSSETAGAAAVCSA
PETRDEEVRGDNQSVTTTTPGSQSAPAAAEHPGKVIVTDVTINSLTVTFKEAMVAEGFFKGY

>Lca_LOC108889108
MELSAIGEQVFAVESIVKKRVRKGNVEYLLKWKGWPPKYSTWEPEEHILDQRLVQAYEEKEQRDRAVGHRRKGSKSKR
LLLQNTVYTMDLRSAHKTPEKPPPRLRLSLTRSLVPEDEDEPYDACEQDPRQQGAHHKSRPRRSQFRCLDSNPPSPTQ
EDWEGLGEEEEEEEEEEEEEDEDNVEETEKETTEKSGGILNGKCIGWSPAADPDEVTASEKPDDVWRPVIAPGEVTV
TDVTLNSLTVTFRESRVAKGFFRDWGLDV

>Lca_Cbx8 (Gene ID: 108884564)
MELSAVGERVFAAESIIKRRIRKGRIEYLVKWKGWSPKYSTWEPEENILDSRLFAAFEQRERERELYGPKKRGPKPKT
FLLKAQAKVKAKSYEFRSEAIRGMHITYPTPEPVVTPRAREGLRAVVPTIFPPSTVNRGESVRVRSPELSTREHQQAS
LQQTSPDGLIHAPKKRGPKPKPRFKDSSCSPMASEPHKRRAEEQVSHGPHKLAKLQAVEEMRLFKVARRHPENHGHSH
KHHHHHHHHHHHQHNRELSSGSSYKQLYSDRGLHPHRTDMDTHRTKEGSSYLAPAHFKHQSKISQSLSRPTELPLME
KPYFLDRPSPTRLDDDLDEVSWRPSLGSVEKVLVTDVTTNFLTVTIKESSTSKGFFKDKR

>Lca_LOC108879657

MELSAVGESVFAAESIIKRRIRRGRWEYLVKWKGWSQKYSTWEPEENILDERLFAAFEERERERELFGPKKRGPKPET
FLLKAKAKAKEKTYEFRREAPRGIQVSYPIPEPVITPRAREGLRAVVPTIFPPSAVNRGESVHVPPPEPDRRPRPPAA
LTLQESARFPKKRGRKPKLHLHYDKNDGGGAAEPDSKRSRLVVEEEEEEEEEEDDEEEEEETVSHHGLSKTTRRLHHH
GETSDHSLIQLTKRFQEETTITPKSSSEQRRAGGLSYTCAFRPDGRKSDQRGHRTNCLSVPQPGRPKHPTEHRSHHQV
REYCLPGRGQRPSRTSPQPESIHDSSARPASSWTPSFTNLDSVTVTDVTMNFLTVTIRESSTDKGFFRDKR

>Spa_Cbx2 (Gene ID: 103356360)
MEELSAVGEQVFDAECILNKRLRKGKLEFLVKWRGWSSKHNSWEPQENILDPRLLAAFNKKEQEKELLMRKRGKRPRG
RPRKILDNVPEAAKSSSSSSGSSSSSSDSSSSCSSSSSSSEDEDDDSHVKQANPSVRARELHPVPQKKAQIVMAKQEP
PKKRSRKPLPPEMKEFPQNKGSRKILKTTKDADLPGAIKKPVHPASFTFMGFHRGSARDAVSGQNRSSLAQGGAVKNS
MSPVGSGRSSVQPASASMNKSSQSRNVTDGKLSISNMSSGTSLDLKAAASKSKGVAALNLNTSKHPVQGTTQHTLSSP
NGQKKPQAPVPTVQRIPNAKPSLPSKNASSNQGSGLQLLNLQNKVAQTNDAPGNGTTPTSGPRNATNPARKTTVTQSQ
ECNPPKSPATPGRPPARKTQPGADKVREVNEIQSSRVQGRLEKSSVHKSSAEVQSQHERSASKDGKKAKMNDMSTGEE
ESSSDSDQDSSYAGQGRSVTVQNQDWKPTRSLIEHVFVTDVTANLVTVTVKESPTSVGFFSIRNY

>Spa_LOC103370168
MEGVTVGQVFDAECILSKRPRKGKFEYLVKWRGWSSKHNSWEPEENILDPRLLAAFHKREQERELLFQKKGKRPRGRP
RKILPAPAATKDSRSSSSSSSGLSSSASSSSSEDEDHTKKPKPGPRLHPVPQKRPQIMLAKPDPPRKKKRGRKPLHPD
LRALRQAKPRPPAPPPPPAPRHHQVLRRDEPRPGVKKPLQPASFTYTGLSRSSRDDGSQTSSSFSQTASKPGSLSCIW
TNRSLSSSSPSSGSAHKSSPSPQSKNSLSELKRSVSETSRDGFKVSSLKQGGSLGSHGFGPTAAHRSPLAQRRQDGGP
TGLVPHKQQNSSLSKPSSSSPSPRDRASQALSLRALNLQSVKPPAGSLQGNTSGAVAAVARSSLRSGSIVVKGGVGKE
MRTPGGQRSSLTAGGAAEQNRAKEKLAGGSSGRGRPEKHGLSSQNRSLNELSTGDSDDSSSSESEQDAAMFPGNSRPS
LGNNATESDTETDWRPTRSLLEHVFVTDVTANFITVTVKESPTSVGFFNSRNH

>Spa_Cbx4 (Gene ID: 103372324)
MELPAAGEHVFAVESIEKKRSRKGRVEYLVKWRGWSPRYNTWEPEENILDPRLLDAFQDRERQEQLMGYRKRGPKPKH
LLVQVPSFARRSSILADLHEASLDDDGCQKSSPIQMLRPQAQQYQLNSKKHHQYQPLCREAEQQPNGKKFYYQLNSKK
HHHYQPDLKAHEPLFAKPREVKAPELANKGYNLPPVLQQKWVRDKDSGCLTKVKDITMELKKLPADLNGHKEPEKAKP
KEEALQQSNGVSSSKLKIVKNKNKNGRIVIVMSKYMENGMQAAKIKNGESAEKPPLGTDGSAENQLEKMKLVKKLGLM
NGFAKNPKDKPTGFNGDCPKEKEQSPKTEPTVTEQDKHVEVRGQGQLPADQPLQLTTKPNLLPLTSDRGVSSPPDKRG
SQGGFQGLKRHLSDTDGEEYVGGKRFLSCSSVSAPNAASLPAQSISVDQNGHQSHSGLQDCGYADQEEPIDLSIVKSR
PPAVQPETQTQAEAPTRGETQTQTDVQTEPQPQTEAQQPAEEEKVDSVSVSNHEKRKEETFPSFQPFLGNIVITDITT
NCLTVTFKEYVTA

>Spa_LOC103361728
MELPAAGEHVFAVEGIEKKRIRKGKIEYLVKWRGWSPKYNTWEPEENILDPRLLVAFQHRERQEQLMGYRKRGPKPKH
LLLQVPSFARRSSIPAGFDETSPDAESSPKSDPIQVQRSQPQQYQLNSKKHHQYQPSSQEVPADQLANSKKKFIYQLN
SKKHHHYEPDPNMYDAQASRLKEVVKVQEAVSKPANPGWNLPLALQQKWVRDKDTGCLSKVKELAVEVRKPAVKEAES
EHALKPNPKDATLPSAISSKMKIIKNKNKNGRIVIVMSKYMDSNKVHGAKGKHGESSREDKPQNTKPLENNPAHRTKM
AEHPENGIPKEICSGSSLAAAEHPTKCSPKDRQFSKPSPSTAEEYNTEVARGQADLPDDLPLQLTASSPPTSWTIDTN
ILTPTAVDQIRIPSFPGDRKRKLSDPVEDRSVAKIYLASRSFSVPSTAVTPPQDKPMDLHCSGSRHSSICTYEALGSQ
EEPMDLSCPKSKKQVEAETQLEPEPEPERELEREPEPEPEPEPEPEPEPEPEPELAVKDTPPVTEDTQTSTEKPAAVK
KVSPFMGNIIITDITTNSLTVTFKEYVSF

>Spa_Cbx6 (Gene ID: 103368006)
MEPSAAGDRIFAAEAILKRRVRKGKLEYLVKWKGWAMKHSTWEPEENILDDRLILGFEQKERDRELHGPKKRGPKPKN
LVMKSRGPKRETSSRASNSRRNTSRSSSSASSRAAPASSSSSSAPPHHLPSSSSSSSTVAPSPKLNSLAATHKLKKDI
HRCHRMSRRPLPRSDPMAATFSNPGGFPSRMHVSPFSETVRILNRRVKPREVKRGRIILNLKVIDKPGRGGTAAGSRN
IAAGRQNIPSRNRIIGKKGEAPYRPFQPPLKMLGFPMYGKPFGLQCGGPVPFHAHPGSCSSTGARERRTTSSQYQSPP
SPSSSSGSEGKSPTNATATKPQPATGASPSSKDAKSSKPHTETQKGQSSKPSAPAPSSDANPAAASASPCLPSSPSSS
LEDEEEEGAPDHSAPSEGGRKNPRQRRAKRQPPTTLPSVTPGDQSSAPAEPQRVPAEGDPDWHPEMAPSCKDVVVTDV
TTNLVTVTIKEFPSPASGPASPSASPENASSPPPATASEDPSPAKP

>Spa_Cbx7 (Gene ID: 103376032)
MELSAIGEQVFAVESIVKKRVRKGNVEYLLKWKGWPPKYSTWEPEEHILDQRLVQAFEEKEQKDRTLGHRKKGSKSKR
LLLENTVYTMDLRSAHKTPEKPPPRLRLSLTRSLVPEDEDQPYDDCRQGPGPHLAHRKSKQRRSQFRCLNSNPPSPTQ
EDWEGWEEEEEEEEVENNVEDDEDREEKEEAQKETATKRDSILHGQERTGDWSSATRPVEVTAAEKPDDVWRPVIRPG
EVTVTDVTLNSLTVTFKESRVAKGFFRDLGLEV

>Spa_LOC103373459
MELSSIGDQVFAVESITKKRVRKGNVEYLLKWQGWPPKYSTWEPEDNILDPRLVLAYEENQEKIRALAYRRKGLRPRR
LVLRNIFAMDLRSAHKVTEKPPARLRLSLTRSMSTDVDQVERGGMYRRPPRRRSRQRLNRSGTEGPPNKPIRPQRKKE
EPMEEDWGGTSEEDKQESESTTEERCEDSLYGQSECSSPPLLERQDLEMEVEEKVEADLTVVSTDMWTDRSGGGTSET
RQKQTFASDESDDSALVPEAKLGDVAPVGDGSEREESVASGWESVGVESSSGQRSNTTSVIVSVQGSSETAADAAAVC
CAAEERKEEVRGDNQTITTKAADSQTGPAEAEHPGKVIVTDVTINSLTVTFKEALVAEGFFKNY

>Spa_Cbx8 (Gene ID: 103372326)

MELSAVGERVFAAESIIKRRIRKGRIEYLVKWKGWSPKYSTWEPEENILDSRLFAAFEQRERERELYGPKKRGPKPKT
FLLKAQAKVKAKSYEFRSEAVRGMHITYPTPEPVVTPRAREGLRAVVPTIFPPSTVNRGESVRIRQPELSSREHQQPS
LQQTTSDGLIHMPKKRGPKPKPRFKDGSLGPAISEPHKRRVEEQMGHGPHKLSKLQGGEDMRLVKLAHRHPENHGHSH
KHHHHHHHHHHHHHTHSRGLSGGSAYKQLYSHPHRTDMDSHRTKDSSGYLAPAHFKHHSSKMSLSRPVELPQMEKPYF
LDRPSPTRLDDDLDEVTWRPSIGNVEKVLVTDVTTNFLTVTIKESSTSKGFFKDKR

>Spa_LOC103361729
MELSAVGESVFAAESIIKRRIRRGRWEYLVKWKGWSQKYSTWEPEENILDARLFAAFEERERGREMFGPKKRGPKPET
FLLKAKEKAKEKTYEFRREAPRGIQVCYPIPEPVITPRAREGLRTVVPTIFPPSAINRGESVHVGPPEPERRPRMTPP
APLTAQEPLRFPKKRGRKPKLHLHYDKDDGSSSAEPEAKRSRLLEEPLAHGLSKMSQRLHHHHHHHHHHHGETSDHSL
VQLTKRFQEETTITPKPSSGQRSTGGAGLSYSCAFSPDVRKSDQRAHRTNCLSRMYIPQPGKLKHRGGHRSHQVTECC
PAREQPAVISSSQSIHDSSARPASSWSPCFTNLDTVTVTDVTMNFLTVTIRESSTDEGFFRDKR

>Apo_LOC110965974
MEGVTVGQVFDAECILSKRPRKGKFEYLVKWRGWSSKHNSWEPEENILDPRLLAAFHKREQERELLFQKKGKRPRGRP
RKILQPAPAATKDSRSSSSTSSGLSSSASSSSSEDEDPTKKPKPGPRLHPVPQKRPQIMLAKPDPPRKKKRGRKPLHP
DLRALRQAKLRPPVPPLPPPASPRHHQAVRRDDPRPGVKKPLQPASFTYTGLGRSSRDDGSQTSSSFSQTSASKPGSL
GCIWTNRSLSSSSSGSAHKSGPSPQSKNSLSELKRSISETSRDGFKVSLKQGGGSLGLHGGFGPTAHRSPLSHRRQDG
GPTGLVPHKQQNSSLSKPSSSASPTPRDRANQALSLRALNLQSVKPPTTSLQGNTSGAVAAMARSSLRSGGIMVKGGV
GKEVRTSGGQRSGLTAGAATEQNRVKEKLAGGGSGRGSGRQDKHGLSSQNRNLNELSTGDSDDSSSSESEQDAAIFPG
NSRPSLGNNATESDTETDWRPARSLLEHVFVTDVTANFITVTVKESPTSVGFFNSRNH

>Apo_LOC110948480
MEELSAVGEQVFDAECILNKRLRKGKLEFLVKWRGWSSKHNSWEPQENILDPRLLAAFNKKEHEKELLMLKRGKRPRG
RPRKILENVPEAPKSSSSSSGSSSSSSDSSSSCSSSSSSSSDDEDDSSHVKQVNPSVRTDLHPVPQKKAQIVMAKQEP
PKKRSRKPLSPEMKEFPQNKGPRKVLKTTKDEDLPGAIKKPVHPASFTFMGFHRGSAREAVGGQNRSPLAQGGAVKNS
TSSVGSGRSSVQPASLSMNKSNQSKNVTEGKLSISSISSGTSLDLKAAASKSKGVAALNLNTSKHPIQGTTQHTLSSP
NGQKKPQAPGSTVQRIPNTKPSLQSKNASSNQGPGLQPLNLQNKGAQSNDAPGNGTTPTTGPRNATNPARKTTVTQSQ
ECNPPKSPVTPGRPPARKTQPGADKVREVNEIQTSRVQGRLEKSSVHKSSTEVQGQHERSASKDGKKAKMNDMSTGEE
ESSSDSDQDSSYAGQGHSVGVQNQDWKPTRSLIEHVFVTDVTANLVTVTVKESPTSVGFFSIRNY

>Apo_Cbx4 (Gene ID: 110963665)
MELPAAGEHVFAVESIEKKRSRKGRVEYLVKWRGWSPRYNTWEPEENILDPRLLDAFQDRERQEQLMGYRKRGPKPKH
LLVQVPSFARRSSILADLHGSSLDDDGCQKSSPIQMLRPQAQQYQLNSNKHHQYQPLCREAEQQPNGKKFYYQLNGKK
HHHYQPDLKVHEPLFVKPREVKAPELANKGYNLPPVLQQKWVRDKDSGCLTKVKDITMELKKLPADLNGHKEPEKAKP
KEDTLPQSNGVSSSKLKIVKNKNKNGRIVIVMSKYMENGMQAAKIKNGDSESGEKSPQGTDSNSENQLEKMKLVKKLG
LMNGFAKHPKDKPTGFNGDCPKAKEQSPKTKPTVTEQDKHVEVRGQGQLPADQPLQLTTKPNLLPLTSDQGVSSLSDK
RVGQGGFQGLKRHLSDMDGEEHRGTKRFLSCRSISTPNAASSRGQSISVDQNGHQSHDGLQDCGYTDQEEPIDLSIVK
SRPTATQPETQTQAEALTQDLTQTDVQTENQPQTEALKPAEEKKVDSVSVSNHEKRKAETFPSFQPFLGNIVITDITT
NCLTVTFKEYVTA

>Apo_LOC110961503
MELPAAGEHVFAVEGIEKKRIRKGKIEYLVKWRGWSPKYNTWEPEENILDPRLLVAFQHRERQEQLMGYRKRGPKPKH
LLLQVPSFARRSSIPAGFEETSPDAETSPKSDPIQVQRSQPQQYQLNSKKHHQYQPSSQEVPADQLTSSKKKFIYQLN
SKKHHHYEPDPNMYDAQASRLKEVVKVQEPASKPANPGWNLPLALQQKWVRDKDTGCLSKVKELAVEVRKPAVKEAES
EQALKPNPKDATLPSSISSKMKIIKNKNKNGRIVIVMSKYMDGNKVHGAKGKHGESSREEKNQNTKPTENNPAHRTKM
TEHPENGIPKEICSGSSLAAAEHPIKCSPKDRQFSKPSPSTAEEYNTEVARGQADLPDDLPLQLTASSPPTSWTVDTN
IPTPTAVDQIRIPSFPGDRKRKLSDPVEDRSVAKIYLTSRSFSVPSTVVTPPQDKPMDLHCSGSRQSSLCSYEALGSQ
EEPMDLSCPKPKRQVEAEVQPEPEPEPEPEPAPEPASEPEPAVKDSPPVTDDTQTSTKEKSKEAPVKKVSPFMGNIII
TDITTNSLTVTFKEYVSF

>Apo_cbx6a (ENSAPOG00000013333)
MEPSAAGDRIFAAEAILKRRVRKGRIEYLVKWKGWAMKHSTWEPEENILDDRLILGFEQKERERELHGPKKRGPKPKN
FVMKARGQKRETSSRASSSRQNTPRSSSSTSSRAASSSSSALPHHLPSSSSSSSTVAPSPKLNSLAATHKLKKDIHRC
HRMSRRPLPRSDPMAATFSNPGGFPSRMHVSPFSETVRILNRRVKPREVKRGRIILNLKVIDKPGRGGTASGSRNITA
GRQNIPSRNRIIGKKGEAPYRPFQPPLKMLGFPMYGKPFGLQCGGPVPFHSHPGSCSTTGARDRRTTSSQYQSPPSPS
SSSGSEGKSPTNATAAQPQPAAGASPSSKDAKSSKPHTETQKGQSAKPSAPAPSSDGNPAASASPFLPSSPSSSLEDE
EEEGAQDHSVSSEGGRKNPRQRRAKRQPPTTLPTVAPGDQSSAPAEPQRVPAEGDPDWHPEMAPSCKDVVVTDVTTNL
VTVTIKEFPSPASGPASPSASPENASSPPPATTAEDPSPAKP

>Apo_LOC110954475
MELSAIGEQVFAVESIVKKRVRKGNVEYLLKWKGWPPKYSTWEPEEHILDQRLVQAFEEKEQRDRALGHRRKGSKAKR
LLLENTVYTMDLRSAHKTPEKSPPRLRLSLTRSLVPEDEDQPYSVHRRDPQPHLAHQKSSRSQFKCLNSNSPSPTQED
WEGWGEEEEEEEEENNVEDDEDREEEEAQEETAKTRDSILNGQERTDDWSSTVRPVQVTAAEKSDDVWKPVIGPGEVT
VTDVTLNSLTVTFRESRVAKGFFRDWGLEV

>Apo_LOC110969764

MELSSIGDQVFAVESITKKRVRKGNVEYLLKWQGWPPKYSTWEPEDNILDPRLVLAYEENQEKVRALAYRRKGLRPRR
LVLRNIFAMDLRSAHKVSEKSSSRLRLSLTRSMSTDVDQVERGGMYRRPARRRSRQRLNRSLTEDPANKPMCPQRKKE
EEEAMEEDWGGTSEEDKQESECTTEERCEDSLYGQSECSSPPLLERQDLEMEVEEKVDADLTAVSTEMWTERSGGETS
QTRQKQSFAGDESKDNALASEARLDVSSVGDGSEWDGGEESVESGWECVEVESRNNTTSVIVSVQGSSETAGEAAVVC
STAEERKEEVRGDNQSVTTMTADSQTDAAAAEHPGKVIVTDVTLNSLTVTFKEAMVAKGFFKGY

>Apo_LOC110961504
MELSAVGESVFAAESIIKRRIRRGRWEYLVKWKGWSQKYSTWEPEENILDARLFAAFEERERERMFGPKKRGPKPET
FLLKAKEKAKEKTYEFRREAPRGIQVSYPVPEPIVTPRAREGLRTVVPTIFPPSAINRGESVHVGPPEPQRRPRPTPS
APLAAQEPLRFPKKRGRKPKLHLHFDKEDGSSSVEPEAKRSRLLEEPLAHGLSKMSQRLHHHHHHHHLHHHHGETSDH
SLLQLTKRFQKETTITPKSSSEERNAAGAGLSYSCAFRPGVRKSDQGAHRTHCLSRMYKLKQHGGHGSHDREQPEVIS
SSQSAHDPSARPSTSSWTPCFTNLDTVTVTDVTMNLLTVTIRESSTDKGFFREKR

>Apo_LOC110963666 / LOC110953509 / LOC110945580
MELSAVGERVFAAESIIKRRIRKGRIEYLVKWKGWSPKYSTWEPEENILDSRLFAAFEQRERERELYGPKKRGPKPKT
FLLKAQAKVKAKSYEFRSEAVRGMHITYPTPEPVVTPRAREGLRAVVPTIFPPSTINRGESVRIRQPELSSREHQQPL
LQQTTSDGLIHMPKKRGPKPKPRFKDGSLGPAISEPHKRRAEEQMSHGPHKLSKLHGGEEMRLVKLAHRHPENHGHSH
KHHHHHHHHHHQHHMHSRGISSGSAYKQLYSHPHRTDMDSHRTKDSSGYLAPAHFKHHSSKMSLSRPVELPQMEKPYF
LDRPSPARLDDDLDEVTWRPSLGNVEKVLVTDVTTNFLTVTIKESSTSKGFFKDKR

>Aoc_LOC111568440
MEGVTVGQVFDAECILSKRPRKGKFEYLVKWRGWSSKHNSWEPEENILDPRLLAAFHKREQERELLFQKKGKRPRGRP
RKILPAPAATKDSRSSSSSSSGLSSSASSSSSEDEDPTKKPKPGPRLHPVPQKRPQIMLAKPDPPRKKKRGRKPLHPD
LRALRQAKPRPPAPLPPPVAPRHHQAVRRDDPRPGVKKPLQPASFTYTGLGRSSRDDGSQTSSSFSQTSTSKPGSLSC
IWTNRSLSSSSSSSAHKSSPSPQSKNSLSDLKRSISETSRDGFKVSLKQGGGSLGLHGGFGPTAHRSPLSQRRQDGGP
TGPVPHKQQNSSLSKPSSSASPTPRDRANQALSLRALNLQSVKPPAGSLQSNTSGAMAAVARSSLRSGGIVVKGGVGK
EVRMSGGQRSGLTAGGTTEQNRVKEKLAGGGSGRGGGRQDKHGLSSQNRSLNELSTGDSDDSSSSESEQDAAMFPGNS
RPSLGNNTTESDTETDWRPARSLLEHVFVTDVTANFITVTVKESPTSVGFFNSRNH

>Aoc_LOC111562895
MEELSAVGEQVFDAECILNKRLRKGKLEFLVKWRGWSSKHNSWEPQENILDPRLLAAFNKKEQEKELLMRKRGKRPRG
RPRKILENVPEAPKSSSSSSGSSSSSSDSSSSCSSSSSSSSDDEDDNSHVKQANPSVRTRDLHPVPQKKAQIVMAKQEP
PKKRSRKPLPSEMKEFPQNKGPRKVLKTTKDEDLPGAIKKPVHPASFTFMGFHRGSAREAVGGQNRSPLAQGGAVKNS
TSSVGSGKSSVQPASLSMNKSNQSKNVTEGKLSISNVGSGTSLDLKAAASKSKGVAALNLNTSKHPIQGTTQHTLSSP
NGQKKPQAPVSTVQRIPNTKPSLHSKNASSNQGSGLQPLNLQNKGVQSNDAPGNGTTPTSGPRNATNPARKTTVTQNQ
ECNPPKSPVTPGRPPARKTQPGADKVREVNEIQTSRVQGRLEKSSVHKSSTEVQGQHERSASKDGKKAKMNDMSTGEE
ESSSDSDQDSSYAGQGHSVGVQNQDWKPTRSLIEHVFVTDVTANLVTVTVKESPTSVGFFSIRNY

>Aoc_LOC111577364
MELPAAGEHVFAVEGIEKKRIRKGKIEYLVKWRGWSPKYNTWEPEENILDPRLLVAFQHRERQEQLMGYRKRGPKPKH
LLLQVPSFARRSSIPAGFEETSPDAESSPKSDPVQVQRSQPQQYQLNSKKHHQYQPSSQEVPADQFTSSKKKFIYQLN
SKKHHHYEPDPNMYDAQASRLKEVVKVQEPVSKPANPGWNLPLALQQKWVRDKDTGCLSKVKELAVEVRKPAVKEAES
EQALKPNPKDATLPSSISSKMKIIKNKNKNGRIVIVMSKYMDSNKVHGAKGKHGESSREEKPQNTKPSENNPAHRTKM
AEHPENGIPKEICSGSSLAAAEHPIKCSPKDRQFSKPSPSTAEEYNTEVARGQADLPDDLPLQLTASSPPTSWTVDTN
ILTPTAVDQIRIPSFPSDRKRKLSDPVEDRSVAKIYLTSRSFSVPSTVATPPQDKPMDLHCSGSRQSSLCSYEALGSQ
EEPMDLSCPKPKKQVEPEAQPEPVPEPEPEPAVRDSPPVTDDTQTSTTEKSKEAPVKKVSPFMGNIIITDITTNSLTV
TFKEYVSF

>Aoc_LOC111577320
MELPAAGEHVFAVESIEKKRSRKGRVEYLVKWRGWSPRYNTWEPEENILDPRLLDAFQDRERQEQLMGYRKRGPKPKH
LLVQVPSFARRSSILADLHGASLDDDDCQKSSPIQMLRPQAQQYQLNSSKHHQYQSLCREAEQQPNGKKFYYQLNGKK
HHHYQPDLKVHEPLFVKPRDVKAPELANKGYNLPPVLQQKWVRDKDSGCLTKVKDITMELKKLPADLNGHKEPEKAKP
KEDTLPQSNGVSSSKLKIVKNKNKNGRIVIVMSKYMENGMQAAKIKNGDSESGEKSPQGTDCSPENQLEKMKLVKKLG
LMNGFAKNPKDKPTGFNGDCPKAKEQSPKTEPTVTEQDKHVEVRGQGQLPADQPLQLTTKPNLLPLTSDQGVSSLPDK
RVSQGGFQGLKRHLSDMDGKEHGGTKRFLSCRSISAPNAASLPAQSISVDQNGHQSHDRLQDCGYTDQEEPIDLSIVK
SRPTATQPETQTQAEALTQDLTQTDVQTENQPQTEAQKPAEEKKVDSVSVSNHEKRKEETFPSFQPFLGNIVITDITT
NCLTVTFKEYVTA

>Aoc_cbx6a (ENSAOCG00000009696)
MEPSAAGDRIFAAEAILKRRVRKGRIEYLVKWKGWAMKHSTWEPEENILDDRLILGFEQKERERELHGPKKRGPKPKN
FVMKARGQKRETSSRASSSRQNTPRSSSSTSSRAASSSSSALPHHLPSSSSSSSTVAPSPKLNSLAATHKLKKDIHRC
HRMSRRPLPRSDPMAATFSNPGGFPSRMHVSPFSETVRILNRRVKPREIKRGRIILNLKVVDKPGRGGTASGSRNITA
GRQNIPSRNRIIGKKGEAPYRPFQPPLKMLGFPMYGKPFGLQCGGPVPFHSHPGSCSTTVARDRRTTSSQYQSPPSPS
SSSGSEGKSPTNATAAQPQPAAGASPSSKDAKSSKPHTETQKGQSAKLSAPAPSSDGNPAASASPFLPSSPSSSLEDE
EEEGAQDHSVSSEGERKNPRQRRAKRQPPTTLPSVAPGDQSSAPAEPQRVPAEGDPDWHPEMAPSCKDVVVTDVTTNL
VTVTIKEFPSPASGPASPSSSPENASSPPPVTTSEDPSPAKP

>Aoc_Cbx7 (Gene ID: 111574404)

MELSAIGEQVFAVESIVKKRVRKGNVEYLLKWKGWPPKYSTWEPEEHILDQRLVQAFEEKEQRDRAVGHRRKGSKAKR
LLLENTVYTMDLRSAHKTPEKPPPRLRLSLTRSLVSEDEDQPYSVHRRDPHPHLAHQKSSRSQFRCLNSNSPSPTQED
WEGWGDEEEEEEEEEENNAEDDEDREEEEEAQKETAKMRDSVLNGQERTDDWSSAVRPVQVTTAEKLDDVWRPVIGPGE
VTVTDVTLNSLTVTFRESRVAKGFFRDWGLEV

>Aoc_LOC111570338
MELSSIGDQVFAVESITKKRVRKGNVEYLLKWQGWPPKYSTWEPEDNILDPRLVLAYEENQEKVRALAYRRKGLRPRR
LVLRNIFAMDLRSAHKVSEKPSSRLRLSLTRSMSTDVDQVERGGMYRRPNRRRSRQRLNRSLTEDPANKPMCPQRKKE
EAMEEDWDGTSEEDKQESECTTEERCEDSLYGQSECSSPPLLERQDLEMEVEEKVDADLTAVSTDMWTERSGGETSQT
RQKQTFAGDESKDGALASEARLDVASVGDGSEWDGGEESVESGWECVEAESRNNTTSVIVSVQGSGETAGDAAVVCSA
AEERKEEVKGDNQSVTTMAADGQTDPAAAEHPGKVIVTDVTLNSLTVTFKEAMVAEGFFKGY

>Aoc_LOC111577361
MELSAVGESVFAAESIIKRRIRRGRWEYLVKWKGWSQKYSTWEPEENILDARLFAAFEERERERMFGPKKRGPKPET
FLLKAKEKAKEKTYEFRRETPRGIQVSYPIPEPVVTPRAREGLRTVVPTIFPPSAINRGESVHVGPALERRPRPTPPA
PLAAQEPLRFPKKRGRKPKLHLHFDEEDGSSSVEPEAKRSRLLEEPLTHGLSKMSQRLHHHHHHHHHHHHHHGETSDH
SLLQLTKRFQEETTITPKSSSEQRNAAGAGLSYSCAFRPGARKSDQGAHRTHCLSRMYKLKQHGGHRSHHREQPAVIS
SSQSVHDPSARPSASSWTPCFTNLDTVTVTDVTMNFLTVTVRESSTDKGFFREKR

>Aoc_LOC111577322
MELSAVGERVFAAESIIKRRIRKGRIEYLVKWKGWSPKYSTWEPEENILDSRLFAAFEQREREREELYGPKKRGPKPKT
FLLKAQAKVKAKSYEFRNEAVRGMHITYPTPEPVVTPRAREGLRAVVPTIFPPSTINRGESVRLRQPELSSREHQQPL
LQQTTSDGLIHMPKKRGPKPKPRFKDGSLGPAISEPHKRRAEEQVSHGPHKLAKLHGGEEMRLVKLAHRHPENHGHSH
KHHYHHHHHHHHHMHSRGISSGSAYKQLYSHPHRTDMDSHRTRDSSGYLAPAHFKHHSSKMSLSRPVELPQMEKPYFLD
RPSPARLDDDLDEVTWRPSLGNVEKVLVTDVTTNFLTVTIKESSTSKGFFKDKR

>Ape_Cbx2 (ENSAPEG00000019197)
MEELSAVGEQVFDAECILNKRLRKGKLEFLVKWRGWSSKHNSWEPQENILDPRLLAAFNKKEQEKELLMRKRGKRPRG
RPRKILENVPEAPKSSSSSSGSSSSSSDSSSSCSSSSSSSDDEDDNSHVKQANPSVRTRDLHPVPQKKAQIVMAKQEP
PKKRSRKPLPSEMKEFPQNKGPRKVLKTTKDEDLPGAIKKPVHPASFTFMGFHRGSAREAVGGQNRSPLAQGGAVKNS
TSSVGSGKSSVQPASLSMNKSNQSKNVTEGKLSISNVGSGTSLDLKAAASKSKGVAALNLNTSKHPIQGTTQHTLSSP
NGQKKPQAPVSTVQRIPNTKPSLHSKNASSNQGSGLQPLNLQNKGVQSNDAPGNGTTPTSGPRNATNPARKTTVTQNQ
ECNPPKSPVTPGRPPARKTQPGADKVREVNEIQTSRVQGRLEKSSVHKSSTEVQGQHERSASKDGKKAKMNDMSTGEE
ESSSDSDQDSSYAGQGHSVGVQNQDWKPTRSLIEHVFVTDVTANLVTVTVKESPTSVGFFSIRNY

>Ape_ENSAPEG00000010635
MEGVTVGQVFDAECILSKRPRKGKFEYLVKWRGWSSKHNSWEPEENILDPRLLAAFHKREQERELLFQKKGKRPRGRP
RKILPAPAATKDSRSSSSSSSGLSSSASSSSSEDEDPTKKPKPGPRLHPVPQKRPQIMLAKPDPPRKKKRGRKPLHPD
LRALRQAKPRPPAPLPPPVSPRHHQAVRRDDPRPGVKKPLQPASFTYTGLGRSSRDDGSQTSSSFSQTSTSKPGSLSC
IWTNRSLSSSSSSSAHKSSPSPQSKNSLSDLKRSISETSRDGFKVSLKQGGGSLGLHGGFGPTAHRSPLSQRRQDGGP
TGPVPHKQQNSSLSKPSSSASPTPRDRANQALSLRALNLQSVKPPAGSLQSNTSGAVAAVARSSLRSGGIVVKGGVGK
EVRMSGGQRSGLTAGSTTEHNRVKEKLAGGGSGRGGGRQDKHGLSSQNRSLNELSTGDSDDSSSSESEQDAAMFPVNS
RPSLGNNATESDTETDWRPARSLLEHVFVTDVTANFITVTVKESPTSVGFFNSRNH

>Ape_cbx4 (ENSAPEG00000011118*)
MLILCLVSSLRFPHLHEDPASLQVFEETSPDAESSPKSDPVQVQRSQPQQYQLNSKKHHQYQPSSQEVPADQFTSSKK
KFIYQLNSKKHHHYEPDPNMYDAQASRLKEVVKVQEPASKPANPGWNLPLALQQKWVRDKDTGCLSKVKELAVEVRKP
AVKEAESEHALKPNPKDATLPSSISSKMKIIKNKNKNGRIVIVMSKYMDSNKVHGAKGKHGESSREEKPQNTKPSENN
PAHRTKMPEHPENGIPKEICSGSSLAAAEHPIKCSPKDRQFSKPSPSTAEEYNTEVARGQADLPDDLPLQLTASSPPT
SWTVDTNILTPTAVDQIRIPSFPGDRKRKLSDPVEDRSVAKIYLTSRSFSVPSTVATPPQDKPMDLHCSGSRQSSLCS
YEALGSQEEPMDLSCPKPKKQVEPEAQPEPVPEPEPEPAVRDSPPVTDDTQTSTTEKSKEAPVKKVSPFMGNIIITDI
TTNSLTVTFKEYVSF

>Ape_ENSAPEG00000015378
MELPAAGEHVFAVESIEKKRSRKGRVEYLVKWRGWSPRYNTWEPEENILDPRLLDAFQDRERQEQLMGYRKRGPKPKH
LLVQVPSFARRSSILADLHGASLDNDDCQKSSPIQMLRPQAQQYQLNSSKHHQYQSLCREAEQQPNGKKFYYQLSGKK
HHHYQPDLKVHEPLFVKPRDVKAPELANKGYNLPPVLQQKWVRDKDSGCLTKVKDITMELKKLPADLNGHKEPEKAKP
KEDTLPQSNGVSSSKLKIVKNKNKNGRIVIVMSKYMENGMQAAKIKNGDSESGEKSPQGTDCSPENQLEKMKLVKKLG
LMNGFAKNPKDKPTGFNGDCPKAKEQSPKTEPTVMEQDKHVEVRGQGQLPADQPLQLITKPNLLPLTSDQGVSSLPDK
RVSQGGFQGLKRHLSDMDGEEHGGTKRFLSCRSISAPNAASLPAQSISVDQNGHQSHDRLQDCGYTDQEEPIDLSIVK
SRPTATQPETQTQAEALTQDLTQTDVQTENQPQTEAQKPAEEKKVDSVSVSNHEKRKEETFPSFQPFLGNIVITDITT
NCLTVTFKEYVTA

>Ape_ENSAPEG00000011123*
MELPAAGEHVFAVEGIEKKRIRKGKIEYLVKWRGWSPKYNTWEPEENILDPRLLVAFQHR

>Ape_Cbx6a (ENSAPEG00000009256)

MEPSAAGDRIFAAEAILKRRVRKGRIEYLVKWKGWAMKHSTWEPEENILDDRLILGFEQKERERELHGPKKRGPKPKN
FVMKARGQKRETSSRASSSRQNTPRSSSSTSSRAASSSSSTLPHHLPSSSSSSSTVAPSPKLNSLAATHKLKKDIHRC
HRMSRRPLPRSDPMAATFSNPGGFPSRMHVSPFSETVRILNRRVKPREVKRGRIILNLKVIDKPGRGGTASGSRNITA
GRQNIPSRNRIIGKKGEAPYRPFQPPLKMLGFPMYGKPFGLQCGAPVPFHSHPGSCSTTGARDRRTTSSQYQSPPSPS
SSSGSEGKSPTNATAAQPQPAARASPSSKDAKSSKPHTETQKGQSAKLSAPAPSSDGNPAASASPFLPSSPSSSLEDE
EEEGAQDHSVSSEGERKNPRQRRAKRQPPTTLPSVAPGDQSSAPAEPQRVPAEGDPDWHPEMAPSCKDVVVTDVTTNL
VTVTIKEFPSPASGPASPSSSPENASSPPPVTTSEDPSPAKP

>Ape_Cbx7a (ENSAPEG00000011936)
MELSSIGDQVFAVESITKKRVRKGNVEYLLKWQGWPPKYSTWEPEDNILDPRLVLAYEENQEKVRALAYRRKGLRPRR
LVLRNIFAMDLRSAHKVSEKPSSRLRLSLTRSMSTDVDQVERGGMYRRPNRRRSRQRLNRSLTEDPANKPMCPQRKKE
EAMEEDWDGTSEEDKQESECTTEERCEDSLYGQSECSSPPLLERQDLEMEVEEKVDADLTAVSTDMWMERSGGETSQT
RQKQTFAGDESKDGALASEARLDVASVGDGSEWDGGEESVESGWECVEAESRNNTTSVIVSVQGSGETAGDAAVVCSA
AEERKEEVKGDNQSVTTMAVDGQTDPAAAEHPGKVIVTDVTLNSLTVTFKEAMVAEGFFKGY

>Ape_Cbx7b (ENSAPEG00000020659)
MELSAIGEQVFAVESIVKKRVRKGNVEYLLKWKGWPPYSTWEPEEHILDQRLVQAFEEEQRDRAVGHRRKGSKAKRLL
LENTVYTMDLRSAHKTPEKPPPSLRLSLTHSLVSEDEDQPYSVHRRDPHPHLAHQKSSRSQFRCLNSNSPSPTQEDWE
GWGDEEEEEEENNAEDDEDREEEEEAQKETAKMRDSVLNQERTDDWSSAIRPVQVTTAEKLDDVWRPVIGPGEVTVTD
VTLNSLTVTFRESRVAKGFFRDWGLEV

>Ape_Cbx8a (ENSAPEG00000011079)
MELSAVGESVFAAESIIKRRIRRGRWEYLVKWKGWSQKYSTWEPEENILDARLFAAFEERERΕREMFGPKKRGPKPET
FLLKAKEKAKEKTYEFRRETPRGIQVSYPIPEPVVTPRAREGLRTVVPTIFPPSAINRGESVHVGPAPERRPRPTPPA
PLAAQEPLRFPKKRGRKPKLHLHFDEEDGSSSVEPEAKRSRLLEEPLTHGLSKMSQRLHHHHHHHHHHHHHGETSDH
SLLQLTKRFQEETTITPKSSSEQRNAAGAGFDQGAHRTHCLSRMYKLKQHGGHRSHHREQPAVISSSQSVHDPSARPS
ASSWTPCFTNLDTVTVTDVTMNFLTVTVRESSTDKGFFREKR

>Ape_ENSAPEG00000015391
MELSAVGERVFAAESIIKRRIRKGRIEYLVKWKGWSPKYSTWEPEENILDSRLFAAFEQREREREELYGPKKRGPKPKT
FLLKAQAKVKAKSYEFRNEAVRGMHITYPTPEPVVTPRAREGLRAVVPTIFPPSTINRGESVRLRQPELSSREHQQPL
LQQTTSDGLIHMPKKRGPKPKPRFKDGSLGPAISEPHKRRAEEQVSHGPHKLAKLHGGEEMRLVKLAHRHPENHSHSH
KHHYHHHHHHHHMHSRGISSGSTYKQLYSHPHRTDMDSHRTRDSSGYLAPAHFKHHSSKMSLSRPVELPQMEKPYFLD
RPSPARLDDDLDEVTWRPSLGNVEKVLVTDVTTNFLTVTIKESSTSKGFFKDKR

>Nco_Cbx2 (Gene ID: 104943133)
MELPAAGEHVFAVESIEKKRSRKGRFEYLVKWRGWSPKYNTWEPEENILDPRLLDAFQDRERHEQLMGYRKRGPKPKH
LLVQTKRRDFNAASVT

>Nco_LOC104963701
MEELSAVGEQVFDAECILNKRLKKGKLEYLVKWRGWSSKHNSWEPQENILDPRLLAAFNKKEQEKELLILKKGKRPRG
RPRKILENVPEATKSSSSSSGSSSSSSDSSSSCSSSSCSSEDDDDDSNVTPATPNVRTRDLHPVPQKKAQILVAKQEP
PKKRSRKPPSPEVKEFQQNKGPRKVLKASKDADLPGAIKKPVHPASFTFMGFHRGSPRDTVGSQNKSSVTQGGAVKSS
VGSERSVQPASLSLNKSSQSRNVTEGKLSISSVSCRTSLDLKAAASKSKGVAALNLNTSKHLIQGTTQHTLSSPNGQK
KAQAPASTPQRLPNSKAVAALPSKNSLSNHGSGPQPLNLQNKLVKSNAVPGNGTAPVSGLRNATLPARKITALQDYNP
PKSPANPGRLQARKSQSGVDMVKEATEIQTPRVQGRLVKGSVQKSSTEVQIQQDRLAPKDGKKAKINEMSTGEEESGS
DTDQDSSYVGQDGSVMVQNQDWKPTRSLIEHVFVTDVTANLVTVTVKESPTSVGFFSNY

>Nco_Cbx4 (Gene ID: 104965337)£
MELPAAGEHVFAVEGIEKKRIRKGKIEYLVKWRGWSPKYNTWEPEENILDPRLLVAFQHRERQEQLMGYRKRGPKPKH
LLLQVPSFARRSCIPAGFTDSSQDAEVSLKSDPIPVQRPQAQQYQLNSKKHHQYQPSTHEVPADQPINGKKKFIYQLN
SKKHHHYEPDPNMYEAQASRLKEVVKVQEPGSKPANPGWNLPLALQQKWIRDKDTGCLSKVKELAVEVRKPAVKDAES
EHALKPNPKDATLPSSISSKMKIIKNKNKNGRIVIVMSKYMDGKVNGAKGKHGESSSEVKLQNAKPSENSPAHTTKIV
EHPENGIPKELCNGSSLPAAEHPIKCSHKDRHFSKPSPSTAEEYNTEVARGQADLPEDLPLQLTASSPLTSWSVDSNI
LTPTSVETIRIPSFPNDRKRKLSDPVEDRSISKTYLSSRSFSVPSTVVTPPQDKPMDLHCSGPRHSSTCTFEVVDSQE
EPMDLSCPKTKTQVEPEVHPEPEPAVTNTPPVVEETQKSTEKCKEAPVKKVSPFMGNIIITDITTNSLTVTFKEYVSL
CSVDLSDFACPPVPDIPLQQPSSGAPPPHSETGARSNNRANVRSVLPTAVPGNRKRRDDVLSRLRIMGI

>Nco_LOC104943130
MLLCFFIDTHHSLVPQVPSFARRSSILADLHEASLEEESCLKPSPMQMLRPQVQQYQLNSKKHHPYQPLCRERDTEPP
AHGKKFFYQLNSKKHHQYQPDLKGHDPIFVKPREVKAPELVNKGYNLPPVLQQKWVRDKDSGCLTKVKDITMELKKLP
ADLNGHNEPEKVQPKEDASLTQSNGGSNGKLKIVKNKNKNGRIVIVMSKYMENGMKAAKIKNSGSDIAGKPLQPADSS
TENHLEKMKLVRKLGLMNGFAKNPKDKTTFPSSGFKVHCLKEKQQSPKTEPTVTDQDKPVRVRGQGQLSEDQPLQLTS
CKGTTKPNLLSLPLDRGVPSPLDKRGNQGEFQGLKRHLSDPDSEDHGSSKRFLSCRSISPPHATSPPTQSTNQNTHQS
PTGLQDCGYADQEEPIDLSVVKSRPKATQPETQTETVTQSETRTHDETDTHTDVRTETETTEEDKVNSLSVCNHEKRK
EETFPSLQPFLGNIVITDITTNCLTVTFKEYITA

>Nco_LOC104966035

ILCPPPHLKVIDKPVRGGASGRNHTVGGRQNIPSRNRIIGKKAEGPYRPFHSPLKMLGFPMYGKPFGLQCGGPVTFSS
AQYNSAPSQHNSSPSPCKPSPSQHNFSPSSSEGKSTTGVSLPPSSEAPESSNPVGASSSSSVKDQEDETPKGRKRQLP
DDQSPGNETPAEPQIAEGNPNWHPEMAPSCKDVVVTDVTSNLLTVTIKEFPSQNTAQSEDPET

>Nco_LOC104968319
MELSAIGEQVFAVECILKKRVKKGNVEYLLKWKGWPPKYSTWEPEEHILDQHLVKAYEEKEQKEKQLGHRRKGPKAKR
LFLQ

>Nco_LOC104958407
MERSSVGLIGDQVFAVESITKKRVRKGNVEYLLKWQGWSPKYSTWEPEDNILDPRLVLAYEEHQEKIRALAYRRKGLR
PRRLLLRVRRHLYPHLTLLYLSLPILTAH

>Nco_Gene ID: 104965337)£
MELSAVGESVFAAESILKRRIRRGCWEYLVKWKGWSQKYSTWEPEENILDARLFAAFEERERERELFGPKKRGPKPET
FLLKAKAKEKTYEFRREAPRAIQVSYPTPEPVITPRAREGLRTVVPTIFPPSAVNRGESVQIRPPEPERRPRTTPSAA
LTDLELGVRIPKKRGRKPKLHLHFNETVDGGLSEEPNTKRSRSLEEPVSHGFSNMSRHLLHHGEASDHSLMQLTRRFE
EKTTITPKHSSSEQRHAGLSFSAFNSDVRKRDQLKHMTKCLMSVRRPSSSAEHRKHQVKECSQPAVRSTENSDKATQP
ASCWTPRLTNLDTVTVTDVTMNLLTVTVRESSTEKGFFREKR

>Nco_Cbx8 (Gene ID: 104943131)
MELSAVGERVFAAESIIKRRIRKGRIEYLVKWKGWSPKYSTWEPEDNILDSRLFLAFEERERERELFGPKKRGPKPKT
FLLKAQAKIKAKSYEFRGDAVRGMHITYPTPEPVFTPRAREGLRAVVPTTFSPSTVNRGESMRIHPPELSTREYQQPS
LQISGPDELIHLPKKRGPKLKPRFKDGSCSPAIFEPHKRRAEEQGSHVPHKLTKLQGGQEMRRIKVAHRHPEIHSHKC
HHHNHHHHHHSNNRGFSGGSSFNQLYSERSLHPHRTDSHRTKDSSSHLAPVTFKHQSRMSPSLGRPVEPPQMEKPYFM
DRPSPTRFDLDVDLDEVTWRPSIGNVEKVMVTDVTTNFLTVTIKESSTTKGFFKDKR

>Cse_LOC103382231
MEGVTVGQVFDAECILSKRPRKGKFEYLVKWRGWSSKHNSWEPEENILDPRLLAAFHKREQERELLFQTKGKRPRGRP
RKIQPTPPTTKDSRSSSSSSSGLSSSASSSEDEDPSKKAKPTPRVHPVPQKRPQILLAKPDHKKKRGRKPLHPDLKAL
RQAKSKLPPLSPPPPPPSPSPSSLPPPGLAAPQRHHPSVRPSREELRAGVKKPLQPASFTYTGLGRTSRNEASSASNS
AFVQSVSPKPGALSCAWTNRSLSSPSPGSSHKATPSAQSKHMTSELKSSSRSDGFKTSPLKHGSGTSGSGSLHGTFDR
AAISQRSQSNQRRQDGVSAQTGSGQHKQIPQISSPRDRANQALSLRALNLQSVSRPTCGAVFQGNNTGGAAVSRSSSR
SSGIMVKSGVKDGRPSSCGQRSGTNMVVGEHIRGKEVTPGGGASRGNGSSANRSLNELSTGDSDETSSSESEQGVPLY
PAANSRPSLGSSTADSDTETDWRPARSLLEHVFVTDVTANFITVTVKESPTSVGFFNSLNH

>Cse_LOC103392533
MEELSAVGEQVFDAECILNKRLKKGKLEFLVKWRGWSSKHNSWEPQENILDPRLLAAFNKKEHEKELLLRNKGKRPRG
RPRKIVKNQVPETPKSSSSSSGSSSDSSDSSSSCSSSSSEEDDDDNDVEQANQIVRTRENHPVPQKKAEIVVAKQEPP
NKRSKKPATPEGNELPHKRGLRKNPKMSNDTDHPGAIKKPVQLASYTLMGFQQGLGRDTVGQGVGSLGHGGPQKSAVG
SSKSTQSPSLSLYKSNKSRDVTEGKTSGSSKDTEKHLELNTSSGKSNGVAALSLNTGKNPSQANVRHSLSSPNGQKKP
QGSVSGLQRIPHAKAVGFGPSKNNNSSQGSGFQPLNLQNKPAQNSTTSENEATLLSGTKIAPKPARKANAPQNQDCNP
NKSPESPCRLEAGKNYSEANKTKEAAEIRSTHNQGRLEKSSVHTFPQQEKFAYKTGRKMNDMSTGEDETSSDSDQDST
CSGKDSSMNQTQDWKPTRDLIEHVFVTDVTANLVTVTVKESPTSMGFFSVRNY

>Cse_LOC103384237
MEVAAAGEHVFAVESIEKKRSRKGRVEYLVKWRGWSPKYNTWEPEENILDPRLLDAFQLRERQEQMMGYRKRGPKPKH
LLVQVPSFARRSSILNDLHEASLDEEDEKCDSTSPVHVLRPQGTQYQLNSTNHHKYQSLCREKEMEPPTNGKKFFYQL
NSKKHHHYQPDVKDFKVHEPTFVKPPEVKVPDLANKGYSLPPVLQQKWIRDKDSGCLTKIKDITMELKKLPANLNGHR
ESQSTGLKEDSLPQSNGVSSSKLKIVKNKNKNGRIVIVMSKYMENGMQSAKIKNGDSEPTGNPESGAENHSEKMRLVQ
KLGLTNGFVKNSGDGSTVSTSGFNGDAPQEREQSTKMQPTVTEQAKHVGVRVQGQLPADQLLQLTTKSNLPSLPVDRG
VPSALDKRETQGGCQRLKRPLHDTGTEDCGTTKRFLSSRSFLDASAAPSPTQSTSVDQNGQRSLEGLQVCGYTDQEEP
MDLSMVKSRANSEACTSAQPQAQTTVETLVCSQDEKHTQTQTDSQRETQTPTETRDTSEELDVDSLSEPNKEKRKEET
FPSFQSFLGNIVITDITTNCLTVTFKEYVTV

>Cse_LOC103393067
MELPAAGEHVFAVEGIEKKRIRKGKIEYLVKWRGWSPKYNTWEPEENILDPRLLVAFQHRERQEQQMGYRKRGPKPKH
LLLQVPSFARRSSIPAGFEEASQETEGGVKSDPVQVQRPQIQQYQLNSKKHHQYQPSSPEVSADQLANGKKKFIYQLN
SKKHHHYEPDPNMFDTQASKLREVVKVQEPACKQANPGWNLPLALQQKWVRDKDTGCLSKVKELAVEVRKPVVKEAES
EHALKPDPKDATLPSAVSSKMKIIKNKNKNGRIVIVMSKYMDGNKVHAAKGKHGESSGEEKPMSTKPSESNPGQRTKI
GEHLENGVPKEICNGRSPPAAEHPIKCSPKNRHFSKPSPSTAEEYNTEVARGQADLPDDLPLQLTASSPSMSWAMDTN
IPTPTAVDQIRIPSYPGDRKRKLSDPVEDRGVSKTFLASRSFSVPSTVVPPPQDKPMDLHCSSRRPSSNCTYDFVDSG
GQDEPMDLSCPKTKRQTEPELRPEPQPEPEIEPEPVQKSETQPADAEAPPVMEDTPKSAEKSKEAPVKKISPFMGNII
ITDITSNSLTVTFKEYISF

>Cse_Cbx6 (Gene ID: 103383217)
MEPLAAGDRIFAAEAILKRRVRKGRLEYLVKWKGWAMKHSTWEPEENILDDRLIMGFEQKERDREKSGPKKRGPKPKH
FMKARGQKRDPSSQNASTRQSVSRSASSRAAPSSSTSTSSSSAAATPHLPSLSSSSSAIGPSPKLNSLAATHKLKKDI
HRCHRMSRRPLPRSDPMASSFSSAGGLPSRMHVSPFSETVRILNRRVKPREVKRGRIILNLKVTDKPGRGGASAGSRN

ATSGRQNIPSRNRIIGKKGEAPYRPFQPPLKMLGFPMYGKPFGLQCGGPVPFHSNSGSRSSAAVRNNHAAASQLKTPP
SPSSSSSSEGKSSPTVASQSRPGSSAPPSSHDPEPSDPPTETEKGHGANISDPSFTCENTAVPSSPFLPSSPSSSLED
SVEQEDKEAVDSNEPHQCNANIPSPTAAPAAAPPASQSANPSEPQRVPVEGDPDWHPQIGPSCKDVVVTDVTTNLVTV
TIKEFPSPASGPASPSSAPENASPPPPAISSDDPSPAKP

>Cse_LOC103382143
MELSAIGEQVFAVESIVKKRVRKGNVEYLLKWKGWPPKYSTWEPEEHILDQRLVQAYEEKEQRDRALGYRRKGAKGKR
LLLQDTVYTMDLRSAHKAPEKPPHLRLSQSQSQVSEEDEEDESYGSSRYDLRHQDEKSRTSLIKYLDSDPPSPSQEDW
NEEEEEEEEEEEEEEEEDHVEEEEEETVVVETETTDGDVTEKTDRWCSTITPDEVTVLEDSWSPVISPGDVTVTDVTLN
SLTVTFRESRASRGFFRGWGLEV

>Cse_LOC103393332
MELSSIGDQVFAVESITKKRVRKGNVEYLLKWQGWPQKYSTWEPEDNILDPRLVLAYEENQEKIRALAYRKKGLRPRR
LVLRNIFPMDLRSTNKVPEKPPPRLRLSLTRSMSTDVDGRTIRSRQRRPVPKRVLEGQSNRRVGALRKKVEEEPTEED
WSSNSEEEKQELESATEERREDSVYGQSECSSPPQLERQDSEMEVEDKVDASLTLVGSDTWTDGSDGGTCETRRDQIF
VFDQSKDDSVTPVVIPADPVREESEVDEGEADIKSHSDGLRLDTSDTTTVIMGVEERSQTTGETPSNTAVCSSTEVKE
EEEKEAKDDNQSALTMLPGDGSPPASPERPGKVIMTHVTINSLTVTFKEATVAEGFFKGY

>Cse_LOC103384236
MELSAVGERVFAAESIIKRRIRKGRIEYLVKWKGWSPKYSTWEPEENILDQRLFVAFEQREREREL YGPKKRGPKPKT
FLLKAQAKDKLRSYEFRNESIRGRPISYQTPDAVVTPRAREGLRAVVPTIYPPSTVNRGESICVRQLELSSIEHQEPQ
QTSTGGLVHIPKKRGPKPKPRFKDNVCSSISTLPVSEPQKRRAEEQESHSPHKLAKYQELEEIRLFKAVHRHPENHAH
SRKHHHHHHHHHHQQQQQQQQQQQQHQHHRDQHHYSDPRPHPHRTDLDSHRTKEACTYLMPTHFKNKSKMIPSQRRPA
QLPLMEKPYFLDRPSPTRLDDDMDKVSWRPTLGSAEKVLVTDVTANFLTVTIKESSTSKGFFKEKR

>Cse_LOC103393068
MELSAVGESVFAAESIIKRRIRRGRWEYLVKWKGWSKKYSTWEPEENILDARLFAAFEEREREREQFGPKKRGPKPET
FLLKAKAKEKSYEFRREAPRGIQVSYPVPEPVKTPRAREGLRAVVPTIFPPSAINRGESVHLQPPEPERRPRATPPTA
ASTLQELGRFPKKRGRKPKLQLHYDEDDESSSAEPDTKRARSVQEGDLSNSSRRLHHHHHQHHQHHQHTVKEASDRSL
IQLTKRFQVETTITPKPGGEHRLPGDAALAYTCAFSPDMRTSEPRGHSRPESLRRTCVSQPEKMKHQVKMSCRAPQSQ
STCDFPARAASSWRPSLSNLDRVTVTDVTMNLLTVTIRESSTDQGFFREKR

>Sma_Cbx2 (ENSSMAG00000012696)
MEELSAVGEQVFDAECILNKRLKKGKLEFLVKWRGWSSKHNSWEPQENILDPRLLAAFNKKEQEKELLLRNKGKRPRG
RPRKIPENVPEATKSSSSSSGSSSSSSDSSSSCSSSTSSSEEDDEDGGDVQQAAPSIRTRELHPVPQKKAQIVVAKPE
PPKKRSRKPLSPEVKEFQQNKAPRKILKTSKDADLPGAIKKPVHPASFTFMGFHRGSPRDTAGSHNRGSLTQGGAVKN
SPGSVGSGRSVQPASLALNKSSQSRNVSDGKLSVSGMSGGTSLDLNTAAGKSKGVAALNLNTSKHPIQGTTQHTLSSP
NGQKKPQGPVSALQRMPNTKAVASVPSKNASAIQGSVPQPLNLQNKSAQGKDAPGNGTTPASGLRNATHPARKTTVTQ
NQEYNPPKSPAIAGRLQTRKNPPGADKVKEATEIQSSRVQGRLDKSGVHKASTEVLSQQERAAYKDGKKAKMTDMSTG
EEESSSDSDQDQDSPYAGEDRSLNPNQDWKPTRSLIEHVFVTDVTANLVTVTVKESPTSVGFFSLRNY

>Sma_Cbx4 (ENSSMAG00000014969)
MELPAAGEHVFAVEGIEKKRIRKGKIEYLVKWRGWSPKYNTWEPEENILDPRLLVAFQHRERQEQLMGYRKRGPKPKH
LLLQVPSFARRSSIPAGFEDTCHDAEVGLKSDPVQVQRPQPQQYQLNSKKHHQYQPSSQEVPADQLTNGKKKFIYQLN
SKKHHHYEPDPNMYDTQASRLKEVVKVQEPACKQANPGWNLPLALQQKWVRDKDTGCLSKVKELAVEVGKPAVKEPES
EHVLKPNPKDTALPSATSSKMKIIKNKNKNGRIVIVMSKYMDSNKVHGAKGKHGESSSEEKLQNTKPSENNPAHRTKM
VEHPENGIPKEICNGSSLPAAEHPIKCSPKDRHFSKPSPSTAEEYNTEVARGQADLPEDHPLQLTTGSPPMSWAVDTN
IPTPTAVDQIRIPSFTSDRKRKLSDPVDDRSVSKVYLNSRSFSVPSTVATPPQDKPMDLHCSGRRLGSTCTYEAADAG
SQEEPMDLSCPKTKRQVEPEVQPEPAPAPEPEPVDGDTSPVMEDTEKSTEKSEEEAPIKEISPFMGNIIITDITTNSL
TVTFKEYVSF

>Sma_ENSSMAG00000011638
MELPAAGEHVFAVESIEKKRSRKGRVEYLVKWRGWSPKYNTWEPEENILDPRLLDAFQDRERQEQLMGYRKRGPKPKH
LLVQVPSFARRSSILADLHEASLDQDGCEKSSPIQMLRPQGQQYQLNSKKHHQYQPLCREREAEQQTNGKKFYYQLNS
KKHHHYQPDLKVHEHIFSKPRDVRAPELPNKGLNVPPVLQQKWVRDKDSGCLTKVKDITMELKKLPADLNGHKEPEKV
KPREDALPQSNGVSSKLKIVKNKNKNGRIVIVMSKYMENGMQAAKIKSGDCDDTAEHPRQGTDSGAENHLEKMKLVKK
LGLMNGFARNPKDKPAVPSSGFNVDCPRERHQSPKVEPTATEQDKHVGVRGQGQLPADQLLQLTTKPNLLSLPLERGV
PSALDKRATQGGFQGLKRHLSDTDCEEHGSNKRFLSSRSISAPNAVSPPAQSISIDQNGHRGDVGLQVCGYADQEEPI
DLSVVKSRPIAAASAASQPEKYTQAETLECTRDKTHTQAETQTQADAQTETQLQSETQNAAEKAKVDSLSVSNNDERR
EETFPSFQPFLGNIVITDITTNCLTVTFKEYVTV

>Sma_Cbx6a (ENSSMAG00000021207)
MEFLVKWKGWAMKHSTWEPEENILDDRLLLGFEQKEREREMNGPKKRGPKPKNFVGKARAPKREPSSKASGSRQTTPR
SSSGRATPSSAASARLLPSASSSTIAPTPKLNSLAATHKLKKDIHRCHRMSRRPLPRSDPMASTFSNPGGFPSRMHVS
PFSETVRILNRRVKPREVKRGRIILNLKVIDKPGRGGARNITTGRHNIPSRNRIIGKKGEAPYRPFQPPLKMLGFPMY
GKPFGLQCGGGPLAFHSQTGSCSSTGARDSHTTSSQYQSPPSPSSSSCSEAKTPTTSAARSQSATEASPSSEDPKPSK
PHTETQKGQSAKHSAPAPSSDAKAVGSASPFLPASPSSSLEEEEEEEEGEEGALGRLVSSEGGRKNPRQRRARRPLPAA

LLSVSPGDQSPTPTEPRRVPAEGDPDWHPEMAPSCKDVVVTDVTTNLVTVTIKEFPSPASGPASPSASPENASSPPTA
TTTSDDPSPSKP

>Sma_Cbx7a (ENSSMAG00000006170)
MELSSIGDQVFAVESITKKRVRKGNVEYLLKWQGWPPKYSTWEPEENILDPRLVLAYEENQEKIRALAYRRKGLRPRR
LVLRNIFAMDLRSAQKVAEKPPPRLRLSLTRSMSTDVDQGERSSMFCSQVRRKRVGVKRGPDRPLRPLRTKEEPMEGD
WRDIIEEEKQESESSVEEIREDSLYGRSECSSPPLLERQDLAVEEKVDADLTVEGLETWTDRSDGRVCETFACNQSKD
SAAVPAARAGDAVTAGDRSNWDRGVEEVESGSECPRLERNDTITVIVSVQGSSETAVAAAAAAVVACSTAEEARADNQ
SATTTTTPGSQSAPAAAEHPGKVIETEVTINSLTVTFKEAMVAEGFFKGY

>Sma_Cbx7b (ENSSMAG00000006575)
MSPQGNVEYLLKWKGWPPKYSTWEPEEHILDQRLVQAYEEKEQRDRALGHKRKGSKAKRLLLQNSVYTMDLRSAHKVP
EKPPPRLRLSLTRSLVPEDEDEDEDELYGACTQGPGPRLAPHKGEPGRSQFTCLDSSPPSPAQDDWEGLGEEDEEEED
NVEDDEEEMEAAQKGTTLNGQERTERWSAAVGPDEVAASDDTWRPVVGPAGEVTVTDVTLNSLTVTFRESRVAKGFFS
HWGLEV

>Sma_Cbx8a (ENSSMAG00000014960)
MELSAVGESVFAAESIIKRRIRRGRWEYLVKWKGWSQKYSTWEPEENILDARLFAAFEERERERELFGPKKRGPKPET
FLLKAKAKEKAYEFRREAPRGIQVSYPSPEPVITPRAREGLRAVVPTIFPPSTVNRGESVHVRPPEPERRPRPTPPAA
LTLHESGRFPKKRGRKPKLHLHYDKDNSGGSAEPDTKRSRLAEEPDSHGLSQMARPLHHHGETSDRSLLQLTRRFQEK
TTITPRPSSEQRHAGGAGLSFYTCAFSPDARQSDQRGHRTGCAQGSMSVLKHRVKEFGRPADLSTPTVSTRDSSAPPA
SSCWTPSFTSLDTVTVTDVTTNLLTVTIRESSTDQGFFRDTR

>Sma_ENSSMAG00000011628
NMELSAVGERVFAAESIIKRRIRKGRIEYLVKWKGWSPKYSTWEPEENILDSRLFVAFEQREREREL YGPKKRGPKPK
TFLLKAQAKDRAKSYEFRSEAVRGMHITYPAPEPVVTPRAREGLRAVVPTIFPPSAVNRGESIRVRPPEFGGREYQQP
SLKQTVADELIHVPKKRGPKPKPRFKESGCSPATSEPHKRRAGELLGQGPHKLTKYHGMEELRLFKVAHRHPESHGHG
HKHHHHHHQHHQHHHQHNRELSGGSSYKQLYSDRSPHPHRVDTDTHRTKEGSTYLVPAHFKHQSKVSQSLSRPAEQPL
MEKPYFLDRPSPTRLDDDLDEVSWRPSVGGVEKVLVTDVTTNFLTVTIKESSTSKGFFKDKR

>Ame_Cbx2 (Gene ID: 103045429)
MEELSAVGEQVFDAECILNKRLRKGKLEYLVKWRGWSAKHNSWEPQENLLDPRLLAAFNKREQERELLLQKRGKRPRG
RPRKITEDTVPTVSKSSSSSSSSSGSSSSSSSSSSSSSSSSEEDDDDDDDNDVNGDRKPKPSPRPRELDPVPQKKA
QIVMAKPDPPKKKRGRKPLTPELKALKQSQAKCIRKPMPKDPLSDLRGSIKKPLMPASFTYTGMNRAASREPLAVQSR
GSFTHNSPAKSSPNTQGTGRPAGSLGRLSQGKSTADFKLSDMGSGGLDLKASACKSPGVASLSLHNSKLTACSTNGQA
TGGTPNRTKKQETPTQSPMQRSNSNKPATTALSSVKSSSNLSTSTQPASLHVASKIEKGTGSSVVGVSIQGSVHRNSG
SAVRKGPAQEKSLLINPASQAAGLGMVGTRNNIIPVGIDRVKSEDSSDPAEKLGKANQGRHQKSFPSTLDRNGLKDGT
KAGKTLSEMSTGEEGTSSESEPDSSFSSKSQDLPIGINTSQDWRPTRSLIEHVFVTDVTANLVTVTVKESPTSVGFFG
IRNF

>Ame_Cbx4 (Gene ID: 103045489)
MELPAAGEHVFAVESIEKRRVRKGRFEYLVKWRGWSPKYNTWEPEENILDPRLLVAFQNRERQEQMMGYRKRGPKPKH
VLTQVPSFALRSNILSGLQEASQDNSSYLKMSVDPAQTQQYQLNSKKHHPYQPICKESSGDTLVNGKKKHYYQLNSKK
HHHYQPSTKMYQKVETEPAQTLQQKWVQDKDSGCLGKVRDITMELEKLPASLDGNGKPKLAMKDVTQVSGISSKLKIV
KNKNKNGRIVIVMSKYMENGTLAAKIKNGEVDMGVKQQQKLQENQSSAVTDSVVEKNRHSKKHSPENGWKKDSKDETR
PLPCGLPNGASFPAVDGSVTSSPLAVKTNKSEQKSCSPDLDDQPVQLTTKSSLNPKPLDKGALHHFDQRNVLGALKRR
NSEPDRQQDSEAKKFSTFRSLSAPGTSHCQNNSIHHNGHQHSNGHAMELDCQDEPIDLSCSRARKENNSVINCQINGG
SKRAEHAAGPQSTRTEKEQDLKPVSSFTPFLGNIIITDVTANCLTVTFKEYVTV

>Ame_LOC103031044
MDLPAVGEHVFAVEGIEKKRVRKGRIEYLVKWRGWSPKYNTWEPEENILDPRLLVAFQNRERQEQQMGYRKRGPKPKH
LLIQLPAFARRSSSILAGLQETSLDDENQLKMDCLQMHRSQPQQYQLNSKKHHQYQPNSKEIPAEPHINGKKKHFYQL
NSKKHHHYQPDPKMYDQQPTKPKEVKGHDPSIKGWNLPPALQQKWARDKDNGCLNKVNDLAVEHKQLPATVNKEQQAL
KTSLKEPSLPNGISSKMKIIRNKNKNGRIVIVMSKYMDNGVQSSKVKNGDLSGMEKPHLAEESLNGNRGNGIPVTPGQ
ENGVAKDNGVYSSSATSSNGHKVPHKKIDLPRTKTAVDVDHKADQKMDIIDLSDDQPLRLSTKANSSPVPSVSSETDC
ARYQPGHRKRNLSEPHDGGIDCKRFISSRSISAPNPVFSSPQREPMDLHCSGQNTNTNRSYSYDFTDTIPEEPIDLSC
GRTRLQAETCGNSAETPVTVEEDTPQTSEKSEEPVSYFKPFAGNIIITDVTTNCLTVTFKEYVSI

>Ame_LOC111194212
MFKNLSLTDAHLCPQGRLQYLVKWRGWSAKYNTWEPEENILDPHLLVTFQNREYQEQMMGFRKRGRKPKYHLKQVPSF
ARRPGILSSLQEALSNGKKKHCYHLKSRKHHHNQVDAKMYPKVKEEPKGSWNLPQVLHQQWIQNKVSECLNNVRDITK
ELEKLPACVIGGETSELGSNIIPREAPASGMSSKLKIVKNKNINGRIVIVMGKYAEGGTQTGNSVENESDITGKNRHS
AENDCKEENEDEIELSPCRFTVANMENNLKEEGVTKSIPFLNPSEKSIHLCLDPRSGQGAQKRRFSEPEIEQDSEVKR
FLSSENTGVPISGFSQCQIKNSHIDGRQHSNKQCFELFGCQDEPIDLSYSRKLRSPIPDKSQINRNSKASEKQIESLK
EQTEKEPESTLIQSFTPFLGDVIITDVTTNCLTVTFKEYVTVWK

>Ame_LOC103038090

MELSGMGERVFAAEAILKRRVRKSNIEYLVKWKGWALKHSTWEPEENILDDRLISAFEKKEREQELYGPKKRGPKPKN
LLLKARTQPSPRVSKTRNTPPRSSSSSASSSSSAVVPPVNPVRSSSSSSSSSSLAPTPRLHSLAASHKLKKDIHRCHRM
SRRPLPRYDPDGSSFSFRPPISPFSETVRILNRRVKPREVKRGRIILNLKVIDKCGGRAANRGPTQSHQGRAQIPSRN
RIIGKKQGDMPYRPFQPPMKMLGFPMYGQPFGLQPFPPVSAQPNRRAGSTSGTRGRGGGCGPGRPRKATKFPYQSPPS
PSSSSGSESDASSPPKIQPGPPQASPSETAPSSPPVLSPETPSRGSEPPQNHSSSAKPGSASNPTSFLPSSPSLSSSA
SSSSSPEDEEEKAQGGKRKPCRRIRASEAATSHPAAEKRAERRALKKADPDWRPERSPGCINVVVTDVATNHQTVTIK
EFCKPGAGSKGSTGSSPPSPSQAKNPGSSAPAAT

>Ame_cbx6b (ENSAMXG00000031801) [Sequence reconsititution with LOC103031372]
MELSAAGDRVFAAEAILKRRVRKGRMEYLVKWKGWAIKYSTWEPEENILDDRLVVAFEQKEREQELYGPKKRGPKPKT
LLLKSRAQTSDTPHRVPEFKHTRPQPSSKPAPPPLPAPTYTPTCPSNAKLQSGAAQPKLKKDIHRCHRMSRRPLPRSD
PLSQSVGHSLSPFSETVRILNRKVKPREVKKGRVILNLKMIDKAGSGGSANNKRTQSTTHQSHTARQKVPSRNRVIGK
SRRFGEVSYRGLQLPVSGSGFSMFGKTFNSHPMDNSGNQPAVEHKESGQKSSSKGLSSQSSSQSSKVATSDVPSALNE
PPPSASSSEVSDGESHPTSVAPTKSHQPLSAHDHKSSENNCFKLPSLSLQHNLKDQKQSNQLKDSQPKKSNPSAAQSL
LPSSPMFSSSSSSSSSSEDNEHILDLSVPHGSDRRGKRHYHFCRHRQPKLPEIPISEEASEEEELDWHPEMAAQCANV
VVTDVTTNLLTVTIKEFCHPHGLPPSSSSPC

>Ame_Cbx7 (Gene ID: 103045301)
MELSAIGEQVFAVESIIKKRVRKGNVEYLLKWKGWPPKYSTWEPAEHILDPRLVLAYEEKEHRDRAVGWRKRGPKQKR
LLVQIEHVHKRKRRSSRDPSVEDWEGRDVDDEDEDEVYAREEETETDRATPNSNDGTEGWNSITPKACKEWRPVLGPE
DVTVTDVTINSLTVTFKEAVVAKGFFRSWELEF

>Ame_LOC103030095 (ENSAMXG00000013576)
MELSSLGERVFAVESITKKRVRKGNVEYLLKWQGWSPKYSTWEPEDNILDPRLVLAFEEKEERDRAQAHRRKGLRPRR
LILRNIYAMELRSAHKGPVKPPPRIRLSLARSVGAELEHSGRRFRAKVGGVFHHRQEKRKSRQCAFKRPNYAENPRQR
PPTHKKDSAEEEEEDEEEEEEEEEWEEKEREMRKRRKTEKDDEKTTDMRHDIASVKKMAVDHASSMEQEAVVMANQSD
HCVSSDESGDQPTKPFHSDSSSLVQRLTDQSAISTITDTHEQEPITDRSGGGAYVLTSDDETKSDQSLISSTESVSSL
KGAGSIDRVQRRASVIQLIDSCVQGQVRGAGQADGSVDEPERGTDKDEVTAECTTTLQLSAEVTTKADDDTLHPGKVI
VTDVTINSLTVTFKEAMTAEGFFSGYGLQV

>Ame_LOC103044980
MELSAVGERVFAAESIIKRRIRRGRMEYLVKWKGWSPKYSTWEPEENILDSRLFAAFEEREREREREFGPKKRGPKLKT
FLLKAQAKAKARSYEFRGESSRSIRVSYPSPEPLATPRAREGLRAVVPTIFPPSTVNRGESVRLPPPEPPRDHPLPRD
GFIPTPKKRGRKPKLRFKDGYPSSLHSDQAKMSAEESMSGIPSKMARLELGEDDEDSEDRCSDLRAIKLPHRNQASPA
NPYKPHRIVPSLDWGLHSSRMSVGLQDNRTREHLSHSSHLHRKHLKHLKHLSHHRAFEVLDPPKLPQQKPSLVAKIPV
ARILGEPEEEVEEDEEDDNEDVWNPCFSNVEKVVVTDVTTNFLTVTIKESSTDEGFFKDKR

>Ame_LOC103029671
MELSAVGERVFAAESIIKRRIRRGRMEYLVKWKGWSQRYSTWEPEENILDERLFAAFEEREREREREYGPKKRGPKPGT
YLSKAKAKASTKNYEFRREMSREIRVSFPMTEPVVTPRAREGLRTVVPTIFPPSTVNRGESVRLHPPEPERKARPGQD
HVDFLSAPKKRGPKPKLRFPVHVASSKSFNSRADEHFSLNSRSTVGERSHYGLMQLTQQHLSGLYGSEEPIDDDRLSP
EPLGVKEDGGESWSPCLRNMEKVIVTDITSNSVTVTIKESSTDKGFFREKR

>Pna_Cbx2 (Gene ID: 108433500)
MEELSAMGEQVFDAECILNKRLRKGKLEYLVKWRGWSAKHNSWEPQENLLDPRLLAAFNKREQERELLLQKRGKRPRG
RPRKIMETVPQVSKSSSSSSSSSSSSGSSSSSSSSSSSSSEDDDDDDDGKPKLSPRPRELHPVPQKKAQILVAKPDPPKK
KRGRKPLAPELKALRQSQAKSSRKPMPKDSLSDLRGSIKKPLMPASFTYTGMNRAANREPMAMQSRGSFNHNASSKSS
LGSLGPGRPVGSLGRPSQGKSTADFKLSDMASGGGGLDLKASACKSPGVASLSLHNPKLTTCNSNGQAAVGAPNWTKK
QESLAQSPLQRPANNKPATALSSAKNSNQTTSLQNATLHSVSKMAQGSDTSAVDGQGSIHRNSGSTVRKGLTQEKSLL
LNPTSQATGLGIVGTRNNSMPVGVERVKSEDCSDPAERFGKPNQGRPQKGLNFPPTLDRNGSKDGTKMAKTLSEMSTG
EEGTSSESEHESSLSSKRQDLSIGVQASQDWRPTRSLIEHVFVTDVTANLVTVTVKESPTSVGFFSIRNY

>Pna_Cbx4 (Gene ID: 108424718)
MELPAAGEHVFAVESIEKKRIRKGRFEYLVKWRGWSPKYNTWEPEENILDPRLLIAFQNRERQEQLMGYRKRGPKPKQ
LLTQVSSFARRSSVLSGLQEASQDNSLKAVVDPAQTQQYQLNSRKLHQYQPICKESSGDALVNGKKKHYYQLNSKKHH
HYQPDPKMYQDVKGTEVDKESWNLPKALPQKWLQDKDVVCLNKVKDITIGLEKLPANLHGGVKSELAKEDVNQANGVS
NRLKIVKNKNKNGRIVIVMSKYMENGTQAAKIKNGEMDVGARQQELLENQSDVTDKNKHSKKHSLENGWKKDNKDDGT
PPPCGFTNGISGPAVDGLVTSSTNKAKELHSSSPDQSSQLTTKSCMNSKLLQKAVLHHLDQKTSQGTHKRRYSEPNSE
RDSEAKRFLSFRSISAPNAETSHCQSNGHQNSNGHRFEFSDCQDEPMDLSCSRSRKENSSITNCQINGNSKTSEKEVA
PQQERTQKDPESEHAPSFTPFLGNIIITDVTANCLTVTFKEYVTV

>Pna_LOC108432204
MDLPAVGEHVFAVESIEKKRVRKGRIEYLVKWRGWSPKYNTWEPEENILDPRLLVAFQNRERQEQQMGYRKRGPKPKH
LLIQLPAFARRSSILAGLQETSLDDENQLKMDSLQMHRSQPQQYQLNSKKHHQYQPNSKEIPAEPHINGKKKHFYQLN
SKKHHHYQPDPKMYDQQPTKPKEVKGHDPSIKGWALPPALQQKWARDKDTGCLNKVKDLALEHKQVPVTANKEEQTLK
ISSKEAAVSSGISSKMKIIRNKNKNGRIVIVMSKYMDNGVQASKVKNLDSSGMERPHQTEESINGSAGNVLTVTSGQE
NGVSSTSANSSYVHKVPHNRFELSKAKTNTEVEQKTEQKTDTIDLCSDEPLQLTSKPSSSPKSIVTNAPSQKDSTSYQ

PNHRKRNLSEPNEGGSDCKKLLSSRSISAPSAVFSSPQREPIDLHFSGKSTRRTYSYDFTDAIPEEPIDLSCGKTRLQ
AEPPANPHSTVEDTPETSEKSEEPISYFKPFAGNIIITDVTTNCLTVTFKEYVSI

>Pna_LOC108410419
MELPAAGQHIYAVEHIEKKRIRKGRIEYLVKWRDWSPKYNTWEPEENILDPHLLVDFQKRERQEQVMGYRKRGPKPKH
LLMQAQSFARRSSTLSGLEEVKSGCLKTAVQTQRSSKTHHPYERICKKSLVEPLADGKPQLNSKKHQPNRKIYQKVKE
TEAAKDGWSIPQALQQKWVQNVPAKLKDITIELEELPTNVRCGGKSELGSSLIAKEAHASRISTKLKIVQNKNINGRI
VIVMSKYMENATQDPKMIHSEADLGVKQLLDNESKVAEKLRHSKNPNFENGCEKDSKDKAKPPPSGLTNGPFHPVAND
YITSSLLAAETDKPEELSSSTFNGHFKSSLTAKPSEKDVIAYSQQRSPHGAHKRQYSEPDNERDTEANSAPNSGPSQF
QSNIIGHQHSNRHRLELQDKPMDLSCSQLRKEGKSITDSQVKWNSEKEEGPQPEWTEKQPECEPFPTFMPFFGNIIIT
DVTAHCLTVTFKEYI

>Pna_Cbx6 (Gene ID: 108436630)
MELSAMGDRVFAAEAILKRRVRKGSIEYLVKWKGWALKHSTWEPEENILDGRLITAFEQKEREQELYGPKKRGPKPKN
FLLKARAQTGETSSRGSSARHTPPHSSSSAAVTPNPPSSSSSASLAPTPRLHSLAASHKLKKDIHRCHRMSRRPLPRP
DPDGSSFSSRLPISPFSETVRILNRRVKPREVKRGRIILNLKVIDKYGGGATNRRPAQTQQGRAQLPSRNRIIGKKQG
DMPYRPFQPPMKMLGFPMYGQPFGLQPCLPVSVQTSKRAGSTPVAKGRGPGRPSKPKFPFQSPPSPSSSSGSESDAPS
PPQIQPGPPQAPPETAPSSPPVLSPETPSRCSGVPQSRSSGAKPGSASSPTSFLPSSPSLSSSSSSSPEDEDERAQKL
PAPPKGGRRKPCRRFRASEGANSRPTAEKKALKRGNPDWRPERAPSCANVVVTDVATNQLTVTIKEFCKPGASSTDST
PPSPSQAKSPSFSTLSGT

>Pna_LOC108413245
MELSAAGDRVFAAEAILKRRVRKGRMEYLVKWKGWAIKYSTWEPEENILDERLVAAFEQKEREQELYGPKKRGPKPKT
LLLKSRAQASDGTPRVPEFKHTRPQPSSKPPPPPLPTPSYPPSCPSNAKLQSGAAQPKLKKDIHRCHRMSRRPLPRPD
PLAQSVGHSLSPFSETVRILNRKVKPREVKKGRVILNLKVIDKAGNSGVANNRRTPSTAHQSYIGRQKVPSRNRVIGK
SRRFGDVSYRGLPLPVSGAGFSVFGKAFEAHSMDPSGNQPKLEHSGHKPGKSLSLQSLSQSSKLMTSDVPKALSEAPP
SASSSEVSDGESHLTSSAQSHQPSLAHQSSKVNPAILPPLKPSNQLTDLKPNSSAAKSVLPSSPMFSSSSSSSSSSED
NEHILDLSVPHGSDRRAKRHHHFCRHQQPKLPEIPMSEEASEEEEELDWHPEMVAQCANVVVTDVTTNLLTVTIKEFC
HPHGLPPSASSPCYANHFSASNKTKPQP

>Pna_Cbx7 (Gene ID: 108413508)
MELSAIGEQVFAVESIIKKRVRKGHVEYLLKWKGWPPKYSTWEPEEHILDPRLVLAYEEKEHRDKAVGWRKRGPKPKR
LFVQIDHMDQKFQAKGGTIYRQLVQHKRKKRSSRDPSVEDWGGREEEEEDEEEDESTREEELETDRATPNSSCFSFSG
NVATEGWSSIIGSKEVTVSPMHEEWRPVMGPEEVTVTDVTINSLTVTFREALVAKGFFRSWELEF

>Pna_LOC108441212
MELSSLGEQVFAVESITKKRVRKGNVEYLLKWQGWPPKYSTWEPEENILDPQLVLAFEEKEEKDRAQAHRRKGLRPRR
LILRNIYTMELRSAHKLPEKPTPRIRLSLTRAVGAELDQNGRRYRTREGGVYHHRHEKRKSRHYSSKPPVIAENPRQR
PLTHQKDIMEEEEWEDEEKEGKEQEKRKKKKTEKDDEKTTYSRHDIPSAQEMTGDHIPSAKQKAVVMANQSEHCALSC
ERGDQLTKHVHTETISVQRLTDQSAVSTITETHEPIKDRSGGVASAPVSHNETKIDQSLKSSSELVYSAKEVGLIDRV
QKRASVICLIDSCMQDQVKEAGQTDVSADSTEKETDEDEVTAECTTTLQLSAEVRTDQSQGPGSTEHPGKVIVTDVTI
NCVTVTFKEAMTAEGFFSGYGLQV

>Pna_Cbx8 (Gene ID: 108424812)
MELSAVGERVFAAESIIKRRIRRGRMEYLVKWKGWSPKYSTWEPEENILDSRLFAAFEERERERELFGPKKRGPKLKT
FLMKAQAKAKARSYEFRGESSRSIRITYPSTDALAAPRAREGLRAVVPTIFPPGTVNRGESIRLPPPEPPRELSPPRP
TSDGFIYTPKKRGRKPKLRFSDGYSSGLLHPDKAKMSTEESMSGIPSKMARLELGEDDEDSEDRCSDLRAMKMSHRHQ
ASPVYPYKPHRMIPSMDWSLHSSRMAANFQDCRTRDHLSHLHRKHLKHLKHLSHHRAFETVDQAARLQQQKPTLIAKI
PVARILGEPEEEEEEDEEDEDTWSPCFSSVEKVIVTDVTTNFLTVTIKESSTDEGFFKDKR

>Pna_LOC108432169
MELSAVGERVFAAESIIKRRIRRGRMEYLVKWKGWSQKYSTWEPEENILDERLFAAFEERERERELYGPKKRGPKPET
FLLKAKAKASAKNYEFRREMSREIRVSFPVAEPVVTPRAREGLRTVVPTIFPPSTVNRGESVRVRPPEPERKARPVQG
PVDFFNVPKKRGPKPKLRFPVSAGGCASHESFHRRADERFSLSHAKMGETSAYGLMQLTQQFRSGDVHRHLSRNSLYR
SSDPADDDDALSPEPLDVEEEAGESWSPCLRNMEKVIVTDITSNSLTVTIKESSTDKGFFREKR

>Mar_LOC113135962
MEGVTVGQVFDAECILSKRPRKGKFEYLVKWRGWSSKHNSWEPEENILDPRLLTAFHKREQERELLFQKKGKRPRGRP
RKILPAPAATKDSRSSSSSSSGLSSSASSSSEDEDHSKKAKTGPRVHPVPQKRPQIMLAKPDPPHKKKRGRKPLHPDL
RALRQAKSRASPPRHHQALRLPRDEHRPGVKKPLQPASFTYTGLSRTSREEAASASHTSSSSFSQTTTSKPGSLSCMW
TSRSLSSSSPSSGSSSHNKGSPSLQSKNSLSELKRSISESGSSRGNGFKVSPLKQGGGSGSLGLHGSFGGGQMAVQRS
PLGQRRQEGVAGQTGVVQHKQQNSGLSKPSSSSSPTSRDRANQALSLRALNLQSVSKPLTGSSLQGNTGSAVAAASRA
SLRSGIVVKGVGGIKDTRTSAGGQRSGLTAGGATEQARLREGKDMSGGGGAGRGNSKLRQEERKHGLSSQNRNLNELS
TGDSDETSSSESEHDIPLFPRDSRPSLGNNATESDTETDWRPTRNLLEHVFVTDVTANFITVTVKESPTSVGFFNSRN
H

>Mar_LOC113141452

MEELSAVGEQVFDAECILNKRLRKGKLEFLVKWRGWSSKHNSWEPQENILDPRLLAAFNKKEQEKELLMRKRGKRPRG
RPRKILENVPEVPKSSSSSSGSSSSTDSSSSGSSSSSSSDDDDDDDSSVKPSNPSVRTRDLDPVPQKKAQIVMAKQE
PPKKRSKKPLPPEVKEFQQNKGPRKTPKAARDIDLPGAIKKPVHPASFTFMGFHRGSARDTVGGHNRSFLSQGAAVKN
SMSSVGSGRTVPPASFSLNKSSQSRSVAEGKVSISSVNSGTSLDLKTAASKSKGVAALNLNTSKHPIQGTTQHTLSSP
NGQKKPPPPVSTPQRIPNAKTMASMLSKNTSSTHGSGLQPLNLQNKPLQNNDGPGSGTTPVSGLKNATNPARKATVTQ
NQEYNSLKSPATPGKPQVRKNQSGSDKAKEANEIQTSRVQGSMHKSTTEVPSQQERSVSRDSKKARMNDMSTGEEESS
SDSDQDSSYTRQDCAVAVQNQDWKPTRSLIEHVFVTDVTANLVTVTVKESPTSVGFFSIRNY

>Mar_Cbx4 (Gene ID: 113128646)
MELPAAGEHVFAVESIEKKRSRKGRVEYLVKWRGWSPKYNTWEPEENILDPRLLVAFQDRERQEQLMGYRKRGPKPKH
LLVQVPSFARRSSILADIHEESPDEESCQKTSPIQMFRPQAQQYQLNSKKHHQYQPHCMEREAEQQTNGKKFYYQLNS
KKHHHYQPDLKVHEPIFAKPKEVKAQELSNKGYNLPPVLQQKWIRDKDSGCLTKVKDITMELKKLPADLNGNKEPEKV
KPKEDASPQSNGVSNGKLKIVKNKNKNGRIVIVMSKYMENGMQAAKIKNADSETTEKPQQGNGNSMENHLEKMKLVKQ
LGLMNGFSKIPKDNPTVPSSGFNGDCPKEKEMSPEMEPTVTEQDKHVTVRSQEQLPADQPLQLTTKTNLLSLPLDRGV
LSSRDKRGNQGGFQGLKRHLSDTDREEHESTKRFLSSRSISSPDTVSSPTQSISIDQNGQKNHTGLQDCGYADQEEPI
DLRIIKSRPKAAASTETQPKTHTQETTHTQDQILTQDETRTHAETDTQAQTETPKTAEEEKVESLPKDEKTKEAAFPS
FQPFLGNIIITDITTNCVTVTFKEYVMV

>Mar_LOC113141280
MELPAAGEHVFAVEGIEKKRIRKGKIEYLVKWRGWSPKYNTWEPEENILDPRLLVAFQHRERQEQQMGYRKRGPKPKH
LLLQVPSFARRSSIPAGFEETSQDGEGILKSEPVQVQRSQPQQYQLNSKKHHQYQPSSQEVPADQLISSKKKFIYQLN
SKKHHHYEPDPSMYDPQASRLKEVVKVQETASKPANPGWNLPLALQQKWVRDKDTGCLSKVKELAVEVRKPAVKEAES
EHALKPNPKDATLPSAISSKMKIIKNKNKNGRIVIVMSKYMDSNKVHGAKGKHGESSREEKPQNIKPPENNPPHRIKM
VEHPENGIPKEICNGSSLPTAEHPIKCSPKDRHFSKPSPSTAEEYNTEVARGQADLPDDLPLQLTASSPPASWAVDTN
IPTPTAVDQIRIPSFPNDRKRKLSDPVESRNVSKAYLTSRSFSVPSTVVTPPQDKPMDLHCSDRRLSSTCTYEVMDSG
SQEEPMDLSCPKTKKLAEPETQSEMEPEPGPAVKDTPPVAEDTQQSTEKSKEAPAKKISPFMGNIIITDITTNSLTVT
FKEYVSF

>Mar_Cbx6 (Gene ID: 113127975)
MELSAVGDRVFAAEAILKRRVRKGRIEYLVKWKGWAMKHSTWEPEENILDDRLILGFEQKERERELHGPKKRGPKPKN
FVMKARAQKGTSSRASNSRQNTQRSSSSTSSRATPSSSASPHQLPSLSSASSSVAPSPKLNSLAATHKLKKDIHRCHR
MSRRPLPRSDPMAPTFSNPGGFPSRMHVSPFSETVRILNRRVKPREVKRGRIILNLKVIDKPGRGGTATGSRNITAGR
QNIPSRNRIIGKKGEAPYRPFQPPLKMLGFPMYGKPFGLQCGGPVPFHSHQGSCSSTGAKDSHTTPSQYQSPPSPSSS
SGSEGKSPTTTSAAQSQPTNGTTPSSEGPKTSKPHTETQKGQGTKPSAPAPSSDANPMASVSPFLPSSPSSSLEDDED
EDALDRMVPTEGGRKNLRQRRAKRQVPTKLPSVTPGDQSSAPTEPKRVPAEGDPDWHPEMSPSCKDVVVTDVTTNLVT
VTIKEFPSPASPSASPENASSPTPATTSDDPSPAKP

>Mar_LOC113135651
MELSAIGEQVFAVESIVKKRVRKGNVEYLLKWKGWPPKYSTWEPEEHILDRRLVQAYEEKELRDRALGHRKKGSKAKR
LLLQNTVYTMDLRSAHKTPEEPPPRLRLSLTRSLLPEDEDVPYSPCVQDPRPQMAHHKSKPRRPQFRCLDSNPPSPNQ
DDWEGQGEEEAEEEEDNVEDDEEVMEGKSGGVLDGPERTDEWSSATEPHEVVESEKLDIVWRPIIGPGEVTVTDVTLN
SLTVTFRESRVAKGFFRDWGLEV

>Mar_LOC113141587
MELSSIGDQVFAVESITKKRVRKGNVEYLLKWQGWPPKYSTWEPEDNILDPRLVLAYEENQEKLRALAYRRKGLRPRR
LVLRNIFAMDLRSAHKVPEKPPSRLRLSLTRSMSTDVDQGERGSLYRRPGWRKSKQRVNKRRLDRSSSKPIRSLRKKE
EPVEEDWSGTSEEEKQESESTTEEKHEDSLYGQSECSSPPLLERQDLEMEVEEKAAEEKAAEEKAAEEKVAEEKAAEI
WSDRPGGETPETRLNQTSECNQSEDSASAPVAGPGDAVTVGDRSDVGVEAGSPCPRLDRNNTTSVIVCVQGSNQSVAT
TTPGSETSAEEHPGKVIVTDVTINSLTVTFKEAMVAEGFFKGY

>Mar_LOC113141197
MELSAVGESVFAAESIIKRRIRRGRWEYLVKWKGWSQKYSTWEPEENILDARLFTAFEERERERELFGPKKRGPKPET
FLLKAKAKAKEKIYEFRREAPRGIQVSYPIPEPVITPRAREGLRTVVPTIFPPSAVNRGESVQVRPPEPERRPRPTAP
AALIPQEFVRFPKKRGRKPKLHLHYDRDDGNSSLEPDCKRSRLLEEPMSHKMSRRLHHHGETSDHSLIQLTRRFQEET
TITPKTCSEQRHVGGAGLSYSCAFSSDVHKSDPGGHRTNCLNRMSIPQSSKLKHSAQHRNQQMKKCCLPRKQSAAIST
ESESRPDSSERPAWTPSFTNLDTVTVTDVTMNFLTVTIRESSTDKGFFRDKR

>Mar_LOC113128878
MELSAVGERVYAAESIIKRRIRKGRIEYLVKWKGWSPKYSTWEPEENILDSRLFAAFEQRERERELYGPKKRGPKPKT
FLLKAQGKVKGKAYEFRSEAVRGMHITYPTPEPVVTPRAREGLRAVVPTIFPPSTVNRGESVRIQPLQFSTRERQQPP
LQQSSPDRLIHIPKKRGPKPKPRFSSAVPEPHKRRAEEQVSHSPHKLAKLQGDKDMRLVEVGHRHPDSHSHKHHHHHH
HHHHHHTHSRGQLYSDRSLHPHRTDMDIHRIKDGSSYLAPAQFKHQSKMSQSLNPPTELPQMEKPYFLDRPSPTRLDK
DLDEVTWRPSLGNVEKVLVTDVTANFLTVTIKESSTSKGFFREKR

>Mal_LOC109962840
MEELSAVGEQVFDAECILNKRLRKGKLEFLVKWRGWSSKHNSWEPQENILDPRLLAAFNKKEQEKELLMLKRGKRPRG
RPRKILENVPEAPQSSSSSSGSSSSSDSSSSCSSSSSSTDDDDDDSINRANQSVRTRDLHPVPQKKAQIVMAKQDPPK
KRSRKPLPPEVKEFQQNKGPRKILKAAKDIDLPGVIKKPVHPASFAFMGFHRGSARDTVGGQNRGIVTQGGAIKSSLS

SMGSGRSAQPASFSLNKSSQSRNFFSMNSGTSLDLKAAASKSKGVAAMNLNTSRHPIQGTTAHTQSSSSSGQKKPQGP
VSALQQMPHTKAVSSLASKTASSNHGSNLQPLNLQNKLAESNDAGNGTATVSGLRNAASPARKTTVTQNQEYNSPNSP
VTPGKSQVRKSQSGADKVKEAAEIQTSSVQARLEKSSVHTTEARSQQERSASKDGKKAKMNDISTGEEDSSSDSDQDS
SYNRQNCSVSVQNQDWKPTRSLIEHVFVTDVTANLVTVTVKESPTSVGFFSIRNY

>Mal_LOC109969680
MEGVTVGQVFDAECILSKRARKGKFEYLVKWRGWSSKHNSWEPEENILDPRLLAAFHKREQERELLFQKKGKRPRGRP
RKIQPAPTTTKDSRSSSSSSSGLSSSASSSSEDEDHTKKPKPGPRVHPVPQKRPQILLAKPDPPHKKKHGRKPLHPDL
RALRQVKSRPSPPPRHHQALRPPRDEPRPGVKKPLQPASFTYTGLSRTSRDEAASSQTSTSSFSQTASSKPGSLSCIW
TNRSLSTSSPSSGSSFSHSKANPSPQSKNSVSELKRSISESGSSRGNGFKVSPPKPGGGSGSLSLHSSFRGGQMAVQR
SPLGQRRQDGVGGQTGLVQHKQQNSGLSKSLSSSSPTPRDRASQALSLRALNLQSVSKPLASSSLLGNNPSSSVAAAS
RSSLRNSSSIVVKGGMASIKETRTAPGGQRSGLATGGTTEQGRSREDRGKEMLAGGSSGRGNGNGRQDERKHGLSCQN
RSLNELSTGDSDETSSSESEHDAALYPNNSRPSFGNNATESDTETDWRPTRNLLEHVFVTDVTANFITVTVKESPTSV
GFFNSRNH

>Mal_Cbx4 (Gene ID: 109957911)
MELPAAGEHVFAVESIEKKRSRKGRVEYLVKWRGWSPKYNTWEPEENILDPRLLVAFQDRERQEQLMGYRKRGPKPRH
LLVQVPSFARRSSILADLHEESLDEESCQKTNPIQMLRPQAQQYQLNSKKHHQYQPLCREREAEQQTNGKKFYYQLNS
KKHHHYQPNLKVPEPVFAKPREVKAPELSSKGYNLPPVLQQKWVRDKDSGCLTKVKDITMELKKLPADLNGHKEPEKV
KPKEDASPQSNGVSSSKLKIVKNKNKNGRIVIVMSKYMENGMQAAKIKSGDSETAEKPQQGGGNSVENHLEKMKLVKK
LGLMNGFAKIPKDQPAVLNSGLNGDCPREMEQSPKMELTAREQDKHFEVRGQRQLPADQPLQLTTKPNVLSSPSDREG
PSALDKRGNQGGVQGLKRHHSDTDSKDHGTNKKFLSCRNIIAPNPVSSPAQSTSIDQNRHQRHTGLQDCGYVDQEEPI
DLSIVKSKPKAATSTASQPETHIQAETHTQAPILAQPETHTQNEAQLDTQTETQRPTETSKPAEGNTESSNNEKKREG
TFPSFQPFLGNIIITDITTNCLTVTFKEYVTV

>Mal_LOC109969469
MELPAAGEHVFAVEGIEKKRIRKGKIEYLVKWRGWSPKYNTWEPEENILDPRLLLAFQHRERQEQLMGYRKRGPKPKH
LLLQVPSFARRSSIPASLEEISQDAEGSLKSDPIQVQRSQPQQYQLNSKKHHQYQPSNQEVPADQLINGKKKFIYQLN
SKKHHHYEPDPNMYDPQASRLKEVVKVQEPASKPANPGWNLPLALQQKWIRDKDTGCLSKVKELAVEVRKPAVKEAES
EHALKPNPKDASLPSAISSKMKIIKNKNKNGRIVIVMSKYMDSNKVHGAKGKHGESSREEKPQNTKPSENNPAHRTKM
VEHPENGIPKEICNGSPLPAAEHPIKCSPKDRHFSKPSPSTAEEYNTEVARGQADLPDDLPLQLTASSPPASWAVDNN
ILTPTALDQIRIPSFPSDRKRKLSNPVENMNVSKVFLTSRSISVPGTVVMPPQDKPMDLHCSSRRHSSASTHEVMDSG
SQEEPMDLSCPKTKKLVEPEVQPELEAKPEPAVKDTPPVSEDTQKSAEKSKEAPVKKISPFMGNIIITDITTNSLTVT
FKEYVSF

>Mal_Cbx6 (Gene ID: 109951608)
MENSAGGDRIFAAEAILKRRVRKGRIEYLVKWKGWAMKHSTWEPEENILDDRLILGFEQKERERELHGPKKRGPKPKN
FVMKARSQKGETSSRASNSRQNTPRSSSSTSSRAAPSSSASPHHLPSLSSSSSTVAPSPKLNSLAATHKLKKDIHRCH
RMSRRPLPRSDPMAPTFSNPSGFPSRMHVSPFSETVRILNRRVKPREVKRGRIILNLKVIDKPGRGGTAASSRNITAG
RQNIPSRNRIIGKKGEAPYRPFQPPLKMLGFPMYGKPFGLQCGGPVPFHSQPGSCSSTGAKDSHTTPSQYQSPPSPSS
SSGSEGKSPSTTSAAKSQPTNRTSPSSEGPKPSKPHTETQKGPSAKPSAPAPSSDADPVASASPFLPSSPSSCLEDEE
EEGAVDSSAPTEGSRKSLRQRRAKRQVPTTLPSVTPGDQRPSPTEPKRVPAEGDPDWHPEMAPSCKDVVVTDVTTNLV
TVT

>Mal_LOC109952690
MELSAIGEQVFAVESIVKKRVRKGNVEYLLKWKGWPPKYSTWEPEEHILDRRLVQAYEEKEQRDRALGHRRKGSKAKR
LLLQNTVYTMDLRSAHKPPEKPPPHLRLSMTRSLVPEDEDEDEDEPYSSCRQGPQPQMTHLRSKSRRSQFRCFDSNPP
SPGQEDWEGRGEEEEEEVEDNGDEEEEMEVAQKDTAEKSVGIPNGQKRTDKWSSTIIPDEVTASEKPDNMWRPIFSPG
EVTVTDITLNSLTVTFRESRVAKGFFRGWGLEV

>Mal_LOC109952654
MELSSIGDQVFAVESITKKRVRKGNVEYLLKWQGWPPKYSTWEPEDNILDPRLVLAYEENQEKIRALAYRKKGLRPRR
LVLRNIFAMDLRSAHKVAEKPSPRLRLSLTRAMSTDVDQGERGSMYRRPVRRKSKQRVAKQRSDRCLNKPIHTLIKKE
EPMEDDWSGTSEEEKQESESTEERHEDSLYGQSECSSPPLLERQDLEMEVEEKVDANLTAAGTETWTEGLGADTRQNQ
EFACNQSENSASVPVAGPGDAVILHNKSDGDTGEEGVKSGSECPKSERNNTTSVIVRVNRSSETVGTTAATVLSTAEA
RSDNQSFTTTAPGSQTIAAEDPGKVIVTEVTLNSLTVTFKEATVAEGFFKDY

>Mal_Cbx8 (Gene ID: 109958030)
MELSAVGERVFAAESIIKRRIRKGRIEYLVKWKGWSPKYSTWEPEENILDSRLFAAFEQREREREL YGPKKRGPKPKT
FLLRAQAKVKEKAYEFRSEAVRGMHITYPAPEPVVTPRAREGLRAVVPTIFPPSTVNRGESIHVRPLELGTREHQQPS
LQQSSPEGLIHIPKKRGPKPKPRFSPAVSEAHKRRAEEQVSHGPHKLAKLQGGEDMRLVEVAHRHPENHSHKHHHHHH
THNRELSSGSSYKQLYSDRSLHLHRTCTDIQRTKDGSTYLAPAHFTHQSKMSQSLNHPAALPQMEKPYFLDRPSPTRL
DDNLDEVTWRPSLGNVEKVLVTDVTTNFVTVTIKESSTSKGFFKDKR

Mal_LOC109969470
MELSAVGESVFAAESIIKRRIRRGRWEYLVKWKGWSQKYSTWEPEENILDARLFAAFEERERERELFGPKKRGPKPET
FLLKAKAKEKSYEFRREASRGIQVSYPIPEPVITPRAREGLRTVVPTIFPPSAVNRGESVQVRPPEPERRPRPTPPAA
LTLQESTRFPRKRGRKPKLHLHYDKADGSSSAEPDTKRSRLLEYPMSHGLSKMSRRLHHHGETSDHSLLQLTKRFQEK

TSITPKSCGEHRHVGGVGLSYTCAFSPGVRKRDQEGRRTNCLSRMFVPQPSKPKHSAEHRSQQMTRCSLPREPPHVLS
AQSEFIHDSSETPASSWKPTFTNLDTVTVTDVTMNFLTVTIRESSTDKGFFRDKR

>Ate_LOC113156830
MEGVTVGQVFDAECILSKRPRKGKFEYLVKWRGWSSKHNSWEPEENILDPRLLAAFHKREQERELLFQKKGKRPRGRP
RKILPPVPTATKDSHSSSSGLSSSASSSSSEDEEHTKKAKPGPRVHPVPQKRPQIMLAKPDHPRKKKRGRKPLHPDLR
AKSRPPLPPPPQRHPQPLRTPRDEPRPGVKKPLQPASFTYTGLSRASRDEAASASQTSTSSFSQTAASKPGSLSCVWM
NRSLSTSSPSSGSSSHIKTSPSTQSKNSLSELKRSISESGSSRGNGFKVSPLKQGGSSGSLGLHSSFSGGQTSVHRSP
LAQRRQEGVGGHIGLVQHKQQNSGLSKPSSSSSSPTPRDQANQALSLRALNLQSVSKPSAGSSLQGNNTGSAVARSSL
RSGSGIVIKGGVGGISRTSAGGQRTGVAAGNAAEQGRLRDDRGKEMLAGSSSGRGNGSGRQEERKHGLSSQNRSLNEL
STGDSDETSSSESEPDASLYPHNSRPSLGNNATESDTETDWRPARSLLEHVFVTDVTANFITVTVKESPTSVGFFNSR
NH

>Ate_LOC113158311
MEELSAVGEQVFDAECILNKRLRKGKLEFLVKWRGWSSKHNSWEPQENILDPRLLAAFNKKEQEKELLMRKRGKRPRG
RPRKILDNVPEVSKSSSSSSGSSSSSDSDSTSSCSSSSSSSDDDDDDSNIKQANPSVRTRDLHPVPQKKAQIVMAKQE
PPKKRSKKPLPPEVKELQQNKGLRKVLKTSKETDLPGAIKKPVHPASFTFMGFHRSSAKDTVGGQNRSFLSQGGAVGS
SRSVQPASLGLNKPSQNRNVTEGKISVSNTSSGASLDLKAAASKSKGVAALNLNTSKHHIQGTTQHTLSSPNGQKKPQ
APVSTLQRIPSTKALASLPSKNTSSNHGLGLQPLNLQNKSVQSSNTSGSGTTPLSSGAKKTAVTQNQEYNPKSPTIPG
KPQIRKNQAGADKMKEATEIQTSRVQDRLEKSRVHKSTDIQSQQERLAPKDGSKKSKMNDMSTGEEESSSDSDQDFSY
NEQDASVAVQNQDWKPTRSLIEHVFVTDVTANLVTVTVKESPTSVGFFSIRNY

>Ate_Cbx4 (Gene ID: 113162586)
MELPAAGEHVFAVESIEKKRSRKGRVEYLVKWRGWSPKYNTWEPEENILDPRLLDAFQDRERQEQLMGYRKRGPKPKH
LLVQVPSFARRSSILADLHEASLDQDSCQKTNPIQMLRPQTQQYQLNSKKHHQYQPLCREREAEQQGNGKKFYYQLNS
KKHHHYQPDLKVHEPLFAKPREVKATELSNKGYNLPPVLQQKWVRDKESGCLTKVKDITMELKKLPADLNGHKEPEKV
NPKEDASPQSNGVSSSKLKIVKNKNKNGRIVIVMSKYMDNGMQAAKVKNGDSDTAEKVQQGAENSTENHLEKMKLVKK
LGLMNGFAKNYPRDKPATPSSGLNGDCSKEQERSPRMEPTVTEQNKHVEVRGQGQLAAAQPLQLTTKPNLLSLPLDRG
VSSAADKRGNQSGFQGLKRHLTDTEEHGSSKRFLSCTSVANSISSATQSISNDQNGHQSRAPLQDCGYADQEEPIDLS
IVKSRAKPGASAAMEPETSTQTATHGQAQILTQAETHTQSKTQTDAQTETETQAQTETHESAEEEKVDSVGSVSNSEK
KKEETFPSFQPFLGNIIITDITANCLTVTFKEYVTA

>Ate_LOC113155158
MELPAAGEHVFAVEGIEKKRIRKGKIEYLVKWRGWSPKYNTWEPEENILDPRLLVAFQHRERQEQLMGYRKRGPKPKH
LLLQVPSFARRSSIPAGFEETRQDTEGCLKSDPIQVQRSQPQQYQLNSKKHHQYQPNSQEVPADPLTNGKKKFIYQLN
SKKHHHYEPDLSMYDAQASRLKEVVKVQEPACKTANPGWNLPLALQQKWVRDKDTGCLSKVKESALEVRKPANKDTES
EQALKPSPKDVTLSGTVSSKMKIIKNKNKNGRIVIVMSKYMDSNKVHGTKGKHGESNEEKLQSIQASENNPAHRTKMV
EHPENGIPKEMCNGSSLPAAEHPIKCSPKDRHFSKPSPSTAEEYNTEVARGQADLPDDLPLQLTASSPPTSWAVDTSI
PTPTAVDQIRIPSFPSDRKRKLSDPVEDRKVSKTLLSSRSFSVPSTVATPPQDKPMDLHCSGRHLSSTCTYEIMDSGS
QEEPMDLSCPKTKKQVEPEIQPELEREPEPAIKDTPPLPMTENTQKSTEKSTEAPVNKISPFMGNIIITDITTNSLTV
TFKEYVSF

>Ate_cbx6a (ENSATEG00000003501)
MEQSAAGDRIFAAEAILKRRVRKGRIEYLVKWKGWAMKHSTWEPEENILDDRLILGFEQKERQRELHGPKKRGPKPKN
FVIKARAQRGETSSRASNTRQNAPRASPSTSSRAAPSPSASPHQLPSMSSSTVAPSPKLNSLAATHKLKKDIHRCHRM
SRRPLPRSDPMAPTFSNAGGFPSRMHVSPFSETVRILNRRVKPREVKRGRIILNLKVIDKPGRGGTAAGSRNITAGRQ
NIPSRNRIIGKKGEAPYRPFQPPLKMLGFPMYGKPFGLQCRGPVPFHSHPGSCSSTGTRNSLTTSSQYQSPPSPSSSS
GSEGKSPTATSAAQSQPTTGATPSSEAPKSSKPHTDTQKGQSTKQSAPAPSTDANPTALASPFLPSSPSSSLEDEDEE
GAAERLAPTEGGRKNSRQRRAKRQVPATLPLVAAGVQISATTEPQRVPAEGDPDWHPEMAPSCKDVVVTDVTTNLVTV
TIKEFPSPASCPGSPSASPENASSPPPATTSDDPSPAKP

>Ate_Cbx7 (Gene ID: 113155821)
MELSAIGEQVFAVESIVKKRVRKGNVEYLLKWKGWPPKYSTWEPEEHILDRRLVQAYEEKEERVRALGHRRKGPKSKR
LLLQNTVYTMDLRSAHKTPEKPPPQLHLSLTRSLVPEDEDEAYSDCGQDAQPQMAYCKSKSRKSHFRCLDSNPPSPTE
EDWEGRGEEEEEDDVDVEEDMEEEEETVVAQKETTEKSVGIFNGQERTDKWSSAVGPDEIIASEMTDNVWRPIIGPGE
VTVTDITLNSLTVTFKESRVAKGFFRGWGLEV

>Ate_LOC113152693
MELSSIGDQVFAVESITKKRVRKGNVEYLLKWQGWPPKYSTWEPEDNILDPCLVLAYEENQEKIRALAYRRKGLRPRR
LVLRNVFAMDLRSAHKATEKPPPRLRLSLTRSMSTDVDQGERSMYRRPVQRRSRQRMMAKRGSLIKPIRPLRKKEEPL
EEDWSVTSEEEKQESESTTEERREDSVYGQSECSSPPLLERQDLEMEVEVEEKADTDLTAVGTEMWIDRPGGGTSETR
QNQTFACDQIKDSALARVAGPGDAVTVGDGSDLDRDDEGVKSGSECPRVERNNATSVIVRVQRSSEAAGDAAVDCSAV
EARSEEVKGDNQSETTTTAEHPGKVIVTDVTINSLTVTFKEAMMAEGFFKGY

>Ate_Cbx8 (Gene ID: 113162656)
MELSAVGERVFAAESIIKRRIRKGRIEYLVKWKGWSPKYSTWEPEENILDSRLFVAFEQKERERELYGPKKRGPKPKT
FLLKAQAKVKGKSYEFRSEAARGIHITYPTPEPIVTPRAREGLRAVVPTIFPPSTVNRGESVRVRSPEHSIRDHQQPS

LQQTSPDGLIHVPKKRGPKPRFSHAVSELHKRRAEEQVSHGPHKLAELHGDEEMGLVKAGHRHPENHSHIRKHHHQHS
HHHHHHSHNRGLSSGSSYKQLYSDRSLLPHRMDVDIHRTKDGSSYLAPTHFKHQPKLCQSLNHPAELPQMEKPYFLDR
PSPTRFDDNLDEVTWRPSLGNVEKILVTDVTTNFLTVTIRESSTAKGFFKDKR

>Ate_LOC113155165
MELSAVGESVFAAESIIKRRIRRGRWEYLVKWKGWSQKYSTWEPEENILDARLFAAFEERERERELFGPKKRGPKPET
FLLKAKAKEKTYEFRREAPRGIQVSYPIPEPVITPRAREGLRTVVPTIFPPSAVNRGESVHVRLPEPERRPGPTPPAA
LTLHESAQFPRKRGRKPKLHLHYDKDDGSSSAEPDTKRIRLLEEPVPHGLSKMPRRLHHHGEASDRSLIQLTKRFQEE
TTITPKSCSEQRHAHGSSSLSYTCAFNPDVRRSDQGVHRTHCLRRMSVPQHSKLRHSADHRSHQVKESSLPREQPAAI
CTQSESIDDTSALPASSWTPSFTNVDSVTVTDVTMNFLTVTIKENSTDKGFFRDKR

>Bsp_LOC114845447
MEGVTVGQVFDAECILSKRPRKGKFEYLVKWRGWSSKHNSWEPEENILDPRLLAAFHKREQERELLFQKKGKRPRGRP
RKILPVPTATKDSRSSSSGLSSSASSSSSEDEEHIKKAKPGPRVHPVPQKRPQIMLAKPDSPHKKKRGRKPLHPDLRA
LRQAKIRPPLPPPPPPRHAQPLRPTRDEPRAGVKKPLQPASFTYTGLSRSCRDEAASASQSSSSSFSQTAASKPGSLS
CIWTNRPLSVSSPSSGSSSHIKSNLPPQSKNSLSEQKRSHSESSGSRGNGFKVSPPKPGGSSSSLGLHSSFSGGQTSI
HRSPLGQRRMEGTGTHIGPAQNKQQSLTSSSLTARDQANQALSLRALNLQSVSKPSAGSSLQGHSTGSAAGRCSLRSG
GSLVAKGGTSGGARAALVAGGAADQGRLREDRGSGSSRGNGSGRQEERKHGLSSQNRSLNELSTGDSDESSSSSESEP
DASLFPHNSRPSYGINATESDTETDWRPARSLLEHVFVTDVTANFITVTVKESPTSVGFFNSRNH

>Bsp_LOC114861130
MEELSAVGEQVFDAECILNKRLRKGKLEFLVKWRGWSSKHNSWEPQENILDPRLLAAYNKKEQEKELLMRKRGKRPRG
RPRKILENVPEVSKSSSSSSGSSSSSDSSSSCSSSSSSDDDDDDDEESNEKQANPGVRNRDLHPVPQKKAQIVMAKQE
PPKKRSRKPLPLETKEFLQNKGPRKILKTSKDTDLPGAIKKPVHPASFTFMGFHRNSAKDAPGSQNRSLVSQGGAVAS
GRSVQPASLALNKSSQNRNATEGKLSISNTSSGTSLDLKAAASKSKGVASLNMNTSKNPIQGATQHTLSPPNGQKKPQ
APVSTLQRLASTKAPASVPPKNASSNHGLGLQPLNLQNKSVQSINTPGNGTTPLSSGAKKTPVTQNQEYNPKSPPVLG
KPQVKKNQTGTETIKEAAEIQTSRVQGRQEKNTVHKSNTEVQSQEERLAPKDGKKSKMNDMSTGEDESSSDSDQDSSC
AEQDGSVAIQNQDWKPTRSLIEHVFVTDVTANLVTVTVKESPTSVGFFSIRNY

>Bsp_Cbx4 (Gene ID: 114846950)
MELPAAGEHVFAVESIEKKRSRKGRFEYLVKWRGWSPKYNTWEPEENILDPRLLDAFQDRERQEQLMGYRKRGPKPKH
LLVQVPSFARRSSILADLHEASLEEDSRQKSSPVQMFHPQTQQYQLNSKKHHQYQPLCREREAEQPSNGKKFYYQLNS
KKHHHYQPDLKVQEPMFAKPREVHAPELANKGYNLPPVLQQKWVRDKESGCLTKVKDITMKTISAGLNGHKEQEKEKP
KEDDSPQSNGVSSSKLKIVKNKNKNGRIVIVMSKYMDKGMQAAKAKNGDSETEKAQQGVDHSTERHLEKMKLVKKLGL
MNGFAKNHPKDKPSVPSSGFNGDCPKEKQQSPKIQPAMMERDKHIEVRGQGQLAADQPLQLTTKPNLLSLPMGGGVSS
AVDKRGNQGGFQGLKRHLTDTEEHGSSKRLNCTSVNSVSSSQSLSDQSGLQDCGYADQEEPMDLSMIKARPKAGDYAE
TGAQAETHALACMDQPKTHAESDTKPQTEAQNSPEKVSSDAVSDSSSAKKEETFPSFQPFLGNIIITDITTNCLTVTF
KEYVTV

>Bsp_LOC114860475
MELPAAGEHVFAVEGIEKKRIRKGKIEYLVKWRGWSPKYNTWEPEENILDPRLLVAFQHRERQEQLMGYRKRGPKPKH
LFLQVPSFARRSSIPAGFEDAPQDAEGCLTSDPIHAPRPQPQQYQLNSKKHHQYQPSSQEIPADQMANGKKKFIYQLN
SKKHHHYEPDLSVYDAQASKLKEVVKVQELANKPANPGWNLPPALQQKWVRDKDTGCLSKVKESPVEARQPAVKETES
EHALKPSPKDSAPPSAISSKMKIIKNKNKNGRIVIVMSKYMDGHKVHGMKSKHGEPSTEEKPQSTKLSENNPVHRGLA
VDHPENGLPKEMCNGRSLPAAEHPIKCSPKDRHFSKPSPSTAEEYNTEVARGQADLPDDLPLQLTASSPPASWATDTS
IPTPTAMDQIRIPSFPSDRKRKLTDPVEDRAAAKVYLSSRSFSVPSTVVTPPQDKPMDLHCSSRHFSSACAYEAADCG
GQEEPMDLSCPKTKTQAETEAKPEQESEPEPPPQPAVKDPPPPPVTENTQKSTEKPKEAPVNKAPPFMGNIIITDITT
NSLTVTFKEYVSF

>Bsp_Cbx6 (Gene ID: 114870514)
MEQSAAGDRIFAAEAILKRRVRKARIEYLVKWKGWAMKHSTWEPEENILDDRLILGFEQKERDRELHGPKKRGPKPKN
LVFKARGQKGEASSKTSNNRQNTPRSSSSTSTSGRAAASAPLPPSSSSSAAPSPKLNSLAATHKLKKDIHRCHRMSRR
PLPRSDPMAPTFTTPGGFPSRMHVSPFSETVRILNRRVKPREVKRGRIILNLKVIDKSSRGGTASSNRNITAGRQNIP
SRNRIIGKKGEVPYRPFQPPLKMLGFPMYGKPFGLQCRGPVPFNSHPGSCSSTGTRDSHTTPSQYQSPPSPSSSSGSE
GKSTSTSAALSQTTTGPSPNEAPKSSKLHTESQKGQSAKPSTPSPSKDTSPTGVASPFLPASPSSSLEDEEEKHLVPT
EGGRKNLPQRRAKPQQPAALPPATPGDSSSATTEVLRVPAEGDPDWHPEMSPSCKDVVVTDVTTNLVTVTIKEFPSPA
SPSGSPENASSPAPATTSDDTSPAKP

>Bsp_LOC114846206
MELSAIGEQVFAVESVVKKRVRKGNVEYLLKWKGWPPKYSTWEPEEHILDRRLVQAYEEKEQRVRALGHRRKGSKTKR
LLLENTVYTMDLRSAHKTPEKAPPPLRLSLTRSLIPEDEDDTYSDCTQIPRSHAAYCKSRPRTSQFMCLDSNPPSPTQ
AEWEGRGEDGEDVIGTEEDMEEETAVEQEETDKWSSAVGPDEITASEMPNSSWRPIIGSGEVTVTDISLNSLTVTFRE
SRVAKGFFRGLGLEV

>Bsp_LOC114860262
MELSSIGDQVFAVESITKKRVRKGNVEYLLKWQGWPPKYSTWEPEDNILDPRLVLAYEEHQEKIRALAYRRKGLRPRS
LVLRNVFAMDLRSAHKMAEKPPPRLRLSLTRSMSTDVDQGEHAMYRRPGRRRIRHRAGGKRGPSIKHVRPLRTREKPM
EEDWSGTSEEEKHECESAEERREDSVYGRSECSSPPLLERQDLELEVEEQADVELTAAAPWSDRPGGAAPTQTLACDP

TMDSVSARVAGPGDAVAPGDAPAPHSGEEESGPGPRSERNNTASVIVRVQRSGETAGGDNQGVTTATAEHPGKVIVTD
VTINSLTVTFKEAMTAEGFFKGH

>Bsp_LOC114860488
MELSAVGESVFAAESIIKRRIRRGRWEYLVKWKGWSQKYSTWEPEENILDARLFAAFEERERERELFGPKKRGPKPET
FLLKAKAKEKTYDFRRETPRGISYPVPEPVITPRAREGLRAVVPTIFPPSAVNRGESVHVRPPEPERRPRPALSLQEP
SRFPKKRGRKPKLSLNYDRDDGPSSAEPDTKRSRLLEEPVPHGPSKMPRRLHHYGETSDRSLIQLTRRFQEETTITPK
SCVGSASLSFSCAFSPDQGVHRTHCVSRASLARSSKNRNHRETEGRLTREQPGAVCAQPESTDGSWTPSFTSLDRVTV
TDVTMNFLTVTIKESSTDKGFFRDKR

>Bsp_LOC114847134
MELSAVGERVFAAESIIKRRIRKGRIEYLVKWKGWSPKYSTWEPEENILDSRLFAAFEQKERERELYGPKKRGPKPKT
FLLKAQAKVKGKAYEFRSEAARGMRVTYPSPEPVVTPRAREGLRAVVPTIFPPSTVNRGESVRVPATELATCEIRQPS
LRQTCPGSLMQVPKKRGPKPKPRFGSTASEPHKRRDEEQLSHSPHTLATLQGVEDVTLVKFAQRHPEDHSHCHKHHHH
HRHQHHHHTLSQGLSGGSSYKQLYLDHSPRPHRMDVNIHMAKDGSSHLAPAHFKHQMEKPYFLDRPSPTRFDDDADEV
TWRPSLGNVEKILVTDVTTNFLTVTIKESSTAKGFFKDKR

>Gwi_LOC114481946
MEAVTVGQVFDAECILSKRPRKGKLEYLVKWRGWSSKHNSWEPEENILDPRLLAAFNKREQERELLLQKKGKRPRGRP
RKILPPTPAVAEEGHSSSSSSMCSDLSFSSSDEDQKVKLLPQKRPQMLPAKLEPPRQKKKKRGRKPLALPSSSPPPQQ
RPPHRKPLQPASFTFTGLSRPGSLSALWVRGSDGAPYRHSSFSRSQSFHHKQPNKASASPRERNNQALNLRALNLQSV
TKPSSSYPVSTSNYPVTSAAKYPGSAGRPSTVQRSVLPAGGISGPERTAVRRSEDGKAVLNELSTGDSDDSTSESELG
RASFPSNNRPALRDEDTETDWRPATTLVDHVFVTDVTANFITVTVKESPTSVGFFHSRNH

>Gwi_LOC114469338
MELPAAGERVFAVESIEKKRSRKGRVEYLVKWRGWSPRYNTWEPEENILDPRLLDAFQDRERQEHLMGYRKRGPKPKH
LLVQVPSFARRSSILTDLDEATLKGDLQKPPSLQTLLPQAQQYTLNSKKLHQLQPLGREAEQQANTKKFYYQLNSKKH
HHYQPDHKAREAAFAKPREVNKGYNLPPVLQQKWVRDKDSGCLTKVKDITMELKKLPADLNGHKEPEKVPTKEATLPQ
SNGVRSEKLKIVKNKNKNGRIVIVMSKYMENGMKTVKIKNVDCENAEKLPHGADSVSENHLQKMKLVKELGLMNGFAK
HHKDRATVLSTGFNNTDCSTEKKVLTPKTDLAVTKQDKHAEVKGQGQLPADQPFHLTTKPNLPSPPSGRGAASPEDNT
GGEGRTQGLKRHVSGADGEEHLATKRFLSSSISGSNATSSPSQNISGDQNELQGCGFMEQDEPIDLSMVKSRPETQRD
ALTDTQPQKPSDVNKEDSVSCKEKRQTGTFPNFQPFLGNIIITDITTNCLTVTFKEYVTA

>Gwi_LOC114468810
MELPAAGEHVFAVEGIEKKRIRKGKIEYLVKWRGWSPKYNTWEPEENILDPRLLVAFQHRERQEQMLGYHKRGPKPKH
LLLQVPSFARRSTIPTNIEETSQGPEDNQKADPNQVQCSQPQQYQLNSKKHHQYQPSNQEVPADQLTNCKKKFIYQLN
SKKHHHYEPDPHMYDGQASRLKKLVKVQEGASKPTNPGWNLPLALQQKWVRDKDTGCLSKVKELAVEARKPAVQEADS
EHALKPNPKDATLPSAISSKMKIIKNKNKNGRIVIVMSKYMDVNKVHGIKGKHGESSREEKPHNTKPSENNPAHRTKP
AEHPENGIPKESCNGSPLSAAEHPSKCSPKDRHFSKPSPSTAEEYNTEVARGQADLPDDLPLQLTARSPSWTVDTNIP
TPTAADQLRIPSFPSDRKRKLLDTVEDRSAAKVHLTSRNFSVPSTVVTPPQDKPMDLQLSASRHSNKCTYEIMGSQDE
PMDLSCPKAKRQVEAELQLEPVPQPELETEPEPEPEPEAPEPDKDTPPVTETETVKPTEEAKQAPVQKLSPFMGNIII
TDITTNCLTVTFKEYVSD

>Gwi_Cbx6 (Gene ID: 114466377)
MELSAAGDRIFAAEAILKRRVRKGRLEYLVKWKGWAMKHSTWEPEENILDDRLILGFEQKERERELHGPKKRGPKPKK
MLMKASSQPTASSSSAVPHHFPSTSSSSSPVGLLPKLNSLAATHKLKKDIHRCHRMSRRPLPRCDPTASSFSNPGGFP
SRMHVSPFSETVRIVNRRVKPREVKRSRIILNLKVIEKSGRGGGSASRSRNVQSGRHNIPSRNRIIGKKGEAPYRPFQ
PPLKMLGFPMYGKPFGLQCGGGALPSHANATASRAPPATQDQDRSDRSEEGGKDVPPPSTPPPAPSEEQTAPPAGAQR
VPAEGDPDWHPQRAPSCKDVVVTDVTTNLLTVTIKEFPSPAPASGPASPSASPDHGSAPDDASVAKP

>Gwi_LOC114459779
MELSAAGDRIFAAEAILKRRVRKGRLEYLVKWKGWAMKHSTWEPEENILDDRLILGFEQKERERELHGPKKRGPKPKK
MLMKKGESSSSGRALNSRLKTSRSSSSSSSSSSQPTASSSSAVPHHFPSTSSSSSPVGLLPKLNSLAATHKLKKDIHR
CHRMSRRPLPRCDPTASSFSNPGGFPSRMHVSPFSETVRIVNRRVKPREVKRSRIILNLKVIEKSGRGGGSASRSRNV
QSGRHNIPSRNRIIGKKGEAPYRPFQPPLKMLGFPMYGKPFGLQCGGGALPSHANATASRAPPATQDQDRSDRSEEGG
KDVPPPSTPPPAPSEEQTAPPAGAQRVPAEGDPDWHPQRAPSCKDVVVTDVTTNLLTVTIKEFPSPAPASGPASPSAS
PDHGSAPDDASVAKP

>Gwi_LOC114467897
MELSSIGDQVFAVESITKKRVRKGNVEYLLKWQGWSPKYSTWEPEDNILDPRLVLAYEENQEKNRALAYRKKGLRPRR
LLLRNLFAMDLRSAHKGTEKPPPRLRLSLTRSMSTDVDQTDRRRVYRRAVRRRSQQRVSRRGSERESNKAFHRQRKKV
EDEEWESTSEDDKCESTTEERCADSVYGQSECSSPPVLERQDLEVEVEGKVENDITAVSTWADGVGGATFQTGHDETF
EKSQDSVQPEYSVAGGDREGGNEGGVCQSVCSVVEARGDNQSVTTTTKDGPGKVIMTDVTINSLTVTFKEASVAEGFF
KGC

>Gwi_LOC114481622
MELSAIGEQVFAVESVVRKRVRKGNVEYLLKWKGWPPKYSTWEPEEHILDKRLVQAYEEKKQREQAVGHKRKRSRSKR
FSLQNPEYSMALRSAHRPPEKAAPRLRLSLMRSLSSDQQEEPFDVCGRSQQAVADQRSKDRSSLFRCFDSNPSSPALK

DWDGSREEEEEEGEDEEEDEDSILEDINEEEVRLKATRAKKSKSTLKGQTRKALWSSHDGPDQDSSAERVWTPVDRPGE
VTVTDVTLNSFTATFRESPVAKGFFRNGGLDV

>Gwi_Cbx8 (Gene ID: 114471454)
MELSAVGERVFAAESIIKRRIRKGRIEYLVKWKGWSPKYSTWEPEENILDSRLFVAFEQRSVCSPVCSPVCSPVCSPV
CRPVCSPVCSPVCSPVCRPVCRPVCPKHLCTLLLSRSMCVCGSHREREREMYGPKKRGPKPKTFLLKAEAKVKARSYE
FRSDTVRGMHVSYPTPEPVVTPRAREGLRAIVPTIFPPSAVNRGESALVRAPEWSPRGHSQSPYRSSSDERIHVPKKR
GPKPKPRNSPTASSEQGGPKLHQEEEEEEEEARPAKFIHHRHPENHGYSHKHHHHHPHHHHRLTLSRGLSAGSPYKQL
YAQRGLSLHRTDPPQPHRTKSSGVLLAPAHFQHRSSLTPTRPAQPPPPPPMEKPYFLDRQSPSRLDEHLDEVTWRPSL
GNVEKVLVTDVTTNFLTVTIKESSTSKGFFKEKS

>Gwi_LOC114468472
MELSAVGESVFAAESILKRRIRRGRWEYLVKWKGWSQKYSTWEPEENILDARLFAAFDEREREREELFGPKKRGPKSET
FLLKAKARTKETNYVFRRESPRGIRVSYPSPEPVLTPRAREGLRAVVPTIFPPSSINRGESFLFRPPEPERRPKAADV
IATDLESFPKKRGRKPKLRVLYAEDDGSEPSEPDAKRSRSLIESKTSRHLMHHHGETSDHSLLQLTRRFQEKTTITPR
SNCARSYTCAFSPDVRKGDQTGERSRAGVLQPGTLQRKHPGRESTFQAAPQRAPGAWSPRLMNVGSVTVTDVTMNLLT
VTIRECTTDKGFFTEKR

>Pma_ENSPMGG00000018267*
MEELSAVGEQVFDAECILNKRLRKGKLEFLVKWRGWSAKHNSWEPQENILDPRLLAAFNKK

>Pma_ENSPMGG00000007479
MEGVTVGQVFDAECILSKRPRKGKFEYLVKWRGWSSKHNSWEPEENILDPRLLAAFHKRFVLPPVTPVPSDPHSSSSS
GLSSSCSSSDDEHRPKKSSRSHRTLPVPQKRPQILLKSESSHHKSKKRGRKPLPPDLKALRQSKTRPPTSASLSPPSV
ARPHRDLNVKKPLQPASFTYTGMSRSSREDAHTSAASFTQSSASKSGTVSSMWPGSAAGRALSLTTASPHKTNAATQS
KSKDANAKDRLSSAERRGETNRSVPELSEGESDEEDDSSSDSDHSGTLGGAGVTAANVMVAKAQMLFPVEGRPSLGAH
VDSDSDTEESNWRPSRNVLEHVFVTDVTANFITVTVKESPTSVGFFNS

>Pma_Cbx4 (ENSPMGG00000020462)
MELPAAGEHVFAVEGIEKKRIRKVSPYNTWEPEENILDPRLLVAFQLRERQEQLMGYRKRGPKPKHMLLQVPSFARRS
TIPEALEESPPEPEMSPKSDPVPVQRSQPQHYQLNSKKHHHKKHHHYEPDPSMYDTQSSRLKEVVKVQEVPSKPSNPG
WNLPLALQQKWVRDKDTGCLSKVKEMAVEMRKPAKEIDTVQVLKSDPKDISLPSTISSKMKIIKNKNKNGRIVIVMSK
YMDSKVNGLKTKHGESSGEDKGMESNKPLENSPLQRTKVSEAFHPDNGKSPPAAEIPIKCSPKDRHFSKPSPSTAEEY
NTEVARGQADLPDDLPLQLTASSPPASWTVDTNIPIPTAVDQIRIPSFPIDRKRKLSDPVEQRNITKAFLMSRSLSMP
SAVASPPQDTPMDLHCSRSHNSVRTYQTTDSQDEPIDLSYPSLVDALKLPEKAQEAPKTPSPFMGNIIITDITTNSLT
VTFKEYVSF

>Pma_ENSPMGG00000010294*
MELPAAGEHVFAVESIEKKRSRKGRIEYLVKWRGWSPKYNTWEPEENILDPRLLDAFQDRERQEQLMGYRKRGPKPKH
LLVQVPSFARRSNILADICEEESQQKSNSIQMQYQLNGKKHHPYQPLSQDLFIFSHIIFKIFVGL

>Pma_Cbx6a (ENSPMGG00000019149)
MELSAAGDRIFAAEAILKRRVRKGRLEYLVKWKGWAMKHSTWEPEENILDDRLILGFEQKERERDMNGPKKRGPKPKN
VVLKTRAPRGEASGCSTPPTAPSGPTPSPKLHSLAATHKLKKDIHRCHRMSRRPLPSMSPALSSAGGVRSRPHVSPFS
ETVRILNRRVKPREVKRGRIILNLKVIDKAGRGGGAAGSRGLSTGRHNIPSRNRIIKKGDAPYKPFQSPFKMLGFPMY
GKPFGLQCGGSFPSEPGPFSYSTIPSQATPSQSIPSQATLSQDAPSQALLQRGSEKQVFPAQCSEDQAEDVTLSAGLV
PAKGSEQGSEGPPKGASRWHPQLAPSCKDVVVTDVTTNLVTVTIKEFPSPSDKALQGKRISDLFFQVPNRFKPGL

>Pma_ENSPMGG00000017778*
MELSSIGDQVFAVESITKKRIRKGNVEYLLKWQGWPQKYSTWEPEDNILDPRLVLAYEER

>Pma_ENSPMGG00000010296
MELSAVGERVFAAESIIKRRIRKGRIEYLVKWKGWSPKYSTWEPEENILDSRLLADSAVSWRPSLVTMENVLVTDVTT
NFLTVTIKESRTSKGFFRDKR

>Sfa_LOC115392810
MEGVTVGQVFDAECILSKRPRKGKFEYLVKWRGWSSKHNSWEPEENILDPRLLAAFHKREQERELLFQKKGKRPRGRP
RKILPPAPAVTKDSRSSSSSSALSSSASSSEEEEDEEEDDDEDHGKKAKPGPRAPPVPQKRPQILLAKPDPPRKKKRG
RKPLHPDLRALRQARGRPAPSPPPPPPPPPAGPPRHPQAPRGPRDEARPGVKKPLQPASFTYTGLHRSAREEAASHSS
SSSSFSPSGCVWSGRSLCASSATSSSHGRAGASSHSKSSLDLKRSASDAAAFRASSLKHGGSSGSLGLHAGFAGGQMA
QRSALAQRRQDGAAVPHKQQSSGLSRPAPAPTPRERANQALSLRALNLQSVGSLPGGTGAAARASLRSAGGMLVKGGA
KDSRTAAGGQRCGPSAGGGAEAGRAKEKLPGGGGGGRPEERKHGLGTQSRSLTELSAGDSDESSSSESEHAALFPSNS
RPSLGNTAAESDTETDWRPARSLLEHVFVTDVTANFITVTVKESPTSVGFFTEPSH

>Sfa_LOC115385554
MEELSAVGEQVFDAECILNKRLRKGKLEFLVKWRGWSSKHNSWEPQENILDPRLLAAFNKKEQEKELLMRKKGKRPRG
RPRKVLENVPEVSKSSSSSSGSSSSSSDSSSSCSSSSSSEDEDEEDGGGETPGTAGRTRELHPVPQKKAQIVVAKQSP
PRKRGRKPPPADAKDFPPSKGARKVPKTARDPDLPGAIKKPVHPASFTFMGFHRGASREAAAGPARGPLTPGGAAKNP

AGGPASSGRSPVQTASLSLNRSGHNRSLSDGRQAASGASLDLKTAAGKSKGVAALNLNTSKPPGQGTAQLSPPSGQRK
PPAPLSTLPRIPHPKPAASVPSKNPPSGPGPGLQPLNLQSKLAQSGGAPRTAAKKTPVSQNQERSAPKTPPTPQRLAV
RKPQTPADRVQTARVPGRLEKTCGEVQNPERTGSKEGKKARMNDMSTGEDESSSDSDQDSSYPGRPPVQNQDWKPTRS
LIEHVFVTDVTANLVTVTVKESPTSVGFFSLRHY

>Sfa_LOC115385547
MEELSAVGEQVFDAECILNKRLRKGKLEFLVKWRGWSSKHNSWEPQENILDPRLLAAFNKKEQEKELLMRKKGKRPRG
RPRKVLENVPEVSKSSSSSSGSSSSSSDSSSSCSSSSSSEDEDEEDGGGETPGTAGRTRELHPVPQKKAQIVVAKQSP
PRKRGRKPPPADAKDFPPSKGARKVPKTARDPDLPGAIKKPVHPASFTFMGFHRGASREAAAGPARGPLTPGGAAKNP
AGGPASSGRSPVQTASLSLNRSGHNRSVSDGRQAASGASLDLKTAAGKSKGVAALNLNTSKPPGQGTAQLSPPSGQRK
PPAPLSTLPRIPHPKPAASVPSKNPPSGPGPGLQPLNLQSKLAQSGGALRTAAKKTPVSQNQERSAPKTPPTPQRLAV
RKPQTPADRVQTARVPGRLEKTCGEVQNPERTGSKEGKKARMNDMSTGEDESSSDSDQDSSYPGRPPVQNQDWKPTRS
LIEHVFVTDVTANLVTVTVKESPTSVGFFSLRHY

>Sfa_Cbx4 (Gene ID: 115390982)
MELPAAGEHVFAVESIEKKRSRKGRVEYLVKWRGWSPRYNTWEPEENILDPRLLDAFQDRERQEQLMGFRKRGPKPKH
VLVQVPSFARRSSILADLREDEDGRHKPASIQMLRPQAPQYTLNSKKHHQFQPLCREREAEQQANGKKFYYQLHSKKH
HHFQPELKAHEPRFAKPRDAKAPAAAEKGYNLPAVLQQKWVRDKDSGCLTKVRDITMELKKLPADLNGRQEPEPARTK
DAALSRANGVSSGKLKIVKNKNKNGRIVIVMSKYMENGMQTAKVKAGGCEAVSLPPGADGDSERHLEKMKLVKKLGLM
NGFAKSLKDKPGAPGGGLNGDGPRETEQSPRTEPAVTEKDKLGGVEGQAQNPTRQPLLLTARCPLLSPPLERAVPPPT
DPTGSPGGSEGLKRRLSEPGAEEHGGAKRFFSCRSISAPNAASSPVQGVSADQNGRRDVSGPQGVSEPENCGFPDQDE
PMDLSMVKSRPAAAPETHTRSDTQAQAETLKPAEGDRDASPLPVSHKEKTKEDRFPAFQPFLGNILITDITTNCLTVT
FKEYVTA

>Sfa_LOC115386984
MELPAAGEHVFAVEGIEKKRIRKGKIEYLVKWRGWSPKYNTWEPEENILDPRLLVAFQHRERQEQMMGYRKRGPKPKH
LLLQVPSFARRSSIPASLEEASQDTDSSVKSDPIQVPRSQPQQYQLNSKKHHQYQPSSQEVPADQLTNSKKKFIYQLN
SKKHHHYEPDPNMYDAQASRLKEVVKVQEPASKLSNPGWNLPLALQQKWVRDKDTGCLSKVKELAVEVRKPAVKEAES
EHALKPNPKDATLPSAISSKMKIIKNKNKNGRIVIVMSKYMDSNKVHGAKGKHGESSREEKPQNAKPSENNPAQRTKM
AEHPENGIPKEICSGSTLTAAEHPIKCSPKDRHFSKPSPSTAEEYNTEVARGQADLPDDLPLQLTASSPSWTTDTNIP
TPTAVDQIRIPSFPGDRKRKLSEPVEDRSVAKVYLTSRTLSAPEPVVTPPQDKPMDLHCSGPRHSSICTYEALGSQEE
PMDLSCPRAKKQVEAELQPEPEPAPAVTETPPATETNTQKPAGESKKQPVKKISPFMGNIIITDITTNSLTVTFKEYV
TF

>Sfa_Cbx6 (Gene ID: 115389716)
MELSAAGDRIFAAEAILKRRVRKGKLEYLVKWKGWAMKHSTWEPEENILDDRLILGFEQKERDREIHGPKKRGPKPKN
LFVKARAKKETSNRASSSRQSASRSSSAASTRAAPPSSSSSAAPQPVPSSSSPSAVSLTPKLNSLAATHKLKKDIHRC
HRMSRRPLPRSDPMASTFPNAGGFPSRMHVSPFSETVRIVNRRVKPREVKRSRIILNLKVIDKPGRASTSASNRNVPP
GRQNIPSRNRIIGKKGEAPYRPFQPPLKMLGFPMYGKPFGLQCGGPMPFQPYPVSCSSRGARDGRTTSSQLPSPPPPS
CSGGSEGTSLTSASAAQSQPTPGAKCSKPAESAKSFAPSPSSNTNQAAPESPLNSPSPSSSLGDEEEGPTQDPSPRQN
STAKLQSPTTLPLVASGDQTAAPVEPQRVPAEGDPDWHPEMAPSCKDVVVTDVTTNLVTVTIKEFPSPASGPASPSAS
PDSTMSPPPAATSEDPSPAKP

>Sfa_LOC115387617
MELSSIGDQVFAVESITKKRVRKGNVEYLLKWQGWPPKYSTWEPEDNILDPRLVLAYEENQEKIRALAYRKKGLRPRR
LVLRNIFAMDLRSANKEAEKPPPRLRLSLTRSMSTDVDQVERGGIYRRPLRRRRSRQRATRGPPEGAAGKPIRPQRKK
VEEPTEEEDEEEWGGTSEEDKHESESTIGERCEDSVYGPSECSSPPLLERQDLEIEVEEKVEDELIAARTEIWTDGEC
GGTSDTGHRETVACDQSKDSASASEDRAGDEASVGDRSDWEGVESREGRLCPRSNATSVIVSILGNRETAGDGTTTTA
AGAAVRSSDEAGKEEARGDNQSVNTAEPGRQIGKVIMTDVTINSLTVTFKEAMVAEGFFKGY

>Sfa_LOC115392998
MELSAIGEQVFAVESIVKKRVRKGNVEYLLKWKGWPPKYSTWEPEEHILDKRLVQAYEEKEERERALGHRRKGSKAKR
PLQNTVYTMDLRSAHRPPEKPAPRLRLSLTRSLPSEDEDPTFGACRRSLHRKSKQRSQIRCLSSSSSSSTPDDWEGEE
EEEEEEEDVIEDDEDKEDDGVQKETAKTIEGTLNGNGRTGDWSAAAGPEEPAAPEGAWRPPVGPGQVTVTDVTLNCLT
VTFRESRVAKGFFRDCEPEV

>Sfa_Cbx8 (Gene ID: 115390143)
MELSAVGERVFAAESIIKRRIRKGRLEYLVKWKGWSPKYSTWEPEENILDQRLFVAFEQREREREL YGPKKRGPKPKT
FLLKAQAKVRAKSYEFRSDAVRGMHISYPTPEPVATPRAREGLRAVVPTIFPPSTVNRGESVRVRTPDWSPRESPLRH
ADPDELPHSPKKRGPKPRARFLDSSEAHRRWAEEPASHGAHKLAKLQGAEDMRKFSHRHPDNYRHHHQHHQHHTHGRV
LPKQLYSDRLQPHRTDPDPHRTPDGPGYLAPAHFKHHSRPGPGPGPGPGPSRPAGLPLMEKPYFLDRPSPTRLDDDPD
EVTWRPSLGNVEKVLVTDVTSNFLTVTIKESSTSKGFFREKR

>Sfa_LOC115386985
MELSAVGESVFAAESIIKRRIRRGRWEYLVKWKGWSQKYSTWEPEENILDARLFAAFEERERERE MFGPKKRGPKPET
FLLKAKAKAKEKTYDFRREAARGMQVSYPIPEPVVTPRAREGLRTVVPTIFPPSAVNRGESIPVRPPEPERRPRLPPP
ADLMVHDTVHYPKKRGRKPKMQLHYDHDKGGGSAEPEAKRSRLLEGQGSHGLSKPSRPVHYHGEPSDRSLLQLTRRFQ

EETTITPKSRHMGGYACAYRPDVHKGVQGGHRTYSVSRMSIPHSSHQAKMYCPPRDQPSASLQSVHEASTQRVSSWRP
SFANMDTVTVTDVTSNFLTVTIRESSTDKGFFREKR

>Ola_Cbx2 (Gene ID: 100125497)
MEELSAVGEQVFDAECILNKRVRKGKLEFLVKWRGWSSKSSHAAFSSIREQEKELLMLKRGKRPRGRPRKILVRTSRR
AAPRLSDSDRTNARFFSTIQDSLPEPPKSSTSSSAMSSSSDSSSSCSSSSSSSEDEDEDEDSHLQQTAPTVRTRDHDP
VPQKKAQILMAKPDPSKKRRGEAKELQQNKAPPKTDKGPDLPGGPHKPVQPPGFAFLGFHRGSAREAVGAQNRSPLAQ
GGGVKNAPGSAGPGRSSVQTSLPLNKSGQSRPSAVSSGASLDLKKAKGVAALHLNPSKHPLQGAAQHTPGGQRKAHTP
LSTMQRMLSTKTGTPPPKNPPSNPGSGPQPLNLHHKLSQGSGTAGNAQRGAATPAKKAATPAQVHHDDPQRRPAARKS
QPDKSGSEVQKQPERSKKPRMTEMSTGEEESSSDSDHDASFNGAAVQNQDWKPTRSLIEHVFVTDVTANLVTVTVKES
PTSVGFFSLRNY

>Ola_LOC101172611
MEGVTVGQVFDAECILSKRSRKGKFEYLVKWRGWSSKHNSWEPEENILDPRLLAAFHKREQERELLFQKKGKRPRGRP
RKILPPLTPDKDSRSSSCSSSGLSSPRSSSSSEDEEHTKKAKPSPVPQKRPHIMLAKADPPHKKKRGRKPLPPDLKAL
RQVKNRPSPPPPPPPPPPPRHHQPIRLPREDTRPGIKKPLQPASFTYLSRTSREEAAAPSLAFSQSAASKVGSISMWVG
RSLSASSPSSSKTSLSQKNSYLKRSFSESGGGSRADGLKMSAVKPGGTSSSPGAHGSFSGGQVGIQSPAPGQKRPEGQ
AAKTGVVQHKQQNPVLSKPSSSSPFLRDRASQALNLRALNLQSVGKTYAGGAHGSTDFRSGTAKGGPRSGLSAGGGAE
RGRDREPAIRRDGALRDRRNQRSGSQDRGLNELSTGDSDEVSSSESERGAALFPDKIRPSLGASATDSDTETDWRPER
CLLEHVSVTDVTANFITVTVKESPTSVGFFNSRNH

>Ola_cbx4 (Gene ID: 101158167)
MELPAAGEHVFAVESIEKKRSRKGRVEYLVKWRGWSPRYNTWEPEENILDPRLLDAFQDRERQEQLMGYRKRGPKPKH
LLLQVPSFARRSNVLADLQEASLEDDGRQKSGPIQTLLPQAQQQYQLNSKKHHQYQPLCREAEQQTNGKKFYYQLNSK
KHHHYQPDLKIHEPVFAKPLEVKAPELASKGYNLPPVLQQKWVRDKDSGCLTKVKDITMELKKLPADLNGLKGSDKVK
TKEESSANGGGKLKIVKNKNKNGRIVIVMSKYMENGMQTAKIKNGDCEGAGKPAEGLEGGMEKHLEKMKLVKKLGLMN
GFPKPPKDKPIVLCSGFRAESSPKEKETSAQIQLALTEQDKHDGVRGQEELPQDHPLQLTGKPNLLSLPLEGRAPSSP
LDTRGSCQGLKRHLSDADGEEHNPSKRFLSCRTLSTPSAAPSPDRTGAAGVSSLRDCGYAEQEEPIDLRIVKPRPSAA
AEPPERPQDDSRTETPALPQPLEPAEDKAGSESELESRKEDAFPSFQPFLGNIIITDITANCLTVTFKEYVTT

>Ola_LOC101160166
MELPAAGEHVFAVEGIEKKRLRKGKIEYLVKWRGWSPKYNTWEPEENILDPRLLVAFQHRERQEQLMGYKKRGPKPKH
LLIQVPSFARRSSIPAGLEETSQEVEITPKSDSTQVQRSQPQQYQLNSKKHHQYQPSSQEVPADPLPNSKKRLIYQLN
SKKHHHYEPDPHMYDIQASRLKEVVKVQEAATKPANPGWNLPLALQQKWVRDKDTGCLNKVKELAVEVRKPAVKESEN
ALKPNPKDAALPTAVSSKMKIIKNKNKNGRIVIVMGKYMDKVHGAKGKHGESSKEGDSQSTKPPENGSKMVELSGSGL
CDSSSVSAAEHLMKCSPKDRHFSKPSPSTAEEYNTEVARGQADLPEDLPLQLTASSPPTSWTVDTNIPTPTAVDQIRI
PSFPSHRKRKLSDPVEDRNVPKTFLTSRSLSMPSTVVAPPQDKPMDLHCSGPRPSVCTYDAAGNQEEPMDLSCPKNRS
ALDAKAQPEPDSEAAVKDTPTVTEETQKSTEKLNDPPKNISPFMGNIIITDITTNSLTVTFKEFVSF

>Ola_cbx6a (ENSORLG00000025101)
MELSAAGDRIFAAEAILKRRVRKGKLEYLVKWKGWAMKHSTWEPEENILDDRLILGFEQKEREREIHGPKKRGPKPKT
AVMKARPQKGESSSSSSRQTTPHPTSSRTPPSSASSPHLLSSSSSSSAAAPSPKLNSLAATHKLKKDIHRCHRMSRRP
LPRSDPMASTYSSAGGFPSRMHVSPFSETVRILNRRVKPREVKRGRIILNLKVIDKPGRGGSASGSKNTAAGRQNIPS
RNRIIGKKGEAPYRPFQPPLKMLGFPMYGKPFGLQCGGPVPFPPHPGSCSSTGTRVSKNASSRYQSPPSPSSSSGSGE
KSPTGVTAAQSQPATEDVPPSKDSSTPAEGTQPAPASSSDPNPAASAATFLPSSPSSSLDEEQGAEDRSVPEEGGKKD
PCQRRAKQPPPSAAPSIPAGDQTSPLTDPPRVPTEGDTDWHPEMAPSCKDVVVTDVTTNLVTVTIKEFPTPASGPASP
SASPQNASSPPHVTSSEEPSATKP

>Ola_LOC101163772
MELSAIGEQVFAVESILKKRVRKGNVEYLLKWKGWPPKYSTWEPEEHILDRRLVLAYEEKEEREQALKGLKAQKLFLQ
DGVYTMDLRSAHKGPEKAPPPLQFSVASCLESEDEDDSNSDCRSRRPLAHRECKQRMSQRRYFDSSSSSPAQEDWNEL
ESQESVEEEEEEEGAANTYKGFLNEQRGADGWSSAVTPNAVEASAWRPGVAPGDVTVTDVTLNALTVTFRESGMATGF
FRPAQPACLQPHGSH

>Ola_LOC101168624
MELSSIGDQVFAVESITKKRVRKGTVEYLLKWQGWPPKYSTWEPEDNILDPRLVLAYEENQEKVRALAYRRKGLRPRR
LLLRSVFPMDLRSAHKMPEKPPPRLRLSLTRSTSTDVEQVYRRSVGRRNRLRVSSMGHVRSSSQHKRKKMVQVEEDWG
VTSEDDKPESEGTIEEKCEDSSYGQSECSSPLLMEPPDLDMEVEEEAKAPPAAESTETWTERPEDANQRQTSVCSQLT
DVQKSLSAFEAPPEVSEGSDWDFCEKEPECPEEKSGCCCERSNATSVIMRVQGSGVTPGDAADGEQDKKEAKDDNQSV
AMTTTDSPVPTGKVIVTQVTVNSLTVTFTEATEADGFFKGF

>Ola_Cbx8 (Gene ID: 101157915)
MELSAVGERVFAAESIIKRRIRKGRIEYLVKWKGWSPKYSTWEPEENILDSRLFAAFEQREREREL YGPKKRGPKPKT
FLLKAHAKDKARSYDFRSEEVRGIHFPTAEPVVTPRAREGLRAVVPTIFPPSAVNRGESVCLPSLQQHSSVSLVHTPK
KRGPKPRFSDPQLLKRRPEDQASPGSHKFTKIQGGEEASQVSVGHRHSENLGHGYKHHHHHHHHHSQSHGGCSGSFHK
HLHRTNPDTHRTKISSGYLAPAHFKHHSTAAPPAAPPPVEKPYFMDRPSPTRLDDDFDEVTWRPCLSNVEKVLVTDVT
SNFLTVTIKESSTSKGFFKEKR

>Ola_LOC101160413
MELSAVGESVFAAESIIKRRIRRGRWEYLVKWKGWSQKYSTWEPEENILDARLFAAFEEREKERELFGPKKRGPKPET
FLLKAKAKEKTYEFRMESPRGIQVSYPVPEPIVTPRAREGLRTVVPTLFPPSAINRGESVHVFTPEPERRPRPPPPPL
GAQESLRFPKKRGRKPKCLHHEKHDESAPEEPDAKRRLLEEMPTVPRRLHHREETSEHTLIQLTKRFQEKTTITPKGS
SQHRHVGGARLSYTCAFSPDVRKRDHDCLSRMQQSALRRAAEERRLRAMDSCALQRQVGVTSRPDHDSAPRSASCWTP
SFTNLDSVTVTDVTINLLTVTVRESSTDKGFFKEER

>Gac_Cbx2 (ENSGACG00000014410)
MEELSAVGEQVFDAECILNKRLKKGKLEFLVKWRGWSSKHNSWEPQENILDPRLLAAFNRKEQEKELLILKKGKRPRG
RPRKILENVPDAQRSSSSSSGSSWSSDSSSSCSSDSSSLDDAEDDERMALEPPGVRTRDLHPPQKKAQILVAKPELAK
KWISQPLSLDARELQQCKGPRDGELPGAIKKPVNPAGFTFMGFHRGIPRDAAAGPTRGSPGPGGSVKHCVRSVQPAPL
SLSRPVPSRSLGEGKLPVSGGTDTDPKASAGKSKGVAALNLNTPKHPAQGTAYHSPSPPTGHKKPPPPGGKVAAALPP
NNAPAGQCPGPPPLNLQGRRAQSGGVAGGPRSAAHPARQTPVLQNQEYNPAKGPAAPGRLQTRKSQPGADKVKEATGT
PTPRVQARLEPSGALTEVPTPLERSEGKKAKMNDMSTGEDESCSDSDQDPPYVVGGVRAVASQNLNQDWKPTRSLIEH
VFVTDVTANLVTVTVKESPTSVGFFMRHY

>Gac_ENSGACG00000010130
VMEGVTVGQVFDAECILSKRPRKGKSEYLVKWRGWSSKHNSWEPEENILDPRLLAAFHKREQERELLFQKKGKRPRGR
PRKILVDLLHSSRSRPLQKMAHSKKTKPRVHAVPVKRPPMAPPQPEPPCKKKRGRKPLHPDLRLLRQAKSSQQGLRPP
REDPRSAVKKPLQPASFTYTGLSRASREEASSHASSSSFSQTSSSKAGSLGCIWTGRSMSPSSYGRTGPAPPSSETGS
RGDGHKAPKPGGGSGSSVSHGGIGSLAAAQRSVLGQRRQDGPVQHRRENLPRERASQALSLRALNLQSVGAARSSARS
GAKRGARSGGEQARLREDQGKENRSLTELSTGDSEETSSSESERGGAASYPSGDLASGRLGLGGGGGSDGETDWRPAR
SLLEHVFVTDVTANFITVTVKESPTSVGFFNSRNH

>Gac_Cbx4 (ENSGACG00000011731)
MELPAAGEHVFAVEGIEKKRIRKGKIEYLVKWRGWSPKYNTWEPEENILDPRLLVAFQHRERQEQMLGYRKRGPKPKH
LLLQVPSFARRSCIPAGFEDSSPDTEAVLVSDPTSVQRPQAQQYQLNSKKHHQYQPNFQEVPADQPTNGKKKFIYQLN
SKKHHHYEPDPSMYDAQASRLKEVVKVQEPASKPANPGWNLPLALQQKWVRDKDTGCLSKVKELAVEVRKPAVKDAES
EHALKANPKDATLPSSVSSKMKIIKNKNKNGRIVIVMSKYMDSNKAHGAKGKHSESSSDVKPQNTKPSENNPAHTTKT
AEHPENGIPKQLCSGSSLPAAEHPIKCSPKDRHFSKPSPSTAEEYNTEVARGQADLPDDLPLQLTASSPLTSWSVDTN
IPTPTLVEIRIPSFPNDRKRKLSGPAEDRSVSKTYLTSRSFSVPSTVVTPPQDKPMDLHCSGTRHSSTYTYEAGDGGS
QEEPMDLSCPKNKNQVDMEILAEPEPAVTEPTQVVEDTHKSTEESKEANISPFMGNIIITDITTNSLTVTFKEFVSF

>Gac_ENSGACG00000018219
MELPAAGEHVFAVESIEKKRIRKGRFEYLVKWRGWSPKYNTWEPEENILDPRLLDAFQDRERQEQLMGYRKRGPKPKH
LLVQVPSFARRSSLLAGHHAASLDEDSCQKTGPIQLLRPQSQQYQMNGKKHHQYQPLCREREAEQQQANGKKFYYQLN
SKKHHPYQPDLKDHEAVFVKPRELKASELANKGYNLPPVLQQKWVRDKESGCLTKVKDIAMELKKLPADLNGHQEPER
VGPKEDAAPQSNGASNGKLKIVKNKNKNGRIVIVMSKYMENGMKAPKVKDGDSESGVEPPRGTDNGTENHLEKMKLVR
KLGLMNGLAKNSKDKPTVVSSGLNKRCPQEKEPSPKTEPTVLDKQDKHGGVRGQRQLPADQPLRLATQLDLPSDRGGA
SPLDKGGSPGGFQGLKRHLSDSEVHGSCKRSLSSRSTGALNEASSPTKSLGVDHNGPPSHVGPQDCGYADQEEPIDLS
VVKSRPSAAQPETHTQPQTARPAEARTQDETDTQADARTDAQRETERNEENVDSSSDSDHPFLGNIVITDITTNCLTV
TFKEYITA

>Gac_Cbx6a (ENSGACG00000019854)
MEVSSTGDRVFAAEAILKRRVRKGRLEYLVKWKGWAMKHSTWEPEENILDDRLILGFERKEREQEMHGPKKRGPKPKT
PTGSAPGRAPPSSLAPPHAASSSASSSTVAPSPKLNSLAATHKLKKDIHRCHRMSRRPLPRSDPMAPSFSTPGGLPSR
LHASPFSETVRILNRRVKPREVKRGRIILNLKVIDSVTAGRQNIPSRNRIIGKKGDAPYRPFQPPLKMLGFPMYGKPF
GLQCGGPASLQPHPGSSTEERNAGSASAHPERRPPTSGAAASPPTEAIESSQKGPNATPADASWSSDPPLLPSSSSSS
LPVAPGDQSSALTDSQRPPAEGDPNWHPEMAPSSKDVVVTDVTSNLLTVTIKEFPSPEDLE

>Gac_Cbx7a (ENSGACG00000007724)
MELSSVGDQVFAVESITKKRVRKGNVEYLLKWQGWSPKYSTWEPEDNILDPRLVLAYEENQEKIRALAYRRKGLRPRR
LVLRNIFAMDLRSANKVSEKPTPRLRLSLTRAMSTDVDQGERGSLYRRLARRKIKQRLSKRGQGGPTNKTLLPLRKKA
EPIEEDWGTSEEEKQESESTTEEIREDSLYGLQGRSECSSPPLLERQDLEMELEEKADADLTVVGAQTWADRPGGGTS
ETIQNQMFASDQSEGSVSAPEAIPEDEVSAGDGSDWDRGVESGSEGHMIGSGVSQTDNMTSVMVSVEKSSETSGDCGA
TEETKEEARGDNQRVTVTTPGDQSSPAAAQHPKNVISTDVTVNCLTVTFKEASVAEGFF

>Gac_Cbx8a (ENSGACG00000011737)
MELSAVGESVFAAESIIKRRIRRGRWEYLVKWKGWSQKYSTWEPEENILDARLFAAFEERERERELFGPKKRGPKPET
FLLKARAKEKTYDSRREAPRRIQVSYPVPEPVITPRAREGLRAVLPTIFPPSSVNRGESVLIQPPQPDRRPRSTPPAP
LTLQEDVRFPKKRGRKPKLHLHYDEDEPDAERGRPSEEQGSHGVSGASRRLLHRGETSDHGLVQLTRRFQEKTTITPK
HSGARGHAGLFYGCAFSPPDVHEREHRTTCLPGWSVPRSGSSAEHQRHQAKDLRLPPEQAAVTSTQSESTRDQSTRPA
STWTPRFTSLDTVTVTDVTMNFLTVTVRESSTEKGFFREKR

>Gac_ENSGACG00000018216
MELSAVGERVFAAESIIKRRIRKGRIEYLVKWKGWSPKYSTWEPEENILDSRLFAAFEQREREREELYGPKKRGPKPKT
FLLKAQAKIKAKSYEFRGDLVRGITYPTPEPVVTPRAREGLRAVVPNIFPASTVNRGESVRVHPPGLSSREYQQPSLQ
QTSPGGLIRLPKKRGPKPKPRFKDSSCSPAVFESHQRRAEEQVSYSPHKLAKLQGGEEMRRIKVAHRHAELHDNRGIT

GSSSYKLYSDRILHPHRTDMDTHRTKNRSTYLAPVHFKHTSKMSQGPSGPAERPPMEKPYFLDRPSPTRLEVDLDEVT
WRPSVGNVEKVLVTDVTTNFLTVTIKESSTSQGFFKDKR

>Hco_Cbx2 (Gene ID: 109527817)
MEELSAVGEQVFDAECILNKRLRKGKLEFLVKWRGWSSKHNSWEPQENILDPRLLAAFNKKEQEKELLIRKRGKRPRG
RPRKIPETVLEAAKSNSSSSGTSSSSSDSSSSCSSSSSSSSEDEDEEQQASPGVRMRDLHPNPVPQKKAQIVVAKQEP
PKKRGRKSLSKGLRKSLKKDSDLPGAIKKPVHLASFAFMGFHRGSAGCPNRSPPTHGGTSRNSLSTVGSARSIPPSPV
PLMKSSPGRNVTEGKVPTPRPDSFKPRGMEAFTSNTTKQHLPGSPQRTTNSPSVQKAHVLQRITNAKAMAAQAAKNIT
TNQPLNLHNKVTQGNKSSGSGSSPGSALRNKTNQNQEYKTPQSPASPGGTRKSAAGKDLTEIQTYRGKLEKGEVHKSE
NHLERPACKDGKMSTGEDESSSDSDRDSSDAGREPLAVVHNQDWKPTRSLIEHVFVTDVTANLVTVTVKESPTSVGFF
SIRNY

>Hco_LOC109518595
MEGVTVGQVFDAECILSKRPRKGKFEYLVKWRGWSSKHNSWEPEENILDPRLLAAFHKREQERELLYQTKGKRPRGRP
RKFPLPVSPAAKDSRSSSSSFSGASSSVASSSEEDDEAERDRKKAKGAAAGPRLHPAPQRRPQILPAKAEPHRKKRGR
KPLHANVGALRPAKSRTPPLPSAPSHHQLLPGFLRKYGKVDPRPGIKKPLQPASFTYTGLSRTAREHHTDAQSAVFSQ
VSAASKGGCVWNRPAPASSPSLSKAGGSPQRKTSHGELKRSMSGPGGSGGRGEALKGMPPPLRPAFGGGPTAAQRLAP
PPRRQDSHGGYTALVQHKRALAKAPSSSSQRPSNQTLGLRAVNQQNVTKTPLAGHQGNSGALVRSGAAVRSSSRSGSL
VVIRDTCVTPTGQRPVLPAGGGAEKARKDATLPAGGGRQVERKSGRGLNDLSTGDSEDSSSGEWERDATSYPSDGRPS
LGDAATESDTETDWRPTRNLLEQVFVTDVTANFITVTVKESPTSVGFFNPRNH

>Hco_LOC109527525
MELPAAGEHVFAVESIEKKRSRKGRVEYLVKWRGWSPKYNTWEPEENILDPRLLDAFEDRERQEQLMGYRKRGPKPKH
LLVQVPSFARRSSILAGRHESSLQGDSCHDTNSVPMLRPQSQQYQLNGKKHHQFQPMSRECETEQHANGTKFYYQLNT
KKHHHYQPGLQMHESVFAKPREVNARELPIKGYNLPPVLQQKWFRDKTSGVLTKVKDITMELKKLPADLNGHKEQEEA
KPKDPASVQHNGVSGSKLKIVKNKNKNGRIVIVMSKYMENGMEDAKVTNAESQSAEAFSQGADNAERHLEKMKLVKHL
GLVNGFAKKPKADSGFTGDSTKESEQPAHTKLAVTEQDQHFEVRGQCLLPAHQPLQLTPKSNLVSSPLDTGVPVFTDK
NTTQDEFQGLKRHHTDTDNEEHGSNKRLFISHHTLSSPTQSISTDQNDLRGPFSLQHCDYADHDQEEPIDLSVVKSRP
QVAASAGSQPERHTQDGTQTDSQVKEQTESQPQAETENIAIVDTVDSPTVGHQETGKVETAFPSFQPFLGNIVITDIT
TNCLTVTFKEYVTV

>Hco_LOC109522747
MELPAAGEHVFAVEGIEKKRIRKGKIEYLVKWRGWSPKYNTWEPEENILDPRLLVAFQYRERQEQLMGYRKRGPKPKH
LLLQVPSFARRSSIPAGLEDASADADDSPKSDAVPVQRSQPQQYQLNSKKHHQYQPSSQEVPVEQLANSKKKYIYQLN
SKKHHHYEPDPNMYDAQASKLKKVVKVQEAASKPANPGWNLPLALQQKWVRDKDTGCLSKVKEQAVEGRKAAPKDAES
EQALKADPKDATLPSSVGSKMKIIKNKNKNGRIVIVMSKYMDGNKVHGAKGKHGESSSGGEKTPNGLASPSNPAHRTK
MAENGLPKEICGSSSLPAADHPQKCSPKDRHFFKPSPSTAEEYNTEVARGQADLPDDLPLQLTASSPPTSWPADGNIP
TSTIIEHIRIPSYPSSRKRKLAEPADERRVSKTLLATRSLGVSGTVLSPPQDKPMDLHCTGRIAAYDAADRGGGQEEP
MDLSCPKTKRQTEPETRAEPAPRPEPQPELDPQPERAPRLEPQTETAAQPEPSPPTPAAPVASEDTPQKTKAALAKKI
SPFMGNIIITDVTTNSLTVTFKEYVSF

>Hco_Cbx6 (Gene ID: 109511478)
MELSAMGDRVFAAEAILKRRVRKGRLEYLVKWKGWAMKDSTWEPEENILDDRLILGFEQKERERERNGPKKRGPKPKN
LVIKPQKGETSSQCTKSRQAKSRSASSRVTPSSSSSSVPHTLAPTSPKLSSLAATHKLKKDIHRCHRMSRRPLPRSDP
MASSFPGSGGFPSRPHASPFSETVRILNRRVKPREVKRGRIILNLKVIDKPGRSGSTSSGSRSLPGGRQNIPSRNRII
GKKGEAPYRPFQPPLKMLGFPMYGKPFGLQCGGPLAFHPQTGSCNGRNTRPTPSDTPASTQSVPQSKTPAESPNIAPS
APANPAASLTLPTGEDAGDAPGASLPQGGGGPPEQARVPAEGDPDWHPQMGPSCKDVVVTDVTTNLVTVTIKEFPSPA
SGPASPVSAEGAASPLPCAADTTDTTPPKP

>Hco_Cbx7 (Gene ID: 109512097)
MELSAIGEQVFAVESIMKKRVRKGNVEYLLKWKGWPPKYSTWEPEEHILDQRLVQAYEEKEQRDKTLGHRRKRSKAKK
RLLLQNTVYTMDLRSAHKAADMPKPRLRLSLTRSLLPEEDEEEEEEEEEDEEEEEEEEEEEDPSYTAWRRAPRYNKRRP
RSRCLNFRPASPAHEDWKSLGEEEEVEEDLVGDEEEENEDTGEKTAGQRRSAAWSPVPSGDHLTEAEMPETAWRPVVG
PAEVTVTDVTINALTVTFREARVARGFFRVWGGGGLSGAPRFSKLIVNASS

>Hco_LOC109527051
MELSSIGDQVFAVESITKKRVRKGNVEYLLKWQGWPPKYSTWEPEDNILDPRLVLAYEENQQKIRALAYRKKGLRPRS
LVLRNIFAMDLRSAHKAPDKPAPRLRLSLTRAMSTDVDHIERGGAYCRSARRRRTKQLASKRSACARSNRTVGHRRKK
TEDWDDTSEEEKQESQSATEERRADSLYGHSECSSPPVLERHDLELDMEENGPKMFSDPEDVDVATIACDQSKDGAST
LDDGAADAVTADNGSDGDTGEEEGQSRPVSPTGAHLKDDLTSAVAGADTVVRIQNVTTTTATAQSQHPGKVIMTEVTI
NSLTVTFKEAAEAEGFFKDC

>Hco_LOC109522748
MELSAVGESVFAAESIIKRRIRRGRWEYLVKWKGWSHKYSTWEPEENILDERLLAAFEERERERELFGPKKRGPKPET
FLLKAKAREKTYEFRRDPVRPIQVSYPVPEPIKTPRAREGLRAVVPTIFPPSAVNRGESVVSQALEPERRPRSATSSH
QESSPKKRGRKPKLRHSYEQDDDESAEPAPKMSRCLPHDRTASDPSLAQLTRRFQEETTISPRSSSDHMGMAYTRTDL
GGRTHYMALLKHRDKYRNLPIITGEELGVVCPPTASGSSWNPRFTNMDTVTVTDVTMNLLTVTVRENYTDKGFFRGKR

>Hco_LOC109527520
MELSAVGERVFAAESIIKRRIRKGRLEYLVKWKGWSPKYSTWEPEENILDSRLFAAFEQRERERELFGPKKRGPKPKK
LLLKAQATKSYEFRNETMRGMRFSYATREPVVTHRAREGLRAVVPTIFPPSTVNRGESVFVSPPDLAHEQQANSEELV
HLPKKRGPKPKLRFQDSIPAAYEPDERRADELSNHSPQKLMKLQSGEDMKLLKVSHHRSPEYHGHSHKHHRHHRHHHH
HHSQNRPMTGRGSHKQLYAESDLHSQRDTHRTKNSSGPSKHQRGPSQLVPTEKPYFLDKPSPTRLDVNLDEVTWRPSL
CSVEKVLVTDVTANFLTVTIKESSTSKGFFKDKR

>Pki_LOC111855945
MEELSAVGEQVFDAECILNKRLRKGKLEYLVKWRGWSSKHNSWEPQENILDPRLLAAFHKREQEKEILFRKRGKRPRG
RPRKIMEAVPAATKSSSSSSSSSSSSSSSSSSSSSSSSSSSSDDDDNDGEEAKVKAGQRGRELHPVPQKKAQIVVAKQ
EPVKKKRGRKALPPELKALRQSKGARKLLRPAARERELLSQTQESKTPIKKPLQPASFTYTGMSRVGGAREPSGAQSR
GSFSQAVRNPLGAVGPTRPTATSPVSRVPQNKATPDFKLSVSDVGSGSDLDLRITTSKSPGVAALNLHNAKVSCGGGG
GQGTASSAAGTPNGQRKQETPAQTLSPRTAAQPLSKGPGLQALSLQGLNLQSVSRQTQGCVSTGNGTANPARKGPSQN
PGTGGGGQQTRRGLGGGQGRVTEAGELGLGTDRQAKKSRRPEAKPSTESPAARGRGRRPSSKDGGKPGKVLSEMSTGE
EESSSDSEHDSPLPSPAQNLSVSVQPGQDWKPTRSLIEHVFVTDVTANLVTVTVKESPTSVGFFSLRQY

>Pki_Cbx4 (Gene ID: 111833586)
MELPAAGEHVFAVESIEKKRTRKGRTEYLVKWRGWSPKHSTWEPEENILDPRLLVAFQNREKQEQLLGYRKRGPKPKH
LLVQLPAFARRSSVLSGLQGSSLDEDSRQKSDAVPMHRSQTQQYQLNSKKHHQYQPNCKEGQVEQQIHGKKKFYYQLN
SKKHHHYQPDPKMYDLQYQKTREGKGQESNSQGWNLPSALQEKWLCDKDISLNKISSLTEDMKKLPGQFNGSEPTLTS
NNKEPVVPNGISSKMKIVKNKNKNGRIVIVMSKYMENGMQSARIKNGDPNSAERPNDAKLSEDSSAESLLDKIKLVKK
PVQDNGLPKENKTEMSATPLPLTQSSVEQEHSAKEDTIVTEGEKTDECGSQPDLPQDQPLQLTTKPNLTPWPFEMGVL
SRLDHSTNQPGLSTSPSLKRQLSEPLEDRGRYKKFISSRSISTPSSVSSVPQTKPMDLNLSSFHCSKGPDCDFLDSIP
EEPIDLSCVKPRAEASTPPKSLTPVDQAKATTAAEAEDPKPCFKPFLGNIIITDVTANCLTVTFKEYITV

>Pki_LOC111843199
MDLPATGEHVFAVESIEKKRFKKGRIEYLVKWRGWSSKYNTWEPEENILDPRLLTAFQNREKQEQLMGYRKRGPKTKP
LFLQLPSFARRSGLFSEFQEMSLEEEHRSKSDPVMMSHLQPQQYQLNSKKYHQYQPNGKESVMEQQANAKKKYFYQLN
SKKHHHYQPDPKIYDLQFQKPKEVKGEEPTNHKWKLPPTTHQKWVCDKTPGCLSKMKDLSLELNKSEGDKEEISCNPK
EMDLPNGISSKMKIIKNKNKNGRIVIVMSKYMENGSQSAKIKNGDPSVVEKPVDGKRPEGSESQKFIDGAERIESPAL
ENGTSSDYRSVACNNDSKQTDGQNCSRGEQANEKELIGGDGVGVAVSRVEPNMTHQSPASPMMNKEGDRPAPSASRFL
KRHLSDPEEDHGRSKVFLSVRSVSAPGGVPTAADPEHPSYGLSYSQGQSLLGVDLEEPIDLSCVRSRQERDALPKAEL
PSEGDKAKKPEPSCAFRPFLGNIIITDVTTNCLTVTFKEYVSV

>Pki_LOC111845190
MELSAVGDRIFAAESIIKRRVRKGRIEYLVKWKGWAIKYSTWEPEENILDGRLIAGFEQKERERELYGPKKRGPKPKN
FVLKARAQAAETRGPSRVPDVRLRQSTGSSSRPSTSSAPSSSMSSSSTSSSSSAPTPKLHSGAASHKLKKDIRRCHRM
SRRPLPRPDPLASGSAGGPFPSRPPVSPFSETVRILNRKVKPRETKRGRIILNLKVIDKTPQPPTSRGRPNIPSRNRI
IGKRLNEVSYRGLQPPVKLPGFPMYGKPFGVQQVSSDVGRLRAGTGNSTRSCGGAGNSGPGSSNRNSASETHGGTAEP
PVSQYQAPPSPSSSGGVDSSPPSPGQAPPTPSEAPPQSLHVSAASASPPKLSPQASSEPEDALGLQPGPQPASYLPSC
PSPSSSPSSSEDDDEAAVDLSAPRITKRRPRGRPQRRRRPNRATAPGTSSPDPDSSPPLLSPEPPRAVLEGDPDWHPE
MAPCCADVVVTDVTTNLLTVTIKEFCRPPELDKSGPASPSSGATAPPSPTPSTPHPKP

>Pki_cbx6b (ENSPKIG00000001275)
MELSAVGDRVFAAESIIKRRVRKGRIEYLVKWKGWAIKYSTWEPEENILDGRLISAFEQKERERELYGPKKRGPKPKT
FLLKARAQAAETSTQVPDIQQYHSSASSTSRPSSSSASPASSLHSPYPSSSSSSSSSAPTVKLQSGAAHHKLKKDIRR
CHRMSRRPLPRPDPLAPPSGSSSSSSAFPSRPPVSPFSETVRILNRKVKPREAKRGRIILNLKVIDKAGGGRRAPSMA
HQQGMHHSQPGRPKIPSRNRIIGKSKRFTEVPYRGLQPPLKVTGFPMYAKPFGIQQPISQSQSSTGSSENTEGKGAGE
GAPPLPGSALLNPDSVMPEPQTLPRYQPSPSSSTSSGSDSGPNSPPHIQPTPLQAIPEPASAASPSPPNLSPKPLESK
QEAPHNPPSFLPSPSSIFLSSLSSSSSTSTSEDEEVLDLSVPHEDKRNARRRQHMRRLQGKSASAISIPLTPKNLGIG
PPLLSPEQTRTLSEGNPDWHPEMAPRANVVVTDVTTNLLTVTIKEFCHPPEFEKPEPPSPSSSSVSLQVATPSSAATA
TCQSKL

>Pki_Cbx7 (Gene ID: 111845235)
MELSAIGEQVFAVESIIKKRVRKGNVEYLLKWKGWPPKYSTWEPEEHILDPRLVLAYEEKEQRDRALGYRKRGPKPKR
LFLQNVYTMDLRSAHKTPEKPQARLRLSLTRSLAPEPGTELPPCQGGGGTLYGQLAQPRNKQRASQILRLSPQPATTP
PAPNRSNGDWGHREEDEEEEEMEEEEEVGQEAESDMLNGPDRAEGWSSSMDEESVTMRERPEGWNPVMGPGEVTVTDV
TINSLTVTFREALAAKGFFRDWGLEF

>Pki_LOC111846417
MELSSIGDQVFAVESITKKRVRKGNVEYLLKWQGWPSKYSTWEPEDHILDPHLVLAYEEKEEKDRALAYRRRGLRPRK
LLVQNIYSMDLRSSHRAPVKPPPRLRLSLSRSLGAELEARGRDRAAGGGSSRQKKRRRLSQGSSTERPARAAVRDFPN
QDWRAEDEKKETVVDAEKQADSVPISKSDLPDPGKEEQNPLSKLVQEELTGLGVVHDQPGGPTPVSELKQEATTHGSP
SDSSGSGAVTAGATEGQEANDPDASVQEQGVRTPTDGLAEDRDLGTELRAAADPPRTNGCILVTGPGEAAFNGAPLSD
PTVPTPSGQPESSKPPEETQSSAVSGTQESRMTPTVEPKEETTSDITGGAAPGAERQEGAPSAGEPGTVIVTDITINS
LTVTFREALAAKGFFRAWGSELKCGGGE

>Pki_Cbx8 (Gene ID: 111833462)

MELSAVGERVFAAESIIKRRIRRGRMEYLVKWKGWSPKYSTWEPEENILDSRLFAAFEERERERELFGPKKRGPKPKT
FLLRAQAKAKAKMYEFRSEAARGIRVSFPSPEPVITPRAREGLRAIVPMIFPPSTVNRGESVRVRPEEFARDHRPAIP
AKPVPDGLVSTPKKRGPKPKIRFKDNLYGEAEHPKRRSEEHMTYLPVKMRKHTPMESEKTVPLKVIKLHQRHQEDPGY
SQVQMRAAPSGSSQQLLGSERSSHAHRASLEMQSCRTPERPDAMPSVHPRLERLASQQSSLIAKIPVTRILGQAEEDS
WSPCLENMEKVVVTDVTTNFLTVTIKESSTDQGFFKDKR

>Pki_LOC111843316
MELSAVGERVFAAESIIKRRIRRGRMEYLVKWKGWSQKYSTWEPEENILDDRLLAAFKERERERELFGPKKRGPKPET
FLLKAQAKAQAKTYEFRSDMARGIRVSFPVPEPVITPRAREGLRTVVPTIFPPSTVNRGESVRVRPPEPDRGHRTSGS
PRPVAEGFVAAPKKRGRKPKLRFVEGPDSSASAVPAKRSRMEDQMAYNPAKVGKHGIPEGQKSELHGLQLNRRLQRDS
GYPLTQAPAVPSSSMFKYCSEVSSHKGGFHLPACRTKECAGAISTQQPRLKHLSKNNLYEPNSPSQRELPNRGTKSPV
SRVFEAAVEECWSPCPTKVEKVVVTDVTANFLTVTIKESSTDKGFFKDKR

>Sfo_LOC114909229*
MARLRYSVHQYKHQADRHHHPVTFRPGQWVWLSTRNLKPYKVSHLQAPRAPEPPLPVQVDGAPAYRVNELLDSLQSYG
QLQYLVDWEGYGPEERSWVPAQDILDPDLVMSFHRDRLDRPVPRPRGRPLRRAAEAAHRVEGTVMPPGRSEAPAFN

>Sfo_LOC108932946
MEELSAVGEQVFDAECILNKRLRKGKLEYLVKWRGWSSKHNSWEPQENILDPRLLAAFHKREQEKEILFRKRGKRPRG
RPRKTMESIPTTTKSGSSSSSSSSSSSSSSSSSSSSSSSSSSSDEDNEGEEVNEKPGHRGRELHPVPQKKAQIVLAKQEPVK
KKRGRKALPPELKALRQTKGARKLPKQTIRESPPMDPKIPLKKPLQPASFTYTSMSRASSTRDTQGTQNRGCFMQCAR
SPLSTVGPGRPTCPPTVNRIPQSKGAFDFKLSVSDMGSGSDVDLRIAASKSPGVAALNLHNAQVSSGMIAGQGSSLPM
GQLNGQRKQENTVQGSPQSSAALPPPKGSAHQVLSLQALNLQSISKPAQGCITAGHGCAPPRSTANPARKTLGGNPRQ
AQGSAQSPATSGSQQSRRTLLGSEQGKAVEGGDMSPIGDKQIKKLKRLNTNPPAEGGVTRGQGQRSSSKDSSKLGKVL
SEMSTGEDESSSDSEHESPPFSSNGQNMSVSLQTSQDWKPTRSLIEHVFVTDVTANLVTVTVKESPTSVGFFNLHQY

>Sfo_Cbx4 (Gene ID: 108939836)
MELPSAGEHVFAVESIEKKRIRKGRIEYLVKWRGWSPKYNTWEPEENILDPRLLVAFQNREKQEQLMGYRKRGPKPKH
LLVQLPSFARRSSVLSGLQETSLDEENRPKSDAIPAHRSQTQQYQLNSKKHHQYQPNCKETQVDQQVNGKKKFYYQLN
SKKHHHYQPDPKMYDLQFQKPRESKGQEHISQGWNLPPALQQKGFCDKDLGLSKASNFSVEKKLPDPLNCAEAPLTTD
TKETILPSGMSSKMKIVKNKNKNGRIVIVMSKYMENGMQSARIKNGDSDVGEKLHEGKLSEDDSAENLSDKMKFVKPR
LPDNGITKDSENGTHTPAAAVSIRQSSVQQINSSKEEPAVPEGGKTDEAVTQADLPQDQPLQLTTKPNLTPWPFEMGV
LSRVDHRRKQQGPSAASSLKRQLSEPLEDKGRCKKFLTSRSISAPSSVPSVPRNKATDPNPSNYSVSASQTCDFTDPI
PEEPIDLSCVKCRAESSSRPKTPPPVENNSAAVPERAEDPLPSFKPFLGNLIITDVTANCLTVTFKEYVKV

>Sfo_LOC108937346
MDLPAAGEHVFAVESIEKKRLKKGRTEYLVKWRGWSSKYNTWEPEENILDPRLLVAFQNREKQEQLMGYRKRGPKPKN
LFVQLPSFARRSSLFSEFQESSLHEDHHSQLDPVKIHSQPQQYQLNSRKHHQYQPNGKESQTEQQVNGKKKYFYQLNS
KKHHHYQPDPKMYDVQYQRSKELKDQELMNHEWKLQKKLACDKTSGCLSKMKELSLEVKGGDARDSREATFPRSISSK
MKIIKNKNKNGRIVIVMSKYMENGTQDAKIKNGDSSGVDEPLHKIGCEVGNLDPLTDGTAKTGCHVLEPSITKDDGDV
MSPKISKESKNQSSLEWADALEDKLSGPEQHEKGLAVPQGKVPEKQPSQVSIKPDEPQAPISFLPDRDGEQSVPMAAR
ILKRRLSETEETRDRSKVFVGSRSISTPAPSWQMSTDPQLTSYHSDFLDSGLEEPIDLSCVRSKPGSDSFARAKTPAA
EDSTWTKESVFSFKPFLGNIIITDVTTNCLTVTFKEYISV

>Sfo_Cbx6b (ENSSFOG00015008724)
MELSAVGDRVFAAECILKRRVRKGRIEYLVKWKGWAIKYSTWEPEENILDSRLIAAFEQKERERELYGPKKRGPKPKT
FLLKARAQAAETTTRVPELRQYQPAASSSSRASSSSASPASSSHSVYPSSSSSSAPTAKLQSGAAHHKLKKDIHRCHR
MSRRPLPRPDPLAPPQGSSGSSFSSRPPISPFSETVRILNRKVKPREVKRGRIILNLKVIDKPGSSNAGATSRRTAIS
HQQDSAHSTHPGRSRIPSRNRIIGKSKRFGEVPYRGLQSPLKMPGFPMYGKPFGIQQSGSGPGPAQTGPGHYATGTSG
SSAAKGTSSGAPSSLTSAQAYPDRPTGQPQVLPRYQPLPSPSSSCGSDSSPSSPPQIQPTSLQAQPEPPSASAASPSP
PSLSPEPIELKQKQAPTPTSFLPAPPSIFLSSSTSSSEDEEILDLSVPHEGKRNARRRHHPRRRPSKHSPASAGSAPL
PSRNARSSPPLLSPEHPEAVSEGDPDWHPEMAPCCANVVVTDVTTNLLTVTIKEFCRPPEFDKPASPSSSSSSSSSSS
ATLPPVTPSSPASSTPNPKLQP

>Sfo_ENSSFOG00015032962*/LOC108938841
MIVTMKSSVTPIERITFRSPCFSGRERGNCTGRRKEDLNLKTLFLRPERKLLRSVPPQGSRTRACATMLSTPPDPPPH
QGSLHRHLCLPLRPPPAPTPKLHSGAASHKLKKDIRRCHRMSRRPLPRPDPLAPPTSCSSSSRPPVSPFSETVRILNR
KVKPREVKRGRIILNLKVIDKSGKASSPAALHHPPSQRGRPPIPSRNRIIGKKFSDGAYRGLQPPLKLPGFPMYGKPF
GLQQVGPVPGQPPLGMRSGTGRSGSGTPAHTVSIPPRLSVASEGPGGVAALPVSQYQPPLSPSSSSGPDSTPASPTQI
QLPPLPAPASVSPPRLSPQGLPGPEEAPGLQSNPSPVAFLPSCPSPSSSPSTSEDEEAILDQTTARATKRKSRGRPVR
RRRRNKAAATTPVAGSSVSPGAREPYPNSSPPLLSPEAPRGAGEGDPNWRPEMAPCHANVVVTDVTTNLLTVTIKEFC
LPPDLDMSSPASPTSPSSPPAAPSSPIPPVSQLKP

>Sfo_LOC108938841/ ENSSFOG00015032962*
MELSAVGDRVFAAECILKRRVRKGRIEYLVKWKGWAIKYSTWEPEENILDSRLIAAFEQKERERELYGPKKRGPKPKT
FLLKARAQAAETTTRVPELRQYQPAASSSSRASSSSASPASSSHSVYPSSSSSSAPTAKLQSGAAHHKLKKDIHRCHR
MSRRPLPRPDPLAPPQGSSGSSFSSRPPISPFSETVRILNRKVKPREVKRGRIILNLKVIDKPGSSSAGATSRRTAIS
HQQDSAHSTHPGRSRIPSRNRIIGKSKRFGEVPYRGLQSPLKMPGFPMYGKPFGIQQSGSGPGPAQTGPGHYATGTSG

SSAAKGTSSGAPSSLTSAQAYPDRPTGQPQVLPRYQPLPSPSSSCGSDSSPSSPPQIQPTSLQAQPEPPSASAASPSP
PSLSPEPIELKQKQAPTPTSFLPAPPSIFLSSSTSSSEDEEILDLSVPHEGKRNARRRHHPRRRPSKHSPASAGSAPL
PSRNARSSPPLLSPEHPEAVSEGDPDWHPEMAPCCANVVVTDVTTNLLTVTIKEFCRPPEFDKPASP

>Sfo_Cbx7 (Gene ID: 108939814)
MELSAIGEQVFAVESIIKKRVRKGNVEYLLKWKGWPPKYSTWEPEEHILDPRLVLAYEEKEQKDRALGYRKRGPKPKR
LLLQNIYTMDLRSAHKGPEKPPARLRLSLTRSLTPEAGAEGPPCRTGGALYRRLAQRRNKQRASKLLRISPQPRPTTP
TPESLDRDWGDREDEEEEEEDEEEEAARQEAEREIHHDMLNGSDQAECWSSSVGAETETMTERSEGWSPGTGPGEVTV
TERPEGWSPVISPGEVTVTDVTINSVTVTFREALVAKGFFKSWELEF

>Sfo_Cbx8 (Gene ID: 108939914)
MELSAVGERVFAAESIIKRRIRRGRMEYLVKWKGWSPKYSTWEPEENILDSRLFAAFEERERERELFGPKKRGPKPKT
FLLRAQAKAKAKTYEFRSEAERGIRVSFPNPEPVITPRAREGLRTIVPMIFPPSTVNRGDSVRVRPVESLREHRPPML
HKPSPDEFVSVPKKRGPKPKLRFKDSLSSGPSPEHAKRRLEEQMAYVPVKMRKAERSEGEKMDLPLRMLKLARRHPED
PGYTQKQVFAMPGGSSQHFSAYRSSYRHRESSDLQSCRTKDCSGGVSSLHSKLNRMSKSSMCQPDVPSSPGQPSLIAK
IPMSRILGEPEEDSWSPCLNNIEKVVVTDITTNCVTVTIKESSTDKGFFKDKR

>Sfo_LOC108936948
MELSAVGERVFAAESIIKRRIRRGRMEYLVKWKGWSQKYSTWEPEENILDARLFAAFEERERERELFGPKKRGPKPET
FLLKAQAKAKAKTYEFRSDMTRGIRVSFPVPEPVVTPRAREGLRTVVPTLFPPSTVNRGESVRIRPPEPERGHHSSGP
LRPLVAEGFVSGPKKRGRKPKLRFIDGPNSSSPLVPTKRSRPEEQMTYHPAKMGKHGLAEDEKQDLGVIQLARKHKGD
PSYAQTQLRAQSSAGVLKYGSDTCYHRAGFEMQSCRTKELSCNISGQHPRLKHSSRNNLHQANGPPQREPPSLVGSEP
MSGDSGESPEDSWSPCPNKVEKVVVTDVTTNFLTVTIRESSTDKGFFKDKG

>Gmo_Cbx2 (ENSGMOG00000013005)
AMEGVTVGQVFDAECILSKRPRKGKFEYLVKWRGWSSKHNSWEPEDNILDPRLLAAFHKREQERELLFQKKGKRPRGR
PRKILVSPPKTAHAKKAKPGPRPLHPGPQKRPQILLAKPDPVPKKRGRKPLHPDLRALRQAKGSQQALRPHRDGPREE
SRSGVKKPLQPASFTYTGLSRGSREVAPPGSQGPAGSFTQSASSKPASLNSMWANRSLSNSSPSTTKPPPPPSDTGSG
RGEGLKTCPPALKHQVGGRRYPASHSGGFGPREASQRSLLGQRRQEGVAGQHQHNHLPRDRVTQALSLRALNLQSRSN
SRGKGSGGGGGREQGEASQTLKELSTGDSDESSSSGAERGSAAXXXXXXXXPGNRPSLGGVGGLDGETDWRPARTLLE
HVFVTDVTANFITVTVKESPTSVGFFNARK
H

>Gmo_ENSGMOG00000008628
MEELSAVGEQVYDAECILNKRLKKGKLEFLVKWRGWSSKDNSWEPQKNILDPRLLAAFNKKEQEKEQIMRKKGKRPRG
RPRKVVEINAEATKSSSSSSGSSSSSSDSSSCSSSSSSSSEDDDDDDDGAKQVSPGVRTRELHPGPQKKAQIVVAKQE
PPKKRGRKPQASPELRTFQPIKGLRKIASREPMAIEPPGAIRKPAHPAGFSFPGYEGFPQGTAGGANRGALSPAGPVR
PRGRAAQPASPSLNRSGQNRNSADAKLSVSVASGVPVLAGNKSKGVASLSVNAPKRAPQGSAQPPLGSPLRQRKPTPA
SPALQPANSFNGQANSLQPLNLQSG

>Gmo_Cbx4 (ENSGMOG00000011295)
MELPAAGEHVFAVEGIEKKRIRKGKIEYLVKWRGWSPKYNTWEPEENILDPRLLVAFQSRERQEQLMGYRKRGPKPKH
ILLQVPSFARRSNVPTGFQETSQDAKAAMTDVLQTSRTQPQQYQLNSKKHHQYQPNSQEAPPDQLSNGKKKLIYQLNS
KKHHHYEPDLNMYDAPASRLKEVVKIQEPAGKQANPGWNLPLALQQKWVRDKDTGCLSKVKELAVEVRKPAVNAKVAE
GKEEDGGSLKPHSKDAAAPTSGGGKMKIIKNKDKNGRIVIVMSKYMDGHKAHGSKGKQGEPSSEEKALHAKSSQSNPS
HKTKTGEPLHPENGVPKEICDGRSPPAADHPKKCCPKDRHFSKPSPSTAEEYNTEVARGQADLPDDLPMQLTASSPQT
SWTMDTNMLARTLHIRIPTFSADRKRKLSEPVEERCVSKVFQSSRSFSTPSVVDATPPQEKPMDLHCSGSRPRGASEG
DIGDMDSQEQPMDLSCPKTKTQHPEPKVQPEPEPEPVPEPIRVPDQKESYAELLKEETVTKISPFMGNIVITDITTNS
LTVTFKEYVSF

>Gmo_ENSGMOG00000003065
ELPAAGEHVFAVESIEKRRNRKGRVEYLVKWRGWSPKYNTWEPEENILDPRLLDAFKDREGQDQLMGYRKRGPKPKHL
LVQVPSFARRSSILSDLQEASLNEDSCQKTAPIHMLHSQSQQYQLNSKKHHQFLPLGKDAEQQANGKKKFYYQLNSKK
HHPYQPDLRMYEAPHLKPRDAKVPEVANQAWNLPPALQQKWVRDKDSGCLTKVKDITMELKKLPADLNGHKEPEKVQP
SAEKDNPSPPSSVSNGKLKIVKNKNKNGRIVIVMSKYMESGMKTAKIKNGDSKTVDEPTPDEESIDNEVEKMKLIKKL
GLMNGFAKKPKDKPKVSRAEHTETDPPLEAKAQVTEQDKQEEVGGQTPPLLDQPLQLTTPDRRADEAVPCLSESGRSH
EGLKRQSSAMDNEDQPGGSKRFLSSRISTPHTLSSPPQSAVEDQNVPGQYTNREYGYTEQEEPIDLSVVRTRPEAVIQ
TELCTETETVTQADTETLSEPFLGNIVITDITTNCLTVTFKEYVTA

>Gmo_ENSGMOG00000009785*
MELSAVGDRVFAAESILKRRVRKGSLEYLVKWKGWALKHSTWEPEENILDDRLLLGFEQKEREREMHGPKKRGPKPKS
PGSASPTPPSSSTPSAAAATSSSSASATSVTPAAKNSSLASTHKLKKDIHRCHRMSRRPLPRALAASSGPGPGGFPSR
PHASPFSETVRILNRRVKPREVKRGRII

>Gmo_Cbx7b (ENSGMOG00000013491)

MELSAIGEQVFAVESILKKRVKKGNVEYLLKWKGWPPKYSTWEPEQHILDQRLLQAFQEKEQRDRVVGHKRKGSKVKR
LLLENTIYTMDLRSAHKAPEKPQSRLRLSLTGSLDPGAEDQERLAHDERSQRSSSLTPSSEVFRTREVGLSHRPPRSL
EVTVTDVTINSLTVTFREALVAKGFFRAWG

>Gmo_Cbx8a (ENSGMOG00000011305)
MELSAVGESVFAAESITKRRIRRGRWEYLVKWKGWSQKYSTWEPEENILDARLFAAFEERERERELFGPKKRGPKPEN
FLLKAKAKIKPYEFRREPYRGIQVSYPVPEPVITPRAREGLRAVVPTIFPPSTVNRGESVGLHHTPHEPEKRSRPPLL
PFQEGAHVPKKRGRKPKKKVQSGEDLEAPVESAKRCRLEEQGFHGLSIPPRDLNHRRKAPDNGLLQLTRKFQEKTTIT
PRSIVQKQAVGLNRGLLIPEFGDQHKANHGSQGASDSSRTHFPQSKMKHLVKTRNHRSLEFSLARERSPVPVKPPESR
EGKSSNWGPWITKFDNVIVTDVTTNLLTVTIKESATDKGFFKNRR

>Gmo_ENSGMOG00000013071
MELSAVGERVFAAESIIKRRIRRGRMEYLVKWKGWSPKYSTWEPEENILDSRLFVAFEERERERELYGPKKRGPKPKT
FLVKAQTKVRSYEFRSEAVRGMRITYPSPEPTAPPRAREGLRAVVPTIFPPSTVNRGESVRVWPPSPSANTEDPRPGG
QAPEPKKRGPKPRLQFKISREGPTPAHSDPHKRRPEGGGGGGRLHLHLHPAGGAVTRGARGSAPGHDLRALPYGAPHR
RSTTRGRCWPGAPDRTTGAPGHRAPVHFKHKSKSNLSQLRTELSSAVKPAFLERLSPTPPAGDGERVKWRPSLANVEK
VLVTDVTSNFLTVTIKESSSSQGFFKDQR

>Ipu_Cbx2 (Gene ID: 108272989)
MEELSAVGEQVFDAECILSKRLRKGKLEYLVKWRGWSSKHNSWEPQENLLDPRLLAAFNKREQERELLIRKRGKRPRG
RPRKIMEPVQPVSKSSSSSSSSSSSDSSSSSSSSSSSSSSSSSEDDDDDQDDNDRKAKPGPRVRELHPVPQKKAQIVLA
KPDPPKKKKRGRKPLAPELRALKQNQAKNARKVMKPPLKDSLSDLRSNIKKPLMPASFTYTGLNRAANRETIAVQNRG
SFNANESPKNTLGSMQSQGKPVTEFKLSVSDMGNGGGLDLKNSSSKSPGVASLSLHNSKLTNSTNGQAAGGAPNWMKK
QETPALQRPVNIKPSVSPLSSMKIPGTQVSSLSGVNKTQLGVSASPKDVTSLRDSGNIVKKGPVQEKSALAEGGIMGR
AGSRDISTPAGVEKVKMEDPLDRLGKVTPGRQRKVPTFTGSKDGGKMTKPLSEMSTGEEGSSSESEQELPFTGKRQEL
SLEVQSECKDWRPTRSLIEHVFVTDVTANLVTVTVKESPTSVGFFSIRNY

>Ipu_Cbx4 (Gene ID: 108273333)
MELPAAGEQVFAVESIEKKRIRKGRFEYLVKWRGWSPKYNTWEPEENILDPRLLVAFQHREREEQLMGYRKRGPKPKH
FLTQVPSFALRSSVLSDLHDASQENGVHLKTSVDPTLMRQCQLQSRRQHPYQSLDNVLINGKNDQKQYYQLNSRKHHQ
YQPDPKLYQQVQAQEPDSGRHSDTKAVSMELEKVLDGGKEESTLSKESTRPVKGINSKLKIVENKNKNGRIVIVMSKY
LENGKEADGGVKQDEPSERGTNHANGTETNGLENGHHKDDRNGQVHRAVIEEFQNVETSQLMKKSGPISSENGIHKRR
HSEHSSIRRDAKSFLDCRSISAPNASSSQGRSELSDACQDEPMDLSFTGSRGQRNTIKDCRTNGHSNPTERETTPSTR
EPERAPSFTPFLGNIIITDVTTNCLTVTFKEYVPV

>Ipu_LOC108260285
MDLPAVGEHVFAVEGIEKKRIRKGRTEYLVKWRGWSPRYNTWEPEENILDPRLLVAFQNRERQEQQMGYRKRGPKPKH
LLIQLPAFARRSSILADLQETSLDEENQFKLDSLQMHRSQPQHYQLNSKKHHQYQPNGKEIPAEAHTNGKKKHFYQLN
SKKHHHYQPDPKMYDQQTTKPKEVKGHDPSIKGWNLPPALQQKWVRDKDTGSLNKVKDLATEHKQLLIPANKEDQMVK
TTSKDPLLSSGISSKMKIIRNKNKNGRIVIVMSKYMDNGVQPSKVKNRDSPGTEKPKQTEESINGNAANLVKVTAGQD
NGASIHSGDSACVTSEFVHKVPHKKFELAKAKLNTEAGHRTEQDMNKINLSHNHSLQLSTKTRSSPISAGEDTPSQKD
STNHHLSHRKRNLSEPNKPASDCKKFLSSRSISAPNPVIFSPQREPIDLHCSGQINSRGYSYNVTNSIPDEPIDLSCG
RTRYAEVPTESHVAVDNTPEASEKSEEPVSYFKPFSGNIIITDVTTNCLTVTFKEYVSV

>Ipu_LOC108260787
MELSASGDRVFAAEAILKRRVRKGRMEYLVKWKGWAIKYSTWEPEENILDDRLVAAFEQKEREQELYGPKKRGPKPKT
LLLKSRAQAAESSPRVLEFKPSRPQPSSKPPPPPPPASSYHPSGPSNAKLQSGAAQPKLKKDIHRCHRMARRPLPRPD
PLAPPIGSSGPFSSRPTVSPFCETVRILNRKVKPREVKKGRVILNLKVMDKTGPANNKRTQNSSHQSHLGRQKVPSRN
RVIGKSRRFGDVSFRCLQQPMGGAGFSVFRKPIEAYPMDPSEEKPKAEQSGHKPGTNPSPSSSQSSKVKNSAVPEALV
EPPRSASSSEVSDSEPHPPLPPQFHPNQLPQSGEANSQISHAAKSSPQLTDSKAKLTQSAMPSSPMFSSSSSSSSLSS
SSEENEHILDLSVPHGTDRRARQRQHFRGRRQPKVPEIPISEEASEEDQDLDWHPEMAAQCANVVITDVTTNLLTVTI
KEFCHPPGLPAVASSPCCANNLPSSKKTNP

>Ipu_LOC108259424
MELTAIGDRVFAAEAILKRRVRKGNIEYLVKWKGWALKHSTWEPEENILDDRLIAVFEQKERDQELYGPKKRGPKPKN
FVLKARAQAGETLSRGSSTRRAPPCTSSSSPVPPNPPSSSSLAPTPRLHSLAASHKLKKDIHRCHRMSRRPLPRPDPL
ANPDGSSFVSRLPISPFSETVRILNRRVKPREVKRGRIILNLKVIDKYGAGTTKGHQGRAHIPSRNRIIGKRQGEMPY
KPFQPPMKMPGFPMYGQPFGLQPCPPASASAGTRAGSAIGSKTRAHSSADSSRVRIQSPPSPSSSSGTESDAPSSRQI
HPRRKKAQLAAAATSSPPLALSTETSSQAHNRSTCTKSGSSAPRLPSSPPHSSSSSSLSSSPEDKKGRAQNLPFVARG
RKRKARRRFQTSNPSSGDGTEKTAPKPGDPDWHPKRTPTCANVVVTDVATNHLTVTIKEFCQSRAASGPPSPSHSENA
KSSTT

>Ipu_LOC108273000
MELSSIGEQVFAVESITKKRVRKGNVEYLLKWQGWPPKYSTWEPEDNILDPRLVLAFEEKEERDRAQAHRRKGLRPRR
LVLRNIYAMELRSAQKTSEKPAPRFRLSLARSMGAGLQHNARRCRNGEGGVYHRLLKRRSRQFVPKSLESAESPRQRT
LTLEKNAAEEDEQEKEWEKEEEQQEKKKRRKTENEEHHDIPHGPEMTEDHVLSTQETVITDGKPEHCASSPDCEVKPP
STEIGSATTAADQSASTTITNTYEHKPITDTSGVGVTQDQPTTDKSLNSSSGVGVITDRLQNRASVITVRFGSIDRSA

QDEEKETGETDSDAKRETDKDEVTAECTTTLQLSAEVRTDESQASECTRHPGKVIVTNVTINSLTVTFKEATAAEGFF
SSYGLQV

>Ipu_LOC108273945
MELSAIGEQVFAVESIIKKRVRKGNVEYLLKWKGWPPKYSTWEPEEHILDPRLVLAYEEKEHKDRAVGWRKRGPKPKR
LLVQGIYSMDLRSAHKAAEKSPGHLHLSLTHPLNPQAGHTAQKFCSKESDVYRQLVHHKRKKRNLKGYSVEDWEELEK
DDNYEEEVIRQEETETGRATLNSNVETERWSPITGSEEMTVSPVCEEWRPVMGLEEVTVTDVTINSLTVTFREALVAK
GFFRSWELEL

>Ipu_Cbx8 (Gene ID: 108272375)
MELSAVGERVFAAESIIKRRIRRGRMEYLVKWKGWSQKYSTWEPEENILDSRLFAAFEERERERELFGPKKRGPKLKT
FLMKAQAKAKARSYEFRGESSRSIRINYPNPEPLVTPRAREGLRSVVPTIFPPSTVNRGESVRPAHQELTQDHRPLLS
QRAESGRMGHIPKKRGRKPKLFREGYSSVLHSEKSKMDESMSGIPSKMARLELGEDDEDALSARSQKRQNPLLFPRRT
LEWTKTSDECRTREHASSHFHRKYLKHIRHHRAFKHGDTLAIKHQHHHHQHHPHHHHHHHHHHQPSLIAKIPMARILG
EPEEEAEQEEDEDAWRPCFSNVEKVTITDVTTNFLTVTIKESDTDEGFFKDKR

>Ipu_LOC108261002
MELSAVGERVFAAESIIKRRIRRGRMEYLVKWKGWSQKYSTWEPEENILDERLFAAFDERERERELYGPKKRGPKPET
FLLKAKAKAKSDEFRREMSREIRVSFPVAEPVVTPRAREGLRTVVPTIFPPSTVNRGESVRVRSPEPERRPRPPLSPT
HSLLDPVNTPKKRGPKPKLRFPVSAPSSSYSPTESLKRRADEHLSFSPAKMSRPAHHSGESSNCSLIQLTQRFQAESG
KSPKQLGCGSSGSVALGGTLRKTAHDMLRHGTKHFSSGPSVAHTKRKHLSKNSLFQPDDLPIDRLSPKYPVPQNPGDE
DEESWSPCLKNMEKITVTDITSNSLTVTIKESSTDQGFFKEQR

>Eel_LOC113568410
MEELSAVGEQVFDAECILNKRLRKGKLEYLVKWRGWSSKHNSWEPQENLLDPRLLAAFNKREQERELLLRKKGKRPRG
RPRKIMKTPTLSKTSSSSSSSSTSGSSSTSTSSSSSSEDDDDEDDDEDDDDDEVGGDRKRKPSPRVRELHPVPQKKAQ
IVVAKPGPPTKRRGRRPLAPELKAQRQIQAKGPRKLAKHTAKDSLAELRASIKKPLMPASFTYTDRVKAEEASSPADR
MGRAAQARPHKGPTPDTPRDRNGSKVGKAPSGTSTGEEGSSSESEQESPYPSDRQDVSVGVQTGPDWRPTRSLIEHVF
VTDVTANLVTVTVKESPTSVGFFSIRNY

>Eel_LOC113578871
MELPAAGEHVFAVESIEKKRIRKGRFEYLVKWRGWSPRYNTWEPEENILDPRLLVAFQNRERQEQMLGYRKRGPKPKQ
PFIQVPSFARRSSILSGLQEAPHDACLRMAMDPAQAQQYQLNSRKHHLYQPLPSESPGGTVPNGKKKHYYQLNSKKHH
HYQPDPKMYQMPCDRSKEPAVPQVGRDGWSLPPTLQQKWDEDSRCLGKAQDFTMELNGGGHEPELSAELPVKQDGAPA
NGISSKLKIVKNKNKNGRIVIVMSKYMENGIQAARVKGGEVDGGLKQSEQLPESKAEDVDNEKNKLPKKHGFESLGCG
KDSKGQDKPPLCGLMNRTTLPDSVMSGPVAAEVNKEQEFRNLEHSTHEDQPLQLTTKSCLSPKTSEKAVPSQLDQRNR
QGAHKRCSELDRDHGEAKRFVSVQSVSAPNTGTSQCQNCTTNLNGHQLTNGRKFEFQHSSQDEPIDLSCARSRKERNS
VMDCQINGNSEQCEKETEITVEETPFTPFLGNIIITDVTANCLTVTFKEYVTV

>Eel_LOC113573270
MDLPAVGEHVFAVEGIEKKRIRKGRIEYLVKWRGWSPKYNTWEPEENILDPRLLVAFQNRERQEQQIGYRKRGPKPKH
LLIQLPAFARRSSILAGLQETSLDDENHLKVDSLQMHRSQPQHYQLNSKKHHQYQPCSKEIPAEPHVNGKKKYFYQLN
SKKHHQYQPDPKMYEQQSTKPKEVKCHDLSINGWNLPTDLQQKRVQDKEPDCIHKEKELAIEHKLPVTNNKADQTVKT
SPKEPSGISSKMKIIRNKNKNGRIVIVMSKYMDNGVQPSKVKNVDSAIMEKPQQNEEILNGSTDNHVPVTTGQENGTL
KDHGVSSASALSEFVHKVPHRKFELLKAKPDIEVEHKTEDASLDKFGLSDDQPLQLTTKPSSSPLSAQTDMVSQRDFP
TYQRSHRKRNLSDPNESNSNCKRFLSSRSISGSNPALSSPESEPIDLHYSGHSTTRAYSYEFTDVIPEEPIDLSCGRT
RLQTEGPAEPHVTMEDIPETSEKFEEPVSCFNPFVGNIIITDVTTNCLTVTFKEYVSI

>Eel_LOC113569862
MELSVAGDRVFAAEAILKRRVRKGRIEYLVKWKGWAIKYSTWEPEENILDDRLVAAFEQKEREQELYGPKKRGPKPKT
LLLKSRAHAAEGSPRVPEFKHSRPQPSSKPPPQPPPLSPPSYPPSGPSNAKLQSGAAQPKLKKDIHRCHRISRRPLPR
LDPLAQQVGSSGPFSSRPTVSPFSETVRILNRKVKPREVKKGRVILNLKVIDKPGGGGVANNRRTPSATHQPHIGRQK
VPSRNRVIGKSRRFGDVSYRGLHLPATAAGFPVFGKPFECHPMEVSEEKPKVEQCVPKVACNSSSLPSSSQSPKVMTP
DIPKALSEPPCSPSSPEVSEGELLFPPPTHPQPPPSAAQPCQANLAILPPAKPNTQLTDCKPNSTPAQSTLPSSPMFS
SSSSSSSSEDSEHILDLSVPHSTERRCRRRNSFGPRRPAEFPENPLPHKVSEEDEDLDWRPEMAARCANVVVTDVTTN
LLTVTIKEFCHPPGLPPPVSSACFSNHLSASNKTNTRP

>Eel_LOC113585732
MELSAIGDRVFAAEAILKRRVRKGRIEYLVKWKGWALKHSTWEPEENILDDRLITAFERKEREQELYGPKKRGPKPKT
FVLKARAQAGEPSSRGSTTRQAPPRSSSSSVAPANPPSSSSSSSLAPTPRLHSLAASHKLKKDIHRCHRMSRRPLPRP
DLLANPDGSSFASRPPISPFSETVRILNRRVKPREVKRGRIILNLKVIDKYGSGGGGATHRRPAQSHQGRAQIPSRNR
IIGKRQGELPYRPFQPPMKMPGFPMYGQPFGLQPCPMVSVKTSRSAGPTTGSKGAGRNCGPGHSSAEQFQSPPSPSSS
SGSESDAPSLPQIQATPPKAPPDSAPSSPPVLSPETPSRATDDPQNCPSSAQPGSVSDPVPFLPSSPSLSSSSPSSSS
PEDDDERARNVPPAPKDGKWKPRRRTRASVSKSARQGDNSRPLAEKSAPERGDPDWRPERAPSCANVVVTDVATNHLT
VTIKEFCQPGAGSAGSVASAPASPSHSENLTSFTPSSA
>Eel_LOC113574891

MELSAIGEQVFAVESIIKKRVRKGNVEYLLKWKGWPPKYSTWEPAEHILDPRLVLAYEEKEHRDRAVGWRKRGPKPKR
LLVQNIYTMDLRSAHKAVEKSPAHLHLSLTGSLDPQTGHVDQKFQSKRDRVYQQLIQHKRKKRNSIDPSVEDWEGRED
ENEDEEEDGMRQEEAETDRATPTSNIRTEGWNPITGTEEVTECPLSDEWRPLMVPEEVTVTDVTLNSLTVTFREALVA
KGFFRSWELEF

>Eel_LOC113588928
MELSSIGEQVFAVESITKKRVRKGNVEYLLKWQGWPPKYSTWEPEDNILDPRLVLAFEEKEERDRAQAHRRKGLRPRR
LILRNIYTMELRSAHKAPEKPAPRIRLSLTRSVGAELEHSGRRCRIGEGRVKHQRLEKRKSRQCVSRHTADNTENPRQ
RTLTHKKDSTEEEWEEEKEQEKKKRKTEKDDEKTDEHRDIPSAQEKTEDSTFSAEQNVTAMANQTEHHSSTHDYGEDS
AKDSSDGTCSAPTVTDKSASDTITDTHEQGPITDGSEIRVSALELHDHTTTHQSTKTSSEAVLTAKEGSAIDRGQNRV
SVIAVRHLIDSCGRGQAKETAETECSTAKLKEETGEDEGTAEYMTTLQLSTEVRTNQSQASESSQHPGKVIVTEVTIN
SLTVTFKEAVSAEGFFSGCELQV

>Eel_Cbx8 (Gene ID: 113578829)
MELSAVGERVFAAESIIKRRIRRGRMEYLVKWKGWSPKYSTWEPEENILDSRLFAAFEERERERELFGPKKRGPKLKT
FLLKAQAKAKARSYEFRGESSRSMRVTYPSPEPLVTPRAREGLRAVVPTIFPPSTVNRGESVRLPLPEPAREYRPHPS
SRPSSDGFIPTPKKRGRKPKLRFPEGFPSSLHPDQARRRADESISGIPSKMARLGLGEDEEDGHSDLRAMKADHRHQT
SPLYPHKQHTTVPSMDWSLDSSRRNLDMQQCRTREHGSGLHRQHLKHLKHLSHQRALEPGVHAARQQRQPTLIAKIPV
ARIFGEPEEEDEEEEDKEDAWSPCFSNVEKVIITDVTTNLLTVTIKENSTDKGFFKDKR

>Eel_LOC113573271
MELSAVGERVFAAESIIKRRIRRGRMEYLVKWKGWSQKYSTWEPEENILDERLFAAFEERERERELYGPKKRGPKPET
FLLKAKAKAKAKSYEYRREMSREIRVSFPVAEPVVTPRAREGLRTVVPTIFPPSTVNRGESVRLRPPEPERRPRPPVS
SSRDFMDFDNVPKKRGPKPKLRFPVNTPIGCSSTEPVKRRADEHLCFSHSKMGRPAHLTEETSKCSAIQLTKTSTIQK
QNGSGSSGTVMLGGTLQKTGHDILQHETKDFSSGTSDHPKMKHLSKNMFRSDDLLDDLNSPKFPLSLMMLEDETEETW
TPCLTNMEKIVVTDITSNSLTVTIKESSTDKGFFKEKR

>Cha_Cbx2 (Gene ID: 105898869)
MEELSAVGEQVFDAECILNKRLRKGKVEYLVKWRGWSSKHNSWEPQENLLDPRLLAAFNKSEQEKEMLYRKKGKRPRG
RPRKIVETVPEDTKSSSSSSSSSSDSSSSSSSSSSSEDDDNEDESDRKARPAPRPRELHPVPQKKAQIVVAKQEPAKK
KRGRKALPPELRALRQGKPPRKIIKPTIKDSPTELRPNTIKKPLQPASFTYTGMARSANRDSLALHGRGAFSQSGAVK
PSLSSVSPGRSLSPTSPSFSRPPQGKGAPDFKLSVSESDSSARLDLKSSSSSSSSSPSCKSPGVASLNLHNSKLSANT
SNQGAPQSVLGSPNGQKKHNVSPGQTPLQRVPGNKTTAAAASSPSVKSPTNQAMNMQALNLQSANKPLQCAGTAGNGN
MAGGNFRGAANQTRKTLSGNSRQESSHQPATASSGPQQQAKKSQPGEDKIKAEDTTDLGPTPERSAKRPPNRVEKTPS
IKLPTEIREPVGIRERSSSKDGGKAKVLIEMSTGEEGSSSDSDQDSPFPSNGQDLSIAVQGSQDWKPTRSLIEHVFVT
DVTANLVTVTVKESPTSVGFFSMRNF

>Cha_Cbx4 (Gene ID: 105892293)
MELPAAGEHVFAVESIEKRRIRKGRFEYLVKWRGWSPKYNTWEPEENILDPRLLVAFQDRERQEQLTGYRKRGPKPKH
LLVQLPSFARRSSVLSGLQETSMDDVKLHKVEPVQTQQYQLNSKKHHQYQPHSKESHQAEQQVNSKKKFYYQLNSKKH
HHYQPDPKMYDSQFQKAKVPETSPPSTGWSLPAPLKQKWVQDKDSGCLSKVKDITMELKKLPASLDSGRVTEVNSCTK
EDPPPSGISGKLKIVKNKNKNGRIVIVMSKYMENGTQAARIKSGEGDADKLSPDHRVENSTEKKKAVQRQGSDHTHRK
DSKDKGHESHASGTINGQSTQKDSLADKSAAVETNESQRVKGGQDLSEDQPLQLTTKPRSSPWLLQNKVASCLDGAYT
EPPQKPSLSNKRRHSEHNEDQGGGKRFLSSRSISAPSTVTSPAQNHSMDLTGPQYPSNGHDFNFLGANPEEPIDLSCV
RSREERNEVAKIQTTTPEELEPMSTAAGLLPTSAPSSGPTSGATSAPSSGPTSGPTSAPTSVPASGPAQEDPAPAFRP
FLGNIIITDVTANCLTVTFKEYVTI

>Cha_LOC105891371
MDLPAVGEHVFAVEGIEKKRIRRGRIEYLVKWRGWSPKYNTWEPEENILDPRLLVAFQNRERHEQLLGYRKRGPKPKH
LLVQLPAFARRSSILANLQDSPQDEENQIKLDSVPLHRSQPQQYQLNSKKHHQYLPNCQEASAEPQIHGKKKYFYQLN
SKKHHHYQPDPKMYDVQFAKVKDTKPQGPPSWSLPPALQQKWVRDKDTGCLSKVKDLIVEPKKPSANTSQPEQRPNTS
PKEGSLPSAVSSKMKIIKNKNKNGRIIIVMSKYMDSNVTSTDLANGDPSRSPHQGREPETSSTDHYPEQAKVTSHINQ
QAKVTSHLNQENGIAEKDTRGHTLDNKKARSFKEHRFPKEKITVEEHCRMDTSEGQFNTKVDQPLQLTNKPTSASGAP
LEVSRCHSIPNDRKRTLSEPERGSDCKRFLCSRSTSAPGAMSSPYQSEPMNLHLGDQCMRNGSSEFTDTNPEEPIDLS
FWKSPKQTEPSVETPTSEQQSVETPKRPEEPVTHFKPFQGNIVITDITTNCLTVTFKEYVSI

>Cha_Cbx6 (Gene ID: 105912870)*
MHSLFSLNTTDGCILRHSTWEPEDNILDGRLVSAFEQKEREQEMYGPKKRGPKPKTLMLKARAQAGASSSRILPLRQQ
QQPPKSSPRPASSSSSSSFPSSSSSSSSLSNAKLQSGAAQPKLKKDIHRCHRMSRRPLSRPDPLSQPMGSSGPLTARP
PSFSETVRILNRKVKPREVKRGRIILNLKVVDKPAGAGKPPATQRPQVQQPQQPNPAHQPSQAGRQTVPSRNRIIGKS
RRFGEVPYRGLPAPLKVPTFPPPPRRTLGAHQTNSDPTRTQPGGTGAAGPSDPSALPTRPPPAGSDRTGPKAGPPPEH
LAPPSSEGPPSDPPSFLDPEDTHTAADSLSPPPSSSPSCSSSSDDGVQILDLSLTHDAERSRPDDHHHHRRCQNDFSS
EPHHHGNLSGQEGDPDWHPEMTVRCANVVVTDVTTNLLTVTIKEFCPPPGNATATSATATCPAASPQQAILPPQAEP

>Cha_LOC105903838
MELSAVGDRVFAAEAILKRRVRKGQIEYLVKWKGWALKNSTWEPEENILDDRLVTAFEQKERQQELYGPKKRGPKPKN
FVLKARAHAGETSSRATDPRLISRRSSSRPPSSRPPTSRPPSSTSTALVSAPASSSSSSSPAPSPKLNSLAATHKLKK
DIRRCHRMSRRPLPRPDPLAPSSGSGSFPSRPPVSPFSETVRILNRRVKPREVKRGRIILNLKGSCMDKAGGGGPAAR

RAAIPSRNRIIGKRFGDVPYRPFQPPMKMTGFPMYGKPYGMQRIGSDSFQANAGMASNTEASIGGTSAPASASTPHAV
PQYMSPPSPSSSSGSDSRPPTPRQSKLAAPHPPPRTRASPPKLRAPAAPQGSGTSQHQPTAAQSAAVPESAAFLPCSP
SLSSSSSSLEDEEGEDAANLSASQKVKRSLRRRRSQASQPTSASDNNTVSSPAEDATSAALPGETRAPVEGDPDWRPE
MAPRCANVVVTDVTTNLLTVTIKEFCQPAEAGSGPPSPSATSSSPPPPSQAELPSLPLPATSVAQP

>Cha_LOC105888811
MELSAIGEQVFAVESIIKKRIRKGHVEYLLKWKGWPPKYSTWEPSEHILDPRLVLVYEEKEQRDRALGYRKRGPKPKK
LLLQNVYAMDLRSAHKPLTQPPARLRLSLTRTLDPESPTSTDQHYQLAGGGLYDGLAQHKNKQRLAQYVCVPSHPQTP
TPSQGLSEEDWEEEEEEEDEDEEDGDNEDDDENGPEEAEGEMAMQGDALSRPGSKCWSPIAQPEEVTASQISEGWSPF
TSAKDITVTDVTINSLTVTFKEALTAKGFFRSWGLEF

>Cha_LOC105897974
MELSSLGEQVFAVESITKKRVRKGNVEYLLKWQGWPQKYSTWEPEDHILDPRLVLEYEEREEKDRALAYRRKGLRPRR
LVLRNLYPMDLRSATKVPEKPPPPRLCLSLTRAMGAELDQGVRACRAGVLRRLEKRRSKQGSVRPLLRAARPPPPPPP
TEKPPPHHTEEDDCGGGKGDHVMEQPDRREIVLQDDDNKTEQDDNKTDQDKDIPSAPQYSDGYCSSTEQDMALMSEMA
AEEDSGSCRGPREESVVECETPPVAGGTGLMKNDTGRRTQGDHPPLNTTPNGLGGSDSPSEETPDVVPMTDDESQSSS
TTACEADATHISLDTVHSCHREPTDSSTDTCIKAEAEYKLVVVSKQDVAIAEKGSEGSTTSGKVIVTDVTINSLTVTF
KEALKAEGFFKGCGME

>Cha_Cbx8 (Gene ID: 105892295)
MELSAVGERVFAAESIIKRRIRRGRMEYLVKWKGWSPKYSTWEPEENILDSRLFVAFDEREREELYGPKKRGPKPKT
FLLRAQAKEKGKTYEFRSEANRGIHVTYPSPEPVVTPRAREGLRAVVPTIFPPSTVNRGESVRVRQPEPAHERRLPPA
LSRPTGDGFAPIPKKRGPKPKLRFKDGLLTGPPREQRLKDGLLTGSPREHRFKDGPLTGPPREQRLKDGLLPGPPREQ
RLNDGLLTGPPRELAKRRAEEPPVYGASKLAKHALADGQDRDSEMRVIKLAHRPLQGPPSYPPKPPTRLMPAGGSRQH
CPTGRSSLHSHRPGLDAHGSRTTDCLVRQTPTHLKHLAHSSLQREDAPAQRQPTLIAKIPISHLLDGPEKDARWNPCL
TNLEKVVVTDVTTNFLTVTIKESSTDQGFFKGKR

>Cha_LOC116220815
MELSAVGERVFAAESIIKRRIRRGRMEYLVKWKGWSQKYSTWEPEENILDERLFAAFEEREREELYGPKKRGPKPET
FLMKARAKAKGKSYEFRREMSRGMHVSYPVAEPVITPRAREGLRTVVPTIFPPSTVNRGESVRPQELERRPRPTGSPM
RVTDSFVSTPKKRGPKPKLRFNVNASSCAYTEVSKRRSDEQIAYGTSKMGRYGHREGEMSDCSVTQMTERFKPNFKKT
MVAATSGYHLIQASALSKPRQDIQGHRTVKECSSETLQQSKGHNPSRPHSYEDDELETEQSGSKQPVPSIFGDQSNES
WRPSISNMEKVVVTDVTSNFLTVTIKESTTDKGFFREK

>Dcl_Cbx2 (Gene ID: 114794490)
MEELSAVGEQVFDAECILNKRLRKGKLEYLVKWRGWSSKHNSWEPQENLLDPRLLAAFNKREQEKELLFRKRGKRPRG
RPRKILDIVPTGTKSSSSSSSSSTSGSSSTSSSSSEEDDDDDDDDDDDENDRKPKPGPRPRELHPVPQKKAQIVMAK
QEPIRKKRGRKALPPELKALRQGKVPRKMVARDTLVDFRGAIKKPLQPASFTYTGFGRNSSRDSIGIHNRGSFTSGGL
KPQFSTLGSCRSVGSTSQSFSRPVQSKSSPDFKLSVSESGNGGGGGSGLDMNSSACKSPGVAALNLHNSKLSATTNQG
HQQPQAAMSSASGGQKRHEAPHGQTPLQRMNSSKPVGSFSLAKSPTSQAMNMQALNLQSVNRPTGNGGTPVANQRSANN
PMRKNDHLRQDLSPAPNPGTLQSKKSQPGGDKIKAEDTADFCSTGERLAKKPQGRVEKNTMLNTSTEIPGSRDRSSSK
DGAKGKSLSEMSTGEEGSSSDSEQDLPFSGDRQDLSISVPATQDWKPTPSLIEHVFVTDVTANLVTVTVKESPTSVGF
FNIRNY

>Dcl_Cbx4 (Gene ID: 114786797)
MELPAVGEHVFAVESIEKRRIRKGRLEYLVKWRGWSPKYNTWEPEENILDPRLLVAFQNREREQEQLTGYRKRGPKPKH
LLVQLPSFARRSSVLSGLQETSLEDDKWHKATPIQPLCPPGQQYQLSSKKHHPYQPHGKEGPAEQAAGGKKKFYYQLN
SKKHHHYQPDPKMYDAQRPKAKEAKVPETLPATLWKLPSALQQKWTRDKDSGCLSEVKDITMELKKLPACLEGSGEPE
VSAGAKHDSAPNGISGKLKIVKNKNKNGRIVIVMSKYMENGTQAARIKSGEAADKAGQGAAAAENGPADSRKRDSVPP
CGNTAGPEGTSTSGPVAGQKDQSAGVRGREELPSDRPLQLTAKLSPAPPTSGPLSHLDQQKTTGLPSKRRSSEPHEDQ
GGVKRFLSSRSMSAPGAVAPPPSAGDADLYGLSDSNPEEPIDLSYVRTPVPKDGRNGVSLAQTNTPAEPAAPAPTAEA
AGRESFPEFKPFLGNIIITDVTANCLTVTFKEYVTV

>Dcl_LOC114793848(a)
MELPAAGEHVFAVEGIERKRVRKGGAEYLVKWTGWPPKFNTWEPEENILDRRLLVAFENREREQEQLLNCHKRGARPKD
LLLQLPSFARRSSFPEGHPQAYQLNGKRHHHKPKPRLSAEPRVHSASRQDRDLESSAMHPKRVQDGPPSFLREVNNFAR
EMNEPNANNSSSGREQPPPNPINSKMKIIKNKNQNGRIIIVMSKDVDGSVPSSEEKNRHSSGTRRRAEQTDGSGGMIP
VRNSCGKVLLAEDVTRRRDPAQPDESVQLNSSRARLTPKRHERQLLNPQANGSALPSKPTGLQGGGPDSRNGSEHDPA
DAHPDQPINLSLADAEQRARPPAARATEQKRQKLLSEAGEPASYFKPFNGNIVITDVTANCLTVTFKEYVSDYMDVDP
ERVEYYGLRGAGVN

>Dcl_LOC114786320
MELSAVGDRVFAAEAILKRRVRKGRIEYLVKWKGWALKYSTWEPEENILDDRLITGFERKEREQELYGPKKRGPKPKN
FILKARAQTGDTSNRFSVARQTHNRSAATRPSSSSASSVLAPHTSTSSASSSLAPTAKLNSLAATHKLKKDIRRCHRM
SRRPLPRPDPVGPSNNTSFSSRPPVSPFSETVRILNRRVKPREVKRGRIILNLKVIDKPGSGAGTSSRKVPTSHLGRA
TIPSRNRIIGRKCGNMPYRPFQPPVKMPGFPMYGKPFGLQQMNPVPFQTHIRFGCNTNARGAHSTSASATTGAPQALQ
HYQPPPSPSSSGSSESQPPSPPSIQVSAPSAVAGAPTSTAKLRSQTQPQPGDPQFQPGSTQASTAPVPFLPASPSLSS

SQSSSSFEDDEDEGRGGLSTQSKGKRRGRHCHHHQSSDCHATLVIANASQPLTQAIDRNAYQPSTEQARAPVEGDPDW
RPDMAPCCANVVVTDVTTNLLTVTIKEFCQPTETGSMPPSPSINSTPPSVSTTSVATESKP

>Dcl_Cbx7 (Gene ID: 114795196)
MELSAIGEQVFAVESIIKKRIRKGHVEYLLKWKGWPPKYSTWEPEEHILDPRLVLAYEEKEHRERVPGCRKRGPKPKR
LFLQNFYTMDLRTKHRAPALSLTPPSADLFPPAGGALYQLGSGQPRRAQFVHRPGTGPSEDNEEEGGMRGHVLGGEGG
PETWASISQPAQGTWHPQGAPRDVTVTDITINSLTVTFREAVAARGFFSRWDLDL

>Dcl_LOC114793357
MELSSIGEQVFAVESITKKRVRKGNVEYLLKWEGWPPKYSTWEPEEHILDPRLVLAYEEKEERERALAYRRRGLRPRA
VLLRNIYPMDLRSAHKAPEKPAPRLRLSLARSMASCRTGARGGALHRQDRGKSRQRAARTMESDPSRLKVPTLMQDSM
EDDDSGEEPCREENVLEENVAEKDIPCGVERSDGYPSSPELEMVPDAGRRPQGGATDAEQDRTSDRTEGSCDVVTDGA
GDAQTGEAGSSEPHAATEANRGPDVGSGGVESSGKVTVTDVTINSLTVTFREALSAEGFFKSWGLEI

>Dcl_Cbx8 (Gene ID: 114786798)
MELSAVGERVFAAESIIKRRIRRGRMEYLVKWKGWSPKYSTWEPEENILDSRLFAAFEERERERELFGPKKRGPKPKT
FLLRAQAKEKAKTYEFRSEAARGLRVTYPSPEPVATPRAREGLRAVVPTIFPPSTVNRGESVRVQALEPPRDHRSPPA
APRPIGDDFVHVPKKRGPKPKLRFQDTLSSDSSRPELAKRRADEPSLYGPPKISKHGMLGVERGSELRLSNLAHRHHH
HHHHHHHHRRHHHHHQEERCGAGYQAKLMRALHAHPPPAPFQGYRTKQCPGHQAPPRGSTYHLGHYPPAARPAPSLVA
RIPVSRILGEPDQDAWSPSLSNIEKVVVTDVTTNFLTVTIKESSTDQGFFKDKR

>Dcl_LOC114793848(b)
MELSAVGERVFAAESIIKRRIRRGRLEYLVKWKGWSQKYSTWEPEENILDERLFAAFEERWVVRGPRSNSPARRSRLG
FIRRERERELYGPKKRGPKPETFLLKAKAKAKSYGIRSELPRGIHVSFPVAEPVATPRAREGLRAVVPTIFPPSAVNR
GQCARAPADDPARAPRKRGPKPRLRLRLGAATPPCTGAAVQRFALDPRRPGLEVQIQRVQVQDSWRPSFTDVDQVVVT
DVTSNFLTVTIKESSTDKGFFKEKR

>Elu_Cbx2 (Gene ID: 105022926)
MEELSAVGEQVFDAECILNKRLRKGKLEFLVKWRGWSSKHNSWEPQENILDPRLLAAFTKSEQEKEILNGKRGKRPRG
RPRKVVETVPEVSKSSSSSSSSSSSSGSSSSSSSSSSSSSDDDDDTDEERKAKPGPRTRELHPVPQKKAQIIVAKPEP
PRKKRGRRALPAEMKAIQQNKGQRKIIKTVSKDSPSDSRVGIKKPLHPASFTFMGLNSRGPLGAPGRSSLPHGGATKS
LLSTTGAGRSNTSPSVNQRSHQSKSQASDFKLSVSDVGSGSGLDLKATLSKLPGVAALNLHNPKHTTSSGQPPLGSPN
GPKKPDASRQTALNRVATNPPALPFPGPKGPSNQAANLQALNPQGVNPSAQGSSAPDSGAMPVSNLRSAANPARKAVT
GQCGQEPSPVQCQATPGGQQPRKNKPGVDKLKLDETGEVGAAAERQSTTRAQGRAEKGGVQNPPSEARVMEGKRERST
SKDGGKQAKVLSEMSTGEEESTSDSDQDSPYPSNGRDLSISVQAGQDWKPTRSLIEHVFVTDVTANLVTVTVKESPTS
VGFFNIRNY

>Elu_LOC105027558
MDWRAVFTALEEVCEENISVLSAPADLPYGDPSRRLVTCMGKILEHGRALEPVVSGITAVYHHYDFDSDTPGNGYRTL
VKVLQSCLQHIIQKGRYVASNCGRALFRADHNASEMEAYGSVLCQLRALLYTAQRLLQETSPGELYGEPGGDLSRGLV
REYTSMYKACFYGRCLGFQFSPSLRPILQSIVISMVSFGESYDKQHSGLGMAAFSLLTSGKYVIDPELRGEEYERITQ
NLDIRFWKSFWNLTESELLSGLTSLGSNLVQVNLTLTIPPEPLFLPLASDPRLSTSVPPPLAHLGPGPVHMRLISHEL
REGQVILHLSLSVVEAAPISVSLVPFGAQKLPPSPWLLIHFHGGGFVAQTSKSHENYLKGWSKDLNVPILSVDYSLAP
DAPFPRALEECFYSYCWALKNCRLLGSTAERVCLAGDSAGGNLCITVSMRALSYGVRVPDGIMAAYPATLLTTDASPS
RLLCSIDLLLPLGVLSKCINAYTGEGSEWVQPTEGTSTLRALGRDTVLLISQLTQGASSWVQSFFPVEVVGPSSSYRR
SLGNLAHQRAVLPPSSDSSQGPMNYPEGFKPLRSKSLAVIQAPCCPVAKNPFVSPLLAPEDLLRGLPPVHLVASALDA
*LLDDSVMFAKKLRDMGQPVTLVVVE*DLPHGFLSLSGLAKEIQVASDICVERIRKGKFEYLVKWRGWSSKHNSWEPEEN
ILDPRLLAAFHKREQERELLFRKRGKRPRGRPRKVLEPEPNETKSDRSSSSSSDLSSSPSCSSSEEEEDEDHGKKAKP
GPQLRNLHPMPLKRPQIVVAKQEPVRKKRGRKPLLPELRALRQAKTRSPLLPPPPSRHHQVLRPPREPSREEPRGGVK
KPLQPASFTYTGLSSSRTSRDEEVPQEVAGGAFFQAGTSKPGQLNSMWSGRSMSSTTTTHSSPPSSSSLGRLHSKGLS
DLKRSISASDIGGTRAEGLKVSSSLKQGGSTSHLHSSKTSSGFGGGLAACSPTQRSPAGPRRQQEGYGGQGALAVQPQ
RSGGTKPASPPPSSVRDRVSQALSLRALNLQSVNKTTPGNGVHGNGTSGVTGVAVSRSSLRSSAGPAVGSIKETHPLS
GLNQNLAPGGAQHLGSVPSAREDRGGRGKSMGAGGVGRGNGKVEKGAAGGVSSARVGGAGVVRPEDRMSGQGLNERNS
RTLNELSTGDSDDTSSSESEEHDSSPYNENTNGPRLVSLEMETETDWRPARSLLEHVFVTDVTANFVTVTVKESPTSV
GFFNVRDQ

>Elu_Cbx4 (Gene ID: 105021558)
MELPAAGEHVFAVESIEKKRNRKGRIEYLVKWRGWSPKYNTWEPEENILDPRLLDAFQNRERYEQLMGYRKRGPKPKH
LMGQVPSFARRSSILLGLQETSLDEDNRQKMDPIQTLRSQTQQYQLNSKKHHQYQPLLAKEREAEQQAIGKKKFYYQL
NSKKHHHYQPDPKMYDNTQCLKPREVTKAPELPINRDWNLPPALQQKWVRDKESGCLSKVKDITMELKKLPAHLNGHS
VSELIEDPTKEDAAPVKANGDSKLKIVKNKNNNGRIVIVMSKYMENGTQAARIKNGDLEELDQLQPDKCEDAAEKHLE
KMRLIQKLGLTNGFAKVHKDVSKTHVPHCNRSVGFKCPDEKVDSPETEISVTKQEKHLDVRGLKQATKDEPLQLTTKP
NLSPWPFEEARKRTRPETAYPPALKRHLSEVVEDRGCYKRFLSSRSISAPSTVSSPPQSNSTDQNGHESHNDFLESNQ
DEPMDLSCVRSRVEALVPIDTQVTPIAEKKAAEQIEQPQAIATEQAAEEIEQPLAIATEQAVESQVFPSFKPFLGNIV
ITDITTNCLTVTFKEYVTV

>Elu_LOC105013028

MELPAAGEHVFAVEGIEKKRIRKGKMEYLVKWRGWSPKYNTWEPEENILDPRLLVAFQNRERQDQLIGYRKRGPKPKH
LLVQLPSFARRSSIPAGFEEPSSEAENCLRSDPIQMHRSQPQQYQLNSKKHHQYQPSNQEVPADQLANGKRKFFYQLN
SKKHHHYQPDPKMYDAQSARPKEVVKAQEPSPPIIPNPGWNLPPALQQKWVRDKDTGCLSKVKDVAVAEMKEPAVRVK
EVESERALKPSPKEAALPSAVSSKMKIIKNKNKNGRIVIVMSKYMDSNKAGAAKGKHSLDLSGEEKLQHTKKVQDTTS
VPKEIFNGSSPPAAEHPKKLSPEDGHFSKPSPSTAEEYNTEVARGQADLPDDHPLQLTTNPNPTPWVIDTPTTTSSPM
DQIRALATQNNDRKRKLSDPKEDRGACKRFLSSRSISVPIAVPIPPQDKPMDLHLIVPHQSSNSGYKVMDNSTSQEEP
IDLSCSRNKRQSEPEIVTVAEPEPESKGPTSVVEELERTTIQTASSAKTTTEKAVEEPVLTFSPFMGNIVITDITTNC
LTVTFKEYVSV

>Elu_ENSELUG00000020955
MELSAVGERVFAAEAILKRRVRKGRLEYLVKWKGWALKHSTWEPEENILDDRLIAGFEQKERERELHGPKKRGPKPKN
LTAKARREATARAPRSTSSSFPRAPPSSPYPSLPSSSSSAPAPSPKLNSLAATHKLKKDIRRCTSMSRRPLPRPDPSA
APGTFSGPGGFSSRPPVSPFSETVRILNRRVKPREVKRGRIILNLKVIDKPGSGGGGRGGANRRAPSSSVSFPGGRQN
LPSRNRIIGKRGGEAPYRPFQPMLGFPMYGKPFGLQCGSPGPLHISQPTTGPSTAAPGAAGESNTQSAHSSVGSCYQP
PPSPSRSSGSEAIPAQNELVPAGHQAQPAPGASASGSVPGTGPHVLASAFSVQSSSQPPEVQKNTNQKPNVLSSSPSR
SSGSTSSSFEEDDDYDGDALDLSATQDGKRKPHRCGRQRRQPPSESTSMPLPSTASTPLTSDPKRVPAEGDPDWHPEM
AASSANVVVTDVTSNLLTVTIKEFPSPSSQPPSPSSTSAASPKPPHPEESTSSSAPAATTKETPQAKS

>Elu_Cbx7 (Gene ID: 105025077)
MELSAIGEQVFAVESIIKKRVRKGNVEYLLKWKGWPPKYSTWEPEEHILDQRLVQAYEEKEQKDRALGYRKRGTKGKR
LLVQNTIYTMDLRSAHKTPEKPPARLRLSLTRSLDSEAEDPTYGACRRSVHPRMVHRKNKPRRSQFVCLASPAGSPPP
TQDPTHEDWGSREEECDEDMEEEEEAGSQQEEFERDTEICSSILNGQDRAEDWSSVIGSEEVTGSERSDVWSPVIGPG
EVTVTDVTINSLTVTFREALVAKGFFRGWGLEF

>Elu_LOC105025793
MELSSLGEQVFAVESITKKRVRKGNVEYLLKWQGWPPKYSTWEPEDHILDPRLVLAYEEKEKKERALAYRRKGLRPRR
LILRNIYAMDLRSSHKVPDKPTPRLRLSLTRSVGPELGKGSQPYRAGERRSIYRRFTGCKKQRVSKPVPDKNSPQPLT
HVDHEWEGAEEEEMESETHEDSLFGRSMSECSSSLMDQDMDDSTDRVESCSNSSLCSGDQTLGGRALLLLGTGEVVVT
GGPEGRTSENEQKLMDVSNQSECCYSPEGPKHLDVSNRLVNSTAEIKLRTDSMIDRVAVITQAPTDGGQRLSVEGRHE
ATAADLLVCSDVKKAVDRDRNEQRDGVLIQRPDRDDAEVLPGKVIVTKVTVNSLTVVFQEASDAEGFFRG

>Elu_Cbx8 (Gene ID: 105021557)
MELSAVGERVFAAESIIKRRIRRGRMEYLVKWKGWSPKFSTWEPEENILDSRLFVAFEERERERELFGPKKRGPKPKT
FLLKAQAKAKAKSYEFRSEAVRGIRITYPTPEPIVTPRAREGLRAVVPTIFPPSTVNRGESVRVRPPEPVREHRPLAL
QRPQGPDGFLSGPKKRGPKPKLRFKDNPSNALPTIPEPPKRRAEEQATYSPLKLAKLGLSDGDKRGSDMRVKSAHRHQ
ERLAGHSHRQMRPVPSGSTQQHYSQNRGMLQSQRIGSDAQGCRTTECPSSYVSSPHLKHLSKKNLHQPNEELAQMEKP
SLIAKIPVSRILGEPDEVTWRPCLTNVEKVLVTDVTTNFLTVTIRESSTDQGFFKDKR

>Elu_LOC105013027
MELSAVGERVFAAESIIKRRIRRGRMEYLVKWKGWSQKYSTWEPEDNILDARLLAGFEERERERERFFGPKKRGPKPET
FLLKAQNKAKTYECRRQMPRAIRVSYLVPEPVNTPRAREGLRAVVPTIFPPSTVNRGDSVMFRQPEPARRPRSAPTPS
FVSEEFGNVPKKRGPKAKLFNIDQDSCPTVDPAKRGRFEEQQPPYVLNKISRHCHHKGETSERSLSQLTRRFQEETNF
SQKSNDAKSPLPIESYRRTHNHLGKVARKARLDHQSPRTKDCIGGVTITPPKLKYMSKNNLYEVIASPSMERSMTTVL
DQTTLSRRCEEPSHTSWRPRTGDVDKVVVTDVTSNFLTVTIKENSTDKGFFKDKR

>Ssa_LOC106612095
MEGVTVGQVFDAECILNKRPRKGKFEYLVKWRGWSSKHNSWEPEENILDPRLMAAFHKREQERELLFGKRGKRPRGRP
RKILEPEPTETKSDRSSSSSSSGLSSSPSASSSEEEDEDHGKKAKPGPRRRNLNLHPVLQTRPHIVVANQEPIRKKRG
RKPLLPELRALRQANTRPPLPPPPLSRHHQVLRPPRELPKEEPRGGVKKPLQPASFTYTSLSASRGSRDETAPQRAAG
GAFFQAGASKPGLLNSIWPGRSMSTITPSSPSSSSSLSRPPPPHSKGLSDLKRSLSDASNSSGRAEGLKVASSVKPGV
STSTSLGLHSSKSSSGFSGSPAACSPAQRSPAGQRRQHEGPGSQAVVVVQQQGAKPSSSPPPSARDRISQALSLRALN
LQSVKKRVPGNGLQGYGTSGTTGVAVSRSSLRSGASQAGKGAGGSIKEMHTLSGLDQSLASGGLGAGVLRSGSIPQAR
EERGERGKTTGAETEREMDSKGPRGVGSGNGRLERGGSVQGGVSLAGGGGSSAAREEDRRSEQSLNERKSRSVENSRT
LNELSTGDSDDTNSSESEEPDSSPYPNNNGPRLVSMETEMETDWRPALSLLEHVFVTDVTANFVTVTVKESPTSVGFF
NVRNP

>Ssa_LOC106586561*
MWKAASEQQPFPLPCLIDGQPAYTVRCLMKVRPLGRGFQYLVDWKGYGLEEKCWVPARDILDPGLIAEFQHRHPGHPV
LVS

>Ssa_LOC106606958
MEELSAVGEQVFDAECILNKRLRKGKLEFLVKWRGWSSKHNSWEPQANILDPRLLAAFNKSEQEKEILICKRGKRPRG
RPRKVVETVPEVSKSSSSSSSSSSGSSSSSSSSSSSSDDDDDDNNDRKAKPGPRTRELHPVPQKKAQIVVAKPEPPR
KKRGRKALPAEMKAIQQNKGQRKIIKTVAKDSPADLRGGIKKPFHPASFTFMGLNSRGPLSVQGRCSLAQGGTTKNSM
NTAGSGRSNNSASLSFNQRSNQSKSQASDFKLSVSDVDSGAGLDLKTTASKSPGVAALNLHNSKLSTSNGNPQGAFQP
QLGSHNGQKKPDAPGQTPLQQVPNNKPAAPFSTPKGPANQAASLQALNLQSVNKSTPGNDTPGNGTAPVSNLRSTTNP
ARKDTVGQYGQEKNPVQSPATPGGQQPRKNHPGVDKVKAEETSEVGVMAERPERLTTTRAQGRVEKSIVQNPSVEARD

ILGKRERSASKDSGKQAKVLLSEMSTGEESTSDSDQDSPYPSNSQDLSISVQTGQDWKPTHSLIEHVFVTDVTANLVT
VTVKESPTSVGFFNIHNY

>Ssa_LOC106606946
MEELSAVGEQVFDAECILNKRLRKGKLEFLVKWRGWSSKHNSWEPQANILDPRLLAAFNKSEQEKEILICKRGKRPRG
RPRKVVETVPEVSKSSSSSSSSSSSGSSSSSSSSSSSSDDDDDDNNDRKAKPGPRTRELHPVPQKKAQIVVAKPEPPR
KKRGRKALPAEMKAIQQNKGQRKIIKTVAKESPADLRGGIKKPFHPASFTFMGLNSRGPLSVQGRCSLAQGGTTKSSM
NTAGSGRSNTSASLSFNQRCNQSKSQASDFKLSVSDVYSGAGLDLKTTASKSPGVAALNLHNSKLSTSNGNPQGALQP
QLGSHNGQKKPDAPGQTPLQQVPNNKPAAPFSTPKGPANQAASLQALNLQSVNKSTPGNGTPGNGTAPVSNLRSTANP
VRKDTVGQYGQEKNPVQSPATPGGQQPRKSHPGIKVKAEETSEVGVMAERPERLTTTRAQGRVEKSIVQNPSVEARDI
LGKRERSASKDSGKQAKVLLSEMSTGEESTSDSDQDSPYPSNSQDLSISVQTGQDWKPTHSLIEHVFVTDVTANLVTV
TVKESPTSVGFFNIHNY

>Ssa_LOC106589865
MEGVTVGHVFDAECILNKRPRKGKFEYLVKWRGWSSKHNSWEPEENILDPRLLAAFHKRAQERELLFRKRGKRPRGRP
RKILEPEPTETKSDRSSSSSSSGLSSSPSSSSSEEEEEDHGKKAKPGPRLRNLYPVPQKRPQIVVAKNEPVRKKRGRK
PLLPELRALRQAKTRPPLPPPSPSRHHQVLRPPREPFKEEPRGGVKKPLQPASFTYTGLSSSRGSRDEVAHQVAAGGA
FYQAGASKPGPLNSIWSGRSMSTNTPTPSSPSSSSSSLSRAPPPYSKGWSDLKRSLSVSDTGSSNGRAEGLKVASSLK
PGVSTSTSLCLHSSKTSSGFRDSPTACSPAQRFPAGQRRQQEGPGGQAGMVVQQQGAKPPSSTPPSARDRISQALSLR
ALNLQSVKKIAPGNGLQGNGTSGTTGVAVSRLSLRSSASPAGKRAGGSIKETHTSSGLNQGLVSGGLGGGALHSGSVP
PARVERGERGKDTGAETEREMDNKGPGGVGRGNGRVEKGGSVQAQGGVSTVRRGRANGGRQEDRKSGQSLTINERNSR
SGDNSRTLNELSTGDSDDTSSSESEECDSSPYPDNNNNNNGARLVSMEMETETDWRPARSLLEHVFVTDVTANFVTVT
VKESPTSVGFFNVRNH

>Ssa_LOC106606687
MELPAAGEHVFAVEGIEKKRIRKGKIEYLVKWRGWSPKYNTWEPEENILDPRLLVAFQNRERQEQLMGYRKRGPKPKH
LLVQLPSFARRSSIPAGFEETSPEAENHLRSDPIQMHRSQPQQYQLNSKKHHQYQPSKQEVSADQLGNGKKKLFYQLN
SKKHHHYQPDPNMYNTQSTRPKDVVKGQEPSPPTNPNPGWNLPPALQQKWVRDKNTGCLSKVKDMTVVEMKKAAVCVN
EAESECALKPSPKEAALPSAVSSKMKIIKNKNKNGRIVIVMSKYMDGNKAGAAKGKHSSDLTGEEKPQHAEQSDMTKV
QGTTNVPKEICNASSPPAAEHPKKRSLEDGHFSKPSPSTAEEYNTEVARGQADLPDDHPLQLTTNPTPSPWVMDTSLP
TPSPMDQIRTLPTTNNDRKHKLSHPEEDRGACKKFLSSRSISVPSAVPTPPQDKPMDLHLIVPRRSSSSGYGYEVTDT
STSQEEPMDLSCSRTRKQPEPEPVPEPEPEPKDPTAVVQESETTTMTTTTATTTTTEEAEEEPVLKFSPFMGNIVITD
ITTNCLTVTFKEYVSV

>Ssa_LOC106588430
MELPAAGEHVFAVESIEKKRNRKGRMEYLVKWRGWSPKYNTWEPEENILDPRLLDAFQNRERYEQLMGYRKRGPKPKH
LMGQVPSFARRSSVLSGLQETTLDEDNRQKVDPIQTVRSQTQQYQLNSKKHHQYQPLLAKERDAEQQANGKKKLYYQL
NSKKHHHYQPDPKMYDSTQCMKPREATKAPELPTNHSWNLPPALQQKWVRDKDSGCLSKVKDITMELKKLPAHLNGHS
GESEQSKATDKEDAPLLKANGVSDSKLKIVKNKNKNGRIVIVMSKYMENGMQAARIKNGDVETLDEQPLMNLDEQPDN
NDDAAENQLEKMRLVQKLGLTNGFAKDYNKGSNRSNVFKCPADKVDSPKMELSATEQNKHFNVRGQKEATEDQPLQLT
TKPNLAPWPFEMGVHRTQPGNGSSPALKRHLSEADKGEDRGGCKQFLNSRSISAPCTVSSPPQSNSMDQNGHHSHIGH
QDYDFLESNQDEPMDLSCVRSRREAPVPTETQVAPSAEEEKTAEQTKLPLAITTEQTVKGEPFPSFKPFLGNIVITDI
TTNCLTVTFKEYVTV

>Ssa_LOC106564893
MELPAAGEHVFAVESIEKKRNRKGRMEYLVKWRGWSPKYNTWEPEENILDPRLLDAFQNRERYEQLMGYRKRGPKPKH
LIGQVPSFARRSSVLSGLQETTLDEDNRQKVDPIQTLRSQTQQYQLNSKKHHQYQPLLAKEREAEQQANGKKKLYYQL
NSKKHHHYQPDPKMYDSTQCMKPREATKAPELPTNHSWNLPPALQQKWVRDKDSGCLSKVKDITMELKKLPAHLNDHS
GESEQSKATAKEDAPLLKANDVSDSKLKIVKNKNKNGRIVIVMSKYMENGMQAARIKNGDVETLDEPQPDNDAAENQL
EKMRLVQKLGLTNGFAKDCNKDVKLHVLNSGSNGSNVFKCPAEKVDSPKMELSATEQPLRLTTKPNLAPWPFEMGVQR
RTQPGNGSSPALKRHLSEADKGEDRGGCKRFLNSRSISAPCTVSSPPQSNSMDQNGHHSHNGHQDYDFLESNQDEPMD
LSCVRSRGEAPVPTETQVAPSAEEEKTAEQTKPPLAITTEYTVEEEPFPSFKPFLGNIVITDITTNCLTVTFKEYVTV

>Ssa_Cbx6 (Gene ID: 106591466)
MELSAVGERVFAAEAILKRRVRKSRLEYLVKWKGWALKHSTWEPEENILDGRLIAGFEQKERERELNGPKKRGPKPKN
LPAKARGAQKRETPTTRAPRSTSSSSPRAPSSSFPSASPAPSPKLNSLAATHKLKKDIRRCTSMSRRPLPRPDPLATP
GSFSGPSGFPSRPPVSPFSETVRILNRRVKPREVKRGRIILNLKVIDKPGSGGGGRGGGGNRRAPSGSMSLQGGRQNI
PSRNRIIGKRSGEAPYRPFQPMLGFPMYGKPFGLQCGGPGPLHLPHSATGSSTGTSGARDTQPPPSPSSSSGSTTPGQ
NHPTPPGPATGASGSAPNLSAQASASSAQSTPSHPPELQKNQNAEQGPTPLLPSLSFASGSSSSSFDEEEDDADALDL
SAPQEGKRKLRRRRRQKRQPPSVSTASPPHPPGACPLISNPQRVPAEGDPDWHPEMVASSANVVVTDVTTNLLTVTIK
EFPSPSSRPSSPQPILPENTTSSPPPPPPPPLKNPPTVQAIGRD

>Ssa_LOC106564427*
MELSAVGERVFAAEAILKRRVRKSRLEYLVKWKGWALKHSTWEPEENILDGRLIAGFEQKERERELHGPKKRGPKPKN
LPAKARGAQKRETPTTRAPRSTSSSSPRAPSSSFPSASPAPSPKLNSLAATHKLKKDIRRCT

>Ssa_LOC106595625*

TVRILNRRVKPREVKRGRIILNLKVIDKPGSGGGGRGGGGNRRAPSSSMSLQGGRQNIPSRNRIIGKRSGEAPYRPFQ
PMLGFPMYGKPFGLQCGGPGPLHLPHSATGSSTGTSGARDTQPPPSPSSSSGSTTPAQNHPTPPGPATGASGSAPNLS
AQASASSAQSTPSHPPELQKNQNAEQGPNPLLPSLSFASGSSSSSFDEEDDADALDLSAPQEGKRKLRRRRRQKRQPP
SVSTASPPHPPGACPLTSNPQRVPAEGNPDWHPEMVASSANVVVTDVTTNLLTVTIKEFPSPSSRPSSP

>Ssa_LOC106608943
MELSAIGEQVFAVESIIKKRVRKGNVEYLLKWKGWPPKYSTWEPEEHILDQRLVQAYEEKDQKDRALGYRKRGPKAKR
LLLQNTIYTMDLRSAHKVPEKPPARLRLSLTRSLDSEAEDPTYGACRRSVHPHLDHHKNKPRRSQFVRLASPPGPPTP
TQDPTHEDWGRRGEEDEEEDMEEEEAQQDESARETEMSNGILNGQNRAEDWSSVIGSEEVTVSERSAVWNPVIGPGEV
TVTDVTINSLTVTFREALAAKGFFRGWGLEF

>Ssa_LOC106589529
MELSAIGEQVFAVESIIKKRVKKGNVEYLLKWKGWPPKYSTWEPEEHILDQRLVQAYDEKEQKDRPLGYRKRGPKAKR
LLLQNTIYNMDLRSAHKALEKPPARLRLSLTRSLESEAEDPTYGACRRSLHPRLAHRKNKPRRSQFMCLASPPGPPNP
TKDPTHDDWGRREEEDDMEEEEETRQEQSERETEICSGILNGQDRAEDWSSVIGSDEVTASERSAVWSPVIGPGEVTV
TDVTINSLTVTFKEALAAKGFFRGWGLEF

>Ssa_LOC106607170
MELSSIGEQVFAVESIIKKRVRKGNVEYLLKWQGWPPKYSTWEPEDHILDPRLVLAYEEKEEKDRALAYRRKGLRPRR
LVLRSSLLLQNIYAMDLRSAHKVPDTPRLRLSLTRSMGSELDQGSLPYRAGEGGSVYRRLARRKNKQRVSKPVSDNNS
LQPLTRIQDPMEHEWQGAEERPESDTRHSRSECSSPMMEQEVESSTDRVESCGSALGSGDETLGGGALLRVTGAGYRS
EGGTSETGQEQMDRTDQSESCVSAEGPKYLNISNRLVNSGTEVELGTDTLIDKVERLGTANVIERVEGLGTDYFIDKV
EMGTHILIDGDSTSMVDVGDETVADGSVVCTDVEVAEEVVENDTKQQGEEVVTQCPDSSGAEVNPGKVIVTNVTINSL
TVTFKEALAAEGFFKG

>Ssa_LOC106601246
MELSSIGEQVFAVESITKKRVRKGNVEYLLKWQGWPPKYSTWEPEDHILDPRLVVAYEEKEEKDRALAYRRKGLRPRR
LVLRNIYAMDLRSAHKVPDTPRLRLSLTRSMGSELDQGSLPYRAGEGGSVYRRLARRKNKQRVSKPVSDNNSLQPLTR
IQDPMEHEWQGAEERPESELTTDTRENSLFRHSRSECSSPMMEQEVEESTDRVESCGSALGSGDETLGGGALLRVTGA
GYRSEGWTSETGQEQMDRTDQSESCVSAQGPKDNISNRLVDSDTEVELGTDTLIDKVERLGTVNVIERVEGLGTDYFI
DRVEEMGTHILIERDSTSVVDVGDETVADGSVVCTDVEVAEEVVENDTKQQGEEVVTQCPDSSGAEVNPGKVIVTDVT
INSLTVTFKEALAAEGFFKG

>Ssa_Cbx8 (Gene ID: 106588425)
MELSAVGERVFAAESIIKRRIRRGRMEYLVKWKGWSPKFSTWEPEENILDSRLFVAFEERERERELFGPKKRGPKPKT
FLLKAQAKAKAKSYEFRSDAVRGIRVTYPTPEPVITPRAREGLRAVVPTIFPPSTVNRGESVRVRPPELVREHRQPVL
QRPPGPDGFVTVPKKRGPKPKLRFKDNPFSAPPAAPEPPKRRAEEQATYGPLKLAKLGLSGGEERGSEMRGNKLAHRH
QEELGGYPHKQIRPVASGSTQQHCGPDRSMLHSHRIGSDSQACRTKECPSNYFSPPQLKHLSKKNVHQPSEELPQRGK
PSLIAKIPVSRILGQTDEVTWKPCLNNVEKVLVTDVTTNFLTVTIRESSTDQGFFKDKR

>Ssa_LOC106564894
MELSAVGERVFAAESIIKRRIRRGRMEYLVKWKGWSPKFSTWEPEENILDSRLFVAFEERERERELFGPKKRGPKPKT
FLLKAQAKAKSYEFRSDAVRGIRVTYPTPEPVITPRAREGLRAVVPTIFPPSTVNRGESVRVRPPEPVREHRQPVLQR
PLGPDGFVIVPKKRGPKPKLRFKDNPFNATSAATELHKRRAEEQATYGPLKLAKLGLSGGEERESEMRGIKLAHRHQL
ELGGYPHKQMRPVASGSTQQHSGPDRGLLHSHRIGSDSQACRTKECPSNYLSPPHLKHLSKKNVHQPNDELPQRGKPS
LIAKIPVSRILGQTDEVTWKPCMNNVEKVLVTDVTTNFLTVTIRESSTDQGFFKDKR

>Ssa_LOC106606673
MELSAVGERVFAAESIIKQRIRRGRMEYLVKWKGWSKKERERELFGPKKRGPKPETFLLKAKAKATAKTYECRREMPR
GIRVSYPVPTPMITPRAREGLRAVVPTIFPPSTVNRGESVRVRLPETERRPRPAPPPSFMAEEFVTIPKKRGPKPKLR
LRFNMEPDSCPTEEPGKRSRLEEQQVPYGQSKMSRHCHHEGETSERSVIQLTHRFQEGTSIVPKSNKAQRQVGTMSHP
GTHSHDGRLVHKARLDHQSRRTLECPGGMSIPHPKLKHVSKNHFYRASDSSSSMQQSRPIVVAKSPASRSSGEQSAVS
WRPRLDNVETVVVTDVTTNFLTVTIKESSTDKGFFKDKR

>Ssa_LOC106601767*
DLAFQVHVYETPYLSNLTTFIQAKAKATAKTYECRREMPRGIRVSYPVPTPMITPRAREGLRAVVPTIFPPSTVNRGE
SVRVRLPETERRPRPAPPPSFMAEEFVTIPKKRGPKPKLRLRFNMEPDSCPTEEPGKRSRLEEQQVPYGQSKMSRHCH
HEGETSERSVIQLTHRFQEGTSIVPKSNKAQRQVGTMSHPGTHSHDGRLVHKARLDHQSRRTLECPGGMSIPHPKLKH
VSKNHFYRASDSSSSMQQSRPIVVAKSPASRSSGEQSAVSWRPRLDNVETVVVTDVTTNFLTVTIKESSTDKGFFKDK
R

>Ssa_LOC106596252*
MPRGIRVSYPVPTPMITPRAREGLRAVVPTIFPPSTVNRGESVRVRLPETERRPRPAPPPSFMAEEFVTIPKKRGPKP
KLRLRFNMEPDSCPTEEPGKRSRLEEQQVPYGQSKMSRHCHHEGETSERSVIQLTHRFQEGTSIVPKSNKAQRQVGTM
SHPGTHSHDGRLVHKARLDHQSRRTLECPGGMSIPHPKLKHVSKNHFYRASDSSSSMQQSRPIVVAKSPASRSSGEQS
AVSWRPRLDNVETVVVTDVTTNFLTVTIKESSTDKGFFKDKR

>Omy_LOC110538562
MEELSAVGEQVFDAECILNKRLRKGKLEFLVKWRGWSSKHNSWEPQANILDPRLLAAFNKSEQEKEIQICKRGKRPRG
RPRKVVETVPEVSKSSSSSSSSSSSGSSSSSSSSSSSSSDDDDDDNNDRKAKPGPRTRELHPVPQKKAQIVVAKPEPPR
KKRGRKALPAEMKAIQQNKGQRKIIKTVAKDSPTDLRGGITKPFHPASFTFMGLNSRGPLSVQGRCSLAQGGTTKNSM
NTAGSGRSNTSASLSFNQRSNQSKSQASDFKLSVSDVDSGAGLDLKTTASKSGVAVLNLHNSKLSTSNGNPQGAFQPQ
LGSHNGQKKPDAPGQTPLQQVPNNKPASPFSTPKGPANQAASLQALNLQSVNKSTQGNGIPGNGTAPMSNLRSTANPA
RKNTVGQYGQEQNPVQSPATPGGQQPRKNHSGVDKVKAEETSEVGFTAERPERLTTTRAQGRVEKSIVQNTSTEARDI
LGKRERSASKDSGKQAKVLLSEMSTGEESTSDSDQDSPYPSNSQDLSISVQTGQDWKPTHSLIEHVFVTDVTANLVTV
TVKESPTSVGFFNIHNY

>Omy_LOC110486592
MEELSAVGEQVFDAECILNKRLRKGKLEFLVKWRGWSSKHNSWEPQANILDPRLLAAFNKSEQEKEILICKRGKRPRG
RPRKVVETVPEVSKSSSSSSSSSSSGSSSSSSSSSSSSSDEDDDDNNDRKAKPGPRTRELHPVPQKKAQIVVAKPEPPR
KKRGRKALPAEMKAIQQNKGQRKIIKTVAKDSPADLRGGIKKPFHPASFTFMGLNSRGPLSVQGRCSLAQGGTTKNSM
NTAASGRSNTLASLSFNQRSNQSKSQASDFKLSVSEVDSGAGLDLKTTASKSPGVAALNLHNSKLSTSNSNLQGAFQP
QLGSHNGQKKPDAPGQTPVQQVPNNKTAAPFSTPKGPANQAASLQALNLQSVNKSTQGNGTPGNGTAPVSNLRSTANP
ARKDTVGQYGLEKNPVQSPATPGGQQPRKNHPGVDKVKAEEVSETGVMTERPERLTTTRAQGRVEKSIVQNPSAEARD
ILGKRERSASKDSGKQAKVLLSEMSTGEESTSDSDQDSPYPSNSQDLSISVQTGQDWKPTHSLIEHVFVTDVTANLVT
VTVKESPTSVGFFNIHNY

>Omy_LOC110502771
MEGVTVGQVFDAECILNKRPRKGKFEYLVKWRGWSSKHNSWEPEENILDPRLMAAFHKRELERELLFGKRGKRPRGRP
RKILEPEPTETKSDQSSSASSSGLSSSPSASSSEDEDHGKKAKPGPRRLNLHPVLQMRPHIVVANQEPVRKKRGRKPL
LPELRALRQANTRLPLPPPPLSRHHQVLRPPREPPKEEPRGGVKKPLQPASFTYTSLSASRSSRDETAPQRAAGGAFF
QAGASKPGLLNSIWSGRSMSTITPSSPSSSSSLSRPPPPHSKGLSDLKHSLSVSDAGNSSGRAEGLKVASSVKPGVST
STSLVLHSSKTSSGFGGSPAACSPAQRSPAGQRRQQEGPGSQAVVVVQQQGAKPSSSPPPSARDRISQALSLRALNLQ
SVNKRAPGNGLQGYGTSGTTGVAVSRSSLRSGACQAGKGAGGIIKEMHTSSGLDQSLASGGLGAGMLRSGSIPQAGEE
RGERGKTTGAETEREMDSKRPRGVGSGNGRLERGGSVQGGVSSAGGGGSSAAREEDRRSEQSLNERNSRSVENSRTLN
ELSTGDSEDTSSSESEEPDSSPYPNNNGPRLVSMETEIETDWRPALSLLEHVFVTDVTANFVTVTVKESPTSVGFFNI
RNP

>Omy_LOC110499438
MEGVTVGHVFDAECILNKRPRKGKFEYLVKWRGWSSKHNSWEPEENILDPRLLAAFHKRAQERELLFRKRGKRPRGRP
RKILEPEPTETKSDRSSSSSSSSGLSSSPSSSSSEEEEEEEEDHRKKAKPGPRLRNLYPVPQKRPQIVVAKNEPVRKKR
GRKPLLPELRALRQAKTRPPLPPPSPSRHQQVLRPPREPFKEEPRGGVKKPLQPASFTYTGLSSSRGSRDEVAHQVAA
GGAFYQAGASKPGPLNSIWSGRSMSTNTPTPSSPSSSSSLSRPPPPYSKGWSDLKRSLSVSDAGSSNGRAEGFKVASS
LKPGMSTSTLCLHSSKTSSGCGGSLTACSPAQRFPAGQRRQQEGPGGQAGMVVQQQGAKPPSSTPPSARDRISQALSL
RALNLQSVKRIAPGNGLQGNGTSGTTGVAVSRLSLRSSASPAGKRAGGSIKETHTSSGLNQGLVSGGLGGGALHSGSV
TPARVERGERGKDTGAETEREMDNKGPGGVGRGNGRVEKGGSVQAQGGVSTARRGRANGGRQEDRKSGQSLTVNERNS
RSGDNSRTLNELSTGDSDDTSSSESEECDSPYPDNNNRARLVSMEMETETDWRPARSLLEHVFVTDVTANFVTVTVKE
SPTSVGFFNVRNH

>Omy_Cbx4 (Gene ID: 110485970)
MELPAAGEHVFAVESIEKKRNRKGRMEYLVKWRGWSPKYNTWEPEENILDPRLLDAFQNRERYEQLMGYRKRGPKPKH
LMGQVPSFARRSSVLSGLQETTLDEDNRQQVDPIQTVRSQTQQYQLNSKKHHQYQPLLAKEREAEQQANGKKKLYYQL
NSKKHHHYQPDPKMYDSTQCMKPREATKAPELPTNHSWNLPPALQQKWVRDKDSGCLSKVKDITMELKKLPAHLNGPS
GESEQSKATAKEDAPLLEADSVSDSKLKIVKNKNKNGRIVIVMSKYMENGMQAARIKNGDVETLDEPQPDNDDDAAEN
QLEKMRLVQKLGLTNGFAKDCNKGSNRSNVFKCPADKVDSPKMGLSATEQNKHFNVRVQKEATEDQPLQLTNKPNLAP
WPIEMGVHRTQPGKGSSPALKRHLSEADKGEDRGGCKQFLNSRSISIPCTVSSPPQSNSTDQNGHHSHNGQRDYDFLE
SNQDEPMDLSCVRSRREAPVPTETQVAPSAEEEKTAEQTKPPLAITTEQTVEEEPFPSFKPFLGNIVITDITTNCLTV
TFKEYVTV

>Omy_LOC110493691
MELPAAGEHVFAVESIEKKRNRKGRMEYLVKWRGWSPKYNTWEPEENILDPRLLDAFQNRERYEQLMGYRKRGPKPKH
MIAQVPSFARRSSVLSGLQETTLDEENRQKVDPIQTLRSQTQQYQLNSKKHHQYQPLLAKEREAEQQANGKKKLYYQL
NSKKHHHYQPDPKMYDSTQCMKPREATKAPELPTNHNWNLPPALQQKWVRDKDSGCLSKVKDITMELKKLPAHLNDHS
GESEQSKATAKEDAPLLKANGVSDSKLKIVKNKNKNGRIVIVMSKYMENGMQAARIKNGDVETLDEPQPDNDDDAAEN
QLEKMRLVQKLGLTNGFAKDCNKDVKLHVLNSGSNGSNVFKCPAEKVDSPKMELSATEQPLRLTTKPNLAPWPFEMGV
HRRTQPVNGSSPAFKRHLSEADKGEDRGGCKRFLNSRSISAPCTVSSPPQSNSMDQNGHHSHNGHQDYDFLESNQDEP
MDLSCVRSKREAPVPTETQVAPSAEEEKTAEQTKPPLAITTEQTKPPLAITTEHTVEEEPFPSFKPFLGNIVITDITT
NCLTVTFKEYVTV

>Omy_LOC110486813
MELPAAGEHVFAVEGIEKKRIRKGKIEYLVKWRGWSPKYNTWEPEENILDPRLLVAFQNRERQEQLMGYRKRGPKPKH
LLVQLPSFARRSSIPAGFEETSPEAENYLRSDPIQTHRSQPQQYQLNSKKHHQYQPSKQEVSADQLGNGKKKLFYQLN
SKKHHHYQPDPNMYNTQSTRPKDMVKGQEPSPPTNPNPGWNLPPALQQKWVRDKNTGCLSKVKDVTVVEMKKAAVRVN
EAESECALKPSPKEAALPSAVSSKMKIIKNKNKNGRIVIVMSKYMDGNKAGAAKGKHSSDLTGEEKPQHAEQSDMSKV
QGTTNVPKEICNASSTPAAEHPKKRSPEDGHFSKPSPSTAEEYNTEVARGQADLPDDHPLQLTTNPTPSPWAMDTSLP

TPSPMDQTRTLPTTNNDRKRKLSHPEEDRGVCKTFLSSRSISVPSAVPTPPQDKPMDLHLIVPRRSSSSGYGYEITDT
SISQEEPMDLSCSRTRKQPEPEPEPEPEHKDPTAVVQESETTTTTTTATTTTEEAKEESVLMFSPFMGNIVITDITT
NCLTVTFKEYVSV

>Omy_LOC110486808
ELPAAGEHVFAVEGIEKKRIRKGKIEYLVKWRGWSPKYNTWEPEENILDPRLLVAFQNRERQEQLMGYRKRGPKPKHL
LVQLPSFARRSSIPAGFEETSPEAENYLRSDPIQTHRSQPQQYQLNSKKHHQYQPSKQEVSADQLGNGKKKLFYQLNS
KKHHHYQPDPNMYNTQSTRPKDMVKGQEPSPPTNPNPGWNLPPALQQKWVRDKNTGCLSKVKDVTVVEMKKAAVRVNE
AESECALKPSPKEAALPSAVSSKMKIIKNKNKNGRIVIVMSKYMDGNKAGAAKGKHSSDLTGEEKPQHAEQSDMSKVQ
GTTNVPKEICNASSTPAAEHPKKRSPEDGHFSKPSPSTAEEYNTEVARGQADLPDDHPLQLTTNPTPSPWAMDTSLPT
PSPMDQTRTLPTTNNDRKRKLSHPEEDRGACKTFLSSRSISVPSAVPTPPQDKPMDLHLIVPRRSSSSGYGYEITDTS
ISQEEPMDLSCSRTRKQPEPEPEPEPEHKDPTAVVQESETTTTTTTATTTTEEAKEESVLMFSPFMGNIVITDITTN
CLTVTFKEYVSV

>Omy_Cbx6 (Gene ID: 110485591)
MELSAVGERVFAAEAILKRRVRKSRLEYLVKWKGWALKHSTWEPEENILDDRLIAGFEQKERERELHGPKKRGPKPKN
LPAKARGAQKRETSTPRAHDRLIAGFEQKERERELHGPKKRGPKPKNLPAKARGAQKRETSTPRAPRSTSSSSPRAPP
SSFPSASPAPSPKLNSLAATHKLKKDIRRCTSMSRRPLPRPDPLASPGSFSGSSGFPSRPPVSPFSETVRILNRRVKP
REVKRGRIILNLKVIDKPGSGGGGRGGGGNRRAPSGSMSLQGGRQNIPSRNRIIGKRSGEAPYRPFQPMLGFPMYGKP
FGLQCGGPGPLHLPHSATGSSTGTSGARDTQPPPSPSSSSGSTTPAQNHPTPPGPATGASASGSAPNLSTQALASSAQ
STPSHPPELQKNQNAEQGPNPLLPSLSFASGSSSSSFDEEEDDADALDLSSPQEGKRKLRRRRRQKRQPPSVSTASPP
HPPAARACPLTSNPQRVPAEGDPDWHPEMVASSANVVVTDVTTNLLTVTIKEFPSPSSRPSSPQPIHPENTTSSSPQL
KNPPQPKP

>Omy_LOC110536509*
MELSAVGERVFAAEAILKRRVRKSRLEYLVKWKGWALKHSTWEPEENILDDRLIAGFEQKERERELHGPKKRGPKPKN
LPAKARGAQKRETSTPRAPRSTSSSSPRAPPSSFPSASPYISPSPSLASTALFCSLLSLLALLGIGGNLYTMGLLIRR
RRGRRRRGCSSCCLGGRGVPLPSCLSSSPPSLSPSSSPTSSSSTSLHLQVLSLALADLLYLFTAPFIVYDSLAADWAF
GELGCRLLFSLDLITLHASIYTLTAMSLDRYRAVANPLATSSSPSSGLLRVALAWGLAVALSLPMMITLHLEDGDDGQ
LCVPAWDEQSSKVYMSVLFCTSMIGPGLAIGALYATLGRLYWVSQTQPWASGSGGSTSYPPRAPRPKVLLLILGIVLA
FWACFLPFWVWQLLPLYRPDMLRTVPVGTQVTVNRILTGLTYGNSCVNPFFYTLLTGGKRRRNRQTPTSANQLCRKSS
PQ

>Omy_LOC110538378
MELSSIGEQVFAVESITKKRVRKGNVEYLLKWQGWPPNTLTGELATTVRHLVHFRNVKSPSRSSVNPIPHASKTEGYS
TWEPEDHILDPRLVLAYEEKEEKDRALAYRRKGLRPRRLVLRSSLLLQNIYAMDLRSAHKVPDTPRLRLSLTRSMGSE
LDQGSLPYRAGEGGSVYRRLARRKNKQRVSKPVSDNNSLQPLTRIQDPMEHEWQGAEERPESELTTDTRENRHSRSEC
SSPMMEQEVEEPTDRVESCGSALGSGDETLGGGALLRVTGAWYRSEGRTSETGQEQIDRTDQSESCVSAEGPKDNISN
RLVDSATEVELGTDTLIDKVERLGTDNVIERVEGLGTDYFIDRVEEMGGHILIDRDSTSVVDVGDETVADGSVVCTDV
EVAEEVVENDTKQQGEEVVTQCPDSSGAEVNPGKVIVTDVTINSLTVTFKEALAAEGFFKS

>Omy_LOC110486408
MELSSIGEQVFAVESITKKRVRKGNVEYLLKWQGWPPNTLTGELATTVRHLVHFRNVKSPSRSSVNPIPHASKTEGYS
TWEPEDHILDPRLVLAYEEKEEKDRALAYRRKGLRPRRLVLRSSLLLQNIYAMDLRSAHKVPDTPRLRLSLTRSMGSE
LDQGSLPYRAGEGGSVYPRLARRKNKQRVSKPVSDNNSLQPLTRIQDPMEHEWQGAEERPESELTTDTRENSLFRHSR
SECSYPMMEQEVESTTDRVESCGSTLGSGDETLGGGALLRVTGAGYRSEGGTSETGQEQMDRTDQSESCGSAEGPEDL
NISNRLVDSDTEVELGTDTLIDRVERLGTANAIERVEGLGTDYFIDRVEMGTHILIDRDSTSVVDVGDETMADGSVVC
TDVEVAEEVVENDTKQQGEEVVTQCPDSSGAEVNPGKVIVTYVTINSLTVTFKEALAAEGFFKS

>Omy_LOC110499099
MELSAIGEQVFAVESIIKKRVRKGNVEYLLKWKGWPPKYSTWEPEEHILDQRLVQTYDEKEQKDRALGYRKRGPKAKR
LLLQNTIYNMDLRSAHKALEKPPARLRLSLTRSLESEAEDPTYGACRRSLHPRLAHRKNKPRRSQFMCLASPPGPPNP
TKVPTHDDWGRREEEDDMEEEEETRQEQSERETEICSGILNGQDRAEDWSSVIGSDEVTASERSAVWSPVIGPGEVTV
TDVTINSLTVTFKEALAAKGFFRGWGLEF

>Omy_LOC110502860
MELSAIGEQVFAVESIIKKRVRKGNVEYLLKWKGWPPKYSTWEPEEHILDQRLVQAYEEKDQKDRALGYRKRGPKAKR
LLLQNTIYTMDLRSAHKAPEKHPARLRLSLTRSLDSEAEDPTYGACRRSLHPHLDHHKNKPRRSQFVRLASPPGPPTP
TQDPTHEDWGRREEEDEEEEARQDESERETEMSSGILNGQNRAEDWSSVIGSEEVTVSERSDVWNPVIGPGEVTVTDV
TINSLTVTFREALAAKGFFRGWGLEF

>Omy_Cbx8 (Gene ID: 110485969)
MELSAVGERVFAAESIIKRRIRRGRMEYLVKWKGWSPKFSTWEPEENILDSRLFVAFEERERERELFGPKKRGPKPKT
FLLKAQAKAKAKLYEFRSDAVRGIRVTYPTPEPVITPRAREGLRAVVPTIFPPSTVNRGESVRVRPPEPVREHRQPVL
QRPPGPDGFVIVPKKRGPKPKLRFKDNPFNAPPAALSPPKRRAEEQATYGPLKLAKLGLSGGEERGSEMRGIKLAHRH

QEELGGYPHKQIRPVASGSTQQHCGPDRGMLHSHRIGSDSQACRTKECPSNYLSPPQLKHLSKKNVHQPSEELPQRGK
PSLIAKIPVSRILGQTDEVTWKPCLNNVEKVLVTDVTTNFLTVTIRESSTDQGFFKDKR

>Omy_LOC110519423
LQGRMEYLVKWKSWSKKYSTWEPEENILDARLFAAFEEREREREFGPKKRGPKPETFLLKAKAKATAKTYECRREMP
RGIRVSYPVPMPMITPRAREGLRAIVPTIFPPSTVNRGESVRVRLPETERRPRPAPPHPFMAEEFVNIPKKRGPKPKL
RLRFNMEPDSCPTEEPAKRSRLEEQQVPYGLSKMSRHCHHEGETSKRSVIQLTRRFQEGTSIVPKSNKAQRLLGTVSH
PGTHSHDGRLLHKARLDQQSRRTLECPGGMSVPHPKLKHVSTKNHLYRASDSSSSMQQSRPIVVAKSPASRSSGEQSA
VSWRPCLDNVENVVVTDVTTNFLTVTIKESSTDKGFFKDNR

>Omy_LOC110493689
MELSAVGERVFAAESIIKRRIRRGRMEYLVKWKGWSPKFSTWEPEENILDSRLFVAFEEREREREFGPKKRGPKPKT
FLLKAQAKAKAKSYEFRSDAVRGIRVTYPTPEPVITPRAREGLRAVVPTIFPPSTVNRGESVRVRPPEPVREHRQPVL
QRPPGPDGFVIVPKKRGPKPKLRFKDNPFNATSAATEPHKRRAEEQATYGPLKLAKLGLSDGEERGSEMRGIKLAHRH
QVELGGYPHKQMRPVASGSTQQHYGPDRGLLHSHRIGSDSQACRTKECPSNYLSPPHLKHLSKKNGHQPSEELPQRGK
PSLIAKIPVSRILGQTDEVTWKPCMNNVEKVLVTDVTTNFLTVTIRESSTDQGFFKDKR

>Omy_LOC110486816
MELSAVGERVFAAESIIKQRIRRGRMEYLVKWKSWSKKYSTWEPEENILDARLFAAFEEREREREFGPKKRGPKPET
FLLKAKAKATAKTYECRREMPRGIRVSYPVPMPMITPRAREGLRAIVPTIFPPSTVNRGESVRVRLPETERRPRPAPP
HPFMAEEFVNIPKKRGPKPKLRLRFNMEPDSCPTEEPAKRSRLEEQQVPYGLSKMSRHCHHEGETSKRSVIQLTRRFQ
EGTSIVPKSNKAQRLLGTVSHPGTHSHDGRLLHKARLDQQSRRTLECPGGMSVPHPKLKHVSTKNHLYRASDSSSSMQ
QSRPIVVAKSPASRSSGEQSAVSWRPCLDNVENVVVTDVTTNFLTVTIKESSTDKGFFKDNR

>Omy_LOC110486815*
MPRGIRVSYPVPMPMITPRAREGLRAIVPTIFPPSTVNRGESVRVRLPETERRPRPAPPHPFMAEEFVNIPKKRGPKP
KLRLRFNMEPDSCPTEEPAKRSRLEEQQVPYGLSKMSRHCHHEGETSKRSVIQLTRRFQEGTSIVPKSNKAQRLLGTV
SHPGTHSHDGRLLHKARLDQQSRRTLECPGGMSVPHPKLKHVSTKNHLYRASDSSSSMQQSRPIVVAKSPASRSSGEQ
SAVSWRPCLDNVENVVVTDVTTNFLTVTIKESSTDKGFFKDNR

>Omy_LOC110486809*
MPRGIRVSYPVPMPMITPRAREGLRAIVPTIFPPSTVNRGESVRVRLPETERRPRPAPPHPFMAEEFVNIPKKRGPKP
KLRLRFNMEPDSCPTEEPAKRSRLEEQQVPYGLSKMSRHCHHEGETSKRSVIQLTRRFQEGTSIVPKSNKAQRLLGTV
SHPGTHSHDGRLLHKARLDQQSRRTLECPGGMSVPHPKLKHVSTKNHLYRASDSSSSMQQSRPIVVAKSPASRSSGEQ
SAVSWRPCLDNVENVVVTDVTTNFLTVTIKESSTDKGFFKDNR

>Oki_LOC109898685
MEELSAVGEQVFDAECILNKRLRKGKLEFLVKWRGWSSKHNSWEPQANILDPRLLAAFNKSEQEKEILICKRGKRPRG
RPRKVVETVPEVSKSSSSSSSSSSSGSSSSSSSSSSSSSDDDDDDNNDRKAKPGPRTRELHPVPQKKAQIVVAKPEPPR
KKRGRKALPAEMKAIQQNKGQRKIIKTVAKDSPADLRGGIKKPFHPASFTFMGLNSRGPLSVQGRCSLAQGGTTKNSM
NTAGSGRSNTLASLSFNQRSNQSKSQASDFKLSVSEVDSGAGLDLKTTASKSPGVAALNLHNSKLSTSNGNLQGAFQP
QLGSHNGQKKPDAPGQTPVQQVPNNKTAAPFSTPKGPANQAASLQALNLQSVNKSTPGNGTPGNGTAPVSNLRSTANP
ARKDTVGQYDLEKNPVQSPATPGGQQPRKNHPGVDKVKAEEISEAGVMTERPERLTTTRAQGRVEKSIVQNPSTEARD
ILGKRERSASKDSGKQAKVLLSEMSTGEESTSDSDQDSPYPSNSQDLSISVQTGQDWKPTHSLIEHVFVTDVTANLVT
VTVKESPTSVGFFNIHNY

>Oki_LOC109879638
MEELSAVGEQVFDAECILNKRLRKGKLEFLVKWRGWSSKHNSWEPQANILDPRLLAAFNKSEQEKEILICKRGKRPRG
RPRKVVETVPEVSKSSSSSSSSSSSGSSSSSSSSSSSSSDDDDDDNNDRKAKPGPRTRELHPVPQKKAQIVVAKPEPPR
KKRGRKALPAEMKAIQQNKGQRKIIKTVAKDSPADLRGGIKKPFHPASFTFMGLNSRGPLSVQGRCSLAQGGTTKNSM
NTAGSGRSNTSASLSFNQRSNQSKSQASDFKLSVSDVDSGAGLDLKTTASKSGVAVLNLHNSKLSTSNGNLQGAFQPQ
LGSHNGQKKPDAPGQTPVQQVPNNKTAAPFSTPKGPANQAASLQALNLQSVNKSTPGNGTPGNGTAPVSNLRSTANPA
RKDTVGQYDLEKNPVQSPATPGGQQPRKNHPGVDKVKAEEISEAGVMTERPERLTTTRAQGRVEKSIVQNPSTEARDI
LGKRERSASKDSGKQAKVLLSEMSTGEESTSDSDQDSPYPSNSQDLSISVQTGQDWKPTHSLIEHVFVTDVTANLVTV
TVKESPTSVGFFNIHNY

>Oki_LOC109870514
MEGVTVGHVFDAECILNKRPRKGKFEYLVKWRGWSSKHNSWEPEENILDPRLLAAFHKRAQERELLFRKRGKRPRGRP
RKILEPEPTETKSDRSSSSSSSGLSSSPSSSSSEEEEEEDHRKKAKPGPRLRNLYPVPQKRPQIVVAKNEPVRKKRGR
KPLLPELRALRQAKTRPPLPPPSPSRHQQVLRPPREPFKEEPRGGVKKPLQPASFTYTGLSSSRGSRDEVAHQVAAGG
AFYQAGASKPGPLNSIWSGRSMSTNTPTPSSPSSSSSLSRPPPPYSKGWSDLKRSLSVSDAGSSNGRAEGFKVASSLK
PGMSTTTLCLHSSKTSSGCGGSSTACSPAQRFPAGQRRQQEGPGGQAGMVVQQQGAKPPSSTPPSARDRISQALSLRA
LNLQSVKRIAPGNGPQGNGTSSTTGVAVSRLSLRSSASPAGKRAGGSIKETHTSSGLNQGLVSGGLGGGALHSGSVPP
ARVERGERGKDTGAETEREMDNKGPGGVGMGNGRVEKGVSVQAQGGVSTARRGRANGGRQEDRKSGQSLTVNERNSRT
LNELSTGDSDDTSSSESEECDSPYPDNNNNRARLVSMEMETETDWRPARSLLEHVFVTDVTANFVTVTVKESPTSVGF
FNVRNH

>Oki_LOC109899149
MEGVTVGQVFDAECILNKRPRKGKFEYLVKWRGWSSKHNSWEPEENILDPRLMAAFHKREQERELLFGKRGKRPRGRP
RKILEPEPTETKSDRSSSASSSGLSSSPSASSSEDEDHGKKAKPGPRRLNLHPVLQTRPHIVVANQEPVQKRRGRKPL
LPELRALRQANTRLPLPPPPLSRHHQVLRPPREPPKEEPRGGVKKPLQPASFTYTSLSASRGSRDETASQRAAGGAFF
QAGASKPGMLNSIWSGRSMSTITPSSPSSSSSLSRPPPPHSKGLSDLKRSLSVSDAGNSSGRAEGLKVASSVKPGVST
LTSLGLHSSKTSSGFGGSPAACSPAQRSPAVQRRQQEGPGSQAVVVVQQQGAKPSSSPPPSARDRISQALSLRALNLQ
SVNKRAPGNGLQGYGTSGTTGIAVSRSSLRSGASQAGKGAGGIIKEMHTSSGLDQSLASGGLGAGMLRSGSIPQAREE
RGKTTGAETEREMDSKGPRGVGSGNGRLERGGSVQGGVSSAGGGGSSAAREEDRRSEQSLNERNSRSVENSRTLNELS
TGDSDDTSSSESEEPDSSPYPNNNGPRLVSMETEIETDWRPALSLLEHVFVTDVTANFVTVTVKESPTSVGFFNVRNP

>Oki_Cbx4 (Gene ID: 109865985)
MELPAAGEHVFAVESIEKKRNRKGRMEYLVKWRGWSPKYNTWEPEENILDPRLLDAFQNRERYEQLMGYRKRGPKPKH
LMGQVPSFARRSSVLSGLQETTLDEDNRQQVDPIQTVRSQTQQYQLNSKKHHQYQPLLAKEREAEQQANGKKKLYYQL
NSKKHHHYQPDPKMYDSTQCMKPREATKAPELPTNHSWNLPPALQQKWVRDKDSGCLSKVKDITMELKKLPAHLNGPS
GESEQSKATAKEDAPLLEADGVSDSKLKIVKNKNKNGRIVIVMSKYMENGMQAARIKNGDVETLGEPQPDNNDDAAEN
QLEKMRLVQKLGLTNGFAKDCNKGSNRSNEFKCPADKVDSPKMGLSATEQNKHFNVRDQKEATEDQPLQLTNKPSLAP
WPIEMGVHRTQPGKGSSLALKRHLSEADKGEDRGGCKQFLNSRSISIPCTVSSPPQSNSTDQNGHHSHNGQQDYDFLE
SNQDEPMDLSCVRSRREAPVPTETQVAPSAEEEKTAEQTEPPLAITTEQTVEEEPFPSFKPFLGNIVITDITTNCLTV
TFKEYVTV

>Oki_LOC109894654
MELPAAGEHVFAVESIEKKRNRKGRMEYLVKWRGWSPKYNTWEPEENILDPRLLDAFQNRERYEQLMGYRKRGPKPKH
LIGQVPSFARRSSVLSGLQETTLDEENRQKVDPIQTQQYQLNSKKHHQYQPLLAKEREAEQQANGKKKLYYQLNSKKH
HHYQPDPKMYDSTQCMKPREATKAPELPTNHSWNLPPALQQKWVRDKDSGCLSKVKDITMELKKLPAHLNDHSGKSEQ
SKATAKEDAPLLKANDVSDSKLKIVKNKNKNGRIVIVMSKYMENGMQAARIKNGDVETLDEPQPDNDDDAAENQLEKM
RLVQKLGLTNGFAKDCNKDVKLHSLNSGSNGSNVFKCPAEKVDSPKMELSATEQPLRLTTKPNLAPWPFEMGVHRRTQ
PVNGSSPAFKRHLSEADKDEDRGGCKRFLNSRSISAPCTVSSPPQSNSMDQNGHHSHNGHQDYDFLESNQDEPMDLSC
VRSKREAPVPTETQVAPSAEEEKTAEQTKPPLAITTEQTKPPLAITTEHTVEEEPFPSFKPFLGNIVITDITTNCLTV
TFKEYVTV

>Oki_LOC109887742
MELPAAGEHVFAVESIEKKRNRKGRMEYLVKWRGWSPKYNTWEPEENILDPRLLDAFQNRERYEQLMGYRKRGPKPKH
LIGQVPSFARRSSVLSGLQETTLDEENRQKVDPIQTQQYQLNSKKHHQYQPLLAKEREAEQQANGKKKLYYQLNSKKH
HHYQPDPKMYDSTQCMKPREATKAPELPTNHSWNLPPALQQKWVRDKDSGCLSKVKDITMELKKLPAHLNDHSGKSEQ
SKATAKEDAPLLKANDVSDSKLKIVKNKNKNGRIVIVMSKYMENGMQAARIKNGDVETLDEPQPDNDDDAAENQLEKM
RLVQKLGLTNGFAKDCNKDVKLHSLNSGSNGSNVFKCPAEKVDSPKMELSATEQPLRLTTKPNLAPWPFEMGVHRRTQ
PVNGSSPAFKRHLSEADKDEDRGGCKRFLNSRSISAPCTVSSPPQSNSMDQNGHHSHNGHQDYDFLESNQDEPMDLSC
VRSKREAPVPTETQVAPSAEEEKTAEQTKPPLAITTEQTKPPLAITTEHTVEEEPFPSFKPFLGNIVITDITTNCLTV
TFKEYVTV

>Oki_LOC109882827
MELPAAGEHVFAVEGIEKKRIRKGKIEYLVKWRGWSPKYNTWEPEENILDPRLLVAFQNRERQEQLMGYRKRGPKPKH
LLVQLPSFARRSSIPAGFEETSPEAENHLRSDPIQMHRSQPQQYQLNSKKHHQYQPSKQEVSADQLGNGKKKLFYQLN
SKKHHHYPGAPEPXYVQPQSTRPKDMVKGQEPSPPTNPNPGWNLPPALQQKWVRDKNTGCLSKVKDVTVXDEKAVVRV
NEAESECALKPSPKEAALPSAVSSKMKTSRTRTRMDALSSSMPAAEHPKKHSPEDGHFSKPSPSTAEEYNTEDKPMDL
LHLIVPRRSSSSGYGYEITDTSTSQEEPMDLSCSRTRKQPEPEPVPEPEPEHKDPTAVVQESETTTTTTTTATTTTEE
TKEESVLMLSPFMGNIVITDITTNCLTVTFKEYVSV

>Oki_Cbx6 (Gene ID: 109884207)
MELSAVGERVFAAEAILKRRIRKSRLEYLVKWKGWALKHSTWEPEENILDDRLIAGFEQKERERELHGPKKRGPKPKN
LPAKARGAQKRETSTPRAPRSTSSSSPRAPPSSFPSASPAPTPKLNSLAATHKLKKDIRRCTSMSRRPLPRPDPLASP
GSFSGSSGFPSRPPVSPFSETVRILNRRVKPREVKRGRIILNLKVIDKPGSGGGGRGGGGNRRAPSGSMSLQGGRQNI
PSRNRIIGKRSGEAPYRPFQPMLGFPMYGKPFGLQCGGPGXLHLPHSATGSSTGTSGARDTQPPPSPSSSSGSTTPAQ
NHPTPPGPATGAXGSAPNLSTQASASSAQSTPSHPPELQKNQNAEQGPNPLLPSLSFASGSSSSSFDEEEDDADALDL
SAPQEGKRKLRRRRRQKRQPPSVSTASPPHPAAASSCPLTSNPQRVPAEGDPDWHPEKVASSANVVVTDVTTNLLTVT
IKEFPSPSSRPSSPQPIHPENTTSSPQLKNPPQPKP

>Oki_Cbx7 (Gene ID: 109899160)
MELSAIGEQVFAVESIIKKRVKKGKVEYLLKWKGWPPKYSTWEPEEHILDQRLVQAYEEKDQKDRALGYRKRGPKAKR
LLLQNTIYTMDLRSAHKAPEKPPARIRLSLTRSLDSEAEDPTYGACRRSLHPHLDHHKNKPRRSQFVRLASPPGPPTP
TQDPTHEDWGTREEEDEEEDMEEEEARQDESERETEMSCGILNGQNRAEDWSSVIGSEEVTVSERSDVWNPVIGPGEV
TVTDVTINSLTVTFREALAAKGFFRGWGLEF

>Oki_LOC109898530
MELSSIGEQVFAVESITKKRVRKGNVEYLLKWQGWPPKYSTWEPEDHILDPRLVLAYEEKEEKDRALAYRRKGLRPRR
LVLRSSLLLQNIYAMDLRSAHKVPDTPRLRLSLTRSMGSELDQGSLPYRAGEGGSIYPRLARRKNKQRVSKPVSDNNS
LQPLTRIQDPMEHEWQGAEERPESELTTDTRHSRSECSYPMMEQEVESTPDRVESCGSTLGSGDETLGGGALLRVTVA
GYRSEGGTSETGQEQMDRTDQSESCVSAEGPEDLNISNRLVDSDTEVELGTDTLIDRVERLGTANAIERVEGLGTDYF

IDRVEEMGTHILIDRDSTSVVDVGDETVADGSVVCTDVEVAEEVVENDTKQQGEEVVTQCPNSSGAEVNPGKVIVTYV
TINSLTVTFKEALAAEGFFKS

>Oki_LOC109870400
MELSAIGEQVFAVESIIKKRVRKGNVEYLLKWKGWPPKYSTWEPEEHILDQRLVQTYDEKEQKDRALGYRKRGPKAKR
LLLQNTIYNMDLRSAHKALEKPPARLRLSLTRSLESEAEDPTYGACRRSLHPRLAHRKNKPRRSQFMCLASPPGPPNP
TKVPTHDDWGRREEEDDMEEEEETRQEQSERETEICSGILNGQDRAEDWSSVIGSDEVTASERSAVWSPVIGPGEVTV
TDVTINSLTVTFKEALAAKGFFRGWGLEF

>Oki_LOC109889501
MELSAVGERVFAAESIIKRRIRRGRMEYLVKWKGWSPKFSTWEPEENILDSRLFVAFEERERERELFGPKKRGPKPKT
FLLKAQAKAKSYEFRSDAVRGIRVTYPTPEPVITPRAREGLRAVVPTIFPPSTVNRGESVRVRPPEPVREHRQPVLQR
PPGPDGFVIVPKKRGPKPKLRFKDNPFNATSAATEPHKRRAEEQATYSPLKLAKLGLSDGEERGSEMRGIKLAQRPQV
ELGGYPHKQMRPVASGSTQQHYGPDRGLLHSHRIGSDSQACRTKECPSNYLSPPQLKHLSKKNVHQPSEELPQRGKPS
LIAKIPVSRILGQTDEVTWKPCMNNVEKVLVTDVTTNFLTVTIRESSTDQGFFKDKR

>Oki_LOC109882830
MELSAVGERVFAAESIIKQRIRRGRMEYLVKWKGWSKKYSTWEPEENILDARLFAAFEERERERELFGPKKRGPKPET
FLLKAKAKATAKTYECRREMPRGIRVSYPVPMPMITPRAREGLRAVVPTIFPPSTVNRGESVRVRLPETERRLRPAPP
LPFMAEEFVNIPKKRGPKPKLRLRFNMEPDSCPTEEPAKRSRLEEQQVPYGLSKMSRHCHHEGETSKRSVIQLTRRFQ
EGTSIVPKSNKAQRLLGTVSHPGTHSHDGRLVHKARLDQQSRRTLECPGGMSVPHPKLKHVSKNHFYRASDSSSSMQQ
SRPIVVAKSPASRSSGEQSAVSWRPCLDNVEKVVVTDVTTNFLTVTIKESSTDKGFFKDNR

Oki_LOC109882828*
MSPTPRLIFMYVLFLCWLSPSAADLCNWSGSGLAREAEANSRTVQQVRLRCTEGSVEWVYPGQALRVVLEPNLSSARH
TTVCIKPFRSFNGASVYIERAGELDLLMTDGGRPEQVFCFPADGPQKPAIFLLANPQRDISQRAVGFRYELLGNQTTA
PNLGRSVLQASCRPCNDTELLLAICSSDFVVRGSIRNVSHNAERQTSLVEVSDGRVYRQRSGVFERHTGTIGVPGSSS
SWHGHIHTLLQCHVKPGGGEFLFTGTEHFGEAWLGCAPRYKDFMSLYQSAWVARQNPCEFPLD

Oki_LOC109866077
MELSAVGERVFAAESIIKRRIRRGRMEYLVKWKGWSPKFSTWEPEENILDSRLFVAFEERERERELFGPKKRGPKPKT
FLLKAQAKAKAKSYEFRSDAVRGIRVTYPTPEPVITPRAREGLRAVVPTIFPPSTVNRGESVRVRPPEPIREHRQPVL
QRPRGPDGFVIVPKKRGPKPKLRFKDNPFNAPPAALSPPKRRAEEQATYGPLKLAKLGLSGGEERGSEMRGIKLAHRH
QEELGGYPHKQIRPVASGSTQQHCGPDRGMLHSHRIGSDSQACRTKECPSNYLSPPQLKHLSKKNVHQPSEELPQRGK
PSLIAKIPVSRILGQTDEVTWKPCLNNVEKVLVTDVTTNFLTVTIRESSTDQGFFKDKR

>Sal_LOC111977406
MEGVTVGQVFDAECILNKRPRKGKFEYLVKWRGWSSKHNSWEPEENILDPRLMAAFHKREQERELLFGKRGKRPRGRP
RKILEPEPTETKSDRSSSSSSSGLSSSPSASSSEEEDEDHGKKAKPGPRRRNLNLQPVLQTRPHIVVANQEPIRKKRG
RKPLLPELRALRQANIRPPLPPPPLSRHHQVLRPPREPPKEEPRGGVKKPLQPASFTYTSLSASRGSRDETAPQRAAG
GAFFQAGASKPGPLNSIWSGRSMSTITPSSPSSSSSLSRPPPPHSKGLSDLKRSLSVSDAGNSSGRAEGLKVAFSVKP
GVSTSTSLGLHSSKTSSGFGGSPAACSPAQRSPAGQRRQQEGPGSQAVVVVQQQGAKPSSSPPLSARDRISQALSLRA
LNLQSVNKRAPGNGLQGYGTSGTTGVAVSRSSLRSGASQAGKGAGGSIKEMHTSSGLDQSLASGGLGAGVLRSGCIPQ
AREERGERGKTTGAETEREMDSKGPRGVGSGNGRLERGGSAQGGMSSAGGGGSSAAREEDRRSEQSLNERNSRSIENS
RTLNELSTGDSDDTSSSESEEPDSSPYPNNNGPRLVSMETEMETDWRPALSLLEHVFVTDVTANFVTVTVKESPTSVG
FFNVRNP

>Sal_LOC112077672
MEGVTVGHVFDAECILNKRPRKGKFEYLVKWRGWSSKHNSWEPEENILDPRLLAAFHKRAQERELLFRKRGKRPRGRP
RKILEPEPTETKSDRSSSSSSSGLSSSPSASSSEEEDEDHGKKAKPGPRRRNLNLQPVLQTRPHIVVANQEPIRKKRG
RKPLLPELRALRQAKTRPPLPPPSPSRHHQVLRPPREPFKEEPRGGVKKPLQPASFTYTGLSSSRGSRDEVAHQVAAG
GAFYQAGVSKPGPLNSIWSGRSMSTNTPTPSSPSSSSSLSRPLPSYSKGWSDLKRSLSVSDAGSSNGRAEGLKVASSL
KPGVSTSTSLCLHSSKTSSGCGGSPTACSPAQRFPAGQRRQQEGLGGQAGMVVQQQGAKPPSSTPPSARDRISQALSL
RALNLQSVKKIAPCNGLHGNGTSGTTGVAVSRLSLRSSASPAGKRAGGSIKETHTTSGLNQGLVSGGLGGGALHSGSV
PPARVERGERGKDTGVETEREMDNKGPGGVGRGNGRVEKGGSVQAQGGVSTARRGRANGGRQEDRKSGQSLTVNERNS
RSGDNSRTLNELSTGDSDDTSSSESEECDSSPYPDNNNRARLVSMEMETETDWRPARSLLEHVFVTDVTANFVTVTVK
ESPTSVGFFNVRNH

>Sal_LOC112073971
MEELSAVGEQVFDAECILNKRLRKGKLEFLVKWRGWSSKHNSWEPQTNILDPRLLAAFNKSEQEKEILISKRGKRPRG
RPRKVVETVPEVSKSSSSSSSSSSSGSSSSSSSSSSSSDDDDDDNNDRKAKPGPRTRELHPVPQKKAQIVVAKPEPPR
KKRGRKALPAEMKAIQQNKGQRKIIKTVAKDSPADLRGGIKKPFHPASFTFMGLNSRGPLSVQGRCSLAQGGTTKNSM
NTAGRSNTSASLSFNQRSNQSKSHASDFKLSVSEVDSGAGLNLKTTASKSPGVAALNLHNSKLSTSNGNPQGAFQPQL
GSHNGQKKPDAPGQTPXQQVPNNKPAAPLSTPKGPANQAASLQALNLQSVNKSTPGNGTPGNGTVPVSNLRSTANPAR
KDTVGQYGQEKNPXQSPVTPGGQQPRKNHPGVDKVKAEETSEVGVTAERPERLTTTRAQGRVEKSIVQNPSAEARDIL
GKRERSASKDSGKQAKVLLSEMSTGEESTSDSDXDSPYPSNSQXLSISVQTGQDWKPTHSLIEHVFVTDVTANLVTVT
VKESPTSVGFFNIHNY

```
>Sal_LOC112070653
MEELSAVGEQVFDAECILNKRLRKGKLEFLVKWRGWSSKHNSWEPQANILDPRLLAAFNKSEQEKEILISKRGKRPRG
RPRKVVETVPEVSKSSSSSSSSSSSGSSSSSSSSSSSSSDDDDDDNNDRKAKPGPRTRELHPVPQKKAQIVVAKPEPPR
KKRGRKALPAEMKAIQQNKGQRKIIKTVAKDSPADLRGGIKKPFHPASFTFMGLNSRGPLSVQGRCSLAQGGTTKNSM
NTAGRSNTSASLSFNQRSNQSKSXASDFKLSVSXVXSGAGLXLKTTASKSXXXXXXXXAPLSTPKGPANQAASLQALN
LQSVNKSTPGNGTPGNGTVPVSNLRSTANPARKDTVGQYGQEKNPXQSPVTPGGQQPRKNHPGVDKVKAEETSEVGVT
AERPERLTTTRAQGRVEKSIVQNPSAEARDILGKRERSASKDSGKQAKVLLSEMSTGEESTSDSDXDSPXPSNSQXLS
ISVQTGQDWKPTHSLIEHVFVTDVTANLVTVTVKESPTSVGFFNIHNY

>Sal_LOC111956774
MELPAAGEHVFAVESIEKKRNRKGRMEYLVKWRGWSPKYNTWEPEENILDPRLLDAFQNRERYEQLMGYRKRGPKPKH
LMGQVPSFARRSSVLSGLQETALDXDNRQKVDPIQTVRSQTQQYQLNSKKHHQYQPLLAKEREAEQQANGKKKLYYQL
NSKKHHHYQPDPKMYDSTQCMKPREATKAPELPTNHSWNLPPALQQKWVRDKDSGCLSKVKDITMELKKLPAHLNXHS
GESEQSKATAKEDAPLLKDNGVSDSKLKIVKNKNKNGRIVIVMSKYMENGMQAARIKNGDVETLDEPQPGNDDDAAEN
QLEKMRLVQKLGLTNGFAKDCKKGSNGSNVFKCPADKVDSPKMELSATEQNKHFNVRGQKEATEDQPLQLTTKPNLAP
WPFEMGVHRTQXGNGSSPALKRHLSEADKGEDRGGCKQFLNSRSISAPCTVSSPPQSNSMDQNGHHSHNGHQDESNQD
EPMDLSCVRSRREAPVPTETQVAPAEEEKTAEQTKPPLAITTEQTVKGKPFPSFKPFLGNIVITDITTNCLTVTFKEY
VTV

>Sal_LOC111953935
MELPAAGEHVFAVESIEKKRNRKGRMEYLVKWRGWSPKYNTWEPEENILDPRLLDAFQNRERYEQLMGYRKRGPKPKH
LIGQVPSFARRSSVLSGLQETTLDEDNRQKVDPIQTLRSQTQQYQLNSKKHHQYQPLLAKEREAEQQANGKKKLYYQL
NSKKHHHYQPDPKMYDSTQCMKPREATKAPELPTNHSWNLPPALQQKWVRDKDSGCLSKVKDITMELKKLPAHLNDHS
GESEQSKATAKEDAPLLKANDVSDSKLKIVKNKNKNGRIVIVMSKYMENGMQAARIKNGDVETLDEPQPDNDDDAAEN
QLEKMRLVQKLGLTNGFAKDCNKDVKLHVLNSGSNGSNVFKCPAEKVDSPKMELSATEQPLRLTTKPNLAPWPFEMGV
HRRTQPVNGSSPALKRHLSEADKGEDRGGCKRFLNSRSISAPCTVSSPPQSNSMDQNGHHSHNGHQDYDFLESNQDEP
MDLSCVRSRGEAPVPTETQVAPSAEEEKTAEQTKPPLAITTEHTVEEEPFPSFKPFLGNIVITDITTNCLTVTFKEYV
TV

>Sal_LOC111950939
MGLQEWRPTPLPHLIDGQHAYAVRHLLKVRPRGRGFQYLVDWEGYGQEERCWVPARDILDPGLIADFQYRHPGQPGMC
PACCQFILFIKPTSVFPLVPVLCIVPVF

>Sal_LOC112069120
MELPAAGEHVFAVEGIEKKRIRKGKIEYLVKWRGWSPKYNTWEPEENILDPRLLVAFQNRERQEQLMGYRKRGPKPKH
LLVQLPSFARRSSIPAGFEETSPETENHLRSDPIQMHRSQPQQYQLNSKKHHQYQPSKQEVSADQLGNGKKKLFYQLN
SKKHHHYQPDPNMYNTQSTRPKDVVKGQEPSPPTNPNPGWNLPPALQQKWVRDKNTGCLSKVKDVTVVEMKKAAVRVN
EAESECALKPSPKEAALPSAVSSKMKIIKNKNKNGRIVIVMSKYMDGNKAGAAKGKHSSDLTGEEKPQHAEQSDMTKV
QGTTNVPKEICNASSPPAAEHPKKCSPEDGHFSKPSPSTAEEYNTEVARGQADLPDDHPLQLTTNPTPSPWAMDTSLP
TPSPMDQIRTLPTTNNNRKRKLSHPEEDRGACKKFLSSRSISVPSAVPTPPQDKPMDLHLIVPRRSSSSGYGYEVTDS
STSQEEPMDLSCSRTRKQPEPEPVPEPEPEPKDPTAVVQESETTTTTTTTATTTTEEAEKEPVLKFSPFMGNIVITDI
TTNCLTVTFKEYVSV

>Sal_Cbx6 (GeneID: 112069553)
MELSAVGERVFAGEAILNTRVRKVSTAVFLSLQKSRRGRGSPRRASRARTEDLSFSSKMTKQDSIPANLLTQYWEPEE
NILDGRLIAGFEQKERERELHEPKKRGPNPKTSCQGTEPRRKRPHTRAPRSTSSSSPRAPPSFPSASPAPSPKLNSMA
XAHKLKKDIRRCHLRMSRQPLYRRTDPLATPGSFSGPSGFPSRPPVSPFSETVRILNRRVKPREVKRGRIILNLNVID
KXGSGGGGRGGGGNRRAPSGSMSLQGGRQNIPSRNRIIGKRSGEAPYRPFQPMLGFPMYGKPFGLQCGGPGPLHLPHS
ATGSSTGTSGARDTQPPPSPSSSSGSTTPAQNHPTPPGPATGASGSAPNLGAQASASSAQSTPSHPPELQKNQNAEQG
PNPLLPSLSFASGSSSSSFDEEEDDADALDLSAPQEGKRKLRRRRRQKRQPPSVSTASPPHPPGACPLTSNSQRVPAE
GDPDWHPEMVASSANVVVTDVTTNLLTVTIKEFPSPSSRPSSPQPIHPENTTSSSSSSPPPPPPPLKNP

>Sal_LOC111977976
MELSAIGEQVFAVESIIKKRVRKGNVEYLLKWKGWPPKYSTWEPEEHILDQRLVQAYEEKDQKDRALGYWKRGPKAKR
LLLQNTIYTMDLRSAHKAPEKPPARLRLSLTRSLDSEAEDPTYVACRRSLHPHLDHHKNKPRRSQFVRLASPPGPPTP
TQDPTHEDWGRREEEDEEEDMEEEEARQDESERETERSSGILNVQNRAGDWSSVIGSEEVTVSERSDVWNPVIGPGEV
TVTDVTINSLTVTFREALAAK

>Sal_LOC111967894
MELSAIGEQVFAVESIIKKRVRKGNVEYLLKWKGWPPKYSTWEPEEHILDQRLVQAYDEKEQKDRALGYRKRGPKAKR
LLLQNTIYNMDLRSAHKALEKPQARLRLSLTRSLESEAEDPTYGACRRSLHPRLAHRKNKPRRSQFMCLASSPGPPNP
TKDPTHDWGRREEEDDMEETRQEQSERETEICSGILNGQDRAEDWSSVIGSDEVTSSERSAVWSPVIGPGEVTVTDVT
INSLTVTFKEALAAKGFFRGWGLEF

>Sal_LOC112075019
MELSSIGEQVFAVESITKKRVRKGNVEYLLKWQGWPPKYSTWEPEDHILDPRLVLAYEEKEEKDRALAYRRKGLRPRR
LVLRNIYAMDLRSAHKVPDTPRLRLSLTRSMGSELDQGSLPXRAGEGGSVYRRLARRKNKQRVSKPVSDNNSLQPLTR
IQDPMEHEWQGAEERPESELTTDTRHSRSECSSPMMEQEVESTTDRVESCGSALGSGDETLGGGALLTVTGAGYSSDG
```

GTSETGQEQMDRTDQSESCVSAEGPKYLNISNRLVDSATEVELGTDTLIDKVERLGTANAIERVEGLGTDYFIDRVEE
MGTHILIDRDSTSVVDVGDETVSDGSVVCTDVEVAEEVVENDTKQQGEEVVTQCPDSSGTEVNPGKVIVTDVTINSLT
VTFKEALAAEGFFKG

>Sal_LOC112071830
MELSSIGEQVFAVESITKKRVRKGNVEYLLKWQGWPPKYSTWEPEDHILDPRLVLAYEEKEEKDRALAYRRKGLRPRR
LILRSSLLLQNIYAMDLRSAHKVPDTPRLRLSLTRSMGSELDQGSLPXRAGEGGSVYRRLARRKNKQRVPKPVSDNNS
LQPLTRIQDPMEHEWQGAEERPESELTTDTRHSQSECSSPMMEQEVESSTDRVESCGSALGSGDETLGGGALLRVTGA
GYRSEGRTSEPGQEQIDRTDQSESCVSAEGPKDNISNRLVDSATEVELGTDTLIDKVERLGTANVIEGLGTYYFIDSV
EEMGTHILIDRDSTSVVDVGDKTVSDGSVVCTDVEVAEEVVENDTKQQSEEVVTQCPDSSGAEVNPGKVIVTDVTINS
LTVTFKEALAAEGFFKG

>Sal_LOC111956622
MELSAVGERVFAAESIIKRRIRRGRMEYLVKWKGWSPKFSTWEPEENILDSRLFVAFEERERERELFGPKKRGPKPKT
FLLKAQAKAKAKSYEFRSDAVRGIRVTYPTPEPVITPRAREGLRAVVPTIFPPSTVNRGESVRVRPPEPVREHRQPVL
QRPPGPDGFVIVPKKRGPKPKLRFKDNPFNAPPAAPEPPKRRADEQATYGPLKLAKLGLSGGEERGSEIRGNKLAHRH
QEELGGYPHKQIRPVASGSTQQHCVPDRSMLHSHRIGSDSQACRTKECPSNYLSPPKLKHLSKKNVHQPSEELPQRGK
PSLIAKIPVSRILGQTDEVTWKPCLNNVEKVLVTDVTTNFLTVTIRESSTDQGFFKDKR

>Sal_LOC111954549
MELSAVGERVFAAESIIKRRIRRGRMEYLVKWKGWSPKFSTWEPEENILDSRLFVAFEERERERELFGPKKRGPKPKT
FLLKAQAKAKAKSYEFRSDAVRGIRVTYPTPEXVITPRAREGLRAVVPTIFPPSTVNRGESVRVRPPEPVREHRQPVL
QRPPGPDGFVIVPKKRGPKPKLRFKDNPFNATSAATEPHKRRAEEQATYGPLKLAKLGLSGGEERGSEMRGIKLAHRH
QVELGGYPHKQMRPVASGSTQQHYGPDRGLLHSHRIGSDSQACRTKECPSNYLSPPHLKHLSKKNVHQPSEELPQRGK
PSLIAKIPVSRILGQTDEVTWKPCMNNAEKVLVTDVTTNFLTVTIRESSTDQGFFKDKR

>Hhu_Cbx2 (ENSHHUG00000026817)
MEELSAVGEQVFDAECILNKRLRKGKLEFLVKWRGWSSKHNSWEPQANILDPRLLAAFNKSEQEKEILISKRGKRPRG
RPRKVVETVPEVSKSSSSSSSSSSSDSSSSSSSSSSSSSDDDADDNNDRKAKPGPRTRELHPVPQKKAQIVVAKPEPL
RKKRGRKALPAEMKAIQQNKGQRKIIKTVAKDSPADFRGGIKKPFHPASFTFMGLNSRGPLSVQGRCSLAQGGTTKNS
MNTAGSGRSNTSASLSFNQRSNQSKSQASDFKLSVSDVGSGAGLDLKTTASKSPGVAVLNLHNSKLSTSNGNPQGAFQ
PQLGSHNGQKKPDATGQTPLQQVPNNKPSAPFSTPKGPANQAASLQALNLQSVNKSTQGNGTPGNGTAPVSNLRSTVN
PARKDTVGQYGQEQNPVQSPAMPGRQQPIK
NHPGVDKVKAEETSEVGVTAERPERLTTTRAQGRVEKSIVQNPSAEARDILGKRERSASKDSGKQAKVLLSEMSTGEE
STSDSDQDSPYPSNGQDLSISVQTGQDWKPTHSLIEHVFVTDVTANLVTVTVKESPTSVGFFNIHNY

>Hhu_ENSHHUG00000050943
MEELSAVGEQVFDAECILNKRLRKGKLEFLVKWRGWSSKHNSWEPQANILDPRLLAAFNKSEQEKEILICKRGKRPRG
RPRKVVETVPEVSKSSSSSSSSSSSGSSSSSSSSSSSSSEDDADDNNDRKAKPGPRTRELHPVPQKKAQIVVAKLEPP
RKKRGRKALPAEMKAIQQNKGQRKIIKTVAKDSPADLRGGIKKPFHPASFTFMGLNSRGPLNVQGRCSLAQGGTTKNS
MNTAGSGRSNTSASLSFNQRSNQSKSQASDFKLSVSDVGSGAGLDLKMTASKSPGVTVLNLHNSKLSTSNVNPQGAFQ
PQLGSHKGQKKPDAPGQTPLQQVPNNKHAASFSTPKGPANQAASLQALNLQSVNKSTQGNGTPGNGTVPVSNLRSTVN
PARKDTVGQYGQEQNPVQSPATPGGQQPRK
NHPGVDKVKAEETSEVGVTAERPERLTTTRAQGRVEKSIVQNPSAEARDILGKRERSASKDRGKQAKVLLSEMSTGEE
STSDSDQDSPYPSNGQDLSISVQTGQDWKPTHSLIEHVFVTDVTANLVTVTVKESPTSVGFFNIHNY

>Hhu_ENSHHUG00000045610
MEGVTVGHVFDAECILNKRPRKGKFEYLVKWRGWSSKHNSWEPEENILDPRLLAAFHKRAQERELLFRKRGKRPRGRP
RKILEPEPTETKSDRSSSSSSSSSGLSSSPSSSSSEEEEEDHGKKAKPGPRLRNLYPVPQKRPQIVVAKNEPVRKRRG
RKPLLPELRVLRQAKTRPPLPPPSPSRHHQVLRLPREPFKEEPRGGVKKPLQPASFTYTGLSSSRGSRDEVAHQGAAG
GAFYQAGASKPGPLNSIWSGRSISTNTATPSSPSSSSSLSRPPQSYSKGWSDLKRSLSVSDAGSINGRAEGLKVASSL
KPGVSTSTSTSLCLHSSKTSSGFGGSLAVCSPAQRFPAGQRRQQEGPGGQTGMVVQQQGAMPPSSTPPSARDRISQALSL
RALNLQSVKKIAPGNGLQGNGTSGTTGVAV
SRLSLRSSASPAGKRAGGSIKETHTSSGLSQGLVSGGLGGGALHSGSVPAARVEREERGKDTGAEMEREMDNKGPGCV
GRGNGRVEKGGSVQAQGGVSTARRGGANGGRQEDRKSGQSLTLNERNSRSGDNARTLNELSTGDSDDTSSSESEECDS
SPYPDNNNNGARLVSMEMETETDWRPARSLLEHVFVTDVTANFVTVTVKESPTSVGFFNVRNH

>Hhu_ENSHHUG00000028893
MEGVTVGQVFDAECILNKRPRKGKFEYLVKWRGWSSKHNSWEPEENILDPRLMAAFHKREQERELLFGKRGKRPRGRP
RKILEPEPTETKSDRSSSYSSSGLSSSPSASSSEEEDEDHGKKAKPGPRRRNLNLHPVLQTRPHIVVANQEPIRKKRG
RKPLLPELRALRQANTRPPLPPPPLSRHHQVLRPPREPPKEEPRGGVKKPLQPASFTYTSLSASRGSRDETAPQRAAG
GAFFQAGASKPGLLNSIWSGRSMSPATPSSPSSSSSLSRPPPTHSKGLSDLKRSLSVSDAGNSSGRAEGLKVASSVKP
GVSTSTSLGLHSSKTSSGFGGSPAACSPAQRSPAGQRRQQEGPGSQAVVVVQQQGAKPSSSPPPSARDRISQALSLRA
LNLQSVNKRAPGNGLQGYGTSGTTGVAVSRSSLRSGASQAGKGAGGSIKEMHTSSGLDQSLASGGLGAGVLRSGSIPQ
AREERGERGKTTGAETEREMDSKGPRSVGRGNGRLEKGGSVQGGVSSAGGGGSSAAREEDRKSGQSLNERNSRSVENS
RILNELSTGDSDNTSSSESEGRDSSPYPNNNGPRLVSMETEMETDWRPALSLLEHVFVTDVTANFVTVTVKESPTSVG
FFNVRNP

>Hhu_Cbx4 (ENSHHUG00000045583)
MELPAAGEHVFAVEGIEKKRIRKGKIEYLVKWRGWSPKYNTWEPEENILDPRLLVAFQNRERQEQLMGYRKRGPKPKH
LLVQLPSFARRSSIPAGFEETSPEAENHLKSDPIQMHRSQPQQYQLNSKKHHQYQPSKQEVPADQLGNGKKKLFYQLN
SKKHHHYQPDPNMYNTQSARPKDVVKGQEPSPPTNPNPGWNLPPALQQKWVRDKNTGCLSKVKDVTVVEMKKAAVRVN
EAESECALKPSPKEAALPSAVSSKMKIIKNKNKNGRIVIVMSKYMDGNKAGAAKGKHSSDLTGEEKPQHAEQSDMTKV
QGTTNVPKEICNASSPPAAEHPKKRSAEDGHFSKPSPSTAEEYNTEVARGQADLPDDHPLQLTTNPTPSPWAMDTSLP
TPSPMDQIRTLPTTNNDRKRKLSHPEEDRGACKRFLSSRSNSVPSAVPTPPQDKPMDLHLIVPRRSSSSGYGYEVTDT
STNQEEPMDLSCSRTRKQPEPEPVPEPEPEPKDPTAVVQESVTTTMTTTAATTTEEAEEEPVLKFSPFMGNIVITDI
TTNCLTVTFKEYVSV

>Hhu_ENSHHUG00000007216
MELPAAGEHVFAVEGIEKKRIRKVKLLTNAHFNSVELNSNVIGFLTKSLSFSFLLQGKIEYLVKWRGWSPKYNTWEPE
ENILDPRLLVAFQNRERQEQLMGYRKRGPKPKHLLVQLPSFARRSSIPAGFEETSPEAENHLRSDPIQMHRSQPQQYQ
LNSKKHHQYQPSKQEVPADQLGNGKKKLFYQLNSKKHHHYQPDPNMYNTQSARSKDVVKGQEPSPPTNPNPGWNLPPA
LQQKWVRDKNTGCLSKVKDVTVVEMKKSAVRVNEAESECALKPSPKEAALPSAVSSKMKIIKNKNKNGRIVIVMSKYM
DGNKAGAAKGKHSSDLTGEEKPQHTEQSDMTKVQATTNVPKEICNASSPPAAEHPKKRSPEDGHFSKPSPSTAEEYNT
EVARGQADLPDDHPLQLTTNPTTSPWAMDTSLPTPSPMDQIRTLPTTNNDRKRKLSHPEEDRGACKKFLSSRSISVPS
AVPTPPQDKPMDLHLIVPRRSSSSGYGYEVTDTSTNQEEPMDLSCSRTRKQPEPEPVPEPVPEPEPEPKDPTAVVQES
VTTTTTTIATTTEEAEEEPVLKFSPFMGNIVITDITTNCLTVTFKEYVSV

>Hhu_ENSHHUG00000045521
MELPAAGEHVFAVESIEKKRNRKGRMEYLVKWRGWSPKYNTWEPEENILDPRLLDAFQNRERYEQLMGYRKRGPKPKH
LMGQVPSFARRSSVLSGLQETTLDEDNQQKVDPIQTVRSQTQQYQLNSKKHHQYQPLLAKEREAEQQANGKKKLYYQL
NSKKHHHYQPDPKMYDSTQCMKPREATKAPELPTNHSWNLPPALQQKWVRDKDSGCLSKVKDITMELKKLPAHLNGHS
GELEQSKATAKEANGVSDSKLKIVKNKNKNGRIVIVMSKYMENGMQAARIKNGDVETLDELQPDNDDDAAENQLEKMR
LVQKLGLTNGFAKDCNKGSNRSNVFKCPADKVDSPKMELSATEQNKHFNVRGQKEATEDQPLQLTTKPNLGPWPFEMG
VHRRTHPGNVSSPALKRHLSEADKGEDRGGCKQFLNSRSISAPCTVSSPPQSNSMHQNGHHSHNGHQDYNFLESNQDE
PMDLSCVRSRREAPVPTETQVAPAEEEKTAEQTKPPLAITTEQTVEEEPFPSFKPFLGNIVITDITTNCLTVTFKEYV
TV

>Hhu_ENSHHUG00000050487
MEYLVKWRGWSPKYNTWEPEENILDPRLLDAFQNRERYEQLMGYRKRGPKPKHLIGQVPSFARRSSVLSGLQETTVDE
DNRQKVDSIQTLRSQTQQYQLNSKKHHQYQPLLAKEREAEQQANGKKKLYYQLNSKKHHHYQPDPKMYDSTQCMKPRE
ATKAPELPTNHSWNLPPALQQKRVRDKDSGCLSKVKDITMELKKLPAHLNDHSGNSEQSKATAKEDASMLKANDVSDS
KLKIVKNKNKNGRIVIVMSKYMENGMQAARIKNGDVETLDELQPDNDDDAAENQLEKMRLIQKLGLTNGFAKDCNKDV
KLHVPNSGSNVFKCPAEKVDSPKMELSATEQPLQLTTKPNLAPWPFEMGVHRRTQPRNGSSPALKRHLSEADKGEDRG
GCKRFLNSRSISAPCTVSSLPQSKSMDQNGHHSHNGHQDYDFLESNQDEPMDLSCVRSRREAPVPTETQVAPSAEEEK
TAEQTKPPLAITTEHTVEEEPFPSFKPFLGNIVITDITTNCLTVTFKEYVTV

>Hhu_Cbx6 (ENSHHUG00000007276)
MELSAVGERVFAAEAILKRRVRKSRLEYLVKWKGWALKHSTWEPEENILDDRLIAGFEQKEREREELHGPKKRGPKPKN
LPAKARGAQKRETSTTRAPRSTSSSSPRAPPSSSHPSFPSSAPVPSPKLNSLAATHKLKKDIRRCTSMSRRPLPRSDP
LASPGSFSGPSGFPSRPPVSPFSETVRILNRRVKPREVKRGRIILNLKVIDKPGSGGGGRGGGGNRRVPSGSMSLQGG
RQNIPSRNRIIGKRSGEAPYRPFQPMLGFPMYGKPFGLQCGGPGPLHLPHSATGSSTGTSGARDTQPPPSPSSSSGST
TPAPNHPGPDAQPATGTSASGSAPNMSAQASASSAQSTPSNPPELQKNQNAEQGPNPLLHSLSFASGSSSSSFDEEEE
DADALDLSAPQEGKRKLRRRRLQKRQPPSVSTASPPHPPAAGACPLTSNPQRVPAEGDPDWHPEMVASSANVVVTDVT
TNLLTVTIKEFPSPSSRPSSPQPIHPENTTTTEEPPTTQAIGSD

>Hhu_Cbx6a (ENSHHUG00000051753)
MELSAVGERVFAAEAILKRRVRKSRLEYLVKWKGWALKHSTWEPEENILDDRLIAGFEQKEREREELHGPKKRGPKPKN
LPAKARGAQKRETSTTRAPRSTSSSSPRAPPSSSHPSFPSSAPAPSPKLNSLAATHKLKKDIRRCTSMSRRPLPRSDP
LASPGSFSGPSGFPSRPPVSPFSETVRILNRRVKPREVKRGRIILNLKVIDKPGSGGGGRGGGGNRRVPSGSMSLQGG
RQNIPSRNRIIGRRGGEAPYRPFQPMLGFPMYGKPFGLQCGGPRPLHLPHSATGSSTGTSGARDTQPPPSPSSSSGST
TPASSHPTPLGPEAQPATGASASGSAPNLSAQASASSAQSTPSNPPELQKNQNAEQGPNPPLPSLSFASGSSSSSFDE
EEDHADALDLSAPQEGKRKLRHRRRQKRQPPSVSTASPPHPPAAGACPLTSNPQRVPAEGDPDWHPEMVASSANVVVT
DVTTNLLTVTIKEFPSPSSCPSSPQPIHPENTTTTEEPPTTQAIGRD

>Hhu_ENSHHUG00000038356
MELSSIGEQVFAVESITKKRVRKGNVEYLLKWQGWPPKYSTWEPEDHILDPRLVLAYEEKEEKDRALAYRRKGLRPRR
LVLRKIYAMDLRSAHKVPDTPRLRLSLTRSMGSELDQGSLPYRAGEGGSVYRRLARRKNKQRVSKPVSDNNSLQPLTR
IQDPMEHEWQGAEERPESELTTETRHSRSECSSPMMEQEVESSTDRVESCGSALGSGDETLGGGALLRVTGAGDRSEG
GTSETGQEQMDRTDQSESCVSAEGPKYLNINNRLVDSATELELGTDTLIDKVERLGAANVIERVEGLGTDYFIDRVEE
MGTHILIDRDSTSVVDVGDETVADGSVVCTDVEVAEEVVENDTKQQGEEVVTQCPDSSGADVNPGKVIVTDVTINSLT
VTFKEALAAEGFFKG

>Hhu_ENSHHUG00000052693
MCGSYAVYLYVTPTGGEQRTGRWIGGNVEYLLKWQGWPPKYSTWEPEDHILDPRLVLAYEEKEEKDRALAYRRKGLRP
RRLVLRKIYAMDLRSAHKVPDTPRLRLSLTRSMGSELDQGSLPYRAGEGGSVYRRLARRKNKQRVSKPVSDNNSLQPL

TRIQDPMEHEWQGAEERPESELTTETRHSRSECSSPMMEQEVEESTDRVESCGSALGSGDETLGGGALLRVTGGEDRS
EGGTSETGQEQTDRTDQSESCVSAEGPKYLNISNRLVDSATEVELGTDTLIDKVERLGAANVIERVEGLGTDYFIDRV
EEMGTHILIDRDSTSVVDVGDETMADGSVVCTDVEVAEEVVENDTKQQGEEVVTQCPDSSGAEVNPGKVIVTDVTINS
LTVTFKEALAAEGFFKG

>Hhu_Cbx7b (ENSHHUG00000042715)
MELSAIGEQVFAVESIIKKRVRKGNVEYLLKWKGWPPKYSTWEPEEHILDQRLVQAYEEKEQKDRALGYRKRGPKAKR
LLLQNTIYTMDLRSAHKAPEKPPARLRLSLTRSLDSEAEDPTFGACRRSLYPHLAHKNKPRKSQFVRLASPPGPPTPT
QDPTHTDWGRREEEDEDMEEKEARQDESERETEMSSGILNGQNRAEDWSSVIGSEEVTVSERSDVWNPVIGPGEVTVT
DVTINSLTVTFREALAAKGFFRGWGLEF

>Hhu_ENSHHUG00000046898
MELSAIGEQVFAVESIIKKRVRKGNVEYLLKWKGWPPKYSTWEPEEHILDQRLVQAYDEKEQKDRALGYRKRGPKAKR
LLLQNTIYNMDLRSAHKALEKPPARLRLSLTRSLESEAEDPTYGACRRSLHPRLAHRKNKPRRSQFMCLATPPGPPNP
TNDPTHDDWGRREEEDDMEEEEETRQEQSERETEICSGILNGQDRAEDWSSVIGSDEVNASERSAVWSPVIGPGEVTV
TDVTINSLTVTFKEALAAKGFFRGWGLEF

>Hhu_Cbx8a (ENSHHUG00000045580)
MELSAVGERVFAAESIIKQRIRRGRMEYFVKWKGWSKKYSTWEPEENILDARLFAAFEEREREREELFGPKKRGPKPET
FLLKAKAKATAKTYECRREMPRGIRVSYPVLTPMITPRAREGLRAVVPTIFPPSTVNRGESVRVRPPETERRPQPAPP
PSFMAQEFVNIPKKRGPKPKLRLRFNMEPDSCPTEEPAKRSLLEEQQVPYGLSKMSRHCHHEGKTSERSVIQLTRRFQ
EGTSIVPKSNNAQRRVGTVSHSGTHSHDGRLVHKARLDHQSCGTLECPGGMSIPHPKLKHVSKKHFYRASDSSSMQQS
RPIVVTKSPALRSSGEQSAVSWRPRLDNVEKVVVTDVTTNFLTVTIKESSTDKGFFKDKR

>Hhu_ENSHHUG00000007201
MELSAVGERVFAAESIIKQRIRRGRMEYLVKWKGWSKKYSTWEPEENILDARLFAAFEEREREREELFGPKKRGPKPET
FLLKAKAKATAKTYECRREMPRGICVSYPVPTPMITPRAREGLRAVVPTIFPPSTVNRGESVRVRPPETERRPRPAPP
PSFMAEEFVNIPKKRGPKPKLHLRFNMEPDSCPTEEPAKRSRLEEQQVPYGLSKMSRHCHHEGKTSERSVIQLTRRFQ
EGTSIVPKSNNAQRRVGTVSHPGTHSHDGRLVHKARLDHQSRGTLKCPGAMSIPHPKLKHVSKKHFYRASDSSSSMQQ
SRPIVVTKSPALRSSGEQSAVSWRPRLDNVEKVVVTDVTTNFLTVTIKESSTDKGFFKDKR

>Hhu_Cbx8b (ENSHHUG00000050484)
MELSAVGERVFAAESIIKRRIRRGRMEYLVKWKGWSPKFSTWEPEENILDSRLFVAFEEREREREELFGPKKRGPKPKT
FLLKAQAKAKSYEFRSEAVRGIRVTYPTPEPIITPRAREGLRAVVPTIFPPSTVNRGESVRIRPPEPVREHRQPVLQR
PPGPDGFVIVPKKRGPKPKLRFKDNPFNATSAATEPPKRRAEEQATYGPLKLAKLGLSGGEERGSEMRGIKLAHRRQE
ELGGYPHKQMRPVASGSTQQHYGPDRGLLHSHKIGSDSQACRTKECPSNYLSPPHLKHLSKKNVHQPSEELPQRGKPS
LIAKIPVSRILGQTDVVTWKPCMNNMEKVLVTDVTTNFLTVTIRESSTDQGFFKDKR

Supplementary File S2. ***List of the protein sequences encoded by the teleost Pc orthologues identified in this study.*** Sequences of the chromodomain (SM000298) are highlighted in bleu, of the AT hook motif (SM000384) in green and of the Pc box (PF17218) in violet.
