# Supplementary material for: The Polycomb Orthologues in Teleost Fishes and Their Expression in the Zebrafish Model
Source: Genes (Basel). 2020 Mar 27;11(4):362. doi: 10.3390/genes11040362 (PMC7230241; doi:10.3390/genes11040362)
Supplement: Supplementary file 1 [file genes-11-00362-s001.zip › Supp Material/Supp File S3.pdf]

>Dre_cbx3a
MVNMGKKQAGKSKKEVQEIEEFVVEKVMDQRVVNGKVEFFLKWKGFTDADNTWEPEENLDCPELIAAFLESQKGV
VEKPEAVKRKSSTDEPETEESKAKRKKEMSDKPRGFARNLEPERIIGATDSSGELMFLMKWKDSDEADLVPAREA
NTRCPQIVIAFYEERLTWHSCPEDEQQ

>Dre_cbx3b
MRKKQNVKNRKAEETTVVQEFAVEKIIRRRVNNGKVEYYLKWKGFTDAENTWEPEDNLDCPELIEEYLRNLTVLG
QETEQEECQSLDHEVQPKEELSELAADMAHQQPQEELIERTNEEVEEHNAEIPVGQPGHPEPDCIIGCTDQQGEL
MFLIKWKNTDEVALLAAVEASKRWPQMVIRFYEDKLTWHGDDEQ

>Cca_LOC109108755
MRKKQNVKNRKAEETTVVQEFSVEKIIRRRVNDGKVEYYLKWKGFTDAENTWEPEDNLDCPELIEEFLRNLTVSG
ETEQEECQSLDHEVQPKQELTELDADTAHQQPQEELIERRNEEVNDHNAEIPAGQSSNPEPDCIIGCTDQQGELM
FLIKWKNTDEVALLSAGETSKRWPEMVIRFYEDKLTWHGEDEQ

>Cca_LOC109110995
MSVDMGKKQAGKSKKDVQEVEEFVVEKVIDQRLVNGKVEFFLKWKGFSDADNTWEPEENLDCPELISAFLESQKG
VVEKPEAEKRKSSTDEPETDESKAKRKKEMSDKPRGFARNLEPERIIGATDSSGELMFLMKWKDSDEADLVPAKE
ANTRCPQIVIAFYEERLTWHSCPEDEQQ

>Cca_LOC109072345
MSVDMGKKQAGKSKKDVQEVEEFVVEKVIDQRLVNGKVEFFLKWKGFSDADNTWEPEENLDCPELISAFLESQKG
VVEKPEAEKRKSSTDEPETDESKAKRKKEMSDKPRGFARNLEPERIIGATDSSGELMFLMKW

>Cca_LOC109111778
MGKKQGGKSKKEVQEVEEFVVEKVIDQRVVNGKVEFFLKWKGFTEADNTWEPEENLDCPELIAAFLESQKGVVEK
PEAVKRKSSTDEPETDESKAKRKKEMSDKPRGFARNLEPERIIGATDSSGELMFLMKWKDSDEADLVPAREANTR
CPQIVIAFYEERLTWHSCPEDEQQ

>Cca_LOC109049867
MRKKQNVKNRKAEETTVVQEFSVEKIIRRRVNDGKVEYYLKWKGFTDAENTWEPEDNLDCPELIEEFLRNLTVSG
ETEQEGCQSLDLEVQPKQELTELDADMAHQQPQEELIERGNEEVDDHNAEIPAGQSSNPEPDCIIGCTDQQGELM
FLIKWKNTDDVALLSAGETSKRWPEMVIRFYEDKLTWHGEDEQ

>Cau_LOC113116259
MRKKQNVKNRKAEETTVVQEFSVEKIIRRRVNDGKVEYYLKWKGFTDAENTWEPEDNLDCPELIEEFLRNLTVSG
ETEQEGCQSLDLEVQPKQELSELDADMAHQQPQEELTERGIEEVDDHSAEIPAGQSRNPEPDCIIGCTDQQGELM
FLVKWKNTDDVALLSAGETSKRWPEMVIRFYEDKLTWHGEDEQ

>Cau_LOC113060165
MRKKQNVKNRKAEETTVVQEFAVEKIIRRRVTGGKVEYYLKWKGFTDAENTWEPEDNLDCPELIEEFLRNLTVSG
ETEQEGCQSLDHEVQPKQELTQLDADMAHQEELIKMGNEEVNDHNAEIPVDQSGNPEPNCIIGCTDQQGELMFLI
KWKNTDEVALLSAGETSKRWPEMVIRFYEDKLTWHGEDEQ

>Cau_LOC113120071
MGKKQGSKSKKDVQEVEEFVVEKVIDQRLVNGKVEFFLKWKGFTDADNTWEPEENLDCPELISAFLESQKGVVEK
PEAEKRKSSTDEPETDESKAKRKKEMSDKPRGFARNLEPERIIGATDSSGELMFLMKWKDSDEADLVPAKEANTR
CPQIVIAFYEERLTWHSCPEDEQQ

>Cau_LOC113062284
MGKKQGGKSKKEVQEVEEYVVEKVIDQRVVNGKVEFFLKWKGFTDADNTWEPEENLGCPELIAAFLESQKGVVEK
PEAVKRKSSTDEPETEESSAKRKKEMSDKPRGFARSLEPERIIGATDSSGELMFLMKWKDSDEADLVPAKEANTR
CPQIVIAFYEERLTWHSCPEDEQQ

>Sgr_LOC107602402
MGKKQGGKSKKDVQEVEEFVVEKVIDQRVVNGKVEFFLKWKGFTDADNTWEPEENLGCPELIAAFLESQKGVVEN
PEAIKRKSSTDEPETDESVAKRKKEMSDKPRGFARNLEPERIIGATDSSGELMFLMKWKDSDEADLVPAREANTR
CPQIVIAFYEERLTWHSCPEDEQQ

>Sgr_LOC107550827

MRKKQNVKNRKAEETTVVQEFAVEKIIRRRVNDGKVEYYLKWKGFTDVENTWEPEDNMDCPELIEEFLRNLTVSG
ETEQEGCRALDHEVQSKQELTELDADTAHQQPQEELIERGNEEVDDHSAEIPAGQSSNPEPDGIIGCTDQQGELM
FLIKWKNTDEVALLSAGETSKKWPEMVIRFYEDKLTWHGEDEQ

>Sgr_LOC107596654
MRKKQNVKNRKAEETTVVQEFAVEKIIRRRVNDGKVEYYLKWKGFTNAENTWEPEDNLDCPELIEEFLRNLTVSG
ETEREGCQSLDLEVQPKQELTELDANMAHQQPQEELMEKGNEEVDDHNAEIPAGQSSNPEPDCIIGCTDQQGELM
FLIKWKNTDEAALLSAGEASKRWPEMVIRFYEDKLTWHGEDEQ

>Sgr_LOC107592008
MGKKQGSKTKKDVQEVEEFVVEKVIDQRLVNGKVEFFLKWKGFTDADNTWEPEDNLDCPELISAFLESQKGVVEN
PEAVKRKSSTDEPETDESKAKRKKEMSDKPRGFARNLEPERIIGATDSSGELMFLMKWKDSDEADLVPAKEANTR
CPQIVIAFYEERLTWHSCPEDEQQ

>Srh_LOC107744695
MRKKQNVKNRKAEETTVVQEFAVEKIIRRRVNDGKVEYYLKWKGFTDVENTWEPEDNMDCPELIEEFLRNLTVSG
ETEQEGCQSLDHEVQSKQELTELDADMAHQQPQEELIERGNEEVDDHNAEIPAGQSSNPEPDGIIGCTDQQGELM
FLIKWKNTDEVALLSAGETSKKWPEMVIRFYEDKLTWHGEDEQ

>Srh_LOC107710359
MGKKQGGKSKKDVQEVEEFVVEKVIDQRVVNGKVEFFLKWKGFTDADNTWEPEENLGCPELIAAFLESQKGVVEN
PEAVKRKSSTDEPETDESAAKRKKEMSDKPRGFARNLEPERIIGATDSSGELMFLMKWKDSDEADLVPAREANTR
CPQIVIAFYEERLTWHSCPEDEQQ

>Srh_LOC107748667
MGKKQGSKTKKDVQEVEEFVVEKVIDQRLVNGKVEFFLKWKGFTDADNTWEPEDNLDCPELISAFLESQKGVVEK
PEAVKRKSSTDEPETDESKAKRKKEMSDKPRGFARNLEPERIIGATDSSGELMFLMKWKDSDEADLVPAKEANTR
CPQIVIAFYEERLTWHSCPEDEQQ

>Srh_LOC107743152
MRKKQNVKNRKAEETTVVQEFAVEKIIRRRVNDGKVEYYLKWKGFTNAENTWEPEDNLDCPELIEEFLRNLTVSG
EIEQEGCQSLDLEVQPMQELTELDANMAHQQPQEELIEKGNEEVGDHNAEIPAGQSSNPEPDCIIGCTDQQGELM
FLIKWKNTDEVALLSAGEASKRWPEMVIRFYEDKLTWHGEDEQ

>San_LOC107688340
MGKKQGGKSKKDVQEVEEFVVEKVIDQRVVNGKVEFFLKWKGFTDADNTWEPEENLGCPELIAAFLESQKGVVEN
PEAVKRKSSTDEPETDESAAKRKKEMSDKPRGFARNLEPERIIGATDSSGELMFLMKWKDSDEADLVPAREANTR
CPQIVIAFYEERLTWHSCPEDEQQ

>San_LOC107684532
MRKKQNVKNRKAEETTVVQEFAVEKIIRRRVNDGKVEYYLKWKGFTDVENTWEPEDNMDCPELIEEFLRNLTVSG
ETEQSLDHEVQSKQELTELDADTAHQQPQEELIERGNEDVDDHNAEIPAGQSSSPEPDGIIGCTDQQGELMFLIK
WKNTDEVALLSAGETSKKWPEMVIRFYEDKLTWHGEDEQ

>San_LOC107685582
MRKKQNVKNRKAGETTVVQEFAVEKIIRRRVNDGKVEYYLKWKGFTNAENTWEPEDNLDCPELIEEFLRNLTVSG
ETEQEGCQSLDLEVQPKQELTELDANMAHQQPQEELIEKGNEEVDDHNAEIPAGQSSNPEPDCIIGCTDQQGELM
FLIKW

>San_LOC107656668
MGKKQGSKTKKDVQEVEEFVVEKVIDQRLVNGKVEFFLKWKGFTDADNTWEPEDNLDCPELISAFLESQKGVVEK
PEAVKRKSSTDEPETDESKAKRKKEMSDKPRGFARNLEPERIIGATDSSGELMFLMKWKDSDEADLVPAKEANTR
CPQIVIAFYEERLTWHSCPEDEQQ

>Cva_LOC107101725
MRKKQTAKQRKTEETSVVQEFVVEKIIRRRVLNGRVEYFLKWKGFTDADNTWEPEDNLDCPGLIQEFLRDADLSK
EAEEEQSEQQVIKEDMAEQETEIQQSNNSVLKPDDEESDAPTDLSNYLEPECIIGSTDRKGELMFLVKWKNSDDV
ALLSAREASARCPQVVIDFYEQKFTWDCRDEKQ

>Cva_LOC107084325
MGKKQNTKPRKENQTQEEPEEFVVEKVIDQRIVNGKVEFYLKWKGFTDADNTWEPEENLDCPELISAFLEAQKNI
KEKPAAVKRKASTEEPETDSSKKTDAESPRGFARNLDPERIIGATDSSGELMFLMKWKGSDEADLVPAREANKRC
PQVVISFYEERLTWHSCPEDEAQ

>Xma_LOC106699776
MRKKQTAKQRKTEETTEVQEFVVEKIIRRRIFNGRVEYFLKWKGFTDAENTWEPEDNLDCPGLIEEFLRDAHLSR
EAEEEQSEQEFTPKEEMAKQETEISHVTPQEQNSSVLKPDDESDAPTDLSTYLEPECIIGSTDRKGELMFLVKWK
NSDEVALMPAREASARCPQVVIDFYEQKLTWHCGEGEQ

>Xma_LOC102224488
MGKKQNTKTKKEAQTQEEPEEFVVEKVMDQRIVNGKVEFYLKWKGFTDADNTWEPEENLDCPELISAFLESQKNI
KEKPTALKRKASTEEGETDSAKKIEAEQPRGFARNLNPEKIIGATDSSGELMFLMKWKDSDEADLVPAREANTRC
PQVVIAFYEERLTWHSCPEDEAQ

>Pfo_LOC103145272
MGKKQNTKTKKEAQTQEEPEEFVVEKVMDQRIVNGKVEFYLKWKGFTDADNTWEPEENLDCPELISAFLESQKNI
KEKPSAVKRKASTEEGETDSAKKTETEQPRGFARNLNPEKIIGATDSSGELMFLMKWKDSDEADLVPAREANTRC
PQVVIAFYEERLTWHSCPEDEAQ

>Pfo_LOC103150371
MRKKQTAKQRKTEETTEVQEFVVEKIIRRRIFNGRVEYFLKWKGFTDAENTWEPEDNLDCPGLIEEFLRDAHLSR
EVEEEQSEQEFIPKEEMAKQETEISHVTPQEQNSGVLKPDDEESDAPTDLSTYLEPECIIGSTDRKGELMFLVKW
KNSDEVALMSAREASARCPQVVIDFYEQKLTWHCGEGEQ

>Gaf_cbx3a (ENSGAFG00000017896)
MGKKQNTKTKKEAQTQEEPEEFVVEKVMDQRIVNGKVEFYLKWKGFTDADNTWEPEENLDCPELISAFLESQKNI
KEKPVAVKRKASTEEGETDSAKKIEAEQPRGFARNLNPEKIIGATDSSGELMFLMKWKDSDEADLVPAREANTRC
PQVVIAFYEERLTWHSCPEDEAQ

>Gaf_cbx3b (ENSGAFG00000005602)
MRKKQTAKQRKTEETTEVQEFVVEKIIRRRIFNGRVEYFLKWKGFTDAENTWEPEDNLDCPGLIEEFLRDAHLSR
EVEEEQSEEEFIPKEEMAKQETEISHVTPQEQNSSVLKPDDESDAPTDLSTYLEPECIIGSTDRKGELMFLVKWK
NSDEVALMSAREASARCPQVVIDFYEQKLTWHCGEGEQ

>Pre_LOC103472202
MGKKQNTKTKKDAQTQEEPEEFVVEKVMDQRIVNGKVEFFLKWKGFTDADNTWEPEENLDCPELISAFLESQKTI
KEKPAAVKRKASTEEGETDSAKKTETEQPRGFARNLNPEKIIGATDSSGELMFLMKWKDSDEADLVPAREANTRC
PQVVIAFYEERLTWHSCPEDEAQ

>Pre_LOC103478581
MRKKQTAKQRKTEETTEVQEFVVEKIIRRRIFNGRVEYFLKWKGFTDAENTWEPEDNLDCPGLIEEFLRDAHLSR
EVEEEQSEQEFIPKEEMAKQETEISHVTPQEQNSSVLKPDDEESDAPTDLSTYLEPECIIGSTDRKGELMFLVKW
KNSDEVALMSAREASARCPQVVIDFYEQKLTWHCGEGEQ

>Xco_LOC114141930
MRKKQTAKQRKTEETTEVQEFVVEKIIRRRIFNGRVEYFLKWKGFTDAENTWEPEDNLDCPGLIEEFLRDAHLPR
EAEEEQSEQEFTPKEEMAKQETEISHVTPQEQNSSVLKPDDESDAPTDLSTYLEPECIIGSTDRKGELMFLVKWK
NSDEVALMPAREASARCPQVVIDFYEQKLTWHCGEGEQ

>Xco_LOC114156510
MGKKQNTKTKKEAQTQEEPEEFVVEKVMDQRIVNGKVEFYLKWKGFTDADNTWEPEENLDCPELISAFLESQKNI
KEKPTAVKRKASTEEGETDSAKKIEAEQPRGFARNLNPEKIIGATDSSGELMFLMKWKDSDEADLVPAREANTRC
PQVVIAFYEERLTWHSCPEDEAQ

>Pla_LOC106945372

MGKKQNTKTKKEAQTQEEPEEFVVEKVMDQRIVNGKVEFYLKWKGFTDADNTWEPEENLDCPELISAFLESQKNI
KEKPSAVKRKASREEGETDSAKKTETEQPRGFARNLNPEKIIGATDSSGELMFLMKWKDSDEADLVPAREANTRC
PQVVIAFYEERLTWHSCPEDEAQ

>Pla_LOC106950175
MRKKQTAKQRKTEETTEVQEFVVEKIIRRRIFNGRVEYFLKWKGFTDAENTWEPEDNLDCPGLIEEFLRDAHLSR
EVEEEQSEQEFIPKEEMAKQETEISHVTPQEQNSGVLKPDDEESGAPTDLSTYLEPECIIGSTDRKGELMFLVKW
KNSDEVALMSAREASARCPQVVIDFYEQKLTWHCGEGEQ

>Pme_LOC106923566
MGKKQNTKTKKEAQTQEEPEEFVVEKVMDQRIVNGKVEFYLKWKGFTDADNTWEPEENLDCPELISAFLESQKNI
KEKPSAVKRKASTEEGETDSAKKTETEQPRGFARNLNPEKIIGATDSSGELMFLMKWKDSDEADLVPAREANTRC
PQVVIAFYEERLTWHSCPEDEAQ

>Pme_LOC106928233
MRKKQTAKQRKTEETTEVQEFVVEKIIRRRIFNGRVEYFLKWKGFTDAENTWEPEDNLDCPGLIEEFLRDAHLSR
EVEEEQSEQEFIPKEEMAKQETEISHVTPQEQNSGVLKPDDEESDAPTDLSTYLEPECIIGSTDRKGELMFLVKW
KNSDEVALMSAREASARCPQVVIDFYEQKLTWHCGEGEQ

>Nfu_cbx3 (Gene ID: 107383357)
MGKKQNTKPKKETETQEEPEEFVVEKVLDQRIVNGKVEFYLKWKGFTEADNTWEPEDNLDCPELIQAFLDSQKNI
KEKPAAVKRKASTDDAEEEPKKKDVPDKPRGFARNLEPERIIGATDSSGELMFLMKWKESDEADLVPAREANTRC
PQVVISFYEERLTWHSCPEDEAQ

>Nfu_LOC107395330
MRKKQTAKQRKTEEPAEVQEFVVDRIIRRRVFDGRVEYFLKWKGFTDAENTWEPEDNLDCPELIEEFLRSAPPTE
DEQSEQQFRPKEEMTEQDVELSSEQSNCSNGVPKQDDEQSDAPINLSTYLEPECIIGSTDRKGELMFLVKWKNSD
DVALLPAREASLRCPQVVIDFYEQKLTWHCGDEEP

>Fhe_LOC105926367
MRKKQTAKQRKTEETAEVQEFVVEKIIRRRICNGRVEYFLKWKGFTDADNTWEPEDNLDCPGLIEEFLRDADLSG
ETEEERSEQQFIPREEMAEQETEISCVRSQQQSNNSVLKPDDEASDAPTNLSTYLEPECIIGSTDRQGELMFLVK
WKNSDEFALLSAREASARCPQVVIDFYEQKLTWHSDNKGQ

>Fhe_LOC105934310
MGKKQHTRPKKEAQAQEEPEEFVVEKVIDQRIVNGKVEFYLKWKGFTEADNTWEPEENLDCPELISAFLEAQKNI
KEKPAAVKRKASTEEVEADPAKKTEAEKPRGFARNLDPERIIGATDSSGELMFLMKWKESDEADLVPAREANTRC
PQVVISFYEERLTWHSCPEDEAQ

>Kma_cbx3 (Gene ID: 108238543)
MGKKQNTKSKKDDQTQEEPEEFVVEKVLDQRLVNGKVEFFLKWKGFTDADNTWEPEENLDCPELIQAFLDSQKNV
KEKPAPVKRKASTDEPESESKKKDVEEKPRGFARNLEPERIIGATDSSGELMFLMKWKDSDEADLVPAREANTRC
PQVVISFYEERLTWHSCPEDEA

>Kma_LOC108241914
MRKKQTAKPRKAEETAEVQEFVVEKIIRRRVSNGRVEYFLKWKGFTDAENTWEPEDNLDCPELIEEFLRDAPLQE
ENEEEPNEPCFVPKEEMTEQETEISSVQSGGGGGGDGGGVLQADDDQPDGPVNLSTYLEPECIIGSTDRKGELMF
LVKWKNSDDVALLPAREASARCPQVVIDFYEQKLSWNVGDEEQ

>Mze_cbx3 (Gene ID: 101470889)
MGKKQNTKSKKDAQDTQEEPEEFVVEKVLDQRVVNGKVEFYLKWKGFTDADNTWEPEENLDCPELISAFLEAQKN
VKEKPAPVKRKASTEEPETEPKKKDVAEKPRGFARNLEPERIIGATDSSGELMFLMKWKDSDEADLVPAREANTR
CPQVVISFYEERLTWHSCPEDEAQ

>Mze_LOC101483327
MRKKQTAKQRKTEETTVVQEFVVEKIIRRRISNGRVEYFLKWKGFTDAENTWEPEDNLDCPELIEEFLRNAHFTE
ENEEEQPEQGFVPKEEMAEEETEISCVQEAQAVKTDNSVLQPDDEQKNSPSDLSTYLEPECIIGSTDRKGELMFL
VKWKNSDDVALLPAREASARCPQVVIDFYEQKLTWHCGDDEQ

>Oni_LOC100694541

MRKKQTAKQRKTEETTVVQEFVVEKIIRRRISNGRVEYFLKWKGFTDAENTWEPEDNLDCPELIEEFLRNAHFSE
ENEEEQTEQGFVPKEEMAEEETEISCVQEAQAVKTDNSVLQPDDEQTDSPSDLSTYLEPECIIGSTDRKGELMFL
VKWKNSDDVALLPAREASARCPQVVIDFYEQKLTWHCGDDEQ

>Oni_LOC100694248
MGKKQNTKSKKDTQETQEEPEEFVVEKVLDQRVVNGKVEFYLKWKGFTDADNTWEPEENLDCPELISAFLEAQKN
VKEKPAPVKRKASTEEPETEPKKKDVAEKPRGFARNLEPERIIGATDSSGELMFLMKWKDSDEADLVPAREANTR
CPQVVISFYEERLTWHSCPEDEAQ

>Hbu_cbx3 (Gene ID: 102307347)
MRKKQTAKQRKTEETTVVQEFVVEKIIRRRISNGRVEYFLKWKGFTDAENTWEPEDNLDCPELIEEFLRNAHFTE
ENEEEPPEQGFVPKEEMAEEETEISCVQEAQAVKTDNSVLQPDDEQTNSPSDLSTYLEPECIIGSTDRKGELMFL
VKWKNSDDVALLPAREASARCPQVVIDFYEQKLTWHCGDDEQ

>Hbu_LOC102308912
MGKKQNTKSKKDAQDTQEEPEEFVVEKVLDQRVVNGKVEFYLKWKGFTDADNTWEPEENLDCPELISAFLEAQKN
VKEKPAPVKRKASTEEPETEPKKKDVAEKPRGFARNLEPERIIGATDSSGELMFLMKWKDSDEADLVPAREANTR
CPQVVISFYEERLTWHSCPEDEAQ

>Nbi_LOC102791258
MRKKQTAKQRKTEETTVVQEFVVEKIIRRRISNGRVEYFLKWKGFTDAENTWEPEDNLDCPELIEEFLRNAHFTE
ENEEEQPEQGFVPKEEMAEEETEISCVQEAQAVKTDNSVLQPDDEQTNSPSDLSTYLEPECIIGSTDRKGELMFL
VKWKNSDDVALLPAREASARCPQVVIDFYEQKLTWHCGDDEQ

>Nbi_LOC102789869
MGKKQNTKSKKDAQDTQEEPEEFVVEKVLDQRVVNGKVEFYLKWKGFTDADNTWEPEENLDCPELISAFLEAQKN
VKEKPAPVKRKASTEEPETEPKKKDVAEKPRGFARNLEPERIIGATDSSGELMFLMKWKDSDEADLVPAREANTR
CPQVVISFYEERLTWHSCPEDEAQ

>Pny_cbx 3 (Gene ID: 102213635)
MGKKQNTKSKKDAQDTQEEPEEFVVEKVLDQRVVNGKVEFYLKWKGFTDADNTWEPEENLDCPELISAFLEAQKN
VKEKPAPVKRKASTEEPETEPKKKDVAEKPRGFARNLEPERIIGATDSSGELMFLMKWKDSDEADLVPAREANTR
CPQVVISFYEERLTWHSCPEDEAQ

>Pny_LOC102197024
MRKKQTAKQRKTEETTVVQEFVVEKIIRRRISNGRVEYFLKWKGFTDAENTWEPEDNLDCPELIEEFLRNAHFTE
ENEEEQPEQGFVPKEEMAEEETEISCVQEAQAVKTDNSVLQPDDEQTNSPSDLSTYLEPECIIGSTDRKGELMFL
VKWKNSDDVALLPAREASARCPQVVIDFYEQKLTWHCGDDEQ

>Aca_LOC113032446
MRKKQTAKQRKTEETTVVQEFVVEKIIRRRISNGRVEYFLKWKGFTDAENTWEPEDNLDCPELIEEFLRNAHFTE
ENEEEQPEQGFVPKEEMAEEETEISCVQEAQAVKTDNSVLQPDDEQKNSPSDLSTYLEPECIIGSTDRKGELMFL
VKWKNSDDVALLPAREASARCPQVVIDFYEQKLTWHCGDDEQ

>Aca_LOC113014873
MGKKQNTKSKKDAQDTQEEPEEFVVEKVLDQRVVNGKVEFYLKWKGFTDADNTWEPEENLDCPELISAFLEAQKN
VKEKPAPVKRKASTEEPETEPKKKDVAEKPRGFARNLEPERIIGATDSSGELMFLMKWKDSDEADLVPAREANTR
CPQVVISFYEERLTWHSCPEDEAQ

>Aci_cbx3a (ENSACIG00000002111)
MGKKQNTKSRKDTQETQEEPEEFVVEKVLDQRVVNGKVEFYLKWKGFTDADNTWEPEENLDCPELISAFLESQKN
VKEKPAPVKRKASTEEPETESKKKDVKPRGFARNLEPERIIGATDSSGELMFLMKWKDSDEADLVPAREANTRCP
QVVISFYEERLTWHSCPEDEAQ

>Aci_cbx3b (ENSACIG00000016058)
MRKKQTAKQRKTEETTVVQEFVVEKIIRRRILNGRVEYFLKWKGFTAENTWEPEDNLDCPELIEEFLRNAHFSEE
NEDEQTEQESQQQAQAVKTDNSVLQPDDEQSHSPTDLSTYLEPECIIGSTDRKGELMFLVKRKNSDDVALLPARE
ASARCPQVVIDFYEQKLTWHCGDDEQ

>Tru_LOC101067559

MGKKQKTKRDSMETTEQPEEFVVEKILDQRLVNGKVEFYLKWKGFTDADNTWEPEENLDCPDLISAFLEAQKNIK
EKTAPKRKSTDEPETEAKKKDAEKPRGFARNLEPERIIGATDSSGELMFLMKWKGSDEADLVPAREANVRCPQIV
ISFYEERLTWHSCPEDEAQ

>Tru_LOC101061787
MRKKQTAKQRKTEETAVVQEFAVDKIIRRRVLNGRVEYFLKWKGFTDAENTWEPEDNLDCPELIEEFLRNTPFPD
GIEEGQEFVPKEEMTEQETEIPRPPSDGVVPEPDEERSDTHSDRSSYPEPECIIGSTDRQGELVFLIKWKNSEDV
ALLPAREASARYPQMVIDFYERKLTWHCGDEEQ

>Tni_ENSTNIG00000001200
MGKKQKTKKDSMETTTEQPEEFVVEKILDQRIVNGKVEFYLKWKGFTDADNTWEPEENLYCPDLISAFLEAQKNI
KEKSAPKRKLTDEPETEAKKKDAEKPRGFARNLDPERIIGATDSSGELMFLMKW

>Tni_ENSTNIG00000018425
MRKKQTAKQRKTEETAVVQEFAVDKIIRRRVVDGRVEYFLKWKGFTDAENTWEPEDNLDCPELIEEFLRNTRFPQ
EEGLELVPKEEMTEQEAEMVSVRTNAKVEELSFALLEPDEEQTDPRDDPGASPEPECIIGSTDRQGELVFLVKWK
NSEDVGLLPAREASVRYPQMVIGFYERKLAWHCGDDEQ

>Mmo_cbx3a (ENSMMOG00000000435)
MGKKQKTKRDSQGNQEEPEEFVVEKVLDQRLVNGKVEFYLKWKGFTDASLVWGCPEENLDCPELISAFLEAQKNI
TEKPAAVKRKASTDEPETEAKKKDVAEKPRGFARNLEPERIIGATDSSGELMFLMKWKDSDEADLVPAREANTRC
PQVVISFYEERLTWHSCPEDEVQ

>Mmo_cbx3b (ENSMMOG00000001662)
MRKKQTAKQRKTEETAVVQEFVVEKIIRRRIFNGRVEYFLKWKGFTDAENTWEPEDNLDCPELIEEFLRNAIYVH
LEKPSLNRGGGLRHHLSNLEPNDDHSDAPANLSTYLEPECIIGSTDRQGELMFLIKWKNSDDVALLPAREASARC
PQMVIDFYEQKLTWHCGDEEQ

>Lcr_LOC104940580
MGKKQNTKGKKDSQESQEEPEEFVVEKVLDQRIVNGKVEFFLKWKGFTDADNTWEPEENLDCPELISGFLEAQKN
VKEKPAPVKRKASTDEPETEAKKKDTEKPRGFARNLDPERIIGATDSSGELMFLMKWKDSDEADLVPAREANTRC
PQVVISFYEERLTWHSCPEDEAQ

>Lcr_LOC104927770
MRKKQTAKQRKTEETTVVQEFVVEKIIRRRVFNGRVEYFLKWKGFTDAENTWEPEDNLDCPELIEEFLRNAHFSE
EIEEEQSEQELIPKEEMTEQETEISCMQSQQAHTVQSNSDVLEPNDEQSDTPTNLTTYLEPECIIGSTDRQGELM
FLVKWKNSDDVALLPAREASARCPQVVIDFYEQKLTWHCGDEEQ

>Pfl_cbx3 (Gene ID: 114567991)
MHNMGKKQNTKGNVKKDAQDTQEEPEEFVVEKVLDQRLVNGKVEFFLKWKGFTEADNTWEPEENLDCPELISAFL
EAQKNVKEKPAPVKRKASTDEPETEAKKKDAEKPRGFARNLDPERIIGATDSSGELMFLMKWKDSDEADLVPARE
ANTRCPQVVISFYEERLTWHSCPEDEAQ

>Pfl_LOC114557836
MRKKQTAKQRKTEETPTVVQEFVVEKIILRRIFNGRVEYFLKWKGFTDAENTWEPEDNLDCPELIEEFLRNTHFS
EVNEEERSEQEFIPKEEMTEQETEISFMQSQQSNSEPNDKQSDSPTNLNTYLEPECIIGSTDRQGELMFLVKWK
NSDDVALLPAREASVRCPQVVIDFYEQKLTWHCGDEEQ

>Sdu_LOC111223223
MGKKQNTKGKKDAQETQEEPEEFVVEKVLDQRIVNGKVEFYLKWKGFTDADNTWEPEENLDCPELISAFLEAQKN
ITEKPAPVKRKASTEEPETEAKKKDVAEKPRGFARNLEPERIIGATDSSGELMFLMKWKDSDEADLVPAREANTR
CPQVVISFYEERLTWHSCPEDEAQ

>Sdu_LOC111238784
MRKKQTAKQRKTEETTVVQEFVVEKIIRRRISNGRVEYFLKWKGFTDAENTWEPEDNLDCPELIEEFLRNTHLSE
ENEEEQTEQEFVPKEEMTEQETEISQRQTHTLQSDSNVLEPNDKQSDAPTNLSTYLEPECIIGSTDRQGELMFLV
KWKNSDDVALLPAREASARCPQVVIDFYEQKLTWHCGDEEQ

>Sla_LOC111673325

MGHAGSRRRHDYSLWRPSCRAADEIKKWPREHNMGKKQNTKGKKDAQETQEEPEEFVVEKVLDQRIVNGKVEFYL
KWKGFTDADNTWEPEENLDCPELISAFLEAQKNITEKPAPVKRKASTEEPETEAKKKDVQPEKPRGFARNLEPER
IIGATDSSGELMFLMKWKDSDEADLVPAREANTRCPQVVISFYEERLTWHSCPEDEAQ

>Sla_LOC111652798
MRKKQTAKQRKTEETTVVQEFVVEKIIRRRISNGRVEYFLKWKGFTDAENTWEPEDNLDCPELIEEFLRNTHLLS
EENEEEQTEQEFVPKEEMTEQETEISQRQTHTLQSDSNVLEPNDKQSDAPTNLSTYLEPECIIGSTDRQGELMFL
VKWKNSDDVALLPAREASARCPQVVIDFYEQKLTWHCGDEEQ

>Lbe_cbx3 (Gene ID: 109994620)
MGKKQNTKGKKDAQETQEEPEEFVVEKVLDQRIVNGKVEFFLKWKGFTDADNTWEPEENLDCPELISGFLEAQKN
VKEKPAPVKRKASTDEPETEAKKKDVPEKPRGFARNLEPERIIGATDSSGELMFLMKWKDSDEADLVPAREANTR
CPQVVISFYEERLTWHACPEDEAQ

>Lbe_LOC109986704
MRKKQTAKQRKTEVTTVVQEFVVEKIIRRRIFNGRVEYFLKWKGFTDAENTWEPEDNLDCPELIEHFLNTHFSEE
NEEEQTEVGFIFKEEMTEQETEISCLQSQQRTPIMQTNSDVLEQNDEQSDTPTNLSTYLEPECIIGSTDRQGELM
FLVKWKNCDEVALLPAREASARCPQVVIDFYERKLTWHCGEEEL

>Cgo_LOC115021151
MGGRAVPDHWTRNGLLSDWSAVRSSPSSPPGFPGMTFSLGNHFLRASTAIFNLKMRKKQTAKQRKTEETPVVQEF
VVEKIILRRIFNGRVEYFLKWKGFTDAENTWEPEDNLDCPELIEEFLRNTHYLNEEEQNVQELIPKEEMTEQETE
ISYMQSQQQAHTEQSNSEPNDEQSETPTNLSTYLEPECIIGSTDKQGELMFLVKWKNSDDVALLPAREASARCPQ
VVIDFYEQKLTWHCGDEEQ

>Cgo_LOC115016275
MGKKQNTKGKKDAQDTQEEPEEFVVEKVLDQRLVNGKVEFFLKWKGFTEADNTWEPEENLDCPELISAFLEAQKN
VKEKPAPVKRKASTDEPETEAKKKDVAEKPRGFARNLDPERIIGATDSSGELMFLMKWKDSDEADLVPAREANTR
CPQVVISFYEERLTWHSCPEDEAQ

>Ena_LOC115051003
MGKKQNTKGKKDAQETQEEPEEFVVEKVLDQRIVNGKVEFFLKWKGFTDADNTWEPEENLDCPELISAFLESQKN
ITEKPAPVKRKASSDEPETEAKKKDVAEKPRGFARNLEPERIIGATDSSGELMFLMKWKDSDEADLVPAREANTR
CPQVVISFYEERLTWHSCPEDEVQ

>Ena_LOC115045440
MRKKQTAKQRKTEETTVVQEFVVEKIIHRRISNGRVEYFLKWKGFTDAENTWEPEDNLDCPELIEEFLRNTHLSD
ENEEEQTEQDFVPKEEMTEQETEISQQQAHTLQSDGDVLEPNDEQSDAPTNLSTYLEPECIIGSTDRQGELMFLV
KWKNSDDVALLPAREASARCPQVVINFYEQKLTWHCGDEDQ

>Pra_cbx3 (Gene ID: 114450982)
MGKKQNTKTKKDSSEAQEEPEEFVVEKVLDQRLVNGKVEFFLKWKGFTDADNTWEPEENLDCPELISAFLESQKN
IKEKPAPIKRKASTDEAETDAKKKDVEKPRGFARNLEPERIIGATDSSGELMFLMKWKDSDEADLVPAREANTRC
PQVVISFYEERLTWHSCPEDEAQ

>Pra_LOC114448349
MRKKQTAKQRKTEETTDVQEFVVEKIIRRRIFNGRVEYFLKWKGFTDAENTWEPEDNLDCPELIEEFLRNTQISE
ENEEEQTEFVPKEEMTEQETEISCVQQTCAVQSDGSVLQPDDQPSDSPTNLSTYLEPECIIGSTDRKGELMFLVK
WKNSDDVALLPAREASARCPQVVIDFYEQKLTWHCGDEEQ

>Lcr_LOC108891497
MRKKQTAKQRKTEETTVVQEFVVEKIIRRRVSNGRVEYFLKWKGFTDAENTWEPEDNLDCPELIEEFLRNTNLSE
ENEEERTDQEFIPKEEMTEQETEISCMQSQRQAHAVQSDSDVLEPNDEQSDAPPNLSTYLEPECIIGSTDRQGEL
MFLVKWKNSDDVALLPAREVSARCPQVVIDFYEQKLTWHCGDEEQ

>Lcr_LOC108877463
MGKKQNTKGKRDSQESQEEPEEFVVEKVLDQRIVNGKVEFFLKWKGFTDADNTWEPEENLDCPELISAFLESQKN
IKEKPAPVKRKASTDEPETEAKRKDVAEKPRGFARNLDPERIIGATDSSGELMFLMKWKDSDEADLVPAREANTR
CPQVVISFYEERLTWHSCPEDEAQ

>Spa_cbx3 (Gene ID: 103364037)

MRKKQTAKQRKTEETTVVQEFVVEKIIRRRISNGRVEYYLKWKGFTDAENTWEPEDNLDCPELIEEFLRNAHLSD
EEEEFIPKEEMTEQETEISCVQQAHAVHSNEDIVLQPDDEQSDAPANLSTYLEPECIIGSTDRKGELMFLVKWKN
SDDVALLPAREASARCPQVVIDFYEQKLTWHCGDEEQ

>Spa_LOC103364624
MGKKQNTKGKKDAQETQEEPEEFVVEKVLDQRLVNGKVEFYLKWKGFTDADNTWEPEENLDCPELISAFLEAQKN
VKEKPAPVKRKASTDEPETESKKKDVAEKPRGFARNLEPERIIGATDSSGELMFLMKWKDSDEADLVPAREANTR
CPQVVISFYEERLTWHSCPEDEAQ

>Apo_LOC110955790
MGKKQNTKTKKDAQETQEEPEEFVVEKVLDQRLVNGKVEFFLKWKGFTDADNTWEPEENLDCPELISAFLEAQKN
VKEKPAPVKRKASTDEPETESKKKEVAEKPRGFARNLEPERIIGATDSSGELMFLMKWKDSDEADLVPAREANTR
CPQVVISFYEERLTWHSCPEDEAQ

>Apo_LOC110951430
MRKKQTAKQRKTEETTVVQEFVVETIIRRRVFNGRVEYYLKWKGFTDAENTWEPEDNLDCPELIEEFLRTYNPED
DEVFIPKEEMSEQETEISCVQEAHAVQSNSSLVLQPDEEQSDAPANLSTYLEPECIIGSTDRKGELMFLVKWKNS
DDVALLPAREASARCPQVVIDFYEQKLTWHSGDEEQ

>Aoc_LOC111579711
MGKKQNTKTKKDAQDTQDEPEEFVVEKVLDQRLVNGKVEFFLKWKGFTDADNTWEPEENLDCPELISAFLEAQKN
VKEKPAPVKRKASTDEPETESKKKDVAEKPRGFARNLEPERIIGATDSSGELMFLMKWKDSDEADLVPAREANTR
CPQVVISFYEERLTWHSCPEDEAQ

>Aoc_LOC111588833
MRKKQTAKQRKTEETTVVQEFVVETIIRRRVFNGRVEYYLKWKGFTDAENTWEPEDNLDCPELIEEFLRTYNPED
DEVFIPKEEMTEQETEISCVQEPHAVQSNSSLVLQPDEEQSDAHTNLSTYLEPECIIGSTDRKGELMFLVKWKNS
DDVALLPAREASARCPQVVIDFYEQKLTWHSGDDEQ

>Ape_cbx3a (ENSAPEG00000019335)
MGKKQNTKTKKDAQDTQEEPEEFVVEKVLDQRLVNGKVEFFLKWKGFTDADNTWEPEENLDCPELISAFLEAQKN
VKEKPAPVKRKASTDEPETESKKKDVAEKPRGFARNLEPERIIGATDSSGELMFLMKWKDSDEADLVPAREANTR
CPQVVISFYEERLTWHSCPEDEAQ

>Ape_cbx3b (ENSAPEG00000010544)
MRKKQTAKQRKTEETTVVQEFVVETIIRRRVFNGRVEYYLKWKGFTDAENTWEPEDNLDCPELIEEFLRTYNPED
DEVFIPKEEMTEQETEISCVQEPHAVQSNSSLVLQPDEEQSDAPTNLSTYLEPECIIGSTDRKGELMFLVKWKNS
DDVALLPAREASARCPQVVIDFYEQKLTWHSGDDEQ

>Nco_cbx3 (Gene ID: 104949902)
MRKKQTAKQRKTEETPVVQEFVVEQIILRRIYNGRVEYFLKWKGFTDADNTWEPEDNLDCPELIQEFLRNTHFFN
EEEQNEQEFIPKEEMTEQETEISCMQSLQQTHTVQSNSEPNDEQSDTPTNLSTHLEPECIIGATDRKGELMFLVK
WKNSDDVALLPAREASARCPQVVIDFYEQKLTWHCGDEEQ

>Nco_LOC104966666
MAKKQNTKGKKDAQDPQEEPEEFVVEKVLDQRLVNGKVEFFLKWKGFTEADNTWEPEENLDCPELISAFLEAQKN
VKEKPAPVKRKLSTEEPESEAKKKDVPEKPRGFARNLDPERIIGATDSSGELMFLMKWKDSDEADLVPAREANTR
CPQVVISFYEERLTWHSCPEDEAQ

>Cse_LOC103394257
MGKKQNKKDGQDTQQEPEEFVVEKVLDQRIFNGKVEFFLKWKGFTEADNTWEPEENLDCPELISAFLEAQKNIKE
KPATVKRKLSADEPESEAKKKEAEKPRGFARNLDPERIIGATDSSGELMFLMKWKNSDEADLVPSREANVRCPQV
VISFYEERLTWLRR

>Cse_LOC103388431
MRKKQTAKQRKTEETAVVQEFAVEKIIRRRIFNGRVEYFLKWKGFTDADNTWEPEDNLDCPELIEEFLRNSHLSQ
QNEEEDTEFIPKEEMTEQETGIQRDHTLPTDSGVLEPDEEPSDLSAHLVPECIIGSTDRQGELMFLVKWKNSDDV
ALLPAREVSARSPQVVIDFYEQKLTWHCGEEEQ

>Sma_cbx3a (ENSSMAG00000014860)

MGKKQTIKGKKDSQETQEEPEEFVVEKVLDQRLVNGKVEFFLKWKGFTDADNTWEPEENLDCPELISAFLEAQKT
ITEKPAPMKRKASTDEPETEAKKKDVAEKPRGFARNLDPERIIGATDSSGELMFLMKW

>Sma_cbx3b (ENSSMAG00000020282)
MRKKQTAKQRKTEETTVVQEFVVEKIIRRRVFNGRVEYFLKWKGFTDAENTWEPEDNLDCPELIEEFLRNTHCLE
ENEEEPTQFIPKEEMTEQETEISCLQSQQQVCALQSDGGVLLEPNGDEQSDAPANLSNYLEPECIIGSTDRQGEL
MFLVKWKNSDDVALLPAREASARCPQVVIDFYEQKLTWNCGDEEQ

>Ame_LOC103046426
MGKKQLTKSKKEVEEPEEFVVEKVIDQRVVNGKVEFFLKWKGFPDTDNTWEPEENLDCPELISAFLTSQKGVVEN
PDSNKRKSSTDEPETDENSAKRKKELSEKPRGFARNLEPERIIGATDSSGELMFLMKWKDSDEADLVPAREANTR
CPQVVIAFYEERLTWHSCPEDEQQ

>Pna_LOC108425284
MGKKQVNKSKKEVEEPEEFVVEKVLDQRVVNGKVEFFLKWKGFPDTDNTWEPEENLDCPELISAFLTSQKGVVEK
PDSNKRKSSTDEPETEENSAKRKKEMSEKPRGFARNLEPERIIGATDSSGELMFLMKWKDSDEADLVPAREANTR
CPQVVIAFYEERLTWHSCPEDEQQ

>Pna_LOC108441518
MRKKQNVKNRKAEETTVVQEFAVEKIIRRRVNDGKVEYYLKWKGFTDAENTWEPEDNLDCPELIEEFLRNMAVSG
EREDSNSLEPVIQPKEELTELDADTVHQQQCQSKQTESKTGQPGDHDTATADNRSTSPEPERIIGSTDRHGELMF
LIKWKDTDDVALLSAREASARWPQMVIGFYEDKLTWHGEEEP

>Mar_LOC113122625
MRKKQTAKQRKTEETTDVQEFVVEKIIRRRISDGRVEYFLKWKGFTDAENTWEPEDNLDCPELIEEFLRNSQLSG
ENEEEQPELIPKEELTEQETEISCVRPQLQPRTVQSGGDVLEPNSEPSGVHANLSTYLEPECIIGSTDRQGELMF
LVKWKNSDDVALLSAREASARCPQVVIDFYEQKLTWHCGDEEQ

>Mar_LOC113125891
MGKKQNTKTKKDTQEEEPEEFVVEKVLDQRVVNGKVEFFLKWKGFTDADNTWEPEENLDCPELISAFLESQKNIK
EKPAPVKRKASTDEPEPEAKKKDAEKPRGFARNLEPERIIGATDSSGELMFLMKWKDSDEADLVPAREANVRCPQ
VVISFYEERLTWHSCPEDEAQ

>Mal_LOC109968149
MGKKQNTKGKKDAQEEPEEFVVEKVLDQRIVNGKVEFYLKWKGFTDADNTWEPEENLDCPELISAFLEAQKSIKE
KPAPVKRKASTDEPEAQAKKKDMAEKPRGFARNLEPERIIGATDSSGELMFLMKWKDSDEADLVPAREANTRCPQ
VVISFYEERLTWHS

>Mal_LOC109967352
MRKKQTAKQRKTEETTLVQEFVVEKIIRRRVSNGRVEYFLKWKGFTDAENTWEPEDNLDCPELIGEFLRTNHVSE
ENDEEQTEQEFIPKEEPTEQETEISCMRPQQQAHTVSNDSDVLEPNSERSDATTNLSSYLEPECIIGSTDRQGEL
MFLVKWKNSDDVALLPAREASARCPQVVIDFYEQKLTWHCGDEEQ

>Ate_LOC113170961
MRKKQTAKQRKTEETVVQEFVVEKIIRRRVFNGRVEYFLKWKGFTDAENTWEPEDNLDCPELIEEFLRNSRLSEG
NEETDQELIPKEEMTEQETEISCMRPQQQIHTVPSDRGVLEPNDEHSDTPTNLSTYLEPECIIGSTDRQGELMFL
VKWKDSDDVALLPAREASARCPQVVIDFYEQKLTWHCGDEEQ

>Ate_LOC113153869
MGKKQNTKSKKDAQETQDEPEEFVVEKVLDQRVVNGKVEFFLKWKGFTDADNTWEPEENLDCPELISAFLESQKN
IKEKPAPVKRKASTDEPETDAKKKDVTEKPRGFARNLEPERIIGATDSSGELMFLMKWKDSDEADLVPACEANTR
CPQVVISFYEERLTWHSCPEDEAQ

>Bsp_LOC114865848
MGKKQNTKGKKDAQETQDEPEEFVVEKVLDQRVVNGKVEFFLKWKGFTDADNTWEPEENLDCPELISAFLESQKN
VKEKPAPVKRKASTDEPETDAGDAKKKEVTEKPRGFARNLDPERIIGATDSSGELMFLMKWKDSDEADLVPAREA
NTRCPQVVISFYEERLTWHSCPEDEAQ

>Bsp_LOC114843466

MRKKQTAKQRKTEETVVQEFVVEKIIRRRVFNGRVEYFLKWKGFTDAENTWEPEDNLDCPELIDEFLRNAHLSDE
NEETEQDLIPKEEMTEQETEISCMRPQSDGGGGGGALEPHDEPPDAPYLEPECIIGSTDRQGELMFLVKWKDSDD
VALLPAREASARCPQVVIAFYEQKLSWHCGDDEP

>Gwi_LOC114472522
MGKKQNSKSKDATGVQAEPEEFVVEKVIDQRLVNGKVEFFLKWKGFTEADNTWEPEENLDCPELISAFLEAQKNI
KEKPGGVKRKASTEEPDTDGKKKESEKPRGFARNLDPERIIGATDSSGELMFLMKWKDSDEADLVPAREANTRCP
QVVISFYEERLTWHSCPEDEAQ

>Gwi_LOC114478298
MRKKQTAKQRKTEDTAVVQEFAVEKIMRRRVYNGRVEYFLKWKGFTDSAENTWEPEDNLDCPELIEDYLKNGHNL
ENEDDQCNEFTPKEEISMQETEIAHSQDVKSDSVALQPLNDPTDLSTYLEPECIIGSTDRKGELMFLVKWKNSDD
VALLSAREASTRCPQVVIDFYEQKLSWHCGDQEP

>Pma_cbx3a (ENSPMGG00000006267)
MGKKQNTKSKKDSSTSEEPEEFVVEKILDQRLVNGKVEFYLKWKGFTEADNTWEPEENLDXXXXQKNIKEKPAAV
KRKASTEEAEPEPKKTNEKPRGFARNLEPERIIGATDSSGELMFLMKWKDSDEADLVPAKEANKRCPQVVISFYE
ERLTWHSCPEDEAQ

>Pma_cbx3b (ENSPMGG00000011799)
MRKKQTAKQRKTEDSSVVQEFVVEKIIRRRVSNGRVEYFLKWKGFTDADNTWEPEDNLDCPELIEAFLVKNAHLT
ENSEEDQGEIIPKEEMTEQETEIVSTSNAELSDSKSHLSPRQEPECIIGSTDRRGELMFLVKWKNSEDVALLPAR
EVSARCPQVVINFYEQKLTWHCSEDEQ

>Sfa_LOC115396622
MRKKQTAKQRKTEETTVVQEFAVDQIIRRRVFNGRVEYFLKWKGFTDAENTWEPEDNLDCPELIEEFLRNSHFSE
QNEEEQAEFIPKEEMTERETEISCLQSQHQAQTMQSDSRILQPDEEHSDGPTDLSSYLEPECIIGSTDRKGELMF
LVKWKNSDDVALLPAHEASARCPQVVIDFYERKLTWHCREEEAAAEEEEQ

>Sfa_LOC115381000
MGKKQNTKGKRDSSETQEEPEEFVVEKVLDQRLVNGKVEFFLKWKGFTDADNTWEPEENLDCPELISAFLEAQKN
IKEKPATVKRKASTEEPEMDSKKKEVAEKPRGFARNLEPERIIGATDSSGELMFLMKWKDSDEADLVPSREANTR
CPQVVISFYEERLTWHSCPEDEAQ

>Ola_LOC101159942
MRKKQTAKQRKTEETTAVVQEFVVEKIIRRRVFNGRVEYFLKWKGFTDAENTWEPEDNLDCPELIEEFLRNAPAF
PEENEEEQSEQTLVPKEEMTEQETEIFSELASASHGDGYVLQSDDEQSDAPTDLSNYLEPECIIGSTDRKGELMF
LVKWKNSEDVALLPAREASTRCPQVVIDFYEQKLTWHCGDEEQ

>Ola_LOC100049459
MGKKQNTKSKKDSQDTQEEPEEFVVEKVLDQRLVNGKVEFYLKWKGFTDADNTWEPEENLDCPELISAFLESQKN
IKEKPAAVKRKASTDEPETEPKKKDMAEKPRGFARNLEPERIIGATDSSGELMFLMKWKDSDEADLVPAREANTR
CPQVVISFYEERLTWHSCPEDEAQ

>Gac_cbx3a (ENSGACG00000007069)
FAPDKFLVHNMGKKPNTKGKKDAHDTPEEPEEFVVEKVLDQRIVNGKVEFFLKWKGFTEADNTWEPEDNLDCPEL
ISAFLEAQKNVKEKPAPVKRKASTDEPETEAKKKDVAEKPRGFARNLDPERIIGATDSSGELMFLMKWKDSDEAD
LVPAREANTRCPQVVISFYEERLTWHSCPEDEAQ

>Gac_cbx3b (ENSGACG00000008334)
MRKKQTAKQRKTEETPVVQDFVVEKIIRRRIFDGRAEYFLKWKGFTDAENTWEPEDNLDCPELIDEFLRNTHFPE
GNEEDQRAERQLIPKEEMTEQETEIVSPGDRQSDTPTDLSAYREPECIIGSTDRQGELMFLVKWKNSSDVALLPA
REASARCPQVVIDFYEKKLTWHCGEEEQ

>Hco_LOC109522111
MGKKQNTKSRKDSQGGQGTQEEPEEFVVEKVLDQRIVNGKVEFFLKWKGFTEADNTWEPEENLDCPELINAFLEA
QKNIKEKPASLKRKAPADETEMDAKKKDAPEKPRGFARKLDPERIIGATDSSGELMFLMKWKDSDEADLVPAREA
NTKCPQVVIAFYEERLTWHSCPEDEAQ

>Hco_LOC109525650

MRKKQTAKQRKTEDSCDVQEFVVEKITRRRIFNGRVEYFLKWKGFTEADNTWEPEDNLDCPELIEEYLRNNPSNE
NEEVKQEFVPKEEMTEGTEISPPQIHTQPCGDDPDSPADLAAYPELECIIGSADRRGELMFLVKWKNSGDVALLP
AHEASTRCPQAVIDFYEQKLTWHCGDDEQ

>Pki_LOC111846950
MGKKQNGKSKKEMEQPEEFVVEKVIDQRIVNGKVEYFLKWKGFTDADNTWEPEENLDCPDLIGAFLATQKSAVEL
DSNKRKPSTDGTDSEESKTKKKKDVAEKPRGFARNLEPERIIGATDSSGELMFLMKWKDSDEADLVPAKEANVRC
PQVVIAFYEERLTWHSCPEDEQQ

>Pki_LOC111838987
MRKKQNMKHRKNEETTVVQEFVVEKIIQRRVSNGKVEYFLKWKGFSDADNTWEPEDNLDCPELIEEFLKSLAKNM
EVGEDLESPQPPKPLEVPADVEPQQTEQQPLAEHHGNALQEPWGQETERPASFTSHFEPERIIGSTDKQGELMFL
VKWKGTDQVALLPAREASTRCPKIVIAYYEEKLTWHSGDEDQQ

>Sfo_LOC108933414
MGKKQTSKSRKEMEQPEEFVVEKVLDQRIVNEKVEYLLKWKGFSEADNTWEPEENLDCPELIEEFLASQKSVAER
PDSNKRKQSTDEMETEEAKSKKKKDQSEKPRGFARNLEPERIIGATDSSGELMFLMKWKDSDEADLVPAKEANVR
CPQVVIAFYEERLTWHSCPEDEHQ

>Sfo_LOC108933440
KGRGFLAHVVTRSGARGEARPREKVCGKAVFNQIRFRRALVKKQASKKNKEMEQTQEFIVEKILDQRVVDGKVEY
LLKWKGFSDADNTWEPEENLNCPELIEAFLASQKCVVERPDSCKRRQSTDQMESEESKVKNVTKRPHGFARSLEP
ERIIGATDSSGELMFLMKWKDSNEADMVPAKEANMRCPQVVIAFYEERLSWNSCSEDEHC

>Sfo_LOC108927377
MRKKQNVKHRKTEETTVVQEFVVEKIIQRRVVNGKVEYFLKWKGFTDADNTWEPEDNLDCPELIEEFLRSLPGQG
DHNVEDLQLPEVPQLTEPAELDSQPTLQQPLEEPLQGVREPGDQETGSPTGLVPQYEPECIIGSTDRQGELMFLV
KWKGTEEVALMSAREASARCPQIVIAFYEEKLTWNSRDDDQQ

>Gmo_cbx3 (Gene ID: 115535606)
MGKKQNTKSAKKDVAEEPEEFVVEKVLDQRLVNGKVEFFLKWKGFTEADNTWEPEENLDCPELISAFLEAHKSIK
EKPATVKRKSSADESEPEAKKKDVSDKPRGFARNLDPERIIGATDSSGELMFLMKWKDSDEADLVPAREANSRCP
QVVISFYEERLTWHSCPEDEA

>Gmo_LOC115554036
MMRKKQTVRQRKPEETTMVQEFLVEKIIRRRVFNGRVEYFLKWKGFTDADNTWEPQDNLDCPELIDEFLRNMRLS
DNEEEQPQEFILNEELSEQEAEICSQYREQSDVVLDFQKRDVTGAGLDLDPDLELDLNREPECIIGSTDQHGELM
FLVKWKTSVSLLAAREASARCPQAVVDFYQRRLNWHSGDEDQ

>Ipu_cbx3 (Gene ID: 108271872)
MRKKQNVKNRKAEETTVVQEFAVEKIIRRRVNDGKVEYYLKWKGFTDAENTWEPEDNLDCPELIEEFLRNLAVSG
ETERKDNLPLDPVIQPKEEQTELDANIHRQQSEKEQTVCETGESTDHNTETTNDQSLIQEPERIIGSTDRQGELM
FLIKWKDTDDVALLSAREASVRWPKMVIGFYEDKLSYHEEEEQ

>Ipu_cbx3-like (Gene ID: 100528511)
MGKKQTSKSKKEMEEPEEFVVEKVMDQRLVNGKVEFFLKWKGFPDSDNTWEPEENLDCPELIAAFLESQKGVVEK
PDSNKRKSSTDEPETEESKAKKKKELNEKPRGFARNLEPERIIGATDSSGELMFLMKWKDSDEADLVPAREANTH
CPQVVIAFYEERLTWHSCPEDEQH

>Eel_LOC113571151
MRKKQNVKNRKAEETTVVQEFAVEKIIRRRVNDGKVEYYLKWKGFTDAENTWEPEDNLDCPELIEEFLRNLAVSG
ETGRGDSPAPEPVIQPKEELTEFDADQQSELIEREASDPGDCDPKAVAEQPPTPEPECIIGSTARHGELMFLVKW
RDMDDVALLSAREASSRWPQMVIGFYEEKLTWHAEEEQ

>Eel_LOC113580431
MYMRGRLVARLCFSSFQPRNALVPVVEEEVWLASCQEAPYKTPARLLHLCNRLYKANMGKKQLNKSKKEVEEPEE
FVVEKVLDQRVVNGKVEFFLKWKGFPESDNTWEPEENLDCPELIAAFLESQKGVVEKPDSNKRKPSTDEPETEES
KAKKKKETNEKPRGFARNLEPERIIGATDSSGELMFLMKWKDSDEADLVPAREANTRCPQVVIAFYEERLTWHSC
PEDEQQ

>Cha_cbx3 (Gene ID: 105907686)

MDVRPKRPLSQETAAMGKKQMGKSKKEVSEPEEFVVEKVMDQRVVNGKVEYFLKWKGFSDADNTWEPEENLDCPE
LIAAFHVAQKGVVEKPDSNKRKTSTDEPETEESKAKKKKESEKPRGFARNLEPERIIGATDSSGELMFLMKWKDS
DEADLVPAREANTRCPQVVISFYEERLTWHSCPEDEQQ

>Cha_LOC105911400
MRKKQNVKHRRPEEATVVVKHVQEFVVEKIIRRRVMDGKVEYFLKWKGFTDAENTWEPEDNLDCPELIEEFLRGL
ALGRNPSEEGGPLPQHQIQPKEEEEEVETERHQQCPAQALEPLQERTAIPECIMGRIEPECIMGHIEPGCIIGST
DRHGELTFLIKWKDSEEVALMSAREVSERNPQMVIAFYEERLRWTSADEEP

>Dcl_LOC114797297
MGKKQLTKSKKEVSEPEEFVVEKVIDQRVVNGKVEFFLKWKGFTDADNTWEPEENLDCPELISAFLDSQKGVVEK
PESNKRKPSTEEPESEENKAKKKKEANEKPRGFARNLEPERIIGATDSSGELMFLMKWKDSDEADLVPAREANTR
CPQVVISFYEERLTWHSCPEDEQQ

>Dcl_LOC114790202
MRKKQNVKHRKTEEKAVVQEFVVEKIIRRRVTDGRVEYFLKWKGFNDTDNTWEPEDNLDCPELIEEFLRGLSLSG
KAEGDDVPSLGSHIQLKEEQEDLETPIQDEPGDSGAQEPSEERTGIPEDYTSHLEPECIIGSTDKEGELMFLIRW
KDSEEVALMSAREASMRCPQMVISFYEEKLTWQSAEEEQQ

>Elu_LOC105023664
MGKKQNGKGKKEVLEADPPEEYVVEKVMDQRIVNGKVEFFLKWKGFTEADNTWEPEENLDCPELISAFLEAQKSV
VEKPDSNKRKSSTDEPETEENKAKKKKDVSDKPRGFARNLAPERIIGATDSSGELMFLMKWKDSDEADLVPAREA
NTRCPQVVISFYEERLTWHSCPEDEAQ

>Elu_LOC105018804
MRKKQNVKQRKAETITATIVTTPSESTTNTTPVVQEFVVEKIIRRRVSDGRVEYFLKWKGFNDADNTWEPEDNLD
CPELIEEFLRNLCLSGENHNEEENQFPVDPELVPKEELTEQETEISSQQVYEEQRKMELQEPGDQESDSPTDLNL
IGPLEPERIIGSTDRRGELMFLVKWKNSDEVALLSAREASARCPQVVVAFYEEKLTWHSGDEDQ

>Ssa_cbx3 (Gene ID: 100195583)
MKTSSPLVSQPNMGKKQNGKGKKEVLEAPPEEYVVEKVMDQRIVNGKVEFFLKWKGFTEADNTWEPEDNLDCPE
LISAFLEAQKNVVEKPDSNKRKSSTDEPESEENKAKKKKDVSDKPRGFARSLDPERIIGATDSSGELMFLMKWKD
SDEADLVPAREANTRCPQVVISFYEERLTWHSCPEDEAQ

>Ssa_LOC106588845
MGKKQNGKGKKEVLEAPPEEYVVEKVMDQRIVNGKVEFFLKWKGFTEADNTWEPEDNLDCPELISAFLEAQKTV
VENPDSNKRKSSTDEPETEGNKAKKKKDVSDKPRGFARSLDPERIIGATDSSGELMFLMKWKDSDEADLVPAREA
NTRCPQVVISFYEERLTWHSCPEDEAQ

>Ssa_LOC106573104
MKSFCKDTGTLLHLATHLTNHWTHSVVFVSACSSVCYIVAYFCHRQSWTESVRMRKKQNVKQRKAETITATIITP
TPLTTTTLSTTTPPVVQEFVVEKIIHRRVSNGRVEYYLKWKGFTDADNTWEPEDNLVCPELIEEFLRNLCLSGEN
QVEEENLQSVEPELVPKEELAEQETEIQVYSEQRHNDLQEPADRESDSPTALTCPLEAERIIGSTDRHGELMFLI
KWKNRDEVALLSAREASARYPEVVVAFYEDKLTWHSGDEDQ

>Omy_LOC110506239
MRKKQNVKQRKAETIITPTPLTTTTLSTTTTAAAVVQEFVVEKIIHRRVFNGRVEYYLKWKGFTDADNTWEPEDN
LVCPELIEEFLRNLCLSGENQVEEENLRPVEPELVPKEELAEQETEIQVYSEQRHNDLQEPADQDSPTALTCPLE
PADQDSPTALTCPLEPEHIIGSTERHGELMFLIKWKNRDEVALLSAREASARYPEMVVAFYEDKLTWHSGDEDQ

>Omy_LOC110496562
MGKKQNGKGKKEVLEAPPEEYVVEKVMDQRIVNGKVEFFLKWKGFTEADNTWEPEDNLDCPELISAFLEAQKTV
VENPDSNKRKSSTDEPETEGNKAKKKKDVSDKPRGFARSLDPERIIGATDSSGELMFLMKWKDSDEADLVPAREA
NTRCPQVVISFYEERLTWHSCPEDEAQ

>Omy_LOC110487945
MGKKQNGKGKKEVLEAPPEEYVVEKVMDQRIVNGKVEFFLKWKGFTEADNTWEPEDNLDCPELISAFLEAQKNV
VEKPDSNKRKSSTDEPETEENKAKKKKDVSDKPRGFARSLDPERIIGATDSSGELMFLMKWKDSDEADLVPAREA
NTRCPQVVISFYEERLTWHSCPEDEAQ

>Oki_LOC109878229
MRKKQNVKQRKAETIITPTPLTTTTLSTTTTAAAVVQEFVVEKIIQRRLYNGRVEYYLKWKGFTDADNTWEPEDN
LVCPELIEEFLRNLCLSGENQVEEENLRPVEPELVPKEELAEQETEIQVYSEQRHNDLQEPADQDSPTALTCPLE
PERIIGSTDRHGELMFLIKWKNRDEVALLSAREASARYPEVVVAFYEDKLTWHSGDEDQ

>Oki_LOC109907267
MGKKQNGKGKKEVLEAEPPEEYVVEKVMDQRIVNGKVEFFLKWKGFTEADNTWEPEDNLDCPELISAFLEAQKTV
VENPDSNKRKSSTDEPETEGNKAKKKKDVSDKPRGFARSLDPERIIGATDSSGELMFLMKWKDSDEADLVPAREA
NTRCPQVVISFYEERLTWHSCPEDEAQ

>Oki_LOC109903322
MGKKQNGKGKKEVLEAEPPEEYVVEKVMDQRIVNGKVEFFLKWKGFTEADNTWEPEDNLDCPELISAFLEAQKNV
VEKPDSNKRKSSTDEPETEENKAKKKKDVSDKPRGFARSLDPERIIGATDSSGELMFLMKWKDSDEADLVPAREA
NTRCPQVVISFYEERLTWHSCPEDEAQ

>Sal_LOC111955440
MGKKQNGKGKKEVLEAEPPEEYVVEKVMDQRIVNGKVEFFLKWKGFTEADNTWEPEDNLDCPELISAFLEAQKTV
VENPDSNKRKSSTDEPETEGNKAKKKKDVSDKPRGFARSLDPERIIGATDSSGELMFLMKWKDSDEADLVPAREA
NTRCPQVVISFYEERLTWHSCPEDEAQ

>Sal_LOC112070715
MGKKQNGKGKKEVLEAEPPEEYVVEKVMDQRIVNGKVEFFLKWKGFTEADNTWEPEDNLDCPELISAFLEAQKNV
VEKPDSNKRKSSTDEPETEENKAKKKKDVSDKPRGFARSLDPERIIGATDSSGELMFLMKWKDSDEADLVPAREA
NTRCPQVVISFYEERLTWHSCPEDEAQ

>Hhu_ENSHHUG00000039337
MTEVIGTLEDVVPVEEEEEEEEYVVEKVLNRRVVKGRVEYLLKWKGFSDDDNTWEPEDNLDCPDLIAQFLQKQKL
AHESVGKRKSAETSVEGEESRPKKRKDDPEKLRGFARGLDPERIIGATDSTGELMFLMKWKNSDEADLVPAKEAN
VKCPQVVISFYEERLTWHSYPTEEKEDDKN

>Hhu_cbx3a (ENSHHUG00000011830)
MGKKQNGKGKKEVLEAEPPEEYVVEKVMDQRIVNGKVEFFLKWKGFTEADNTWEPEDNLDCPELISAFLEAQKNV
VEKPDSNKRKSSTDEPESEENKAKKKKDVSDKPRGFARSLDPERIIGATDSSGELMFLMKWKDSDEADLVPAREA
NTRCPQVVISFYEERLTWHSCPEDEAQ

>Hhu_ENSHHUG00000050410
MGKKQNGKGKKEVLEAEPPEEYVVEKVMDQRIVNGKVEFFLKWKGFTEADNTWEPEDNLDCPELISAFLEAQKTV
VENPDSNKRKSSTDEPETEGNKAKKKKDVSDKPRGFARSLDPERIIGATDSSGELMFLMKWKDSDEADLVPAREA
NTRCPQVVISFYEERLTWHSCPEDEHV

Supplementary File S3. ***List of Cbx3 protein sequences encoded by the teleost genomes identified in this study.***
