# Supplementary material for: The Polycomb Orthologues in Teleost Fishes and Their Expression in the Zebrafish Model
Source: Genes (Basel). 2020 Mar 27;11(4):362. doi: 10.3390/genes11040362 (PMC7230241; doi:10.3390/genes11040362)
Supplement: Supplementary file 1 [file genes-11-00362-s001.zip › Supp Material/Supp File S5.pdf]

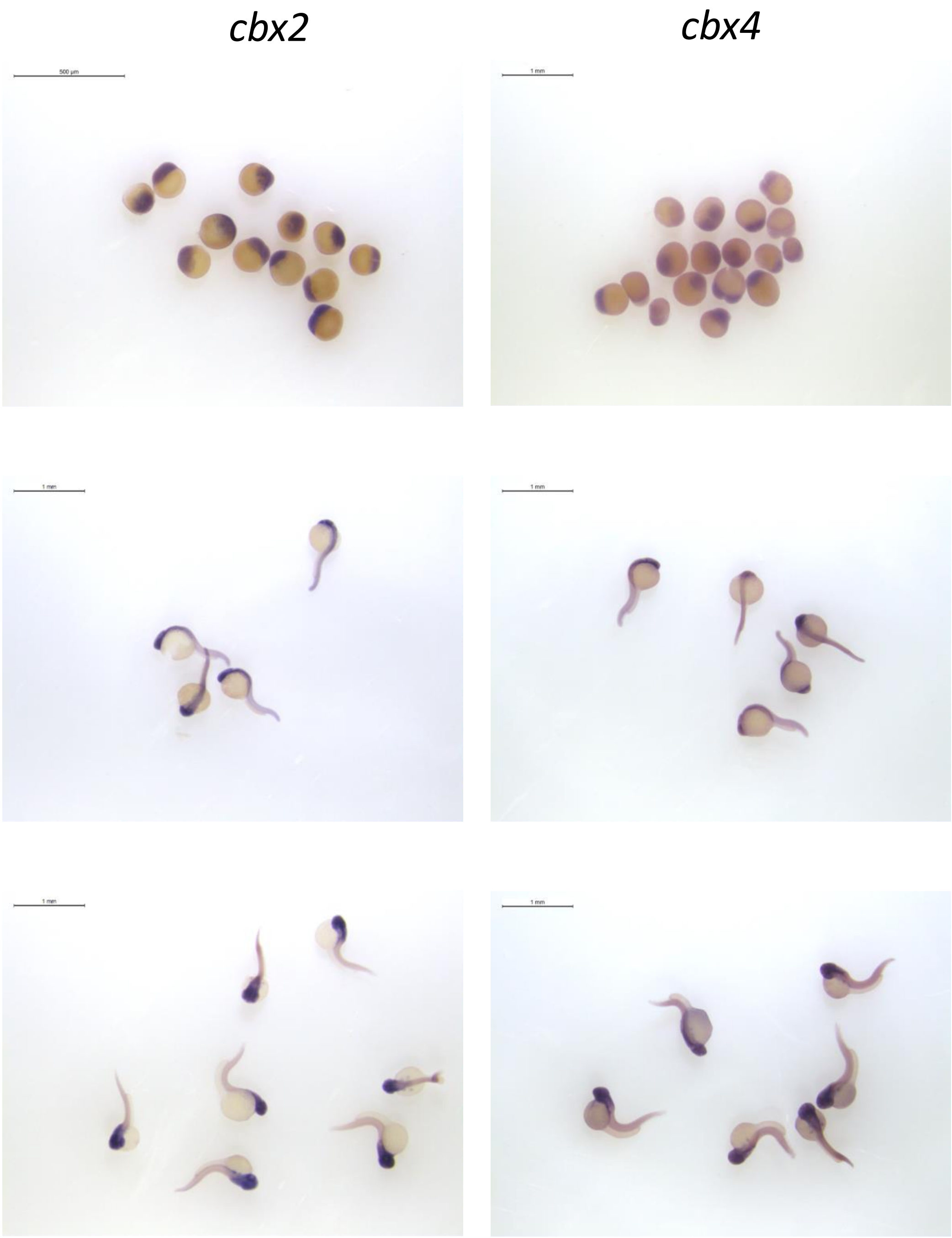
cbx2
cbx4

cbx6a *cbx6b*

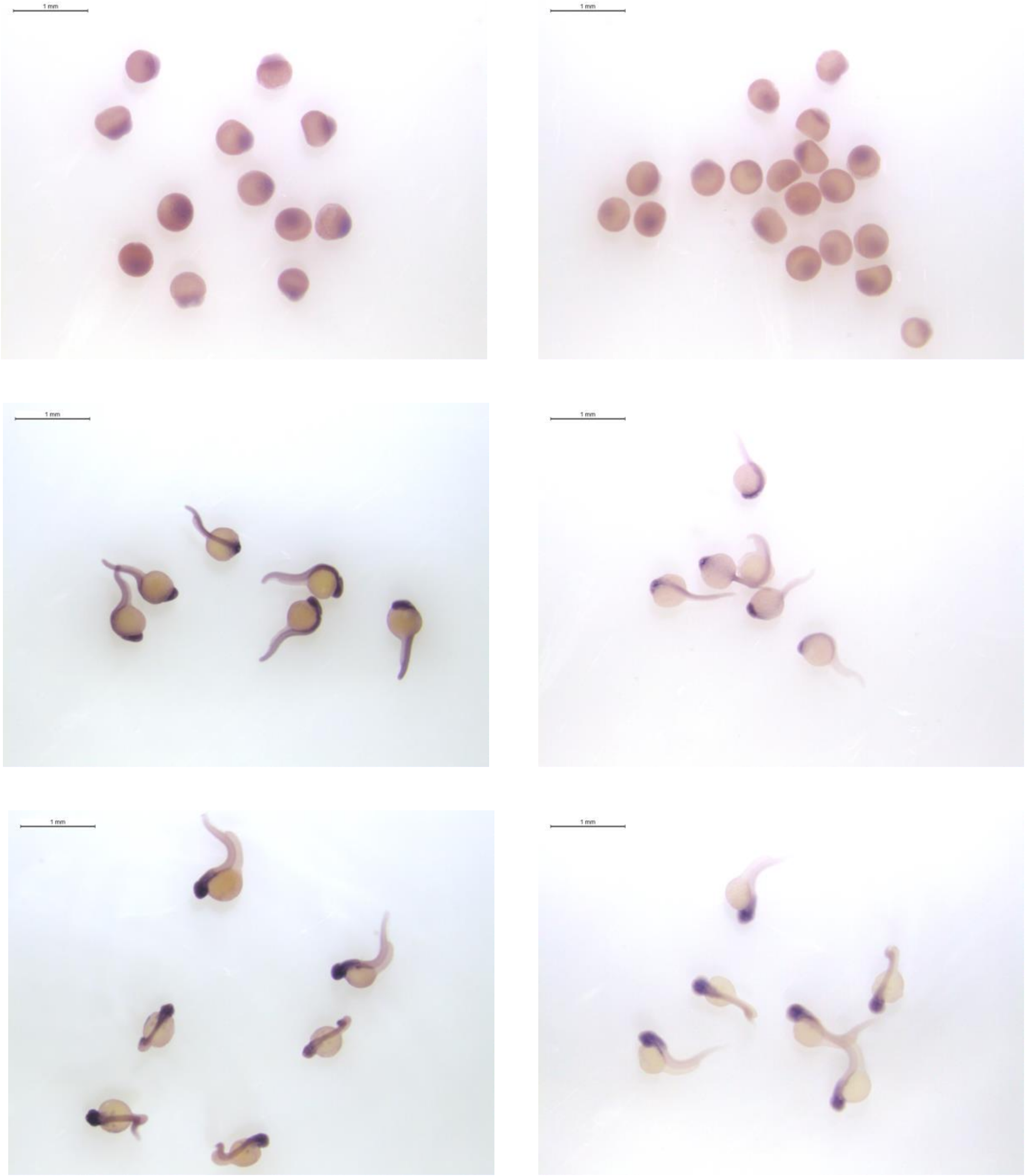

cbx7a *cbx7b*

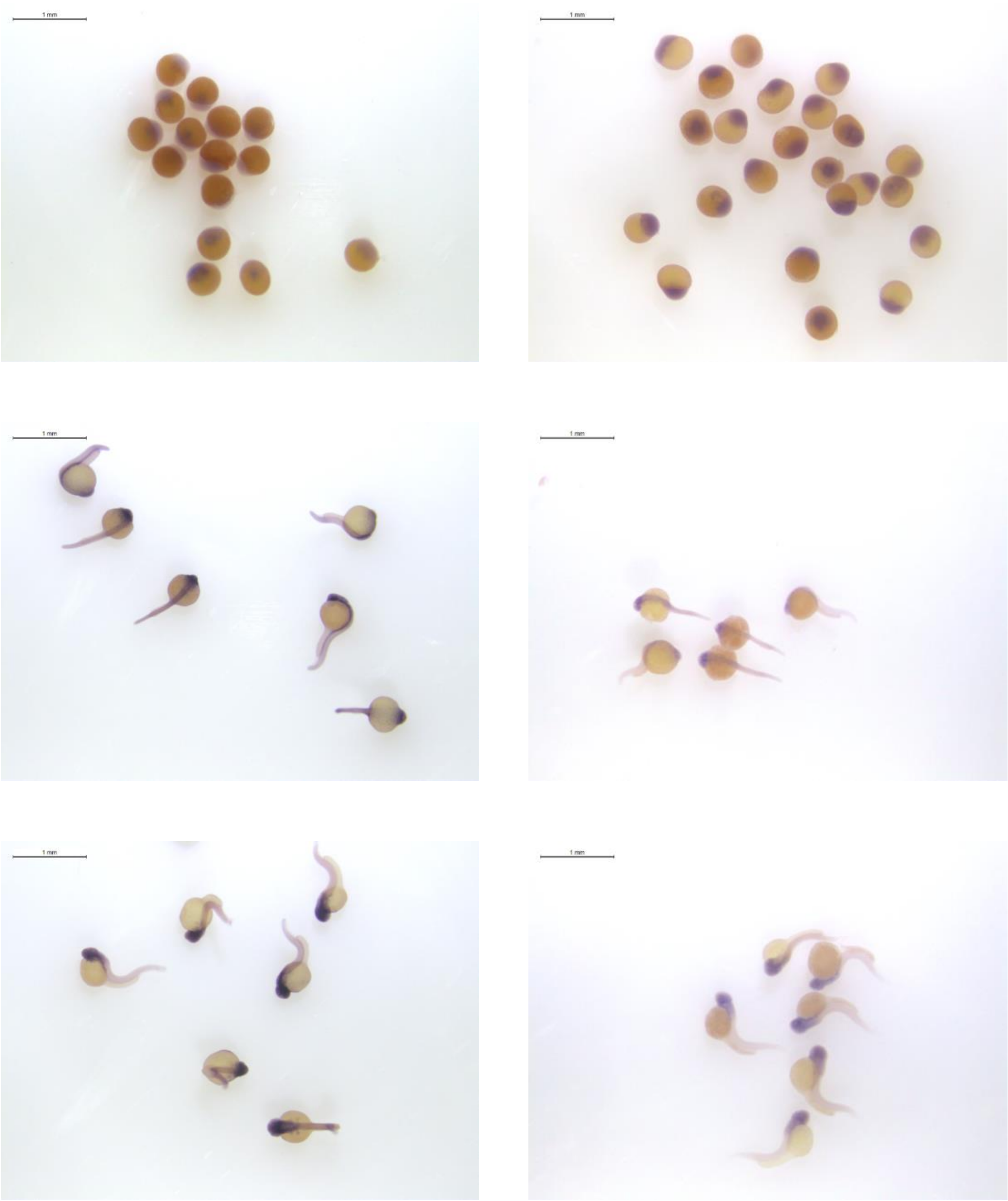

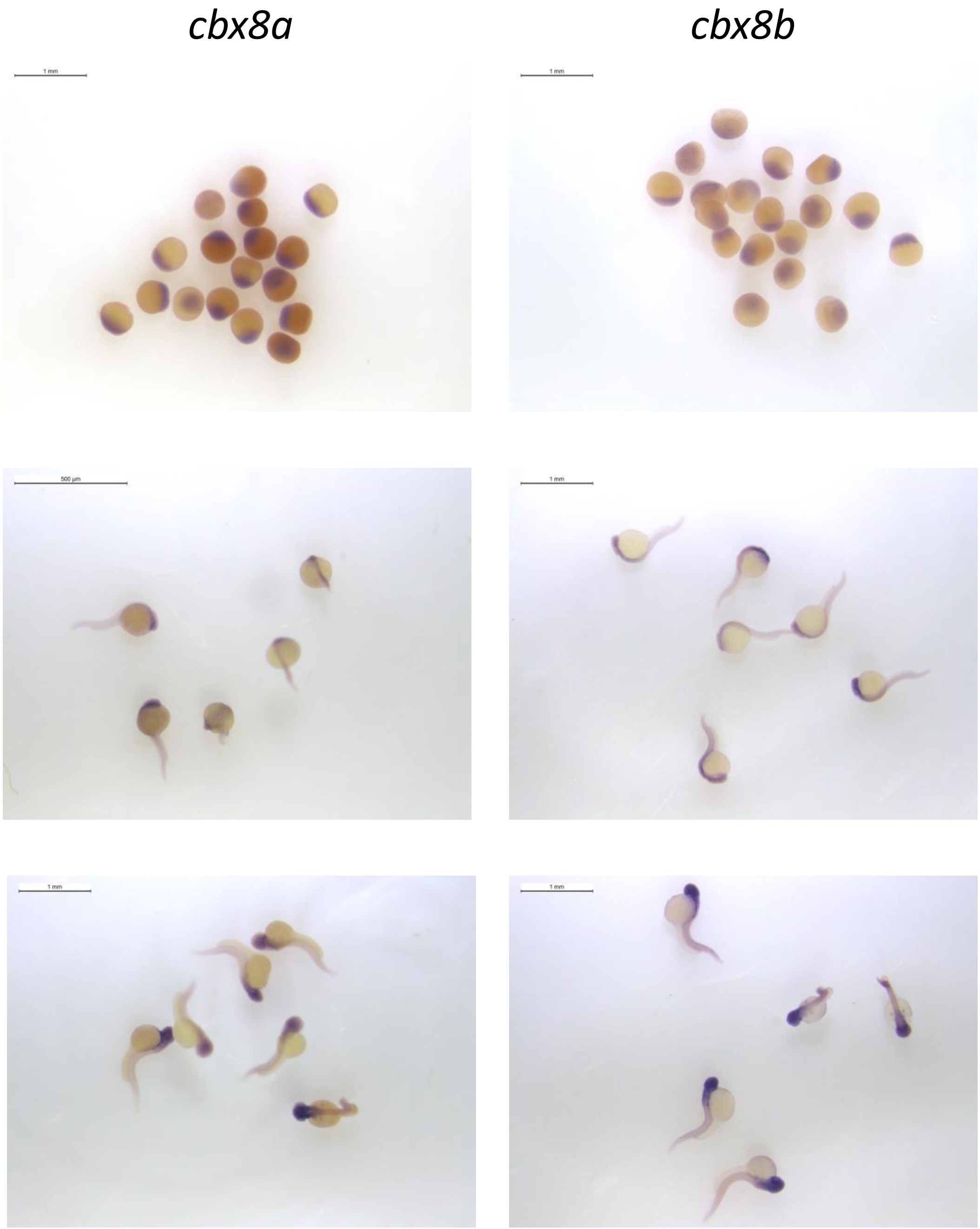

Supplementary File S5. ***In situ hybridization on groups of zebrafish embryos at the 1-cell stage (upper panel), 24 hpf (middle panel) and 48 hpf (lower panel) for cbx2, cbx4, cbx6a, cbx6b, cbx7a, cbx7b, cbx8a and cbx8b.*** Scale bar is 1 mm.
