# Supplementary material for: The Polycomb Orthologues in Teleost Fishes and Their Expression in the Zebrafish Model
Source: Genes (Basel). 2020 Mar 27;11(4):362. doi: 10.3390/genes11040362 (PMC7230241; doi:10.3390/genes11040362)
Supplement: Supplementary file 1 [file genes-11-00362-s001.zip › Supp Material/Supp File S6.pdf]

cbx2 (128bp)

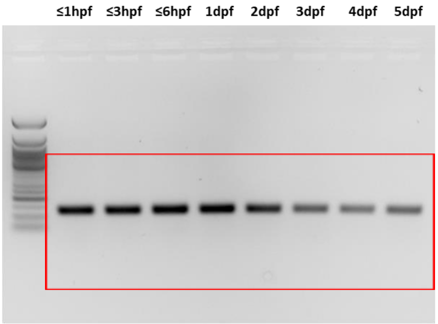

08.01.2020 - RT (600ng); RNA from first extraction

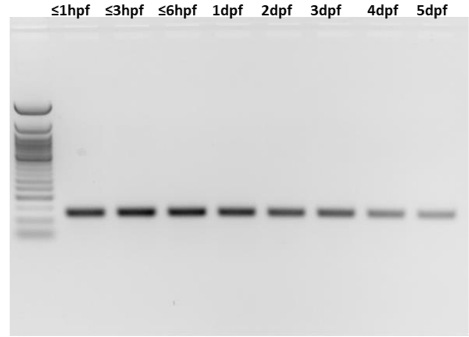

30.12.2019 - RT (1µg); RNA from first extraction

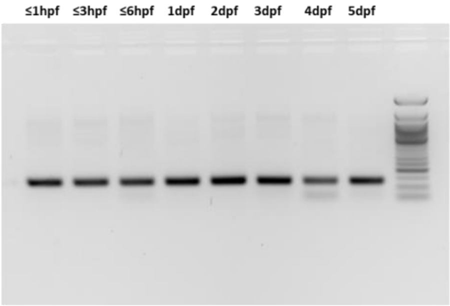

23.12.2019 - RT (700ng); RNA from second extraction

cbx4a (130bp)

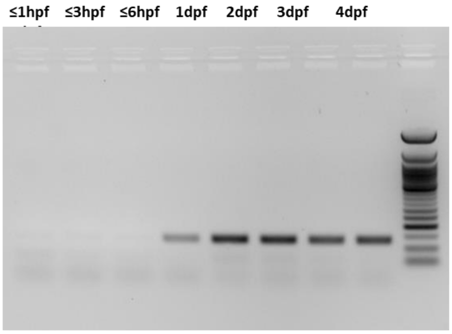

08.01.2020 - RT (600ng); RNA from first extraction

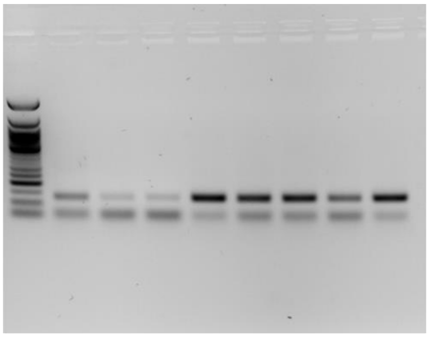

30.12.19 - RT (700ng); RNA from second extraction

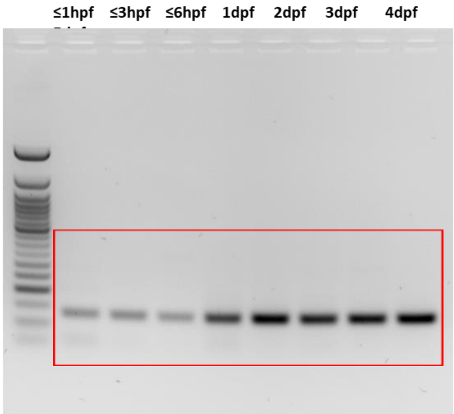

13.01.2020 - RT (600ng); RNA from first extraction

cbx6a (167bp)

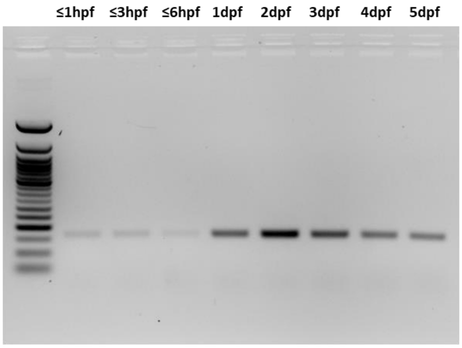

09.01.2020 - RT (600ng); RNA from first extraction

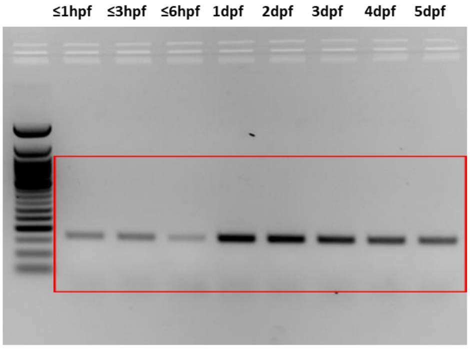

08.01.2020 - RT (700ng); RNA from second extraction

cbx6b (163bp)

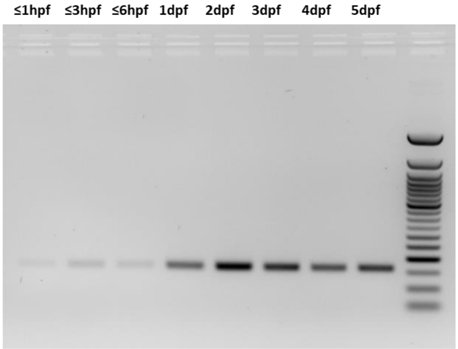

09.01.2020 - RT (600ng); RNA from first extraction

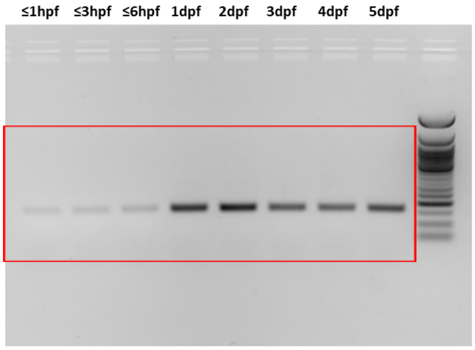

08.01.2020 - RT (700ng); RNA from second extraction

cbx7a (243bp)

≤1hpf ≤3hpf ≤6hpf 1dpf 2dpf 3dpf 4dpf 5dpf

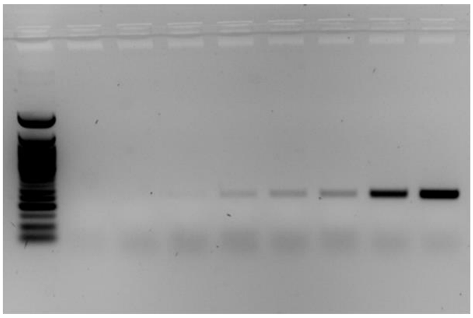

08.01.2020 - RT (700ng); RNA from second extraction

≤1hpf ≤3hpf ≤6hpf 1dpf 2dpf 3dpf 4dpf 5dpf

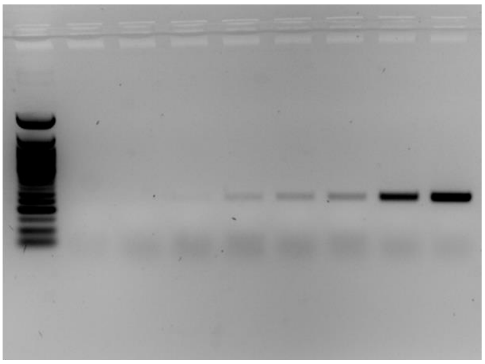

09.01.2020 - RT (600ng); RNA from first extraction

≤1hpf ≤3hpf ≤6hpf 1dpf 2dpf 3dpf 4dpf 5dpf

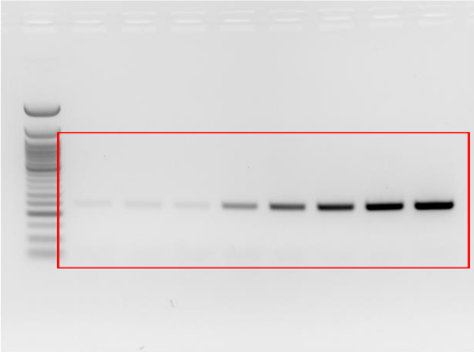

10.01.2020 - RT (600ng); RNA from first extraction
(35 cycles)

cbx7b (125bp)

≤1hpf ≤3hpf ≤6hpf 1dpf 2dpf 3dpf 4dpf 5dpf

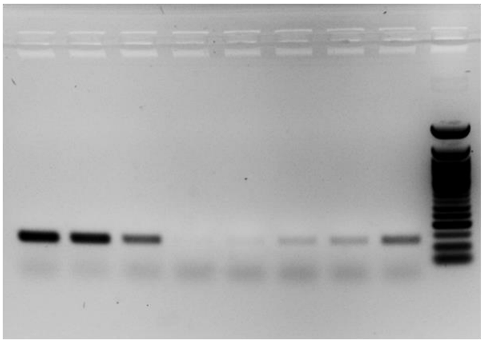

08.01.2020 - RT (700ng); RNA from second extraction

≤1hpf ≤3hpf ≤6hpf 1dpf 2dpf 3dpf 4dpf 5dpf

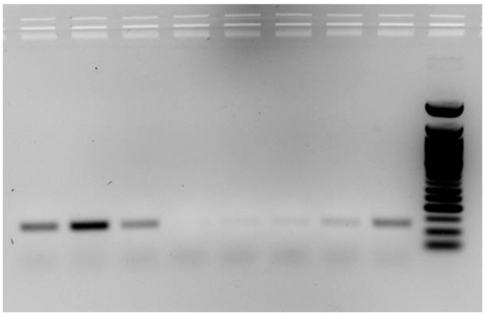

09.01.2020 - RT (600ng); RNA from first extraction

≤1hpf ≤3hpf ≤6hpf 1dpf 2dpf 3dpf 4dpf 5dpf

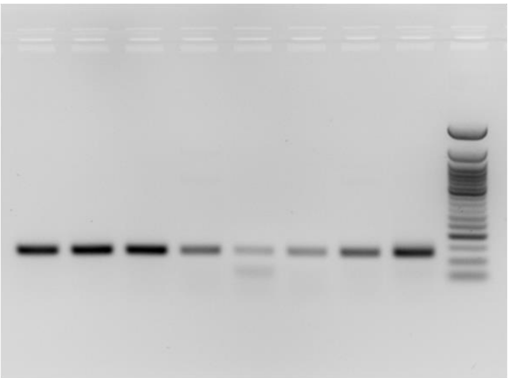

10.01.2020 - RT (600ng); RNA from first extraction
(35cycles)

≤1hpf ≤3hpf ≤6hpf 1dpf 2dpf 3dpf 4dpf 5dpf

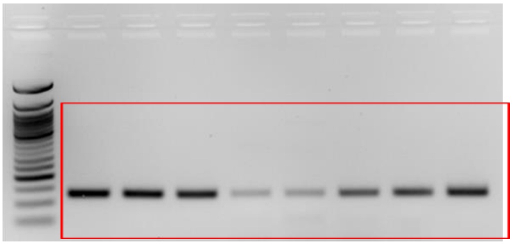

10.01.2020 - RT (700ng); RNA from second extraction
(35 cycles)

cbx8a (147bp)

≤1hpf ≤3hpf ≤6hpf 1dpf 2dpf 3dpf 4dpf 5dpf

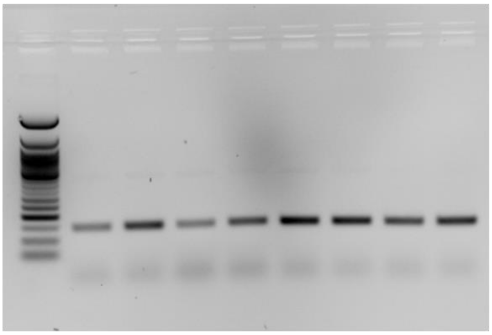

08.01.20 - RT (600ng); RNA from first extraction

≤1hpf ≤3hpf ≤6hpf 1dpf 2dpf 3dpf 4dpf 5dpf

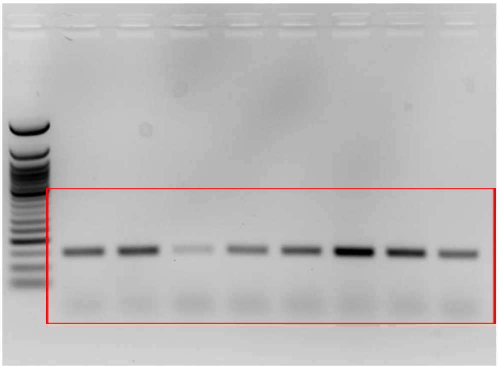

09.01.20 - RT (1µg); RNA from first extraction

≤1hpf ≤3hpf ≤6hpf 1dpf 2dpf 3dpf 4dpf 5dpf

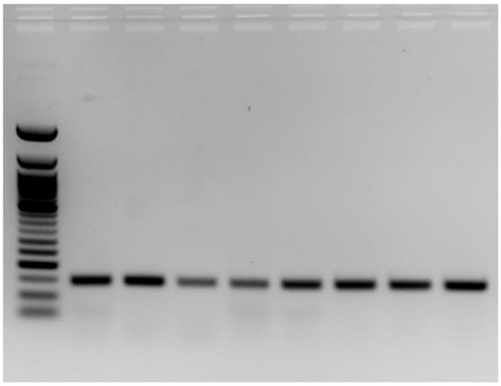

31.12.2019 - RT (700ng); RNA from second extraction

cbx8b (259bp)

≤1hpf ≤3hpf ≤6hpf 1dpf 2dpf 3dpf 4dpf 5dpf

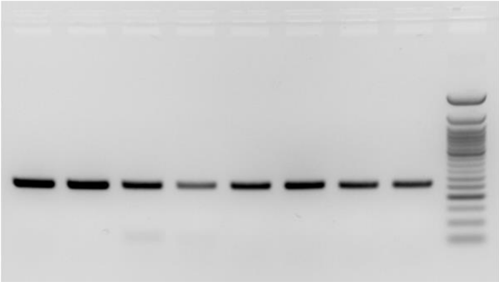

08.01.2020 - RT (600ng); RNA from first extraction

≤1hpf ≤3hpf ≤6hpf 1dpf 2dpf 3dpf 4dpf 5dpf

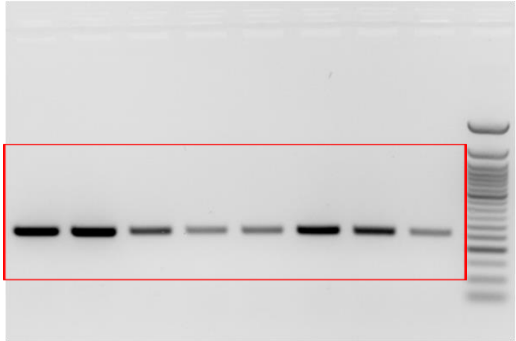

09.01.2020 - RT (1µg); RNA from first extraction

≤1hpf ≤3hpf ≤6hpf 1dpf 2dpf 3dpf 4dpf 5dpf

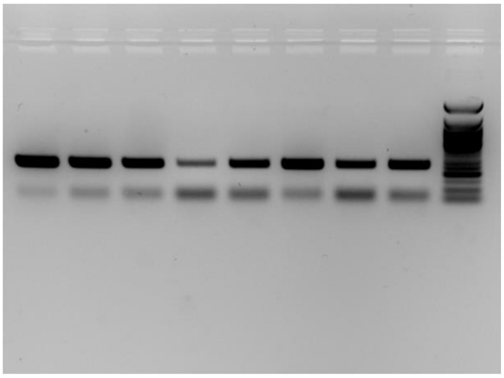

31.12.2019 - RT (700ng); RNA from second extraction

cbx2 (128bp)

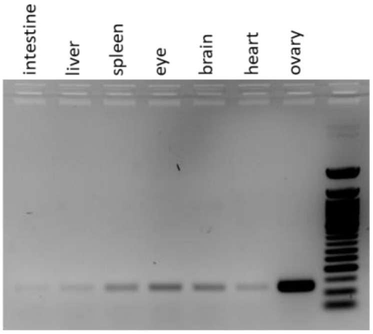

RT (1µg) 09.12.19; PCR 30 cycles 09.01.20

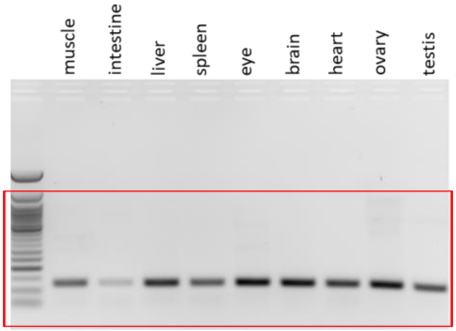

RT (500ng) 09/2019; PCR 35 cycles 14.01.20

cbx4a (130bp)

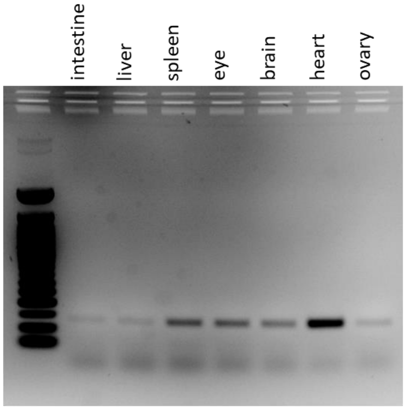

RT (1µg) 09.12.19; PCR 30 cycles 10.01.20

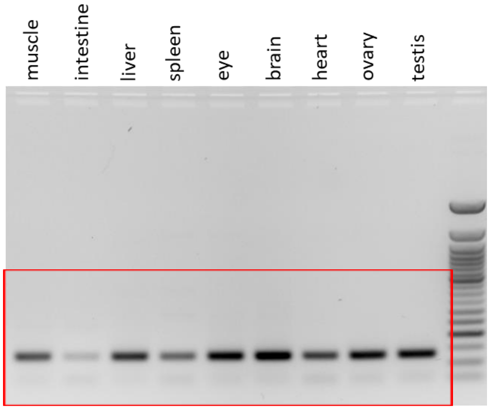

RT (500ng) 09/2019; PCR 35 cycles 14.01.20

cbx6a (167bp)

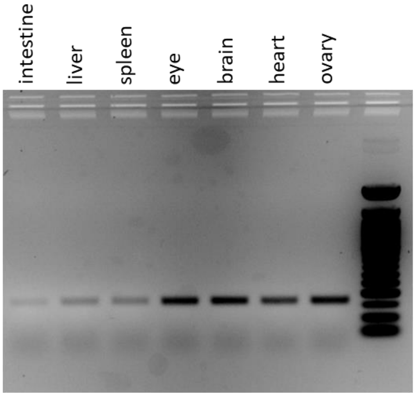

RT (1µg) 09.12.19; PCR 30 cycles 10.01.20

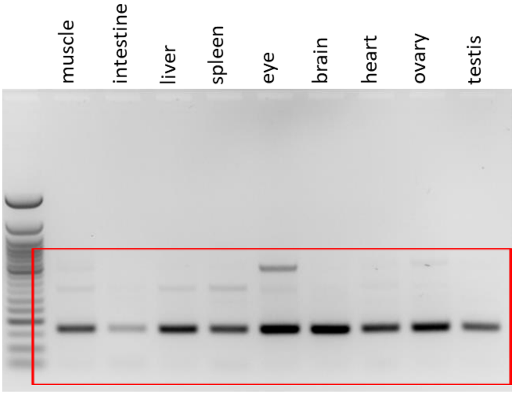

RT (500ng) 09/2019; PCR 35 cycles 14.01.20

cbx6b (163bp)

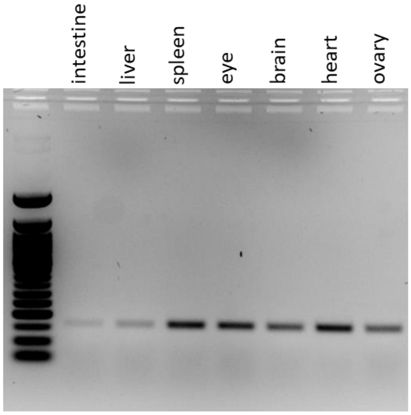

RT (1µg) 09.12.19; PCR 30 cycles 10.01.20

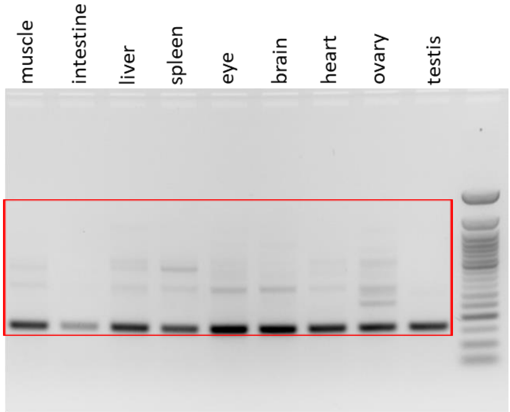

RT (500ng) 09/2019; PCR 35 cycles 14.01.20

cbx7a (243bp)

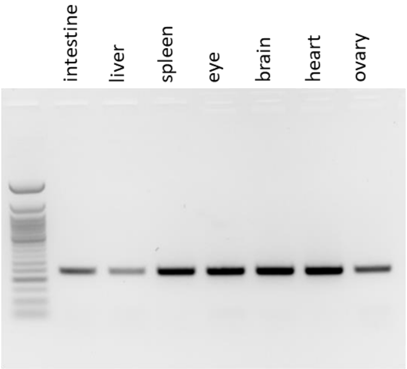

RT (1µg) 09.12.19; PCR 35 cycles 10.01.20

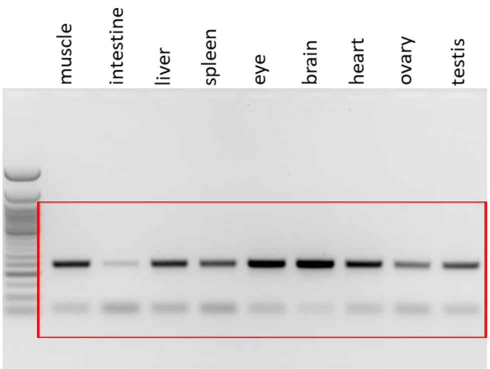

RT (500ng) 09/2019; PCR 35 cycles 14.01.20

cbx7b (125bp)

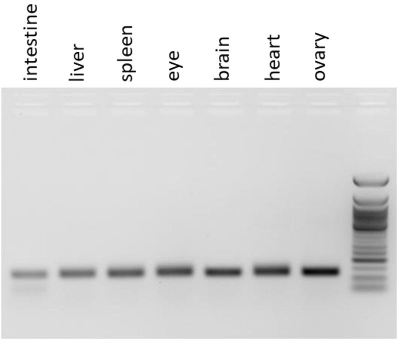

RT (1µg) 09.12.19; PCR 35 cycles 10.01.20

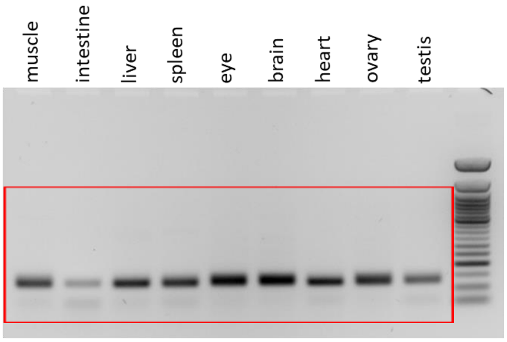

RT (500ng) 09/2019; PCR 35 cycles 14.01.20

cbx8a (147bp)

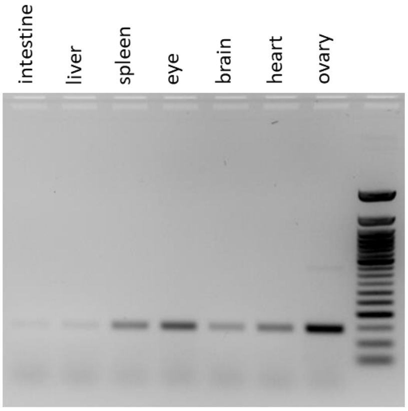

RT (1µg) 09.12.19; PCR 30 cycles 10.01.20

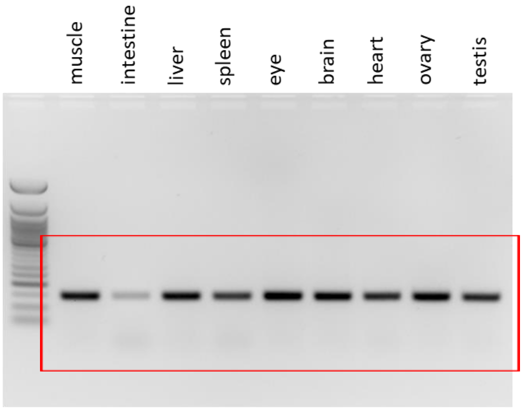

RT (500ng) 09/2019; PCR 35 cycles 14.01.20

cbx8b (259bp)

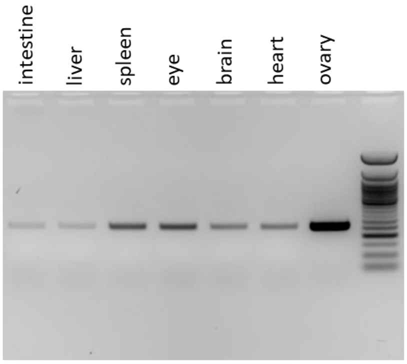

RT (1µg) 09.12.19; PCR 30 cycles 14.01.20

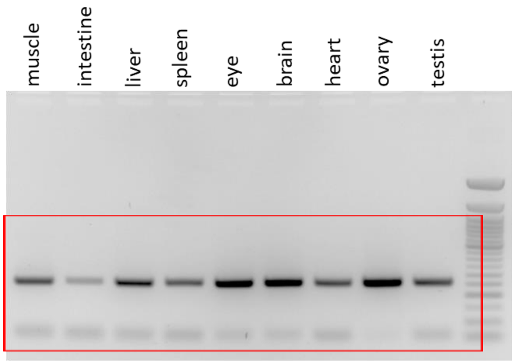

RT (500ng) 09/2019; PCR 35 cycles 14.01.20

b-actin (reference gene; 443bp)

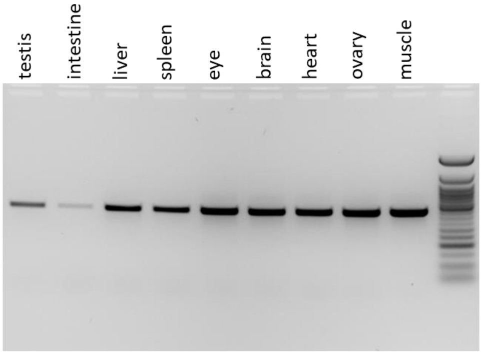

RT (500ng) 09/2019; PCR 25 cycles 14.01.20

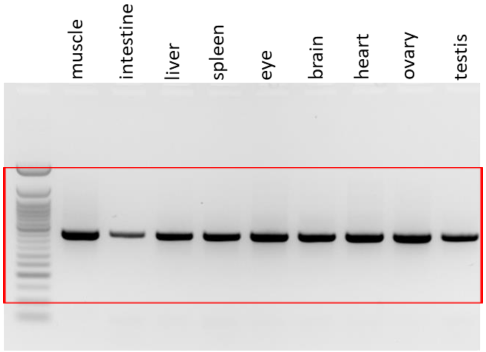

RT (500ng) 09/2019; PCR 30 cycles 14.01.20

Supplementary File S6 Original RT-PCR gels. The red boxes indicate the gel from which the panels of Figure 8 originate from.
