# Supplementary material for: The Polycomb Orthologues in Teleost Fishes and Their Expression in the Zebrafish Model
Source: Genes (Basel). 2020 Mar 27;11(4):362. doi: 10.3390/genes11040362 (PMC7230241; doi:10.3390/genes11040362)
Supplement: Supplementary file 1 [file genes-11-00362-s001.zip › Supp Material/Supp Table S1.pdf]

Supplementary Table S1: The teleost Pc orthologues

		CBX2	CBX4	CBX6	CBX7	CBX8
**Dre**	Zebrafish (*Danio rerio*)	cbx2 (Gene ID: 327291)	cbx4 (Gene ID: 573110)	cbx6a (Gene ID: 799294)<br>cbx6b (ENSDARG00000097543)	cbx7a (Gene ID: 550551)<br>cbx7b (Gene ID: 101882803)	cbx8a (Gene ID: 100150672)<br>cbx8b (Gene ID: 799361)
**Cca**	Common carp (*Cyprinus carpio*)	LOC109087174<br>LOC109046180	cbx4 (Gene ID: 109102575)<br>LOC109092326<br>LOC109075587°	LOC109066300<br>LOC109066296<br>LOC109095357<br>LOC109053813°	LOC109100093<br>LOC109076671<br>LOC109061103	LOC109102576<br>LOC109092325<br>LOC109075586°
**Cau**	Goldfish (*Carassius auratus*)	LOC113055003<br>LOC113067828<br>LOC113067392	cbx4 (Gene ID: 113050689)<br>LOC113098321<br>LOC113052031<br>LOC113046661	LOC113065021<br>LOC113039859<br>LOC113046746<br>LOC113047918	LOC113047419<br>LOC113046823<br>LOC113111712<br>LOC113056054	LOC113052035<br>LOC113046660<br>LOC113098307<br>LOC113050690
**Sgr**	Golden-line barbel (*Sinocyclocheilus grahami*)	LOC107571836<br>LOC107601561*	cbx4 (Gene ID: 107591927)<br>LOC107553295<br>LOC107562304<br>LOC107559247	LOC107602318<br>LOC107585029<br>LOC107602676<br>LOC107576933*	LOC107584093<br>LOC107554058<br>LOC107602332	LOC107578989<br>LOC107559248<br>LOC107601979 / LOC107591932<br>LOC107553294
**Srh**	犀角金线鲃 (*Sinocyclocheilus rhinocerous)*	LOC107718939<br>LOC107710978	cbx4 (Gene ID: 107710281)<br>LOC107758118<br>LOC107736694*/LOC107728779*<br>LOC107710010	LOC107720374<br>LOC107717667<br>LOC107710048<br>LOC107740604	LOC107734182<br>LOC107709653<br>LOC107748788<br>LOC107723608	LOC107758117<br>LOC107728583<br>LOC107710282<br>LOC107710028
**San**	安水金线鲃 (*Sinocyclocheilus anshuiensis*)	LOC107696361<br>LOC107695690	cbx4 (Gene ID: 107701204)<br>LOC107669529<br>LOC107690020<br>LOC107670662	LOC107667890<br>LOC107704552<br>LOC107690738<br>LOC107662367	LOC107695130<br>LOC107672137<br>LOC107692056<br>LOC107660863	cbx8 (Gene ID: 107701205)<br>LOC107669528<br>LOC107670683<br>LOC107689994
**Cva**	Sheepshead minnow (*Cyprinodon variegatus*)	LOC107089316<br>LOC107082907	cbx4 (Gene ID: 107085514)<br>LOC107092841	cbx6 (Gene ID: 107090076)	LOC107092875<br>LOC107093081	LOC107092814<br>LOC107085518
**Xma**	Platyfish (*Xiphophorus maculatus*)	LOC102229509<br>LOC102223386	cbx4 (Gene ID: 102230727)<br>LOC102232224	cbx6 (Gene ID: 102234048)	LOC102225821<br>LOC102232562	cbx8 (Gene ID: 102230986)<br>LOC102227807
**Pfo**	Amazon molly (*Poecilia formosa*)	LOC103146900<br>LOC103147077	cbx4 (Gene ID: 103133063)<br>LOC103141632	cbx6 (Gene ID: 103154832)	LOC103143382<br>LOC103153429	LOC103133078<br>LOC103141633
**Gaf**	Western mosquitofish (*Gambusia affinis*)	cbx2 (ENSGAFG00000004995)<br>ENSGAFG00000015448	cbx4 (ENSGAFG00000020219)<br>ENSGAFG00000007827	ENSGAFG00000007822	cbx7a (ENSGAFG00000010693)<br>cbx7b (ENSGAFG00000008146)	cbx8a (ENSGAFG00000020216)
**Pre**	Guppy (*Poecilia reticulata*)	cbx2 (Gene ID: 103469046)<br>LOC103481889	cbx4 (Gene ID: 103463659)<br>LOC103469229	cbx6 (Gene ID: 108166384)	LOC103482006<br>LOC103468464	cbx8 (Gene ID: 103463651)<br>LOC103469230
**Xco**	Monterrey platyfish (*Xiphophorus couchianus*)	LOC114160441<br>LOC114152221	cbx4 (Gene ID: 114145263)<br>LOC114160311	cbx6 (Gene ID: 114145658)	LOC114160060<br>LOC114151814	cbx8 (Gene ID: 114145391)<br>LOC114160318
**Pla**	Sailfin molly (*Poecilia latipinna*)	LOC106937812<br>LOC106943510	cbx4 (Gene ID: 106958633)<br>LOC106957183	cbx6 (Gene ID: 106936647)	LOC106961268<br>LOC106961608	LOC106957191<br>LOC106958634
**Pme**	Shortfin molly (*Poecilia mexicana*)	LOC106924748<br>LOC106914323	cbx4 (Gene ID: 106928181)<br>LOC106904416	cbx6 (Gene ID: 106904597)	LOC106910083<br>LOC106933457	LOC106904421<br>LOC106928182
**Nfu**	Turquoise killifish (*Nothobranchius furzeri*)	cbx2 (Gene ID: 107397219)<br>LOC107387235	LOC107376532<br>LOC107395725	cbx6 (Gene ID: 107375179)	LOC107388224<br>LOC107376332	LOC107395726<br>LOC107376533
**Fhe**	Mummichog (*Fundulus heteroclitus*)	cbx2 (Gene ID: 105932784)<br>LOC105937668	cbx4 (Gene ID: 105940177)<br>LOC105933040	cbx6a (ENSFHEG00000010219)	LOC105930863<br>LOC105917993	cbx8 (Gene ID: 105940179)<br>LOC105933049
**Kma**	Mangrove rivulus (*Kryptolebias marmoratus*)	cbx2 (Gene ID: 108237824)<br>LOC108249256	cbx4 (Gene ID: 108245485)<br>LOC108239245	cbx6 (Gene ID: 108231568)	LOC108236253<br>LOC108250439	LOC108228443<br>LOC108239259
**Mze**	Zebra mbuna (*Maylandia zebra*)	LOC101465599	LOC101477503<br>LOC101467610	ENSMZEG00005006699	cbx7 (Gene ID: 101468311)<br>LOC101477848	cbx8 (Gene ID: 101477208)<br>LOC101467917
**Oni**	Tilapia (*Oreochromis niloticus*)	cbx2 (Gene ID: 100704060)<br>LOC100695490	cbx4 (Gene ID: 100712470)<br>ENSONIG00000017179	cbx6 (Gene ID: 102077022)	cbx7 (Gene ID: 102076083)<br>LOC100700457	cbx8 (Gene ID: 100712202)<br>ENSONIG00000017182
**Hbu**	Burton's mouthbrooder (*Haplochromis burtoni*)	LOC102307411<br>LOC102302910	cbx4 (Gene ID: 102295423)<br>LOC102309824	cbx6 (Gene ID: 102311013)	cbx7 (Gene ID: 102304092)<br>LOC102302921	cbx8 (Gene ID: 102295731)<br>LOC102309719* / LOC102309508
**Nbr**	Princess of Burundi (*Neolamprologus brichardi*)	LOC102797064<br>LOC102782953	LOC102794988<br>LOC102784839	LOC102784197	LOC102778087<br>LOC102783421	LOC102790274<br>LOC102784560

**Pny**	Makobe Island cichlid (*Pundamilia nyererei*)	cbx2 (Gene ID: 102192419)	LOC102215081<br>LOC102204981	cbx6 (Gene ID: 102193150)	cbx7 (Gene ID: 102210279)<br>LOC102212884	cbx8 (Gene ID: 102214806)<br>LOC102195395*
**Aca**	Eastern happy (*Astatotilapia calliptera*)	cbx2 (Gene ID:113020225)<br>LOC113027934	LOC113023624<br>LOC113020834	cbx6 (Gene ID:113024612)	LOC113027596<br>LOC113021152	cbx8 (Gene ID:113023623)<br>LOC113020844
**Aci**	Midas cichlid (*Amphilophus citrinellus*)	cbx2 (ENSACIG00000010758)<br>ENSACIG00000023237	ENSACIG00000019976*<br>ENSACIG00000021421*	cbx6a (ENSACIG00000003935*)	ENSACIG00000010051*<br>ENSACIG00000021398*	cbx8a (ENSACIG00000021387)<br>ENSACIG00000019984*
**Tru**	Japanese puffer (*Takifugu rubripes*)	LOC101075336<br>LOC101071244*	LOC101076082<br>LOC101075073	cbx6 (Gene ID: 101072963)	LOC101070872<br>LOC101079131	cbx8 (Gene ID: 101076311)<br>LOC101068461
**Tni**	Tetraodon (*Tetraodon nigroviridis*)	cbx2 (ENSTNIG00000011276)<br>ENSTNIG00000003572	cbx4 (ENSTNIG00000017994)<br>ENSTNIG00000004608	cbx6a (ENSTNIG00000018842)	cbx7a (ENSTNIG00000006270)<br>cbx7b (ENSTNIG00000012945)	Cbx8a (ENSTNIG00000017995)<br>ENSTNIG00000004609
**Mmo**	Ocean sunfish (*Mola mola*)	cbx2 (ENSMMOG00000020204*)<br>ENSMMOG00000009201	ENSMMOG00000006960*<br>ENSMMOG00000007637*	cbx6a (ENSMMOG00000011204)	cbx7b (ENSMMOG00000003559)<br>ENSMMOG00000002654*	cbx8a (ENSMMOG00000007636)
**Lcr**	Large yellow croaker (*Larimichthys crocea*)	cbx2 (Gene ID: 104923389)<br>LOC104928297	cbx4 (Gene ID: 104921555)<br>LOC104918816	cbx6 (Gene ID: 104925448)	LOC104932065<br>LOC104918881	cbx8 (Gene ID: 104921554)<br>LOC113747116
**Pfl**	Yellow perch (*Perca flavescens*)	cbx2 (Gee ID: 114569523)<br>LOC114548436	cbx4 (Gene ID: 114546081)<br>LOC114569420[£]	cbx6 (Gene ID: 114569708)	cbx7 (Gene ID: 114548596)<br>LOC114570306	cbx8 (Gene ID: 114569533)<br>LOC114569420[£]
**Sdu**	Greater amberjack (*Seriola dumerili*)	LOC111237087<br>LOC111217430	cbx4 (Gene ID: 111218007)<br>LOC111227040	cbx6 (Gene ID: 111216565)	cbx7 (Gene ID: 111219914)<br>LOC111230359	cbx8 (Gene ID: 111217943)<br>LOC111227041
**Sla**	Yellowtail amberjack (*Seriola lalandi dorsalis*)	LOC111655533*<br>LOC111664318	LOC111667573<br>LOC111655477	LOC111673425[$]	LOC111646103<br>LOC111647569	cbx8 (Gene ID: 111655455)<br>LOC111667559
**Lbe**	Ballan wrasse (*Labrus bergylta*)	LOC114919638<br>LOC109989300°	LOC109999250<br>LOC109985836	cbx6 (Gene ID: 109991257)	cbx7 (Gene ID: 109992249)<br>LOC110004121	cbx8 (Gene ID: 109999244)<br>LOC109985837
**Cgo**	Channel bull blenny (*Cottoperca gobio*)	LOC115005214<br>LOC115012765	cbx4 (Gene ID: 115013642)<br>LOC115012777	cbx6 (Gene ID: 115005610*)	LOC115012745<br>LOC115024515	cbx8 (Gene ID: 115013830)<br>LOC115012434
**Ena**	Live sharksucker (*Echeneis naucrates*)	cbx2 (Gene ID: 115047492)<br>LOC115059891	LOC115047550<br>LOC115044326	cbx6 (Gene ID: 115044595)	cbx7 (Gene ID: 115060227)<br>LOC115047474	LOC115045432<br>LOC115047560
**Pra**	Indian glassy fish (*Parambassis ranga*)	cbx2 (Gene ID: 11444058)<br>LOC114452074	cbx4 (Gene ID: 114436730)<br>LOC114439615	cbx6 (Gene ID: 114439322)	LOC114437794<br>LOC114439674	cbx8 (Gene ID: 114436737)<br>LOC114439616
**Lca**	Barramundi perch (*Lates calcarifer*)	LOC108874733<br>LOC108874388	cbx4 (Gene ID: 108884566)<br>LOC108879647	cbx6 (Gene ID: 108887174)	LOC108877652<br>LOC108889108	Cbx8 (Gene ID: 108884564)<br>LOC108879657
**Spa**	Bicolor damselfish (*Stegastes partitus*)	cbx2 (Gene ID: 103356360)<br>LOC103370168	cbx4 (Gene ID: 103372324)<br>LOC103361728	cbx6 (Gene ID: 103368006)	cbx7 (Gene ID: 103376032)<br>LOC103373459	cbx8 (Gene ID: 103372326)<br>LOC103361729
**Apo**	Spiny chromis (*Acanthochromis polyacanthus*)	LOC110965974<br>LOC110948480	cbx4 (Gene ID: 110963665)<br>LOC110961503	cbx6a (ENSAPOG00000013333)	LOC110954475<br>LOC110969764	LOC110961504<br>LOC110963666 / LOC110953509
**Aoc**	Clown anemonefish (*Amphiprion ocellaris*)	LOC111568440<br>LOC111562895	LOC111577364<br>LOC111577320	cbx6a (ENSAOCG00000009696)	cbx7 (Gene ID: 111574404)<br>LOC111570338	LOC111577361<br>LOC111577322
**Ape**	Orange clownfish (*Amphiprion percula*)	cbx2 (ENSAPEG00000019197)<br>ENSAPEG00000010635	cbx4 (ENSAPEG00000011118*)<br>ENSAPEG00000015378<br>ENSAPEG00000011123*	cbx6a (ENSAPEG00000009256)	cbx7a (ENSAPEG00000011936)<br>cbx7b (ENSAPEG00000020659)	cbx8a (ENSAPEG00000011079)<br>ENSAPEG00000015391
**Nco**	Black rockcod (*Notothenia coriiceps*)	cbx2 (Gene ID: 104943133)*<br>LOC104963701	cbx4 (Gene ID: 104965337)[£]<br>LOC104943130*	LOC104966035*	LOC104968319*<br>LOC104958407*	Gene ID: 104965337[£]<br>cbx8 (Gene ID: 104943131)
**Cse**	Tongue sole (*Cynoglossus semilaevis*)	LOC103382231<br>LOC103392533	LOC103384237<br>LOC103393067	cbx6 (Gene ID: 103383217)	LOC103382143<br>LOC103393332	LOC103384236<br>LOC103393068
**Sma**	Turbot (*Scophthalmus maximus*)	cbx2 (ENSSMAG00000012696)	cbx4 (ENSSMAG00000014969)<br>ENSSMAG00000011638	cbx6a (ENSSMAG00000021207*)	cbx7a (ENSSMAG00000006170)<br>cbx7b (ENSSMAG00000006575)	cbx8a (ENSSMAG00000014960)<br>ENSSMAG00000011628
**Ame**	Mexican cavefish (*Astyanax mexicanus*)	cbx2 (Gene ID: 103045429)	cbx4 (Gene ID: 103045489)<br>LOC103031044<br>LOC111194212	LOC103038090<br>cbx6b (ENSAMXG00000031801)	cbx7 (Gene ID: 103045301)<br>LOC103030095	LOC103044980<br>LOC103029671
**Pna**	Red-bellied piranha (*Pygocentrus nattereri*)	cbx2 (Gene ID: 108433500)	cbx4 (Gene ID: 108424718)<br>LOC108432204<br>LOC108410419	cbx6 (Gene ID: 108436630)<br>LOC108413245	cbx7 (Gene ID: 108413508)<br>LOC108441212	cbx8 (Gene ID: 108424812)<br>LOC108432169
**Mar**	Zig-zag eel (*Mastacembelus armatus*)	LOC113135962<br>LOC113141452	cbx4 (Gene ID: 113128646)<br>LOC113141280	cbx6 (Gene ID: 113127975)	LOC113135651<br>LOC113141587	LOC113141197<br>LOC113128878

**Mal**	Swamp eel (*Monopterus albus*)	LOC109962840<br>LOC109969680	cbx4 (Gene ID: 109957911)<br>LOC109969469	cbx6 (Gene ID: 109951608)	LOC109952690<br>LOC109952654	cbx8 (Gene ID: 109958030)<br>LOC109969470
**Ate**	Climbing perch (*Anabas testudineus*)	LOC113156830<br>LOC113158311	cbx4 (Gene ID: 113162586)<br>LOC113155158	cbx6a (ENSATEG00000003501)	cbx7 (Gene ID: 113155821)<br>LOC113152693	cbx8 (Gene ID: 113162656)<br>LOC113155165
**Bsp**	Siamese fighting fish (*Betta splendens*)	LOC114845447<br>LOC114861130	cbx4 (Gene ID: 114846950<br>LOC114860475	cbx6 (Gene ID: 114870514)	LOC114846206<br>LOC114860262	LOC114860488<br>LOC114847134
**Gwi**	Blunt-snouted clingfish (*Gouania willdenowi*)	LOC114481946<br>LOC114468341°	LOC114469338<br>LOC114468810	cbx6 (Gene ID: 114466377)<br>LOC114459779	LOC114467897<br>LOC114481622	cbx8 (Gene ID: 114471454)<br>LOC114468472
**Pma**	*Periophthalmus magnuspinnatus*	ENSPMGG00000018267*<br>ENSPMGG00000007479	cbx4 (ENSPMGG00000020462)<br>ENSPMGG00000010294*	cbx6a (ENSPMGG00000019149)	ENSPMGG00000017778*	ENSPMGG00000010296*
**Sfa**	Jewelled blenny (*Salarias fasciatus*)	LOC115392810<br>LOC115385554<br>LOC115385547	cbx4 (Gene ID: 115390982)<br>LOC115386984	cbx6 (Gene ID: 115389716)	LOC115387617<br>LOC115392998	cbx8 (Gene ID: 115390143)<br>LOC115386985
**Ola**	Medaka (*Oryzia latipes*)	cbx2 (Gene ID: 100125497)<br>LOC101172611	cbx4 (Gene ID: 101158167)<br>LOC101160166	cbx6a (ENSORLG00000025101)	LOC101163772<br>LOC101168624	cbx8 (Gene ID: 101157915)<br>LOC101160413
**Gac**	Stickleback (*Gasterosteus aculeatus*)	cbx2 (ENSGACG00000014410)<br>ENSGACG00000010130	cbx4 (ENSGACG00000011731)<br>ENSGACG00000018219	cbx6a (ENSGACG00000019854)[$]	cbx7a (ENSGACG00000007724)	cbx8a (ENSGACG00000011737)<br>ENSGACG00000018216
**Hco**	Tiger tail seahorse (*Hippocampus comes*)	cbx2 (Gene ID: 109527817)<br>LOC109518595	LOC109527525<br>LOC109522747	cbx6 (Gene ID: 109511478)	cbx7 (Gene ID: 109512097)<br>LOC109527051	LOC109522748<br>LOC109527520
**Pki**	Elephantfish (*Paramormyrops kingsleyae*)	LOC111855945	cbx4 (Gene ID: 111833586)<br>LOC111843199	LOC111845190<br>cbx6b (ENSPKIG00000001275)	cbx7 (Gene ID: 111845235)<br>LOC111846417	cbx8 (Gene ID: 111833462)<br>LOC111843316
**Sfo**	Asian bonytongue (*Scleropages formosus*)	LOC114909229*<br>LOC108932946	cbx4 (Gene ID: 108939836)<br>LOC108937346	CBX6 (ENSSFOG00015032962)<br>LOC108938841	cbx7 (Gene ID: 108939814)	cbx8 (Gene ID: 108939914)<br>LOC108936948
**Gmo**	Cod (*Gadus morhua*)	cbx2 (ENSGMOG00000013005)<br>ENSGMOG00000008628*	cbx4 (ENSGMOG00000011295)<br>ENSGMOG00000003065	ENSGMOG00000009785*[$]	cbx7b (ENSGMOG00000013491)	cbx8a (ENSGMOG00000011305)<br>ENSGMOG00000013071
**Ipu**	Channel catfish (*Ictalurus punctatus)*	cbx2 (Gene ID: 108272989)	cbx4 (Gene ID: 108273333)<br>LOC108260285	LOC108260787<br>LOC108259424	LOC108273000<br>LOC108273945	cbx8 (Gene ID: 108272375)<br>LOC108261002
**Eel**	Electric eel (*Electrophorus electricus*)	LOC113568410	LOC113578871<br>LOC113573270	LOC113569862<br>LOC113585732	LOC113574891<br>LOC113588928	cbx8 (Gene ID: 113578829)<br>LOC113573271
**Cha**	Atlantic herring (*Clupea harengus*)	cbx2 (Gene ID: 105898869)	cbx4 (Gene ID: 105892293)<br>LOC105891371	cbx6 (Gene ID: 105912870)*<br>LOC105903838	LOC105888811<br>LOC105897974	cbx8 (Gene ID: 105892295)<br>LOC116220815
**Dcl**	Denticle herring (*Denticeps clupeoides*)	cbx2 (Gene ID: 114794490)	cbx4 (Gene ID: 114786797)<br>LOC114793848[£]	LOC114786320	cbx7 (Gene ID: 114795196)<br>LOC114793357	cbx8 (Gene ID: 114786798)<br>LOC114793848[£]
**Elu**	Northern pike (*Esox lucius*)	cbx2 (Gene ID: 105022926)<br>LOC105027558	cbx4 (Gene ID: 105021558)<br>LOC105013028	ENSELUG00000020955	cbx7 (Gene ID: 105025077)<br>LOC105025793 /LOC114834378*	cbx8 (Gene ID: 105021557)<br>LOC105013027
**Ssa**	Atlantic salmon (*Salmo salar*)	LOC106612095<br>LOC106586561*<br>LOC106606958<br>LOC106606946<br>LOC106589865	LOC106606687<br>LOC106588430<br>LOC106564893	cbx6 (Gene ID: 106591466)<br>LOC106564427*<br>LOC106595625*<br>LOC106604985°	LOC106608943<br>LOC106589529<br>LOC106607170<br>LOC106601246	cbx8 (Gene ID: 106588425)<br>LOC106564894<br>LOC106606673<br>LOC106601767*<br>LOC106596252*
**Omy**	Rainbow trout (*Oncorhynchus mykiss*)	LOC110538562<br>LOC110486592<br>LOC110502771<br>LOC110499438	cbx4 (Gene ID: 110485970)<br>LOC110493691<br>LOC110486813<br>LOC110486808	cbx6 (Gene ID: 110485591)<br>LOC110536509*[$]	LOC110538378<br>LOC110486408<br>LOC110499099<br>LOC110502860	cbx8 (Gene ID: 110485969)<br>LOC110519423<br>LOC110493689<br>LOC110486816<br>LOC110486815*<br>LOC110486809*
**Oki**	Coho salmon (*Oncorhynchus kisutch*)	LOC109898685<br>LOC109879638<br>LOC109870514<br>LOC109899149	cbx4 (Gene ID: 109865985)<br>LOC109894654<br>LOC109887742<br>LOC109882827	cbx6 (Gene ID: 109884207)	cbx7 (Gene ID: 109899160)<br>LOC109898530<br>LOC109870400	LOC109889501<br>LOC109882830<br>LOC109882828*<br>LOC109866077
**Sal**	Arctic char (*Salvelinus alpinus*)	LOC111977406<br>LOC112077672<br>LOC112073971<br>LOC112070653	LOC111956774<br>LOC111953935<br>LOC111950939*<br>LOC112069120	cbx6 (GeneID: 112069553)	LOC111977976<br>LOC111967894<br>LOC112075019<br>LOC112071830	LOC111956622<br>LOC111954549

**Hhu**	Huchen (*Hucho hucho*)	cbx2 (ENSHHUG00000026817)<br>ENSHHUG00000050943<br>ENSHHUG00000045610<br>ENSHHUG00000028893	cbx4 (ENSHHUG00000045583)<br>ENSHHUG00000007216<br>ENSHHUG00000045521<br>ENSHHUG00000050487	cbx6 (ENSHHUG00000007276)<br>cbx6a (ENSHHUG00000051753)	ENSHHUG00000038356<br>ENSHHUG00000052693<br>cbx7b (ENSHHUG00000042715)<br>ENSHHUG00000046898	cbx8a (ENSHHUG00000045580)<br>ENSHHUG00000007201<br>cbx8b (ENSHHUG00000050484)

NCBI gene symbols in black or Ensembl entries in blue are indicated for 689 genes coding for Pc orthologues identified in 68 teleost species

° indicates a pseudogene

* indicates that gene predictions generate an incomplete cDNA (coding for a protein lacking the entire or part of the chromodomain or the Pc box)

£ indicates that gene predictions generate a cbx4-cbx8 fusion gene

$ indicates that gene predictions generate a fusion gene between cbx6-like and nptxr-like neighboring genes
