# Supplementary material for: The Polycomb Orthologues in Teleost Fishes and Their Expression in the Zebrafish Model
Source: Genes (Basel). 2020 Mar 27;11(4):362. doi: 10.3390/genes11040362 (PMC7230241; doi:10.3390/genes11040362)
Supplement: Supplementary file 1 [file genes-11-00362-s001.zip › Supp Material/Supp Table S2.pdf]

Supplementary Table S2: The teleost cbx3 genes

		CBX3
**Dre**	Zebrafish (*Danio rerio*)	cbx3a (Gene ID: 751689)<br>cbx3b (Gene ID: 567497)
**Cca**	Common carp (*Cyprinus carpio*)	LOC109108755<br>LOC109110995<br>LOC109072345*<br>LOC109111778<br>LOC109049867
**Cau**	Goldfish (*Carassius auratus*)	LOC113116259<br>LOC113060165<br>LOC113120071<br>LOC113062284
**Sgr**	Golden-line barbel (*Sinocyclocheilus grahami*)	LOC107602402<br>LOC107550827<br>LOC107596654<br>LOC107592008
**Srh**	犀角金线鲃 *(Sinocyclocheilus rhinocerous)*	LOC107744695<br>LOC107710359<br>LOC107748667<br>LOC107743152
**San**	安水金线鲃 (*Sinocyclocheilus anshuiensis*)	LOC107688340<br>LOC107684532<br>LOC107685582<br>LOC107656668
**Cva**	Sheepshead minnow (*Cyprinodon variegatus*)	LOC107101725<br>LOC107084325
**Xma**	Platyfish (*Xiphophorus maculatus*)	LOC106699776<br>LOC102224488
**Pfo**	Amazon molly (*Poecilia formosa*)	LOC103145272<br>LOC103150371
**Gaf**	Western mosquitofish (*Gambusia affinis*)	cbx3a (ENSGAFG00000017896)<br>cbx3b (ENSGAFG00000005602)
**Pre**	Guppy (*Poecilia reticulata*)	LOC103472202<br>LOC103478581
**Xco**	Monterrey platyfish (*Xiphophorus couchianus*)	LOC114141930<br>LOC114156510
**Pla**	Sailfin molly (*Poecilia latipinna*)	LOC106945372<br>LOC106950175
**Pme**	Shortfin molly (*Poecilia mexicana*)	LOC106923566<br>LOC106928233
**Nfu**	Turquoise killifish (*Nothobranchius furzeri*)	cbx3 (Gene ID: 107383357)<br>LOC107395330
**Fhe**	Mummichog (*Fundulus heteroclitus*)	LOC105926367<br>LOC105934310
**Kma**	Mangrove rivulus (*Kryptolebias marmoratus*)	cbx3 (Gene ID: 108238543)<br>LOC108241914
**Mze**	Zebra mbuna (*Maylandia zebra*)	cbx3 (Gene ID: 101470889)<br>LOC101483327
**Oni**	Tilapia (*Oreochromis niloticus*)	LOC100694541<br>LOC100694248
**Hbu**	Burton's mouthbrooder (*Haplochromis burtoni*)	cbx3 (Gene ID: 102307347)<br>LOC102308912
**Nbr**	Princess of Burundi (*Neolamprologus brichardi*)	LOC102791258<br>LOC102789869
**Pny**	Makobe Island cichlid (*Pundamilia nyererei*)	cbx3 (Gene ID: 102213635)<br>LOC102197024
**Aca**	Eastern happy (*Astatotilapia calliptera*)	LOC113032446<br>LOC113014873<br>LOC113014635°
**Aci**	Midas cichlid (*Amphilophus citrinellus*)	cbx3a (ENSACIG00000002111)<br>cbx3b (ENSACIG00000016058)
**Tru**	Japanese puffer (*Takifugu rubripes*)	LOC101067559<br>LOC101061787
**Tni**	Tetraodon (*Tetraodon nigroviridis*)	ENSTNIG00000001200<br>ENSTNIG00000018425
**Mmo**	Ocean sunfish (*Mola mola*)	cbx3a (ENSMMOG00000000435)<br>cbx3b (ENSMMOG00000001662)
**Lcr**	Large yellow croaker (*Larimichthys crocea*)	LOC104940580<br>LOC104927770
**Pfl**	Yellow perch (*Perca flavescens*)	cbx3 (Gene ID: 114567991)<br>LOC114557836
**Sdu**	Greater amberjack (*Seriola dumerili*)	LOC111223223<br>LOC111238784
**Sla**	Yellowtail amberjack (*Seriola lalandi dorsalis*)	LOC111673325<br>LOC111652798
**Lbe**	Ballan wrasse (*Labrus bergylta*)	cbx3 (Gene ID: 109994620)<br>LOC109986704
**Cgo**	Channel bull blenny (*Cottoperca gobio*)	LOC115021151<br>LOC115016275
**Ena**	Live sharksucker (*Echeneis naucrates*)	LOC115051003<br>LOC115045440

**Pra**	Indian glassy fish (*Parambassis ranga*)	cbx3 (Gene ID: 114450982)<br>LOC114448349
**Lca**	Barramundi perch (*Lates calcarifer*)	LOC108891497<br>LOC108877463
**Spa**	Bicolor damselfish (*Stegastes partitus*)	cbx3 (Gene ID: 103364037)<br>LOC103364624
**Apo**	Spiny chromis (*Acanthochromis polyacanthus*)	LOC110955790<br>LOC110951430
**Aoc**	Clown anemonefish (*Amphiprion ocellaris*)	LOC111579711<br>LOC111588833
**Ape**	Orange clownfish (*Amphiprion percula*)	cbx3a (ENSAPEG00000019335)<br>cbx3b (ENSAPEG00000010544)
**Nco**	Black rockcod (*Notothenia coriiceps*)	cbx3 (Gene ID: 104949902)<br>LOC104966666
**Cse**	Tongue sole (*Cynoglossus semilaevis*)	LOC103394257<br>LOC103388431
**Sma**	Turbot (*Scophthalmus maximus*)	cbx3a (ENSSMAG00000014860)<br>cbx3b (ENSSMAG00000020282)
**Ame**	Mexican cavefish (*Astyanax mexicanus*)	LOC103046426
**Pna**	Red-bellied piranha (*Pygocentrus nattereri*)	LOC108425284<br>LOC108441518
**Mar**	Zig-zag eel (*Mastacembelus armatus*)	LOC113122625<br>LOC113125891
**Mal**	Swamp eel (*Monopterus albus*)	LOC109968149<br>LOC109967352
**Ate**	Climbing perch (*Anabas testudineus*)	LOC113170961<br>LOC113153869
**Bsp**	Siamese fighting fish (*Betta splendens*)	LOC114865848<br>LOC114843466
**Gwi**	Blunt-snouted clingfish (*Gouania willdenowi*)	LOC114472522<br>LOC114478298
**Pma**	*Periophthalmus magnuspinnatus*	cbx3a (ENSPMGG00000006267)<br>cbx3b (ENSPMGG00000011799)
**Sfa**	Jewelled blenny (*Salarias fasciatus*)	LOC115396622<br>LOC115381000
**Ola**	Medaka (*Oryzia latipes*)	LOC101159942<br>LOC100049459
**Gac**	Stickleback (*Gasterosteus aculeatus*)	cbx3a (ENSGACG00000007069)<br>cbx3b (ENSGACG00000008334)
**Hco**	Tiger tail seahorse (*Hippocampus comes*)	LOC109522111<br>LOC109525650
**Pki**	Elephantfish (*Paramormyrops kingsleyae*)	LOC111846950<br>LOC111838987
**Sfo**	Asian bonytongue (*Scleropages formosus*)	LOC108933414<br>LOC108933440<br>LOC108927377
**Gmo**	Cod (*Gadus morhua*)	cbx3 (Gene ID: 115535606)<br>LOC115554036
**Ipu**	Channel catfish (*Ictalurus punctatus)*	cbx3 (Gene ID: 108271872)<br>cbx3-like (Gene ID: 100528511)
**Eel**	Electric eel (*Electrophorus electricus*)	LOC113571151<br>LOC113580431
**Cha**	Atlantic herring (*Clupea harengus*)	cbx3 (Gene ID: 105907686)<br>LOC105911400
**Dcl**	Denticle herring (*Denticeps clupeoides*)	LOC114797297<br>LOC114790202<br>LOC114796795°
**Elu**	Northern pike (*Esox lucius*)	LOC105023664<br>LOC105018804
**Ssa**	Atlantic salmon (*Salmo salar*)	cbx3 (Gene ID: 100195583)<br>LOC106588845<br>LOC106573104
**Omy**	Rainbow trout (*Oncorhynchus mykiss*)	LOC110506239<br>LOC110496562<br>LOC110487945
**Oki**	Coho salmon (*Oncorhynchus kisutch*)	LOC109878229<br>LOC109907267<br>LOC109903322
**Sal**	Arctic char (*Salvelinus alpinus*)	LOC111955440<br>LOC112070715<br>LOC112079746°
**Hhu**	Huchen (*Hucho hucho*)	ENSHHUG00000039337<br>cbx3a (ENSHHUG00000011830)<br>ENSHHUG00000050410

NCBI gene symbols in black or Ensembl entries in blue are indicated for 151 genes coding for CBX3 orthologues identified in 68 teleost species

° indicates a pseudogene
* indicates that gene predictions generate an incomplete cDNA
