# Supplementary figures and images for: The Polycomb Orthologues in Teleost Fishes and Their Expression in the Zebrafish Model
Source: Genes (Basel). 2020 Mar 27;11(4):362. doi: 10.3390/genes11040362 (PMC7230241; doi:10.3390/genes11040362)

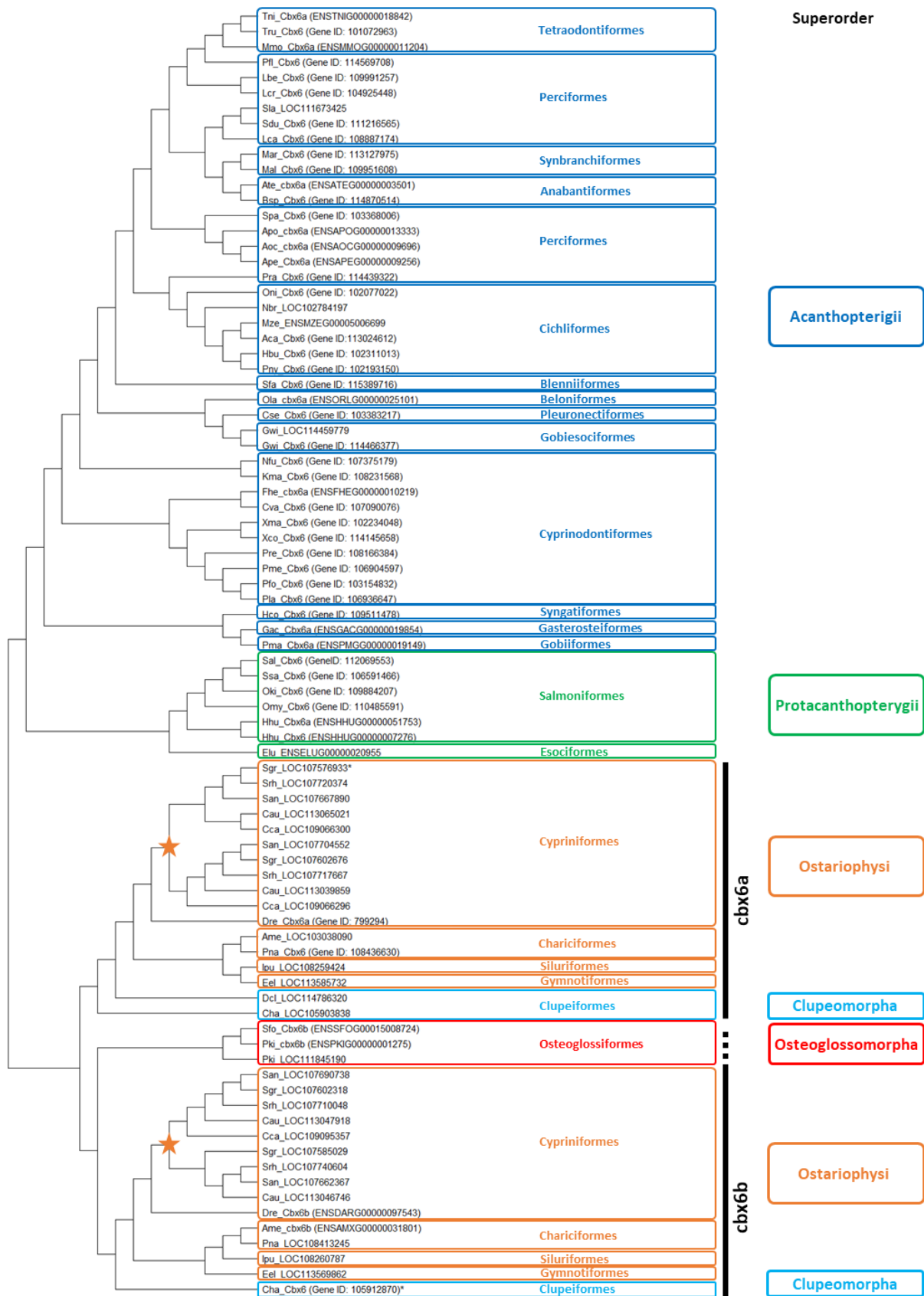

Supplement: Supplementary file 1 [file genes-11-00362-s001.zip › Supp Material/Supp Figure S1.pdf]

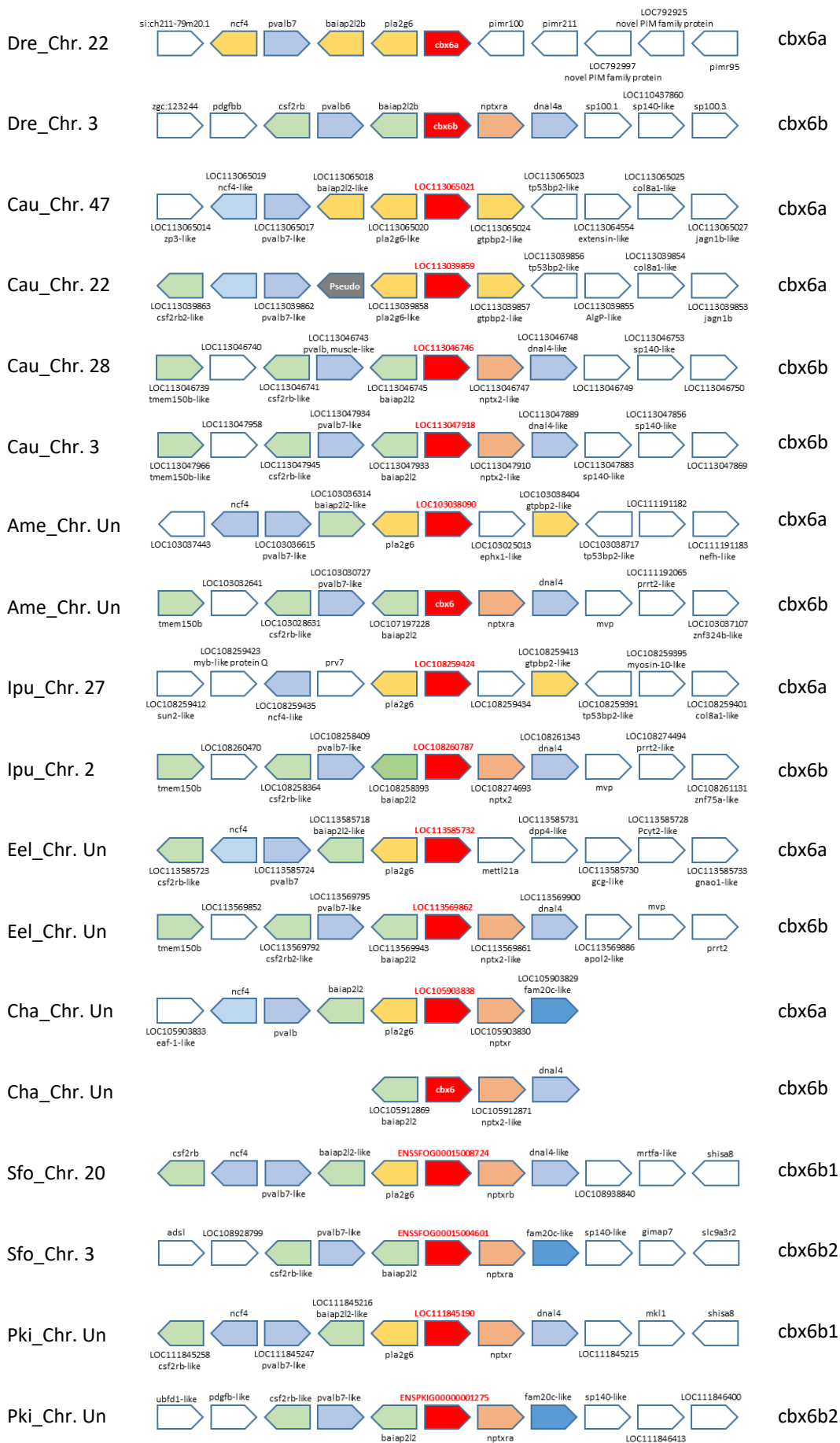

Ostariophysi

Clupeomorpha

Osteoglossomorpha

Supplement: Supplementary file 1 [file genes-11-00362-s001.zip › Supp Material/Supp Figure S3.pdf]

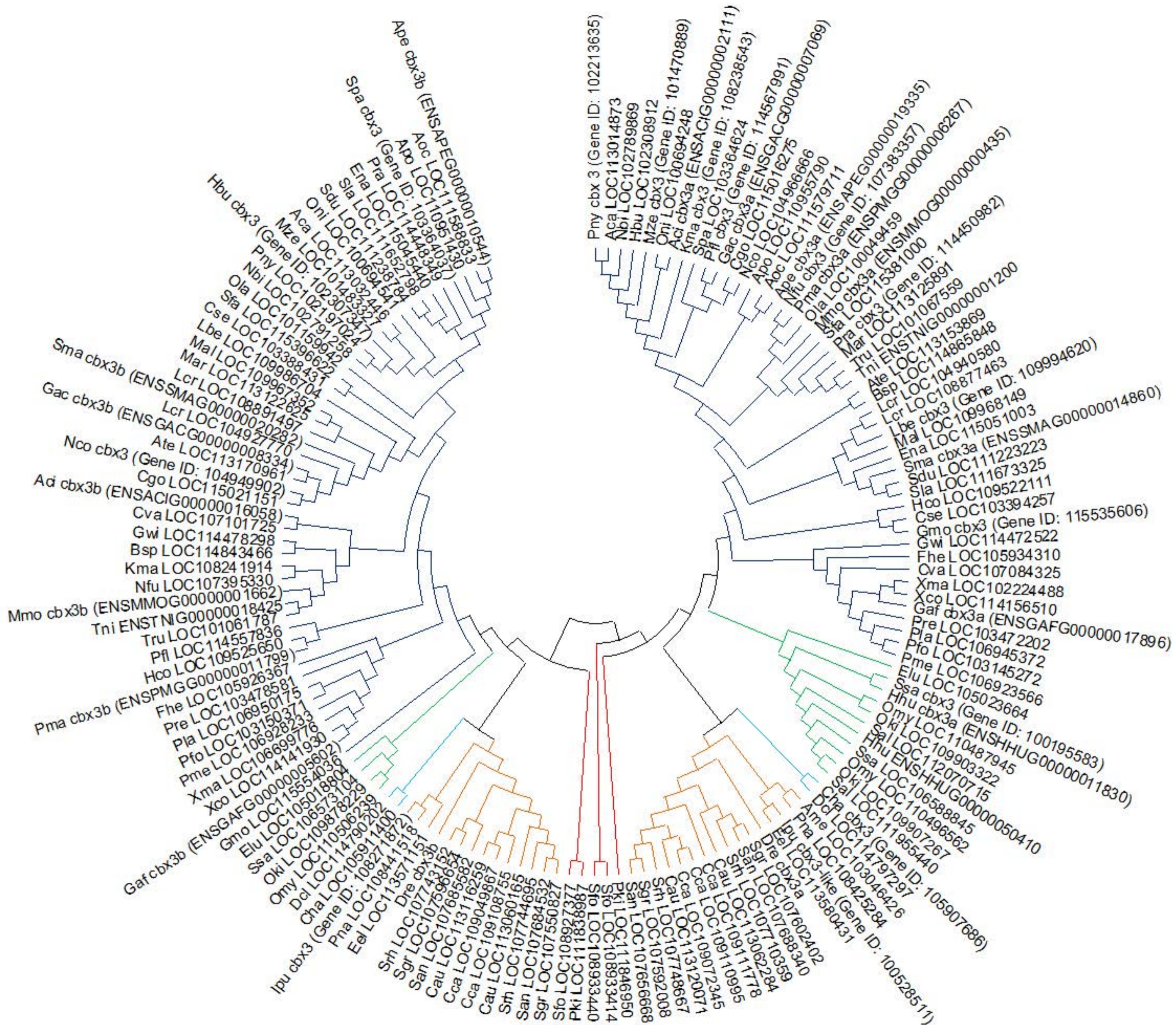

Supplement: Supplementary file 1 [file genes-11-00362-s001.zip › Supp Material/Supp File S4.pdf]
